# A novel route for preparing 5′ cap mimics and capped RNAs: phosphate-modified cap analogues obtained *via* click chemistry†
†Electronic supplementary information (ESI) available: Tables S1–S6 and Fig. S1–S10, experimental procedures, HPLC profiles, NMR and HRMS spectra. See DOI: 10.1039/c6sc02437h



**DOI:** 10.1039/c6sc02437h

**Published:** 2016-08-16

**Authors:** Sylwia Walczak, Anna Nowicka, Dorota Kubacka, Kaja Fac, Przemyslaw Wanat, Seweryn Mroczek, Joanna Kowalska, Jacek Jemielity

**Affiliations:** a Centre of New Technologies , University of Warsaw , Banacha 2c , 02-097 , Warsaw , Poland . Email: jacekj@biogeo.uw.edu.pl; b College of Inter-Faculty Individual Studies in Mathematics and Natural Sciences , University of Warsaw , Banacha 2c , 02-097 , Warsaw , Poland; c Division of Biophysics , Institute of Experimental Physics , Faculty of Physics , University of Warsaw , Zwirki i Wigury 93 , 02-089 , Warsaw , Poland; d Department of Genetics and Biotechnology , Faculty of Biology , University of Warsaw , 02-106 Warsaw , Poland; e Institute of Biochemistry and Biophysics , Polish Academy of Sciences , 02-106 Warsaw , Poland

## Abstract

The significant biological role of the mRNA 5′ cap in translation initiation makes it an interesting subject for chemical modifications aimed at producing useful tools for the selective modulation of intercellular processes and development of novel therapeutic interventions. However, traditional approaches to the chemical synthesis of cap analogues are time-consuming and labour-intensive, which impedes the development of novel compounds and their applications. Here, we explore a different approach for synthesizing 5′ cap mimics, making use of click chemistry (CuAAC) to combine two mononucleotide units and yield a novel class of dinucleotide cap analogues containing a triazole ring within the oligophosphate chain. As a result, we synthesized a library of 36 mRNA cap analogues differing in the location of the triazole ring, the polyphosphate chain length, and the type of linkers joining the phosphate and the triazole moieties. After biochemical evaluation, we identified two analogues that, when incorporated into mRNA, produced transcripts translated with efficiency similar to compounds unmodified in the oligophosphate bridge obtained by traditional synthesis. Moreover, we demonstrated that the triazole-modified cap structures can be generated at the RNA 5′ end using two alternative capping strategies: either the typical co-transcriptional approach, or a new post-transcriptional approach based on CuAAC. Our findings open new possibilities for developing chemically modified mRNAs for research and therapeutic applications, including RNA-based vaccinations.

## Abstract 2

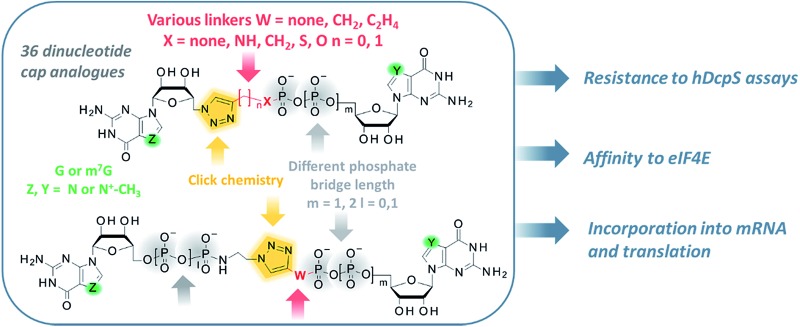
A different approach for synthesizing 5′ cap mimics to yield a novel class of dinucleotide cap analogues containing a triazole ring within the oligophosphate chain.

## Introduction

**Fig. 1:**
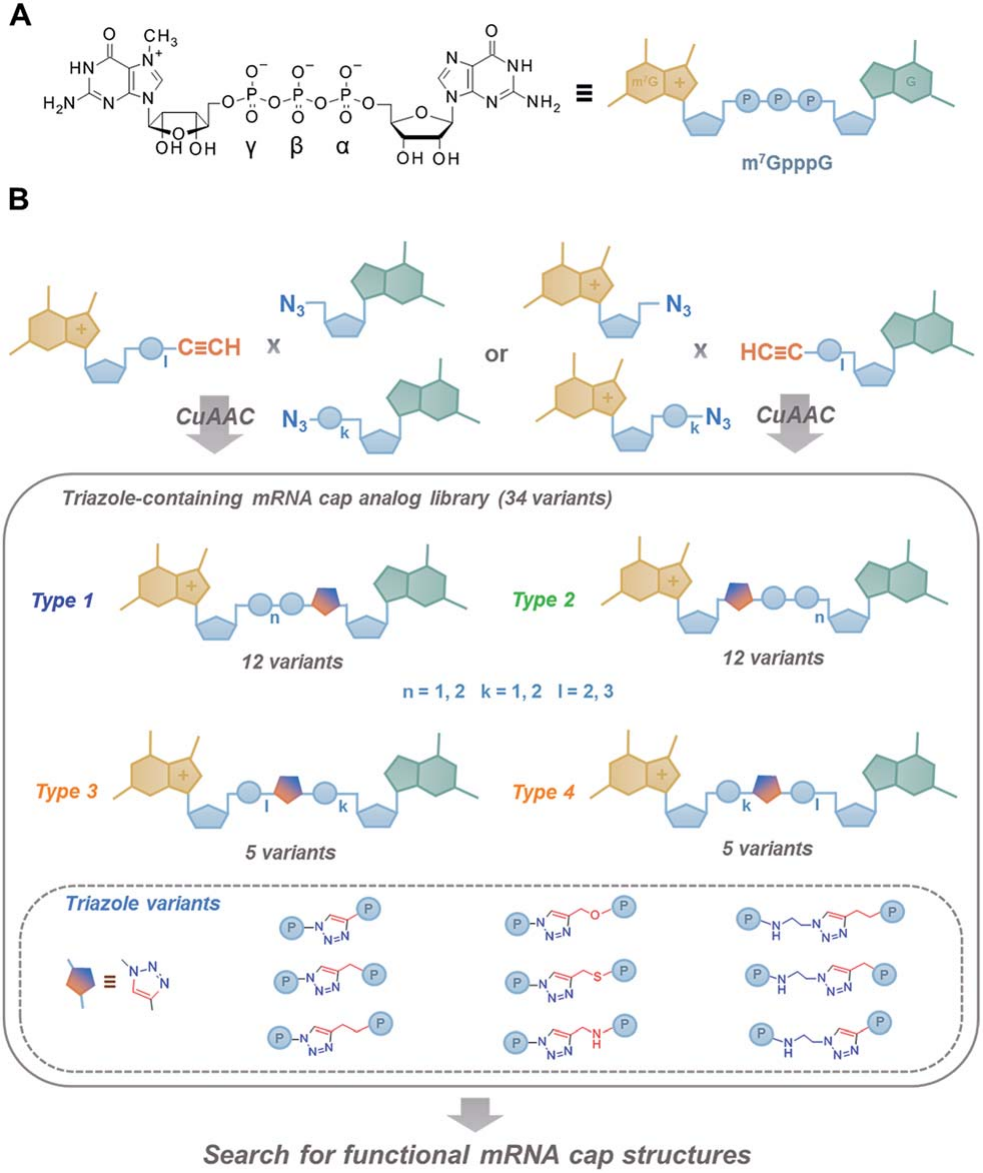
(A) Structure and schematic representation of a standard cap analogue, m^7^GpppG; (B) an overview of the approach towards the development of novel triazole-containing cap analogues by means of CuAAC.

The 7-methylguanosine (m^7^G) cap present at the 5′ end of eukaryotic mRNAs plays a crucial role in numerous fundamental cellular processes, mainly by protecting mRNA from premature cleavage and serving as a molecular platform for proteins acting in mRNA transport and translation.^1^ Thus, the chemical modifications of the 5′ cap can serve as invaluable molecular tools for selective modulation of cap-dependent processes and, consequently, mRNA metabolism.^2^ For example, dinucleotide cap analogues of the m^7^GpppG type (Fig. 1A), especially those modified in the triphosphate bridge, serve as reagents for obtaining stable and efficiently translated mRNAs and have recently gained great attention due to their possible applications in mRNA-based anti-cancer vaccinations and gene-replacement therapies.^3,4^ The potential of cap analogues as small-molecule inhibitors of translation in anti-cancer treatment is also widely recognized.^5^

However, the development of novel cap analogues, either as research tools or compounds with medicinal potential, is limited by difficulties associated with the chemical synthesis of these compounds. The synthesis and purification of mono- and dinucleotide cap analogues are challenging due to the highly ionic nature and chemical lability of 7-methylguanosine.^6,7^ The usual synthetic approach, based on phosphorimidazolide chemistry and divalent-cation-excess-mediated pyrophosphate bond formation in organic solvent,^8–10^ is both time-consuming and labour-intensive, not conducive to upscaling, requires multiple purification steps, and occasionally results in complex mixtures of products and side-products that are difficult to separate.

Therefore, here we aimed at developing a novel class of dinucleotide cap analogues that can be obtained by a simpler and more efficient synthetic approach, based on click chemistry.^11^ We employed a copper(i)-catalysed azide–alkyne cycloaddition (CuAAC) in aqueous medium to combine two 5′-mononucleotide subunits into one 5′,5′-dinucleotide.

CuAAC has been widely applied in the functionalization of RNA and RNA components,^12,13^ including 5′ end labeling,^14–18^ bioconjugation,^19–22^ and chemical ligation.^23^ It has been found that the triazole moiety can replace one or more 5′,3′-phosphodiester bonds in DNA^24,25^ and RNA^26–29^ to generate functional mimics. However, such modification has never been investigated in the context of 5′,5′-oligophosphate moieties. As such, in this work, we aimed to broaden the existing knowledge by exploring how the triazole moiety influences the properties of biologically relevant dinucleoside 5′,5′-oligophosphates, namely mRNA cap analogues.

To that end, we used CuAAC to generate a library of 34 dinucleotide cap analogues containing a triazole moiety within the oligophosphate chain as potential biologically active cap mimics (Fig. 1B). To explore structure–activity relationships, the library was designed to contain cap analogues differing in the length of pyrophosphate chain, the structure of the linker joining the triazole moiety with the phosphate, and the position of the triazole within the oligophosphate bridge.

To assess the influence of the triazole moiety on the interaction with proteins involved in mRNA cap translation and degradation, all analogues were then characterised for their affinity for eukaryotic translation initiation factor 4E (eIF4E) and susceptibility to the decapping scavenger (DcpS) enzyme. We also produced mRNAs terminated with all the triazole-containing cap structures and determined their translational efficiencies in cell lysates. This resulted in the identification of two cap analogues that, despite the presence of triazole modification, had translational properties comparable to RNAs carrying standard 5′ caps, thereby providing proof of concept for the utility of CuAAC in the synthesis of biologically active dinucleoside 5′,5′-oligophosphate analogues.

## Results and discussion

### Synthesis of triazole-modified dinucleotide cap analogues

**Fig. 2:**
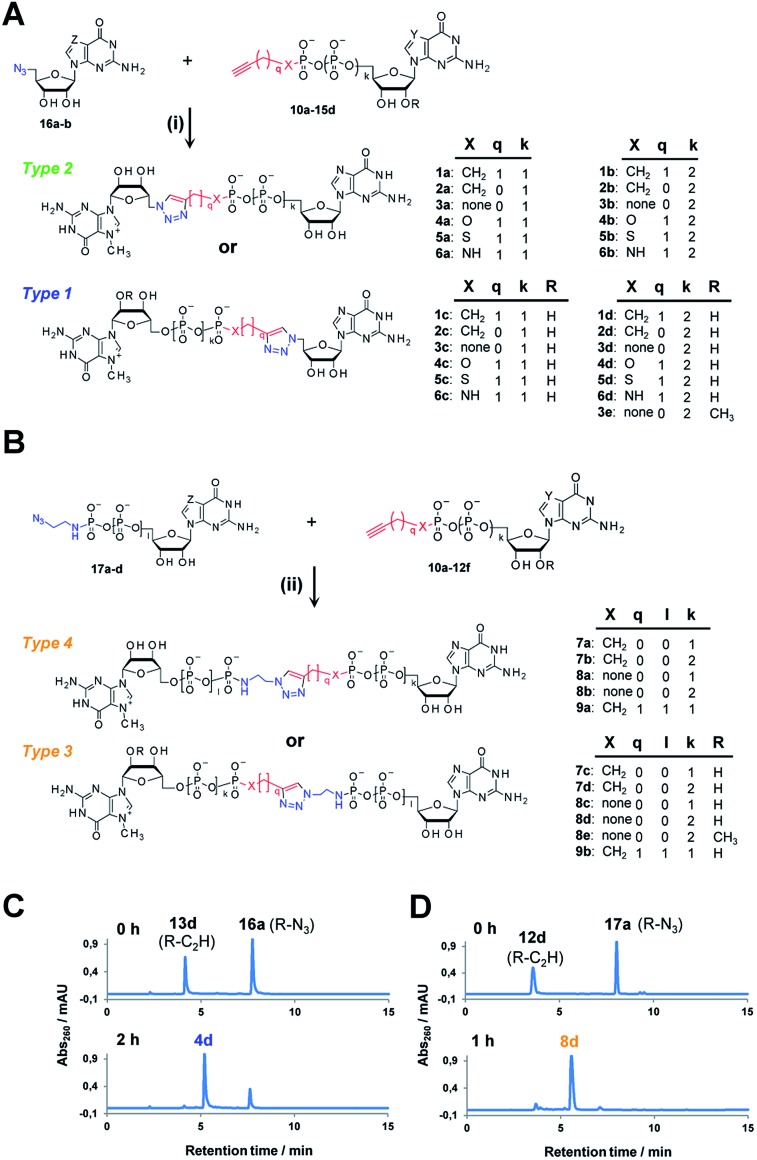
Synthesis of triazole-containing dinucleotide cap analogues by CuAAC: (A) type 1 and 2, (B) type 3 and 4. Conditions: (i) CuSO_4_, sodium ascorbate, H_2_O/DMF, (ii) CuSO_4_, sodium ascorbate, H_2_O (C and D) HPLC profiles of CuAAC reactions leading to analogues **4d** and **8d**.

Triazole-modified cap analogues (**1–9**) were synthesized by CuAAC reactions between two “clickable” mononucleotide analogues, a guanine nucleotide containing a terminal alkyne within the phosphate chain (**10–15**) and an appropriate azido derivative of nucleoside (**16a–b**) or nucleotide (**17a–d**) (Fig. 2). The alkyne- and azide-containing reactants were coupled in 34 different combinations under standard CuAAC conditions (CuSO_4_, sodium ascorbate)^30^ in water or a water/DMF mixture, depending on reactant solubility (Fig. 2 and Table S1†). The reactions were quenched with EDTA to ensure the effective removal of copper ions (Fig. S1†), which could otherwise interfere with subsequent biological experiments, and the products were directly purified by reversed phase high-performance liquid chromatography (RP HPLC). All 34 dinucleotide cap analogues were obtained in good-to-excellent yields (68–100%, usually exceeding 90%; Table S1†) and isolated in high purity.

**Fig. 3:**
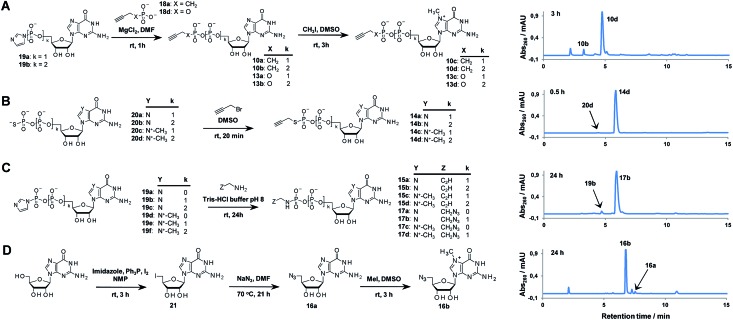
Synthesis of azide- or alkyne-modified building blocks: *C*-phosphonate and phosphoester analogues (A), phosphorothiolate analogues (B), phosphoramidate analogues (C), 5′-azido-nucleosides (D), and HPLC profiles of representative reactions.

A variety of clickable mononucleotides functionalized at the terminal position with azide- or alkyne-containing group was obtained by replacing the terminal phosphate with a phosphoester, phosphorothiolate, phosphoroamidate, or *C*-phosphonate moiety. Different chemical strategies were applied to obtain these compounds, depending on the type of linkage at the terminal phosphate (Fig. 3 and Table S2†). Alkyne-containing *C*-phosphonate analogues (**10a–d**, **11a–d**, **12a–f**) were obtained by MgCl_2_-mediated coupling between triethylammonium salts of *C*-phosphonate subunits (**18a–c**) and guanosine mono- or diphosphate imidazolides (**19a–b**),^31^ followed by *N* ^7^-methylation by methyl iodide (Fig. 3A). The phosphoester analogues (**13a–d**) were obtained under similar conditions by replacing the *C*-phosphonate subunit with propargylphosphate (**18d**).^32^ Compounds containing a phosphorothiolate moiety (**14a–d**) were obtained by selective and efficient *S*-alkylation of nucleotides containing a terminal phosphorothioate moiety (**20a–d**) (Fig. 3B).^33,34^ Finally, phosphoramidate analogues containing either an alkyne or azide moiety (**15a–d**, **17a–d**) were synthesized by reacting propargylamine or 2-azidoethylamine and an appropriate guanine nucleotide imidazolide (**19a–f**) in aqueous buffer (Fig. 3C).^35^ 5′-Azido-5′-deoxyguanosine (**16a**) was synthesized from 5′-iodo-5′-deoxyguanosine^36,37^ (**21**) and *N* ^7^-methylated by methyl iodide in DMF to yield 5′-azido-7-methylguanosine (**16b**) (Fig. 3D). Importantly, all clickable nucleotides were synthesized in high yield and in 2–3 steps from commercially available nucleotides (Table S2†).

The structures of all triazole-modified analogues as well as clickable starting materials were confirmed by HR MS, ^31^P NMR, ^1^H NMR, and two-dimensional ^1^H–^13^C NMR spectroscopy (ESI S2†). The presence and position of the triazole moiety in analogues with triazole directly attached to ribose (type 1 and 2, Fig. 2A) was clearly visible due to a strong deshielding (∼0.5 ppm) effect on the H5′ and H5′′ protons adjacent to the triazole (Fig. S2A†). For analogues containing the triazole between the phosphates, the presence of the modification was confirmed mostly by observing the resulting signal changes in ^31^P NMR spectra (Fig. S2B†).

Before biological evaluation, the chemical stability of representative dinucleotides (**2d**, **3b**, **4d**, **5a**, **6a**, and **9a**) was studied in aqueous buffers with different pH values (3, 5, 7, and 9). All analogues were stable at pH 5 and 7. At pH 9, 7-methylguanosine ring opening was observed, as also observed with unmodified caps.^6^ Only analogues containing the phosphoramidate moiety were unstable at pH 3 due to hydrolytic P–N bond cleavage (Fig. S3†).

### Affinity for eIF4E and susceptibility to hydrolysis by DcpS

**Fig. 4:**
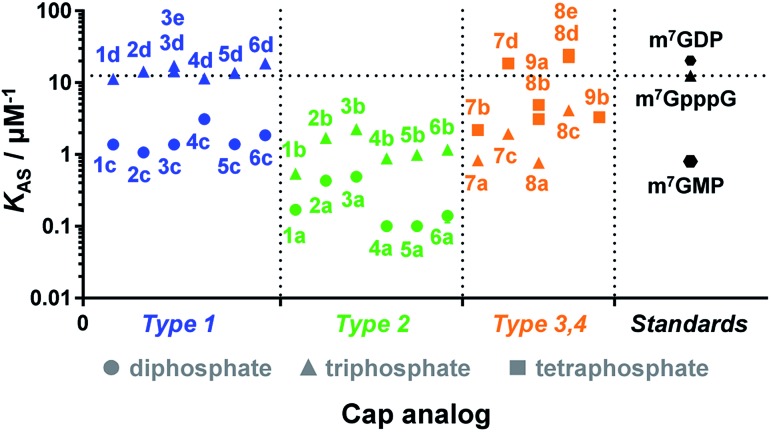
Binding affinities for triazole-containing cap analogues to eIF4E. Cap analogue types 1–4 are defined in Fig. 1 and 2. The *K*_AS_ values are given in Table 1.

Due to the crucial role of eIF4E in translation initiation,^38^ determination of affinity to the protein is usually the first test to verify if a cap analogue may be treated as a good mimic of the mRNA 5′ cap. The affinity of the cap for the eIF4E protein depends mainly upon hydrophobic cation–π interactions with 7-methylguanine (m^7^G) and electrostatic interactions with the negatively charged oligophosphate chain, and it has been previously shown that even minor modifications to the phosphate chain can affect the binding significantly.^39^ The association constants (*K*_AS_) for eIF4E–cap analogue complexes were determined by fluorescence-quenching titration (Fig. S4†).^39^ The affinity for eIF4E varied significantly depending on the analogue structure, including the number of negative charges in the modified oligophosphate chain, the position of the triazole moiety, and the type of linkage involved (Fig. 4 and Table 1). Thus, several conclusions could be drawn from the results. First, the dependence between the number of phosphates and the affinity to eIF4E was similar to that previously observed for unmodified cap analogues, *i.e.* the *K*_AS_ increased approximately 10-fold with an additional phosphate moiety.^40^ For example, analogue **1d** had a *K*_AS_ 8.2-fold higher than analogue **1c**, which was structurally related but contained two phosphate moieties instead of three (*K*_AS_ = 11.25 ± 0.03 μM^–1^ *versus K*_AS_ = 1.38 ± 0.03 μM^–1^ for **1d** and **1c**, respectively).

**Table 1:** Summary of biochemical properties of triazole-containing cap analogs and RNAs capped co-transcriptionally with these compounds^*a*^

Cap analogue	*K*_AS_ cap–eIF4E [μM^–1^]	hDcpS assay^*b*^	Capping efficiency^*c*^	Translation efficiency^*d*^
m^7^GMP	0.81 ± 0.07^39^	n.d.	n.d.	n.d.
m^7^GDP	20.4 ± 1.5^39^	n.d.	n.d.	n.d.
m^7^GpppG	12.5 ± 0.3^41^	0.61 ± 0.09	90%	1.00
GpppG	n.d.	n.d.	92%	0.06 ± 0.01
**1a**	0.17 ± 0.01	1.00	58%	0.07 ± 0.01
**1b**	0.54 ± 0.04	1.00	62%	0.08 ± 0.02
**1c**	1.38 ± 0.03	1.00	68%	0.09 ± 0.01
**1d**	11.25 ± 0.03	0.92	65%	0.14 ± 0.02
**2a**	0.43 ± 0.03	1.00	79%	0.14 ± 0.05
**2b**	1.70 ± 0.04	1.00	65%	0.19 ± 0.02
**2c**	1.07 ± 0.04	1.00	83%	0.09 ± 0.01
**2d**	14.3 ± 0.6	0.88	73%	0.19 ± 0.03
**3a**	0.49 ± 0.03	1.00	77%	0.11 ± 0.01
**3b**	2.24 ± 0.06	1.00	80%	0.19 ± 0.06
**3c**	1.37 ± 0.06	0.99	82%	0.07 ± 0.01
**3d**	17.1 ± 1.1	0.81	79%	0.17 ± 0.02
**4a**	<0.1	1.00	71%	0.06 ± 0.01
**4b**	0.88 ± 0.02	1.00	78%	0.14 ± 0.00
**4c**	3.10 ± 0.13	0.94	86%	0.10 ± 0.01
**4d**	11.5 ± 0.3	0.78	76%	0.18 ± 0.02
**5a**	<0.1	1.00	73%	0.07 ± 0.01
**5b**	0.99 ± 0.04	1.00	77%	0.16 ± 0.04
**5c**	1.39 ± 0.06	0.98	88%	0.14 ± 0.00
**5d**	13.6 ± 1.3	0.73	83%	0.26 ± 0.02
**6a**	0.14 ± 0.03	1.00	58%	0.05 ± 0.00
**6b**	1.16 ± 0.06	1.00	28%	0.09 ± 0.03
**6c**	1.86 ± 0.06	1.00	76%	0.12 ± 0.03
**6d**	18.6 ± 0.7	0.93	62%	0.18 ± 0.01
**7a**	0.83 ± 0.04	1.00	47%	0.08 ± 0.02
**7b**	2.20 ± 0.05	0.99	62%	0.07 ± 0.00
**7c**	1.94 ± 0.07	1.00	54%	0.16 ± 0.02
**7d**	18.6 ± 0.7	0.94	44%	0.94 ± 0.12
**8a**	0.77 ± 0.04	0.97	57%	0.08 ± 0.01
**8b**	3.1 ± 0.2	1.00	57%	0.10 ± 0.03
**8c**	4.13 ± 0.06	0.94	57%	0.50 ± 0.06
**8d**	22.6 ± 0.5	0.86	52%	0.89 ± 0.11
**9a**	4.9 ± 0.1	1.00	30%	0.24 ± 0.03
**9b**	3.3 ± 0.1	1.00	49%	0.15 ± 0.01
m_2_^7,3′-*O*^GpppG	10.2 ± 0.3^41^	n.d.	83%	1.56 ± 0.16
**3e**	14.4 ± 0.4	n.d.	17%	0.55 ± 0.03
**8e**	24.7 ± 0.6	n.d.	41%	1.46 ± 0.18

^*a*^n.d. – not determined.

^*b*^Fraction of cap remaining after 1 h.

^*c*^For 35 nt transcripts synthesized by SP6 RNA Pol.

^*d*^For luciferase-encoding transcripts in rabbit reticulocyte lysates.

Second, the distance between the triazole and the 7-methylguanosine moiety also had a significant influence on the affinity for eIF4E. Type 1 analogues, *i.e.* with the triazole moiety attached to guanosine, containing a triphosphate moiety (**1d–6d**) had affinities similar to or higher than the corresponding unmodified cap structure, m^7^GpppG (*K*_AS_ = 12.5 ± 0.3 μM^–1^).^41^ In contrast, type 2 analogues, *i.e.* with the triazole moiety attached to 7-methylguanosine, containing a triphosphate moiety (**1b–6b**) had affinities lower than m^7^GpppG (*e.g. K*_AS_ = 2.24 ± 0.06 μM^–1^ and 0.54 ± 0.04 μM^–1^ for **3b** and **1b**, respectively). Comparisons within type 3 and 4 analogues also indicated that increasing the distance of the triazole moiety from the m^7^G moiety results in complex stabilization. Finally, the *K*_AS_ values of the structurally related cap analogues of type 1 and 2 differing only in the linkage type (series **1a–6a**, **1b–6b**, *etc.*), revealed that the shortest possible linkage (direct triazole attachment, as in compounds **3a**, **3b** and **3d**) usually maximizes the affinity for cap–eIF4E complexes. These data indicated that properly designed triazole-containing analogues can be specifically recognized by eIF4E.

**Fig. 5:**
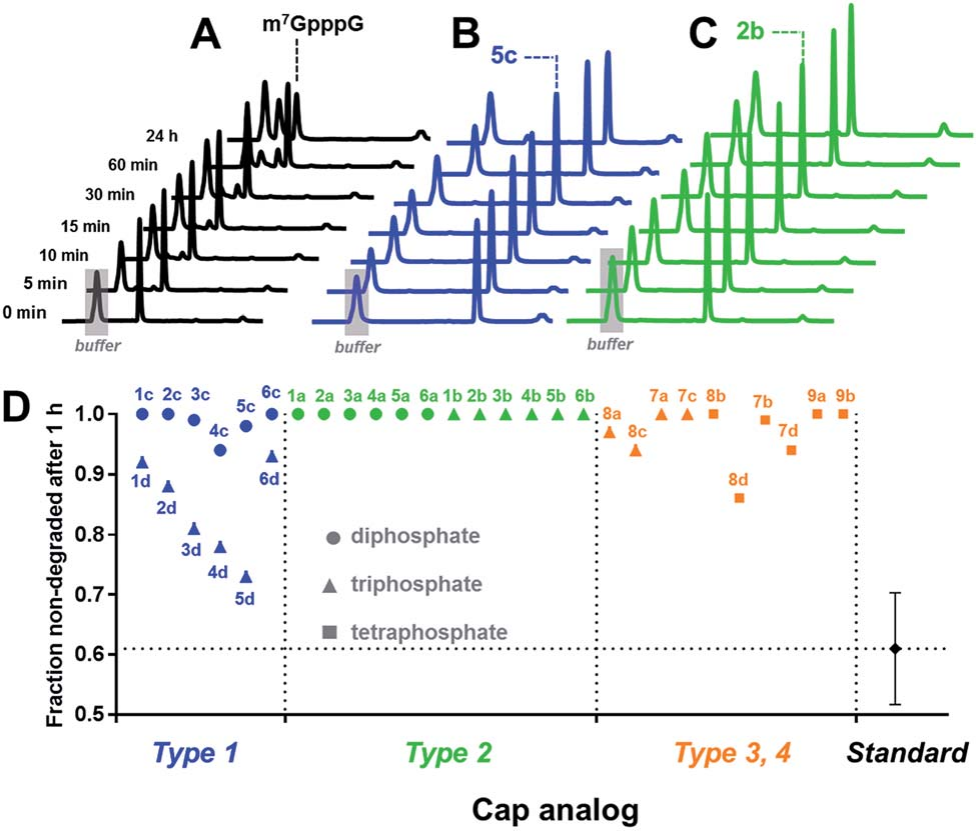
Determination of the susceptibility of analogues to degradation by hDcpS enzyme using an HPLC-based assay. HPLC profiles of fractions from reaction mixtures containing m^7^GpppG (A), **5c** (B), or **2b** (C). (D) Non-degraded fractions after 1 h, for each analogue. The values are also summarized in Table 1.

Cap cleavage is a crucial step in mRNA turnover, involving both 5′ → 3′ and 3′ → 5′ degradation pathways. Therefore, we also tested the influence of the triazole moiety on cap susceptibility to enzymatic hydrolysis. As a model enzyme, we used DcpS, a decapping pyrophosphatase responsible for removing cap structures released from mRNA upon 3′ → 5′ degradation by cleaving them at the β–γ position of triphosphate chain to release m^7^GMP.^42^ Each triazole-containing cap analogue (20 μM concentration) was incubated with 5 nM hDcpS, and the hydrolysis levels at different time points were analysed by RP HPLC and compared to m^7^GpppG (Fig. 5 and Table S3†). Under these conditions, ∼40% cleavage of m^7^GpppG was achieved within 1 h. The susceptibility of the tested cap analogues to DcpS correlated strongly with the position of the triazole ring. All type 2 analogues were completely resistant to hDcpS. Type 1 analogues carrying a diphosphate moiety were also resistant or very slowly hydrolysed. In contrast, type 1 analogues carrying a triphosphate moiety were all DcpS substrates, with the highest susceptibility found for phosphoester and phosphothioester analogues (**4d** and **5d**). Type 3 and 4 analogues were substrates for DcpS only if the triazole moiety was separated from m^7^G by no less than three phosphate moieties. Complete resistance of type 2 analogues was observed even under much higher enzyme concentrations (Table S4†). To gain insight into how the presence of the triazole moiety influences the cap–DcpS interaction, the analogues were also evaluated as hDcpS inhibitors using a recently developed fluorescent HTS assay.^43^ The relatively low %_inhibition_ parameters (Fig. S5†) and high IC_50_ values (Fig. S6†) of the cap analogues compared to the reference inhibitors (m^7^GDP and m^7^GpCH_2_ppG) indicated that the low susceptibility of cap analogues to hDcpS was due to rather poor recognition of the triazole-containing analogues by the enzyme.

Overall, the results indicated that the general requirements for cap-recognition were similar for both the eIF4E and DcpS proteins. Most of the tested analogues were either well-recognized by both proteins (*e.g.* **3d**, **5d**, **8d**) or poorly recognized (*e.g.* **1a**, **6a**). Interestingly, however, some analogues were poor DcpS substrates, but had high affinity for eIF4E (*e.g.* **1d**, **6d**). Such compounds are potentially useful as selective and enzymatically resistant, small-molecule inhibitors of translation in cancer cells.

Most importantly, the high affinity of several triazole-containing analogues for eIF4E suggested that the compounds may be used as functional cap mimics. This finding prompted us to explore the utility of the new analogues as reagents for the synthesis of 5′-capped mRNAs *in vitro* and subject such RNAs to biochemical evaluation.

### Incorporation into RNA and translational efficiency

*In vitro* transcribed (IVT) 5′-capped mRNAs are invaluable tools for studying mRNA translation, transport, and turnover, and are an emerging class of highly promising therapeutic molecules.^4^ We envisaged that the triazole-containing cap analogues could be useful for producing translationally active and enzymatically resistant capped mRNAs, provided that they can be effectively incorporated at the mRNA 5′ end.

**Fig. 6:**
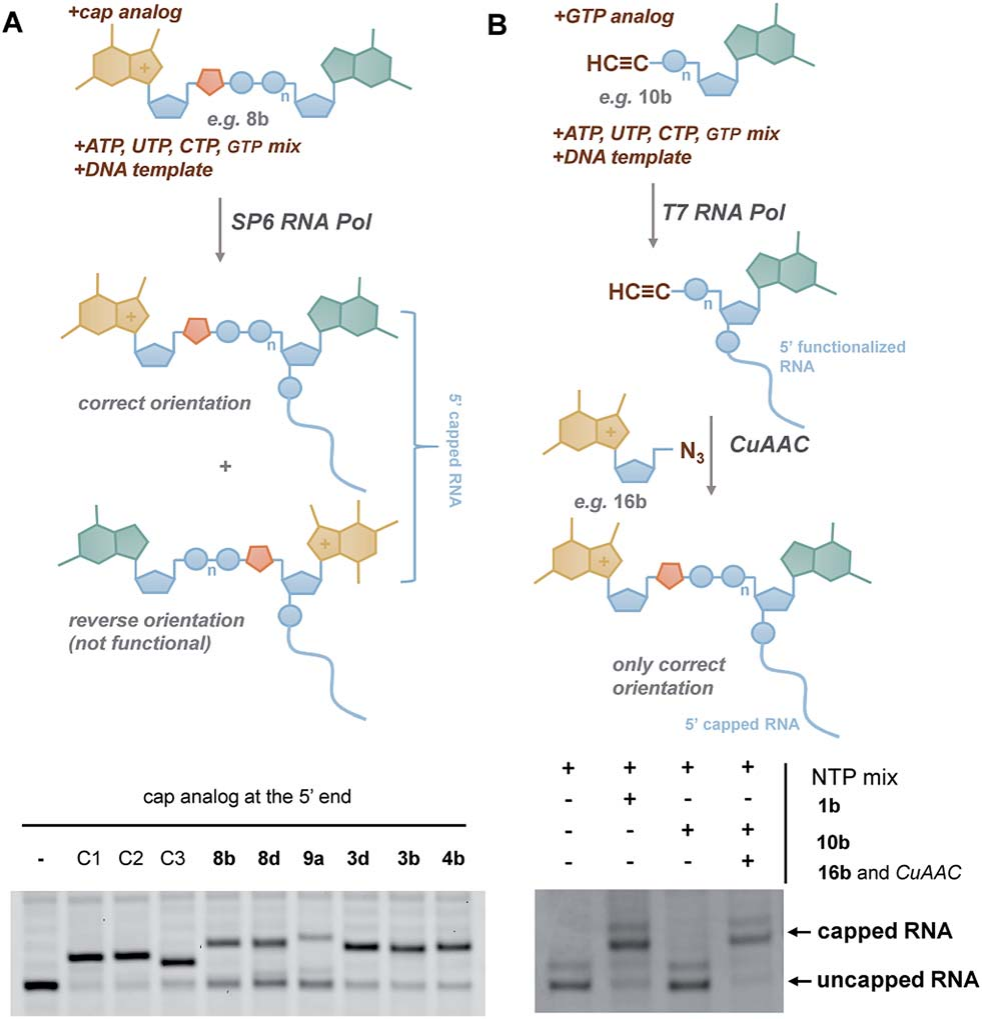
Introduction of selected triazole-containing cap analogues at the RNA 5′ end by co-transcriptional (A) and post-transcriptional (B) capping. The sequences of the transcripts are shown in Table S5,† and the results of co-transcriptional capping for all compounds are shown in Fig. S9.†

In general, the synthesis of 5′-capped mRNAs can be achieved by 2 approaches, both based on *in vitro* transcription. In the co-transcriptional approach (Fig. 6A), RNA synthesis is performed in the presence of all 4 NTPs and a cap dinucleotide, such as m^7^GpppG. The DNA template is designed to incorporate G as the first transcribed nucleotide. Under such conditions, RNA polymerase can start the transcription taking GTP or m^7^GpppG as the initiating nucleotide, thereby incorporating one of these at the 5′ end of the nascent RNA. To increase the percentage of cap analogue incorporation (capping efficiency), the GTP concentration is decreased relative to the other NTPs, and the concentration of the cap dinucleotide is elevated (from 4- to 10-fold excess relative to GTP).^44^ This method is widely employed because of its simplicity and capacity to introduce various chemically modified cap structures. However, the capping efficiencies achieved by this method can vary, and optimization is often required to obtain high-quality mRNAs. In addition, reverse incorporation of cap dinucleotides can potentially occur, resulting in a fraction of ‘Gpppm^7^G-capped’ RNAs, which are translationally inactive. Fortunately, this problem can be overcome by the introduction of m^7^G ribose modifications to produce so called ‘anti-reverse cap analogues’ (ARCAs).^41,45^

In the second approach, *i.e.* post-transcriptional capping, the cap is added enzymatically to IVT 5′-triphosphate RNA using the capping enzyme from a vaccinia virus, which uses GTP and *S*-adenosylmethionine as co-substrates. This method is advantageous because it typically results in high-efficiency capping, although the repertoire of cap modifications is restricted by the capping enzyme's specificity for GTP analogues.^46^

To verify the utility of triazole-containing cap analogues for RNA 5′-capping, we first tested their incorporation at the RNA 5′ end by two alternative methods. One method was the typical co-transcriptional capping approach, and the other was similar to post-transcriptional enzymatic capping, but relied on the chemical assembly of the cap structure under CuAAC conditions. To test the co-transcriptional capping approach, ∼35-nt transcripts were synthesized by RNA Pol SP6 in the presence of a double-stranded DNA template, NTP mix (0.5 mM, except for GTP [0.125 mM]), and a given cap analogue (1.25 mM). The transcripts were trimmed to 25 nt at the 3′ end using DNAzyme 10–23 to reduce heterogeneity,^47^ followed by resolution by polyacrylamide gel electrophoresis (Fig. 6A). As capped RNAs migrated slower than uncapped (GTP-initiated) RNAs, the capping efficiency could be calculated based on the intensity of corresponding bands (Table 1). We found that all analogues were incorporated at the RNA 5′ end under standard conditions, with an efficiency comparable to or lower than that of standard cap structures (Table 1), but in all cases were sufficient for further biological evaluation. However, it should be noted here that the method of analysis could not distinguish between analogues capped in correct and reverse orientations.

The potential problem of reverse incorporation of the cap into RNA does not occur in the post-transcriptional capping strategy (Fig. 6B). To test this approach, an alkyne-containing guanine mononucleotide (**10b**) was incorporated at the 5′ end of 35-nt RNA by *in vitro* transcription, followed by chemical capping of the resulting 5′-functionalized RNA with 5′-azido-5′-deoxy-7-methylguanosine (**16b**). The CuAAC reactions were performed with 10.5 pmol RNA at relatively low concentrations (∼3 μM). Different reaction conditions were tested (Table S6†) to ensure the highest capping efficiency and to minimize RNA degradation triggered by the presence of Cu(i) ions, which is a known problem for CuAAC with RNA, especially at low concentrations.^23^ Under optimized conditions (2.9 μM RNA, 0.14 mM **16b**, 0.29 mM CuSO_4_, 0.58 mM THPTA, and 0.58 mM sodium ascorbate), we obtained 88% capping efficiency with a moderate degree of RNA degradation (Fig. 6B), demonstrating for the first time a chemical, in-solution m^7^G-capping reaction with RNA. Because it has been previously shown that many different guanosine derivatives can be introduced at the RNA 5′ ends,^48–50^ we envisaged that the method could be further extended to structurally divergent cap structures, which we are currently investigating. We also anticipate that the capping efficiencies could be notably improved for larger scale capping reactions, in which higher RNA concentrations could be used.

To assess the functionality of the triazole-containing cap structures in translation, we introduced all analogues into full-length mRNAs and determined their translation efficiencies (Fig. S8†). Luciferase-encoding RNAs capped with various cap analogues (**1–9**) or control analogues (m^7^GpppG [standard cap analogue] and GpppG [non-functional cap]) were synthesized under standard *in vitro* transcription conditions. The mRNAs were translated in rabbit reticulocyte lysate, and luciferase activities were determined after 60 min by luminometry. The luciferase activities were depicted as a function of RNA concentration (Fig. S7†) and the translation efficiencies, defined as the maximum slope, were calculated and normalized to m^7^GpppG–RNA (for which the translation efficiency was set to 1; Table 1). The translation efficiency of mRNA capped with the non-functional cap analogue (GpppG–RNA) occurred at a minimal level (tr. eff. = 0.06 ± 0.01), indicating that the system was well-optimized to exclusively measure cap-dependent translation. The translation efficiencies of mRNAs carrying triazole-containing cap analogues varied between 0.06 and 0.94 under these conditions. The highest translation efficiencies were observed for RNAs capped with compounds **7d** and **8d**, identifying these analogues as fully functional triazole-containing cap mimics. All other cap analogues had notably lower translation efficiencies, varying between 0.06 and 0.50. However, the underlying reasons for these observations with particular analogues are likely to be different. To produce mRNA with a high translational efficiency, the cap analogue must not only favourably interact with eIF4E, but also be incorporated into transcripts by RNA polymerase efficiently and in the forward orientation.^45^ Only analogues **7d** and **8d** fulfilled both these conditions and provided efficiently translated mRNAs. Some RNAs with low translation efficiencies (*e.g.* those capped with compounds **3b**, **4b**, **8b**, and **9a**) were probably poorly recognized by translational machinery due to a relatively low affinity for eIF4E (Fig. 4 and Table 1). In contrast, low translation efficiencies for RNAs capped with type 1 analogues (*e.g.* **3d**, **4d** and **6d**) which had affinity for eIF4E that was comparable or even higher than m^7^GpppG, cannot be explained in the same manner. Instead, we hypothesize that because of the presence of triazole-modified guanosine moiety these analogues are incorporated into RNA solely in the reverse orientation, producing reverse-capped RNAs (similar to GpppG–RNA) as the major product. Therefore, those analogues could still be useful as RNA capping reagents, if conditions could be established that ensure proper (forward) incorporation into RNA.

Notably, even for analogues **7d** and **8d**, the capping efficiencies were lower than for the standard analogue (44% and 52%, respectively, *versus* 92% for m^7^GpppG), and the problem of reverse-incorporation was not eliminated. Therefore, the translation efficiencies of mRNAs capped with **7d**, **8d**, and some of the type 1 analogues could potentially be improved by fine-tuning their structures. To demonstrate this, we synthesized ARCA^41^ versions of **3d** and **8d**, namely m_2_^7,2′-*O*^Gppp-triazole-G (**3e**) and m_2_^7,2′-*O*^Gppp-triazole-C_2_H_4_NHpG (**8e**). These analogues were, again, used as substrates for the transcription of short RNAs to determine the capping efficiencies. The capping efficiencies were lower in comparison to the corresponding non-ARCAs, especially in the case of compound **3e** (Table 1 and Fig. S9†), which confirmed that nucleotides with triazole directly attached to guanosine were not preferred substrates for SP6 RNA polymerase. Nonetheless, both analogues were incorporated into mRNA and translation efficiencies were determined. Compound **8e** gave similar results to the reference compound m_2_^7,3′-*O*^GpppG, which is a commercially available ARCA (tr. eff. 1.46 ± 0.18 and 1.56 ± 0.16, respectively).

This result was observed despite lower capping efficiency for **8e**, suggesting that the compound could perform even better if the capping procedure was optimized. Surprisingly, RNAs capped with **3e** were also translated at a notable level (tr. eff. 0.55 ± 0.03) despite very low efficiency of capping, suggesting that, once incorporated into RNA, this cap analogue is also fully functional. Therefore, as for **3d**, **3e**, and other type 1 analogues, we are currently investigating different approaches for improving the capping RNA efficiencies, *e.g.* through further development of the concept of post-transcriptional capping. The results of these studies will be published elsewhere together with other biological properties of RNAs capped with these analogues.

Finally, we tested if RNAs capped with **3e** and **8e** are susceptible to cleavage by hDcp2, which is an RNA-dependent decapping enzyme acting in the 5′ → 3′ pathway.^51^ We found both triazole-containing caps were susceptible to hDcp2 cleavage similarly as the reference ARCA (Fig. S10†). This finding reveals that modulating susceptibility to hDcp2 cleavage is another strategy to improve the properties of triazole-containing cap analogues in future.

## Conclusions

In conclusion, we developed a novel class of dinucleotides containing a triazole moiety within the oligophosphate bridge, which are efficiently synthesized by CuAAC. The analogues were first characterized as cap mimics in interactions with two key cap-binding proteins, translation factor eIF4E and the decapping enzyme DcpS. Several cap analogues were identified as high-affinity binders of eIF4E with low susceptibility to hydrolysis by hDcpS. The compounds did not inhibit DcpS efficiently, which suggests they are bioorthogonal towards DcpS-related processes. We then incorporated the analogues at 5′ end of short and long RNAs using a co-transcriptional capping approach. Additionally, we demonstrated the possibility to incorporate modified cap structures into RNA by chemical post-transcriptional capping based on CuAAC. Using co-transcriptionally capped full-length luciferase transcripts, we tested the influence of triazole-containing analogues on mRNA translation. As a result, we identified two cap analogues, m^7^Gppp-triazole-C_2_H_4_NHpG (**8d**) and m^7^GpppCH_2_-triazole-C_2_H_4_NHpG (**7d**), which conferred translational properties to mRNA similar to those of mRNAs capped with the unmodified analogue, m^7^GpppG. Further tuning the structure of **8d** by introducing an anti-reverse modification (2′-*O*-methyl group) increased the translation efficiency of mRNA by an additional 50% and afforded a fully functional cap mimic.

Thus, the results of our study feature the successful implementation of CuAAC-based click chemistry for the synthesis of biologically active dinucleoside oligophosphates carrying a 1,2,3-triazole ring within the oligophosphate moiety. We believe that the presented findings open a new field for the development of chemically modified RNA cap analogues conferring increased stability and high translational efficiencies of mRNAs. However, improvements in RNA-capping strategies and further biological studies are required to fully explore the potential of the novel cap structures. This is currently under investigation by our group, along with testing the activities of triazole-containing cap analogues in other cap-dependent processes.
