# Supplementary material for: A novel route for preparing 5′ cap mimics and capped RNAs: phosphate-modified cap analogues obtained via click chemistry
Source: Chem Sci. 2016 Aug 16;8(1):260–7. doi: 10.1039/c6sc02437h (PMC5355871; doi:10.1039/c6sc02437h)
Supplement: Supplementary file 1 [file SC-008-C6SC02437H-s001.pdf]

Electronic Supplementary Material (ESI) for Chemical Science.
This journal is © The Royal Society of Chemistry 2016

A novel route for preparing 5′cap mimics and capped RNAs: phosphate-modified cap analogues obtained *via* click chemistry

S. Walczak,[ab] A. Nowicka,[ac] D. Kubacka,[c] K. Fac,[ab] P. Wanat,[c] S. Mroczek,[de] J. Kowalska[c] and J. Jemielity[a]

[a]*Centre of New Technologies, University of Warsaw, Banacha 2c, 02-097, Warsaw, Poland*
[b]*College of Inter-Faculty Individual Studies in Mathematics and Natural Sciences, University of Warsaw, Banacha 2c, 02-097, Warsaw, Poland*
[c]*Division of Biophysics, Institute of Experimental Physics, Faculty of Physics, University of Warsaw, Zwirki i Wigury 93, 02-089, Warsaw, Poland*
[d]*Department of Genetics and Biotechnology, Faculty of Biology, University of Warsaw, 02-106 Warsaw, Poland*
[e]*Institute of Biochemistry and Biophysics, Polish Academy of Sciences, 02-106 Warsaw, Poland*

Supplementary Information

Content

Tables: 4

Table S1. Yields of syntheses of triazole-modified dinucleotide cap analogues. 4

Table S2. Yields of the syntheses of azide- and alkyne-modified nucleotide analogues. 7

Table S3. The susceptibility of triazole-containing cap analogues to degradation by hDcpS determined by an HPLC assay. 9

Table S4. The susceptibility of triazole-containing cap analogues to degradation by hDcpS under high enzyme concentration determined by an HPLC assay. 10

Table S5. Sequences of DNA and RNA oligonucleotides used in this work. 10

Table S6. Optimization of post-transcriptional RNA capping by CuAAC 11

Figures: 12

Figure S1. ^{1}H NMR (400 MHz, D_2O, 25 °C) spectra of compound 1a purified by semi-preparative HPLC without Na_2EDTA (A) and in the presence of Na_2EDTA (B). 12

Figure S2. Spectroscopic characterization of triazole-containing cap analogues. 12

Figure S3. Chemical stability of representative triazole-containing dinucleotide cap analogues after 24h incubation at different pH. 13

Figure S4. eIF4E fluorescence quenching titration curves for selected cap analogues (**1b**, **1c**, **1d**). 13

Figure S5. The results of hDcpS screening assay. 14

Figure S6. IC_{50} values for inhibition of hDcpS by analogues **5d** and **8d** under the conditions of screening assay. 14

Figure S7. Luciferase activity produced by translation of mRNAs capped with various cap analogues in rabbit reticulocyte lysate. 15

Figure S8. Translation efficiency of luciferase mRNA capped with novel triazole-modified cap analogs. 16

Figure S9. Co-transcriptional capping efficiency of triazole-modified cap analogues. 17

Figure S10. Susceptibility of short transcripts capped with **3e** ($m_2^{7,3'-O}$Gppp-triazole-G) and **8e** ($m_2^{7,3'-O}$Gppp-triazole-C_2H_4NHpG) to hDcp2-catalyzed decapping. 17

Experimental procedures: 18

1. General information 18

1.1 Starting materials and chemical reagents 18

1.2 Chromatography 18

1.2.1 Ion-exchange chromatography 18

1.2.2 Analytical and preparative reverse-phase (RP) HPLC 18

1.3 Yields and concentrations determination 18

1.4 NMR spectroscopy and mass spectrometry 18

2. Chemical synthesis 19

2.1 Synthesis of nucleotide imidazolide derivatives (19a-h): 19

2.1.1 Preparation of compounds for imidazole-activation 19

Preparation of triethylammonium (TEA) salts 19

Synthesis of guanosine 5'-diphosphate (GDP), guanosine 5'-triphosphate (GTP) and 2'-O-methylguanosine 5'-diphosphate ($m^{2'-O}$GDP) 19

N7-methylation of guanine nucleotides (GMP, GDP and GTP) 19

2.1.2 Imidazole-activation 19

Synthesis of nucleotide imidazolides 19

2.2 Synthesis of phosphoester and C-phosphonate analogues 19

2.2.1 Synthesis of O-(2-propynyl) phosphate ester triethylammonium salt (18d) 19

2.3.2. Synthesis of phosphoester nucleotide analogues 20

General procedure A (GP A): Coupling of nucleotide imidazolides with phosphoester subunit (18d) 20

General procedure B (GP B): N^7-methylation of guanine nucleotides using CH_3I 20

General procedure C (GP C): N^7-methylation of guanine nucleotides using $(CH_3)_2SO_4$ 20

(10a-b, 11a-b, 12a-b) β-C-(2-ethynyl), β-C-(2-propargyl) and β-C-(3-butynyl) guanosine diphosphate and γ-C-(2-ethynyl), γ-C-(2-propargyl) and γ-C-(3-butynyl) guanosine triphosphate triethylammonium salts 20

(13a) β-O-(2-propargyl) guanosine diphosphate ammonium salt 20

(13b) γ-O-(2-propargyl) guanosine triphosphate ammonium salt 20

(13c) β-O-(2-propargyl) 7-methylguanosine diphosphate ammonium salt 21

(13d) γ-O-(2-propargyl) 7-methylguanosine triphosphate ammonium salt 21

(10c) β-C-(3-butynyl) 7-methylguanosine diphosphate ammonium salt 21

(10d) γ-C-(3-butynyl) 7-methylguanosine triphosphate ammonium salt 21

(11c) β-C-(2-propargyl) 7-methylguanosine diphosphate ammonium salt 22

(11d) γ-C-(2-propargyl) 7-methylguanosine triphosphate ammonium salt 22

(12c) β-C-(2-ethynyl) 7-methylguanosine diphosphate ammonium salt 22

(12d) γ-C-(2-ethynyl) 7-methylguanosine triphosphate ammonium salt 22

(12e) γ-C-(2-ethynyl) 2'-O-methylguanosine triphosphate triethylammonium salt 22

(12f) γ-C-(2-ethynyl) 2'-O-N7-dimethylguanosine triphosphate ammonium salt 23

1.1 Synthesis of phosphoramidate nucleotide analogues 23

General procedure D (GP D): Coupling of nucleotide imidazolides with amine linker 23

1.1.1 Synthesis of alkyne-modified phosphoramidate nucleotide analogues 23

(15a) N-(2-propargyl) β-phosphoramidate guanosine diphosphate ammonium salt 23

(15b) N-(2-propargyl) γ-phosphoramidate guanosine triphosphate ammonium salt 23

(15c) N-(2-propargyl) β-phosphoramidate 7-methylguanosine diphosphate ammonium salt 24

(15d) N-(2-propargyl) γ-phosphoramidate 7-methylguanosine triphosphate ammonium salt 24

1.1.2 Synthesis of azide-modified phosphoramidate nucleotide analogues 24

(17a) N-(2-azidoethyl) phosphoramidate guanosine monophosphate ammonium salt 24

(17b) N-(2-azidoethyl) β-phosphoramidate guanosine diphosphate ammonium salt 24

(17c) N-(2-azidoethyl) phosphoramidate 7-methylguanosine monophosphate ammonium salt 24

(17d) N-(2-azidoethyl) β-phosphoramidate 7-methylguanosine diphosphate ammonium salt 25

1.2 Synthesis of phosphothioester nucleotide analogues ... 25

Synthesis of thiophosphate nucleotide analogues (20a-d) ... 25

General procedure E (GP E): S-alkylation of thiophosphate nucleotide analogues ... 25

(14a) S-(2-propargyl) β-phosphothioester guanosine diphosphate diammonium salt ... 25

(14b) S-(2-propargyl) γ-phosphothioester guanosine triphosphate ammonium salt ... 25

(14c) S-(2-propargyl) β-phosphothioester 7-methylguanosine diphosphate ammonium salt ... 25

(14d) S-(2-propargyl) γ-phosphothioester 7-methylguanosine triphosphate ammonium salt ... 26

1.3 Synthesis of azide-modified guanosine analogues ... 26

(16a) 5'-azido-5'deoxyguanosine ... 26

(16b) 5'-azido-5'deoxy-7-methylguanosine ... 26

1.4 Synthesis of dinucleotide cap analogues ... 26

General procedure F (GP F): Synthesis of dinucleotide cap analogues containing triazole directly attached to ribose ... 26

General procedure G (GP G): Synthesis of dinucleotide cap analogues containing triazole located between P-subunits ... 27

(1a) m^7G-triazole-C_2H_4ppG ... 27

(1b) m^7G-triazole-C_2H_4pppG(1b) m^7G-triazole-C_2H_4pppG ... 27

(1c) m^7GppC_2H_4-triazole-G ... 27

(1d) m^7GpppC_2H_4-triazole-G ... 27

(2a) m^7G-triazole-CH_2ppG ... 28

(2b) m^7G-triazole-CH_2pppG ... 28

(2c) m^7GppCH_2-triazole-G ... 28

(2d) m^7GpppCH_2-triazole-G ... 28

(3a) m^7G-triazole-ppG ... 29

(3b) m^7G-triazole-pppG ... 29

(3c) m^7Gpp-triazole-G ... 29

(3d) m^7Gppp-triazole-G ... 29

(3e) $m_2^{7,2'-O}$Gppp-triazole-G ... 30

(4a) m^7G-triazole-OCH_2ppG ... 30

(4b) m^7G-triazole-OCH_2pppG ... 30

(4c) m^7GppOCH_2-triazole-G ... 30

(4d) m^7GpppOCH_2-triazole-G ... 31

(5a) m^7G-triazole-SCH_2ppG ... 31

(5b) m^7G-triazole-SCH_2pppG ... 31

(5c) m^7GppSCH_2-triazole-G ... 31

(5d) m^7GpppSCH_2-triazole-G ... 32

(6a) m^7G-triazole-NHCH_2ppG ... 32

(6b) m^7G-triazole-NHCH_2pppG ... 32

(6c) m^7GppNHCH_2-triazole-G ... 32

(6d) m^7GpppNHCH_2-triazole-G ... 32

(7a) m^7GpNHC_2H_4-triazole-CH_2ppG ... 33

(7b) m^7GpNHC_2H_4-triazole-CH_2pppG ... 33

(7c) m^7GppCH_2-triazole-C_2H_4NHpG ... 33

(7d) m^7GpppCH_2-triazole-C_2H_4NHpG ... 34

(8a) m^{7}GpNHC$_2$H$_4$-triazole-ppG 34
(8b) m^{7}GpNHC$_2$H$_4$-triazole-pppG 34
(8c) m^{7}Gpp-triazole-C$_2$H$_4$NHpG 34
(8d) m^{7}Gppp-triazole-C$_2$H$_4$NHpG 35
(8e) m$_2^{7,2'-O}$Gppp-triazole-C$_2$H$_4$NHpG 35
(9a) m^{7}GppNHC$_2$H$_4$-triazole-C$_2$H$_4$ppG 35
(9b) m^{7}GppC$_2$H$_4$-triazole-C$_2$H$_4$NHppG 35
2. Stability studies 36
3. Biological studies 36
3.1 Determination of K_{AS} cap analogue-eIF4E complexes 36
3.2 Incorporation of dinucleotide cap analogues at 5' end of transcripts 36
3.2.1 Post-transcriptional capping 36
3.2.2 Synthesis of transcripts for capping efficiency determination 36
3.3 Translation studies 37
3.3.1 Synthesis of transcripts 37
3.3.2 Translation 37
3.4 Studies of susceptibility to degradation by hDcpS 37
3.5 Screening against hDcpS 37
3.6 hDcp2-catalyzed decapping 38
References: 38

Tables:

Table S1. Yields of syntheses of triazole-modified dinucleotide cap analogues.

Azide-modified compound	Alkyne-modified compound	Product	HPLC yield
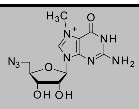 **16b**	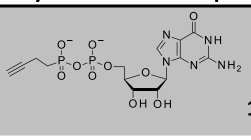 **10a**	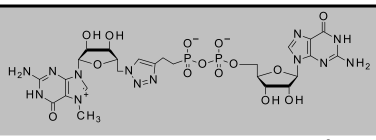 **1a**	>99%
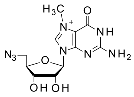 **16b**	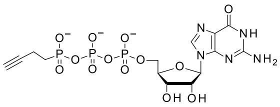 **10b**	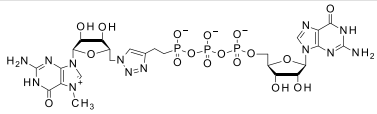 **1b**	76%
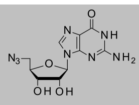 **16a**	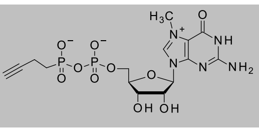 **10c**	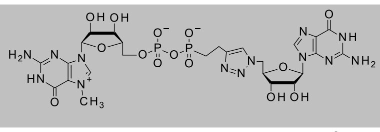 **1c**	81%
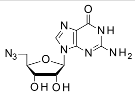 **16a**	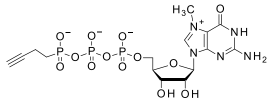 **10d**	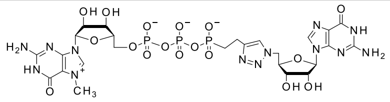 **1d**	77%
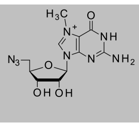 **16b**	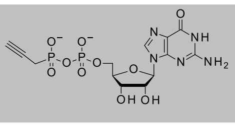 **11a**	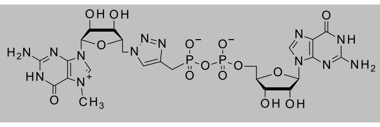 **2a**	74%
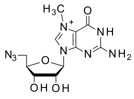 **16b**	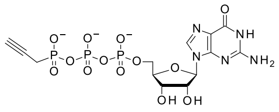 **11b**	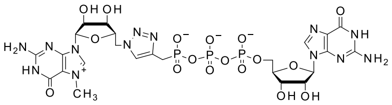 **2b**	84%

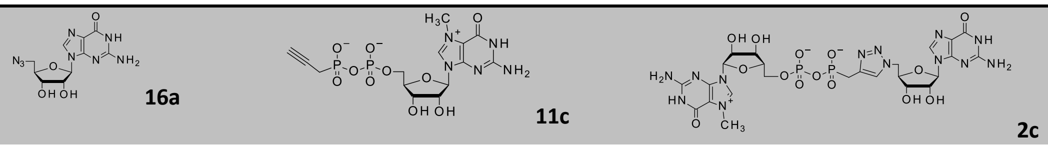 16a	11c	2c	77%
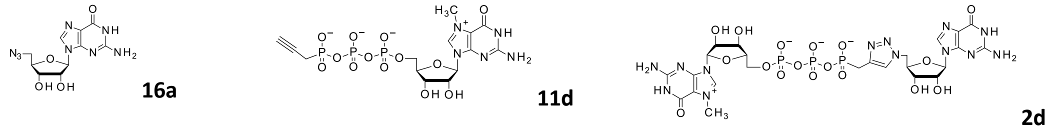 16a	11d	2d	91%
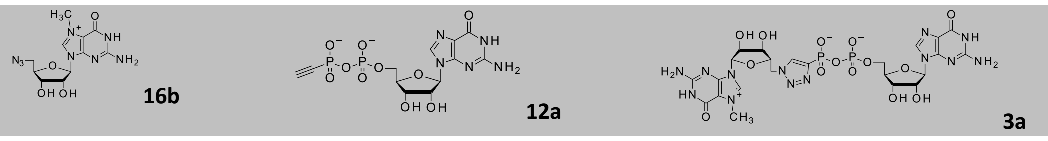 16b	12a	3a	>99%
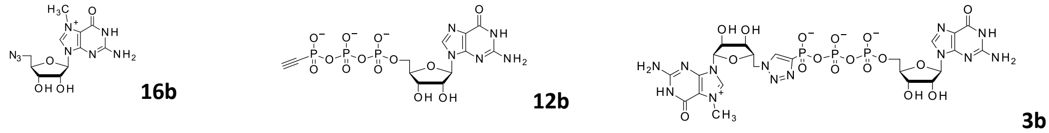 16b	12b	3b	>99%
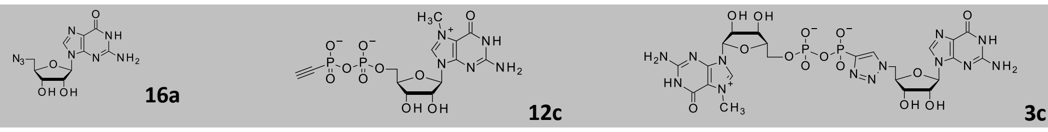 16a	12c	3c	>99%
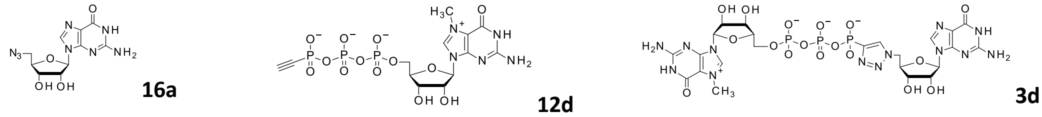 16a	12d	3d	68%
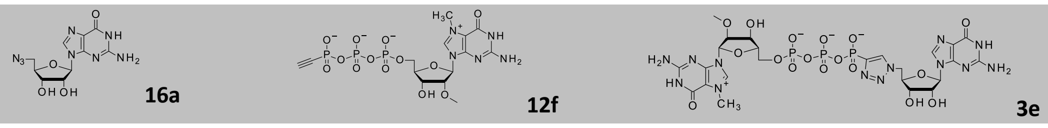 16a	12f	3e	>99%
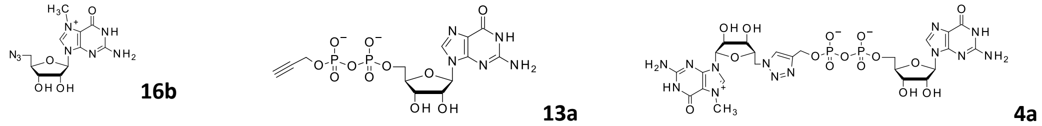 16b	13a	4a	>99%
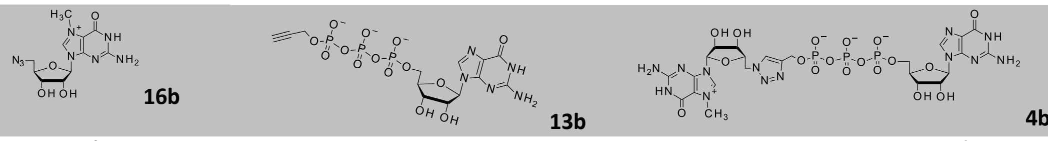 16b	13b	4b	>99%
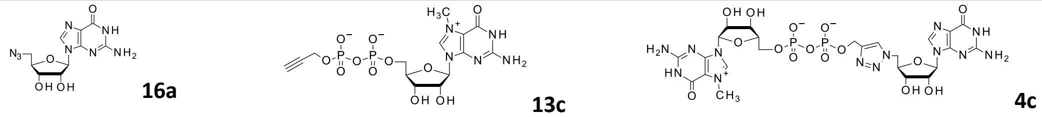 16a	13c	4c	78%
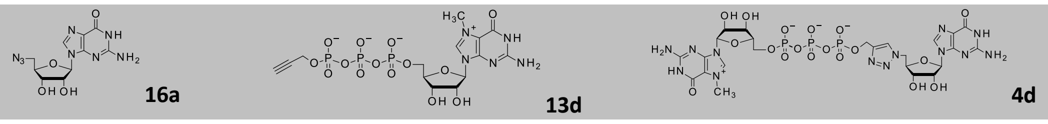 16a	13d	4d	>99%
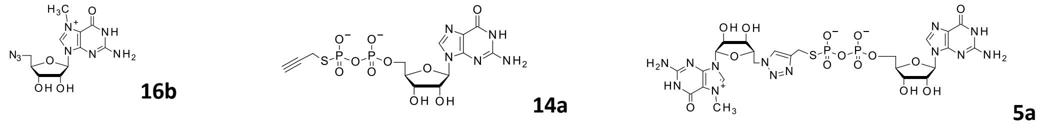 16b	14a	5a	90%
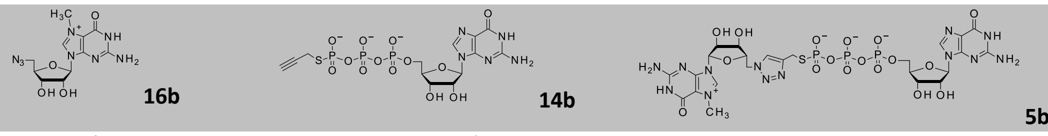 16b	14b	5b	91%
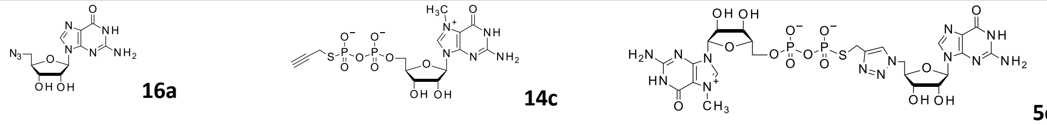 16a	14c	5c	>99%
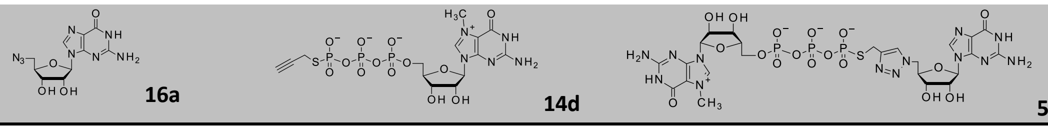 16a	14d	5d	95%(90%)

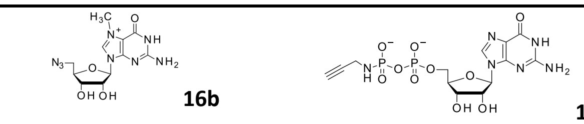 16b	15a	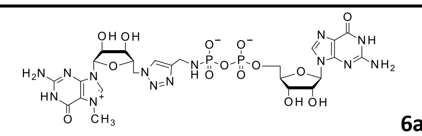 6a	>99%
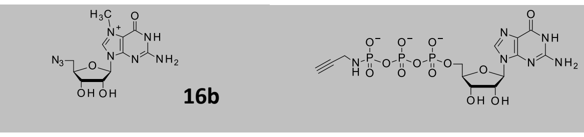 16b	15b	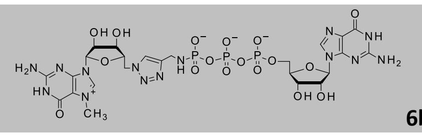 6b	72%
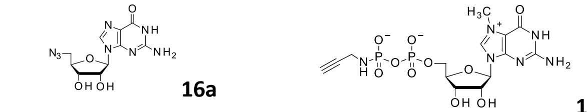 16a	15c	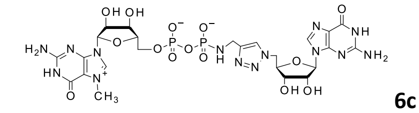 6c	85%
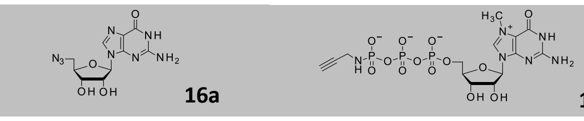 16a	15d	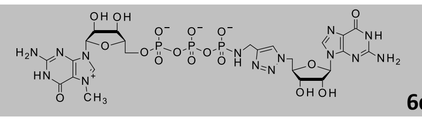 6d	80%
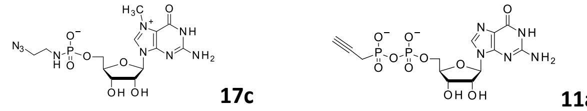 17c	11a	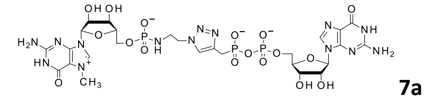 7a	>99%
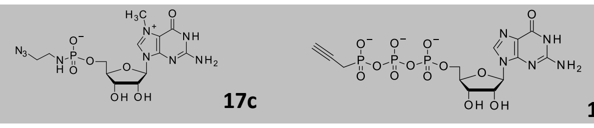 17c	11b	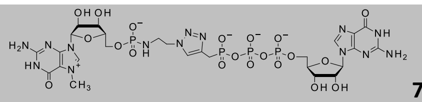 7b	>99%
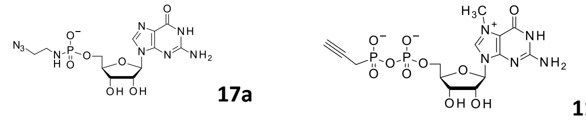 17a	11c	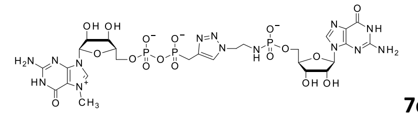 7c	93%
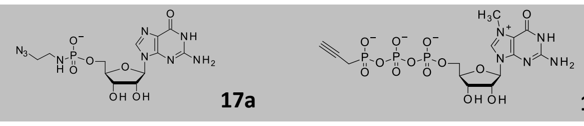 17a	11d	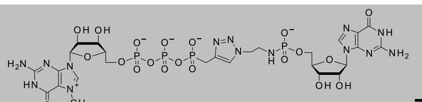 7d	>99%
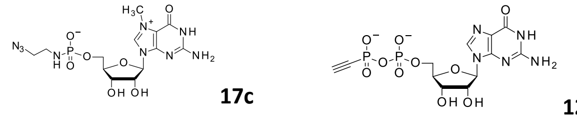 17c	12a	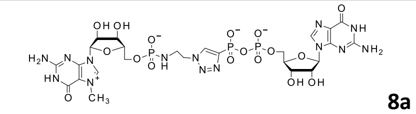 8a	90%
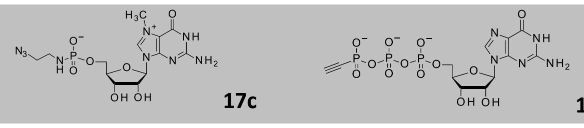 17c	12b	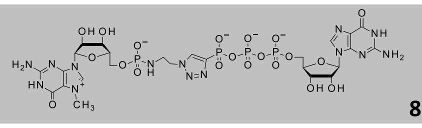 8b	>99%
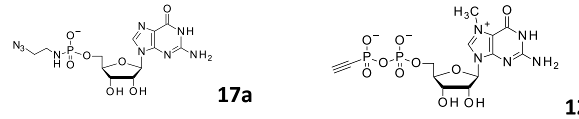 17a	12c	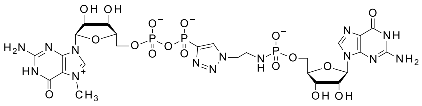 8c	>99%
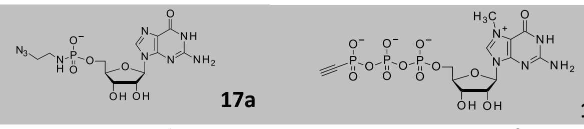 17a	12d	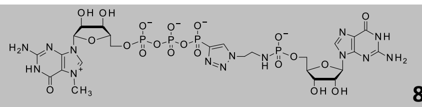 8d	>99%
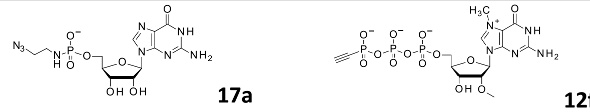 17a	12f	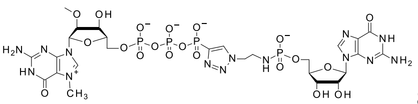 8e	>99%
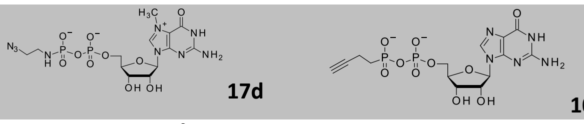 17d	10a	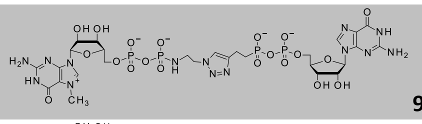 9a	>99%
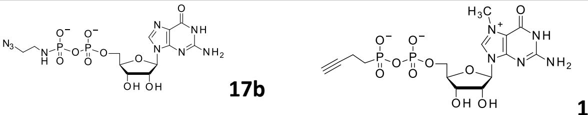 17b	10c	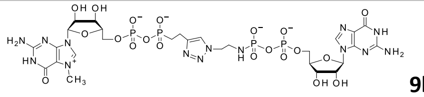 9b	>99%

Table S2. Yields of the syntheses of azide- and alkyne-modified nucleotide analogues.

Starting material	Starting material (nucleotide)	Product	Yield after purification
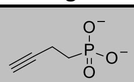 **18a**	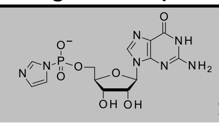 **19a**	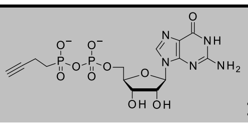 **10a**	70%
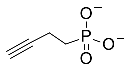 **18a**	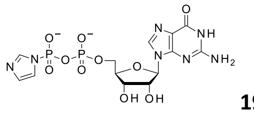 **19b**	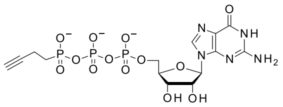 **10b**	85%
**CH_3I**	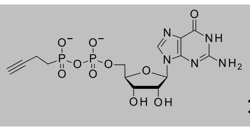 **10a**	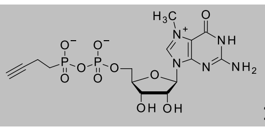 **10c**	68%
**CH_3I**	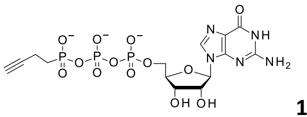 **10b**	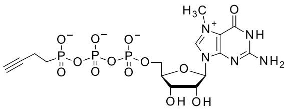 **10d**	74%
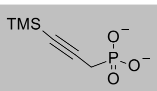 **18b**	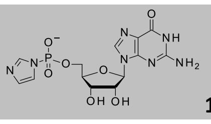 **19a**	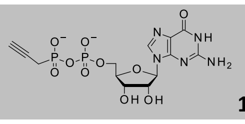 **11a**	72%
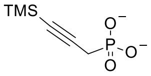 **18b**	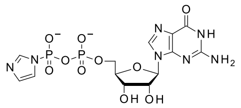 **19b**	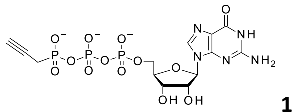 **11b**	90%
**CH_3I**	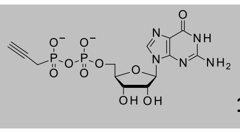 **11a**	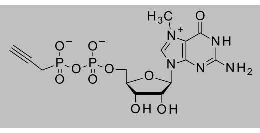 **11c**	60%
**CH_3I**	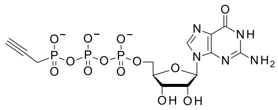 **11b**	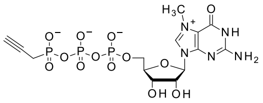 **11d**	67%
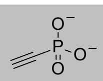 **18c**	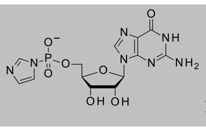 **19a**	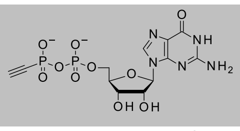 **12a**	45%
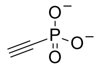 **18c**	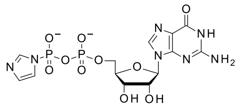 **19b**	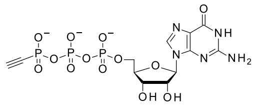 **12b**	56%
**$(CH_3)_2SO_4$**	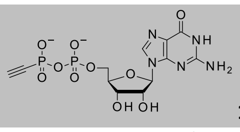 **12a**	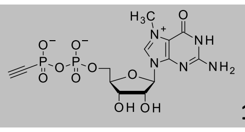 **12c**	51%
**$(CH_3)_2SO_4$**	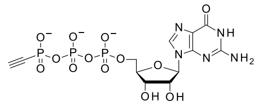 **12b**	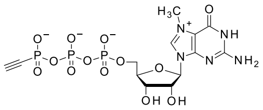 **12d**	59%
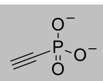 **18c**	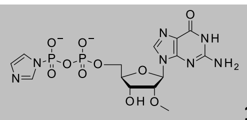 **19h**	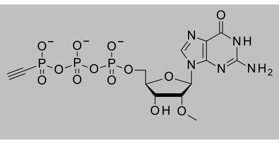 **12e**	93%
**$(CH_3)_2SO_4$**	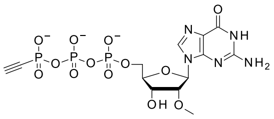 **12e**	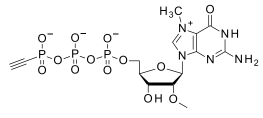 **12f**	79%
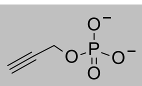 **18d**	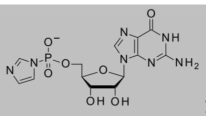 **19a**	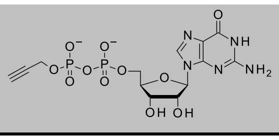 **13a**	72%

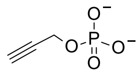 **18d**	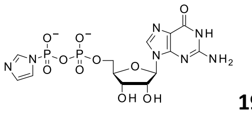 **19b**	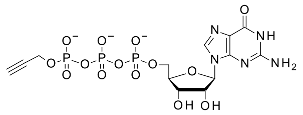 **13b**	75%
**CH_3I**	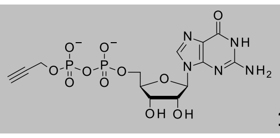 **13a**	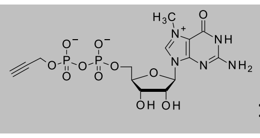 **13c**	80%
**CH_3I**	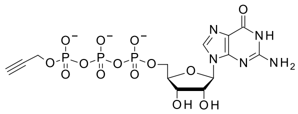 **13b**	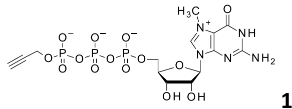 **13d**	74%
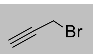	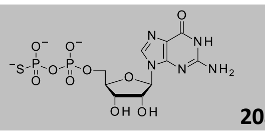 **20a**	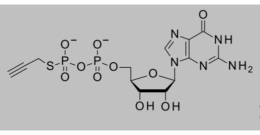 **14a**	54%
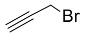	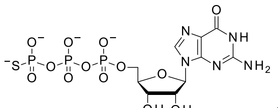 **20b**	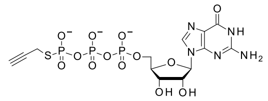 **14b**	74%
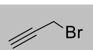	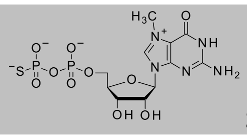 **20c**	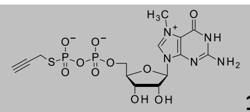 **14c**	66%
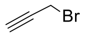	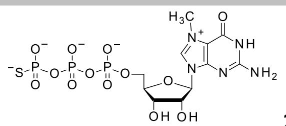 **20d**	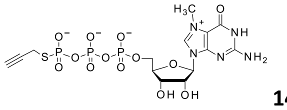 **14d**	74%
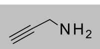	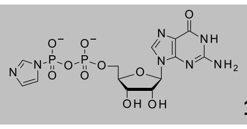 **19b**	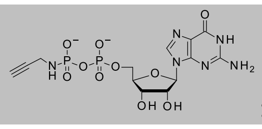 **15a**	92%
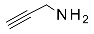	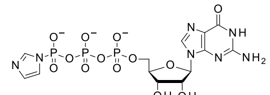 **19c**	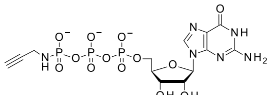 **15b**	65%
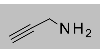	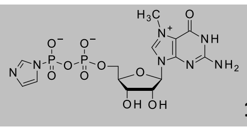 **19e**	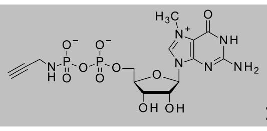 **15c**	68%
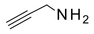	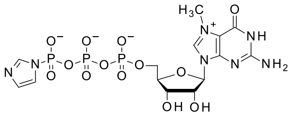 **19f**	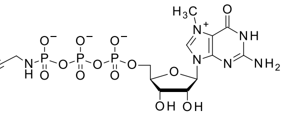 **15d**	70%
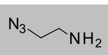	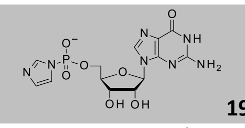 **19a**	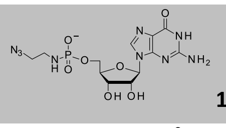 **17a**	83%
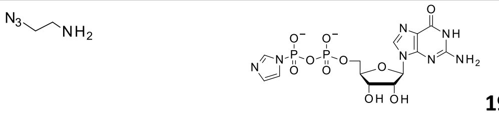	**19b**	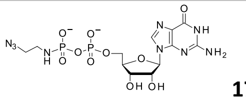 **17b**	86%
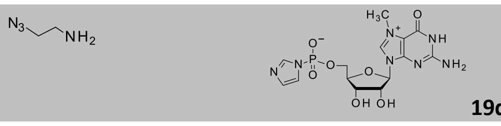	**19d**	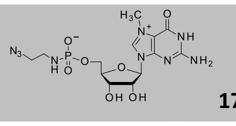 **17c**	35%*
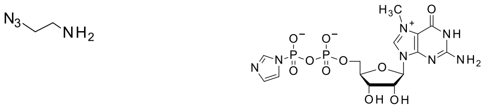	**19e**	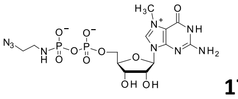 **17d**	49%

*Compound 17c was purified by semi-preparative HPLC directly after synthesis.

Table S3. The susceptibility of triazole-containing cap analogues to degradation by hDcpS determined by an HPLC assay. Conditions: 20 µM analogue, 5 nM hDcpS, 50 mM Tris-HCl, 200 mM KCl, 0.5 mM EDTA, 1 mM DTT. Data for m^7GpppG is average from 5 repetitions as for practical reasons the experiment was performed independently for groups of no more than 8 analogues.

Analogue	Fraction non-degraded at each time point					
	5 min	10 min	15 min	30 min	60 min	24h
**m^7GpppG**	0.90 ± 0.02	0.86 ± 0.03	0.82 ± 0.03	0.74 ± 0.04	0.61 ± 0.09	0.00
**1a**	1.00	1.00	1.00	1.00	1.00	-
**1b**	1.00	1.00	1.00	1.00	1.00	-
**1c**	1.00	1.00	1.00	1.00	1.00	-
**1d**	0.98	0.98	0.97	0.95	0.92	-
**2a**	1.00	1.00	1.00	1.00	1.00	1.00
**2b**	1.00	1.00	1.00	1.00	1.00	1.00
**2c**	1.00	1.00	1.00	1.00	1.00	1.00
**2d**	0.96	0.95	0.94	0.91	0.88	0.46
**3a**	1.00	1.00	1.00	1.00	1.00	1.00
**3b**	1.00	1.00	1.00	1.00	1.00	1.00
**3c**	1.00	1.00	1.00	0.99	0.99	0.97
**3d**	0.94	0.92	0.90	0.87	0.81	0.10
**4a**	1.00	1.00	1.00	1.00	1.00	1.00
**4b**	1.00	1.00	1.00	1.00	1.00	1.00
**4c**	0.98	0.98	0.97	0.96	0.94	0.68
**4d**	0.94	0.92	0.90	0.86	0.78	0.04
**5a**	1.00	1.00	1.00	1.00	1.00	1.00
**5b**	1.00	1.00	1.00	1.00	1.00	1.00
**5c**	0.99	0.99	0.99	0.99	0.98	0.91
**5d**	0.92	0.90	0.89	0.76	0.73	0.01
**6a**	1.00	1.00	1.00	1.00	1.00	-
**6b**	1.00	1.00	1.00	1.00	1.00	1.00
**6c**	1.00	1.00	1.00	1.00	1.00	-
**6d**	0.99	0.99	0.98	0.94	0.93	0.84
**7a**	1.00	1.00	1.00	1.00	1.00	1.00
**7b**	0.99	0.99	0.99	0.99	0.99	0.96
**7c**	1.00	1.00	1.00	1.00	1.00	1.00
**7d**	0.99	0.98	0.98	0.96	0.94	0.44
**8a**	0.97	0.97	0.97	0.97	0.97	0.94
**8b**	1.00	1.00	1.00	1.00	1.00	1.00
**8c**	0.98	0.98	0.97	0.96	0.94	0.74
**8d**	0.96	0.95	0.93	0.90	0.86	0.24
**9a**	1.00	1.00	1.00	1.00	1.00	1.00
**9b**	1.00	1.00	1.00	1.00	1.00	1.00

Table S4. The susceptibility of triazole-containing cap analogues to degradation by hDcpS under high enzyme concentration determined by an HPLC assay.
Conditions: 10 µM analogue, 200 nM hDcpS, 50 mM Tris-HCl, 200 mM KCl, 0.5 mM EDTA, 1 mM DTT.

Analogue	Fraction non-degraded at each time point		
	30 min	60 min	120 min
m^7GpppG	0.00	0.00	0.00
1a	1.00	1.00	1.00
1b	1.00	1.00	1.00
1c	1.00	1.00	1.00
2a	1.00	1.00	1.00
2b	1.00	1.00	1.00
2c	1.00	1.00	1.00
3a	1.00	1.00	1.00
3b	1.00	1.00	1.00
3c	0.79	0.66	0.41
4a	1.00	1.00	1.00
4b	1.00	1.00	1.00
5a	1.00	1.00	1.00
5b	1.00	1.00	1.00
5c	0.15	0.11	0.10
6a	1.00	1.00	1.00
6b	1.00	1.00	1.00
6c	1.00	1.00	1.00
7a	1.00	1.00	1.00
7b	0.73	0.64	0.63
7c	1.00	1.00	1.00
8a	0.69	0.61	0.54
8b	0.65	0.62	0.60
9a	1.00	1.00	1.00
9b	1.00	1.00	1.00

Table S5. Sequences of DNA and RNA oligonucleotides used in this work.

	**Sequence**
**DNA1**	ATACGATTTAGGTGACACTATAGAAGAAGCGGGCATGCGGCCAGCCATAGCCGATCA
**DNA2**	TGATCGGCTATGGCTGGCCGCATGCCCGCTTCTTCTATAGTGTCACCTAAATCGTAT
**DNA3 (DNazyme10-23)**[1]	TGATCGGCTAGGCTAGCTACAACGAGGCTGGCCGC
**RNA1 (35nt)**	GGGAGAGCGGCCGCCAGAUCUUCCGGAUGGCUCGA
**RNA2 (35nt)**	GAAGAAGCGGGCAUGCGGCCAGCCAUAGCCGAUCA
**RNA3 (25nt)**	GAAGAAGCGGGCAUGCGGCCAGCCA

Table S6. Optimization of post-transcriptional RNA capping by CuAAC

**Entry**	**Conditions**			**Capping efficiency**	**RNA degradation**
	**5’ Alkyne-RNA**	**5’ -N_3-m^7G (16 b)**	**Catalyst and solvent**		
1	2.9 µM	14.5 µM (5 equiv.)	$CuSO_4$ (29 µM, 10.equiv.), sodium ascorbate (58 µM, 20 equiv.), THPTA (58 µM, 20 equiv.), H_2O	<1%	Medium
**2**	2.9 µM	72.5 µM (25 equiv.)	$CuSO_4$ (0.14 mM, 50 equiv.), sodium ascorbate (0.29 mM, 100 equiv.), THPTA (0.29 mM, 100 equiv.), H_2O	63%	Medium
**3**	2.9 µM	145 µM (50 equiv.)	$CuSO_4$ (0.29 mM, 100 equiv.), sodium ascorbate (0.58 mM, 200 equiv.), THPTA (0.58 mM, 200 equiv.), H_2O	88%	Medium
**4**	2.9 µM	145 µM (50 equiv.)	CuBr (0.29 mM, 100 equiv.), TBTA (0.58 mM, 200 equiv.), DMSO:t-BuOH 3:1	42%	Low
**5**	2.9 µM	722 µM (250 equiv.)	CuBr (1.45 mM, 500 equiv.), TBTA (2.9 mM, 1000 equiv.), DMSO:t-BuOH 3:1	52%	Low
**6**	2.9 µM	1.45 mM (500 equiv.)	CuBr (2.9 mM, 1000 equiv.), TBTA (5.8 mM, 2000 equiv.), DMSO:t-BuOH 3:1	53%	Low

Figures:

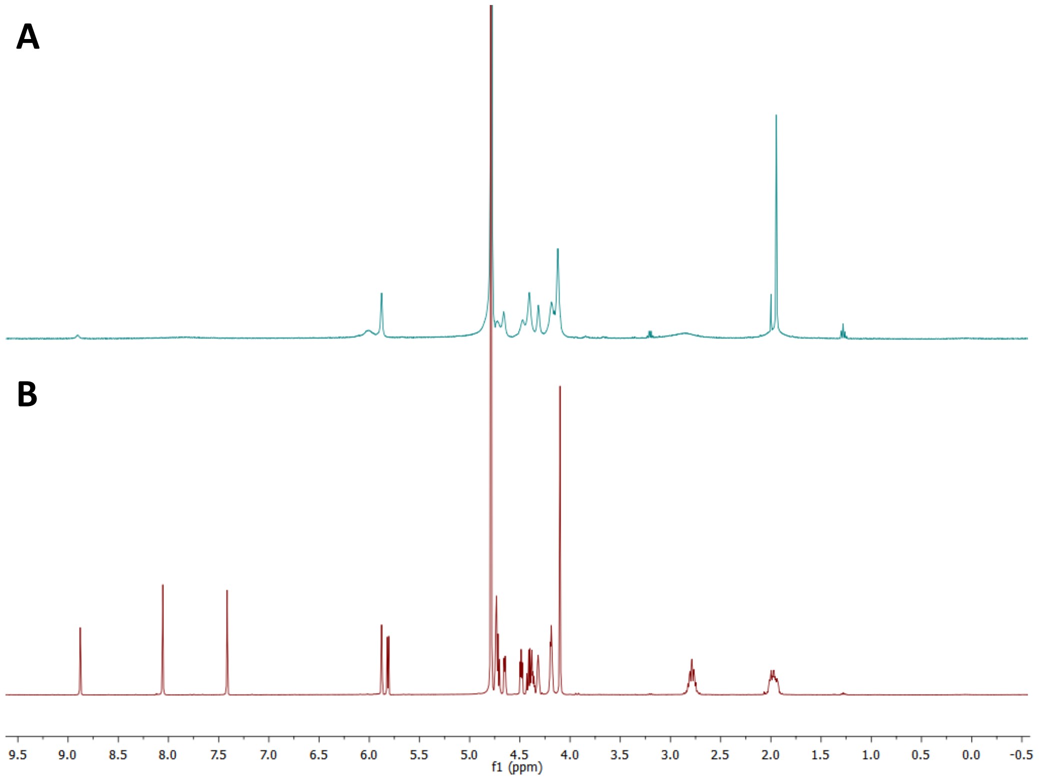

Figure S1. ^{1}H NMR (400 MHz, D_2O, 25 °C) spectra of compound **1a** purified by semi-preparative HPLC without Na_2EDTA (A) and in the presence of Na_2EDTA (B). Significant signal broadening is observed in the presence of residual copper ions.

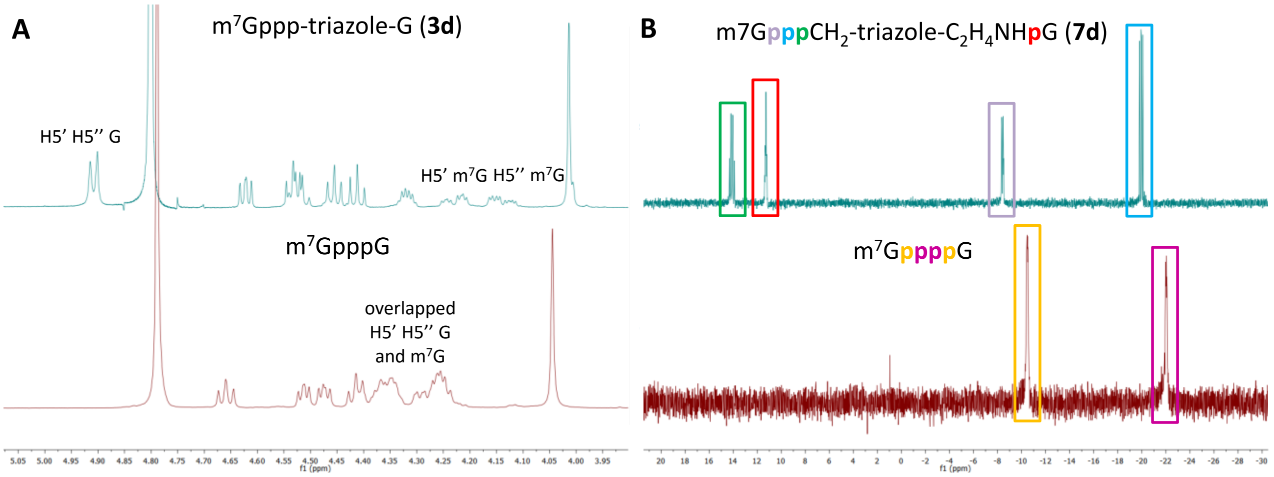

Figure S2. Spectroscopic characterization of triazole-containing cap analogues.

(A) ^{1}H NMR spectra of m^7GpppG and analogue containing triazole directly attached to ribose (**3d**); (B) ^{31}P NMR spectra of m^7GppppG and an analogue containing triazole located between α and β phosphates (**7d**).

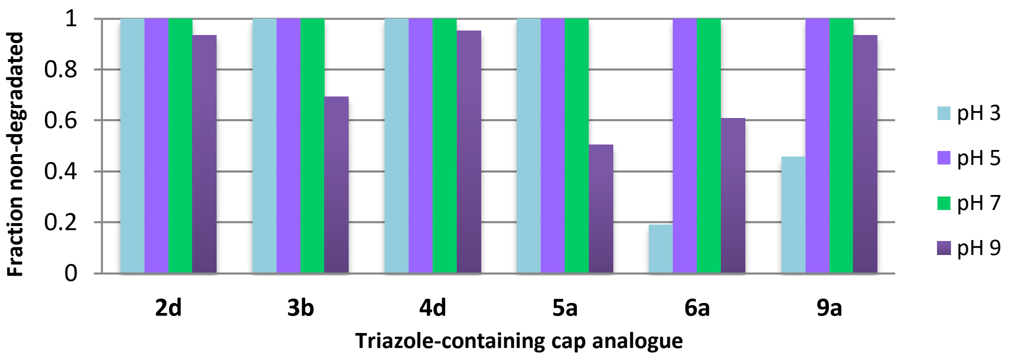

Figure S3. Chemical stability of representative triazole-containing dinucleotide cap analogues after 24h incubation at different pH.

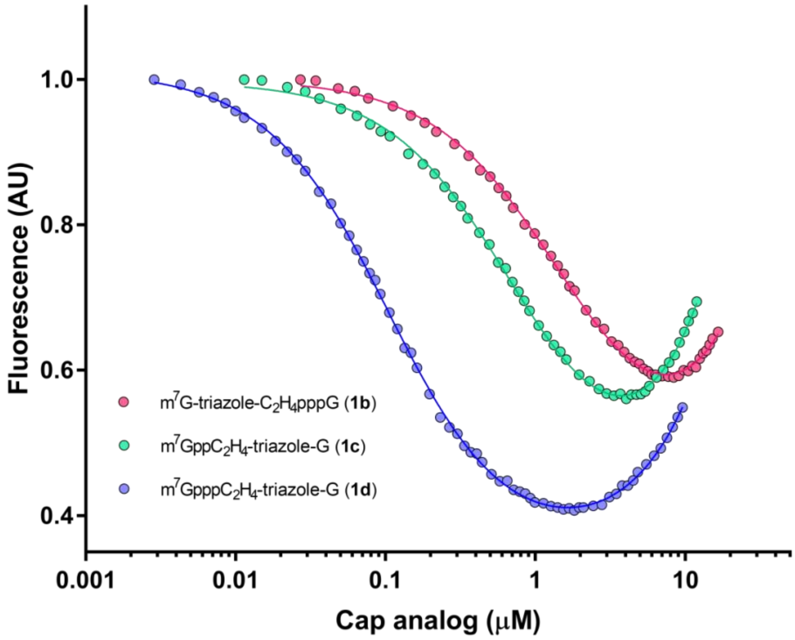

Figure S4. eIF4E fluorescence quenching titration curves for selected cap analogues (**1b**, **1c**, **1d**).

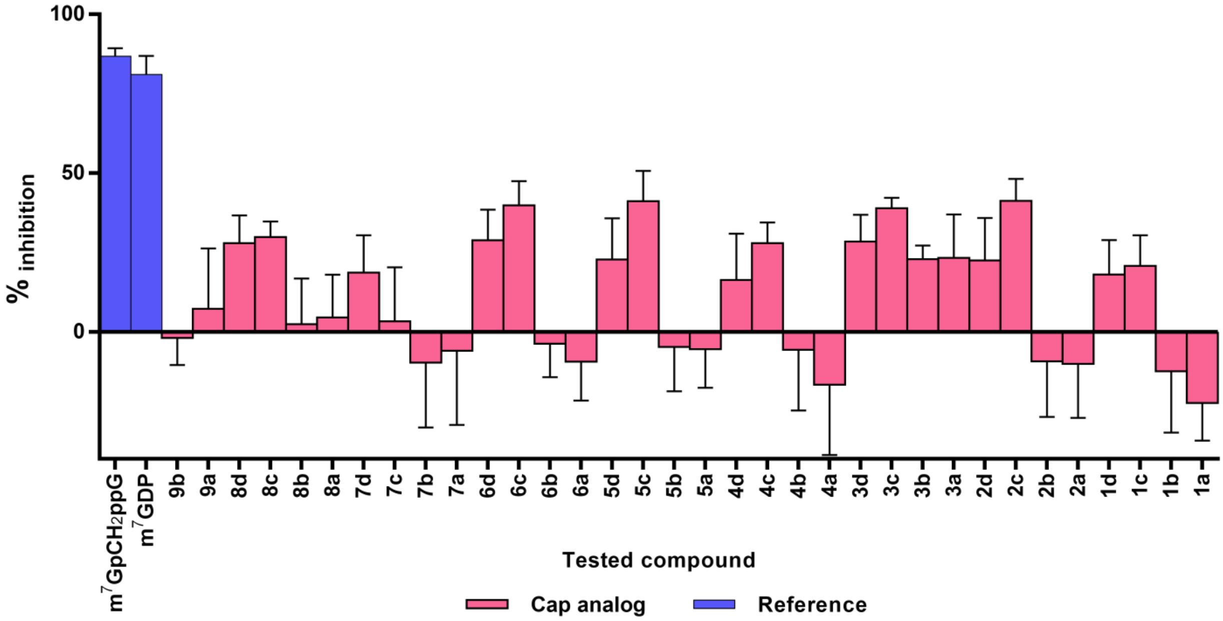

Figure S5. The results of hDcpS screening assay.

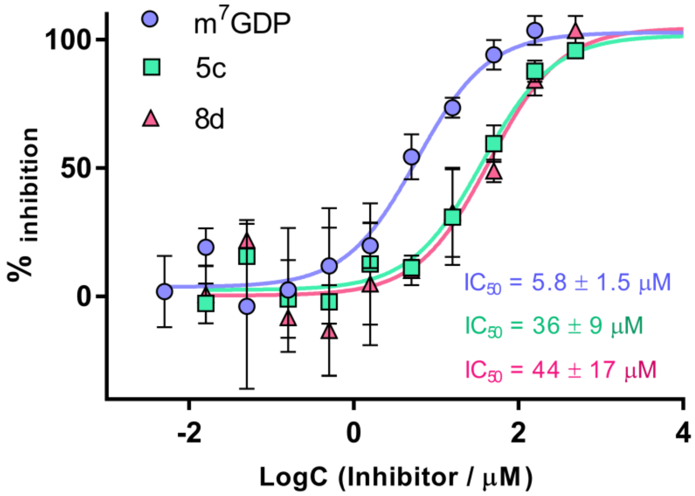

Figure S6. IC_{50} values for inhibition of hDcpS by analogues **5d** and **8d** under the conditions of screening assay.

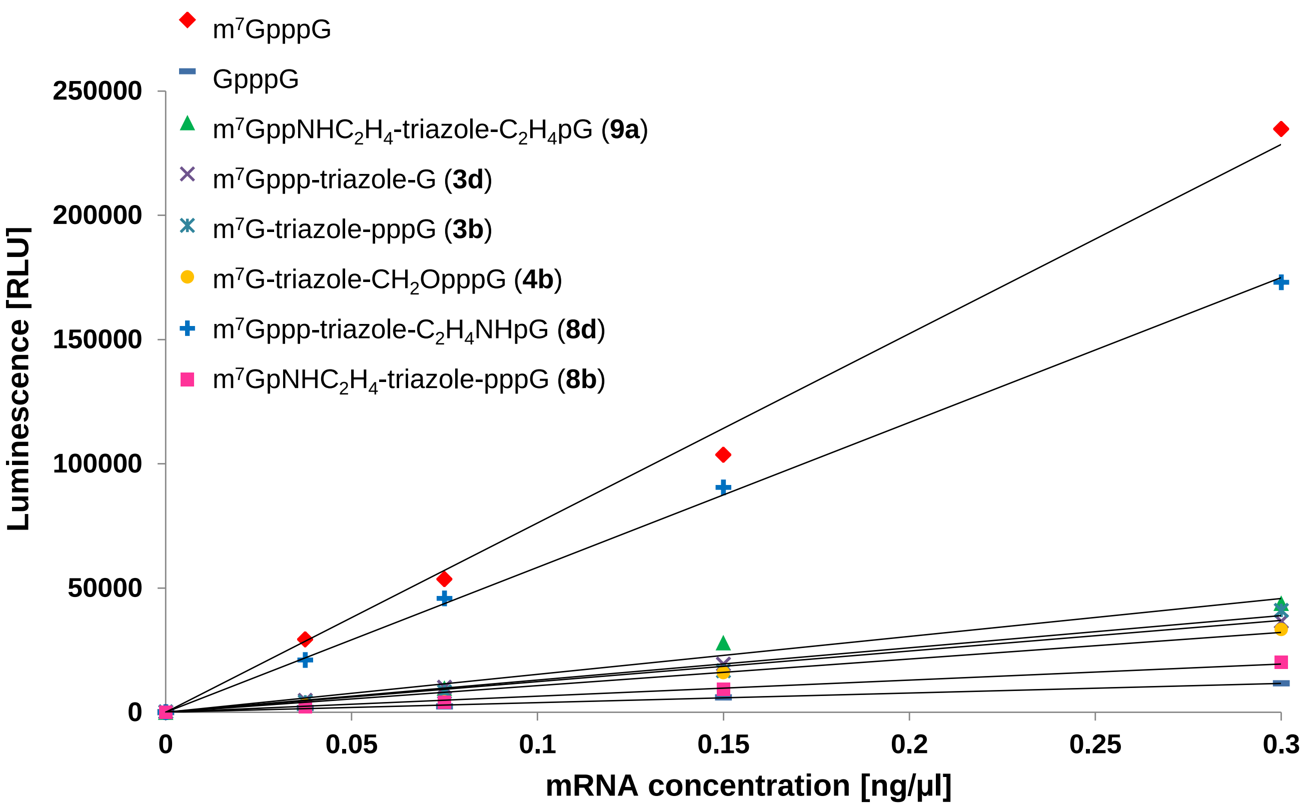

Figure S7. Luciferase activity produced by translation of mRNAs capped with various cap analogues in rabbit reticulocyte lysate – results of a single experiment.

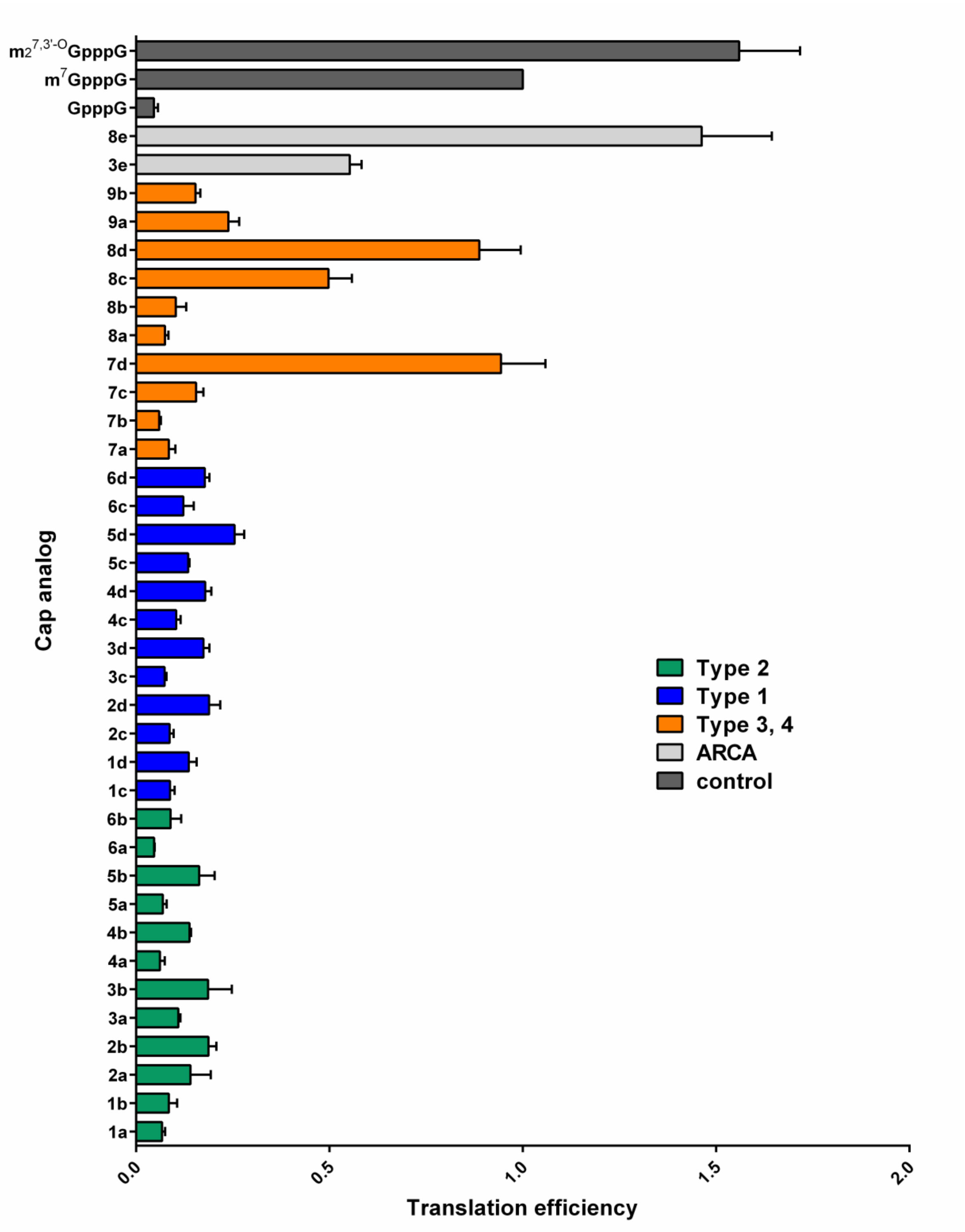

Figure S8. Translation efficiency of luciferase mRNA capped with novel triazole-modified cap analogs.

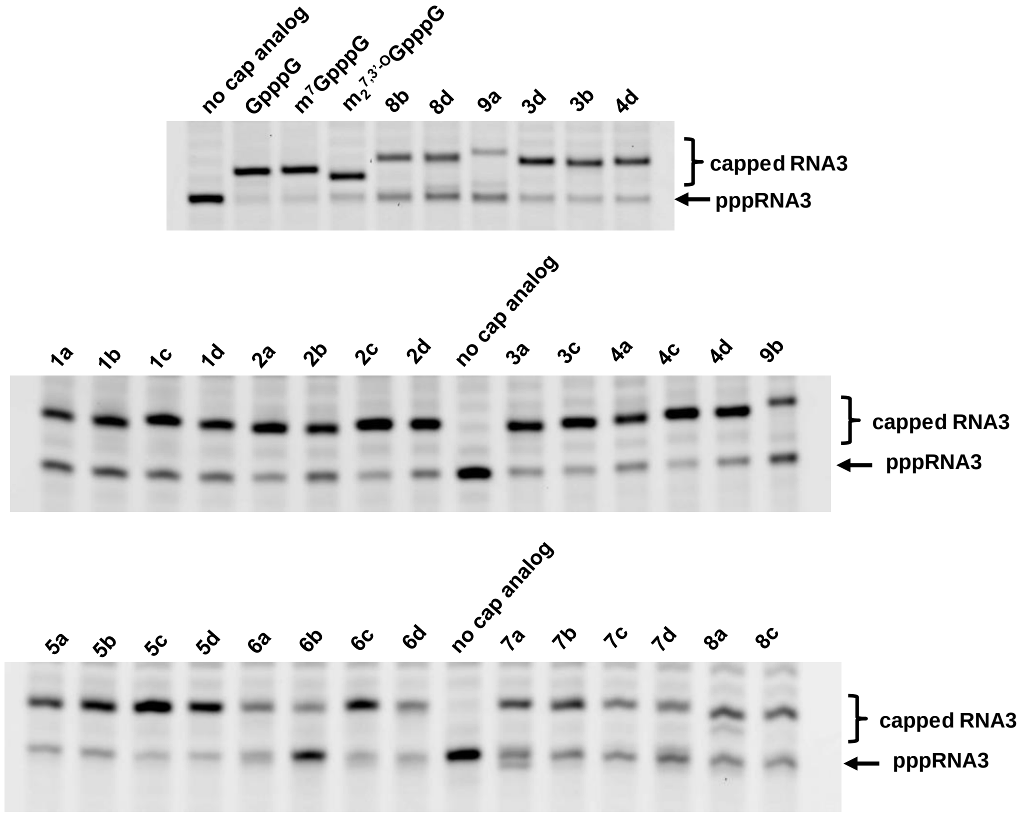

Figure S9. Co-transcriptional capping efficiency of triazole-modified cap analogues.

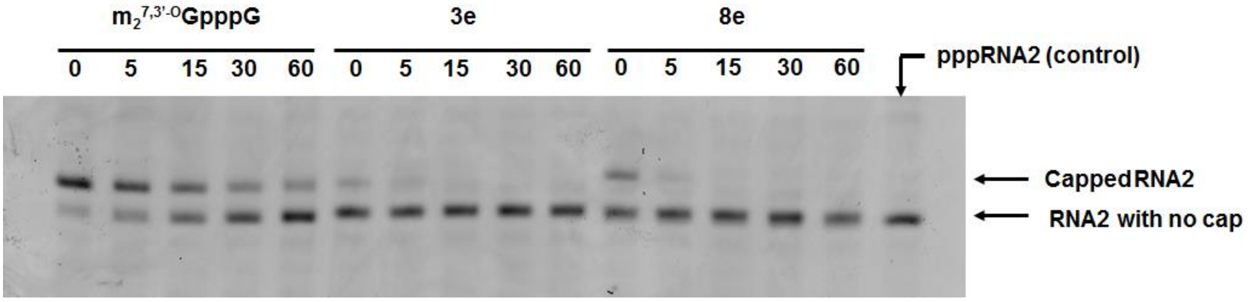

Figure S10. Susceptibility of short transcripts capped with **3e** ($m_2^{7,3'-O}$Gppp-triazole-G) and **8e** ($m_2^{7,3'-O}$Gppp-triazole-C_2H_4NHpG) to hDcp2-catalyzed decapping.

Experimental procedures:

1. General information

1.1 Starting materials and chemical reagents

All solvents and reagents were *synthesis grade* and used without further treatment, unless otherwise stated. Guanosine and guanosine 5'-monophosphate disodium salt were purchased from Carbosynth, 2'-O-methylguanosine from Sigma Aldrich. C-phosphonate nucleotide analogues (**10a-b**, **11a-b**, **12a-b**),[2] 2'-O-methylguanosine 5'-monophosphate[3] and 2-azidoethanamine[4] were synthesized as described previously.

1.2 Chromatography

1.2.1 Ion-exchange chromatography

The synthesized mononucleotide analogues (**10a-d**, **11a-d**, **12a-d**, **13a-d**, **14a-d**, **15a-d** and **17a-d**) were purified by ion-exchange chromatography on DEAE Sephadex A-25 (HCO_3^- form) column. After loading the column with reaction mixture and washing it with water, the products were eluted using different gradients of TEAB in deionized water: 0–0.7 M for nucleoside monophosphates, 0–1.0 M for nucleoside diphosphates or 0–1.2 M nucleoside triphosphates. Fractions containing the desired product were collected together after RP HPLC and spectrophotometric analysis (at 260 nm). Evaporation under reduced pressure with repeated additions of 96% ethanol, then 99.8% ethanol and, at the end, MeCN resulted in isolation of nucleotide analogues as triethylammonium (TEA) salts.

1.2.2 Analytical and preparative reverse-phase (RP) HPLC

Both analytical and semi-preparative HPLC were performed on Agilent Technologies Series 1200 with UV-detection at 254 nm and fluorescence detection (Ex: 260 nm, Em: 370 nm). For chemical and enzymatic reactions, monitoring analytical HPLC was performed using Supelcosil LC-18-T column (4.6 x 250 mm, 5 µm, flow rate 1.3 mL/min) with one of three different linear gradients of methanol in 0.05 M ammonium acetate buffer (pH 5.9): program A – gradient 0–25% of methanol in 15 min, program B – gradient 0–50% of methanol in 15 min, program C – gradient 0–50% of methanol in 7.5 min and then isocratic elution (50% of methanol) until 15 min. For pH-dependent degradation studies and reactions monitoring of different steps of ARCA analogues synthesis analytical HPLC was performed using Grace VisionHT C18-HL column (4.6 x 250 mm, 5µm, flow rate 1.3 mL/min) with linear gradient 0-25% of methanol in 0.05 M ammonium acetate buffer (pH 5.9) in 15 min. Semi-preparative RP HPLC was performed using Discovery RP Amide C-16 HPLC column (25 cm x 21.2 mm, 5 µm, flow rate 5.0 mL/min) with linear gradients of acetonitrile in 0.05 M ammonium acetate buffer (pH 5.9). The products, after at least triple lyophilisation, were isolated as ammonium salts.

1.3 Yields and concentrations determination

The yields of mononucleotide analogues after ion-exchange purification and the concentrations of mono- and dinucleotide analogues solutions used for biophysical and biological experiments were determined on the basis of absorbance measurements performed at 260 nm in 0.1 M phosphate buffer pH 6.0 for 7-methylguanine mononucleotide analogues and in 0.1 M phosphate buffer pH 7.0 for dinucleotide analogues and guanine mononucleotide analogues. The quantities of obtained ion-exchange purified products were expressed as optical density miliunits (opt. mu = absorbance of the solution by volume in mL). For calculations of yields and concentrations following molar extinction coefficients [$M^{-1}cm^{-1}$] were employed: ε = 22600 (dinucleotides), ε = 11400 (m^7G mononucleotides), ε = 12080 (G mononucleotides). Concentrations of transcripts were determined using NanoDrop 2000c Spectrofotometer (Thermo Scientific).

1.4 NMR spectroscopy and mass spectrometry

The structure and purity of each final product were confirmed by high resolution mass spectrometry using negative or positive electrospray ionization (HRMS (-) ESI or HRMS (+) ESI) and ^1H NMR, ^{31}P NMR, gDQCOSY and gHSQCAD spectroscopy. Mass spectra were recorded on Thermo Scientific LTQ OrbitrapVelos spectrometer. NMR spectra were recorded on a Varian INOVA 400 MHz or 500 MHz spectrometer equipped with a high stability temperature unit using 5 mm 4NUC probe, at 25 ^oC if not stated otherwise, at 399.94/500.61 MHz (^1H NMR) and 161.90 MHz (^{31}P NMR). The ^1H NMR and ^{31}P NMR chemical shifts were reported in ppm and referenced to respective internal standards: sodium 3-(trimethylsilyl)-2,2',3,3' tetradeuteropropionate (TSP) and 20% phosphorus acid in D_2O. Signals in ^1H NMR spectra of dinucleotides were assigned according to 2D NMR spectra (gDQCOSY, gHSQCAD). In ^{31}P signal assignment of dinucleotide cap analogues the phosphates were denoted analogously to $m^7Gp_\gamma p_\beta p_\alpha G$.

Phosphoramidate cap analogues (**6a-d**, **7a-9b**) hydrolyzed in D_2O gradually. Although pure compounds (see HPLC profiles, Supporting Information 2) were dissolved in D_2O just before measurements, the spectra indicated some level of hydrolysis. % of hydrolysis is provided along with compound characterization data, if higher than 5%.

2. Chemical synthesis

2.1 Synthesis of nucleotide imidazolide derivatives (19a-h):

2.1.1 Preparation of compounds for imidazole-activation

Preparation of triethylammonium (TEA) salts

The commercially available guanosine 5′-monophosphate (GMP) disodium salt and tetrasodium pyrophosphate were converted into triethylammonium forms by passing their aqueous solutions (ca. 1 g/20 mL) through Dowex 50 W x 8 cationite. The collected eluates were evaporated under reduced pressure with repeated additions of ethanol and acetonitrile to dryness yielding the nucleotide triethylammonium salt as a white solid and triethylammonium pyrophosphate as colorless oil.

Triethylammonium phosphate was prepared by slowly adding triethylamine to the low-concentrated solution of H_2PO_4 in water until pH 7 was obtained which was followed by evaporation to afford oily, colorless residue.

Synthesis of guanosine 5′-diphosphate (GDP), guanosine 5′-triphosphate (GTP) and 2′-O-methylguanosine 5′-diphosphate (m$^{2'-O}$GDP)

Triethylammonium phosphate (4 equiv.) or triethylammonium pyrophosphate (4 equiv.) was suspended in DMF (ca. 0.4 M) in the presence of $ZnCl_2$ (4 equiv.) and stirred for ~5 min to obtain a solution. Then, GMP-Im (**19a**) or m$^{2'-O}$GMP-Im (**19g**) (1 equiv.) along with a second portion of $ZnCl_2$ (4 equiv.) was added and the mixture was stirred for 1-2 h at room temperature. The reaction was stopped by 10-fold dilution with water and addition of EDTA (8 equiv.) and $NaHCO_3$ (ca. 17.6 equiv.). The ion-exchange purification afforded triethylammonium salt of GDP (64%), GTP (79%) and m$^{2'-O}$GDP (95%).

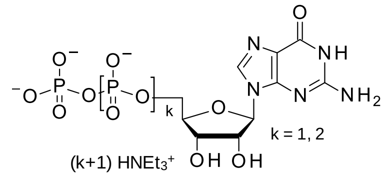

N7-methylation of guanine nucleotides (GMP, GDP and GTP)

An appropriate analogue (GMP, GDP or GTP, TEA salt) was dissolved in dry DMSO to obtain ca. 0.1 M solution followed by addition of CH_3I (8 equiv.). The mixture was stirred at room temperature for several hours until HPLC analysis indicated more than 90% conversion of the substrate and the presence of N^7-methylated nucleotide as the major product. The reaction was stopped by 10-fold dilution with water and organic-soluble compounds were removed by 3-time washing with diethyl ether. The aqueous phase was then treated with a pinch of $Na_2S_2O_5$ to reduce the residual iodine and the pH of solution was set to 7 by addition of solid $NaHCO_3$. The following ion-exchange purification afforded triethylammonium salt of m^{7}GMP (63%), m^{7}GDP (68%) or m^{7}GTP (58%).

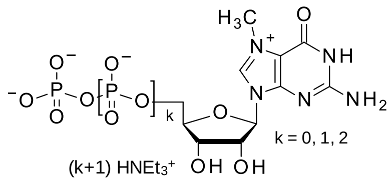

2.1.2 Imidazole-activation

Synthesis of nucleotide imidazolides

Compounds **19a-h** were prepared according to Mukaiyama and Hashimoto.[5] An appropriate nucleotide (1 eq., TEA salt), imidazole (10 equiv.), 2,2′-dithiodipyridine (3 equiv.) were mixed in DMF (~2.5 mL/100 mg of nucleotide) before addition of triethylamine (3 equiv.) and triphenylphosphine (3 equiv.). The mixture was stirred for 6–8 h at room temperature. The addition of a solution of anhydrous $NaClO_4$ (4 equiv.) in dry acetone (~ 8 volumes of DMF volume) resulted in precipitation of the product as sodium salt. The suspension was cooled at 4 °C and the precipitate was filtered off, washed repeatedly with cold, dry acetone and dried in vacuum over P_4O_{10}. Yields 90–100 %.

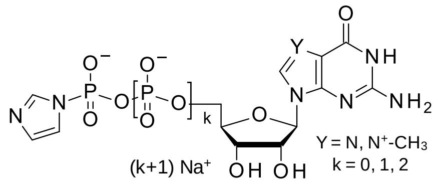

2.2 Synthesis of phosphoester and C-phosphonate analogues

2.2.1 Synthesis of O-(2-propynyl) phosphate ester triethylammonium salt (18d)

Compound **18d** was synthetized according to Lee et al with minor modifications.[6] Phosphorous acid (2.0 g, 24.4 mmol) was dissolved in propargyl alcohol (42.2 mL, 732.0 mmol, 30 equiv) and then triethylamine was added (10.12 mL, 73.2 mmol, 3.0 equiv). After 5 minutes of stirring, iodine (12.3 g, 48.8 mmol, 2.0 equiv) was added in portions. Then reaction was stirred for 30 min and then the mixture of cyclohexylamine (14.0 mL, 122 mmol, 5 equiv) in acetone (633 mL) was added. The white precipitate was centrifuged, and after removing the supernatant, resuspended in acetone. Procedure was repeated three times, then the precipitate

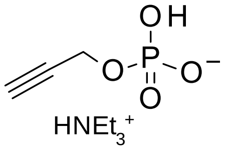

was dried over P_2O_5 in vacuum overnight. Using ion exchange chromatography the cyclohexylammonium salt was converted into the triethylamine salt to afford a yellowish oil (6.00 g, 17.8 mmol, 73%).

^{1}H NMR (400 MHz, D_2O, 25 °C) δ_H: 4.50 (2H, *J* = 9.6, 2.6, dd, CH_2), 3.18 (6H, *J* = 7.4, q, TEA-CH_2), 2.88 (1H, *J* = 2.4, t, CCH), 1.25 (9H, *J* = 7.4, t, TEA-CH_3); ^{31}P NMR (162 MHz, D_2O, 25°C) δ_P: 3.16 (1P, t, *J* = 9.6); HRMS (-) ESI *m/z* found: 134.9839, calc. for $C_3H_4O_4P^-$: 134.9853.

2.3.2. Synthesis of phosphoester nucleotide analogues

General procedure A (GP A): Coupling of nucleotide imidazolides with phosphoester subunit (18d)

Compound **18d** (3 equiv.) was stirred in DMF until complete dissolution (to ~0.3 M concentration). Then, **19a** or **19b** (1 equiv.) along with $ZnCl_2$ (8 equiv.) was added and the mixture was stirred for 1-2 h at room temperature. The reaction was stopped by 10-fold dilution with water and addition of EDTA (8 equiv.) and $NaHCO_3$ (ca. 17.6 equiv.). The product was purified by ion-exchange chromatography on DEAE Sephadex A-25 and evaporated to dryness as described in General Information. Prior to NMR characterization the product was additionally purified by semi-preparative HPLC as described in General Information.

General procedure B (GP B): *N*^{7}-methylation of guanine nucleotides using CH_3I

An appropriate nucleotide (TEA salt) was dissolved in dry DMSO to obtain ca. 0.1 M solution followed by addition of CH_3I (8 equiv.). The mixture was stirred at room temperature for several hours until HPLC analysis indicated more than 90% conversion of the substrate and the presence of *N*^{7}-methylated nucleotide as the major product. The reaction was stopped by 10-fold dilution with water and organic-soluble compounds were removed by 3-time washing with diethyl ether. The aqueous phase was then treated with a pinch of $Na_2S_2O_5$ to reduce the residual iodine and the pH of solution was set to 7 by addition of solid $NaHCO_3$. The product was purified by ion-exchange chromatography on DEAE Sephadex A-25 and evaporated to dryness as described in General Information. Prior to NMR characterization the product was additionally purified by semi-preparative HPLC as described in General Information.

General procedure C (GP C): *N*^{7}-methylation of guanine nucleotides using $(CH_3)_2SO_4$

An appropriate nucleotide (TEA salt) was dissolved at ~0.2 M concentration in ca. 0.5 mM aqueous CH_3COOH (pH 4) to obtain ca. 0.2 M solution. Then, 5 portions of $(CH_3)_2SO_4$ (2 equiv. each) were added every 10 min to the mixture under vigorous stirring and the pH was maintained at 4 by adding 10% KOH if necessary. The stirring was continued at room temperature for several hours until HPLC analysis indicated more than 90% conversion of the substrate and the presence of N^7-methylated nucleotide as the major product. The reaction was stopped by 10-fold dilution with water and organic-soluble compounds were removed by 3-time washing with diethyl ether. The pH of aqueous phase was then set to 7 by addition of solid $NaHCO_3$. The product was purified by ion-exchange chromatography on DEAE Sephadex A-25 and evaporated to dryness as described in General Information. Prior to NMR characterization the product was additionally purified by semi-preparative HPLC as described in General Information.

(10a-b, 11a-b, 12a-b) β-C-(2-ethynyl), β-C-(2-propargyl) and β-C-(3-butynyl) guanosine diphosphate and γ-C-(2-ethynyl), γ-C-(2-propargyl) and γ-C-(3-butynyl) guanosine triphosphate triethylammonium salts

Compounds **10a-b**, **11a-b**, **12a-b** were obtained according to Wanat et al.[2] Briefly, triethylammonium 3-butynyl C-phosphonate (**18a**), tributylammonium 3-trimethylsilyl-1-propargyl C-phosphonate (**18b**) or triethylammonium 2-ethynyl C-phosphonate (**18c**) (each 2.5 equiv.) was stirred in DMF (ca. 0.4 M) until complete dissolution. Then, **19a** or **19b** (1 equiv.) along with $MgCl_2$ (8 equiv.) were added and the mixture was stirred for 1-2 h at room temperature. The reaction was stopped by 10-fold dilution with water. The product was purified by ion-exchange chromatography on DEAE Sephadex A-25 and evaporated to dryness as described in General Information to afford products in a form of triethylammonium salts. Trimethylsililpropargylophosphonate nucleotide analogues were then deprotected by incubation in TBAF/THF (1 M) : ACN (1:3, v/v) mixture (1.1 equiv. of TBAF per nucleotide) at room temperature for 24 h. After removing the solvent under reduced pressure, the crude product was directly subjected to click reactions. Yields: 45-90%.

(13a) β-O-(2-propargyl) guanosine diphosphate ammonium salt

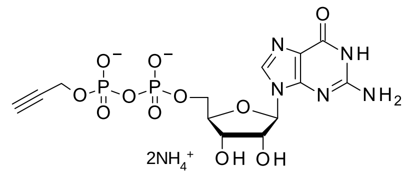

Obtained according to GP A starting from O-(2-propynyl) phosphate ester (**18d**) (1.788 mmol), guanosine 5'-monophosphate P-imidazolide (**19a**) (300 mg, 7200 mOD, 0.596 mmol), $ZnCl_2$ (651 mg, 4.768 mmol) and DMF (6.0 mL). The ion-exchange purification afforded 5184 mOD (0.429 mmol, 72%) of **13a** triethylammonium salt. Additional HPLC purification of a fraction of obtained product gave **13a** as ammonium salt.

^{1}H NMR (400 MHz, D_2O, 25 °C) δ_H: 8.11 (1H, s, H8), 5.94 (1H, dd, $J_{1'-2'}$ = 6.3, H1'), 4.82 (1H, dd, $J_{1'-2'}$ = 6.3, $J_{2'-3'}$ = 5.1, H2'), 4.53-4.56 (3H, overlapped H3', $H_{CH2'}$ and $H_{CH2''}$), 4.35-4.37 (1H, m, H4'), 4.21-4.23 (2H, m, H5' and H5''), 2.84 (1H, dd, $J_{CH2'-CH}$ = 2.4, $J_{CH2''-CH}$ = 2.7, H_{CH}); ^{31}P NMR (162 MHz, D_2O, 25°C) δ_P: -10.63-(-10.56) (2P, overlapped Pα and Pβ); HRMS (-) ESI *m/z* found: 480.0331, calc. for $C_{13}H_{16}N_5O_{11}P_2^-$: 480.0322.

(13b) γ-O-(2-propargyl) guanosine triphosphate ammonium salt

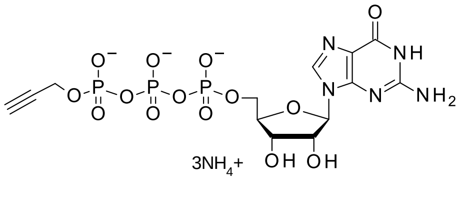

Obtained according to GP A starting from O-(2-propynyl) phosphate ester (**18d**) (1.118 mmol), guanosine 5'-diphosphate P-imidazolide (**19b**) (250 mg, 4500 mOD,

0.372 mmol), $ZnCl_2$ (407 mg, 2.980 mmol) and DMF (3.7 mL). The ion-exchange purification afforded 3375 mOD (0.279 mmol, 75%) of **13b** triethylammonium salt. Additional HPLC purification of a fraction of obtained product gave **13b** as ammonium salt.

^{1}H NMR (400 MHz, D_2O, 25 °C) δ_H: 8.12 (1H, s, H8), 5.93 (1H, dd, $J_{1'-2'}$ = 6.7, H1'), 4.84 (1H, dd, $J_{1'-2'}$ = 6.9, $J_{2'-3'}$ = 4.7, H2'), 5.58 (2H, dd, $J_{CH2-P\gamma}$ = 9.4, J_{CH2-CH} = 2.4, H_{CH2}), 4.56 (1H, dd, $J_{2'-3'}$ = 4.7, $J_{3'-4'}$ = 3.1, H3'), 4.35-4.38 (1H, m, H4'), 4.23-4.26 (2H, m, H5' and H5''), 2.83 (1H, t, J_{CH2-CH} = 2.4, H_{CH}); ^{31}P NMR (162 MHz, D_2O, 25°C) δ_P: -10.89-(-10.60) (2P, overlapped Pα and Pγ), -22.44 (1P, t, $J_{P\beta-P\alpha} = J_{P\beta-P\gamma}$ = 19.1, Pβ); HRMS (-) ESI *m/z* found: 559.9995, calc. for $C_{13}H_{17}N_5O_{14}P_3^-$: 559.9985.

(13c) β-O-(2-propargyl) 7-methylguanosine diphosphate ammonium salt

Obtained according to GP B starting from β-O-(2-propargyl) guanosine diphosphate (**13a**) (3000 mOD, 0.248 mmol), CH_3I (0.124 mL, 1.987 mmol) and DMSO (2.5 mL). The ion-exchange purification afforded 2262 mOD (0.198 mmol, 80%) of **13c** triethylammonium salt. Additional HPLC purification of a fraction of obtained product gave **13c** as ammonium salt.

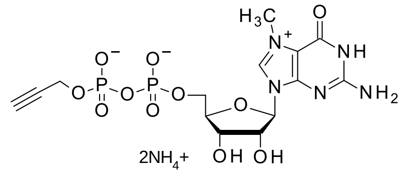

^{1}H NMR (400 MHz, D_2O, 25 °C) δ_H: 6.08 (1H, dd, $J_{1'-2'}$ = 3.9, H1'), 4.69 (1H, dd, $J_{1'-2'}$ = 3.9, $J_{2'-3'}$ = 4.7, H2'), 4.57-4.60 (2H, m, H_{CH2}), 4.51 (1H, dd, $J_{2'-3'}$ = 4.7, $J_{3'-4'}$ = 5.5, H3'), 4.40-4.43 (1H, m, H4'), 4.36 (1H, ddd, $J_{5'-5''}$ = 11.9, J = 3.9, 2.4, H5'), 4.23 (1H, ddd, $J_{5'-5''}$ = 11.9, J = 5.1, 2.4, H5'), 4.13 (3H, s, m^7), 2.87 (1H, t, J_{CH2-CH} = 2.4, H_{CH}); ^{31}P NMR (162 MHz, D_2O, 25°C) δ_P: -10.53-(-10.46) (2P, overlapped Pα and Pβ); HRMS (-) ESI *m/z* found: 494.0488, calc. for $C_{14}H_{18}N_5O_{11}P_2^-$: 494.0478.

(13d) γ-O-(2-propargyl) 7-methylguanosine triphosphate ammonium salt

Obtained according to GP B starting from γ-O-(2-propargyl) guanosine triphosphate (**13b**) (1863 mOD, 0.154 mmol), CH_3I (0.077 mL, 1.234 mOD) and DMSO (1.5 mL). The ion-exchange purification afforded 1299 mOD (0.114 mmol, 74%) of **13d** triethylammonium salt. Additional HPLC purification of a fraction of obtained product gave **13d** as ammonium salt.

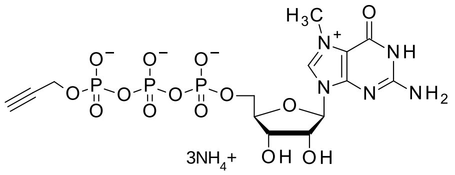

^{1}H NMR (400 MHz, D_2O, 25 °C) δ_H: 6.08 (1H, dd, $J_{1'-2'}$ = 3.9, H1'), 4.69 (1H, dd, $J_{1'-2'}$ = 3.9, $J_{2'-3'}$ = 4.7, H2'), 4.57-4.60 (2H, m, H_{CH2}), 4.51 (1H, dd, $J_{2'-3'}$ = 4.7, $J_{3'-4'}$ = 5.5, H3'), 4.40-4.43 (1H, m, H4'), 4.36 (1H, ddd, $J_{5'-5''}$ = 11.9, J = 3.9, 2.4, H5'), 4.23 (1H, ddd, $J_{5'-5''}$ = 11.9, J = 5.1, 2.4, H5'), 4.13 (3H, s, m^7), 2.87 (1H, t, J_{CH2-CH} = 2.4, H_{CH}); ^{31}P NMR (162 MHz, D_2O, 25°C) δ_P: -10.87-(-10.63) (2P, overlapped Pα and Pγ), -22.34 (1P, dd, J = 19.1, 20.5 Hz); HRMS (-) ESI *m/z* found: 574.0150, calc. for $C_{14}H_{19}N_5O_{14}P_3^-$: 574.0141.

(10c) β-C-(3-butynyl) 7-methylguanosine diphosphate ammonium salt

Obtained according to GP B starting from β-C-(3-butynyl) guanosine diphosphate triethylammonium salt (**10a**) (11000 mOD, 0.910 mmol), CH_3I (0.454 mL, 7.285 mmol) and DMSO (9.0 mL). The ion-exchange purification afforded 7061 mOD (0.619 mmol, 68%) of **10c** triethylammonium salt. Additional HPLC purification of a fraction of obtained product gave **10c** as ammonium salt.

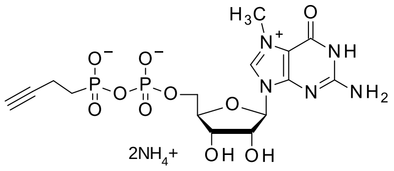

^{1}H NMR (400 MHz, D_2O, 25°C) δ_H: 9.21 (1H, s, H8), 6.08 (1H, d, $J_{1'-2'}$ = 3.6, H1'), 4.70 (1H, dd, $J_{1'-2'}$ = 3.6, $J_{2'-3'}$ = 4.7, H2'), 4.51 (1H, dd, $J_{2'-3'}$ = 4.7, $J_{3'-4'}$ =5.5, H3'), 4.39-4.43 (1H, m, H4'), 4.34 (1H, ddd, $J_{5'-5''}$ = 12.0, J = 4.2, 2.5, H5'), 4.22 (1H, ddd, $J_{5'-5''}$ = 12.0, J = 5.2, 2.2, H5''), 4.13 (3H, s, m^7), 2.46 (2H, dtd, $J_{CH2(C2H)-CH}$ = 2.6, $J_{CH2(C2H)-CH2(P)}$ = 8.1, $J_{CH2(C2H)-P\beta}$ = 10.1, $H_{CH2(C2H)}$), 2.34 (1H, t, $J_{CH-CH2(C2H)}$ = 2.6, H_{CH}), 1.94 (2H, dt, $J_{CH2(C2H)-CH2(P)}$ = 8.1, $J_{CH2(P)-P\beta}$ = 16.9, $H_{CH2(P)}$); ^{31}P NMR (162 MHz, D_2O, 25°C) δ_P: 16.45 (1P, dtt, $J_{CH2(C2H)-P\beta}$ = 10.1, $J_{CH2(P)-P\beta}$ = 16.9, $J_{P\alpha-P\beta}$ = 26.6, Pβ), -11.34 (1P, br d, $J_{P\alpha-P\beta}$ = 26.6, Pα); HRMS (-) ESI *m/z* found: 492.0692, calc. for $C_{15}H_{20}N_5O_{10}P_2^-$: 492.0685.

(10d) γ-C-(3-butynyl) 7-methylguanosine triphosphate ammonium salt

Obtained according to GP B starting from γ-C-(3-butynyl) guanosine triphosphate triethylammonium salt (**10b**) (11000 mOD, 0.910 mmol), CH_3I (0.454 mL, 7.285 mmol) and DMSO (9.0 mL). The ion-exchange purification afforded 7677 mOD (0.673 mmol, 74%) of **10d** triethylammonium salt. Additional HPLC purification of a fraction of obtained product gave **10d** as ammonium salt.

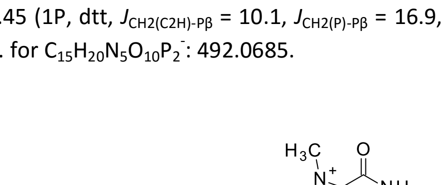

^{1}H NMR (400 MHz, D_2O, 25°C) δ_H: 9.23 (1H, s, H8), 6.08 (1H, d, $J_{1'-2'}$ = 3.5, H1'), 4.69 (1H, dd, $J_{1'-2'}$ = 3.5, $J_{2'-3'}$ = 4.7, H2'), 4.54 (1H, dd, $J_{2'-3'}$ = 4.7, $J_{3'-4'}$ = 5.5, H3'), 4.35-4.42 (2H, overlapped H4' and H5'), 4.26 (1H, ddd, $J_{5'-5''}$ = 12.0, J = 5.2, 1.7, H5''), 4.13 (3H, s, m^7), 2.44 (2H, dtd, $J_{CH-CH2(C2H)}$ = 2.5, $J_{CH2(P)-CH2(C2H)}$= 8.3, $J_{CH2(C2H)-P\gamma}$ = 11.2, $H_{CH2(C2H)}$), 2.26 (1H, t, $J_{CH-CH2(C2H)}$ = 2.5, H_{CH}), 1.99 (2H, dt, $J_{CH2(P)-CH2(C2H)}$= 8.3, $J_{CH2(P)-P\gamma}$ = 16.7, $H_{CH2(P)}$); ^{31}P NMR (162 MHz, D_2O, 25°C) δ_P: 16.46 (1P, dtt, $J_{CH2(C2H)-P\gamma}$ = 11.2, $J_{CH2(P)-P\gamma}$ = 16.7, $J_{P\beta-P\gamma}$ = 25.0, Pγ), -11.47 (1P, br d, $J_{P\alpha-P\beta}$ = 19.2, Pα), -23.10 (1P, dd, $J_{P\alpha-P\beta}$ = 19.2, $J_{P\beta-P\gamma}$ = 25.0, Pβ); HRMS (-) ESI *m/z* found: 572.0357, calc. for $C_{15}H_{21}N_5O_{13}P_3^-$: 572.0349.

(11c) β-C-(2-propargyl) 7-methylguanosine diphosphate ammonium salt

Obtained according to GP B starting from β-C-(2-propargyl) guanosine diphosphate triethylammonium salt (**11a**) (8310 mOD, 0.688 mmol), CH_3I 0.343 mL, 5.503 mmol) and DMSO (7.0 mL). The ion-exchange purification afforded 4706 mOD (0.413 mmol, 60%) of **11c** triethylammonium salt. Additional HPLC purification of a fraction of obtained product gave **11c** as ammonium salt.

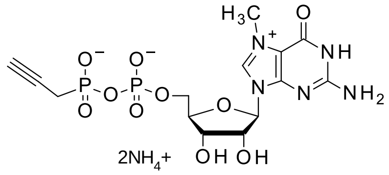

^{1}H NMR (400 MHz, D_2O, 25°C) δ_H: 6.06 (1H, d, $J_{1'-2'}$ = 3.5, H1'), 4.68 (1H, dd, $J_{1'-2'}$ = 3.5, $J_{2'-3'}$ = 5.0, H2'), 4.50 (1H, dd, $J_{2'-3'}$ = 5.0, $J_{3'-4'}$ = 5.5, H3'), 4.40-4.42 (1H, m, H4'), 4.36 (1H, ddd, $J_{5'-5''}$ =11.8, J = 4.0, 2.4, H5'), 4.23 (1H, ddd, $J_{5'-5''}$ =11.8, J =5.4, 2.0, H5''), 4.12 (3H, s, m^7), 2.76 (2H, dd, $J_{CH2-P\beta}$ = 21.4, J_{CH2-CH} = 2.7, H_{CH2}), 2.34 (1H, dt, J_{CH-CH2} = 2.7, $J_{CH-P\beta}$ = 6.5, H_{CH}); ^{31}P NMR (162 MHz, D_2O, 25°C) δ_P: 9.44 (1P, dtd, $J_{P\alpha-P\beta}$ = 24.2, $J_{CH2-P\beta}$ = 21.4, $J_{CH-P\beta}$ = 6.5, Pβ), -10.76 (1P, br d, $J_{P\alpha-P\beta}$ = 24.2, Pα); HRMS (-) ESI *m/z* found: 478.0521, calc. for $C_{14}H_{18}N_5O_{10}P_2^-$: 478.0529.

(11d) γ-C-(2-propargyl) 7-methylguanosine triphosphate ammonium salt

Obtained according to GP B starting from γ-C-(2-propargyl) guanosine triphosphate triethylammonium salt (**11b**) (10000 mOD, 0.828 mmol), CH_3I (0.412 mL, 6.622 mmol) and DMSO (8.0 mL). The ion-exchange purification afforded 6324 mOD (0.555 mmol, 67%) of **11d** triethylammonium salt. Additional HPLC purification of a fraction of obtained product gave **11d** as ammonium salt.

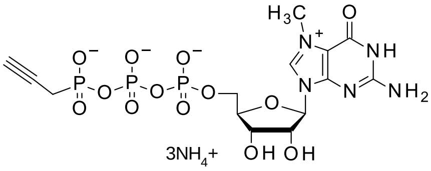

^{1}H NMR (400 MHz, D_2O, 25°C) δ_H: 6.06 (1H, d, $J_{1'-2'}$ = 3.49, H1'), 4.69 (1H, dd, $J_{1'-2'}$ = 3.49, $J_{2'-3'}$ = 4.73, H2'), 4.53 (1H, dd, $J_{2'-3'}$ = 4.73, $J_{3'-4'}$ = 5.48, H3'), 4.35-4.42 (1H, overlapped H4' and H5'), 4.24-4.28 (1H, ddd, $J_{5'-5''}$ =11.7, J = 5.2, 2.0, H5''), 4.13 (3H, s, m^7), 2.78 (2H, dd, $J_{CH2-P\gamma}$ =21.7, J_{CH2-CH} = 2.5, H_{CH2}), 2.34 (1H, dt, J_{CH2-CH} = 2.5, $J_{CH-P\gamma}$ = 6.5, H_{CH}); ^{31}P NMR (162 MHz, D_2O, 25°C) δ_P: 9.38 (1P, dtd, $J_{CH2-P\gamma}$ =21.7, $J_{P\beta-P\gamma}$ = 23.9, $J_{CH-P\gamma}$ = 6.5, Pγ), -10.85 (1H, br d, $J_{P\alpha-P\beta}$ = 19.2, Pα), -22.50 (1P, dd, $J_{P\alpha-P\beta}$ = 19.2, $J_{P\beta-P\gamma}$ = 23.9, Pβ); HRMS (-) ESI *m/z* found: 558.0184, calc. for $C_{14}H_{19}N_5O_{13}P_3^-$: 558.0192.

(12c) β-C-(2-ethynyl) 7-methylguanosine diphosphate ammonium salt

Obtained according to GP C starting from β-C-(2-ethynyl) guanosine diphosphate triethylammonium salt (**12a**) (10000 mOD, 0.828 mmol), $(CH_3)_2SO_4$ (0.785 mL, 8.278 mmol) and CH_3COOH solution pH 4 (8.0 mL). The ion-exchange purification afforded 4814 mOD (0.422 mmol, 51%) of **12c** triethylammonium salt. Additional HPLC purification of a fraction of obtained product gave **12c** as ammonium salt.

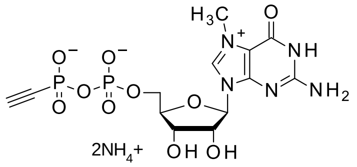

^{1}H NMR (400 MHz, D_2O, 25 °C) δ_H: 6.08 (1H, d, $J_{1'-2'}$ = 3.7, H1'), 4.70 (1H, dd, $J_{1'-2'}$ = 3.7, $J_{2'-3'}$ = 4.7, H2'), 4.51 (1H, dd, $J_{2'-3'}$ = 4.7, $J_{3'-4'}$ = 5.5, H3'), 4.41-4.44 (1H, m, H4'), 4.38 (1H, ddd, $J_{5'-5''}$ = 11.7, J = 4.3, 2.4, H5'), 4.24 (1H, ddd, $J_{5'-5''}$ = 11.7, J = 5.3, 2.2, H5''), 4. 13 (3H, s, m^7), 3.20 (1H, d, $J_{P\gamma-C2H}$ = 11.6, H_{C2H}); ^{31}P NMR (162 MHz, D_2O, 25°C) δ_P: -10.58 (1P, br d, $J_{P\alpha-P\beta}$ = 22.0, Pα),-20.86 (1P, dd, $J_{P\gamma-P\beta}$ = 22.0, $J_{P\gamma-C2H}$ = 11.6, Pβ); HRMS (-) ESI *m/z* found: 464.0378, calc. for $C_{13}H_{16}N_5O_{10}P_2^-$: 464.0372.

(12d) γ-C-(2-ethynyl) 7-methylguanosine triphosphate ammonium salt

Obtained according to GP B starting from γ-C-(2-ethynyl) guanosine triphosphate triethylammonium salt (**12b**) (10000 mOD, 0.828 mmol), $(CH_3)_2SO_4$ (0.785 mL, 8.278 mmol) and CH_3COOH solution pH 4 (8.0 mL). The ion-exchange purification afforded 5569 mOD (0.488 mmol, 59%) of **12d** triethylammonium salt. Additional HPLC purification of a fraction of obtained product gave **12d** as ammonium salt.

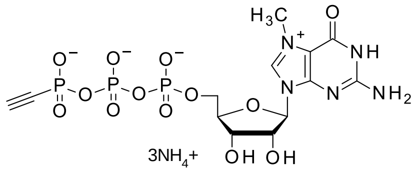

^{1}H NMR (400 MHz, D_2O, 25 °C) δ_H: 6.08 (1H, d, $J_{1'-2'}$ = 3.9, H1'), 4.70 (1H, dd, $J_{1'-2'}$ = 3.9, $J_{2'-3'}$ = 4.9, H2'), 4.54 (1H, dd, $J_{2'-3'}$ = 4.7, $J_{3'-4'}$ = 5.3, H3'), 4.41-4.44 (1H, m, H4'), 4.38 (1H, ddd, $J_{5'-5''}$ = 11.7, J = 4.1, 2.5, H5'), 4.24 (1H, ddd, $J_{5'-5''}$ = 11.7, J = 5.1, 2.0, H5''), 4. 14 (3H, s, m^7), 3.18 (1H, d, J = 12.9, H_{C2H}); ^{31}P NMR (162 MHz, D_2O, 25°C) δ_P: -10.80 (1P, br d, $J_{P\alpha-P\beta}$ = 20.5, Pα), -21.02 (1P, dd, $J_{P\gamma-P\beta}$ = 20.5, $J_{P\gamma-C2H}$ = 11.7, Pγ), -22.93 (1P, t, $J_{P\alpha-P\beta}$ = $J_{P\gamma-P\beta}$ = 20.5, Pβ); HRMS (-) ESI *m/z* found: 544.0042, calc. for $C_{13}H_{17}N_5O_{13}P_3^-$: 544.0036.

(12e) γ-C-(2-ethynyl) 2'-O-methylguanosine triphosphate triethylammonium salt

Analogue **12e** was obtained analogously to compounds **12a-b**. Triethylammonium 2-ethynyl C-phosphonate (**18c**) (1.698 mmol, 0.2 M) was stirred in 8.5 mL DMF until complete dissolution. Then, 2'-O-methylguanosine 5'-diphosphate β-P-imidazolide trisodium salt (**19h**) (6842 mOD, 0.566 mmol) along with $MgCl_2$ (431 mg, 4.531 mmol) were added and the mixture was stirred for 1 h at room temperature. The reaction was

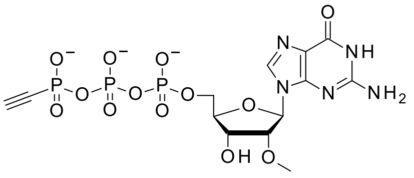

stopped by 10-fold dilution with water. The product was purified by ion-exchange chromatography on DEAE Sephadex A-25 and evaporated to dryness as described in General Information to afford 6385 mOD (0.528 mmol, 93%) of **12e** triethylammonium salt. Additional HPLC purification of a fraction of obtained product gave **12e** as ammonium salt.

^{1}H NMR (400 MHz, D_2O, 25 ^{o}C) δ_H: 8.13 (1H, s, H8), 5.99 (1H, d, $J_{1'-2'}$ = 6.5, H1'), 4.74 (1H, dd, $J_{2'-3'}$ = 5.1, $J_{3'-4'}$ = 3.1, H3'), 4.56 (1H, dd, $J_{2'-3'}$ = 5.1, $J_{1'-2'}$ = 6.5, H2'), 4.36-4.39 (1H, m, H4'), 4.28 (1H, ddd, $J_{5'-5''}$ = 11.7, $J_{P\alpha-5'}$ = 5.9, $J_{4'-5'}$ = 3.5, H5'), 4.24 (1H, ddd, $J_{5'-5''}$ = 11.7, $J_{4'-5''}$ = 5.1, $J_{P\alpha-5''}$ = 3.4, H5''), 3.47 (3H, s, $m^{2'O}$), 3.18 (1H, d, $J_{C2H-P\gamma}$ = 13.1, H_{C2H}); ^{31}P NMR (162 MHz, D_2O, 25°C) δ_P: -10.80 (1P, ddd, $J_{P\alpha-P\beta}$ = 20.5, $J_{P\alpha-5'}$ = 5.9, $J_{P\alpha-5''}$ = 3.4, Pα), -21.02 (1P, dd, $J_{C2H-P\gamma}$ = 13.1, $J_{P\beta-P\gamma}$ = 19.1, Pγ), -22.99 (1P, dd, $J_{P\beta-P\gamma}$ = 10.1, $J_{P\alpha-P\beta}$ = 20.5, Pβ); HRMS (-) ESI *m/z* found: 544.0044, calc. for $C_{13}H_{17}N_5O_{13}P_3^-$: 544.0036.

(12f) γ-C-(2-ethynyl) 2'-O-N7-dimethylguanosine triphosphate ammonium salt

Obtained according to GP C starting from β-C-(2-ethynyl) 2'-O'methylguanosine diphosphate triethylammonium salt (**12e**) (5380 mOD, 0.445 mmol), $(CH_3)SO_4$ (0.422 mL, 4.454 mmol) and CH_3COOH solution pH 4 (5.0 mL). The ion-exchange purification afforded 4014 mOD (0.352 mmol, 79%) of **12f** triethylammonium salt. Additional HPLC purification of a fraction of obtained product gave **12f** as ammonium salt.

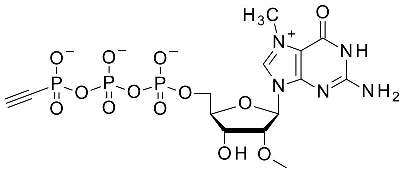

^{1}H NMR (400 MHz, D_2O, 25 ^{o}C) δ_H: 6.16 (1H, d, $J_{1'-2'}$ = 3.1, H1'), 4.65 (1H, dd, J = 5.1, 5.5, H3'), 4.36-4.42 (3H, overlapped H2', H4' and H5'), 4.26 (1H, ddd, $J_{5'-5''}$ = 12.5, $J_{P\alpha-5''}$ = 4.8, $J_{4'-5''}$ = 2.7, H5''), 4.14 (3H, s, m^7), 3.61 (3H, s, $m^{2'O}$), 3.18 (1H, d, $J_{P\gamma-C2H}$ = 12.9, H_{C2H}); ^{31}P NMR (162 MHz, D_2O, 25°C) δ_P: -10.80 (1P, ddd, $J_{P\alpha-P\beta}$ = 20.5, $J_{P\alpha-5'}$ = 7.3, $J_{P\alpha-5''}$ = 4.8, Pα), -21.05 (1P, dd, $J_{P\beta-P\gamma}$ = 19.1, $J_{P\gamma-C2H}$ = 12.9, Pγ), -22.91 (1P, dd, $J_{P\beta-P\gamma}$ = 19.1, $J_{P\alpha-P\beta}$ = 20.5, Pβ); HRMS (-) ESI *m/z* found: 558.0200, calc. for $C_{14}H_{19}N_5O_{13}P_3^-$: 558.0192.

1.1 Synthesis of phosphoramidate nucleotide analogues

General procedure D (GP D): Coupling of nucleotide imidazolides with amine linker

Analogues **15a-d** were synthesized analogously as described in Guranowski et al. for the reaction of diamine linkers with guanosine 5'-phosphorimidazolide.[7] An appropriate nucleotide imidazolide (**19a-f**) was dissolved in 0.1 M Tris-HCl buffer pH 8.0 (approx. 1 mL per 100 mg nucleotide) and propargylamine or 2-azidoethyloamine (8 equiv.) was added. The mixture was stirred at room temperature for 24 h. The reaction was diluted with ten volumes of water and extracted with diethyl ether. After setting pH to 7 with 5% HCl, the mixture was either subjected to ion-exchange chromatography purification as described in General Information to afford the desired product as triethylammonium salt or directly purified by semi-preparative HPLC to afford the desired product as ammonium salt.

1.1.1 Synthesis of alkyne-modified phosphoramidate nucleotide analogues

(15a) N-(2-propargyl) β-phosphoramidate guanosine diphosphate ammonium salt

Obtained according to GP D starting from guanosine 5'-diphosphate β-P-imidazolide disodium salt (**19b**) (195 mg, 3510 mOD, 0.290 mmol), propargylamine (0.149 mL, 2.324 mmol) and 2.0 mL of Tris-HCl buffer. The ion-exchange purification afforded 3223 mOD (0.267 mmol, 92%) of **15a** triethylammonium salt. Additional HPLC purification of a fraction of obtained product gave **15a** as ammonium salt.

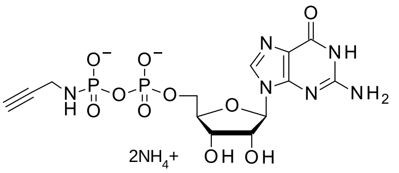

^{1}H NMR (400 MHz, D_2O, 25 ^{o}C) δ_H: 8.11 (1H, s, H8), 5.93 (1H, d, $J_{1'-2'}$ = 6.2, H1'), 4.82 (1h, overlapped with HDO, dd, $J_{1'-2'}$ = 6.2, $J_{2'-3'}$ = 5.0, H2'), 4.54 (1H, dd, $J_{2'-3'}$ = 5.0, $J_{3'-4'}$ = 3.7, H3'), 4.33-4. (1H, m, H4'), 4.19-4.21 (2H, m, H5'and H5''), 3.61 (2H, dd, $J_{CH2-P\beta}$ = 10.6, J_{CH2-CH} = 2.3, H_{CH2}), 2.51 (1H, t, J_{CH2-CH}= 2.3, H_{CH}); ^{31}P NMR (162 MHz, D_2O, 25°C) δ_P: -2.11 (1P, dt, $J_{P\alpha-P\beta}$ =22.1, $J_{CH2-P\beta}$ = 10.6, Pβ), -10.44 (1P, br d, $J_{P\alpha-P\beta}$ =22.1, Pα); HRMS (-) ESI *m/z* found: 479.0474, calc. for $C_{13}H_{17}N_6O_{10}P_2^-$: 479.0481.

(15b) N-(2-propargyl) γ-phosphoramidate guanosine triphosphate ammonium salt

Obtained according to GP D starting from guanosine 5'-triphosphate γ-P-imidazolide trisodium salt (**19c**) (400 mg, 7000 mOD, 0.579 mmol), propargylamine (0.297 mL, 4.636 mmol) and 4.0 mL of Tris-HCl buffer. The ion-exchange purification afforded 4546 mOD (0.376 mmol, 65%) of **15b** triethylammonium salt. Additional HPLC purification of a fraction of obtained product gave **15b** as ammonium salt.

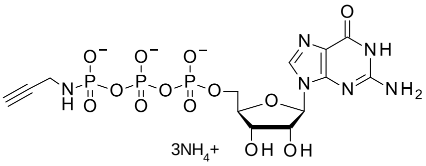

^{1}H NMR (400 MHz, D_2O, 25 ^{o}C) δ_H: 8.12 (1H, s, H8), 5.93 (1H, d, $J_{1'-2'}$ = 6.3, H1'), 4.83 (1H, dd, $J_{1'-2'}$ = 6.3, $J_{2'-3'}$ = 5.5, H2'), 4.56 (1H, dd, $J_{2'-3'}$ = 5.5, $J_{3'-4'}$ = 3.5, H3'), 4.35-4.38 (1H, m, H4'), 4.23-4.36 (2H, m, H5' and H5"), 3.66 (2H, dd, $J_{P\gamma-CH2}$ = 10.2, J_{CH2-CH} = 2.4, H_{CH2}), 2.51 (1H, t, J_{CH2-CH} = 2.4, H_{CH}); ^{31}P NMR (162 MHz, D_2O, 25°C) δ_P: -1.92 (1P, dt, $J_{P\gamma-P\beta}$ = 20.5, $J_{P\gamma-CH2}$ = 10.2, Pγ), -10.58 (1P, dt, $J_{P\alpha-P\beta}$ = 19.1, $J_{P\alpha-5'/5''}$ = 5.9, Pα), -22.06 (1P, dd, $J_{P\gamma-P\beta}$ = 20.5, $J_{P\alpha-P\beta}$ = 19.1, Pβ); HRMS (-) ESI *m/z* found: 559.0152, calc. for $C_{13}H_{18}N_6O_{13}P_3^-$: 559.0145; hydrolysis in D_2O: 8%

(15c) N-(2-propargyl) β-phosphoramidate 7-methylguanosine diphosphate ammonium salt

Obtained according to GP D starting from 7-methyl guanosine 5'-diphosphate β-P-imidazolide disodium salt (**19e**) (200 mg, 3060 mOD, 0.268 mmol), propargylamine (0.138 mL, 2.147 mmol) and 2.0 mL of Tris-HCl buffer. The ion-exchange purification afforded 2077 mOD (0.182 mmol, 68%) of **15c** triethylammonium salt. Additional HPLC purification of a fraction of obtained product gave **15c** as ammonium salt.

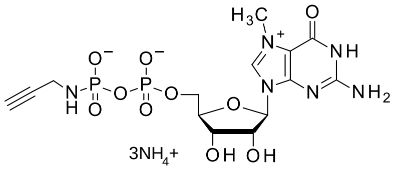

^{1}H NMR (400 MHz, D_2O, 25 °C) δ_H: 6.07 (1H, d, $J_{1'\text{-}2'}$ = 3.5, H1'), 4.68 (1H, dd, $J_{1'\text{-}2'}$ = 3.5, $J_{2'\text{-}3'}$ = 4.7, H2'), 4.51 (1H, dd, $J_{2'\text{-}3'}$ = 4.7, $J_{3'\text{-}4'}$ = 5.5, H3'), 4.42-4.44 (1H, m, H4'), 4.32-4.37 (1H, m, H5'), 4.22 (1H, ddd, $J_{5'\text{-}5''}$ = 12.0, $J_{5''\text{-}P\alpha}$ =5.1, $J_{4'\text{-}5''}$ = 1.6, H5''), 4.13 (1H, s, m^7), 3.68 (2H, dd, $J_{CH2\text{-}P\beta}$ = 11.0, $J_{CH2\text{-}CH}$ = 2.0, H_{CH2}), 2.57 (1H, t, $J_{CH2\text{-}CH}$ = 2.0, H_{CH}); ^{31}P NMR (162 MHz, D_2O, 25°C) δ_P: -2.37 (1P, dt, $J_{P\alpha\text{-}P\beta}$ =22.1, $J_{CH2\text{-}P\beta}$ = 11.0, Pβ), -11.0 (1P, d, $J_{P\alpha\text{-}P\beta}$ =22.1, Pα); HRMS (-) ESI *m/z* found: 493.0642, calc. for $C_{14}H_{19}N_6O_{10}P_2^-$: 493.0638.

(15d) N-(2-propargyl) γ-phosphoramidate 7-methylguanosine triphosphate ammonium salt

Obtained according to GP D starting from 7-methylguanosine 5'-triphosphate γ-P-imidazolide trisodium salt (**19f**) (398 mg, 5851 mOD, 0.513 mmol), propargylamine 0.263 mL, 4.106 mmol) and 4.0 mL of Tris-HCl buffer. The ion-exchange purification afforded 4083 mOD (0.358 mmol, 70%) of **15d** triethylammonium salt. Additional HPLC purification of a fraction of obtained product gave **15d** as ammonium salt.

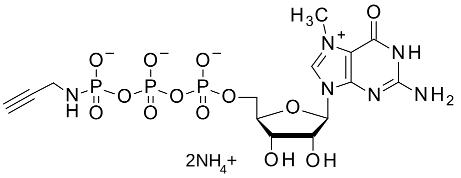

^{1}H NMR (400 MHz, D_2O, 25 °C) δ_H: 6.08 (1H, d, $J_{1'\text{-}2'}$ = 3.5, H1'), 4.70 (1H, dd, $J_{1'\text{-}2'}$ = 3.5, $J_{2'\text{-}3'}$ = 4.7, H2'), 4.55 (1H, dd, $J_{2'\text{-}3'}$ = 4.7, $J_{3'\text{-}4'}$ = 5.5, H3'), 4.40-4.43 (1H, m, H4'), 4.38 (1H, ddd, $J_{5'\text{-}5''}$ = 11.7, J = 3.9, 2.4, H5'), 4.26 (1H, ddd, $J_{5'\text{-}5''}$ = 11.7, J = 5.3, 2.7, H5''), 4.14 (3H, s, m^7), 3.70 (2H, dd, $J_{CH2\text{-}P\gamma}$ = 10.2, $J_{CH2\text{-}CH}$ = 2.7, H_{CH2}), 2.56 (1H, t, $J_{CH2\text{-}CH}$ = 2.7, H_{CH}); ^{31}P NMR (162 MHz, D_2O, 25°C) δ_P: 1.94 (1P, dt, $J_{CH2\text{-}P\gamma}$ = 10.2, $J_{P\beta\text{-}P\gamma}$ = 20.5, Pγ), -10.66 (1P, dt, $J_{P\alpha\text{-}P\beta}$ = 20.5, Pα), -22.02 (1P, dd, $J_{P\alpha\text{-}P\beta}$ = 20.5, $J_{P\beta\text{-}P\gamma}$ = 20.5, Pβ); HRMS (-) ESI *m/z* found: 573.03102, calc. for $C_{14}H_{20}N_6O_{13}P_3^-$: 573.0301.

1.1.2 Synthesis of azide-modified phosphoramidate nucleotide analogues

(17a) N-(2-azidoethyl) phosphoramidate guanosine monophosphate ammonium salt

Obtained according to GP D starting from guanosine 5'-monophosphate P-imidazolide sodium salt (**19a**) (200 mg, 4800 mOD, 0.397 mmol), 2-azidoethylamine (0.349 mL, 3.179 mmol) and 2.0 mL of Tris-HCl buffer. The ion-exchange purification afforded 4004 mOD (0.331 mmol, 83%) of **17a** triethylammonium salt. Additional HPLC purification of a fraction of obtained product gave **17a** as ammonium salt.

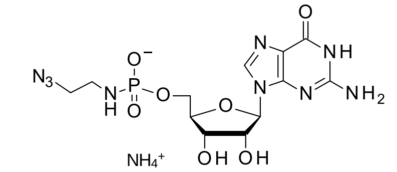

^{1}H NMR (400 MHz, D_2O, 25 °C) δ_H: 8.11 (1H, s, H8), 5.93 (1H, d, $J_{1'\text{-}2'}$ = 5.9, H1'), 4.84 (1H, dd, $J_{1'\text{-}2'}$ = 5.9, $J_{2'\text{-}3'}$ = 5.1, H2'), 4.51 (1H, dd, $J_{2'\text{-}3'}$ = 5.1, $J_{3'\text{-}4'}$ = 3.9, H3'), 4.31-4.34 (1H, m, H4'), 4.04 (1H, ddd, $J_{5'\text{-}5''}$ = 11.7, J = 4.7, 3.1, H5'), 4.00 (1H, ddd, $J_{5'\text{-}5''}$ = 11.7, J = 5.1, 3.5), 3.24 (2H, br t, $J_{CH2(N3)\text{-}CH2(NH)}$ = 5.9, $H_{CH2(N3)}$), 2.89 (2H, dt, $J_{CH2(N3)\text{-}CH2(NH)}$ = 5.9, $J_{CH2(N3)\text{-}P\alpha}$ = 10.2, $H_{CH2(NH)}$); ^{31}P NMR (162 MHz, D_2O, 25°C) δ_P: 9.37-9.55 (1P, m, Pα); HRMS (-) ESI *m/z* found: 430.0991, calc. for $C_{12}H_{17}N_9O_7P^-$: 430.0989.

(17b) N-(2-azidoethyl) β-phosphoramidate guanosine diphosphate ammonium salt

Obtained according to GP D starting from guanosine 5'-diphosphate β-P-imidazolide disodium salt (**19b**) (458 mg, 8244 mOD, 0.682 mmol), 2-azidoethylamine (0.600 mL, 5.460 mmol) and 4.5 mL of Tris-HCl buffer. The ion-exchange purification afforded 7085 mOD (0.586 mmol, 86%) of **17b** triethylammonium salt. Additional HPLC purification of a fraction of obtained product gave **17b** as ammonium salt.

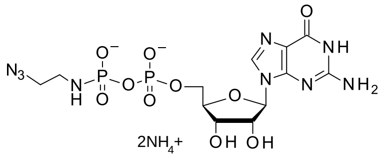

^{1}H NMR (400 MHz, D_2O, 25 °C) δ_H: 8.12 (1H, s, H8), 5.93 (1H, d, $J_{1'\text{-}2'}$ = 6.3, H1'), 4.80 (1H, overlapped with HDO, H2'), 4.53 (1H, dd, J = 3.7, 5.3, H3'), 4.33-4.36 (1H, m, H4'), 4.18-4.21 (1H, m, H5' and H5''), 3.32 (2H, br t, $J_{CH2(N3)\text{-}CH2(NH)}$ = 5.9, $H_{CH2(N3)}$), 3.03 (2H, dt, $J_{CH2(N3)\text{-}CH2(NH)}$ = 5.9, $J_{CH2(N3)\text{-}P\beta}$ = 10.4, $H_{CH2\text{-}NH}$); ^{31}P NMR (162 MHz, D_2O, 25°C) δ_P: -1.04 (1P, dt, $J_{CH2(N3)\text{-}P\beta}$ = 10.4, $J_{P\alpha\text{-}P\beta}$ = 22.0, Pβ), -10.26 (1P, br d, $J_{P\alpha\text{-}P\beta}$ = 22.0, Pα); HRMS (-) ESI *m/z* found: 510.0657, calc. for $C_{12}H_{18}N_9O_{10}P_2^-$: 510.0652; hydrolysis in D_2O: 15%.

(17c) N-(2-azidoethyl) phosphoramidate 7-methylguanosine monophosphate ammonium salt

Obtained according to GP D starting from 7-methyl guanosine 5'-monophosphate P-imidazolide sodium salt (**19d**) (450 mg, 9900 mOD, 0.868 mmol), 2-azidoethylamine (0.764 mL, 6.944 mmol) and 4.5 mL of Tris-HCl buffer. The directly following HPLC purification afforded 3520 mOD of **17c** (0.309 mmol, 36%) as ammonium salt.

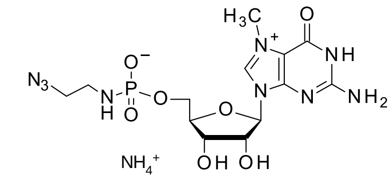

^{1}H NMR (400 MHz, D_2O, 25 °C) δ_H: 6.08 (1H, d, $J_{1'\text{-}2'}$ = 3.9, H1'), 4.68 (1H, dd, $J_{1'\text{-}2'}$ = 3.9, $J_{2'\text{-}3'}$ =

4.9, H2’), 4.48 (1H, dd, $J_{2'-3'}$ = 4.9, $J_{3'-4'}$ = 5.4, H3’), 4.38-4.41 (1H, m, H4’), 4.18 (1H, ddd, $J_{5'-5''}$ = 11.7, J = 4.4, 2.4, H5’), 4. 12 (3H, s, m^7), 4.06 (1H, ddd, $J_{5'-5''}$ = 11.7, J = 4.9, 2.9, H5’’), 3.35 (2H, t, $J_{CH2(N3)-CH2(NH)}$ = 5.9, $H_{CH2(N3)}$), 3.0 (2H, dt, $J_{CH2(N3)-CH2(NH)}$ = 5.9, $J_{P\alpha-CH2(NH)}$ = 10.3, $H_{CH2(NH)}$); ^{31}P NMR (162 MHz, D_2O, 25°C) δ_P: 9.32-9.44 (1P, m, Pα); HRMS (-) ESI *m/z* found: 444.1146, calc. for $C_{13}H_{19}N_9O_7P^-$: 444.1145.

(17d) N-(2-azidoethyl) β-phosphoramidate 7-methylguanosine diphosphate ammonium salt

Obtained according to GP D starting from 7-methylguanosine 5’-diphosphate β-P-imidazolide disodium salt (**19e**) (238 mg, 3641 mOD, 0.319 mmol), 2-azidoethylamine (0.281 mL, 2.555 mmol) and 2.5mL of Tris-HCl buffer. The ion-exchange purification afforded 1785 mOD (0.156 mmol, 49%) of **17d** triethylammonium salt. Additional HPLC purification of a fraction of obtained product gave **17d** as ammonium salt.

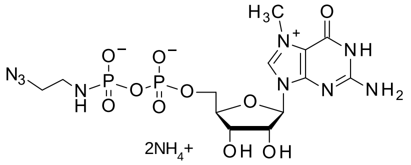

^{1}H NMR (400 MHz, D_2O, 25 °C) δ_H: 6.08 (1H, d, $J_{1'-2'}$ = 3.5, H1’), 4.68 (1H, dd, $J_{1'-2'}$ = 3.5, $J_{2'-3'}$ = 4.7, H2’), 4.51 (1H, $J_{2'-3'}$ = 4.7, $J_{3'-4'}$ = 5.5, H3’), 4.39-4.42 (1H, m, H4’), 4.34 (1H, ddd, $J_{5'-5''}$ = 12.1, J = 4.3, 2.4, H5’), 4.22 (1H, ddd, $J_{5'-5''}$ = 12.1, J = 5.5, 2.4, H5’’), 4.13 (3H, s, m^7), 3.41 (2H, t, , $J_{CH2(N3)-CH2(NH)}$ = 5.9, $H_{CH2(N3)}$), 3.10 (2H, dt, , $J_{CH2(N3)-CH2(NH)}$ = 5.9, $J_{CH2(NH)-P\beta}$ = 10.5, $H_{CH2(NH)}$); ^{31}P NMR (162 MHz, D_2O, 25°C) δ_P: -0.82 (1P, dt, $J_{CH2-P\beta}$ = 10.5, $J_{P\alpha-P\beta}$ = 22.7, Pβ), -10.28 (1P, br d, $J_{P\alpha-P\beta}$ = 22.7, Pα); HRMS (-) ESI *m/z* found: 524.0819, calc. for $C_{13}H_{20}N_9O_{10}P_2^-$: 524.0808.

1.2 Synthesis of phosphothioester nucleotide analogues

Synthesis of thiophosphate nucleotide analogues (20a-d)

Analogues containing thiophosphate moiety at the terminal position of the phosphate chain (**20a-d**) were synthesized as described previously.[8] To a suspension of appropriate nucleotide imidazolide derivative (1 equiv.) and thiophosphate triethylammonium salt in DMF anhydrous $ZnCl_2$ was added (8 equiv.). The resulting solution was stirred for 20 min at room temperature. The reaction was quenched by 10-fold dilution with water and addition of EDTA (8 eq.) and $NaHCO_3$ (ca. 17.6 equiv.). The ion-exchange purification afforded triethylammonium salt of GDPβS (**20a**), GTPγS (**20b**), m^7GDPβS (**20c**) and m^7GTPγS (**20d**). Yields: 80-95%.

General procedure E (GP E): S-alkylation of thiophosphate nucleotide analogues

An appropriate nucleotide (**20a-d**) was dissolved in DMSO to a concentration of ca. 0.1 M and propargyl bromide (1 equiv.) was added. The mixture was stirred at room temperature for approx. 15 min. Then the reaction was diluted with ten volumes of water and extracted with diethyl ether. After setting pH to 7 (if needed), the mixture was subjected to ion-exchange chromatography purification as described in General Information to afford the desired product as triethylammonium salt.

(14a) S-(2-propargyl) β-phosphothioester guanosine diphosphate diammonium salt

Obtained according to GP E starting from guanosine 5’-(β-thiodiphosphate) triethylammonium salt (**20a**) (3383 mOD, 0.280 mmol), propargyl bromide (21 μL) and DMSO (2.8 mL). The ion-exchange purification afforded 1819 mOD (0.150 mmol, 54%) of **14a** triethylammonium salt. Additional HPLC purification of a fraction of obtained product gave **14a** as diammonium salt.

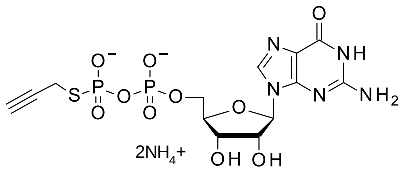

^{1}H NMR (400 MHz, D_2O, 25 °C) δ_H: .12 (1H, s, H8), 5.92 (1H, d, $J_{1'-2'}$ = 6.3, H1’), 4.82 (1H, dd, $J_{1'-2'}$ = 6.3, $J_{2'-3'}$ = 5.3, H2’), 4.54 (1H, dd, $J_{2'-3'}$ = 5.3, $J_{3'-4'}$ = 3.3, H3’), 4.35 (1H, qd, $J_{3'-4'}$ = 3.3, $J_{4'-5'}$ = 2.1, H4’), 4.20-4.25 (2H, m, H5’ and H5’’), 3.53 (2H, dd, $J_{CH2-P\beta}$ = 12.5, J_{CH2-CH} = 2.7, $H_{CH2'}$), 2.55 (1H, dd, J_{CH2-CH} = 2.7, H_{CH}); ^{31}P NMR (162 MHz, D_2O, 25°C) δ_P: 7.43 (1P, ddd, $J_{P\beta-P\alpha}$ = 28.6, $J_{CH2-P\beta}$ = 12.5, Pβ), -11.10 (1P, dt, $J_{P\beta-P\alpha}$ = 28.6, $J_{5'/5''-P\alpha}$ = 5.4, Pα); HRMS (-) ESI *m/z* found: 496.0084, calc. for $C_{13}H_{16}N_5O_{10}P_2S^-$: 496.0099.

(14b) S-(2-propargyl) γ-phosphothioester guanosine triphosphate ammonium salt

Obtained according to GP E starting from guanosine 5’-(γ-thiotriphosphate) triethylammonium salt (**20b**) (2998 mOD, 0.248 mmol), propargyl bromide (19 μL) and DMSO (2.5 mL). The ion-exchange purification afforded 2218 mOD (0.184 mmol, 74%) of **14b** triethylammonium salt. Additional HPLC purification of a fraction of obtained product gave **14b** as ammonium salt.

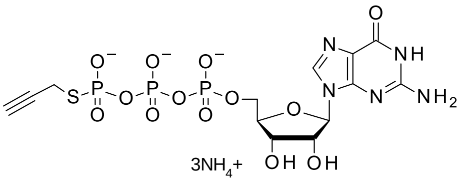

^{1}H NMR (400 MHz, D_2O, 25 °C) δ_H: 8.11 (1H, s, H8), 5.91 (1H, d, $J_{1'-2'}$ = 6.3, H1’), 4.79 (1H, overlapped with HDO, H2’), 4.55 (1H, dd, $J_{2'-3'}$ = 5.0, $J_{3'-4'}$ = 3.5, H3’), 4.35 (1H, qd, $J_{3'-4'}$ = 3.5, $J_{4'-5'}$ = $J_{4'-5''}$ = 1.9, H4’), 4.25 (2H, m, H5’ and H5’’), 3.57 (2H, dd, $J_{CH2-P\gamma}$ = 12.5, J_{CH2-CH} = 2.7, H_{CH2}), 2.54 (1H, dd, J_{CH2-CH} = 2.7, H_{CH}); ^{31}P NMR (162 MHz, D_2O, 25°C) δ_P: 9.69 (1P, ddd, $J_{P\gamma-P\beta}$ = 25.6, $J_{CH2-P\gamma}$ = 12.5, Pγ), -8.51 (1P, dt, $J_{P\beta-P\alpha}$ = 19.0, $J_{5/5''-P\alpha}$ = 5.2, Pα), -20.82 (1P, dd, $J_{P\gamma-P\beta}$ = 25.6, $J_{P\beta-P\alpha}$ = 19.0, Pβ); HRMS (-) ESI *m/z* found: 575.9755, calc. for $C_{13}H_{17}N_5O_{13}P_3S^-$: 575.9762.

(14c) S-(2-propargyl) β-phosphothioester 7-methylguanosine diphosphate ammonium salt

Obtained according to GP E starting from 7-methylguanosine 5’-(β-thiodiphosphate) triethylammonium salt (**20c**) (1700 mOD, 0.149 mmol), propargyl bromide (11 μL) and

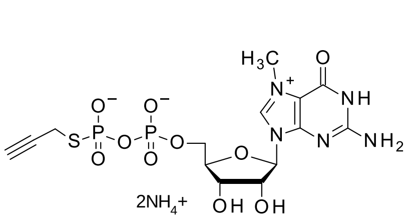

DMSO (1.5 mL). The ion-exchange purification afforded 1125 mOD (0.099 mmol, 66%) of **14c** triethylammonium salt. Additional HPLC purification of a fraction of obtained product gave **14c** as ammonium salt.

^{1}H NMR (400 MHz, D_2O, 25 °C) δ_H: 6.07 (1H, d, $J_{1'-2'}$ = 3.9, H1'), 4.70 (1H, dd, $J_{2'-3'}$ = 5.2, $J_{1'-2'}$ = 3.9, H2'), 4.51 (1H, dd, $J_{2'-3'} = J_{3'-4'}$ = 5.2, H3'), 4.41 (1H, dq, $J_{3'-4'}$ = 5.2, $J_{4'-5'} = J_{4'-5''}$ = 2.5, H4'), 4.35 (1H, ddd, $J_{5'-5''}$ = 11.9, J = 4.2, 2.5, H5'), 4.22 (1H, ddd, $J_{5'-5''}$ = 11.9, J = 5.1, 2.5, H5"), 4.13 (3H, s, m^7), 3.58 (2H, dd, $J_{CH2-P\beta}$ = 12.5, J_{CH2-CH} = 2.7, H_{CH2}), 2.59 (1H, t, J_{CH2-CH} = 2.7, H_{CH}); ^{31}P NMR (162 MHz, D_2O, 25°C) δ_P: 7.43 (1P, ddd, $J_{P\beta-P\alpha}$ = 28.6, $J_{CH2-P\beta}$ = 12.5, Pβ), -11.23 (1P, dq, $J_{P\beta-P\alpha}$ = 28.6, $J_{5'/5''-P\alpha}$ = 4.2, Pα); HRMS (-) ESI *m/z* found: 510.0257, calc. for $C_{14}H_{18}N_5O_{10}P_2S^-$: 510.0255.

(14d) S-(2-propargyl) γ-phosphothioester 7-methylguanosine triphosphate ammonium salt

Obtained according to GP E starting from 7-methylguanosine 5'-(γ-thiotriphosphate) triethylammonium salt (**20d**) (2246 mOD, 0.197 mmol), propargyl bromide (15 µL) and DMSO (2.0 mL). The ion-exchange purification afforded 1669 mOD (0.146 mmol, 74%) of **14d** triethylammonium salt. Additional HPLC purification of a fraction of obtained product gave **14d** as ammonium salt.

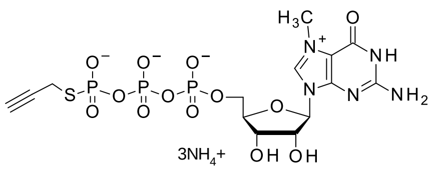

^{1}H NMR (400 MHz, D_2O, 25 °C) δ_H: 6.07 (1H, d, $J_{1'-2'}$ = 3.5, H1'), 4.70 (1H, dd, $J_{2'-3'}$ = 4.9, $J_{1'-2'}$ = 3.5, H2'), 4.54 (1H, dd, $J_{2'-3'}$ = 4.9, $J_{3'-4'}$ = 5.3, H3'), 4.35-4.43 (2H, overlapped H4', H5'), 4.26 (1H, ddd, $J_{5'-5''}$ = 11.9, J = 5.6, 2.3, H5"), 4.13 (3H, s), 3.60 (2H, dd, $J_{CH2-P\gamma}$ =12.1, J_{CH2-CH} = 2.5, H_{CH2}), 2.58 (1H, t, J_{CH2-CH} = 2.5, H_{CH}); ^{31}P NMR (162 MHz, D_2O, 25°C) δ_P: 9.40 (1P, dt, $J_{P\gamma-P\beta}$ = 26.5, $J_{CH2-P\gamma}$ = 12.1, Pγ), -8.57 (1P, dq, $J_{P\beta-P\alpha}$ = 20.0, $J_{5''-P\alpha}$ = 2.3, $J_{5'-P\alpha}$ = 1.8, Pα), -20.83 (1P, dd, $J_{P\gamma-P\beta}$ = 26.5, $J_{P\beta-P\alpha}$ = 20.0, Pβ); HRMS (-) ESI *m/z* found: 589.9920, calc. for $C_{14}H_{19}N_5O_{13}P_3S^-$: 589.9918.

1.3 Synthesis of azide-modified guanosine analogues

(16a) 5'-azido-5'deoxyguanosine

Analogue **16a** was obtained according to Lee et al with minor modifications.[9] Guanosine (1.43 g, 5.05 mmol), imidazole (2.25 g, 33.05 mmol), triphenylphosphine (4.32 g, 16.47 mmol) were vigorously stirred in DMP (18 mL). After complete dissolution, iodine (4.02 g, 15.84 mmol) solution in DMP (2 mL) was gradually added to the mixture. Stirring was continued at room temperature for 3 h after which 200 mL of CH_2Cl_2 and 60 mL of water were added and the mixture stored at 4 °C overnight. Next, resulting white precipitate was filtered and dried in vacuum over P_2O_5 to afford 5'-iodo-5'deoxyguanosine (**21**) (1.59 g, 4.04 mmol, 80%) which was directy used for next step of the synthesis.

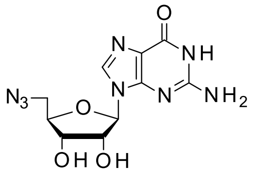

5'-iodo-5'deoxyguanosine (**21**) (1.59 g, 4.04 mmol) and NaN_3 (1.04 g, 16.00 mmol) were stired in DMF (18 mL) at 70 °C for 24 h. Afetrwards, the mixture was concentrated to approx. 1 mL by evaporation of DMF and 20 mL of water was added. The suspension was dissolved after heating to approx. 50°C and then stored at 4 °C overnight. Resulting white precipitate was filtered off, washed with water (5 mL), cold ethanol (5 mL) and diethyl ether (2 mL) and then dried in vacuum over P_2O_5 to afford **16a** (711 mg, 2.31 mmol, 57%).

^{1}H NMR (500 MHz, D_2O, 25 °C) δ_H: 10.76 (1H, br s, NH(1)), 7.96 (1H, s, H8), 6.58 (2H, br s, 2-NH_2), 5.78 (1H, d, $J_{1'-2'}$ = 5.8, H1'), 5.61 (1H, br s, 2'-OH or 3'-OH), 5.37 (1H, br s, 2'-OH or 3'-OH), 4.63 (1H, dd, $J_{1'-2'}$ = 5.8, $J_{2'-3'}$ = 5.2, H2'), 4.12 (1H, $J_{2'-3'}$ = 5.2, $J_{3'-4'}$ = 3.7, H3'), 4.04 (1H, ddd, $J_{3'-4'}$ = 3.7, $J_{5'-4'}$ = 7.0, $J_{5''-4'}$ = 4.0, H4'), 3.72 (1H, dd, $J_{5'-4'}$ = 7.0, H5'), 3.58 (1H, dd, $J_{5''-4'}$ = 4.0, H5''); HRMS (-) ESI *m/z* found: 307.0910, calc. for $C_{10}H_{11}N_8O_4^-$: 307.0903.

(16b) 5'-azido-5'deoxy-7-methylguanosine

5'-azido-5'deoxyguanosine (**16a**) (350 mg, 1.14 mmol) was dissolved in DMF (4 mL) and CH_3I (566 µl, 9.09 mmol) was added. The mixture was stirred at room temperature for 3h (until 95% conversion into the desired product as determined by HPLC). The excess CH_3I was removed under reduced pressure and the solution was concentrated to 0.5 mL. The resutling DMSO solution of **16b** at 2.3 M concentration was stored at -20 °C and used for click reactions without any further treatment.

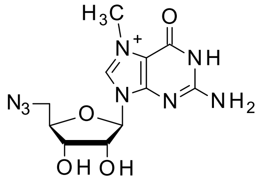

^{1}H NMR (500 MHz, D_2O, 25 °C) δ_H: 6.02 (1H, d, $J_{1'-2'}$ = 3.8, H1'), 4.78 (1H, dd, $J_{1'-2'}$ = 3.8, $J_{2'-3'}$ = 5.2, H2'), 4.42 (1H, dd, $J_{2'-3'}$ = 5.2, $J_{3'-4'}$ = 5.7, H3'), 4.34 (1H, ddd, $J_{3'-4'}$ = 5.7, $J_{4'-5'}$ = 3.2, $J_{4'-5''}$ = 5.0, H4'), 4.11 (3H, s, m^7), 3.84 (1H, dd, $J_{4'-5'}$ = 3.2, $J_{5'-5''}$ = 13.7, H5'), 3.84 (1H, dd, $J_{4'-5''}$ = 5.0, $J_{5'-5''}$ = 13.7, H5''); HRMS (+) ESI *m/z* found: 323.1210, calc. for $C_{11}H_{15}N_8O_4^+$: 323.1216.

1.4 Synthesis of dinucleotide cap analogues

General procedure F (GP F): Synthesis of dinucleotide cap analogues containing triazole directly attached to ribose

An aqueous solution of an alkyne-containing nucleotide (1 equiv., 0.2-1.0 M) and an azide-containing nucleoside in DMF (3 equiv., 0.6-3.0 M) were mixed together followed by addition of aqueous solutions of $CuSO_4{\cdot}5H_2O$ (0.2 equiv., 0.5-6.0 M) and sodium ascorbate (0.4 equiv., 1-12 M). The reaction was stirred at room temperature for several hours and monitored by RP HPLC. Additional portions of

$CuSO_4$ or sodium ascorbate solutions or solvents (DMF or H_2O) were added upon precipitation or slow kinetics. Final concentrations of reagents are given in the detailed procedures below. When completed, the reaction was quenched by 5-fold dilution with water and addition of Na_2EDTA (ten equivalents of added $CuSO_4$) followed by direct semi-preparative RP HPLC purification.

General procedure G (GP G): Synthesis of dinucleotide cap analogues containing triazole located between P-subunits
Aqueous solutions of an alkyne-containing nucleotide (1 equiv., 0.2-1.0 M) and an azide-containing nucleotide (1 equiv., 0.2-1.0 M) were mixed together followed by addition of H_2O (to the concentration of each analogue ca. 50-150 mM) and aqueous solutions of $CuSO_4 \cdot 5H_2O$ (0.2 equiv., 0.5-6.0 M) and sodium ascorbate (0.4 equiv., 1-12 M). The reaction was stirred at room temperature for several hours and monitored by RP HPLC. Additional portions of $CuSO_4$ or sodium ascorbate solutions were added upon slow kinetics. Final concentrations of reagents are given in the detailed procedures below. When completed, the reaction was quenched by 5-fold dilution with water and addition of Na_2EDTA (ten equivalents of added $CuSO_4$) directly followed by semi-preparative RP HPLC purification.

(1a) m^{7}G-triazole-C$_2$H$_4$ppG
Obtained according to GP F from **10a** (1812 mOD, 0.150 mmol, 335 mM) and **16b** (0.450 mmol, 1004 mM) while stirring with $CuSO_4 \cdot 5H_2O$ (15.0 mg, 0.060 mmol, 134 mM) and sodium ascorbate (23.8 mg, 0.120 mmol, 268 mM) in 0.448 mL of DMF/H_2O (2:1, v/v) for 8 h. After quenching the reaction with Na_2EDTA (223.5 mg, 0.60 mmol), the product was subjected to RP HPLC purification which afforded **1a** as ammonium salt.

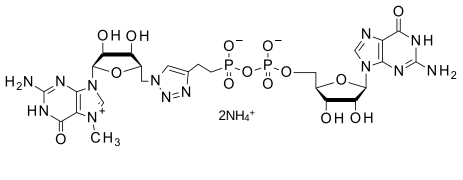

^{1}H NMR (400 MHz, D_2O, 25°C) δ_H: 8.88 (1H, s, H8 m^{7}G), 8.06 (1H, s, H8 G or H$_{triazole}$), 7.42 (1H, s, H8 G or H$_{triazole}$), 5.88 (1H, d, $J_{1'-2'}$ = 2.0, H1′ m^{7}G), 5.82 (1H, d, $J_{1'-2'}$ = 6.0, H1′ G), 4.70-4.73 (3H, overlapped H5′ and H5″ m^{7}G, H2′ G), 4.65 (1H, dd, $J_{1'-2'}$ = 2.0, $J_{2'-3'}$ = 5.1, H2′ m^{7}G), 4.49 (1H, dd, J = 4.0, 4.7, H3′ G), 4.35-4.43(1H, overlapped H3′ and H4′ m^{7}G), 4.31-4.32 (1H, m, H4′ G), 4.18-4.20 (2H, m, H5′and H5″ G), 4.10 (3H, s, m^7), 2.75-2.83 (2H, m, H$_{CH2(triazole)}$), 1.93-1.97 (2H, m, H$_{CH2(P)}$); ^{31}P NMR (162 MHz, D_2O, 25°C) δ_P: 17.05-17.54 17.28 (1P, m, Pβ), -10.84 (1P, br d, $J_{P\alpha-P\beta}$ = 26.9, Pα); HRMS (-) ESI *m/z* found: 800.1675, calc. for $C_{25}H_{32}N_{13}O_{14}P_2^-$: 800.1667.

(1b) m^{7}G-triazole-C$_2$H$_4$pppG(1b) m^{7}G-triazole-C$_2$H$_4$pppG
Obtained according to GP F from **10b** (1812 mOD, 0.150 mmol, 335 mM) and **16b** (0.450 mmol, 1004 mM) while stirring with $CuSO_4 \cdot 5H_2O$ (15.0 mg, 0.060 mmol, 134 mM) and sodium ascorbate (23.8 mg, 0.120 mmol, 268 mM) in 0.448 mL of DMF/H_2O (2:1, v/v) for 8 h. After quenching the reaction with Na_2EDTA (223.5 mg, 0.60 mmol), the product was subjected to RP HPLC purification which afforded **1b** as ammonium salt.

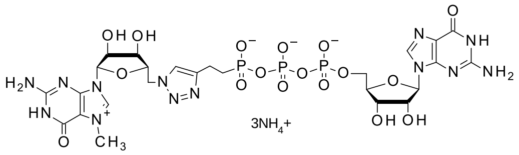

^{1}H NMR (400 MHz, D_2O, 25 ^{o}C) δ_H: 8.02 (1H, s, H8 G or H$_{triazole}$), 7.70 (1H, s, H8 G or H$_{triazole}$), 5.91 (1H, d, $J_{1'-2'}$ =2.0, H1′ m^{7}G), 5.82 (1H, d, $J_{1'-2'}$ = 5.7, H1′ G), 4.68-4.76 (4H, overlapped H5′ and H5″ m^{7}G, H2′ m^{7}G, H2′ G), 4.53 (1H, dd, J = 4.0, 5.0, H3′ G), 4.48 (1H, dd, J = 4.4, 7.8, H3′ m^{7}G), 4.39-4.42 (1H, m, H4′ m^{7}G), 4.30 (1H, m, H4′ G), 4.23-4.25 (2H, m, H5′ and H5″ G), 4.08 (3H, s, m^7), 2.83-2.89 (1H, m, H$_{CH2(triazole)}$), 1.99-2.09 (1H, m, H$_{CH2(P)}$); ^{31}P NMR (162 MHz, D_2O, 25°C) δ_P: 17.45-18.01 (1P, m, Pγ), -10.68 (1P, br d, $J_{P\alpha-P\beta}$ = 19.3, Pα), -22.40 (1P, dd, $J_{P\alpha-P\beta}$ = 19.3, $J_{P\gamma-P\beta}$ = 25.1, Pβ); HRMS (-) ESI *m/z* found: 880.1337, calc. for $C_{25}H_{33}N_{13}O_{17}P_3^-$: 880.1330.

(1c) m^{7}GppC$_2$H$_4$-triazole-G
Obtained according to GP F from **10c** (1710 mOD, 0.150 mmol, 335 mM) and **16a** (138.6 mg, 0.450 mmol, 1004 mM) while stirring with $CuSO_4 \cdot 5H_2O$ (15.0 mg, 0.060 mmol, 134 mM) and sodium ascorbate (23.8 mg, 0.120 mmol, 268 mM) in 0.448 mL of DMF/H_2O (2:1, v/v) for 3.5 h. After quenching the reaction with Na_2EDTA (223.5 mg, 0.60 mmol), the product was subjected to RP HPLC purification which afforded **1c** as ammonium salt.

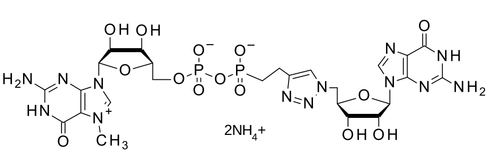

^{1}H NMR (400 MHz, D_2O, 25 ^{o}C) δ_H: 9.15 (1H, s, H8 m^{7}G), 7.66 (1H, s, H8 G or H$_{triazole}$), 7.62 (1H, s, H8 G or H$_{triazole}$), 6.00 (1H, d, J$_{1'-2'}$ = 3.7, H1′ m^{7}G or G), 5.84 (1H, d, J$_{1'-2'}$ = 3.0, H1′ m^{7}G or G), 4.75-4.76 (2H, m, H5′ and H5′′ G), 4.65-4.68 (2H, m, H2′ G and H2′ m^{7}G), 4.51 (1H, dd, J = 6.7, 5.2, H3′ G), 4.49 (1H, dd, J = 5.0, 5.2, H3′ m^{7}G), 4.41 (2H, dt, J = 7.0, 3.7, H4′ G), 4.36-4.38 (1H, m, H4′ m^{7}G), 4.31 (1H, ddd, $J_{5'-5''}$ = 12.0, J = 4.0, 2.5, H5′ m^{7}G), 4.20 (1H, ddd, $J_{5'-5''}$ = 12.0, J = 5.2, 2.2, H5′′ m^{7}G), 4.05 (3H, s, m^7), 2.77-2.85 (2H, m, H$_{CH2(triazole)}$), 1.95-2.05 (2H, m, H$_{CH2(P)}$); ^{31}P NMR (162 MHz, D_2O, 25°C) δ_P: 16.68-17.19 (1P, m, Pβ), -11.34 (1P, br d, $J_{P\beta-P\gamma}$ = 27.3, Pγ); HRMS (-) ESI *m/z* found: 800.1671, calc. for $C_{25}H_{32}N_{13}O_{14}P_2^-$: 800.1667.

(1d) m^{7}GpppC$_2$H$_4$-triazole-G
Obtained according to GP F from **10d** (1710 mOD, 0.150 mmol, 335 mM) and **16a** (138.6 mg, 0.450 mmol, 1004 mM) while stirring with $CuSO_4 \cdot 5H_2O$ (15.0 mg, 0.060 mmol, 134 mM) and sodium ascorbate (23.8 mg, 0.120 mmol, 268 mM) in 0.448

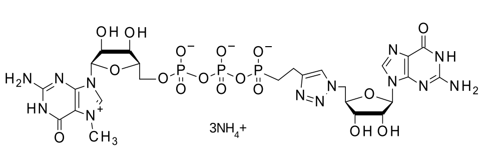

mL of DMF/H_2O (2:1, v/v) for 3.5 h. After quenching the reaction with Na_2EDTA (223.5 mg, 0.60 mmol), the product was subjected to RP HPLC purification which afforded **1d** as ammonium salt.

^{1}H NMR (400 MHz, D_2O, 25 °C) δ_H: 9.16 (1H, s, H8 m^{7}G), 7.69 (1H, s, H8 G or $H_{triazole}$), 7.67 (1H, s, H8 G or $H_{triazole}$), 5.98 (1H, d, $J_{1'-2'}$ = 3.2, H1′ m^{7}G or G), 5.82 (1H, d, $J_{1'-2'}$ = 2.2, H1′ m^{7}G or G), 4.75-4.76 (2H, m, H5′ and H5′′ G), 4.68-4.71 (2H, m, H2′ G and H2′ m^{7}G), 4.53-4.56 (2H, overlapped H3′ G and H3′ m^{7}G), 4.33-4.41 (3H, overlapped H4′ m^{7}G, H4′ G, H5′ m^{7}G), 4.24 (1H, ddd, H5′′ m^{7}G), 4.06 (3H, s, m^7), 2.80-2.86 (2H, m, $H_{CH2(triazole)}$), 1.98-2.09 (2H, m, $H_{CH2(P)}$); ^{31}P NMR (162 MHz, D_2O, 25°C) δ_P: 17.87-18.06 (1P, m, Pβ), - 10.69 (1P, d, $J_{P\gamma-P\delta}$ = 19.5, Pδ), - 22.32 (1P, dd, $J_{P\gamma-P\delta}$ = 19.5, $J_{P\gamma-P\beta}$ = 24.9, Pγ); HRMS (-) ESI *m/z* found: 880.1333, calc. for $C_{25}H_{33}N_{13}O_{17}P_3^-$: 880.1330.

(2a) m^{7}G-triazole-CH$_2$ppG

Obtained according to GP F from **11a** (2000 mOD, 0.166 mmol, 229 mM) and **16b** (0.497 mmol, 687 mM) while stirring with $CuSO_4{\cdot}5H_2O$ (24.8 mg, 0.099 mmol, 138 mM) and sodium ascorbate (39.4 mg, 0.199 mmol, 275 mM) in 0.722 mL of DMF/H_2O (2.2:1, v/v) for 6 h. After quenching the reaction with Na_2EDTA (368.8 mg, 0.99 mmol), the product was subjected to RP HPLC purification which afforded **2a** as ammonium salt.

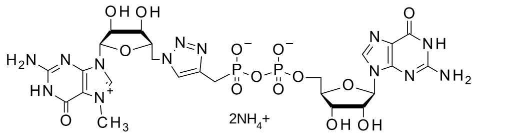

^{1}H NMR (400 MHz, D_2O, 25 °C) δ_H: 8.00 (1H, s, H8 G), 7.83 (1H, d, *J* = 2.4, $H_{triazole}$), 5.88 (1H, d, $J_{1'-2'}$ = 3.3, H1′ m^{7}G), 5.82 (1H, d, $J_{1'-2'}$ = 5.9, H1′ G), 4.79 (2H, overlapped with HDO, H5′ and H5″ m^{7}G), 4.69 (1H, dd, $J_{1'-2'}$ = 5.9, $J_{2'-3'}$ = 5.5, H2′ G), 4.52 (1H, dd, $J_{1'-2'}$ = 3.3, $J_{2'-3'}$ = 5.5, H2′ m^{7}G), 4.44-4.48 (2H, overlapped H3′ G, H4′ m^{7}G), 4.32 (1H, dd, $J_{2'-3'}$ = 5.5, $J_{3'-4'}$ = 8.8, H3′ m^{7}G), 4.25-4.28 (1H, m, H4′ G), 4.09-4.12 (2H, m, H5′ and H5′′ G), 4.08 (3H, s, m^7), 3.22 (1H, dd, $J_{CH2'-CH2''}$ = 15.7, $J_{CH2'-P\beta}$ = 20.0, $H_{CH2'}$), 3.14 (1H, dd, $J_{CH2'-CH2''}$ = 15.7, $J_{CH2''-P\beta}$ = 20.4, $H_{CH2''}$); ^{31}P NMR (162 MHz, D_2O, 25°C) δ_P: 11.22 (1P, dt, $J_{P\alpha-P\beta}$ = 26.4, $J_{P\beta-CH2}$ = 20.2, Pβ), -10.65 (1P, br d, $J_{P\alpha-P\beta}$ = 26.4, Pα); HRMS (-) ESI *m/z* found: 786.1509, calc. for $C_{24}H_{30}N_{13}O_{14}P_2^-$: 786.1510.

(2b) m^{7}G-triazole-CH$_2$pppG

Obtained according to GP F from **11b** (2000 mOD, 0.166 mmol, 229.3 mM) and **16b** (0.497 mmol, 687.3 mM) while stirring with $CuSO_4{\cdot}5H_2O$ (24.8 mg, 0.099 mmol, 138 mM) and sodium ascorbate (39.4 mg, 0.199 mmol, 275 mM) in 0.722 mL of DMF/H_2O (2.2:1, v/v) for 6 h. After quenching the reaction with Na_2EDTA (368.8 mg, 0.99 mmol), the product was subjected to RP HPLC purification which afforded **2b** as ammonium salt.

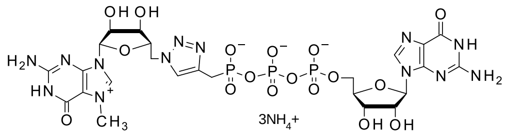

^{1}H NMR (400 MHz, D_2O, 25 °C) δ_H: 8.03 (1H, s, H8 G), 7.98 (1H, s, $H_{triazole}$), 5.90 (1H, d, $J_{1'-2'}$ = 3.1, H1′ m^{7}G), 5.83 (1H, d, $J_{1'-2'}$ = 6.3, H1′ G), 4.78 (overlapped with HDO, H5′ and H5″ m^{7}G), 4.73 (1H, dd, $J_{1'-2'}$ = 6.3, $J_{1'-2'}$ = 4.7, H2′ G), 4.46-4.53 (3H, overlapped H2′ m^{7}G, H3′ G, H4′ m^{7}G), 4.36 (1H, dd, *J* = 5.5, 6.6, H3′ m^{7}G), 4.29-4.31 (1H, m, H4′ G), 4.19-4.23 (2H, m, H5′ and H5′′ G), 4.09 (3H, s, m^7), 3.14-3.31 (2H, m, H_{CH2}); ^{31}P NMR (162 MHz, D_2O, 25°C) δ_P: 11.30-11.71 (1P, m, Pγ), -10.72 (1P, br d, $J_{P\alpha-P\beta}$ = 19.1, Pα), -22.49 (1P, dd, $J_{P\alpha-P\beta}$ = 19.3, $J_{P\gamma-P\beta}$ = 22.0, Pβ); HRMS (-) ESI *m/z* found: 866.1174, calc. for $C_{24}H_{31}N_{13}O_{17}P_3^-$: 866.1174.

(2c) m^{7}GppCH$_2$-triazole-G

Obtained according to GP F from **11c** (2000 mOD, 0.175 mmol, 200 mM) and **16a** (192.0 mg, 0.526 mmol, 599 mM) while stirring with $CuSO_4{\cdot}5H_2O$ (26.3 mg, 0.105 mmol, 120 mM) and sodium ascorbate (41.6 mg, 0.210 mmol, 240 mM) in 0.878 mL of DMF/H_2O (2.2:1, v/v) for 3 h. After quenching the reaction with Na_2EDTA (391.1 mg, 1.05 mmol), the product was subjected to RP HPLC purification which afforded **2c** as ammonium salt.

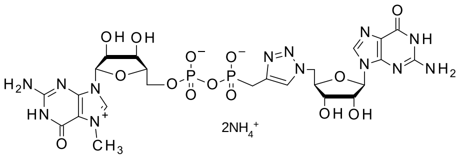

^{1}H NMR (400 MHz, D_2O, 25 °C) δ_H: 7.78 (1H, d, *J* = 2.0, $H_{triazole}$), 7.67 (1H, s, H8 G), 5.98 (1H, d, $J_{1'-2'}$ = 3.5, H1′ m^{7}G), 5.82 (1H, d, $J_{1'-2'}$ = 3.5, H1′ G), 4.78 (2H, overlapped with HDO, H5′ and H5″ G), 4.62 (1H, dd, $J_{1'-2'}$ = 3.5, $J_{2'-3'}$ = 5.1, H2′ m^{7}G), 4.56 (1H, dd, $J_{1'-2'}$ = 3.5, $J_{2'-3'}$ = 4.7, H2′ G), 4.43-4.49 (2H, overlapped H3′ G, H4′ G), 4.41 (1H, dd, $J_{2'-3'}$ = 5.1, $J_{3'-4'}$ = 5.5, H3′ m^{7}G), 4.30-4.34 (1H, m, H4′ m^{7}G), 4.20 (1H, ddd, $J_{5'-5''}$ = 11.7, *J* = 2.4, 4.3, H5′ G), 4.10 (1H, ddd, $J_{5'-5''}$ = 11.7, *J* = 2.4, 5.5, H5′ G), 4.03 (3H, s, m^7), 3.22 (1H, dd, $J_{CH2'-CH2''}$ = 15.7, $J_{CH2'-P\beta}$ = 20.4, $H_{CH2'}$), 3.09 (1H, dd, $J_{CH2'-CH2''}$ = 15.7, $J_{CH2''-P\beta}$ = 20.4, $H_{CH2''}$); ^{31}P NMR (162 MHz, D_2O, 25°C) δ_P: 11.48 (1P, dt, $J_{P\beta-P\gamma}$ = 27.1, $J_{P\beta-CH2}$ =20.4, Pβ), -10.50 (1P, br d, $J_{P\beta-P\gamma}$ = 27.1, Pγ); HRMS (-) ESI *m/z* found: 786.1511, calc. for $C_{24}H_{30}N_{13}O_{14}P_2^-$: 786.1510.

(2d) m^{7}GpppCH$_2$-triazole-G

Obtained according to GP F from **11d** (2000 mOD, 0.175 mmol, 200 mM) and **16a** (192.0 mg, 0.526 mmol, 599 mM) while stirring with $CuSO_4{\cdot}5H_2O$ (26.3 mg, 0.105 mmol, 120 mM) and sodium ascorbate (41.6 mg, 0.210 mmol, 240 mM) in 0.878 mL of DMF/H_2O (2.2:1, v/v) for 2.5 h. After quenching the reaction with Na_2EDTA (391.1 mg, 1.05 mmol), the product was subjected to RP HPLC purification which afforded **2d** as ammonium salt.

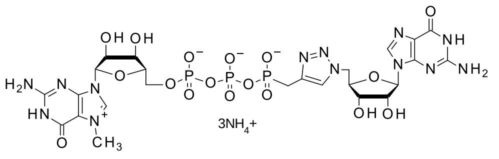

^{1}H NMR (400 MHz, D_2O, 25 °C) δ_H: 7.90 (1H, s, H8 G or $H_{triazole}$), 7.68 (1H, s, H8 G or $H_{triazole}$), 5.98 (1H, d, $J_{1'-2'}$ = 3.5, H1′ m^{7}G), 5.80 (1H, d, $J_{1'-2'}$ = 3.9, H1′ G), 4.80 (2H, overlapped with HDO, H5′ and H5″ G), 4.63 (1H, dd, $J_{1'-2'}$ = 3.5, $J_{2'-3'}$ = 5.1, H2′ m^{7}G), 4.57 (1H, dd, $J_{1'-2'}$ = 3.9, $J_{2'-3'}$ = 4.3, H2′ G), 4.44-4.51 (3H, overlapped H3′ G, H3′ m^{7}G, H4′ G), 4.41 (1H, dd, $J_{2'-3'}$ = 5.1, $J_{3'-4'}$ = 5.5, H3′ m^{7}G), 4.35-4.38 (1H, m, H4′ m^{7}G), 4.30-4.35 (1H, m, H5′ G), 4.20-4.24 (1H, m, H5″ G), 4.05 (3H, s, m^7), 3.26 (1H, dd, $J_{CH2'-CH2''}$ = 16.4, $J_{CH2'-P\beta}$ = 20.0, $H_{CH2'}$), 3.16 (1H, dd, $J_{CH2'-CH2''}$ = 16.4, $J_{CH2''-P\beta}$ = 20.0, $H_{CH2''}$); ^{31}P NMR (162 MHz, D_2O, 25°C) δ_P: 11.53 (1P, dt, $J_{CH2-P\beta}$ = 20.0, $J_{P\gamma-P\beta}$ = 26.4, Pβ), - 10.77 (1P, br d, $J_{P\gamma-P\delta}$ = 19.1, Pδ), - 22.37 (1P, dd, $J_{P\gamma-P\delta}$ = 19.1, $J_{P\gamma-P\beta}$ = 26.4, Pγ); HRMS (-) ESI *m/z* found: 866.1176, calc. for $C_{24}H_{31}N_{13}O_{17}P_3^-$: 866.1174.

(3a) m^{7}G-triazole-ppG

Obtained according to GP F from **12a** (1500 mOD, 0.124 mmol, 99 mM) and **16b** (0.372 mmol, 0.296 mM) while stirring with $CuSO_4{\cdot}5H_2O$ (6.2 mg, 0.025 mmol, 20 mM) and sodium ascorbate (9.9 mg, 0.050 mmol, 39 mM) in 1.260mL of DMF/H_2O (1.7:1, v/v) for 1 h. After quenching the reaction with Na_2EDTA (365.0 mg, 0.98 mmol), the product was subjected to RP HPLC purification which afforded **3a** as ammonium salt.

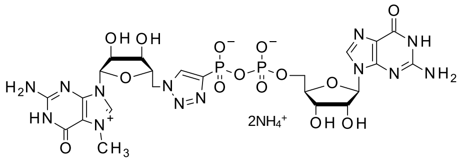

^{1}H NMR (400 MHz, D_2O, 25 °C) δ_H: 8.26 (1H, s, H8 G or $H_{triazole}$), 7.89 (1H, s, H8 G or $H_{triazole}$), 5.84 (1H, d, $J_{1'-2'}$ = 3.9, H1′ m^{7}G), 5.79 (1H, d, $J_{1'-2'}$ = 6.3, H1′ G), 4.88-4.90 (2H, m, H5′ and H5″ m^{7}G), 4.60 (1H, dd, $J_{1'-2'}$ = 6.3, $J_{2'-3'}$ = 5.1, H2′ G), 4.50-4.53 (1H, m, H4′ m^{7}G), 4.48 (1H, dd, $J_{1'-2'}$ = 3.9, $J_{2'-3'}$ = 5.5, H2′ m^{7}G), 4.38 (1H, dd, $J_{2'-3'}$ = 5.1, $J_{3'-4'}$ = 3.5, H3′ G), 4.31 (1H, dd, $J_{2'-3'}$ = 5.5, $J_{3'-4'}$ = 5.9, H3′ m^{7}G), 4.21 (1H, m, H4′ G), 4.08-4.09 (2H, m, H5′ and H5″ G), 4.06 (3H, s, m^7); ^{31}P NMR (162 MHz, D_2O, 25°C) δ_P: -7.52 (1P, d, $J_{P\alpha-P\beta}$ = 25.1, Pβ), -10.81 (1P, dt, $J_{P\alpha-P\beta}$ = 25.1, $J_{P\alpha-5'/5''}$ = 4.4, Pα); HRMS (-) ESI *m/z* found: 772.1362, calc. for $C_{23}H_{28}N_{13}O_{14}P_2^-$: 772.1354.

(3b) m^{7}G-triazole-pppG

Obtained according to GP F from **12b** (1500 mOD, 0.124 mmol, 85 mM) and **16b** (0.372 mmol, 254 mM) while stirring with $CuSO_4{\cdot}5H_2O$ (12.4 mg, 0.050 mmol, 34 mM) and sodium ascorbate (19.6 mg, 0.099 mmol, 68 mM) in 1.470 mL of DMF/H_2O (1.2:1, v/v) for 2 h. After quenching the reaction with Na_2EDTA (186.3 mg, 0.50 mmol), the product was subjected to RP HPLC purification which afforded **3b** as ammonium salt.

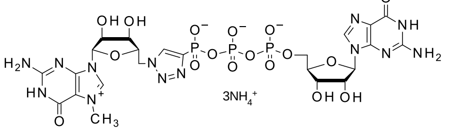

^{1}H NMR (400 MHz, D_2O, 25 °C) δ_H: 8.36 (1H, s, H8 G or $H_{triazole}$), 7.99 (1H, s, H8 G or $H_{triazole}$), 5.88 (1H, d, $J_{1'-2'}$ = 3.5, H1′ m^{7}G), 5.81 (1H, d, $J_{1'-2'}$ = 5.9, H1′ G), 4.92 (1H, dd, $J_{5'-5''}$ = 15.1, $J_{4'-5'}$ = 3.3, H5′ m^{7}G), 4.84 (1H, dd, $J_{5'-5''}$ = 15.1, $J_{4'-5'}$ = 5.9, H5″ m^{7}G), 4.68 (1H, dd, $J_{1'-2'}$ = 5.9, $J_{2'-3'}$ = 5.4, H2′ G), 4.50-4.54 (1H, m, H4′ m^{7}G), 4.51 (1H, dd, $J_{1'-2'}$ = 3.5, $J_{2'-3'}$ = 5.5, H2′ m^{7}G), 4.46 (1H, dd, $J_{2'-3'}$ = 5.4, $J_{3'-4'}$ = 3.3, H3′ G), 4.30 (1H, dd, $J_{2'-3'}$ = 5.5, $J_{3'-4'}$ = 6.3, H3′ m^{7}G), 4.25-4.26 (1H, m, H4′ G), 4.09-4.11 (2H, m, H5′ and H5″ G), 4.08 (3H, s, m^7); ^{31}P NMR (162 MHz, D_2O, 25°C) δ_P: -7.21 (1P, d, $J_{P\gamma-P\beta}$ = 22.0, Pγ), -10.62 (1P, dt, $J_{P\alpha-P\beta}$ = 19.1, $J_{P\alpha-5'/5''}$ = 5.1, Pα), -22.46 (1P, dd, $J_{P\gamma-P\beta}$ = 22.0, $J_{P\alpha-P\beta}$ = 19.1, Pβ); HRMS (-) ESI *m/z* found: 852.1031, calc. for $C_{23}H_{29}N_{13}O_{17}P_3^-$: 852.1017.

(3c) m^{7}Gpp-triazole-G

Obtained according to GP F from **12c** (1000 mOD, 0.088 mmol, 162 mM) and **16a** (81.0 mg, 0.485 mmol, 485 mM) while stirring with $CuSO_4{\cdot}5H_2O$ (8.8 mg, 0.035 mmol, 65 mM) and sodium ascorbate (13.9 mg, 0.070 mmol, 129 mM) in 0.542 mL of DMF/H_2O (1.2:1, v/v) for 24 h. After quenching the reaction with Na_2EDTA (130.3 mg, 0.35 mmol), the product was subjected to RP HPLC purification which afforded **3c** as ammonium salt.

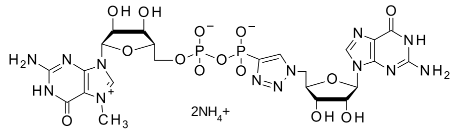

^{1}H NMR (400 MHz, D_2O, 25 °C) δ_H: 8.94 (1H, s, H8 m^{7}G), 8.27 (1H, s, H8 G or $H_{triazole}$), 7.77 (1H, s, H8 G or $H_{triazole}$), 5.90 (1H, d, $J_{1'-2'}$ = 3.3, H1′ m^{7}G), 5.79 (1H, d, $J_{1'-2'}$ = 5.5, H1′ G), 4.95 (1H, dd, $J_{5'-5''}$ = 14.9, $J_{4'-5'}$ = 7.2, H5′ G), 4.89 (1H, dd, $J_{5'-5''}$ = 14.9, $J_{4'-5'}$ = 3.5, H5″ G), 4.58 (1H, dd, $J_{1'-2'}$ = 3.3, $J_{2'-3'}$ = 4.7, H2′ m^{7}G), 4.53 (1H, dd, $J_{1'-2'}$ = 5.5, $J_{2'-3'}$ = 5.5, H2′ G), 4.48-4.51 (1H, m, H4′ G), 4.37-4.40 (2H, overlapped H3′ G and m^{7}G), 4.25-4.27 (1H, m, H4′ m^{7}G), 4.22 (1H, ddd, $J_{5'-5''}$ = 11.7, J = 4.3, 2.4, H5′ m^{7}G), 4.12 (2H, ddd, $J_{5'-5''}$ = 11.7, J = 5.3, 2.2, H5″ m^{7}G), 3.94 (3H, s, m^7); ^{31}P NMR (162 MHz, D_2O, 25°C) δ_P: -7.25 (1P, d, $J_{P\beta-P\gamma}$ = 2.5, Pβ), -10.86 (1P, br d, $J_{P\beta-P\gamma}$ = 2.5, Pγ); HRMS (-) ESI *m/z* found: 772.1363, calc. for $C_{23}H_{28}N_{13}O_{14}P_2^-$: 772.1354.

(3d) m^{7}Gppp-triazole-G

Obtained according to GP F from **12d** (1000 mOD, 0.088 mmol, 162 mM) and **16a** (81.0 mg, 0.485 mmol, 485 mM) while stirring with $CuSO_4{\cdot}5H_2O$ (8.8 mg, 0.035 mmol, 65 mM) and sodium ascorbate (13.9 mg, 0.070 mmol, 129 mM) in 0.542 mL of DMF/H_2O (1.2:1, v/v) for 4 h. After quenching the reaction with Na_2EDTA (130.3 mg, 0.35 mmol), the product was subjected to RP HPLC purification which afforded **3d** as ammonium salt.

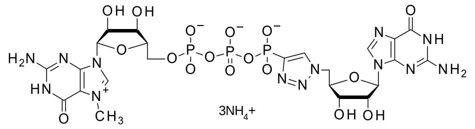

^{1}H NMR (400 MHz, D_2O, 25 °C) δ_H: 9.11 (1H, s, H8 m^{7}G), 8.33 (1H, s, H8 G or $H_{triazole}$), 7.82 (1H, s, H8 G or $H_{triazole}$), 5.96 (1H, d, $J_{1'-2'}$ = 3.5, H1′ m^{7}G), 5.84 (1H, d, $J_{1'-2'}$ = 5.9, H1′ G), 4.90-4.91 (2H, m, H5′ and H5″ G), 4.62 (1H, dd, $J_{1'-2'}$ = 3.5, $J_{2'-3'}$ = 5.1, H2′ m^{7}G), 4.50-4.55 (2H, overlapped H2′ and H4′ G), 4.45 (1H, dd, $J_{2'-3'}$ = 5.1, $J_{3'-4'}$ = 4.7, H3′ m^{7}G), 4.41 (1H, dd, J = 5.1, 5.5, H3′ G), 4.30-4.33 (1H, m, H4′ m^{7}G), 4.23 (1H, ddd, $J_{5'-5''}$ = 11.7, J = 4.3, 2.7, H5′ m^{7}G), 4.14 (2H, ddd, $J_{5'-5''}$ = 11.7, J = 5.5, 2.4, H5″ m^{7}G), 4.01 (3H, s, m^7); ^{31}P NMR (162 MHz,

D_2O, 25°C) δ_P: -7.12 (1P, d, $J_{P\beta\text{-}P\gamma}$ = 22.0, Pβ), -10.68 (1P, br d, $J_{P\delta\text{-}P\gamma}$ = 17.6, Pδ), -22.50 (1P, dd, $J_{P\beta\text{-}P\gamma}$ = 22.0, $J_{P\delta\text{-}P\gamma}$ = 17.6, Pγ); HRMS (-) ESI *m/z* found: 852.1030, calc. for $C_{24}H_{31}N_{13}O_{17}P_3^-$: 852.1017.

(3e) $m_2^{7,2'\text{-}O}$Gppp-triazole-G

Obtained according to GP F from **12f** (650 mOD, 0.057 mmol, 100 mM) and **16a** (52.7 mg, 0.171 mmol, 300 mM) while stirring with $CuSO_4{\cdot}5H_2O$ (2.7 mg, 0.011 mmol, 20 mM) and sodium ascorbate (4.4 mg, 0.022 mmol, 40 mM) in 0.570 mL of DMF/H_2O (1:1, v/v) for 2 h. After quenching the reaction with Na_2EDTA (41.0 mg, 0.11 mmol), the product was subjected to RP HPLC purification which afforded **3d** as ammonium salt.

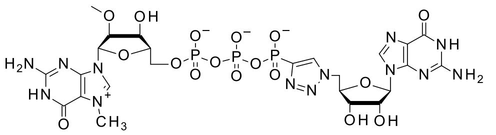

^{1}H NMR (400 MHz, D_2O, 25 °C) δ_H: 9.10 (1H, q, $J_{H8\text{-}m7}$ = 0.8, H8 m^{7}G), 8.34 (1H, s, H8 G or $H_{triazole}$), 7.85 (1H, s, H8 G or $H_{triazole}$), 6.00 (1H, d, $J_{1'\text{-}2'}$ = 3.1, H1′ m^{7}G), 5.84 (1H, d, $J_{1'\text{-}2'}$ = 4.7, H1′ G), 4.91-4.91 (2H, m, H5′ and H5′′ G), 4.52-4.56 (2H, overlapped H4′ G and H3′ m^{7}G), 4,51 (1H, dd, $J_{1'\text{-}2'}$ = 4.7, $J_{2'\text{-}3'}$ = 4.3, H2′ G), 4.40 (1H, t, *J* = 5.5, H3′ G), 4.23-4.30 (3H, overlapped H2′, H4′ and H5′ m^{7}G), 4.14 (1H, ddd, $J_{5'\text{-}5''}$ = 12.5, $J_{4'\text{-}5''}$ = 5.1, $J_{P\delta\text{-}5''}$ = 2.9, H5′′ m^{7}G), 4.02 (3H, d, $J_{H8\text{-}m7}$ = 0.8, m^7), 3.58 (3H, s, m$^{2'\text{-}O}$); ^{31}P NMR (162 MHz, D_2O, 25°C) δ_P: -7.14 (1P, d, $J_{P\beta\text{-}P\gamma}$ = 22.0, Pβ), -16.69 (1P, ddd, $J_{P\delta\text{-}P\gamma}$ = 19.1, $J_{P\delta\text{-}5'}$ = 4.4, $J_{P\delta\text{-}5''}$ = 2.9, Pδ), -22.45 (dd, $J_{P\beta\text{-}P\gamma}$ = 22.0, $J_{P\delta\text{-}P\gamma}$ = 19.1, Pγ); HRMS (-) ESI *m/z* found: 866.1192, calc. for $C_{24}H_{31}N_{13}O_{17}P_3^-$: 866.1174.

(4a) m^{7}G-triazole-OCH$_2$ppG

Obtained according to GP F from **13a** (890 mOD, 0.074 mmol, 97 mM) and **16b** (0.221 mmol, 291 mM) while stirring with $CuSO_4$ (3.7 mg, 0.015 mmol, 19 M) and sodium ascorbate (5.9 mg, 0.030 mmol, 39 mM) in 760 mL of DMF/H_2O (1:1.9, v/v) for 2 h. After quenching the reaction with Na_2EDTA (55.9 mg, 0.15 mmol), the product was subjected to RP HPLC purification which afforded **4a** as ammonium salt.

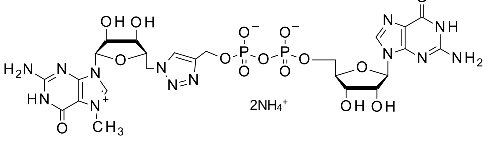

^{1}H NMR (400 MHz, D_2O, 25 °C) δ_H: 7.99 (1H, s, H8 G or $H_{triazole}$), 7.87 (1H, s, H8 G or $H_{triazole}$), 5.88 (1H, d, $J_{1'\text{-}2'}$ = 2.7, H1′ m^{7}G), 5.81 (1H, d, $J_{1'\text{-}2'}$ = 5.9, H1′ G), 4.98 (1H, dd, $J_{CH2'\text{-}CH2''}$ = 12.7, $J_{CH2'\text{-}P\beta}$ = 6.6, $H_{CH2'}$), 4.94 (1H, dd, $J_{CH2'\text{-}CH2''}$ = 12.7, $J_{CH2''\text{-}P\beta}$ = 7.0, $H_{CH2''}$), 4.80 (2H, overlapped with HDO, H5′ and H5′′ m^{7}G), 4.70 (1H, dd, $J_{1'\text{-}2'}$ = 5.9, $J_{2'\text{-}3'}$ = 5.5, H2′ G), 4.60 (1H, dd, $J_{1'\text{-}2'}$ = 2.7, $J_{2'\text{-}3'}$ = 5.1, H2′ m^{7}G), 4.46 (1H, dd, $J_{2'\text{-}3'}$ = 5.5, $J_{3'\text{-}4'}$ = 3.1, H3′ G), 4.40-4.43 (2H, m, H4′ m^{7}G), 4.36 (1H, dd, $J_{2'\text{-}3'}$ = 5.1, $J_{3'\text{-}4'}$ = 7.8, H3′ m^{7}G), 4.28-4.29 (2H, m, H4′ G), 4.12-4.22 (2H, m, H5′ and H5′′ G), 4.08 (3H, s, m^7); ^{31}P NMR (162 MHz, D_2O, 25°C) δ_P: -10.77-(-10.14) (2P, overlapped Pα and Pβ); HRMS (-) ESI *m/z* found: 802.1476, calc. for $C_{24}H_{30}N_{13}O_{15}P_2^-$: 802.1460.

(4b) m^{7}G-triazole-OCH$_2$pppG

Obtained according to GP F from **13b** (800 mOD, 0.066 mmol, 79 mM) and **16b** (0.198 mmol, 238 mM) while stirring with $CuSO_4$ (6.6 mg, 0.026 mmol, 32 mM) and sodium ascorbate (10.5 mg, 0.053 mmol, 64 mM) in 0.834 mL of DMF/H_2O (1:1.8, v/v) for 2 h. After quenching the reaction with Na_2EDTA (245.9 mg, 0.66 mmol), the product was subjected to RP HPLC purification which afforded **4b** as ammonium salt.

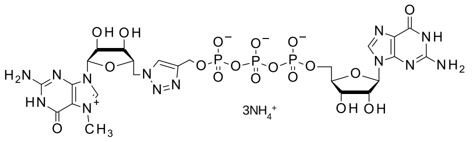

^{1}H NMR (400 MHz, D_2O, 25 °C) δ_H: 8.02 (1H, s, H8 G or $H_{triazole}$), 8.01 (1H, s, H8 G or $H_{triazole}$), 5.90 (1H, d, $J_{1'\text{-}2'}$ = 2.4, H1′ m^{7}G), 5.81 (1H, d, $J_{1'\text{-}2'}$ = 6.3, H1′ G), 5.0 (2H, d, $J_{CH2\text{-}P\gamma}$ = 7.0, H_{CH2}), 4.84 (1H, dd, $J_{5'\text{-}5''}$ = 15.3, $J_{4'\text{-}5'}$ = 2.5, H5′ m^{7}G), 4.78 (1H, dd, $J_{5'\text{-}5''}$ = 15.3, $J_{4'\text{-}5''}$ = 3.1, H5′′ m^{7}G), 4.74 (1H, dd, $J_{1'\text{-}2'}$ = 6.3, $J_{2'\text{-}3'}$ = 5.5, H2′ G), 4.64 (1H, dd, $J_{1'\text{-}2'}$ = 2.4, $J_{2'\text{-}3'}$ = 4.7, H2′ m^{7}G), 4. 51 (1H, dd, $J_{2'\text{-}3'}$ = 5.5, $J_{3'\text{-}4'}$ = 3.5, H3′ G), 4.40-4.46 (2H, overlapped H3′ and H4′ m^{7}G), 4.27-4.31 (1H, m, H4′ G), 4.20-4.26 (2H, m, H5′ and H5′′ m^{7}G), 4.08 (3H, s, m^7); ^{31}P NMR (162 MHz, D_2O, 25°C) δ_P: -10.83 – (-10.51) (2P, overlapped Pα and Pγ), -22.27 (1P, t, $J_{P\beta\text{-}P\gamma}$ = $J_{P\alpha\text{-}P\gamma}$ = 19.1, Pγ); HRMS (-) ESI *m/z* found: 882.1140, calc. for $C_{24}H_{31}N_{13}O_{18}P_3^-$: 882.1123.

(4c) m^{7}GppOCH$_2$-triazole-G

Obtained according to GP F from **13c** (890 mOD, 0.078 mmol, 83 mM) and **16a** (72.1 mg, 0.234 mmol, 250 mM) while stirring with $CuSO_4$ (3.9 mg, 0.016 mmol, 16.7 mM) and sodium ascorbate (6.2 mg, 0.031 mmol, 33 mM) in 0.937 mL of DMF/H_2O (1:1.4, v/v) for 4 h. After quenching the reaction with Na_2EDTA (59.6 mg, 0.16 mmol), the product was subjected to RP HPLC purification which afforded **4c** as ammonium salt.

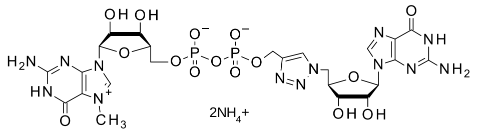

^{1}H NMR (400 MHz, D_2O, 25 °C) δ_H: 7.92 (1H, s, H8 G or $H_{triazole}$), 7.68 (1H, s, H8 G or $H_{triazole}$), 5.96 (1H, d, $J_{1'\text{-}2'}$ = 3.9, H1′ G or m^{7}G), 5.82 (1H, d, $J_{1'\text{-}2'}$ = 3.2, H1′ G or m^{7}G), 4.98 (1H, dd, $J_{CH2'\text{-}CH2''}$ = 12.5, $J_{CH2'\text{-}P\beta}$ = 6.6, $H_{CH2'}$), 4.90 (1H, dd, $J_{CH2'\text{-}CH2''}$ = 12.5, $J_{CH2''\text{-}P\beta}$ = 6.6, $H_{CH2''}$), 4.81-4.82 (2H, overlapped with HDO, H5′ and H5′′G), 4.65 (1H, dd, $J_{1'\text{-}2'}$ = 3.5, $J_{2'\text{-}3'}$ = 5.1, H2′ G or m^{7}G), 5.82 (1H, dd, $J_{1'\text{-}2'}$ = 3.9, $J_{2'\text{-}3'}$ = 4.7, H2′ G or m^{7}G), 4.40-4.48 (3H, overlapped H3′ G, H3′ m^{7}G and H4′ G), 4.31-4.34 (1H, m, H4′ m^{7}G), 4.26 (1H, ddd, $J_{5'\text{-}5''}$ = 11.7, *J* = 3.5, 2.5, H5′

m^{7}G), 4.16 (1H, ddd, $J_{5'\text{-}5''}$ = 11.7, J = 4.7, 2.5, H5'' m^{7}G), 3.99 (3H, s, m^7); ^{31}P NMR (162 MHz, D_2O, 25°C) δ_P: -10.77-(-10.34) (2P, overlapped Pα and Pβ); HRMS (-) ESI *m/z* found: 804.1607, calc. for $C_{24}H_{32}N_{13}O_{15}P_2^+$: 804.1616.

(4d) m^{7}GpppOCH$_2$-triazole-G

Obtained according to GP F from **13d** (690 mOD, 0.061 mmol, 59 mM) and **16a** (56.3 mg, 0.183 mmol, 176 mM) while stirring with $CuSO_4$ (3.0 mg, 0.012 mmol, 12 mM) and sodium ascorbate (4.8 mg, 0.024 mmol, 24 mM) in 1.033 mL of DMF/H_2O (1:2.0, v/v) for 1 h. After quenching the reaction with Na_2EDTA (44.7 mg, 0.12 mmol), the product was subjected to RP HPLC purification which afforded **4d** as ammonium salt.

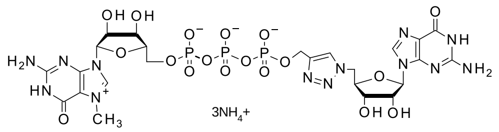

^{1}H NMR (400 MHz, D_2O, 25 ^{o}C) δ_H: 9.15 (1H, s, H8 m^{7}G), 8.00 (1H, s, H8 G or $H_{triazole}$), 7.74 (1H, s, H8 G or $H_{triazole}$), 5.98 (1H, d, $J_{1'\text{-}2'}$ = 3.5, H1' G or m^{7}G), 5.82 (1H, d, $J_{1'\text{-}2'}$ = 3.1, H1' G or m^{7}G), 5.02 (1H, dd, $J_{CH2'\text{-}CH2''}$ = 12.5, $J_{CH2'\text{-}P\beta}$ = 7.0, $H_{CH2'}$), 5.96 (1H, dd, $J_{CH2'\text{-}CH2''}$ = 12.5, $J_{CH2''\text{-}P\beta}$ = 7.0, $H_{CH2''}$), 4.81-4.83 (2H, overlapped with HDO, H5' and H5''G), 4.67 (1H, dd, $J_{1'\text{-}2'}$ = 3.1, $J_{2'\text{-}3'}$ = 3.9, H2' G or m^{7}G), 5.66 (1H, dd, $J_{1'\text{-}2'}$ = 3.5, $J_{2'\text{-}3'}$ = 3.5, H2' G or m^{7}G), 4.50-4.53 (2H, overlapped H3' G, H3' m^{7}G), 4.41-4.45 (1H, m, H4' m^{7}G), 4.32-4.37 (2H,overlapped H4' and H5' m^{7}G), 4.24 (1H, ddd, $J_{5'\text{-}5''}$ = 11.7, J = 5.1, 2.4, H5'' m^{7}G), 4.05 (3H, s, m^7); ^{31}P NMR (162 MHz, D_2O, 25°C) δ_P: -10.77 – (-10.56) (2P, overlapped Pα and Pγ), -22.17 (1P, t, J = 19.1, 20.5, Pγ); HRMS (-) ESI *m/z* found: 882.1143, calc. for $C_{24}H_{31}N_{13}O_{18}P_3^-$: 882.1123.

(5a) m^{7}G-triazole-SCH$_2$ppG

Obtained according to GP F from **14a** (750 mOD, 0.062 mmol, 93 mM) and **16b** (0.186 mmol, 279 mM) while stirring with $CuSO_4$ (6.2 mg, 0.025 mmol, 37 M) and sodium ascorbate (9.8 mg, 0.050 mmol, 74 mM) in 0.668 mL of DMF/H_2O (1.5:1, v/v) for 3 h. After quenching the reaction with Na_2EDTA (93.1mg, 0.25 mmol), the product was subjected to RP HPLC purification which afforded **5a** as ammonium salt.

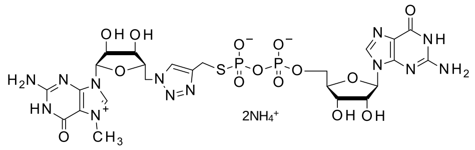

^{1}H NMR (400 MHz, D_2O, 25 ^{o}C) δ_H: 8.04 (1H, s, H8 G or $H_{triazole}$), 7.63 (1H, s, H8 G or $H_{triazole}$), 5.86 (1H, d, $J_{1'\text{-}2'}$ = 2.4, H1' m^{7}G), 5.82 (1H, d, $J_{1'\text{-}2'}$ = 5.9, H1' G), 4.75-4.76 (2H, m, H5' and H5'' m^{7}G), 4.73 (1H, dd, $J_{1'\text{-}2'}$ = 5.9, $J_{2'\text{-}3'}$ = 5.3, H2' G), 4.60 (1H, dd, $J_{1'\text{-}2'}$ = 2.4, $J_{2'\text{-}3'}$ = 4.9, H2' m^{7}G), 4.48 (1H, dd, $J_{2'\text{-}3'}$ = 5.3, $J_{3'\text{-}4'}$ = 3.5, H3' G), 4.37-4.43 (2H, overlapped H3' and H4' m^{7}G), 4.29-4.31 (1H, m, H4' G), 4.15-4.17 (2H, m, H5' and H5'' G), 4.10 (3H, s, m^7), 4.02 (1H, dd, $J_{CH2'\text{-}CH2''}$ = 14.1, $J_{CH2'\text{-}P\beta}$ = 12.1, $H_{CH2'}$), 3.93 (1H, dd, $J_{CH2'\text{-}CH2''}$ = 14.1, $J_{CH2''\text{-}P\beta}$ = 11.4, $H_{CH2''}$); ^{31}P NMR (162 MHz, D_2O, 25°C) δ_P: 6.72 (1P, dt, $J_{P\alpha\text{-}P\beta}$ = 28.6, $J_{P\beta\text{-}CH2}$ =11.7, Pβ), -11.97 (1P, br d, $J_{P\alpha\text{-}P\beta}$ = 28.6, Pα); HRMS (-) ESI *m/z* found: 818.1244, calc. for $C_{24}H_{30}N_{13}O_{14}P_2S^-$: 818.1231.

(5b) m^{7}G-triazole-SCH$_2$pppG

Obtained according to GP F from **14b** (800 mOD, 0.066 mmol, 98 mM) and **16b** (0.198 mmol, 295 mM) while stirring with $CuSO_4$ (6.6 mg, 0.026 mmol, 39 M) and sodium ascorbate (10.5 mg, 0.053 mmol, 79 mM) in 0.674 mL of DMF/H_2O (2:2, v/v) for 2 h. After quenching the reaction with Na_2EDTA (96.8 mg, 0.26 mmol), the product was subjected to RP HPLC purification which afforded **5b** as ammonium salt.

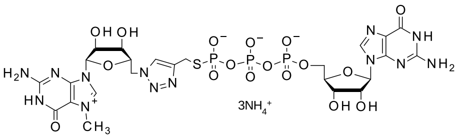

^{1}H NMR (400 MHz, D_2O, 25 ^{o}C) δ_H: 8.03 (1H, s, H8 G or $H_{triazole}$), 7.89 (1H, s, H8 G or $H_{triazole}$), 5.90 (1H, d, $J_{1'\text{-}2'}$ = 2.4, H1' m^{7}G), 5.83 (1H, d, $J_{1'\text{-}2'}$ = 6.3, H1' G), 4.73-4.84 (3H, overlapped H5' and H5" m^{7}G, H2' G), 4.65 (1H, dd, $J_{1'\text{-}2'}$ = 2.4, $J_{2'\text{-}3'}$ = 4.7, H2' m^{7}G), 4.53 (1H, dd, J = 3.5, 5.1, H3' G), 4.41-4.47 (2H, overlapped H3' and H4' m^{7}G), 4.30-4.32 (1H, m, H4' G), 4.23-4.27 (2H, m, H5' and H5" G), 4.09 (1H, s, m^7), 4.07 (1H, dd, $J_{CH2'\text{-}CH2''}$ = 14.3, $J_{CH2'\text{-}P\gamma}$ = 11.9, $H_{CH2'}$), 3.93 (1H, dd, $J_{CH2'\text{-}CH2''}$ = 14.3, $J_{CH2''\text{-}P\gamma}$ = 11.9, $H_{CH2''}$); ^{31}P NMR (162 MHz, D_2O, 25°C) δ_P: 8.01 (1P, dt, $J_{P\gamma\text{-}P\beta}$ = 27.9, $J_{P\gamma\text{-}CH2}$ = 11.9, Pγ), -10.58 (1P, dt, $J_{P\alpha\text{-}P\beta}$ = 19.1, $J_{P\alpha\text{-}5'/5''}$ = 5.1, Pα), -22.92 (1P, dd, $J_{P\gamma\text{-}P\beta}$ = 27.9, $J_{P\alpha\text{-}P\beta}$ = 19.1, Pβ); HRMS (-) ESI *m/z* found: 898.0911, calc. for $C_{24}H_{31}N_{13}O_{17}P_3S^-$: 898.0894.

(5c) m^{7}GppSCH$_2$-triazole-G

Obtained according to GP F from **14c** (825 mOD, 0.072 mmol, 113 mM) and **16a** (66.5 mg, 0.216 mmol, 340 mM) while stirring with $CuSO_4$ (3.6 mg, 0.014 mmol, 23 mM) and sodium ascorbate (5.7 mg, 0.029 mmol, 45 mm) in 0.639 mL of DMF/H_2O (1.7:1, v/v) for 1.5 h. After quenching the reaction with Na_2EDTA (52.2 mg, 0.14 mmol), the product was subjected to RP HPLC purification which afforded **5c** as ammonium salt.

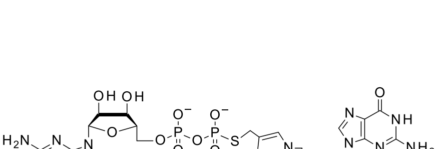

^{1}H NMR (400 MHz, D_2O, 25 ^{o}C) δ_H: 7.76 (1H, s, H8 G or $H_{triazole}$), 7.63 (1H, s, H8 G or $H_{triazole}$), 5.98 (1H, d, $J_{1'\text{-}2'}$ = 3.5, H1' m^{7}G), 5.82 (1H, d, $J_{1'\text{-}2'}$ = 3.1, H1' G), 4.78-4.79 (2H, m, H5' and H5'' m^{7}G), 4.62-4.66 (2H, overlapped H2' G and m^{7}G), 4.49 (1H, dd, J = 5.1, 6.7, H3' G), 4.46 (1H, dd, J = 4.3, 5.1, H3' m^{7}G), 4.41-4.44 (1H, m, H4' G), 4.34-4.37 (1H, m, H4' m^{7}G), 4.26 (1H, ddd, $J_{5'\text{-}5''}$ = 11.9, J = 4.3, 2.5, H5' m^{7}G), 4.14 (2H, ddd, $J_{5'\text{-}5''}$ = 11.9, J = 5.5, 2.4, H5'' m^{7}G), 4.03 (3H, s, m^7), 4.01 (1H, dd, $J_{CH2'\text{-}CH2''}$ = 14.5, $J_{CH2'\text{-}P\beta}$ = 12.5, $H_{CH2'}$), 3.93 (1H, dd, $J_{CH2'\text{-}CH2''}$ = 14.5, $J_{CH2'\text{-}P\beta}$ = 12.3, $H_{CH2''}$); ^{31}P NMR (162 MHz, D_2O, 25°C) δ_P: 7.85 (1P, dt, J = 29.4, 12.4 Hz), -11.22-(-11.04) (1P, m); HRMS (-) ESI *m/z* found: 818.1249, calc. for $C_{24}H_{30}N_{13}O_{14}P_2S^-$: 818.1231.

(5d) m^7GpppSCH$_2$-triazole-G

Obtained according to GP F from **14d** (700 mOD, 0.061 mmol, 100 mM) and **16a** (56.4 mg, 0.183 mmol, 300 mM) while stirring with $CuSO_4$ (3.0 mg, 0.012 mmol, 20 mM) and sodium ascorbate (4.8 mg, 0.024 mmol, 40 mM) in 0.614 mL of DMF/H_2O (1.4:1, v/v) for 1 h. After quenching the reaction with Na_2EDTA (44.7 mg, 0.12 mmol), the product was subjected to RP HPLC purification which afforded **5d** as ammonium salt.

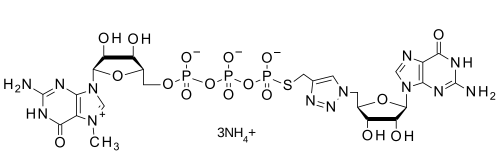

^{1}H NMR (400 MHz, D_2O, 25 °C) δ_H: 7.85 (1H, s, H8 G or $H_{triazole}$), 7.67 (1H, s, H8 G or $H_{triazole}$), 5.98 (1H, d, $J_{1'-2'}$ = 3.5, H1' G or m^7G), 5.82 (1H, d, $J_{1'-2'}$ = 2.7, H1' G or m^7G), 4.78 (2H, overlapped with HDO, H5' and H5" G), 4.65-4.68 (2H, overlapped H2' G and H2' m^7G), 4.51-4.54 (2H, overlapped H3' G and H3' m^7G), 4.40-4.44 (1H, m, H4' m^7G), 4.34-4.48 (2H, overlapped H4' G and H5' G), 4.22-4.27 (1H, m, H5"G), 4.07 (1H, s, m^7), 4.06 (1H, dd, $J_{CH2'-CH2''}$ = 14.3, $J_{CH2'-P\beta}$ = 11.7, $H_{CH2'}$), 3.98 (1H, dd, $J_{CH2'-CH2''}$ = 14.3, $J_{CH2''-P\beta}$ = 11.7, $H_{CH2''}$); ^{31}P NMR (162 MHz, D_2O, 25°C) δ_P: 10.10 (1P, dt, $J_{P\beta-P\gamma}$ = 27.1, $J_{P\beta-CH2}$ = 11.7, Pβ), -8.46 (1P, d, J = 19.1, Pδ), 20.70 (1P, dd, $J_{P\beta-P\gamma}$ = 27.1, $J_{P\delta-P\gamma}$ = 19.1, pγ); HRMS (-) ESI *m/z* found: 898.0913, calc. for $C_{24}H_{31}N_{13}O_{17}P_3S^-$: 898.0894.

(6a) m^7G-triazole-NHCH$_2$ppG

Obtained according to GP F from **15a** (6057 mOD, 0.501 mmol, 358 mM) and **16b** (462.9 mg, 1.503 mmol, 1074 mM) while stirring with $CuSO_4$ (25.0 mg, 0.100 mmol, 72 mM) and sodium ascorbate (39.6 mg, 0.200 mmol, 143 mM) in 1.400 mL of DMF/H_2O (1:1.4, v/v) for 1 h. After quenching the reaction with Na_2EDTA (372.5 mg, 1.00 mmol), the product was subjected to RP HPLC purification which afforded **6a** as ammonium salt.

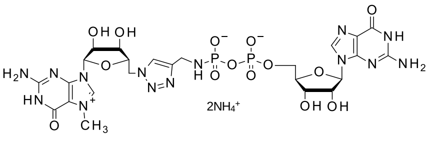

^{1}H NMR (400 MHz, D_2O, 25 °C) δ_H: 8.04 (1H, s, H8 G), 7.69 (1H, s, $H_{triazole}$), 5.90 (1H, d, $J_{1'-2'}$ = 2.5, H1' m^7G), 5.82 (1H, d, $J_{1'-2'}$ = 6.0, H1' G), 4.77-4.80 (2H, overlapped with HDO, H5' and H5" m^7G), 4.73 (1H, dd, $J_{1'-2'}$ = 6.0, $J_{2'-3'}$ = 5.2, H2' G), 4.64 (1H, dd, $J_{1'-2'}$ = 2.5, $J_{2'-3'}$ = 5.2, H2' m^7G), 4.47 (1H, dd, $J_{2'-3'}$ = 5.2, $J_{3'-4'}$ = 3.7, H3' G or m^7G), 4.41-4.45 (1H, m, H4' G or m^7G), 4.38 (1H, dd, $J_{2'-3'}$ = 5.2, $J_{3'-4'}$ = 7.2, H3' G or m^7G), 4.29-4.31 (1H, m, H4' G or m^7G), 4.14-4.17 (2H, m, H5' and H5'' G), 4.10 (3H, s, m^7), 4.08 (1H, dd, $J_{CH2'-CH2''}$ = 15.4, $J_{CH2'-P\beta}$ = 9.7, $H_{CH2'}$), 4.01 (1H, dd, $J_{CH2'-CH2''}$ = 15.4, $J_{CH2''-P\beta}$ = 10.0, $H_{CH2''}$); ^{31}P NMR (162 MHz, D_2O, 25°C) δ_P: -2.53) (1P, dt, $J_{CH2-P\beta}$ = 10.0, $J_{P\alpha-P\beta}$ = 22.2, Pβ), -11.03 (1P, br d, $J_{P\alpha-P\beta}$ = 22.2, Pα); HRMS (-) ESI *m/z* found: 801.1626, calc. for $C_{24}H_{31}N_{14}O_{14}P_2^-$: 801.1619.

(6b) m^7G-triazole-NHCH$_2$pppG

Obtained according to GP F from **15b** (1000 mOD, 0.083 mmol, 124 mM) and **16b** (0.249 mmol, 373 mM) while stirring with $CuSO_4$ (8.3 mg, 0.033 mmol, 25 mM) and sodium ascorbate (13.1 mg, 0.066 mmol, 50 mM) in 0.667 mL of DMF/H_2O (1.7:1, v/v) for 6 h. After quenching the reaction with Na_2EDTA (122.9 mg, 0.33 mmol), the product was subjected to RP HPLC purification which afforded **6b** as ammonium salt.

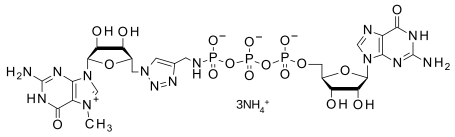

^{1}H NMR (400 MHz, D_2O, 25 °C) δ_H: 8.03 (1H, s, H8 G or $H_{triazole}$), 7.93 (1H, s, H8 G or $H_{triazole}$), 5.90 (1H, d, $J_{1'-2'}$ = 2.7, H1' m^7G), 5.82 (1H, d, $J_{1'-2'}$ = 5.9, H1' G), 4.77-4.84 (2H, m, overlapped with HDO, 5' and 5" m^7G), 4.74 (1H, dd, $J_{1'-2'}$ = 5.9, $J_{2'-3'}$ = 5.1, H2' G), 4.60 (1H, dd, $J_{1'-2'}$ = 2.7, $J_{2'-3'}$ = 5.1, H2' m^7G), 4.51 (1H, dd, $J_{2'-3'}$ = 5.1, $J_{3'-4'}$ = 3.9, H3' G), 4.44-4.47 (1H, m, H4' m^7G), 4.40 (1H, dd, $J_{2'-3'}$ = 5.1, $J_{3'-4'}$ = 7.4, H3' m^7G), 4.30-4.32 (1H, m, H4' G), 4.18-4.28 (1H, m, H5" and H5" G), 4.06-4.18 (1H, m, H_{CH2}), 4.08 (3H, s, m^7); ^{31}P NMR (162 MHz, D_2O, 25°C) δ_P: -1.59 (1P, dt, $J_{P\gamma-P\beta}$ = 22.0, $J_{P\gamma-CH2}$ = 10.3, Pγ), -10.49 (1P, ddd, $J_{P\alpha-P\beta}$ = 19.1, $J_{P\alpha-5'}$ = 4.4, $J_{P\alpha-5''}$ = 5.9, Pα), -21.94 (1P, dd, $J_{P\gamma-P\beta}$ = 22.0, $J_{P\alpha-P\beta}$ = 19.1, Pβ); HRMS (-) ESI *m/z* found: 881.1301, calc. for $C_{24}H_{32}N_{14}O_{17}P_3^-$: 881.1283; hydrolysis in D_2O: 19%.

(6c) m^7GppNHCH$_2$-triazole-G

Obtained according to GP F from **15c** (1026 mOD, 0.090 mmol, 208 mM) and **16a** (83.2 mg, 0.270 mmol, 625 mM) while stirring with $CuSO_4$ (9.0 mg, 0.036 mmol, 83 mM) and sodium ascorbate (14.3 mg, 0.072 mmol, 166 mM) in 0.432 mL of DMF/H_2O (1:2.3, v/v) for 2.5 h. After quenching the reaction with Na_2EDTA (134.1 mg, 0.36 mmol), the product was subjected to RP HPLC purification which afforded **6c** as ammonium salt.

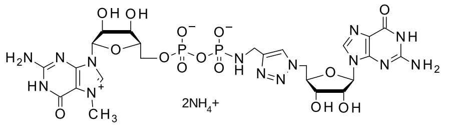

^{1}H NMR (400 MHz, D_2O, 25 °C) δ_H: 7.82 (1H, s, H8 G or $H_{triazole}$), 7.65 (1H, s, H8 G or $H_{triazole}$), 6.00 (1H, d, $J_{1'-2'}$ = 3.5, H1' m^7G or G), 5.82 (1H, d, $J_{1'-2'}$ = 3.2, H1' m^7G or G), 4.79 (overlapped with HDO, H5' and H5" G), 4.63-4.66 (2H, overlapped H2' G and H2' m^7G), 4.42-4.51 (3H, overlapped H3' m^7G, H3' G and H4' G or m^7G), 4.34-4.37 (1H, m, H4' G or m^7G), 4.26 (1H, ddd, $J_{5'-5''}$ = 11.8, J = 2.5, 4.4, H5' m^7G), 4.16 (1H, ddd, $J_{5'-5''}$ = 11.8, J = 3.0, 5.2, H5'' m^7G), 4.11 (1H, dd, $J_{CH2'-CH2''}$ = 15.4, $J_{CH2'-P\beta}$ = 11.2, $H_{CH2'}$), 4.04 (1H, dd, $J_{CH2'-CH2''}$ = 15.4, $J_{CH2''-P\beta}$ = 10.7, $H_{CH2''}$), 4.06 (3H, s, m^7); ^{31}P NMR (162 MHz, D_2O, 25°C) δ_P: -2.24 (1P, dt, $J_{CH2-P\beta}$ = 11.0, $J_{P\beta-P\gamma}$ = 22.0, Pβ), 11.00 (1P, d, $J_{P\beta-P\gamma}$ = 22.0, Pγ); HRMS (-) ESI *m/z* found: 801.1618, calc. for $C_{24}H_{31}N_{14}O_{14}P_2^-$: 801.1619.

(6d) m^7GpppNHCH$_2$-triazole-G

Obtained according to GP F from **15d** (800 mOD, 0.070 mmol, 92 mM) and **16a** (64.7 mg, 0.210 mmol, 277 mM) while stirring with $CuSO_4$ (7.0 mg, 0.028 mmol, 37 mM) and sodium ascorbate (11.1 mg, 0.056 mmol, 74 mM) in 0.760 mL of DMF/H_2O (1:1.6, v/v) for 2.5 h. After quenching the reaction with Na_2EDTA (104.3 mg, 0.28 mmol), the product was subjected to RP HPLC purification which afforded **6d** as ammonium salt.

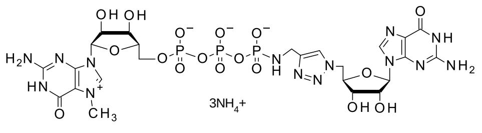

^1H NMR (400 MHz, D_2O, 25 °C) δ_H: 7.91 (1H, s, H8 G or $H_{triazole}$), 7.71 (1H, s, H8 G or $H_{triazole}$), 6.00 (1H, d, $J_{1'-2'}$ = 3.5, H1′ m^7G or G), 5.82 (1H, d, $J_{1'-2'}$ = 3.1, H1′ m^7G or G), 4.79 (2H, overlapped with HDO, H5′ and H5′′ G), 4.67-4.70 (2H, overlapped H2′ G and H2′ m^7G), 4.53-4.56 (2H, overlapped H3′ G and H3′ m^7G), 4.41-4.45 (2H, m, H4′ G), 4.34-4.39 (2H, overlapped H4′ m^7G and H5′ m^7G), 4.25 (1H, ddd, $J_{5'-5''}$ = 11.9, J = 5.7, 2.2, H5′′ m^7G), 4.05-4.18 (2H, m, H_{CH2}), 4.07 (3H, s, m^7); ^{31}P NMR (162 MHz, D_2O, 25°C) δ_P: -1.59 (1P, dt, $J_{P\beta-P\gamma}$ = 20.5, $J_{P\beta-CH2}$ = 10.3, Pβ), -10.55 (1P, d, $J_{P\gamma-P\delta}$ = 19.1, Pδ), -21.86 (1P, dd, $J_{P\gamma-P\delta}$ = 19.1, $J_{P\beta-P\gamma}$ = 20.5, Pγ); HRMS (-) ESI *m/z* found: 881.1300, calc. for $C_{24}H_{32}N_{14}O_{17}P_3^-$: 881.1283; hydrolysis in D_2O: 15%.

(7a) $m^7GpNHC_2H_4$-triazole-CH_2ppG

Obtained according to GP G from **11a** (848 mOD, 0.070 mmol, 150 mM) and **17c** (800 mOD, 0.070 mmol, 150 mM) while stirring with $CuSO_4$ (3.5 mg, 0.014 mmol, 30 mM) and sodium ascorbate (5.5 mg, 0.028 mmol, 60 mM) in 0.701 mL of H_2O for 1 h. After quenching the reaction with Na_2EDTA (52.2 mg, 0.14 mmol), the product was subjected to RP HPLC purification which afforded **7a** as ammonium salt.

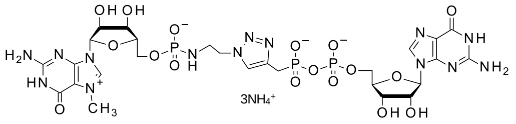

^1H NMR (400 MHz, D_2O, 25 °C) δ_H: 8.03 (1H, s, H8 G), 7.84 (1H, s, J = 2.0, $H_{triazole}$), 5.98 (1H, d, $J_{1'-2'}$ = 3.9, H1′ m^7G), 5.88 (1H, d, $J_{1'-2'}$ = 5.9, H1′ G), 4.77 (1H, dd, $J_{1'-2'}$ = 5.9, $J_{2'-3'}$ = 5.1, H2′ G), 4.69 (1H, dd, $J_{1'-2'}$ = 3.9, $J_{2'-3'}$ = 5.1, H2′ m^7G), 4.50 (1H, dd, $J_{2'-3'}$ = 5.1, $J_{3'-4'}$ = 3.9, H3′ G), 4.42 (1H, dd, $J_{2'-3'}$ = 5.1, $J_{3'-4'}$ = 5.5, H3′ m^7G), 4.37 (2H, br t, $J_{CH2(triazole)m7G-CH2(NH)}$ = 6.1, $H_{CH2(triazole)m7G}$), 4.31-4.34 (2H, m, overlapped H4′ G and m^7G), 4.14-4.21 (2H, m, H5′ and H5″ G), 4.00 (1H, ddd, $J_{5'-5''}$ = 11.7, J = 4.7, 2.7, H5′ m^7G), 3.90 (1H, ddd, $J_{5'-5''}$ = 11.7, J = 4.9, 3.3, H5″ m^7G), 3.23-3.28 (2H, m, $H_{CH2(NH)}$), 3.16 (2H, d, $J_{P\beta-CH2(P)}$ = 20.2, $H_{CH2(P)}$); ^{31}P NMR (162 MHz, D_2O, 25°C) δ_P: 11.70 (1P, dt, $J_{P\beta-CH2(P)}$ = 20.2, $J_{P\alpha-P\beta}$ = 26.4, Pβ), 9.01-9.14 (1P, m, Pδ), -10.40 (1P, br d, $J_{P\alpha-P\beta}$ = 26.4, Pα); HRMS (-) ESI *m/z* found: 909.1618, calc. for $C_{26}H_{36}N_{14}O_{17}P_3^-$: 909.1596; hydrolysis in D_2O: 11%.

(7b) $m^7GpNHC_2H_4$-triazole-CH_2pppG

Obtained according to GP G from **11b** (742 mOD, 0.061 mmol, 150 mM) and **17c** (700 mmol, 0.061 mmol, 150 mM) while stirring with $CuSO_4$ (3.0 mg, 0.012 mmol, 30 mM) and sodium ascorbate (4.8 mg, 0.024 mmol, 60 mM) in 0.409 mL of H_2O for 1 h. After quenching the reaction with Na_2EDTA (44.7 mg, 0.12 mmol), the product was subjected to RP HPLC purification which afforded **7b** as ammonium salt.

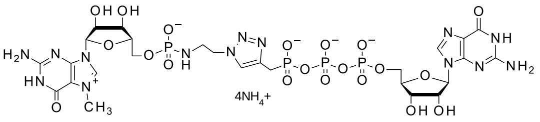

^1H NMR (400 MHz, D_2O, 25 °C) δH: 8.06 (1H, s, H8 G), 7.88 (1H, d, J = 2.0, $H_{triazole}$), 5.98 (1H, d, $J_{1'-2'}$ = 3.9, H1′ m^7G), 5.86 (1H, d, $J_{1'-2'}$ = 6.3, H1′ G), 4.79 (1H, dd, $J_{1'-2'}$ = 6.3, $J_{2'-3}$ = 5.1, H2′ G), 4.74 (1H, dd, $J_{1'-2'}$ = 3.9, $J_{2'-3'}$ = 5.1, H2′ m^7G), 4.54 (1H, dd, $J_{2'-3'}$ = 5.1, $J_{3'-4'}$ = 3.5, H3′ G), 4.37 (1H, dd, $J_{2'-3}$ = 5.1, $J_{2'-3}$ = 5.1, H3′ m^7G), 4.37 (2H, br t, $J_{CH2(triazole)m7G-CH2(NH)}$ = 6.6, $H_{CH2(triazole)m7G}$), 4.32-4.35 (2H, overlapped H4′ G and H4′ m^7G and H5′ G), 4.28 (1H, ddd, $J_{5'-5''}$ = 11.7, J = 5.3, 3.3, H5′ m^7G), 4.23 (1H, ddd, $J_{5'-5''}$ = 11.7, J = 5.9, 3.9, H5″ m^7G), 4.06 (3H, s, m^7), 4.02 (1H, ddd, $J_{5'-5''}$ = 11.7, J = 4.7, 2.7, H5′ G), 3.86 (1H, ddd, $J_{5'-5''}$ = 11.7, J = 5.1, 3.5, H5′ G), 3.21-3.26 (2H, m, $H_{CH2(NH)}$), 3.22 (2H, d, $J_{CH2(P)-P\gamma}$ = 20.4, $H_{CH2(P)}$); ^{31}P NMR (162 MHz, D_2O, 25°C) δ_P: 14.20 (1P, dt, $J_{P\beta-P\gamma}$ = 24.9, $J_{P\gamma-CH2(P)}$ = 20.4, Pγ), 11.20-11.32 (1P, m, Pε), -8.35 (1P, dt, $J_{P\alpha-P\beta}$ = 19.1 $J_{P\alpha-5'/5''}$ = 4.4 , Pα), 20.05 (1P, dd, $J_{P\alpha-P\beta}$ = 19.1, $J_{P\beta-P\gamma}$ = 24.9, Pβ); HRMS (-) ESI *m/z* found: 989.1276, calc. for $C_{26}H_{37}N_{14}O_{20}P_4^-$: 989.1259; hydrolysis in D_2O: 7%.

(7c) m^7GppCH_2-triazole-C_2H_4NHpG

Obtained according to GP G from **11c** (1132 mOD, 0.099 mmol, 150 mM) and **17a** (1200 mOD, 0.099 mmol, 150 mM) while stirring with $CuSO_4$ (4.9 mg, 0.020 mmol, 30 mM) and sodium ascorbate (7.9 mg, 0.040 mmol, 60 mM) in 0.662 mL of H_2O for 2 h. After quenching the reaction with Na_2EDTA (74.5 mg, 0.20 mmol), the product was subjected to RP HPLC purification which afforded **7c** as ammonium salt.

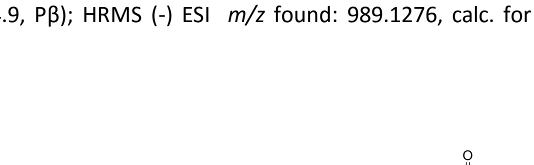

^1H NMR (400 MHz, D_2O, 25 °C) δ_H: 8.01 (1H, s, H8 G), 7.82 (1H, d, J = 2.0, $H_{triazole}$), 6.02 (1H, d, $J_{1'-2'}$ = 3.5, H1′ m^7G), 5.86 (1H, d, $J_{1'-2'}$ = 5.5, H1′ G), 4.83 (1H, dd, $J_{1'-2'}$ = 5.5, $J_{2'-3'}$ = 5.5, H2′G), 4.68 (1H, dd, $J_{1'-2'}$ = 3.5, $J_{2'-3'}$ = 4.7, H2′ m^7G), 4.46 (2H, overlapped H3′ G and m^7G), 4.35-4.37 (1H, m, H4′ G or m^7G), 4.28-4.31 (3H, overlapped H4′ G or m^7G, $H_{CH2(triazole)G'}$), 4.24 (1H, ddd, $J_{5'-5''}$ = 11.7, J = 4.3, 2.4, H5′ G or m^7G), 4.16 (1H, ddd, $J_{5'-5''}$ = 11.7, J = 5.5, 2.4, H5″ G or m^7G), 4.08 (3H, s, m^7), 3.96 (1H, ddd, $J_{5'-5''}$ = 11.7, J = 4.7, 3.1, H5″ G or m^7G), 3.88-3.93 (1H, m, H5″ G or m^7G), 3.22 (2H, d, $J_{CH2(P)-P\gamma}$ = 20.5, $H_{CH2(P)}$), 3.13-3.20 (2H, m, $H_{CH2(NH)}$); ^{31}P NMR (162 MHz, D_2O, 25°C) δ_P: 11.95 (1P, dt, $J_{P\gamma-CH2(P)}$ = 20.5, $J_{P\gamma-P\delta}$ = 26.4, Pγ), 9.05-9.18 (1P, m, Pα), -10.34 (1P, br d, $J_{P\alpha-P\beta}$ = 26.4, Pδ); HRMS (-) ESI *m/z* found: 909.1603, calc. for $C_{26}H_{36}N_{14}O_{17}P_3^-$: 909.1596.

(7d) $m^7GpppCH_2$-triazole-C_2H_4NHpG

Obtained according to GP G from **11d** (700 mOD, 0.061 mmol, 100 mM) and **17a** (742 mOD, 0.061 mmol, 100 mM) while stirring with $CuSO_4$ (6.0 mg, 0.024 mmol, 40 mM) and sodium ascorbate (9.6 mg, 0.048 mmol, 80 mM) in 0.614 mL of H_2O for 24 h. After quenching the reaction with Na_2EDTA (89.4 mg, 0.24 mmol), the product was subjected to RP HPLC purification which afforded **7d** as ammonium salt.

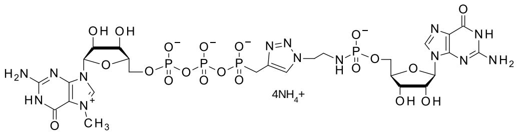

^1H NMR (400 MHz, D_2O, 25 °C) δ_H: 8.00 (1H, s, H8 G), 7.84 (1H, d, $J_{CH2(P)\text{-}H(triazole)}$ = 2.2, $H_{triazole}$), 6.00 (1H, d, $J_{1'\text{-}2'}$ = 3.5, H1′ m^7G), 5.86 (1H, d, $J_{1'\text{-}2'}$ = 5.5, H1′ G), 4.89 (1H, dd, $J_{1'\text{-}2'}$ = 5.5, $J_{2'\text{-}3}$ = 5.1, H2′ G), 4.69 (1H, dd, $J_{1'\text{-}2'}$ = 3.5, $J_{2'\text{-}3'}$ = 5.1, H2′ m^7G), 4.56 (1H, dd, $J_{2'\text{-}3'}$ = 5.1, $J_{3'\text{-}4'}$ = 5.5, H3′ m^7G), 4.47 (1H, dd, $J_{2'\text{-}3}$ = 5.1, $J_{2'\text{-}3}$ = 4.3, H3′ G), 4.36-4.41 (2H, overlapped H4′ and H5′ m^7G), 4.23-4.30 (4H, overlapped H5″ m^7G, H4′G, $H_{CH2(triazole)G}$), 4.08 (3H, s, m^7), 3.98 (1H, ddd, $J_{5'\text{-}5''}$ = 11.7, J = 4.3, 2.9, H5′G), 3.89-3.95 (1H, m, H5″ G), 3.26 (2H, dd, $J_{CH2\text{-}P\gamma}$ = 20.5, $J_{CH2(P)\text{-}H(triazole)}$ = 2.2, $H_{CH2(P)}$), 3.07-3.18 (2H, m, $H_{CH2(NH)}$); ^{31}P NMR (162 MHz, D_2O, 25°C) δ_P: 14.11 (1H, dt, $J_{P\gamma\text{-}P\delta}$ = 24.9, $J_{CH2(P)\text{-}P\gamma}$ = 20.5, Pγ), 11.18-11.37 (1P, m, Pα), -8.40 (1P, br d, $J_{P\varepsilon\text{-}P\delta}$ = 19.1, Pε), -19.96 (1P, dd, $J_{P\varepsilon\text{-}P\delta}$ = 19.1, $J_{P\gamma\text{-}P\delta}$ = 24.9, Pδ); HRMS (-) ESI *m/z* found: 494.0598, calc. for $C_{26}H_{36}N_{14}O_{20}P_4^{2-}$: 494.0590; hydrolysis in D_2O: 9%.

(8a) $m^7GpNHC_2H_4$-triazole-ppG

Obtained according to GP G from **12a** (742 mOD, 0.061 mmol, 100 mM) and **17c** (700 mOD, 0.061 mmol, 100 mM) while stirring with $CuSO_4$ (6.0 mg, 0.024 mmol, 40 mM) and sodium ascorbate (9.6 mg, 0.048 mmol, 80 mM) in 0.614 mL of H_2O for 1 h. After quenching the reaction with Na_2EDTA (89.4 mg, 0.24 mmol), the product was subjected to RP HPLC purification which afforded **8a** as ammonium salt.

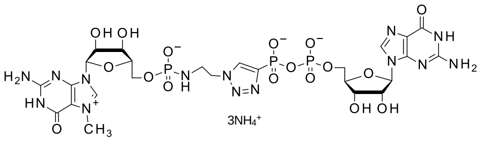

^1H NMR (400 MHz, D_2O, 25 °C) δ_H: 8.25 (1H, s, H8 G or $H_{triazole}$), 7.94 (1H, s, H8 G or $H_{triazole}$), 5.98 (1H, d, $J_{1'\text{-}2'}$ = 3.9, H1′ m^7G), 5.83 (1H, d, $J_{1'\text{-}2'}$ = 5.9, H1′ G), 4.66 (1H, dd, $J_{1'\text{-}2'}$ = 5.9, $J_{2'\text{-}3}$ = 5.1, H2′ G), 4.62 (1H, dd, $J_{1'\text{-}2'}$ = 3.9, $J_{2'\text{-}3'}$ = 5.1, H2′ m^7G), 4.42-4.46 (3H, overlapped $H_{CH2(triazole)m7G'}$ and H3′G), 4.37 (1H, dd, $J_{2'\text{-}3'}$ = 5.1, $J_{3'\text{-}4'}$ = 5.1, H3′ m^7G), 4.29-4.32 (1H, m, H4′ m^7G), 4.25-4.28 (1H, m, H4′ G), 4.13-4.15 (2H, m, H5′ and H5″ G), 4.06 (3H, s, m^7), 3.92 (1H, ddd, $J_{5'\text{-}5''}$ = 11.7, J = 4.7, 2.7, H5′ m^7G), 3.82 (1H, ddd, $J_{5'\text{-}5''}$ = 11.7, J = 5.1, 3.1, H5′ m^7G), 3.25-3.31 (2H, m, $H_{CH2(NH)}$); ^{31}P NMR (162 MHz, D_2O, 25°C) δ_P: 8.92-9.04 (1P, m, Pδ), -6.87 (1P, d, $J_{P\alpha\text{-}P\beta}$ = 22.7, Pβ), -10.80 (1P, dt, $J_{P\alpha\text{-}P\beta}$ = 22.7, $J_{P\alpha\text{-}5'/5'''}$ = 6.6, Pα); HRMS (-) ESI *m/z* found: 895.1456, calc. for $C_{25}H_{34}N_{14}O_{17}P_3^-$: 895.1439; hydrolysis in D_2O: 14%.

(8b) $m^7GpNHC_2H_4$-triazole-pppG

Obtained according to GP G from **12b** (742 mOD, 0.061 mmol, 100 mM) and **17c** (700 mOD, 0.061 mmol, 100 mM) while stirring with $CuSO_4$ (6.0 mg, 0.024 mmol, 40 mM) and sodium ascorbate (9.6 mg, 0.048 mmol, 80 mM) in 0.614 mL of H_2O for 2.5 h. After quenching the reaction with Na_2EDTA (89.4 mg, 0.24 mmol), the product was subjected to RP HPLC purification which afforded **8b** as ammonium salt.

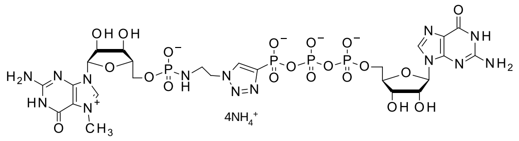

^1H NMR (400 MHz, D_2O, 25 °C) δ_H: 8.30 (1H, s, H8 G or $H_{triazole}$), 7.03 (1H, s, H8 G or $H_{triazole}$), 5.98 (1H, d, $J_{1'\text{-}2'}$ = 3.7, H1′ m^7G), 5.85 (1H, d, $J_{1'\text{-}2'}$ = 6.3, H1′ G), 4.73 (1H, dd, $J_{1'\text{-}2'}$ = 6.3, $J_{2'\text{-}3}$ = 5.1, H2′ G), 4.66 (1H, dd, $J_{1'\text{-}2'}$ = 3.7, $J_{2'\text{-}3'}$ = 4.7, H2′ m^7G), 4.48 (1H, dd, $J_{2'\text{-}3'}$ = 5.1, $J_{3'\text{-}4'}$ = 3.5, H3′ G), 4.45 (2H, br t, $J_{CH2(triazole)m7G\text{-}CH2(NH)}$ = 5.9, $H_{CH2(triazole)m7G}$), 4.38 (1H, dd, $J_{2'\text{-}3'}$ = 4.7, $J_{3'\text{-}4'}$ = 5.9, H3′ m^7G), 4.31-4.34 (1H, m, H4′ m^7G), 4.27-4.30 (1H, m, H4′ G), 4.14-4.16 (2H, m, H5′ and H5″ G), 4.06 (3H, s, m^7), 3.96 (1H, ddd, $J_{5'\text{-}5''}$ = 11.7, J = 4.7, 2.7, H5′ m^7G), 3.84 (1H, ddd, $J_{5'\text{-}5''}$ = 11.7, J = 5.1, 3.5, H5′ m^7G), 3.29 (1H, dt, $J_{CH2(triazole)\text{-}CH2(NH)}$ =5.9, $J_{CH2(NH)\text{-}P\varepsilon}$ = 10.6, $H_{CH2(NH)'}$ and $H_{CH2(NH)''}$); ^{31}P NMR (162 MHz, D_2O, 25°C) δ_P: 8.94-9.12 (1P, m, Pε), -6.56 (1P, d, $J_{P\beta\text{-}P\gamma}$ = 22.0, Pγ), -10.61 (1P, dt, $J_{P\alpha\text{-}P\beta}$ = 19.1, $J_{P\alpha\text{-}5'/5''}$ = 5.1, Pα), -22.49 (1p, dd, $J_{P\alpha\text{-}P\beta}$ = 22.7, $J_{P\alpha\text{-}P\beta}$ = 19.1, Pβ); HRMS (-) ESI *m/z* found: 975.1124, calc. for $C_{25}H_{35}N_{14}O_{20}P_4^-$: 975.1103; hydrolysis in D_2O: 9%.

(8c) m^7Gpp-triazole-C_2H_4NHpG

Obtained according to GP G from **12c** (700 mOD, 0.061 mmol, 100 mM) and **17a** (742 mOD, 0.061 mmol, 100 mM) while stirring with $CuSO_4$ (6.0 mg, 0.024 mmol, 40 mM) and sodium ascorbate (9.6 mg, 0.048 mmol, 80 mM) in 0.614 mL of H_2O for 2 h. After quenching the reaction with Na_2EDTA (89.4 mg, 0.24 mmol), the product was subjected to RP HPLC purification which afforded **8c** as ammonium salt.

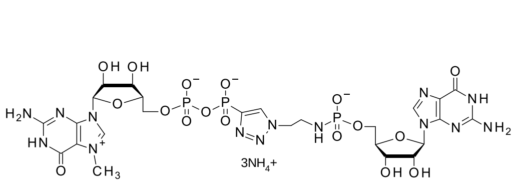

^1H NMR (400 MHz, D_2O, 25 °C) δ_H: 8.24 (1H, s, H8 G or $H_{triazole}$), 8.03 (1H, s, H8 G or $H_{triazole}$), 5.99 (1H, d, $J_{1'\text{-}2'}$ = 3.3, H1′ m^7G), 5.84 (1H, d, $J_{1'\text{-}2'}$ = 5.9, H1′ G), 4.75 (1H, dd, $J_{1'\text{-}2'}$ = 5.9, $J_{2'\text{-}3}$ = 5.1, H2′ G), 4.64 (1H, dd, $J_{1'\text{-}2'}$ = 3.3, $J_{2'\text{-}3'}$ = 4.7, H2′ m^7G), 4.41-4.45 (2H, overlapped with H3′ G and H3′ m^7G), 4.39 (2H, br t, $J_{CH2(triazole)G\text{-}CH2(NH)}$ = 6.3, $H_{CH2(triazole)G}$), 4.34-4.36 (1H, m, H4′ m^7G), 4.24-4.29 (2H, m, overlapped

H5′ m^{7}G and H4′ G), 4.18 (1H, ddd, $J_{5'\text{-}5''}$ = 11.9, J = 5.5, 2.3, H5′′ m^{7}G), 4.04 (3H, s, m^7), 3.90 (1H, ddd, $J = J_{5'\text{-}5''}$ = 11.7, J = 4.5, 2.9, H5′ G), 3.77-3.83 (1H, m, H5″ G), 3.17-3.28 (2H, m, $H_{CH2(NH)}$); ^{31}P NMR (162 MHz, D_2O, 25°C) δ_P: 8.93-9.1 (1P, m, Pα), -6.62 (1P, d, $J_{P\gamma\text{-}P\delta}$ = 23.5, Pγ), -10.75 (1P, br d, $J_{P\gamma\text{-}P\delta}$ = 23.5, Pδ); HRMS (-) ESI *m/z* found: 895.1454, calc. for $C_{25}H_{34}N_{14}O_{17}P_3^-$: 895.1439.

(8d) m^{7}Gppp-triazole-C$_2$H$_4$NHpG

Obtained according to GP G from **12d** (700 mOD, 0.061 mmol, 50 mM) and **17a** (742 mOD, 0.061 mmol, 50 mM) while stirring with $CuSO_4$ (6.0 mg, 0.024 mmol, 20 mM) and sodium ascorbate (9.6 mg, 0.048 mmol, 40 mM) in 1.228 mL of H_2O for 1 h. After quenching the reaction with Na_2EDTA (89.4 mg, 0.24 mmol), the product was subjected to RP HPLC purification which afforded **8d** as ammonium salt.

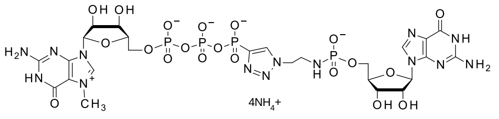

^{1}H NMR (400 MHz, D_2O, 25 °C) δ_H: 8.23 (1H, s, H8 G or $H_{triazole}$), 8.02 (1H, s, H8 G or $H_{triazole}$), 6.00 (1H, d, $J_{1'\text{-}2'}$ = 3.5, H1′ m^{7}G), 5.86 (1H, d, $J_{1'\text{-}2'}$ = 5.5, H1′ G), 4.84 (1H, dd, $J_{1'\text{-}2'}$ = 5.5, $J_{2'\text{-}3}$ = 5.1, H2′ G), 4.66 (1H, dd, $J_{1'\text{-}2'}$ = 3.5, $J_{2'\text{-}3'}$ = 4.7, H2′ m^{7}G), 4.51 (1H, dd, $J_{2'\text{-}3'}$ = 4.7, $J_{3'\text{-}4'}$ = 5.5, H3′ m^{7}G), 4.44 (1H, dd, $J_{2'\text{-}3}$ = 5.1, $J_{2'\text{-}3}$ = 3.9, H3′ G), 4.27-4.37 (5H, overlapped H4′ G and H4′ m^{7}G and H5′ G or m^{7}G, $H_{CH2(triazole)}$), 4.20 (1H, ddd, $J_{5'\text{-}5''}$ = 11.7, J = 5.5, 2.4, H5′′ G or m^{7}G), 4.07 (3H, s, m^7), 3.94 (1H, ddd, $J_{5'\text{-}5''}$ = 11.5, J = 4.3, 3.1, H5′ m^{7}G or G), 3.84-3.89 (1H, m, H5′ m^{7}G or G), 3.13-3.24 (2H, m, $H_{CH2(NH)}$); ^{31}P NMR (162 MHz, D_2O, 25°C) δ_P: 11.0.8-11.21 (1P, m, Pα), -4.38 (1P, d, $J_{P\beta\text{-}P\gamma}$ = 22.0, Pγ), -8.41 (1P, br d, $J_{P\alpha\text{-}P\beta}$ = 19.1, Pε), -20.26 (1P, dd, $J_{P\alpha\text{-}P\beta}$ = 22.0, $J_{P\alpha\text{-}P\beta}$ = 19.1, Pγ); HRMS (-) ESI *m/z* found: 975.1122, calc. for $C_{25}H_{35}N_{14}O_{20}P_4^-$: 975.1103; hydrolysis in D_2O: 13%.

(8e) m$_2^{7,2'\text{-}O}$Gppp-triazole-C$_2$H$_4$NHpG

Obtained according to GP G from **12f** (650 mOD, 0.057 mmol, 100 mM) and **17a** (689 mOD, 0.061 mmol, 50 mM) while stirring with $CuSO_4{\cdot}5H_2O$ (2.7 mg, 0.011 mmol, 20 mM) and sodium ascorbate (4.4 mg, 0.022 mmol, 40 mM) in 0.570 mL of H_2O:*t*-BuOH (2:1,v/v) for 2 h. After quenching the reaction with Na_2EDTA (89.4 mg, 0.24 mmol), the product was subjected to RP HPLC purification which afforded **8e** as ammonium salt.

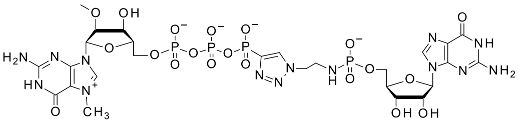

^{1}H NMR (400 MHz, D_2O, 25 °C) δ_H: 9.14 (1H, q, $J_{H8\text{-}m7}$ = 0.8, H8 m^{7}G), 8.24 (1H, s, H8 G or $H_{triazole}$), 8.02 (1H, s, H8 G or $H_{triazole}$), 6.06 (1H, d, $J_{1'\text{-}2'}$ = 2.7, H1′ m^{7}G), 5.84 (1H, d, $J_{1'\text{-}2'}$ = 5.5, H1′ G), 4.80 (1H, overlapped with HDO, H2′ G), 4.58 (1H, dd, J = 6.2, 4.7, H3′ m^{7}G), 4.42 (1H, dd, J = 5.1, 3.9, H3′ G), 4.27-4.36 (6H, overlapped H2′m^{7}G, H4′ G and m^{7}G, H5′ G or m^{7}G, $H_{CH2(triazole)}$), 4.18 (1H, ddd, $J_{5'\text{-}5''}$ = 12.3, J = 5.3, 2.7, H5′′ G or m^{7}G), 4.06 (3H, d, $J_{H8\text{-}m7}$ = 0.8, m^7), 3.92 (1H, ddd, $J_{5'\text{-}5''}$ = 11.7, J = 4.3, 3.1, H5′ G or m^{7}G), 3.84 (1H, ddd, $J_{5'\text{-}5''}$ = 11.7, J = 5.1, 4.7), 3.59 (3H, s, m$^{2'O}$), 3.12-3.26 (2H, m, $H_{CH2(NH)}$); ^{31}P NMR (162 MHz, D_2O, 25°C) δ_P: 8.95-9.15 (1P, m, Pα), -6.57 (1P, d, $J_{P\gamma\text{-}P\delta}$ = 21.3, Pγ), -10.64 (1P, ddd, $J_{P\varepsilon\text{-}P\delta}$ = 19.1, $J_{P\varepsilon\text{-}5'}$ = 4.4, $J_{P\varepsilon\text{-}5''}$ = 2.9, Pε), -22.42 (1P, dd, $J_{P\gamma\text{-}P\delta}$ = 21.3, $J_{P\varepsilon\text{-}P\delta}$ = 19.1, Pδ); HRMS (-) ESI *m/z* found: 989.1272, calc. for $C_{26}H_{37}N_{14}O_{20}P_4^-$: 989.1259; hydrolysis in D_2O: 8%.

(9a) m^{7}GppNHC$_2$H$_4$-triazole-C$_2$H$_4$ppG

Obtained according to GP G from **10a** (580 mOD, 0.048 mmol, 100 mM) and **17d** (437 mOD, 0.038 mmol, 80 mM) while stirring with $CuSO_4$ (4.8 mg, 0.019 mmol, 40 M) and sodium ascorbate (7.5 mg, 0.038 mmol, 80 mM) in 0.485 mL of H_2O for 0.5 h. After quenching the reaction with Na_2EDTA (0.7 mg, 0.19 mmol), the product was subjected to RP HPLC purification which afforded **9a** as ammonium salt.

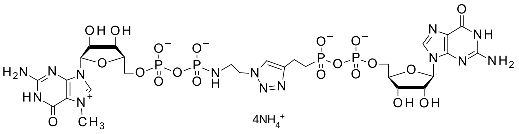

^{1}H NMR (400 MHz, D_2O, 25 °C) δ_H: 9.16 (1H, s, H8 m^{7}G), 8.06 (1H, s, H8 G or $H_{triazole}$), 7.71 (1H, s, H8 G or $H_{triazole}$), 6.00 (1H, d, $J_{1'\text{-}2'}$ = 2.7, H1′ G or m^{7}G), 5.88 (1H, d, $J_{1'\text{-}2'}$ = 5.5, H1′ G or m^{7}G), 4.80 (1H, overlapped with HDO, H2′ G or m^{7}G), 4.69 (2H, dd, $J_{1'\text{-}2'}$ = 2.7, $J_{3'\text{-}4'}$ =5.1, H2′ G or m^{7}G), 4.49-4.54 (2H, overlapped H3′ G and m^{7}G), 4.35-4.39 (4H, overlapped H4′ G and m^{7}G, $H_{CH2(triazole)m7G}$), 4.18-4.33 (4H, overlapped H5′ and H5′′G and m^{7}G), 4.06 (3H, s, m^7), 3.30 (2H, dt, $J_{CH2(NH)\text{-}P\delta}$ = 11.9, $J_{CH2(NH)\text{-}CH2(triazole)m7G}$ = 6.5, $H_{CH2(NH)}$), 2.78 (2H, m, $H_{CH2(triazole)G}$ or $H_{CH2(P)}$), 1.99 (2H, m, $H_{CH2(triazole)G}$ or $H_{CH2(P)}$); ^{31}P NMR (162 MHz, D_2O, 25°C) δ_P: 17.61-18.01 (1P, m, Pβ), -1.54 (1P, dt, $J_{P\delta\text{-}P\varepsilon}$ = 21.3, $J_{P\delta\text{-}CH2(NH)}$ = 11.9, Pδ), -10.27 (1P, br d, $J_{P\delta\text{-}P\varepsilon}$ = 21.3, Pε), -10.52 (1P, br d, $J_{P\alpha\text{-}P\beta}$ = 27.9, Pα); HRMS (-) ESI *m/z* found: 1003.1435, calc. for $C_{27}H_{39}N_{14}O_{20}P_4^-$: 1003.1421; hydrolysis in D_2O: 11%.

(9b) m^{7}GppC$_2$H$_4$-triazole-C$_2$H$_4$NHppG

Obtained according to GP G from **10c** (375 mOD, 0.033 mmol, 100 mM) and **17b** (397 mOD, 0.033 mmol, 100 mM) while stirring with $CuSO_4$ (1.6 mg, 0.0066 mmol, 20 mM) and sodium ascorbate (2.6 mg, 0.013 mmol, 40 mM) in 0.328 mL of H_2O for 1 h. After quenching the reaction with Na_2EDTA (24.6 mg, 0.066 mmol), the product was subjected to RP HPLC purification which afforded **9b** as ammonium salt.

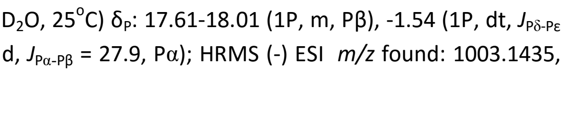

^{1}H NMR (400 MHz, D_2O, 25 °C) δ_H: 9.18 (1H, s, H8 m^{7}G), 8.12 (1H, s, H8 G or $H_{triazole}$), 7.69 (1H, s, H8 G or $H_{triazole}$), 6.02 (1H, d, $J_{1'\text{-}2'}$ = 3.5, H1′ G or m^{7}G), 5.88 (1H, d, $J_{1'\text{-}2'}$ = 5.5, H1′ G or m^{7}G), 4.72-4.76 (2H, overlapped H2′ G and m^{7}G), 4.50-4.54 (2H, overlapped H3′ G and

m^{7}G), 4.40-4.43 (1H, m, H4 G or m^{7}G), 4.33-4.39 (2H, overlapped H4' G or m^{7}G and H5' G or m^{7}G), 4.28 (2H, br t, $J_{CH2(triazole)G-CH2(NH)}$ = 6.6, $H_{CH2(triazole)G}$), 4.17-4.29 (3H, overlapped H5'' G and m^{7}G and H5' G or m^{7}G), 4.06 (3H, s, m^7), 3.20-3.28 (2H, m, $H_{CH2(NH)}$), 2.79-2.85 (2H, m, $H_{CH2(triazole)m7G)}$ or $H_{CH2(P)}$), 1.99-2.08 (2H, m, $H_{CH2(triazole)m7G)}$ or $H_{CH2(P)}$); ^{31}P NMR (162 MHz, D_2O, 25°C) δ_P: 17.61-18.10 (1P, m, Pδ), -1.64 (1P, dt, $J_{P\alpha-P\beta}$ = 22.0, $J_{P\beta-CH2(NH)}$ = 12.5, Pβ), -10.22 (1P, br d, $J_{P\alpha-P\beta}$ = 22.0, Pα), -10.50 (1P, br d, $J_{P\delta-P\varepsilon}$ = 24.9, Pε); HRMS (-) ESI *m/z* found: 1003.1444, calc. for $C_{27}H_{39}N_{14}O_{20}P_4^-$: 1003.1421; hydrolysis in D_2O: 10%.

2. Stability studies

Compounds **2d**, **3b**, **4d**, **5a**, **6a** and **9a** were incubated in sealed vials at 20 µM and at room temperature in four different buffers: 0.1 M formate buffer pH 3.0, 0.1 M acetate buffer pH 5.0, 0.1 M HEPES pH 7.0 and 0.1 M Tris-HCl pH 9.0. Samples of 50 µl for each condition were taken after 0, 2 and 24 h and analysed by RP HPLC. The starting compound peak area was determined for each time-point and % of remaining cap was calculated in reference to the time-point 0 h.

3. Biological studies

3.1 Determination of K_{AS} cap analogue-eIF4E complexes

Association constants for cap analogue-eIF4E complexes were determined using fluorescence quenching titration method as described previously.[10] Measurements were performed using a quartz cuvette with optical path length of 4 mm for absorption and 10 mm for emission on LS-55 spectrofluorometer Perkin-Elmer Co. (Norwalk, CT, USA). Titration experiments were carried out at 20 °C in 50 mM Hepes/KOH buffer, pH 7.20 containing 100 mM KCl, 0.5 mM EDTA, and 1 mM DTT. During the experiment 1 µl aliquots of ligand solution was added to the 1400 µl of 0.1 µM protein solution. Protein fluorescence was monitored at 337 or 345 nm (excitation 280 or 295 nm, respectively). For data analysis fluorescence intensity correction was applied for sample dilution and inner filter effect. Association constants were determined by fitting theoretical dependence of fluorescence intensity on total ligand concentration. The experiments were performed in 1-4 replicates, the association constants were calculated as weighted averages.

3.2 Incorporation of dinucleotide cap analogues at 5' end of transcripts

3.2.1 Post-transcriptional capping

In vitro transcription was performed on pJET1.2 vector linearized at XhoI site (50 ng/µl) with T7 RNA polymerase (New England BioLabs) (2.5 U/µl) in the presence of 40 mM Tris-HCl pH 8.0, 14 mM $MgCl_2$, 1 mM spermidine, 5 mM DTT, 0.01% Triton X-100, RiboLock RNase Inhibitor (ThermoFisher Scientific) (2.0 U/µl), 0.5 mM UTP, 0.5 mM ATP, 0.5 mM CTP, 0.125 mM GTP and 1.25 mM **10b**. The reaction mixture was incubated at 37 °C for 1h followed by addition of TURBO Dnase (Ambion) (0.1 U/µl) and incubation at 37 °C for another 30 min. The reaction was quenched by addition of 2 µl of 0.5 M EDTA pH 8.0 and purified with RNeasy MinElute Cleanup kit (Qiagen) to afford γ-C-(3-butynyl) 5'-triphosphate **RNA1**.

The reference 5'-triphosphate **RNA1** and 5' capped **RNA1** were obtained analogously but with usage of 0.5 mM GTP and no cap analogue or compound **1b** instead of **10b**, respectively.

To the aqueous solution of γ-C-(3-butynyl) 5'-triphosphate **RNA1** (10.5 pmol) and **16b** (0.525 nmol) the aqueous solution of $CuSO_4 \cdot 5H_2O$ (1.05 nmol), THPTA (2.1 nmol) and sodium ascorbate (2.1 nmol) was added. Final concentrations in reaction mixture were as follows: γ-C-(3-butynyl) 5'-triphosphate **RNA1** – 2.89 µM, **16b** – 144.6 µM, $CuSO_4 \cdot 5H_2O$ – 289.2 µM , THPTA – 578.5 µM, and sodium ascorbate – 578.5 µM. The mixture was incubated at 37°C for 1.5 h, then quenched by adding equal amount of loading dye (5 M urea, 44% formamide, 20 mM EDTA, 0.03% bromophenol blue, 0.03% xylene cyanol) and directly analyzed by 15% PAGE.

3.2.2 Synthesis of transcripts for capping efficiency determination

Prior to in vitro transcription annealing of a template was performed. **DNA1** and **DNA2** (20 µM each) were incubated in the presence of 4 mM Tris-HCl pH 8.0, 15 mM NaCl and 0.1 mM EDTA at 95 °C for 2 min and then at room temperature for 30 min. After 10-fold dilution the mixture was directly used for in vitro transcription.

In vitro transcription was performed on pre-annealed **DNA1** and **DNA2** (0.5 µM) with SP6 RNA polymerase (New England BioLabs) (1 U/µl) in the presence of 1x RNA Pol Reaction Buffer (New England BioLabs), 5 mM DTT, RiboLock RNase Inhibitor (ThermoFisher Scientific) (2.0 U/µl), 0.5 mM UTP, 0.5 mM ATP, 0.5 mM CTP, 0.125 mM GTP and 1.25 mM of appropriate triazole-modified dinucleotide cap analogue and reference GpppG, m^{7}GpppG and $m_2^{7,3'-O}$GpppG.[3] The reaction mixture was incubated at 40 °C for 1.5 h followed by addition of TURBO Dnase (Ambion) (0.1 U/µl) and incubation at 37 °C for another 30 min. The reaction was quenched by addition of 2 µl of 0.5 M EDTA pH 8.0 and purified with RNA Clean&Concentrator-5 kit (Zymo Research) to afford capped **RNA2** strands.

To reduce heterogeneity, each capped **RNA2** (11.42 ng/µl, ca. 1 µM) was incubated in presence of 50 mM Tris-HCl pH 8.0, 50 mM $MgCl_2$, RiboLock RNase Inhibitor (ThermoFisher Scientific) (2.0 U/µl) and **DNA3** (1 µM) at 37°C for 1 h to afford **RNA3**.[1] The reaction was stopped by freezing in liquid nitrogen and directly analyzed by 15% PAGE.

Relative bands intensity was determined using CLIQS v1.0 program.

3.3 Translation studies

3.3.1 Synthesis of transcripts

In vitro transcription was performed on PCR product coding *Firefly* luciferase under the control of SP6 promoter (obtained from pGEN-luc (Promega) using primers: ATTTAGGTGACACTATAGAAGTACTGTTGGTAAAGCCACCATGGAAGACGCCAAAAACAT and TTACAATTTGGACTTTCCGCCCT) (5 ng/µl) with SP6 RNA polymerase (1 U/µl) (New England BioLabs) in the presence of 1x RNA Pol Reaction Buffer (New England BioLabs), 5 mM DTT, RiboLock RNase Inhibitor (ThermoFisher Scientific) (2.0 U/µl), 0.5 mM UTP, 0.5 mM ATP, 0.5 mM CTP, 0.125 mM GTP and 1.25 mM of appropriate triazole-modified dinucleotide cap analogue and reference GpppG, m^7GpppG and $m_2^{7,3'-O}$GpppG. The reaction mixture was incubated at 40 °C for 1.5 h followed by addition of TURBO Dnase (Ambion) (0.1 U/µl) and incubation at 37 °C for another 30 min. The reaction was quenched by addition of 2 µl of 0.5 M EDTA pH 8.0 and purified with NucleoSpin RNA Clean-up XS kit (Macherey-Nagel) to afford luciferase-coding RNA strands capped with appropriate cap analogue.

3.3.2 Translation

For each capped luciferase-coding RNA four diluted solutions were prepared – 3.0 ng/µl, 1.5 ng/µl, 0.75 ng/µl, 0.375 ng/µl. Translation studies were performed using Rabbit Reticulocyte Lysate System (Promega). 9 µl of a mixture containing Rabbit Reticulocyte Lysate (4µl), Amino Acid Mixture Minus Leucine (0.2 µl of 1 mM solution), Amino Acid Mixture Minus Methionine (0.2 µl of 1 mM solution), potassium acetate (1.9 µl of 1 M solution), $MgCl_2$ (0.4 µl of 25 mM solution) and 2.1 µl of water was incubated at 30 °C for 1 h after which 1µl of appropriate luciferase-coding RNA solution was added and incubation of the reaction was continued at 37 °C for another hour. The reaction was stopped by freezing in liquid nitrogen.

Translation efficiency was determined using Luciferase Reporter System (Promega). The samples were defrosted just before the experiment. To every sample 50 µl of Luciferase Assay Reagent was added just before measurement of luminescence on Synergy H1 Microplate Reader (Bio Tek). The measurement were performed for every four samples independently due to low stability of luminescence signal. The results are presented as proportions between regression coefficients of linear relationships between capped luciferase-coding RNA concentration in translation reaction (300 pg/µl, 150 pg/µl, 75 pg/µl, 37.5 pg/µl) and corresponding luminescence signal.

3.4 Studies of susceptibility to degradation by hDcpS

hDcpS was expressed in *E.coli* and stored at 10 µM concentration (5 µM concentration of a dimer) in 20 mM Tris buffer, 50 mM KCl, 0.2 mM EDTA, 1 mM DTT, 0.5 mM PMSF, 20% glycerol at pH 7.5 and at -80 °C. An appropriate triazole-modified analogue (20 µM) was incubated at 20 °C in the presence of 50 mM Tris-HCl, 200 mM KCl, 0.5 mM EDTA, 1 mM DTT and 10 nM hDcpS (5 nM of a dimer) at pH 7.6 in the volume of 1 mL. 150 µl aliquots of the reaction mixture were collected after 5, 10, 15, 30, 60 min and 24 h and thermally deactivated at 98 °C for 2 min 40 s. The 150 µl control mixture (time point "0 min") did not contain the enzyme and was treated the same as the sample deactivated after 24 h. The samples were stored on ice and analyzed by RP-HPLC using a linear gradient from 0–50% of methanol in 0.1 M KH_2PO_4 pH 6.0 within 15 min (UV-detection at 260 nm).

Non-hydrolyzed fraction of the substrate (Y) at each time point was calculated as follows:

$\mathbf{Y} = \frac{\mathbf{S}}{\mathbf{0.9 \cdot (P_1 + P_2) + S}}$, where S – absorbance of the substrate, P_1, P_2 – absorbance of the hydrolysis product(s).

The analogue was considered as resistant to degradation by hDcpS if no products of hydrolysis were observed after 24 h of incubation in the presence of the enzyme and then subjected to analogous experiment but using different enzyme and analogue concentrations – 200 nM and 10 µM, respectively. In this case, the reaction was stopped after 30, 120 and 240 min.

3.5 Screening against hDcpS

For inhibitor screening purpose a fluorescence-based method was applied with minor modifications.[11] In order to initially assess inhibitory properties of new cap analogues, hydrolysis reactions of m^7GMPF (60 µM) were carried out in the presence of investigated compound at 20 µM. DcpS-catalyzed cleavage reactions were performed at 30 °C in 50 mM Tris/HCl, pH 7.6 buffer containing 200 mM KCl, 0.5 mM EDTA, 0.75 mg/mL BSA. Total reaction volume was 200 µl. Reactions were quenched after 55 min by addition of 100 µl of ACN. The extent of m^7GMPF hydrolysis was quantified by reaction of the released fluoride ions with fluorogenic probe (bis-(tert-butyldimethylsilyl-fluorescein). Conditions of deprotection reaction was the same as earlier.[11] Fluorescence intensity of formed

fluorescein was measured at 535 nm (excitation 480 nm) on a microplate reader. To determine IC_{50} parameter the same fluorescence-based method was used with each inhibitor evaluated at 10 different concentrations (range 0.02–500 μM).

3.6 hDcp2-catalyzed decapping

The reaction mixture containing synthesized **RNA3** capped with $m_2^{7,3'-O}$GpppG, **3e** and **8e** (for details of the synthesis see 3.2.2) was precipitated and the pellet was dissolved in water and incubated in the presence of 1x TURBO DNase Buffer (Ambion), RiboLock RNase Inhibitor (ThermoFisher Scientific) (2.0 U/μl) and TURBO Dnase (Ambion) (0.1 U/μl) at 37 oC for 30 min. The reaction was then subjected to phenol-chloroform extraction followed by precipitation. The resulting RNA2 water solution was directly used for Dcp2-catalyzed decapping experiments.

Appropriate RNA2 was incubated in the presence of 50 mM NH_4Cl, 5 mM $MgCl_2$, 50 mM Tris-HCl pH 8.0, 0.01% IGEPAL CA-630 (Sigma Aldrich), 2 mM $MnCl_2$, 1 mM DTT and 7 nM hDcp2 at 37 ^oC. 5 μl aliquots of the reaction were collected after 5, 15, 30, 60 min of incubation or just after addition of the enzyme (“0 min” time point) and quenched with loading dye (5 M urea, 44% formamide, 20 mM EDTA, 0.03% bromophenol blue, 0.03% xylene cyanol) and directly analyzed by 15% PAGE.

References:

1. T. M. Coleman, G. C. Wang and F. Q. Huang, *Nucleic Acids Research*, 2004, **32**.
2. P. Wanat, S. Walczak, B. A. Wojtczak, M. Nowakowska, J. Jemielity and J. Kowalska, *Organic Letters*, 2015, **17**, 3062-3065.
3. J. Jemielity, T. Fowler, J. Zuberek, J. Stepinski, M. Lewdorowicz, A. Niedzwiecka, R. Stolarski, E. Darzynkiewicz and R. E. Rhoads, *Rna-a Publication of the Rna Society*, 2003, **9**, 1108-1122.
4. S. Bandyopadhyay, S. Mukherjee and A. Dey, *Rsc Advances*, 2013, **3**, 17174-17187.
5. Mukaiyam.T and Hashimot.M, *Journal of the American Chemical Society*, 1972, **94**, 8528-&.
6. L. V. Lee, M. L. Mitchell, S. J. Huang, V. V. Fokin, K. B. Sharpless and C. H. Wong, *Journal of the American Chemical Society*, 2003, **125**, 9588-9589.
7. A. Guranowski, A. M. Wojdyla, A. M. Rydzik, J. Stepinski and J. Jemielity, *Acta Biochimica Polonica*, 2011, **58**, 131-136.
8. J. Kowalska, M. Lewdorowicz, E. Darzynkiewicz and J. Jemielity, *Tetrahedron Letters*, 2007, **48**, 5475-5479.
9. G.-H. Lee, H. K. Lim and S. S. Hah, *Bulletin of the Korean Chemical Society*, 2011, **32**, 3767-3769.
10. A. Niedzwiecka, J. Marcotrigiano, J. Stepinski, M. Jankowska-Anyszka, A. Wyslouch-Cieszynska, M. Dadlez, A. C. Gingras, P. Mak, E. Darzynkiewicz, N. Sonenberg, S. K. Burley and R. Stolarski, *Journal of Molecular Biology*, 2002, **319**, 615-635.
11. M. R. Baranowski, A. Nowicka, J. Jemielity and J. Kowalska, *Organic & Biomolecular Chemistry*, 2016, **14**, 4595-4604.
