# Supplementary material for: A novel route for preparing 5′ cap mimics and capped RNAs: phosphate-modified cap analogues obtained via click chemistry
Source: Chem Sci. 2016 Aug 16;8(1):260–7. doi: 10.1039/c6sc02437h (PMC5355871; doi:10.1039/c6sc02437h)
Supplement: Supplementary file 2 [file SC-008-C6SC02437H-s002.pdf]

Electronic Supplementary Material (ESI) for Chemical Science.
This journal is © The Royal Society of Chemistry 2016

A novel route for preparing 5′cap mimics and capped RNAs: phosphate-modified cap analogues obtained *via* click chemistry

S. Walczak,[ab] A. Nowicka,[ac] D. Kubacka,[c] K. Fac,[ab] P. Wanat,[c] S. Mroczek,[de] J. Kowalska[c] and J. Jemielity[a]

[a]*Centre of New Technologies, University of Warsaw, Banacha 2c, 02-097, Warsaw, Poland*
[b]*College of Inter-Faculty Individual Studies in Mathematics and Natural Sciences, University of Warsaw, Banacha 2c, 02-097, Warsaw, Poland*
[c]*Division of Biophysics, Institute of Experimental Physics, Faculty of Physics, University of Warsaw, Zwirki i Wigury 93, 02-089, Warsaw, Poland*
[d]*Department of Genetics and Biotechnology, Faculty of Biology, University of Warsaw, 02-106 Warsaw, Poland*
[e]*Institute of Biochemistry and Biophysics, Polish Academy of Sciences, 02-106 Warsaw, Poland*

Supplementary Information

Content

HPLC gradients....3
(1a) m^7G-triazole-C_2H_4ppG....3
(1b) m^7G-triazole-C_2H_4pppG....5
(1c) m^7GppC_2H_4-triazole-G....7
(1d) m^7GpppC_2H_4-triazole-G....9
(2a) m^7G-triazole-CH_2ppG....11
(2b) m^7G-triazole-CH_2pppG....13
(2c) m^7GppCH_2-triazole-G....15
(2d) m^7GpppCH_2-triazole-G....17
(3a) m^7G-triazole-ppG....19
(3b) m^7G-triazole-pppG....21
(3c) m^7Gpp-triazole-G....23
(3d) m^7Gppp-triazole-G....25
(3e) $m_2^{7,2'-O}$Gppp-triazole-G....27
(4a) m^7G-triazole-CH_2OppG....29
(4b) m^7G-triazole-CH_2OpppG....31
(4c) m^7GppOCH_2-triazole-G....33
(4d) m^7GpppOCH_2-triazole-G....35
(5a) m^7G-triazole-CH_2SppG....37
(5b) m^7Gp-triazole-CH_2SpppG....39
(5c) m^7GppSCH_2-triazole-G....42
(5d) m^7GpppSCH_2-triazole-G....44
(6a) m^7G-triazole-CH_2NHppG....46
(6b) m^7G-triazole-CH_2NHpppG....48
(6c) m^7GppNHCH_2-trazole-G....50

(6d) m^7GpppNHCH$_2$-triazole-G 52
(7a) m^7GpNHC$_2$H$_4$-triazole-CH$_2$ppG 54
(7b) m^7GpNHC$_2$H$_4$-triazole-CH$_2$pppG 56
(7c) m^7GppCH$_2$-triazole-C$_2$H$_4$NHpG 58
(7d) m^7GpppCH$_2$-triazole-C$_2$H$_4$NHpG 60
(8a) m^7GpNHC$_2$H$_4$-triazole-ppG 62
(8b) m^7GpNHC$_2$H$_4$-triazole-pppG 65
(8c) m^7Gpp-triazole-C$_2$H$_4$NHpG 67
(8d) m^7Gppp-triazole-C$_2$H$_4$NHpG 69
(8e) $m_2^{7,2'-O}$Gppp-triazole-C$_2$H$_4$NHpG 71
(9a) m^7GppNHC$_2$H$_4$-triazole-C$_2$H$_4$ppG 73
(9b) m^7GppC$_2$H$_4$-triazole-C$_2$H$_4$NHppG 75
(10c) m^7GppC$_4$H$_5$ 77
(10d) m^7GpppC$_4$H$_5$ 79
(11c) m^7GppC$_3$H$_3$ 81
(11d) m^7GpppC$_3$H$_3$ 83
(12c) m^7GppC$_2$H 85
(12d) m^7GpppC$_2$H 87
(12e) $m^{2'-O}$GpppC$_2$H 89
(12f) $m_2^{7,2'-O}$GpppC$_2$H 91
(13a) GppOC$_3$H$_3$ 93
(13b) GpppOC$_3$H$_3$ 95
(13c) m^7GppOC$_3$H$_3$ 97
(13d) m^7GpppOC$_3$H$_3$ 99
(14a) GppSC$_3$H$_3$ 101
(14b) GpppSC$_3$H$_3$ 103
(14c) m^7GppC$_3$H$_3$ 105
(14d) m^7GpppSC$_3$H$_3$ 107
(15a) GppNHC$_3$H$_3$ 109
(15b) GpppNHC$_3$H$_3$ 111
(15c) m^7GppNHC$_3$H$_3$ 113
(15d) m^7GpppNHC$_3$H$_3$ 115
(16a) 5'-N$_3$-Guo 117
(16b) 5'-N$_3$-m^7Guo 119
(17a) GpNHC$_2$H$_4$N$_3$ 121
(17b) GppNHC$_2$H$_4$N$_3$ 123
(17c) m^7GpNHC$_2$H$_4$N$_3$ 125
(17d) m^7GppNHC$_2$H$_4$N$_3$ 127
(18d) O-(2-propynyl) phosphate ester 129

HPLC gradients

Program A - linear gradient 0–25% of methanol in 0.05 M ammonium acetate buffer (pH 5.9) in 15 min

Program B - linear gradient 0–50% of methanol in 0.05 M ammonium acetate buffer (pH 5.9) in 15 min

Program C – linear gradient 0–50% of methanol in 0.05 M ammonium acetate buffer (pH 5.9) in 7.5 min and then isocratic elution (50% of methanol) until 15 min

(1a) m^7G-triazole-C_2H_4ppG

**Structure**	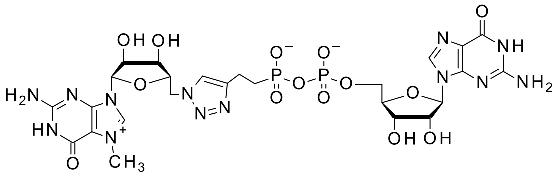
**Reaction HPLC profile**	Program C 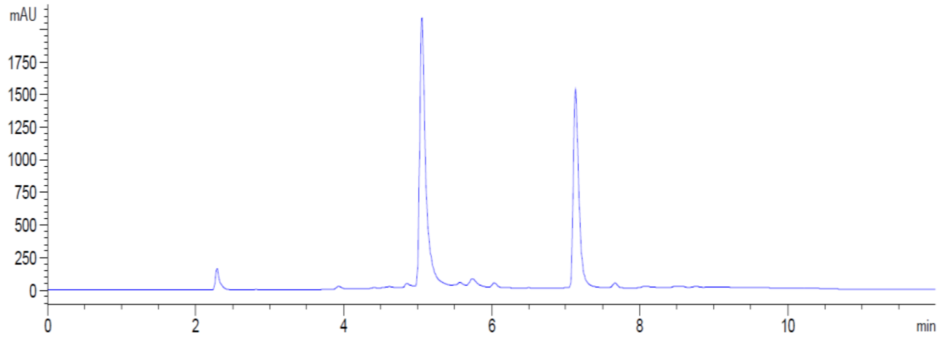
**Purified compound HPLC profile**	Program B 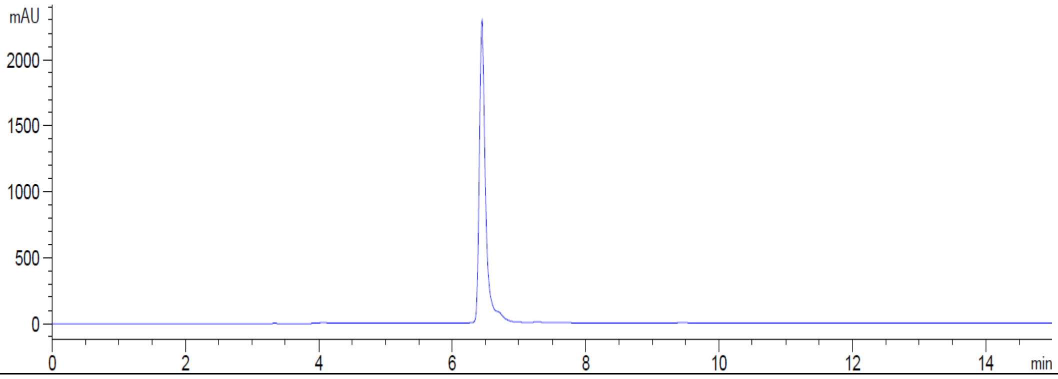

HRMS	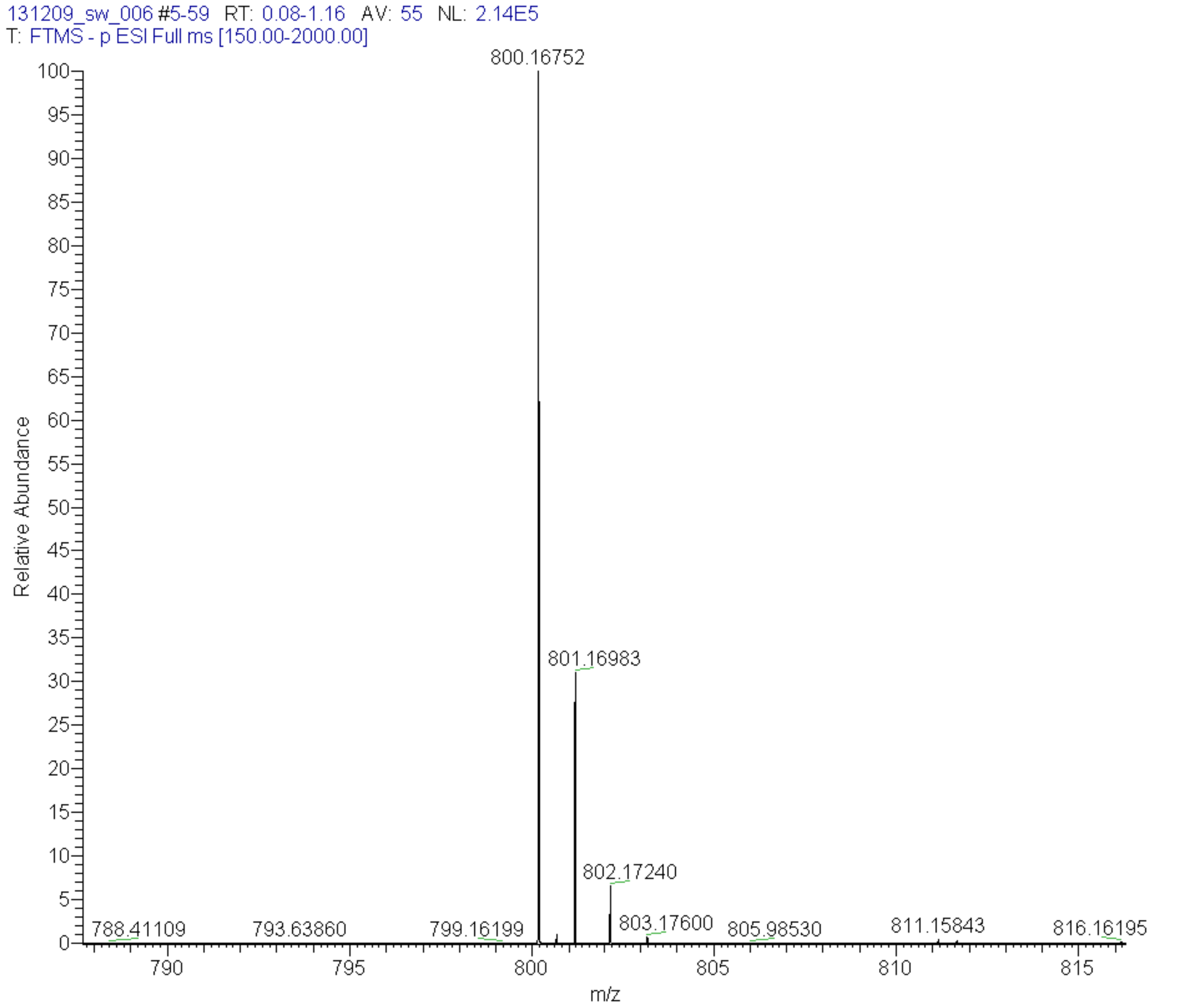
^{1}H NMR	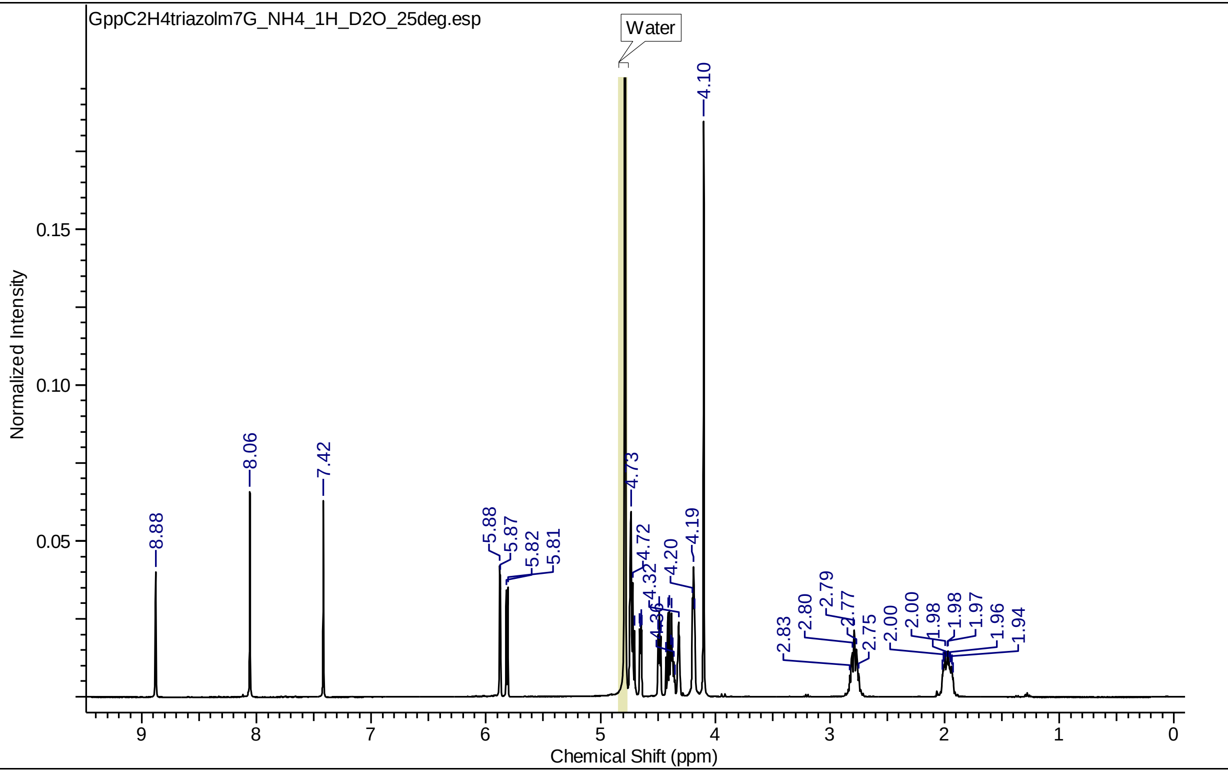

131209_sw_006 #5-59 RT: 0.08-1.16 AV: 55 NL: 2.14E5
T: FTMS - p ESI Full ms [150.00-2000.00]
800.16752
801.16983
802.17240
803.17600
788.41109
793.63860
799.16199
805.98530
811.15843
816.16195
Relative Abundance
m/z

GppC2H4triazolm7G_NH4_1H_D2O_25deg.esp
Water
Normalized Intensity
Chemical Shift (ppm)
8.88
8.06
7.42
5.88
5.87
5.82
5.81
4.73
4.72
4.32
4.20
4.19
4.10
2.83
2.80
2.79
2.77
2.75
2.00
2.00
1.98
1.98
1.97
1.96
1.94

^{31}P NMR

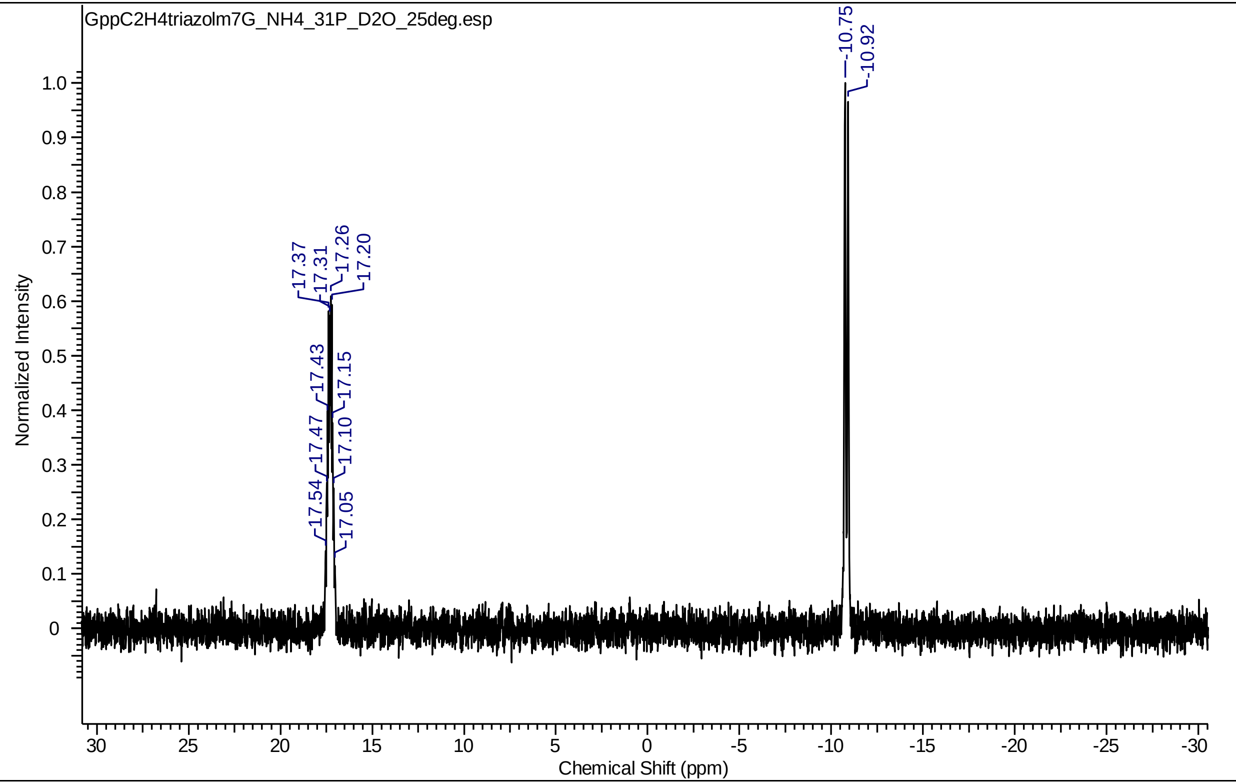

(1b) m^{7}G-triazole-C_2H_4pppG

Structure

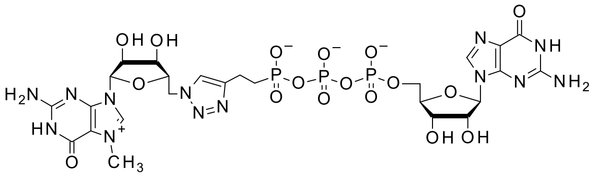

Reaction HPLC profile

Program C

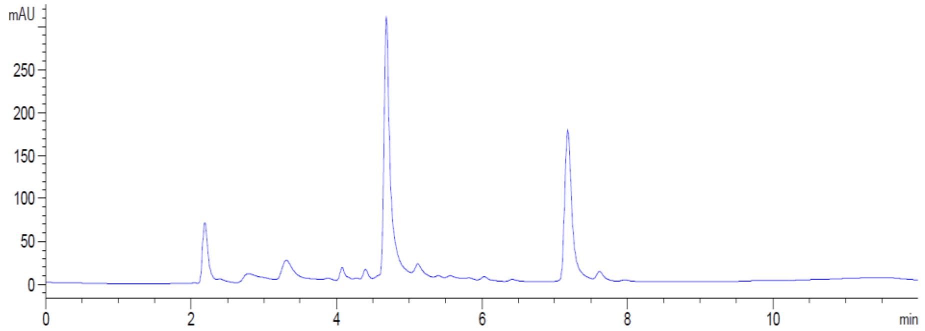

Purified compound HPLC profile

Program B

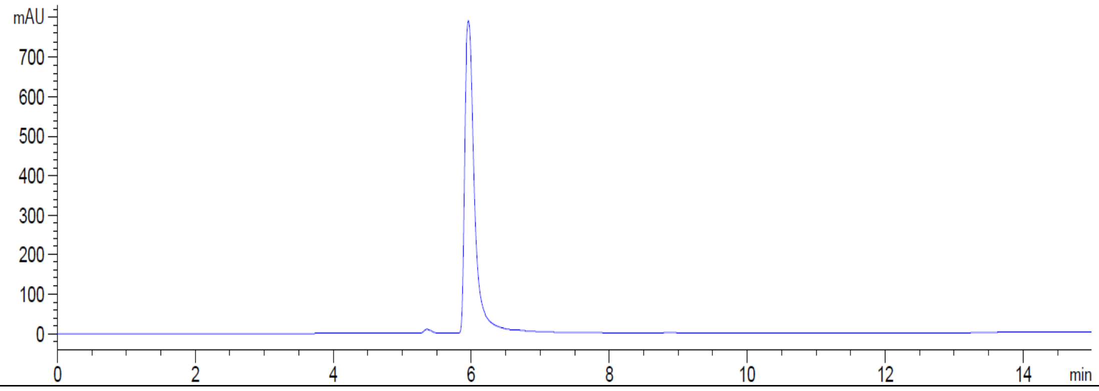

HRMS

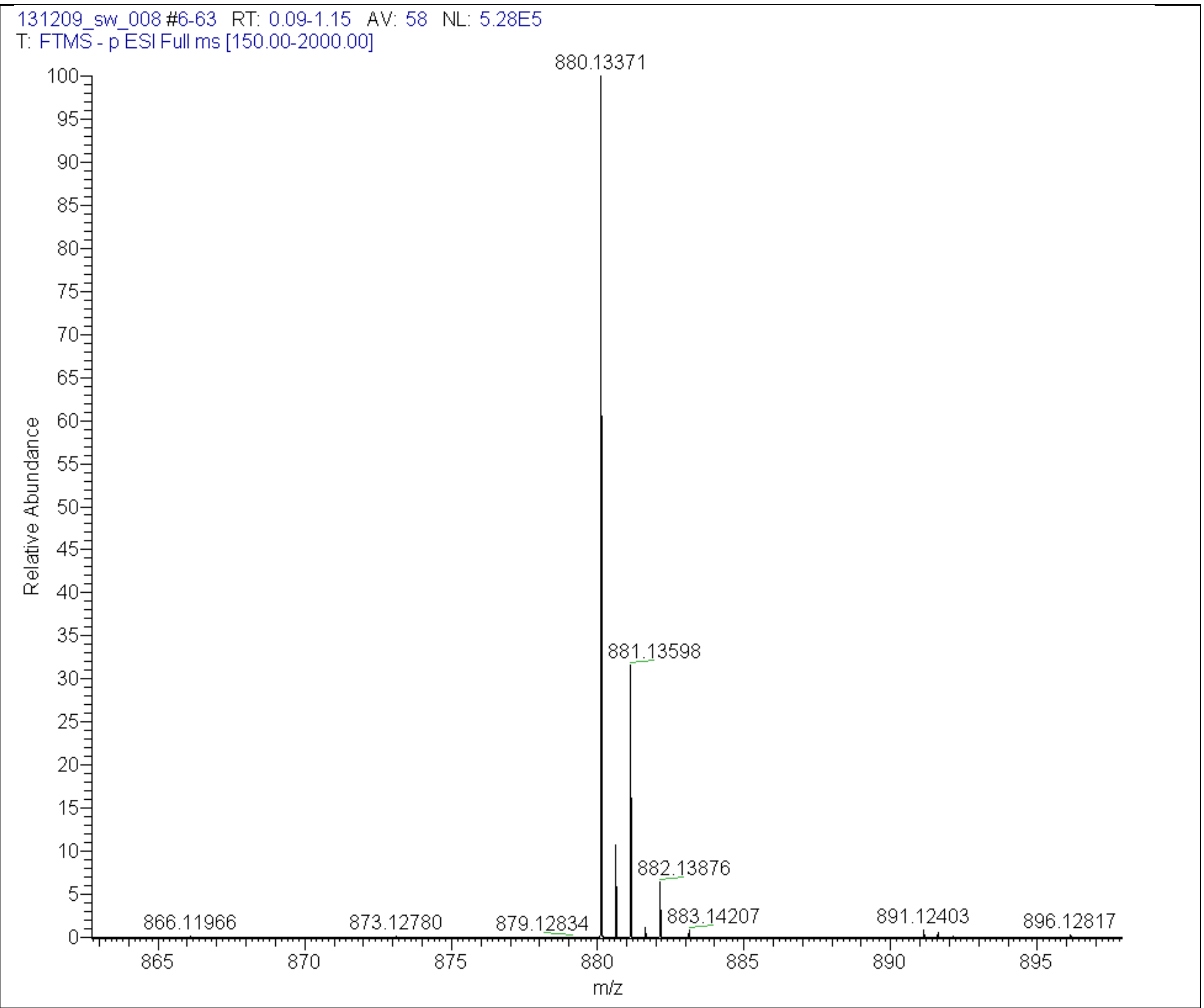
131209_sw_008 #6-63 RT: 0.09-1.15 AV: 58 NL: 5.28E5
T: FTMS - p ESI Full ms [150.00-2000.00]
880.13371
881.13598
882.13876
883.14207
866.11966
873.12780
879.12834
891.12403
896.12817
Relative Abundance
m/z

^{1}H NMR

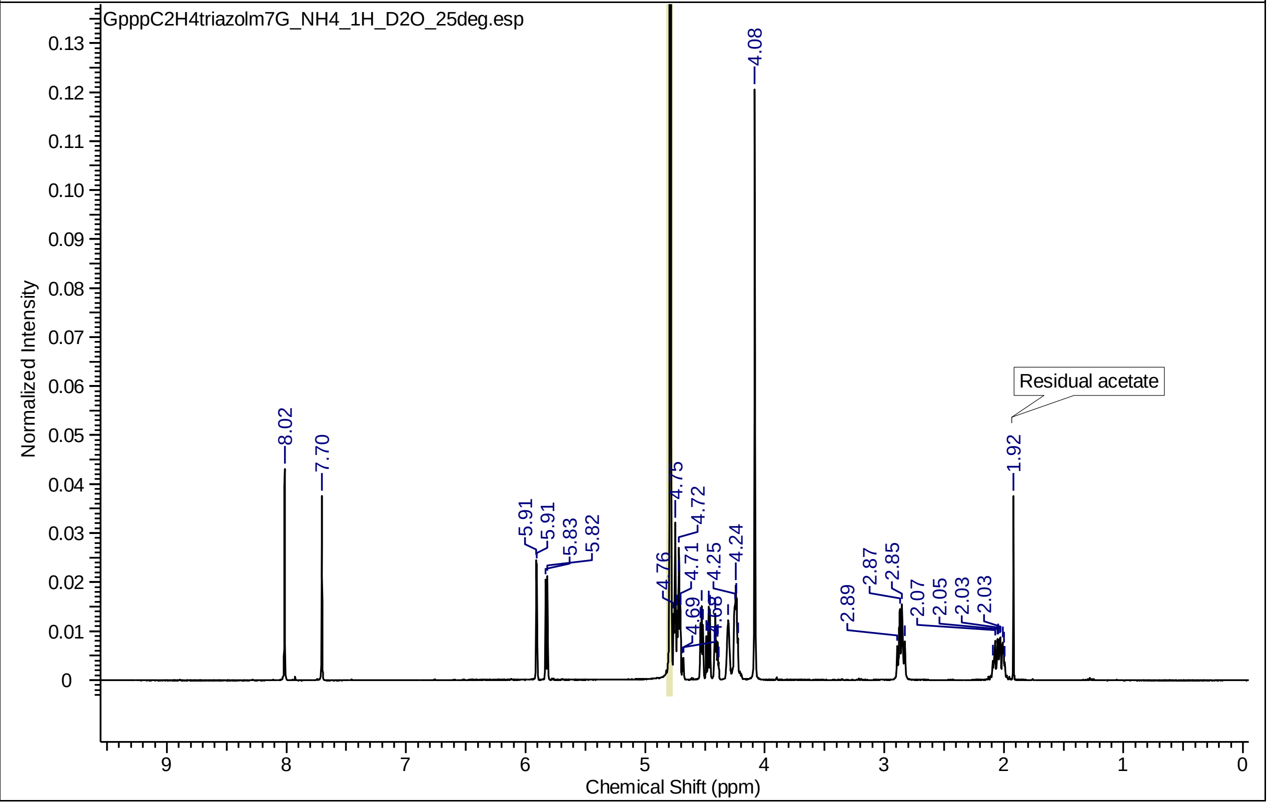
GpppC2H4triazolm7G_NH4_1H_D2O_25deg.esp
Normalized Intensity
Chemical Shift (ppm)
Residual acetate
8.02
7.70
5.91
5.91
5.83
5.82
4.76
4.75
4.72
4.71
4.69
4.25
4.24
4.08
2.89
2.87
2.85
2.07
2.05
2.03
2.03
1.92

^{31}P NMR

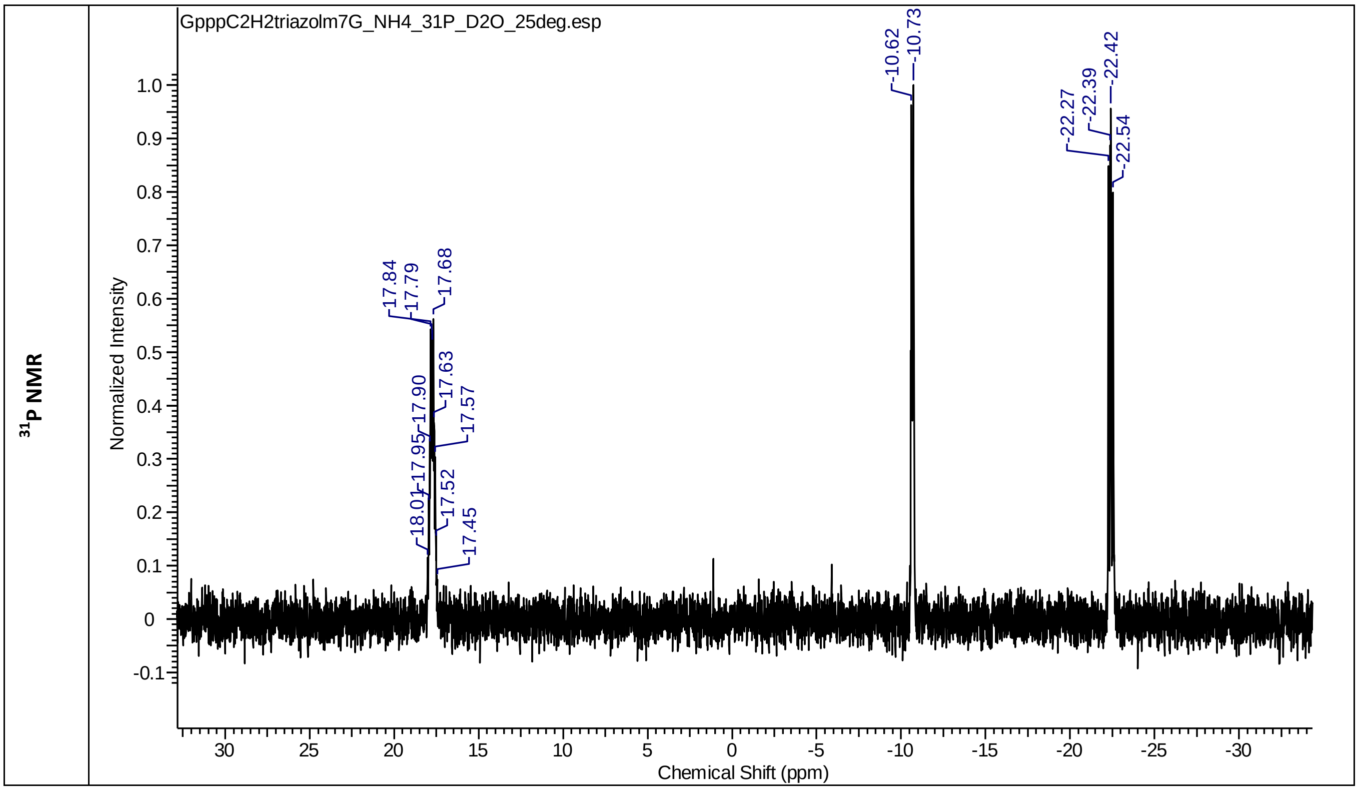

(1c) m^{7}GppC$_2$H$_4$-triazole-G

Structure

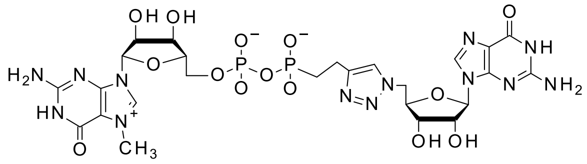

Reaction HPLC profile

Program C

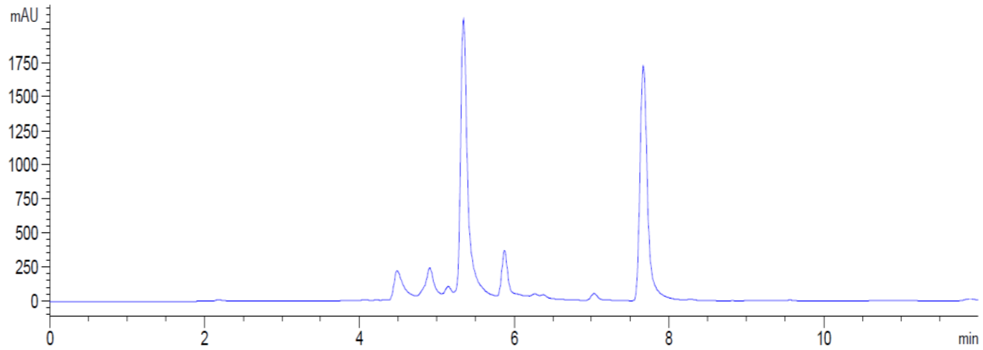

Purified compound HPLC profile

Program B

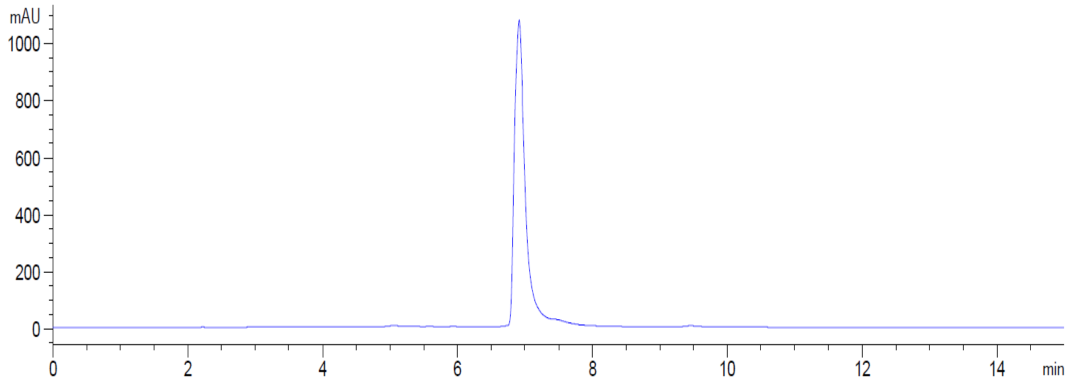

**HRMS**	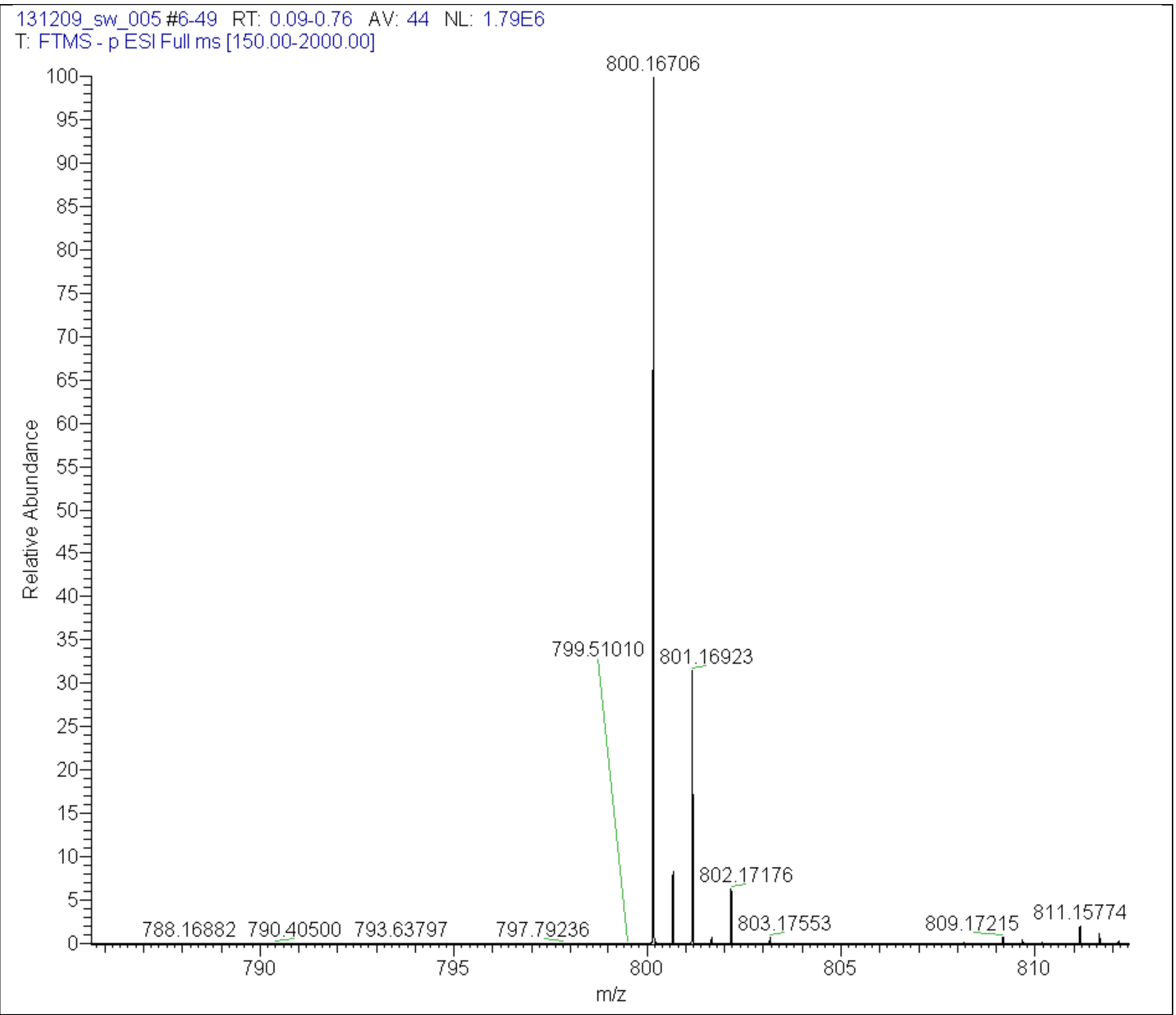
**^{1}H NMR**	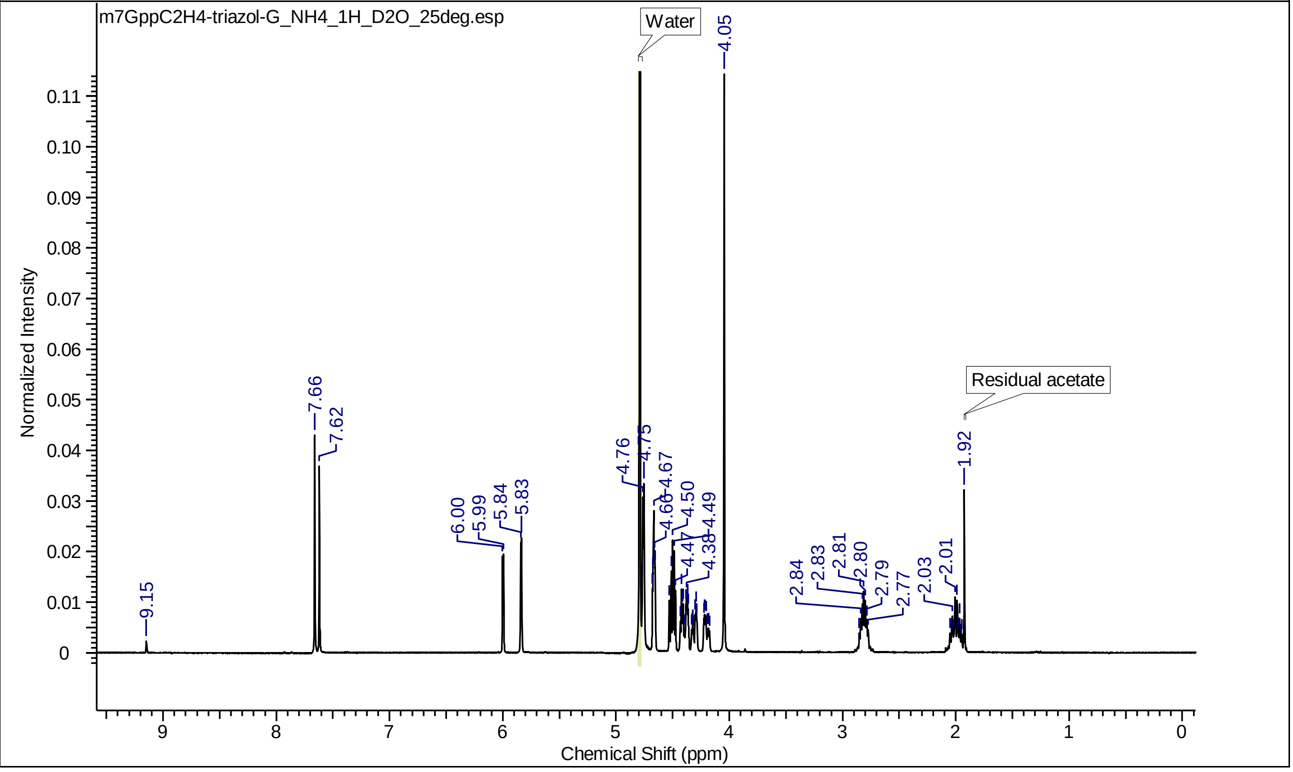

131209_sw_005 #6-49 RT: 0.09-0.76 AV: 44 NL: 1.79E6
T: FTMS - p ESI Full ms [150.00-2000.00]
800.16706
799.51010
801.16923
802.17176
803.17553
788.16882
790.40500
793.63797
797.79236
809.17215
811.15774
Relative Abundance
m/z

m7GppC2H4-triazol-G_NH4_1H_D2O_25deg.esp
Water
Residual acetate
9.15
7.66
7.62
6.00
5.99
5.84
5.83
4.76
4.75
4.67
4.60
4.50
4.49
4.47
4.38
4.05
2.84
2.83
2.81
2.80
2.79
2.77
2.03
2.01
1.92
Normalized Intensity
Chemical Shift (ppm)

**^{31}P NMR**	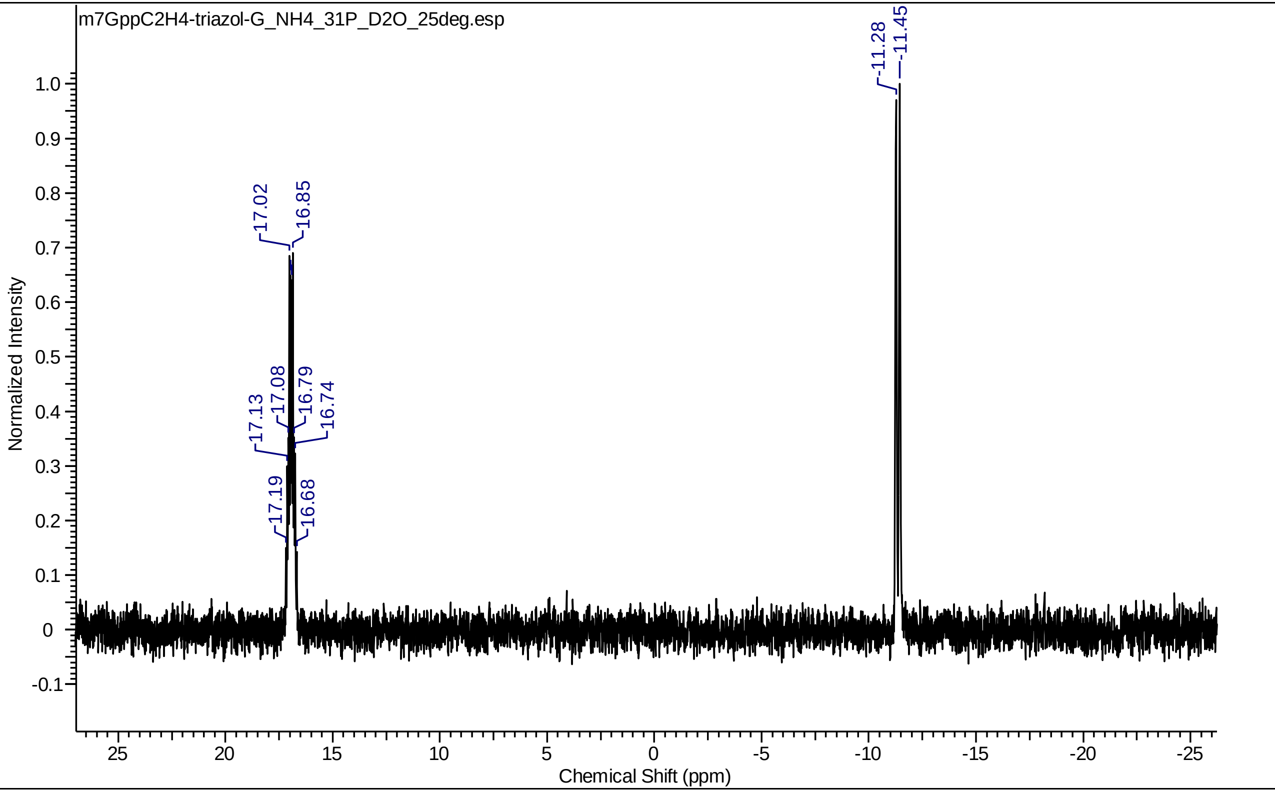

(1d) m^{7}GpppC$_2$H$_4$-triazole-G

**Structure**	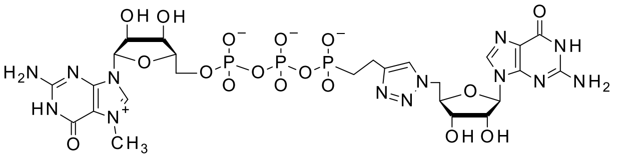
**Reaction HPLC profile**	Program C 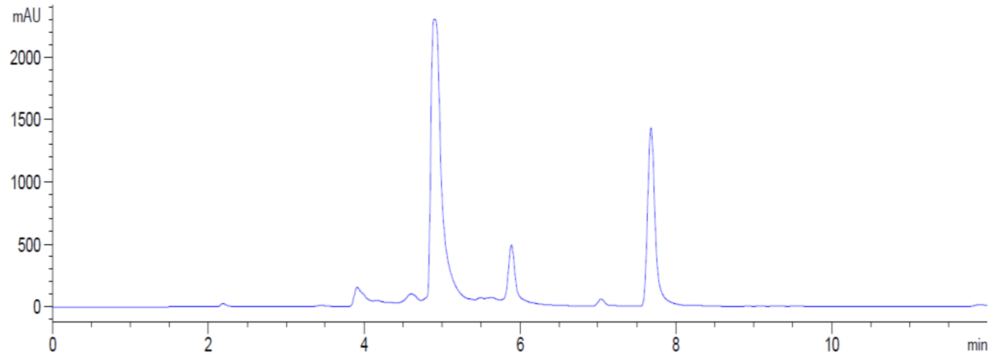
**Purified compound HPLC profile**	Program B 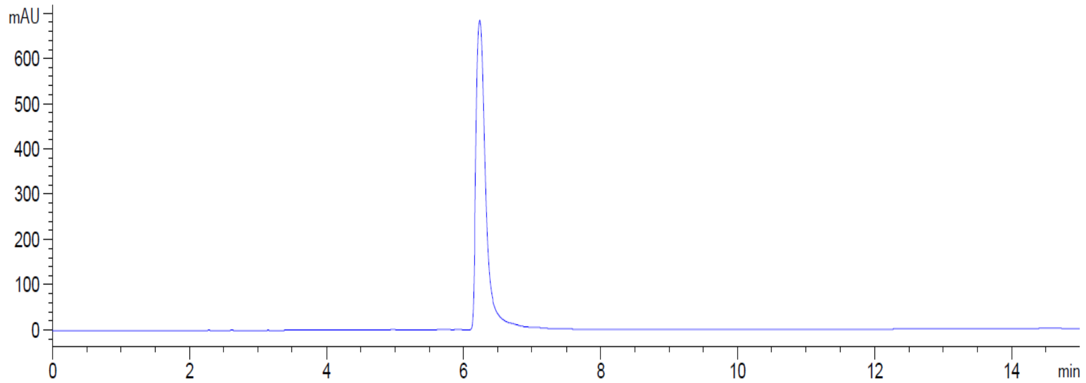

HRMS

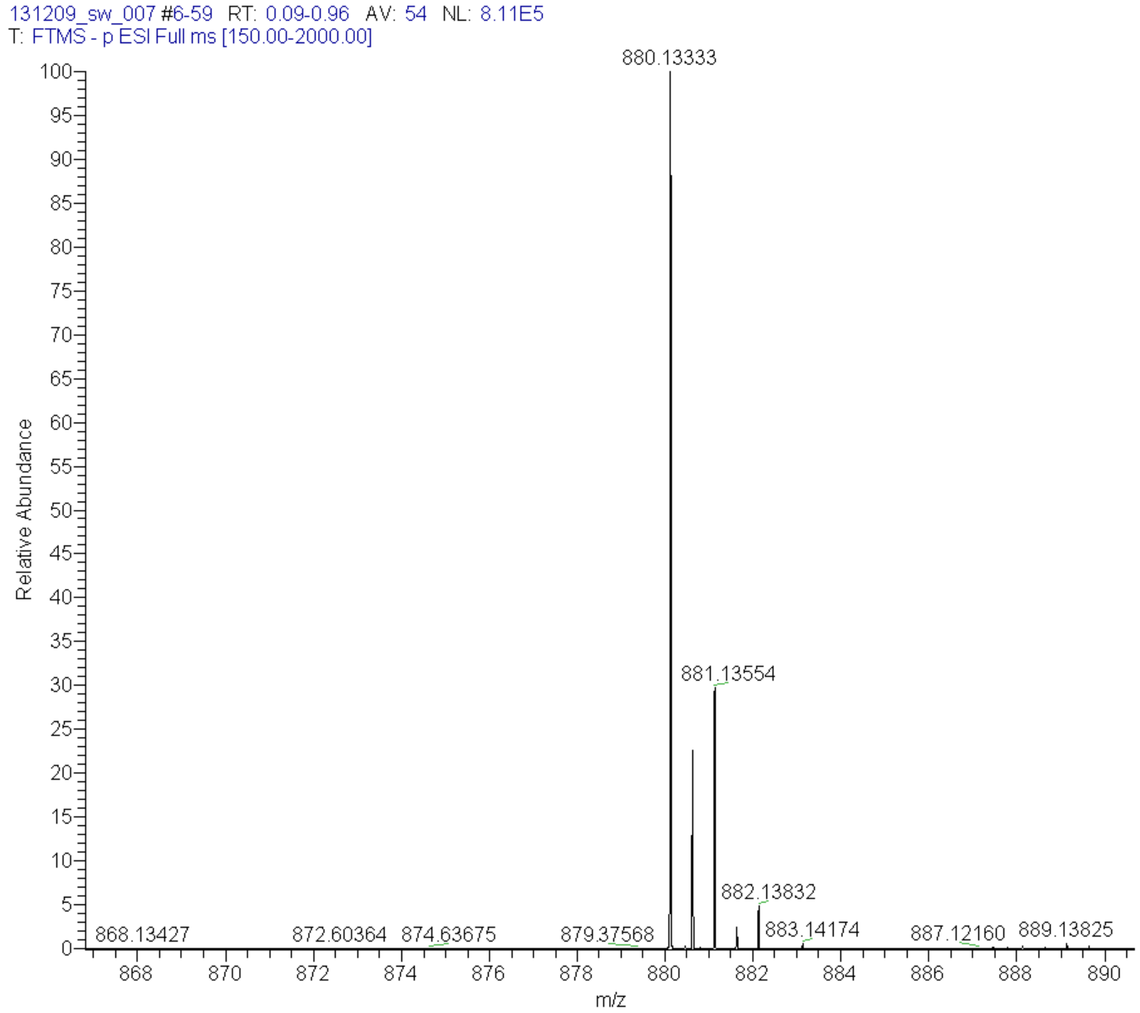

131209_sw_007 #6-59 RT: 0.09-0.96 AV: 54 NL: 8.11E5
T: FTMS - p ESI Full ms [150.00-2000.00]
880.13333
881.13554
882.13832
883.14174
868.13427
872.60364
874.63675
879.37568
887.12160
889.13825
Relative Abundance
m/z

^{1}H NMR

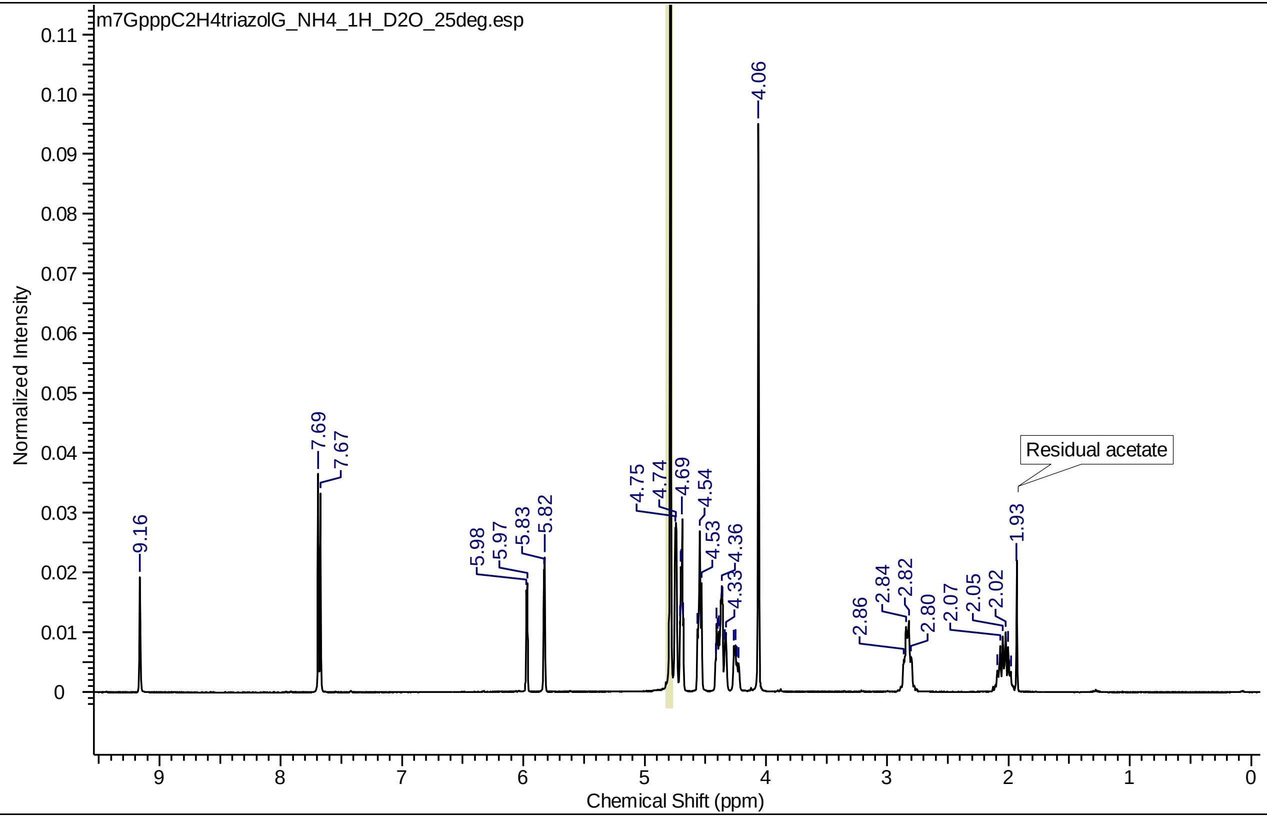

m7GpppC2H4triazolG_NH4_1H_D2O_25deg.esp
9.16
7.69
7.67
5.98
5.97
5.83
5.82
4.75
4.74
4.69
4.54
4.53
4.36
4.33
4.06
2.86
2.84
2.82
2.80
2.07
2.05
2.02
1.93
Residual acetate
Normalized Intensity
Chemical Shift (ppm)

^{31}P NMR

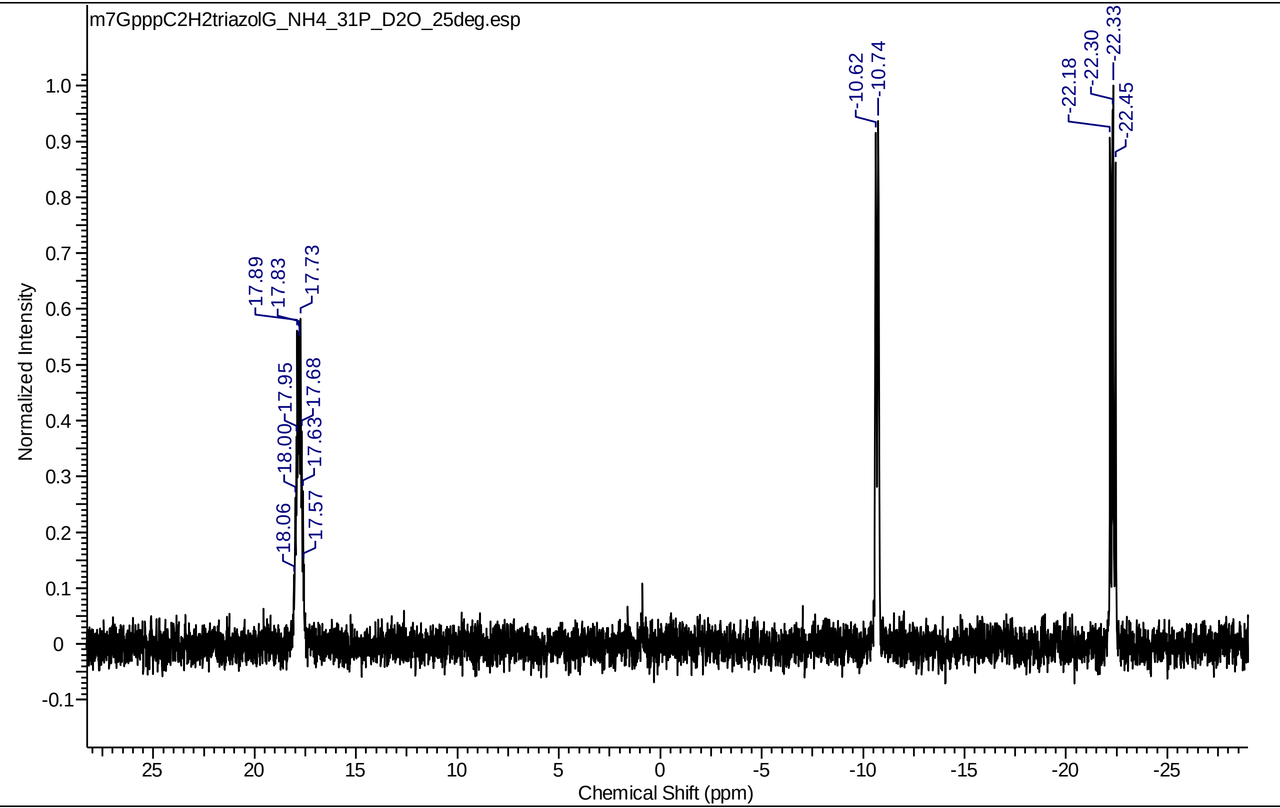

(2a) m[7]G-triazole-CH_2ppG

Structure

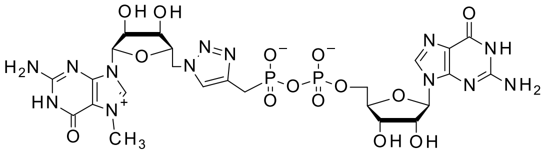

Reaction HPLC profile

Program C

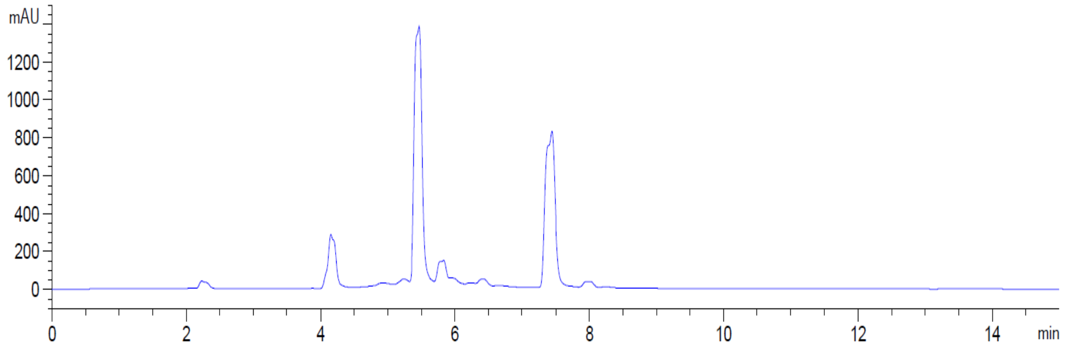

Purified compound HPLC profile

Program B

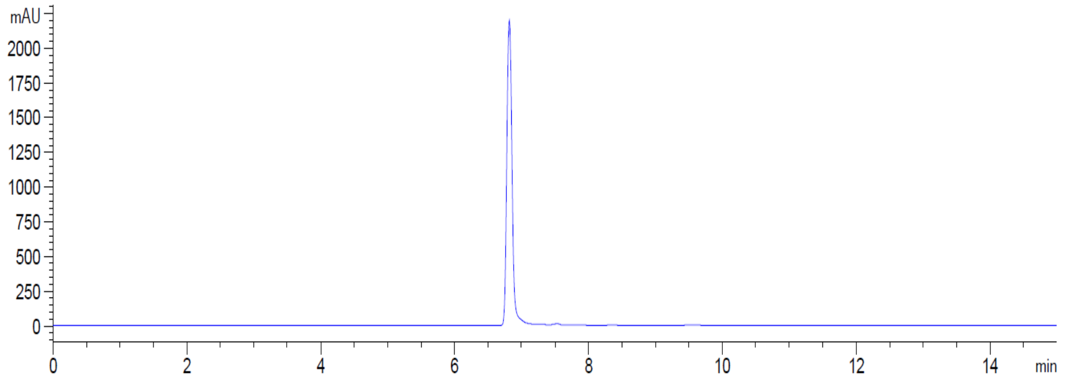

HRMS

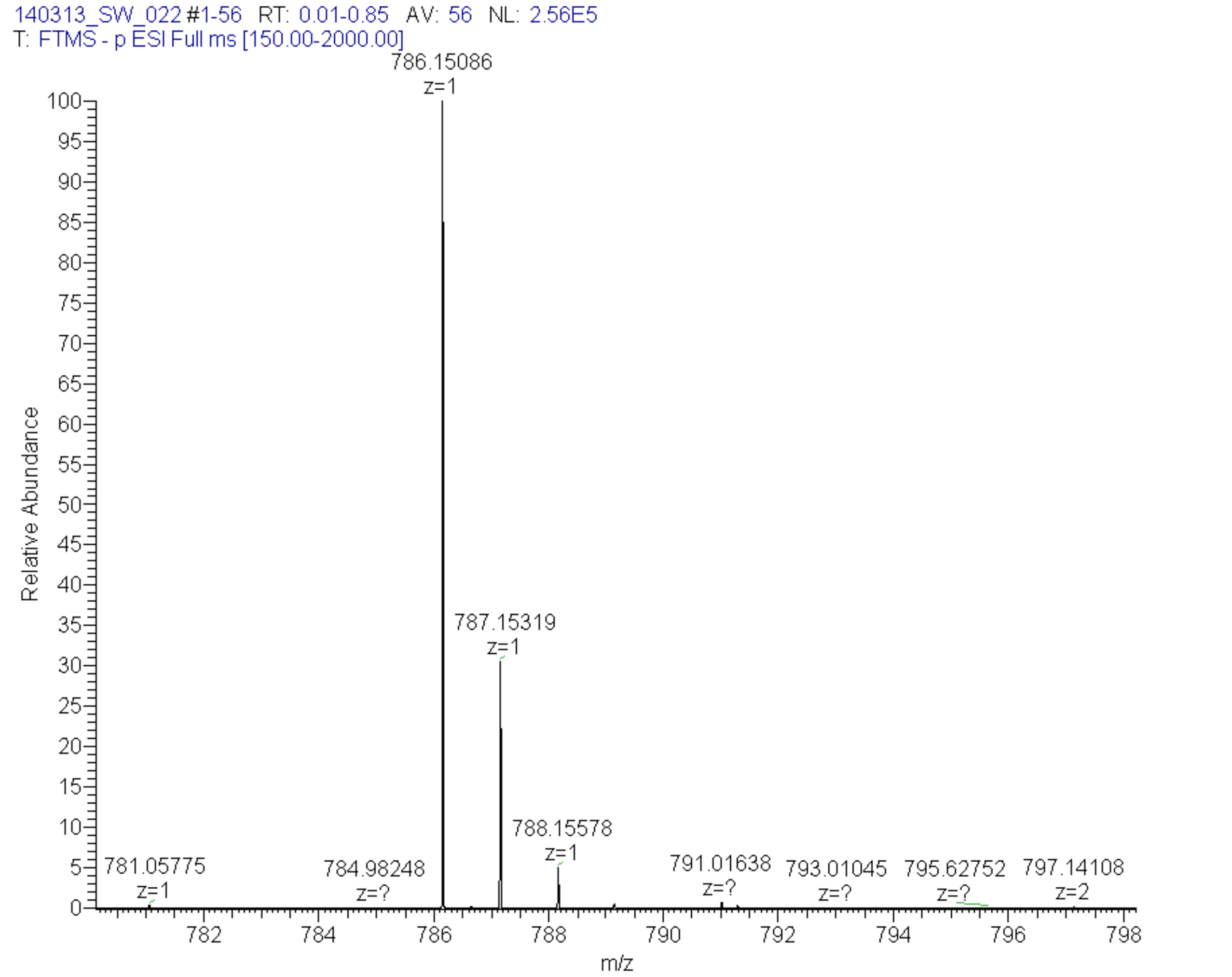

140313_SW_022 #1-56 RT: 0.01-0.85 AV: 56 NL: 2.56E5
T: FTMS - p ESI Full ms [150.00-2000.00]
786.15086 z=1
787.15319 z=1
788.15578 z=1
781.05775 z=1
784.98248 z=?
791.01638 z=?
793.01045 z=?
795.62752 z=?
797.14108 z=2
Relative Abundance
m/z

^{1}H NMR

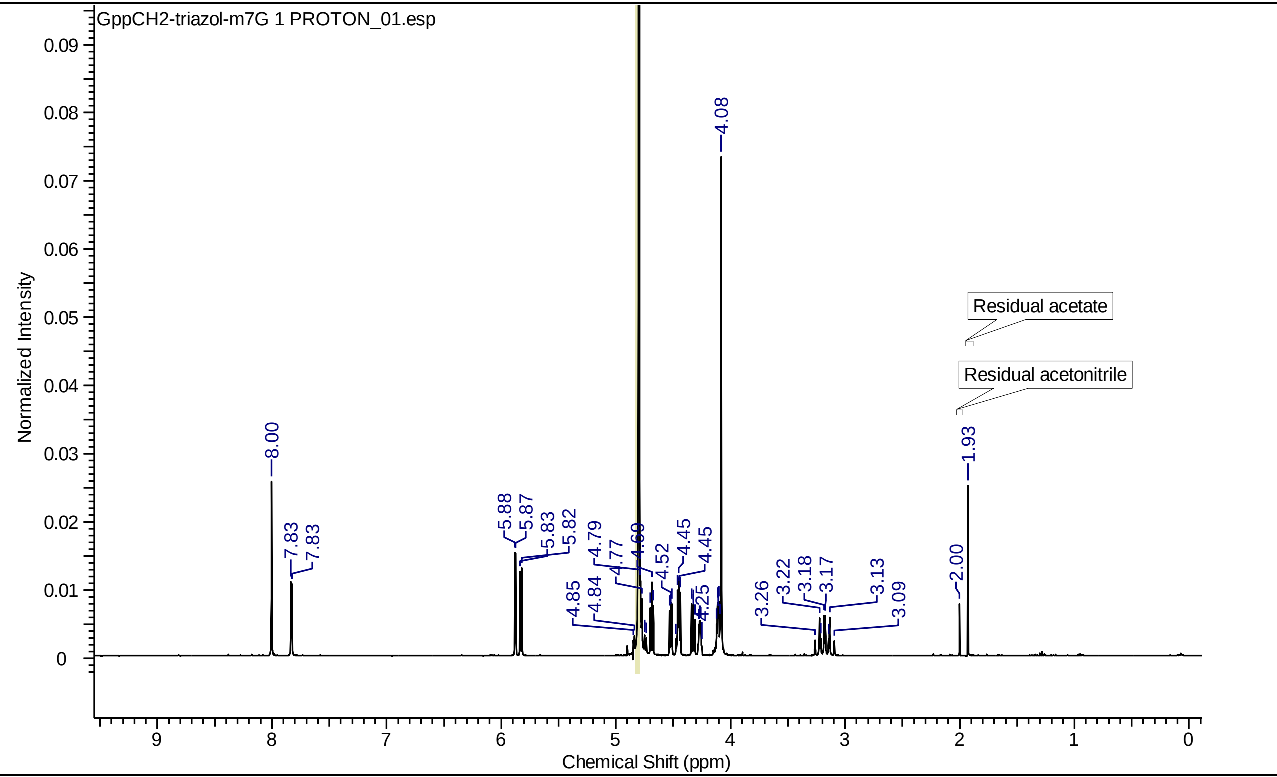

GppCH2-triazol-m7G 1 PROTON_01.esp
Residual acetate
Residual acetonitrile
Normalized Intensity
Chemical Shift (ppm)

^{31}P NMR

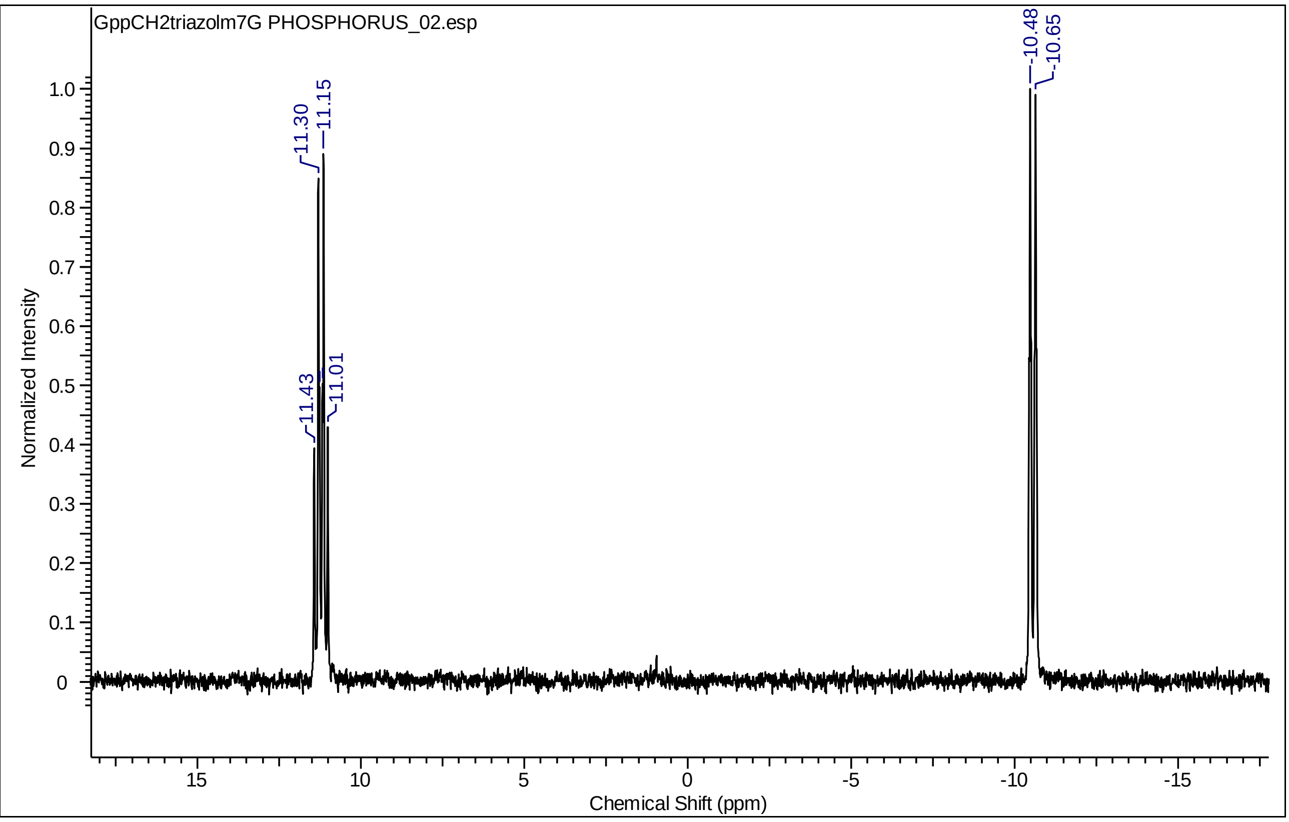

(2b) m^{7}G-triazole-CH$_2$pppG

Structure

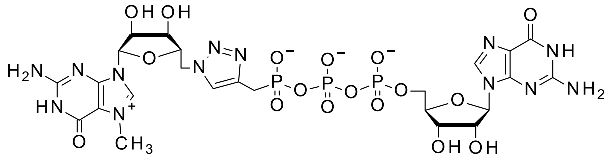

Reaction HPLC profile

Program C

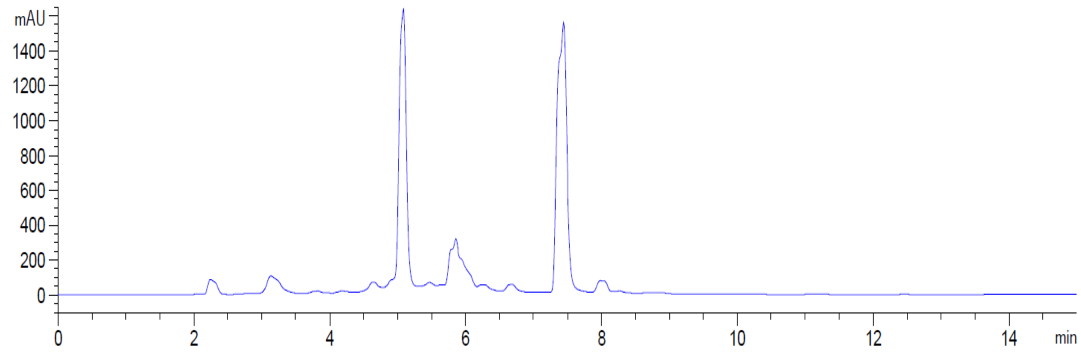

Purified compound HPLC profile

Program B

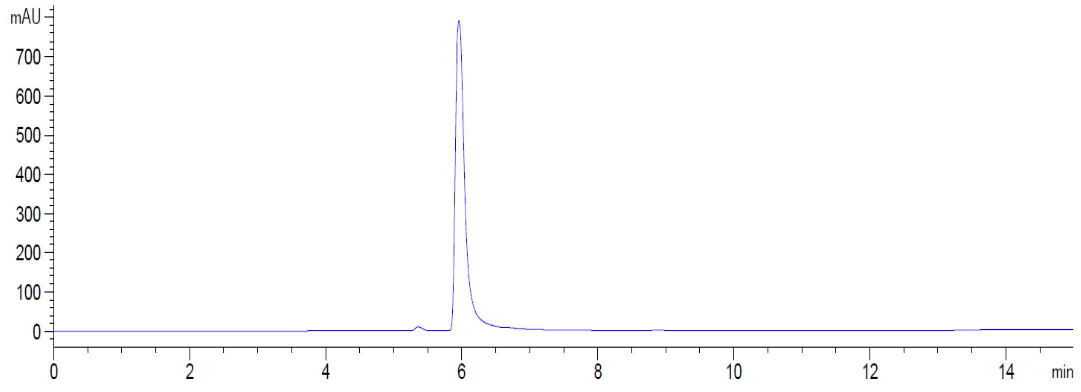

HRMS

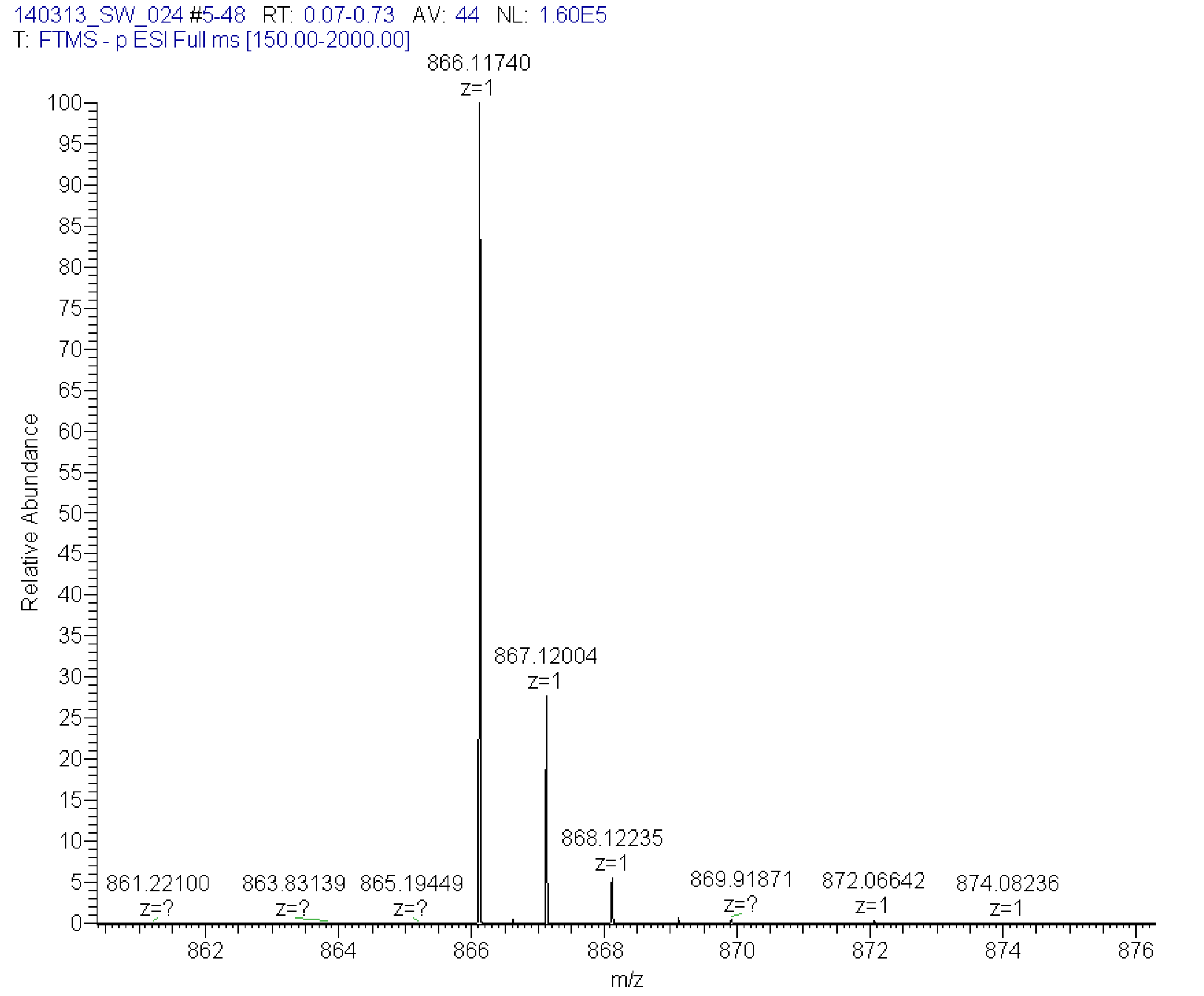

140313_SW_024 #5-48 RT: 0.07-0.73 AV: 44 NL: 1.60E5
T: FTMS - p ESI Full ms [150.00-2000.00]
866.11740 z=1
867.12004 z=1
868.12235 z=1
861.22100 z=?
863.83139 z=?
865.19449 z=?
869.91871 z=?
872.06642 z=1
874.08236 z=1
Relative Abundance
m/z

^{1}H NMR

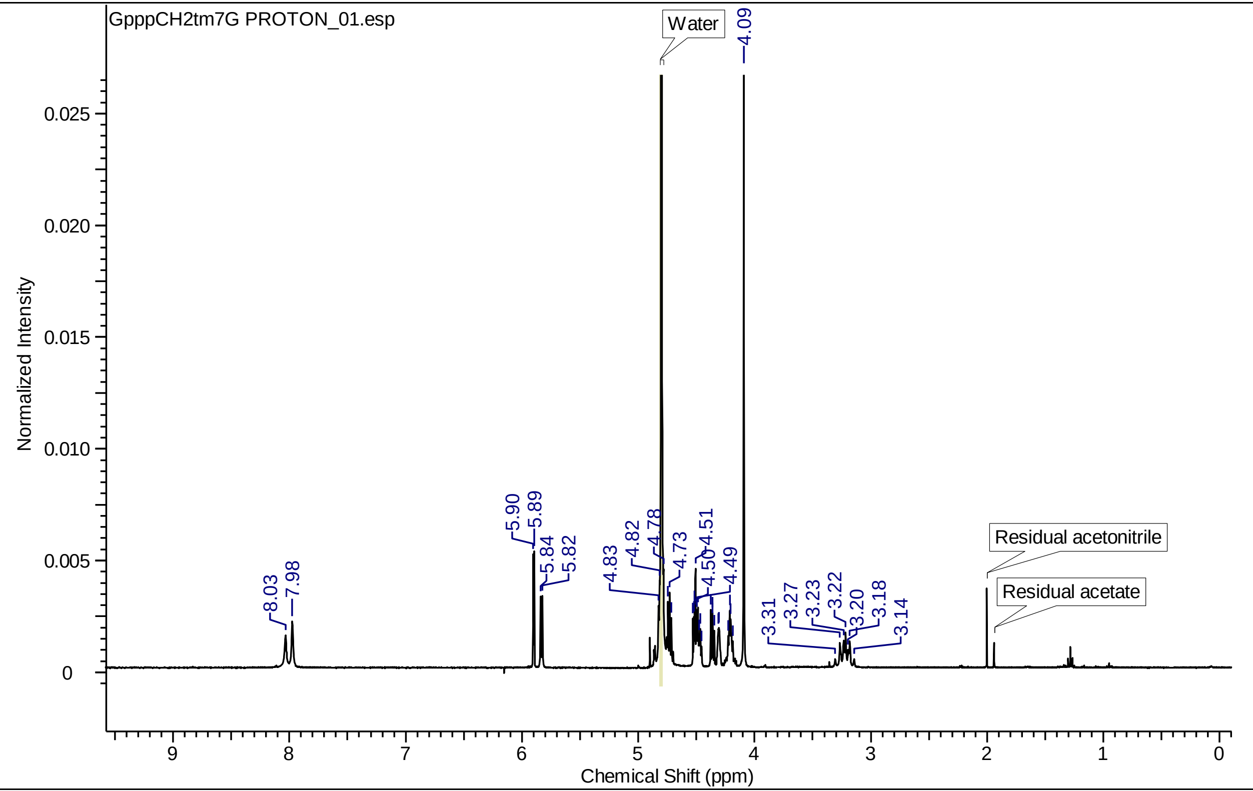

GpppCH2tm7G PROTON_01.esp
Water
4.09
Residual acetonitrile
Residual acetate
8.03
7.98
5.90
5.89
5.84
5.82
4.83
4.82
4.78
4.73
4.51
4.50
4.49
3.31
3.27
3.23
3.22
3.20
3.18
3.14
Normalized Intensity
Chemical Shift (ppm)

^{31}P NMR	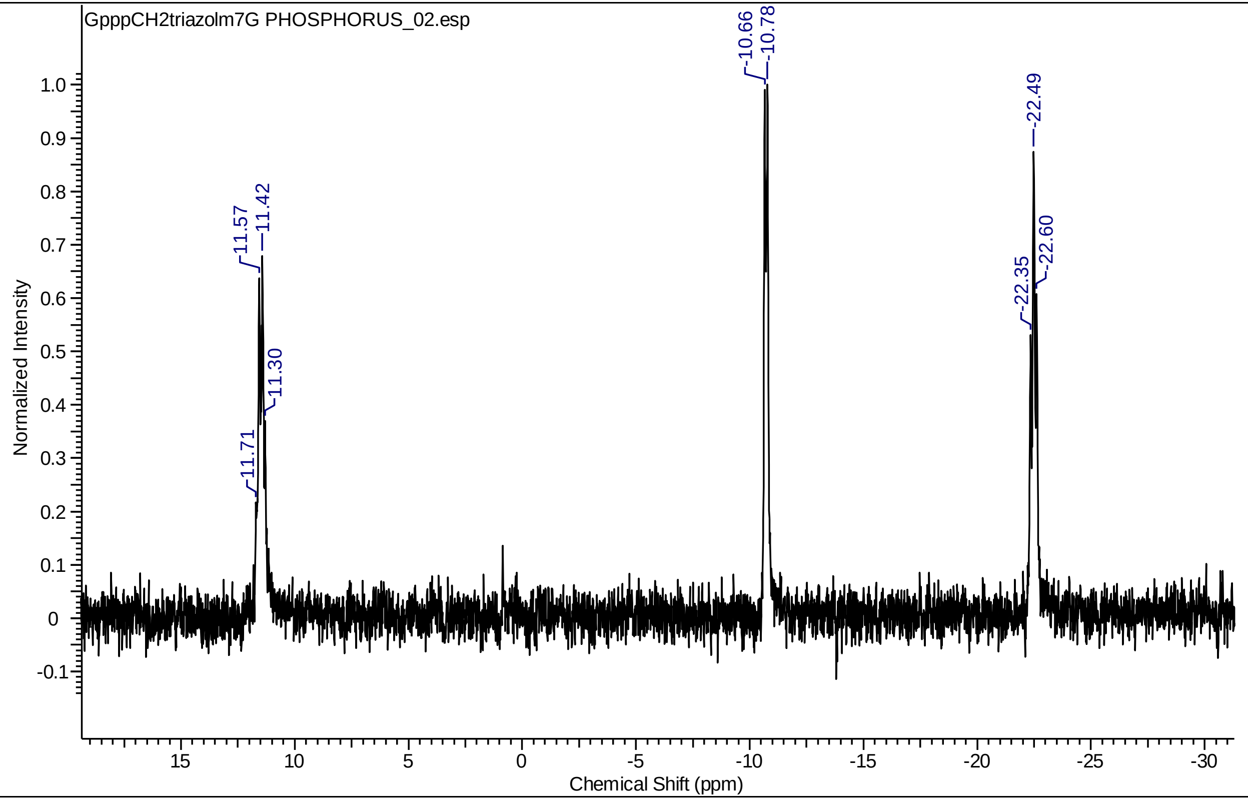

(2c) m^{7}GppCH$_2$-triazole-G

Structure	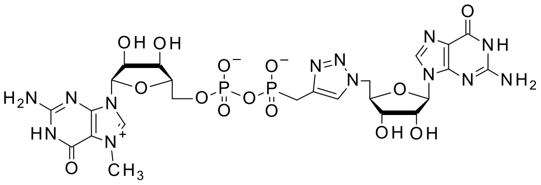
Reaction HPLC profile	Program C 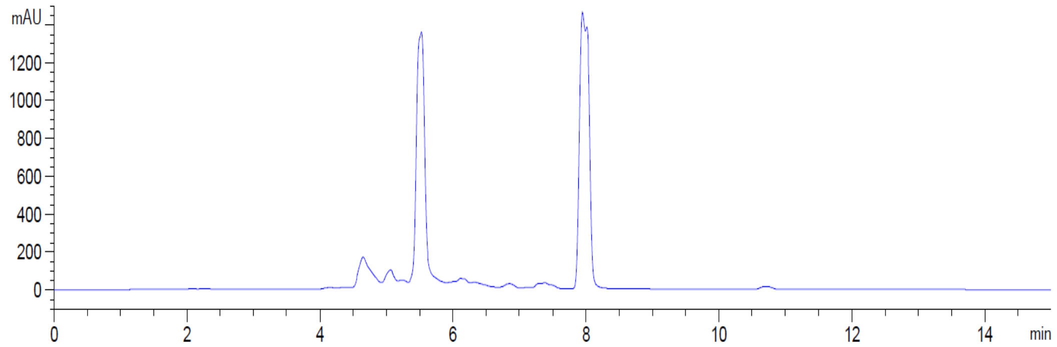
Purified compound HPLC profile	Program B 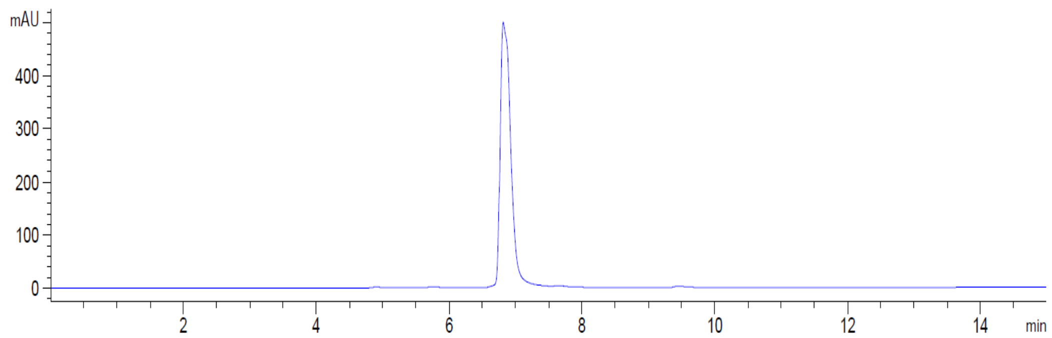

HRMS

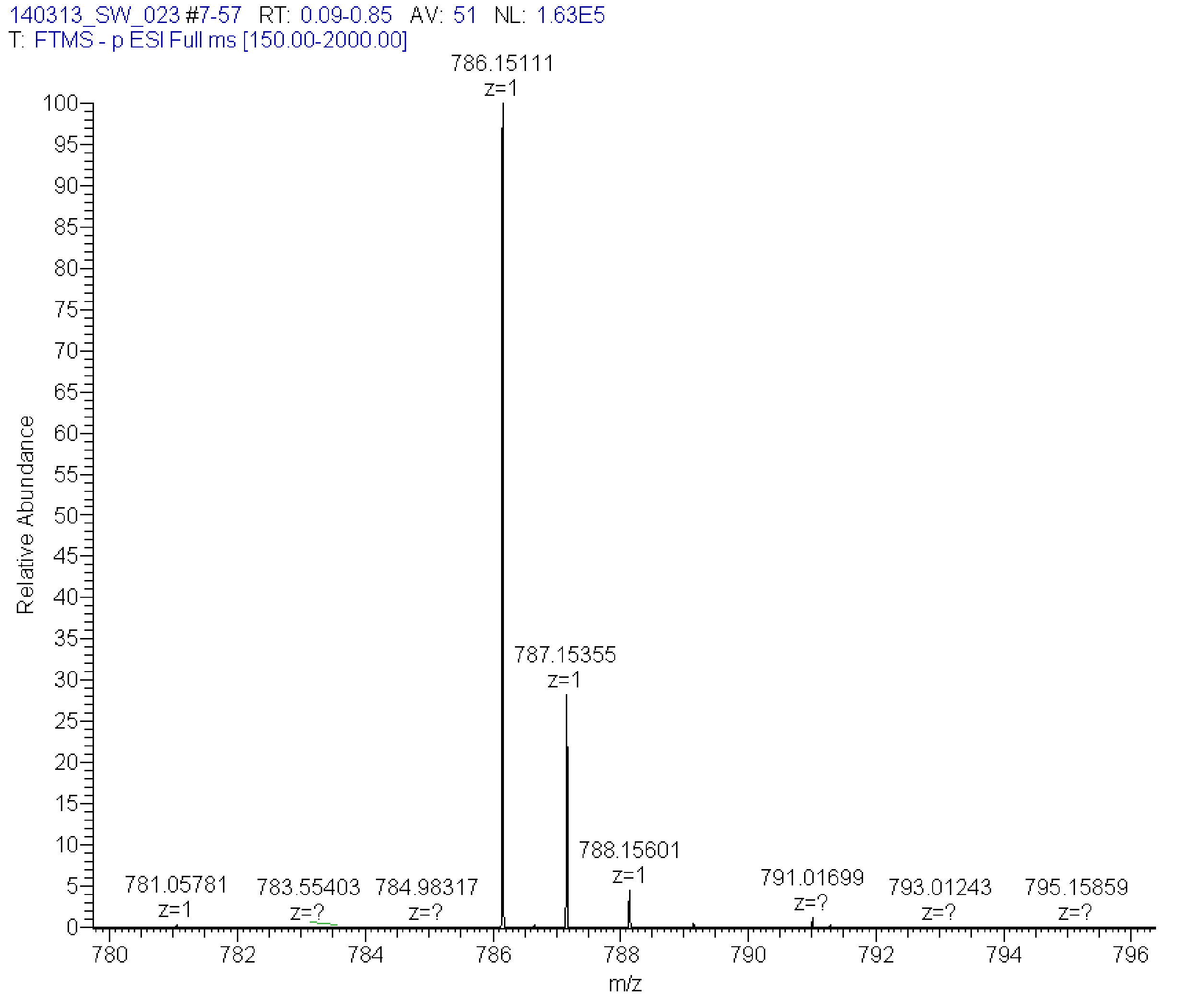
140313_SW_023 #7-57 RT: 0.09-0.85 AV: 51 NL: 1.63E5
T: FTMS - p ESI Full ms [150.00-2000.00]
786.15111
z=1
787.15355
z=1
788.15601
z=1
781.05781
z=1
783.55403
z=?
784.98317
z=?
791.01699
z=?
793.01243
z=?
795.15859
z=?
Relative Abundance
m/z

^{1}H NMR

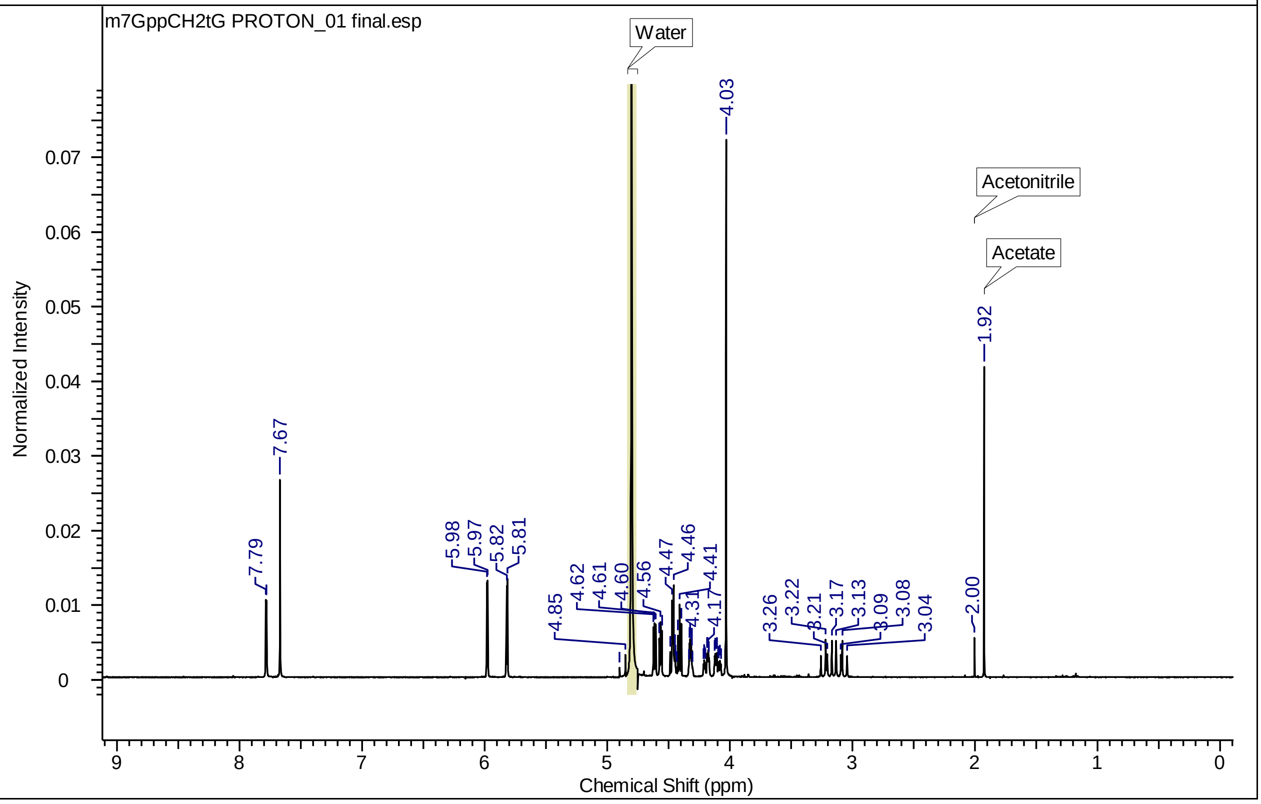
m7GppCH2tG PROTON_01 final.esp
Water
Acetonitrile
Acetate
7.79
7.67
5.98
5.97
5.82
5.81
4.85
4.62
4.61
4.60
4.56
4.47
4.46
4.41
4.31
4.17
4.03
3.26
3.22
3.21
3.17
3.13
3.09
3.08
3.04
2.00
1.92
Normalized Intensity
Chemical Shift (ppm)

^{31}P NMR

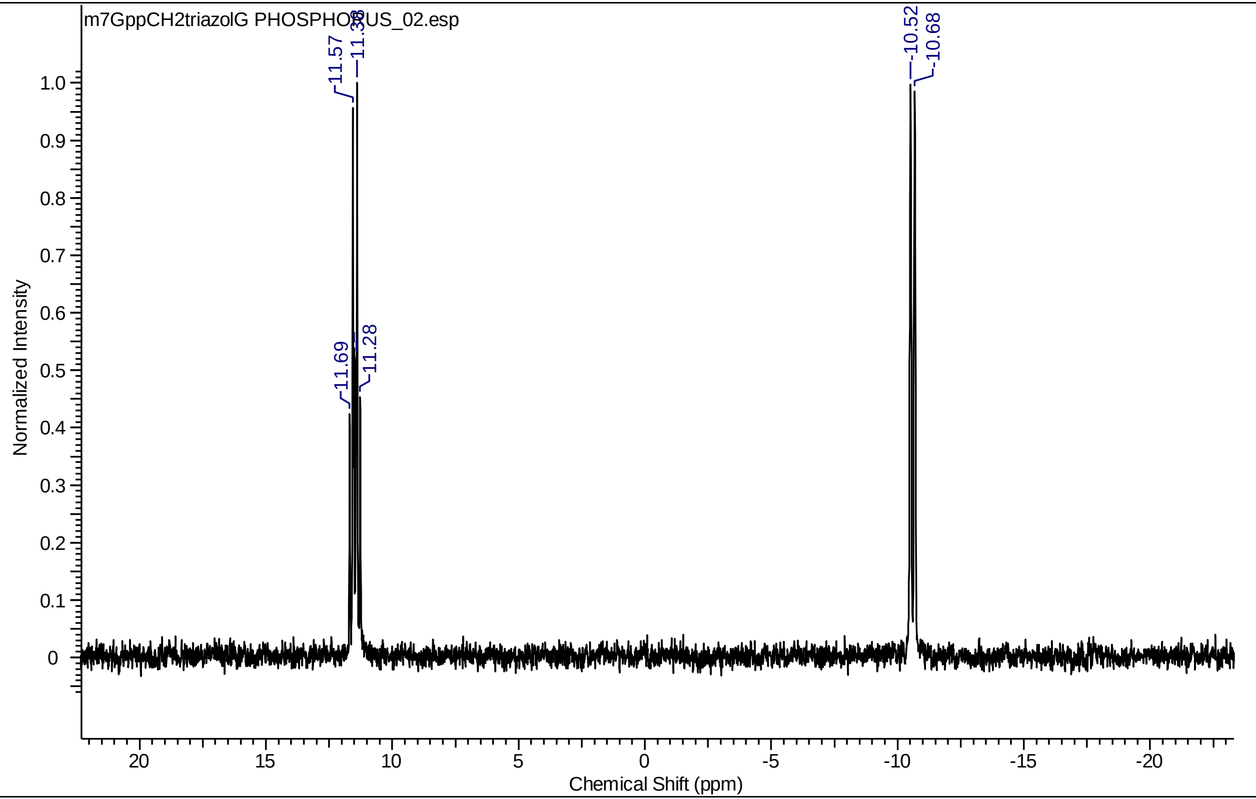

(2d) m^{7}GpppCH$_2$-triazole-G

Structure

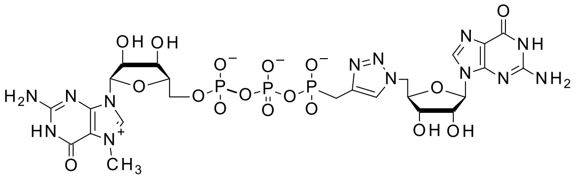

Reaction HPLC profile

Program C

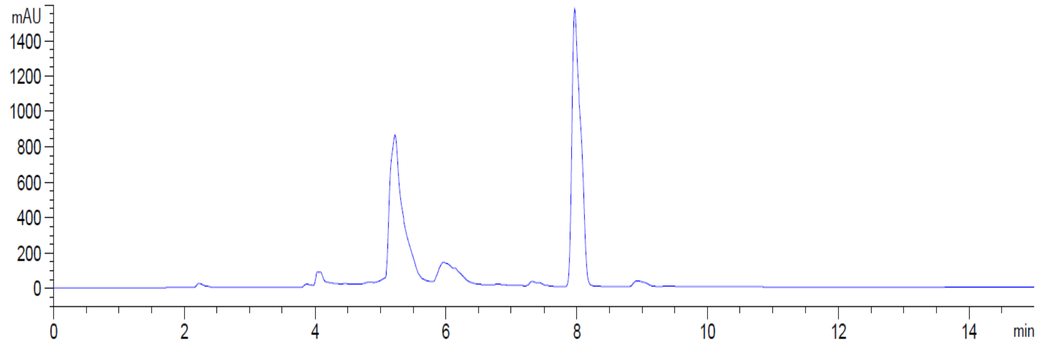

Purified compound HPLC profile

Program B

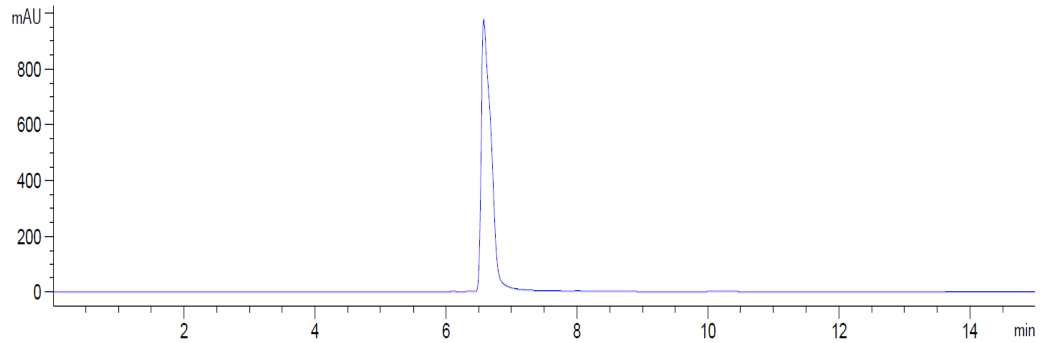

HRMS

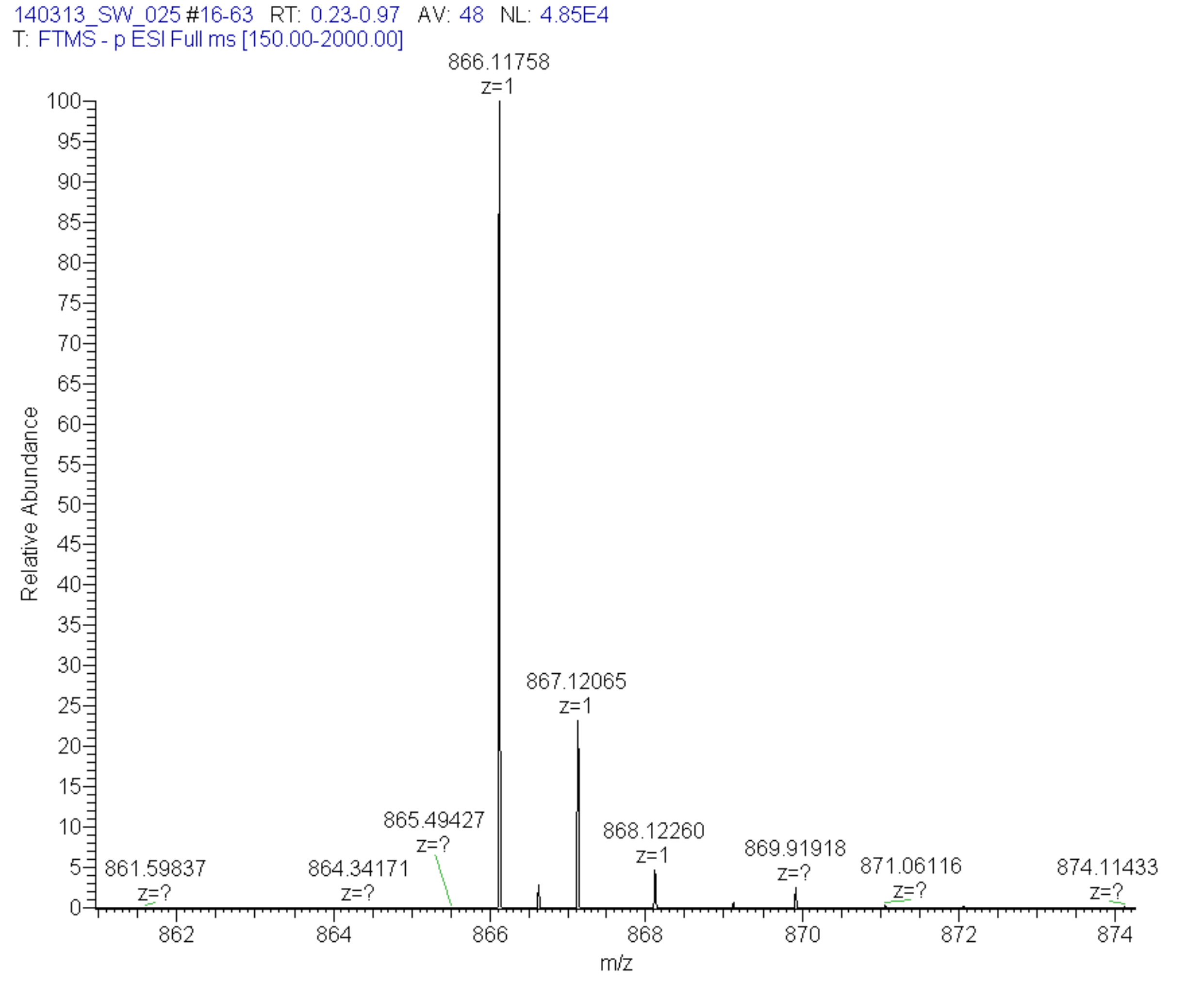
140313_SW_025 #16-63 RT: 0.23-0.97 AV: 48 NL: 4.85E4
T: FTMS - p ESI Full ms [150.00-2000.00]
866.11758 z=1
867.12065 z=1
865.49427 z=?
868.12260 z=1
869.91918 z=?
871.06116 z=?
861.59837 z=?
864.34171 z=?
874.11433 z=?
Relative Abundance
m/z

^{1}H NMR

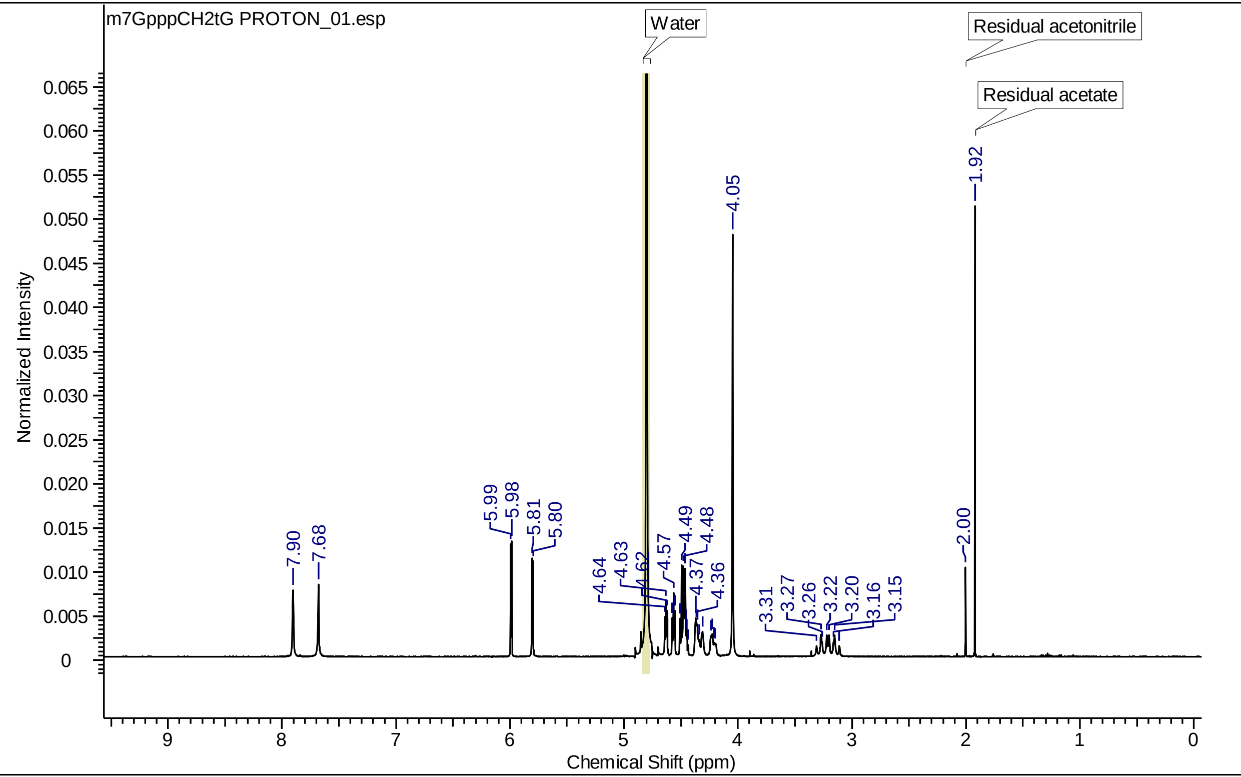
m7GpppCH2tG PROTON_01.esp
Water
Residual acetonitrile
Residual acetate
7.90
7.68
5.99
5.98
5.81
5.80
4.64
4.63
4.62
4.57
4.49
4.48
4.37
4.36
4.05
3.31
3.27
3.26
3.22
3.20
3.16
3.15
2.00
1.92
Normalized Intensity
Chemical Shift (ppm)

^{31}P NMR

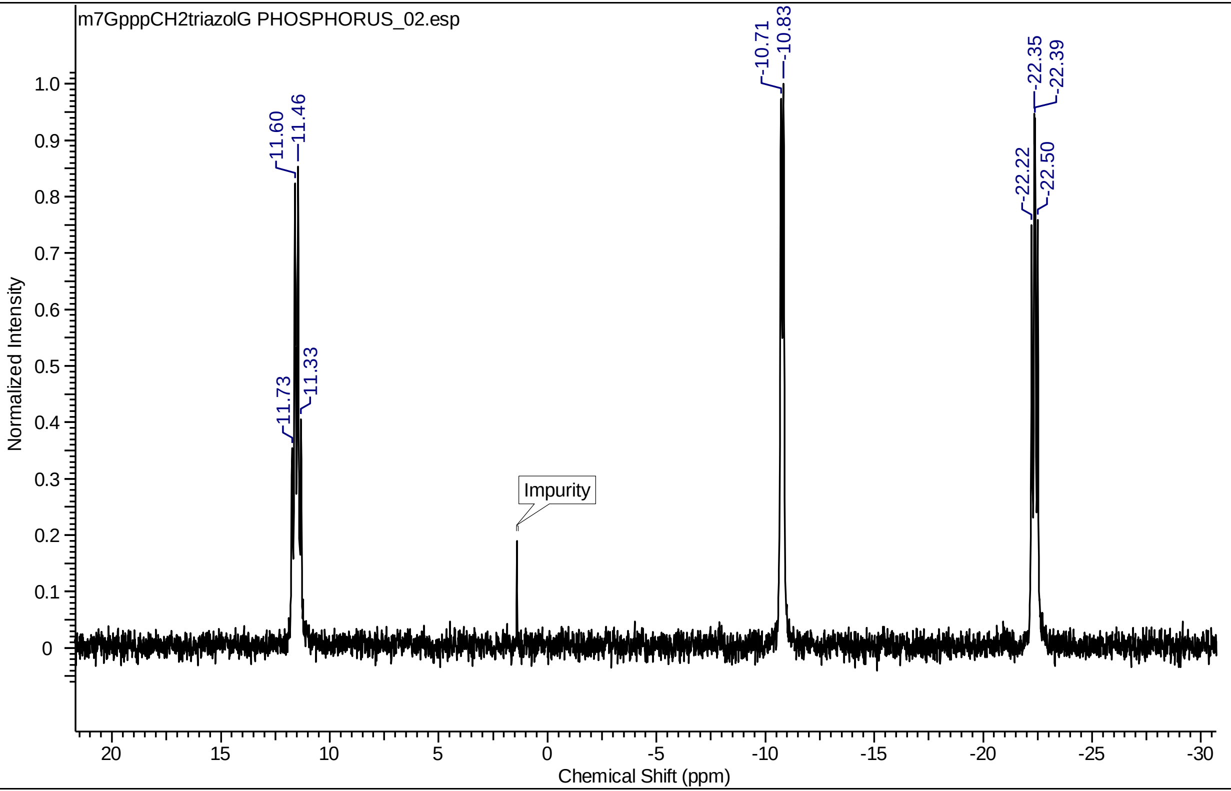

(3a) m^{7}G-triazole-ppG

Structure

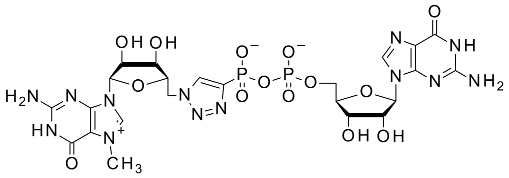

Reaction HPLC profile

Program C

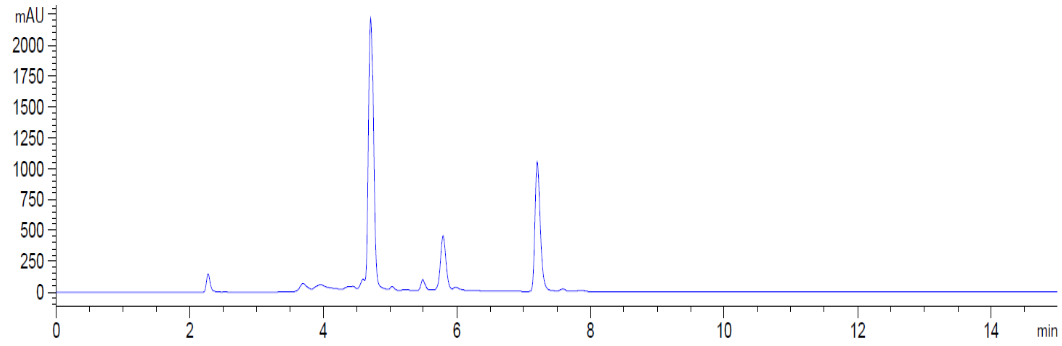

Purified compound HPLC profile

Program B

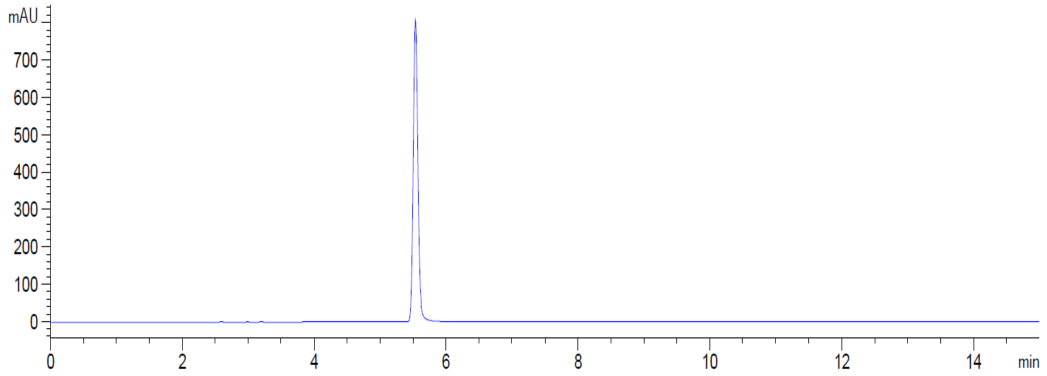

**HRMS**	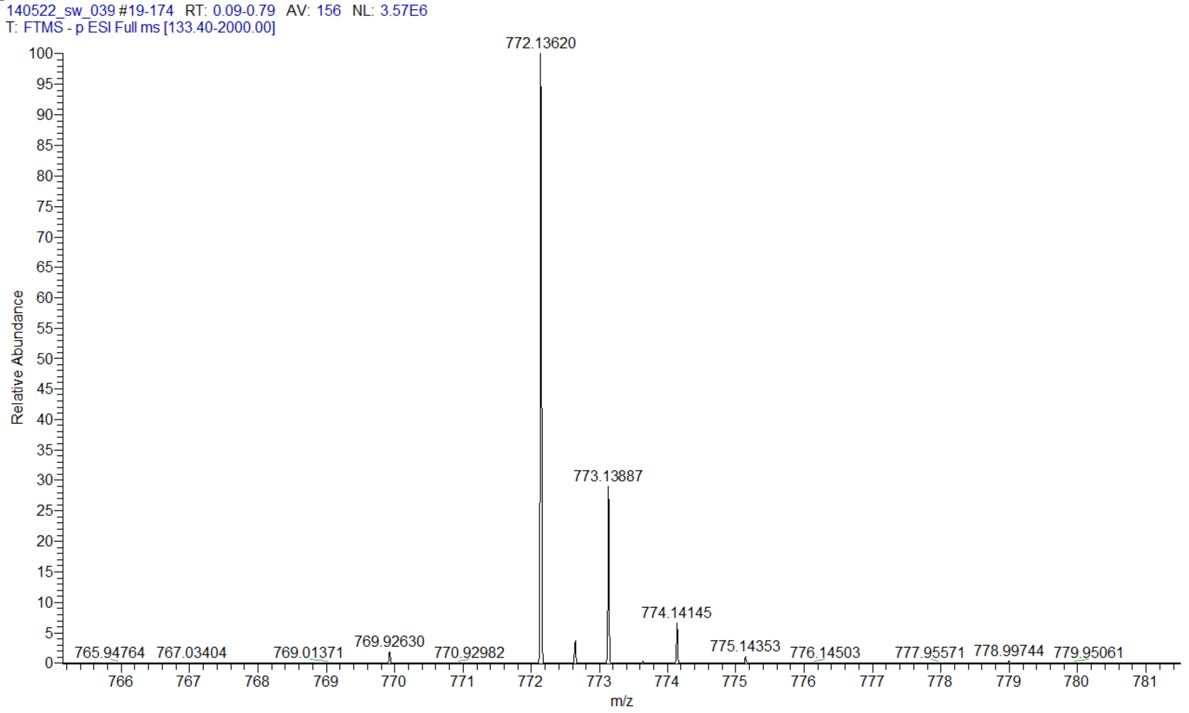
**^{1}H NMR**	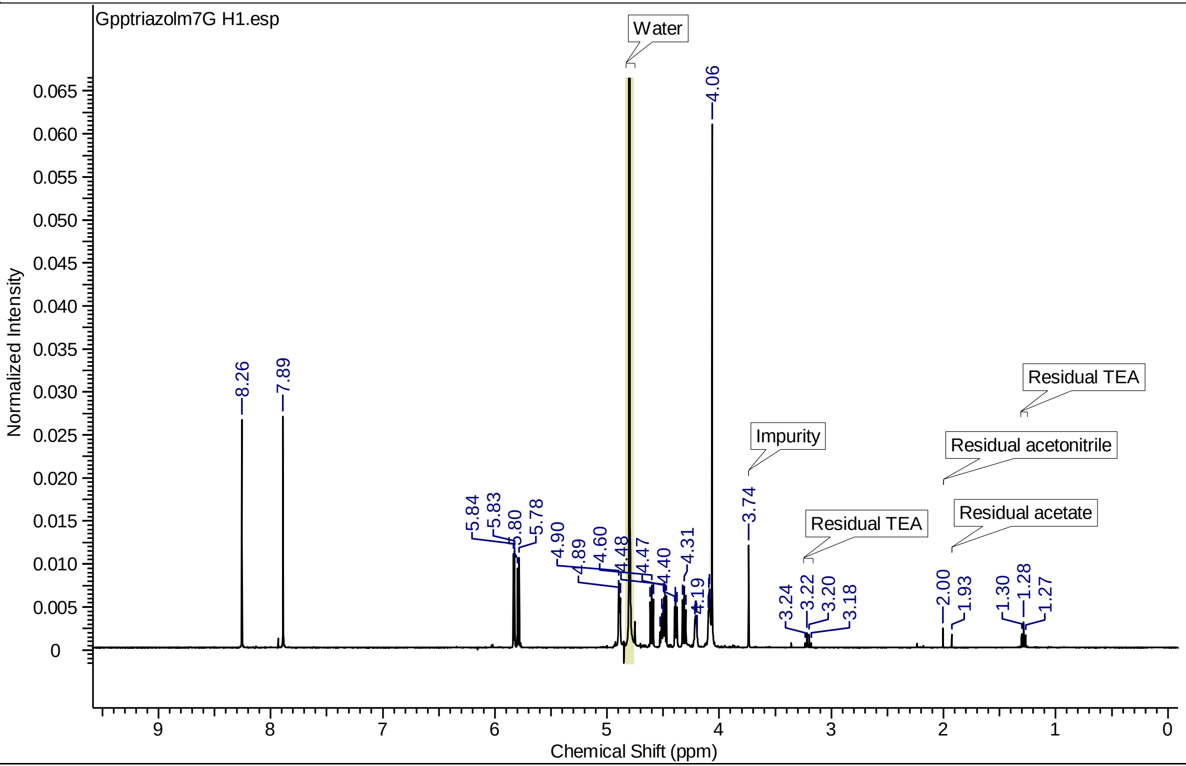

140522_sw_039 #19-174 RT: 0.09-0.79 AV: 156 NL: 3.57E6
T: FTMS - p ESI Full ms [133.40-2000.00]
772.13620
773.13887
774.14145
765.94764
767.03404
769.01371
769.92630
770.92982
775.14353
776.14503
777.95571
778.99744
779.95061
Relative Abundance
m/z

Gpptriazolm7G H1.esp
Water
Impurity
Residual TEA
Residual acetonitrile
Residual acetate
Residual TEA
8.26
7.89
5.84
5.83
5.80
5.78
4.90
4.89
4.60
4.48
4.47
4.40
4.31
4.19
4.06
3.74
3.24
3.22
3.20
3.18
2.00
1.93
1.30
1.28
1.27
Normalized Intensity
Chemical Shift (ppm)

^{31}P NMR	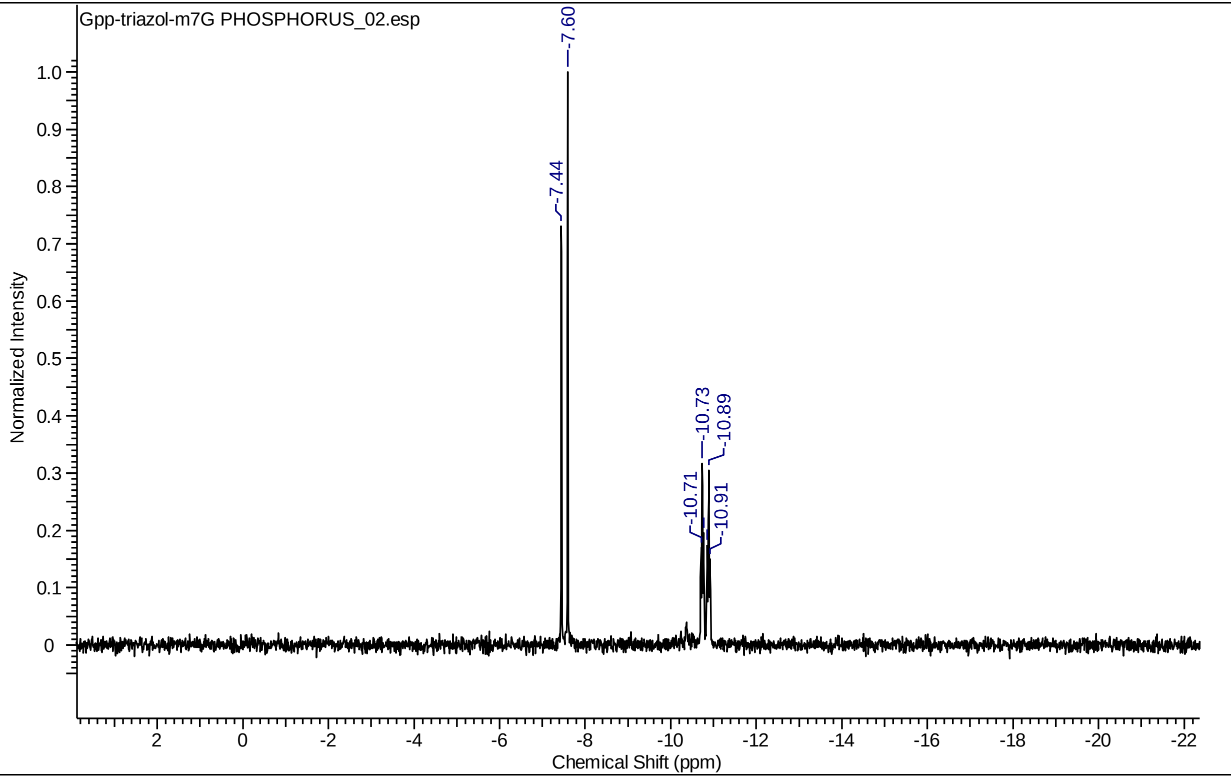

(3b) m^{7}G-triazole-pppG

Structure	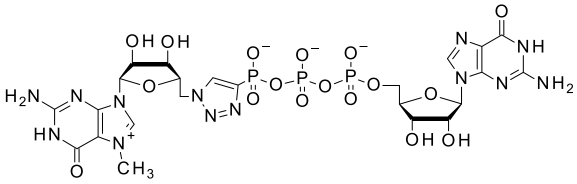
Reaction HPLC profile	Program C<br>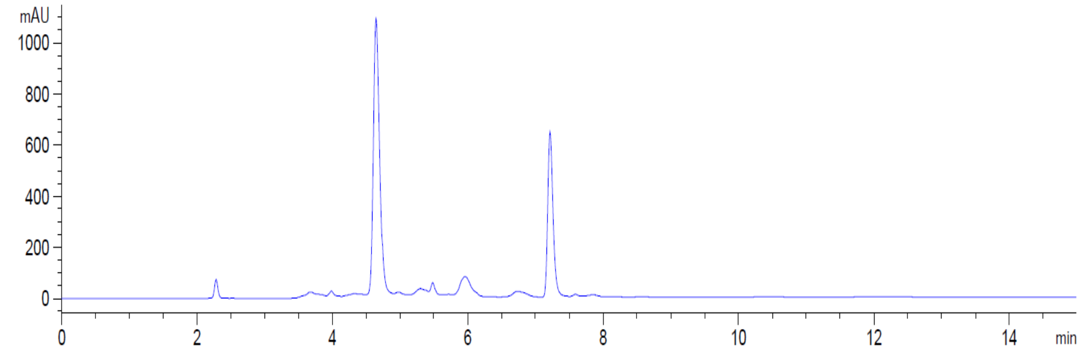
Purified compound HPLC profile	Program B<br>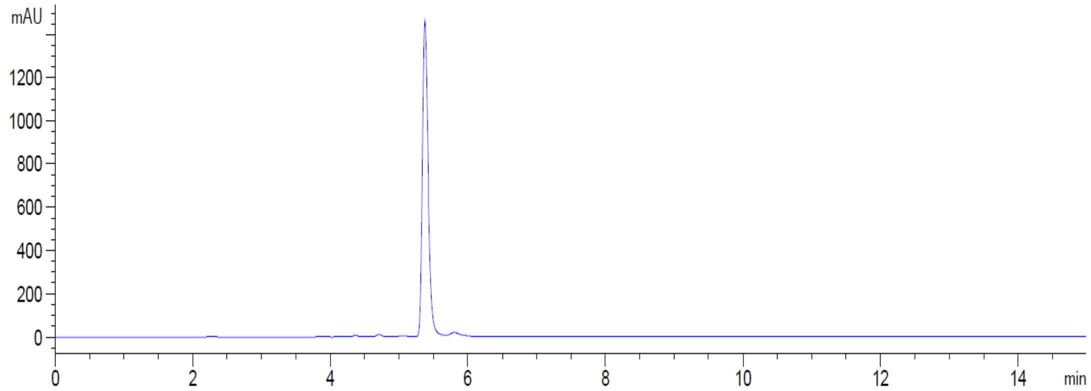

HRMS

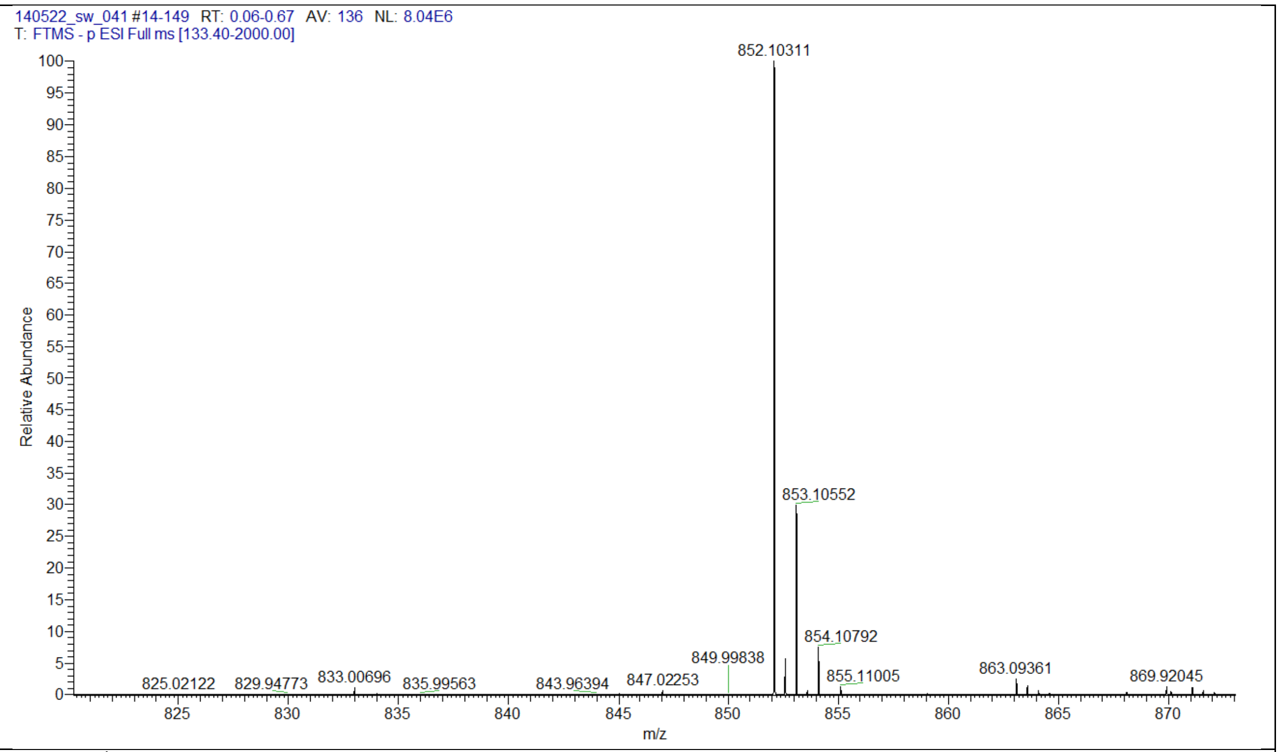

140522_sw_041 #14-149 RT: 0.06-0.67 AV: 136 NL: 8.04E6
T: FTMS - p ESI Full ms [133.40-2000.00]
Relative Abundance
m/z
852.10311
853.10552
854.10792
855.11005
863.09361
869.92045
849.99838
847.02253
843.96394
835.99563
833.00696
829.94773
825.02122

^{1}H NMR

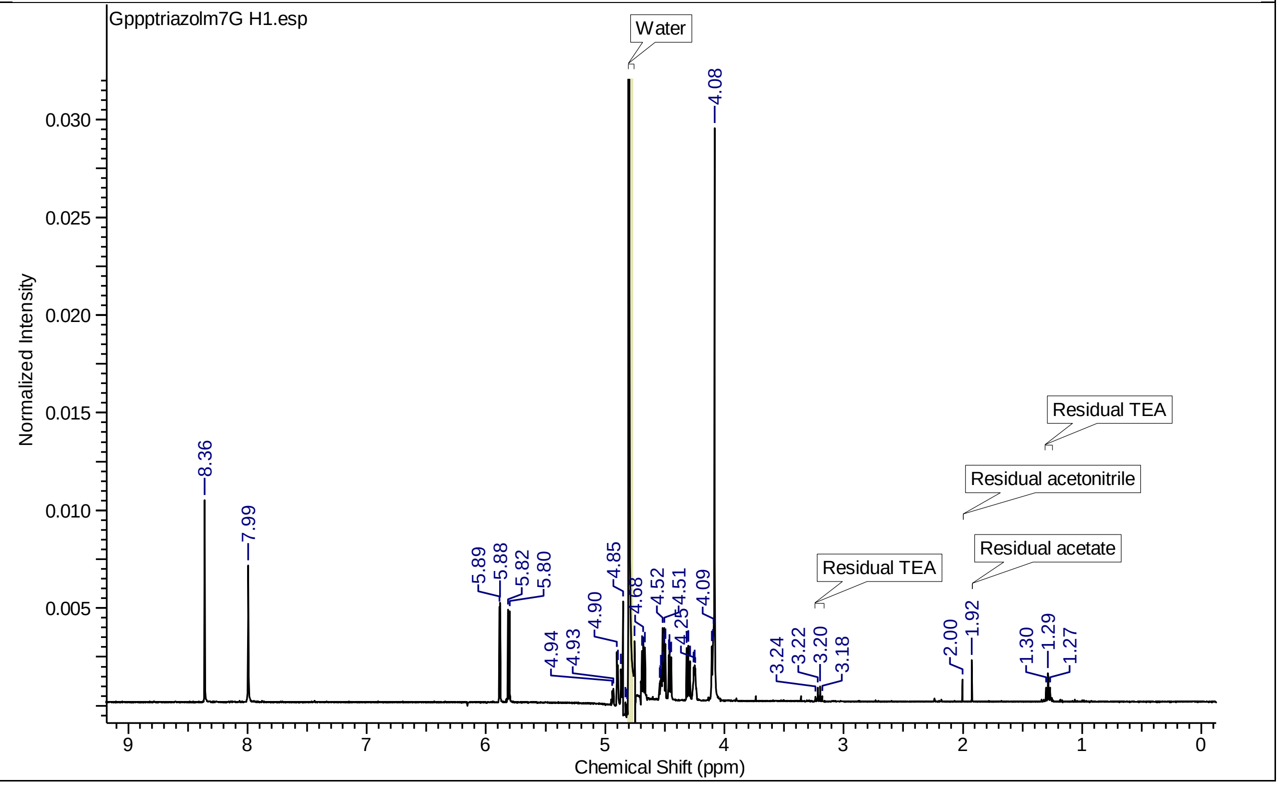

Gppptriazolm7G H1.esp
Water
Residual TEA
Residual acetonitrile
Residual acetate
Residual TEA
Normalized Intensity
Chemical Shift (ppm)
8.36
7.99
5.89
5.88
5.82
5.80
4.94
4.93
4.90
4.85
4.68
4.52
4.51
4.25
4.09
4.08
3.24
3.22
3.20
3.18
2.00
1.92
1.30
1.29
1.27

^{31}P NMR	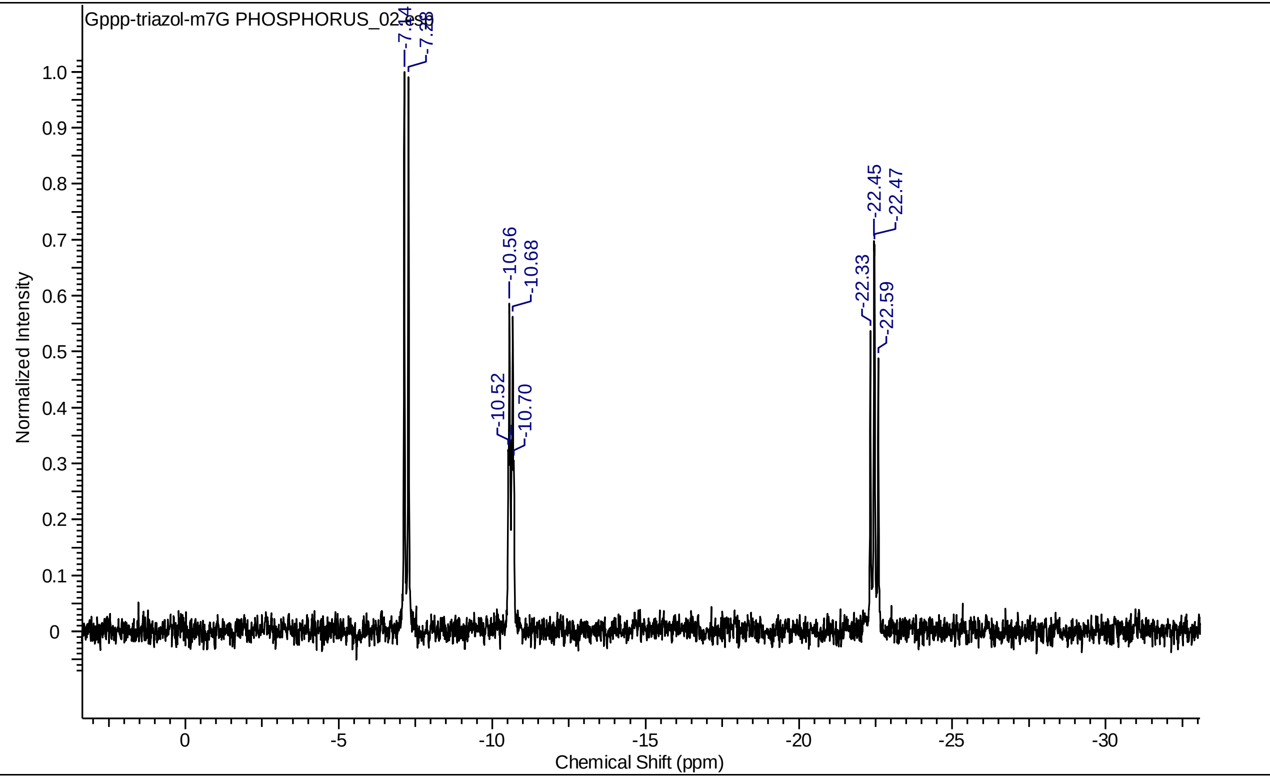

(3c) m^{7}Gpp-triazole-G

Structure	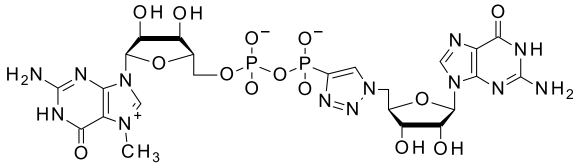
Reaction HPLC profile	Program C 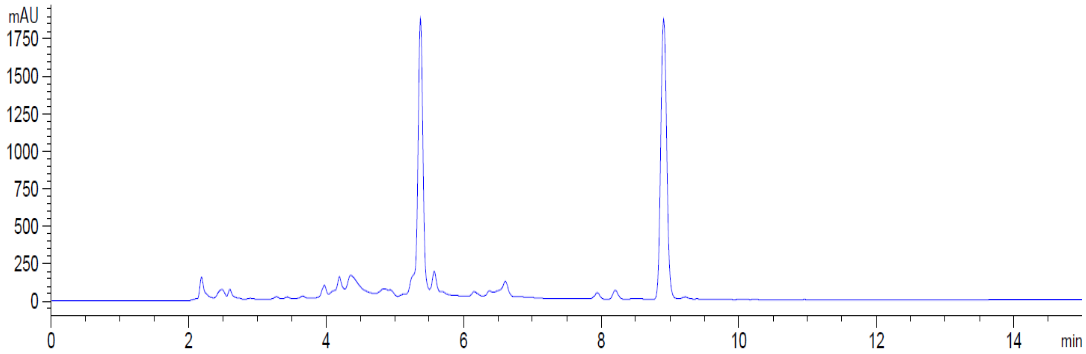
Purified compound HPLC profile	Program B 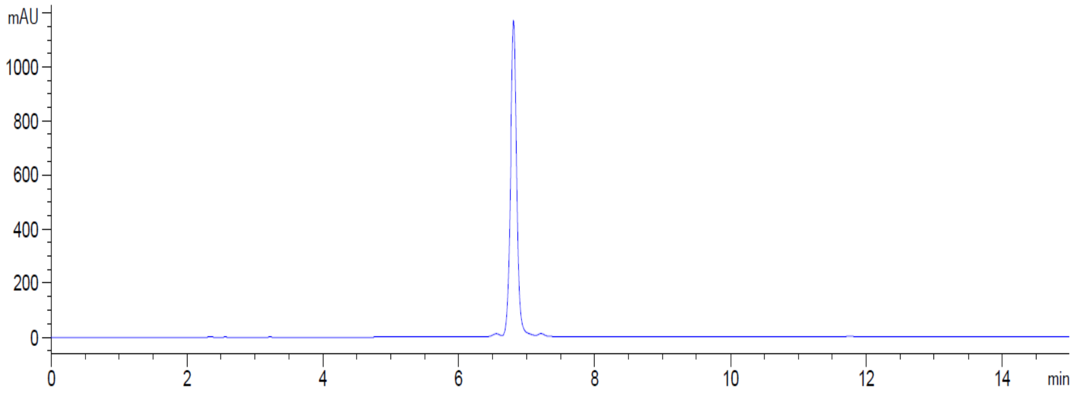

HRMS

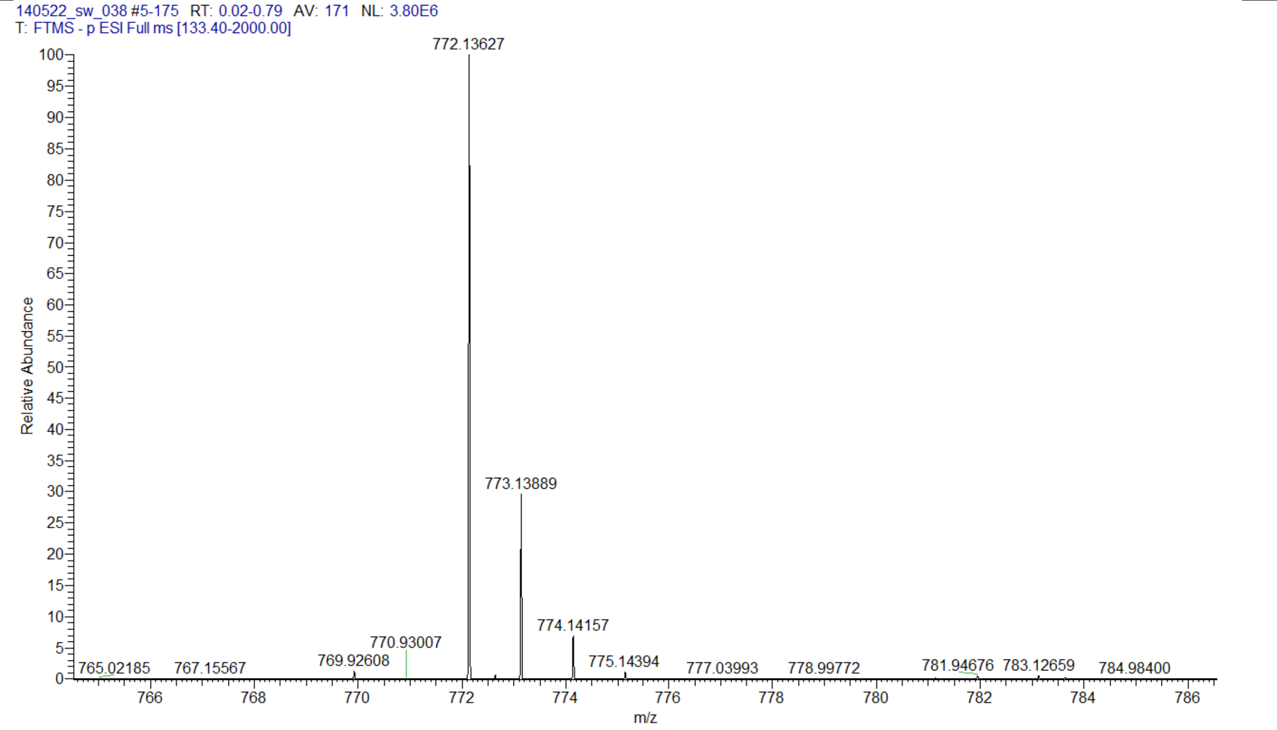

140522_sw_038 #5-175 RT: 0.02-0.79 AV: 171 NL: 3.80E6
T: FTMS - p ESI Full ms [133.40-2000.00]
772.13627
773.13889
774.14157
775.14394
765.02185
767.15567
769.92608
770.93007
777.03993
778.99772
781.94676
783.12659
784.98400
Relative Abundance
m/z

^{1}H NMR

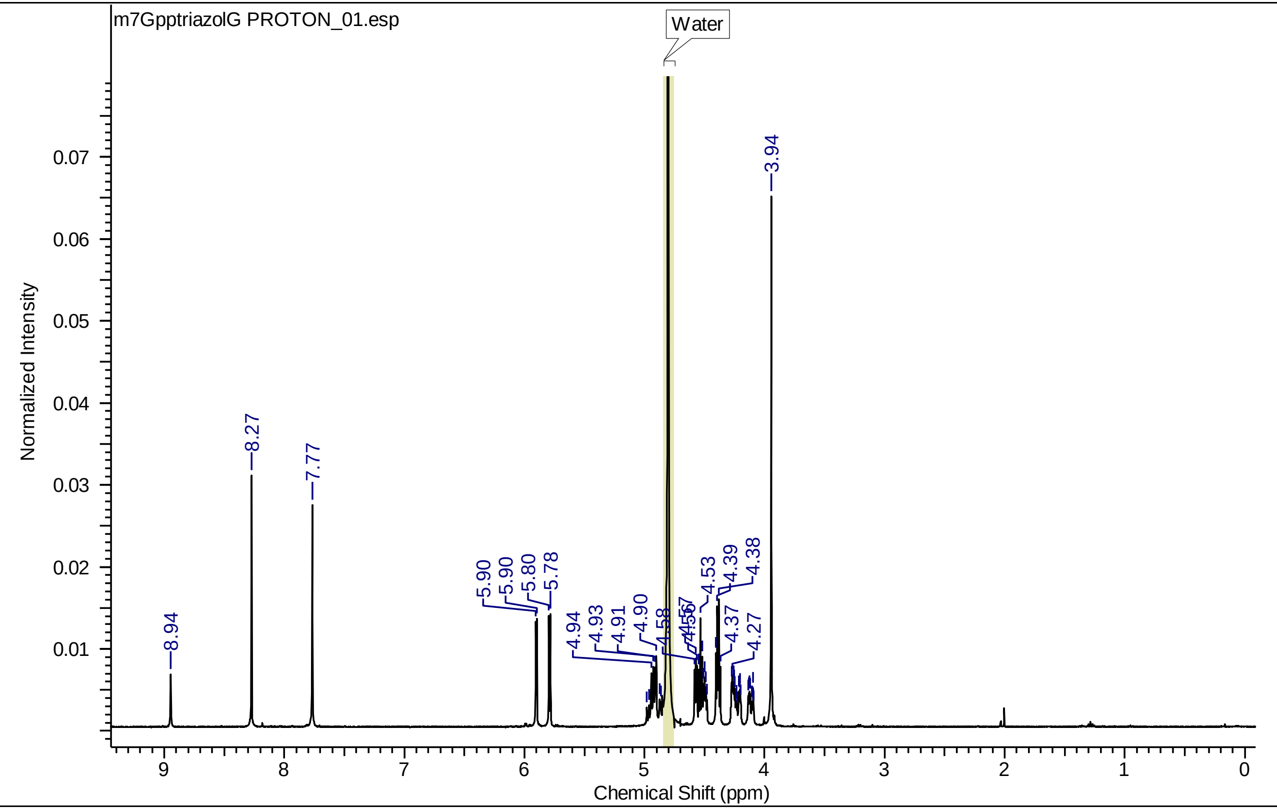

m7GpptriazolG PROTON_01.esp
Water
Normalized Intensity
Chemical Shift (ppm)
8.94
8.27
7.77
5.90
5.90
5.80
5.78
4.94
4.93
4.91
4.90
4.53
4.39
4.38
4.37
4.27
3.94

^{31}P NMR	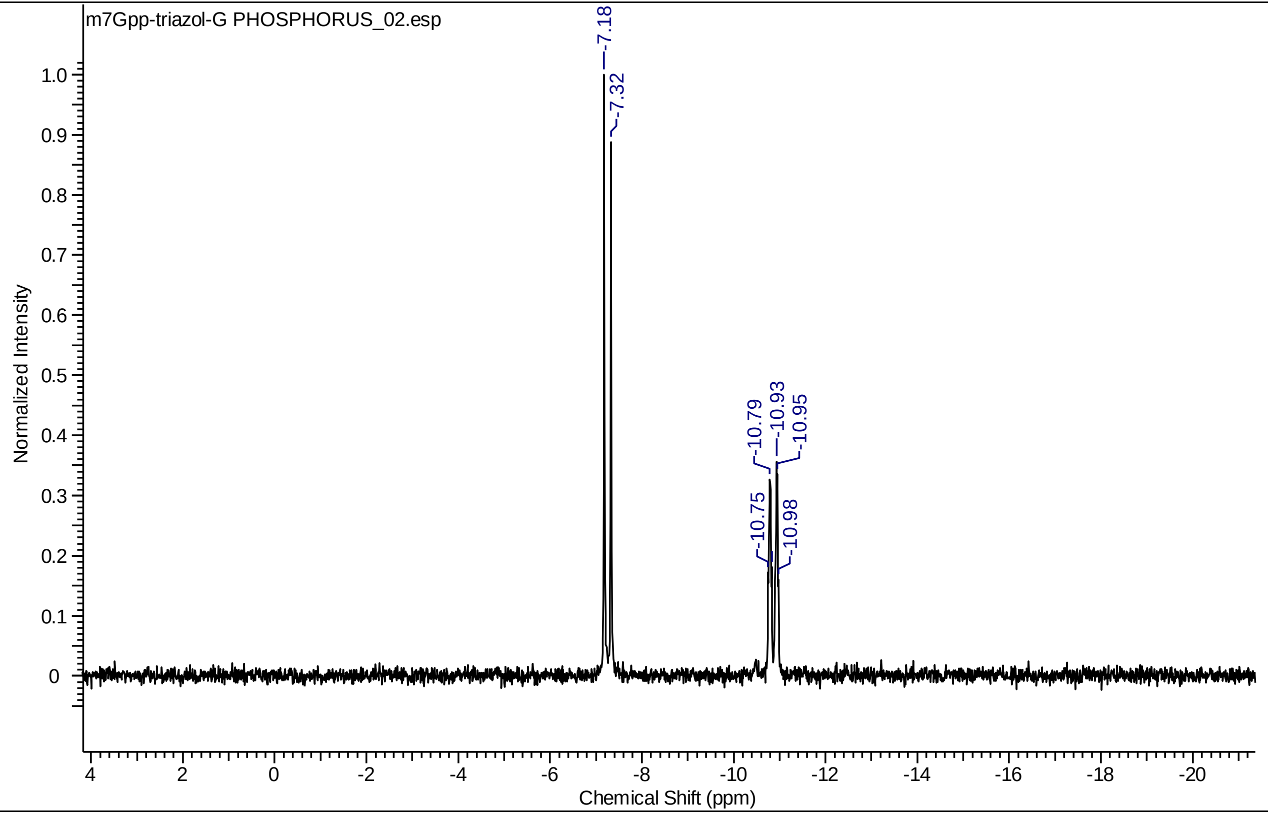

(3d) m^{7}Gppp-triazole-G

Structure	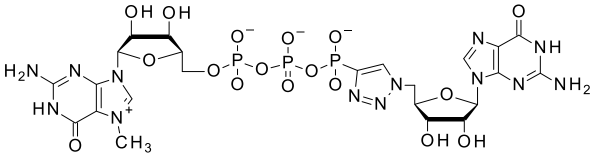
Reaction HPLC profile	Program C<br>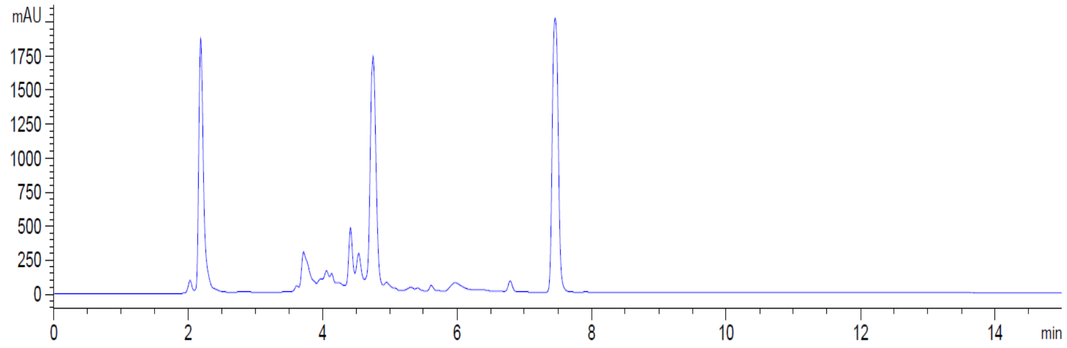
Purified compound HPLC profile	Program B<br>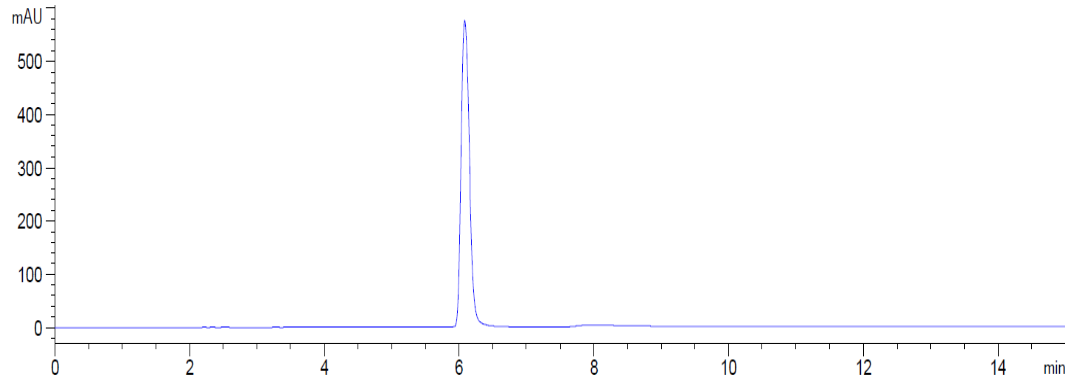

HRMS

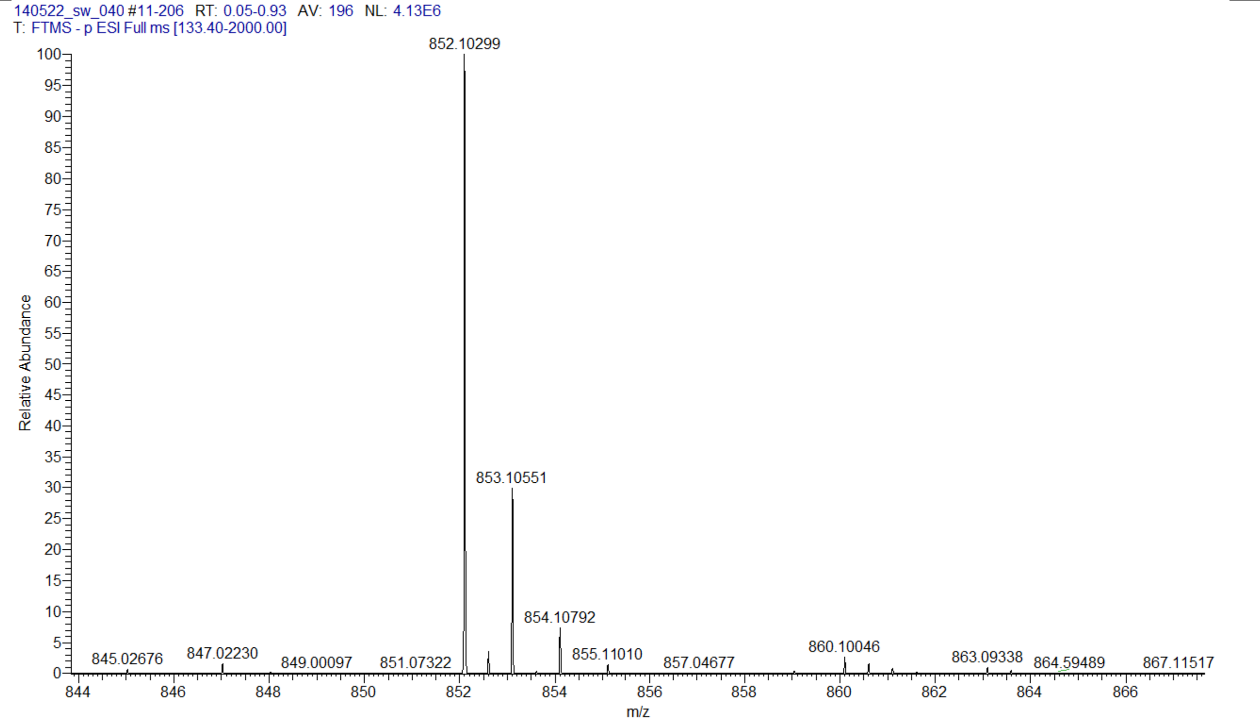

140522_sw_040 #11-206 RT: 0.05-0.93 AV: 196 NL: 4.13E6
T: FTMS - p ESI Full ms [133.40-2000.00]
852.10299
853.10551
854.10792
855.11010
845.02676
847.02230
849.00097
851.07322
857.04677
860.10046
863.09338
864.59489
867.11517
Relative Abundance
m/z

^{1}H NMR

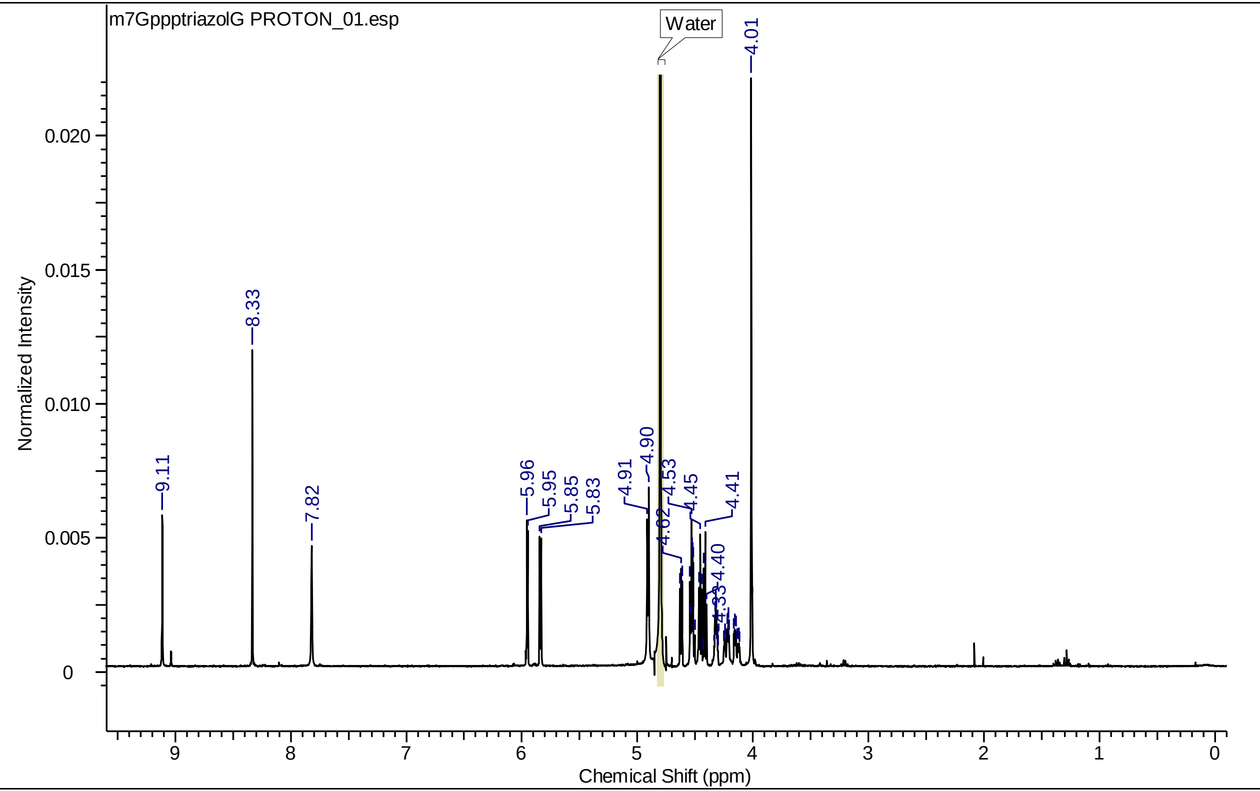

m7GppptriazolG PROTON_01.esp
Water
9.11
8.33
7.82
5.96
5.95
5.85
5.83
4.91
4.90
4.62
4.53
4.45
4.41
4.40
4.33
4.01
Normalized Intensity
Chemical Shift (ppm)

^{31}P NMR

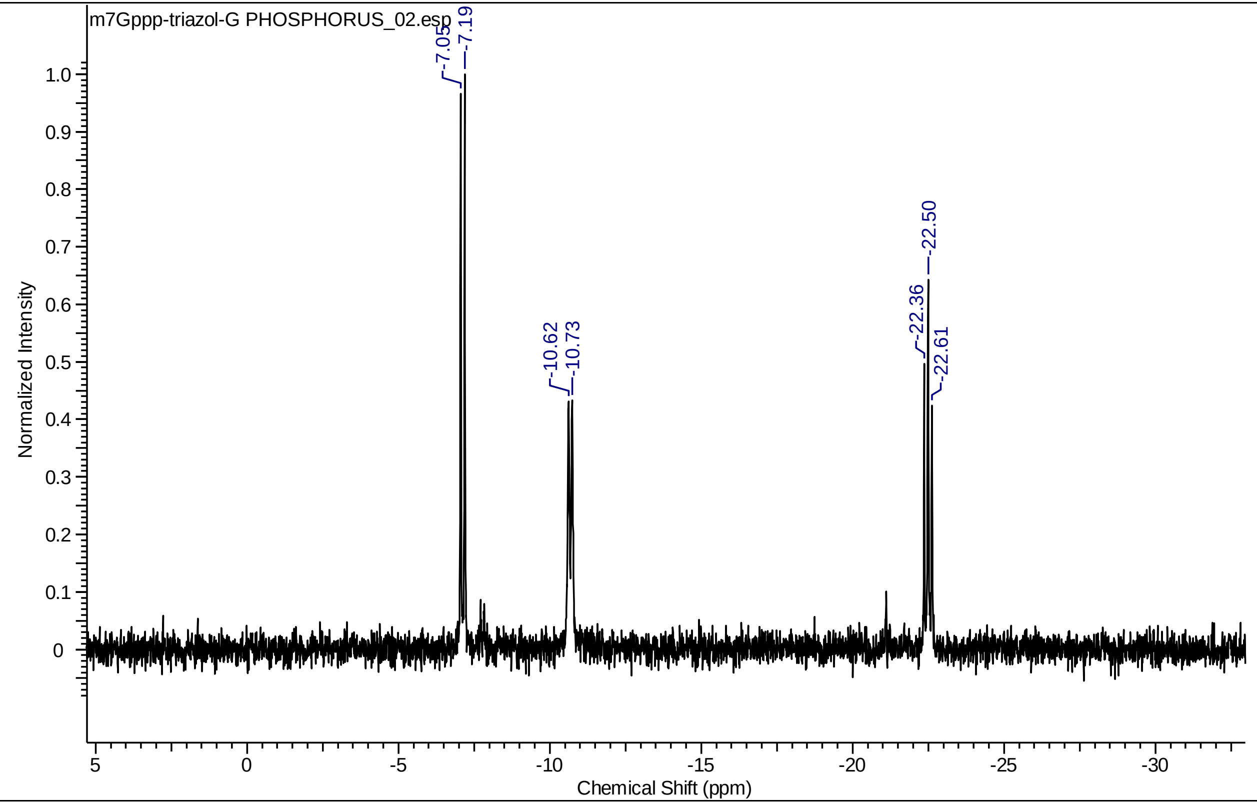

(3e) m$_2$$^{7,2'\text{-}O}$Gppp-triazole-G

Structure

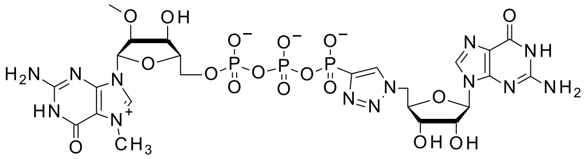

Reaction HPLC profile

Program B; before and after the reaction

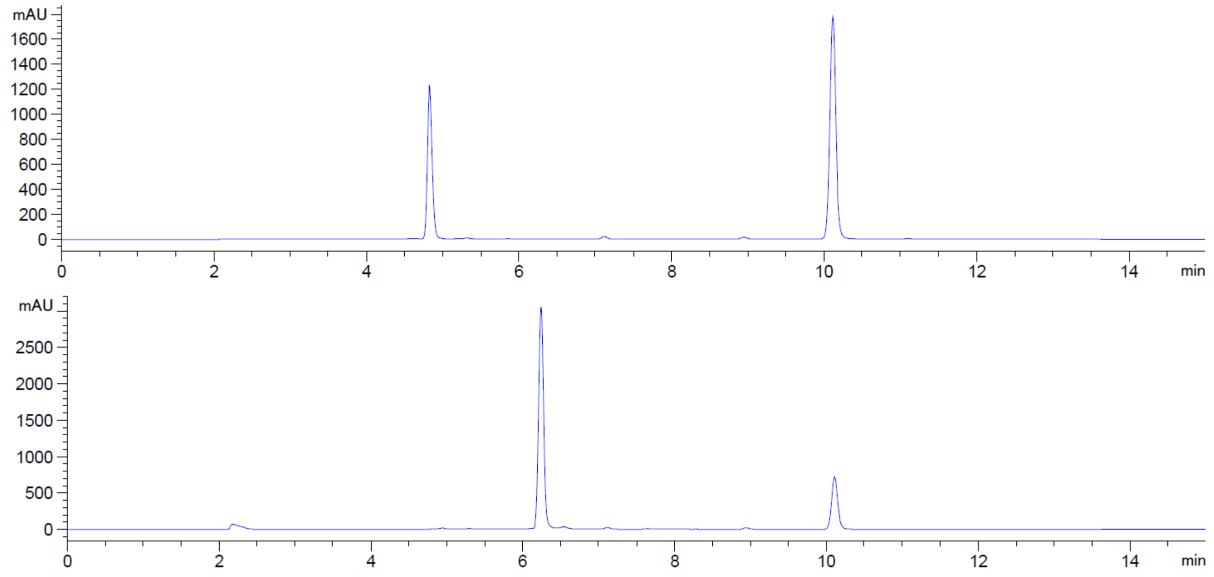

Purified compound HPLC profile

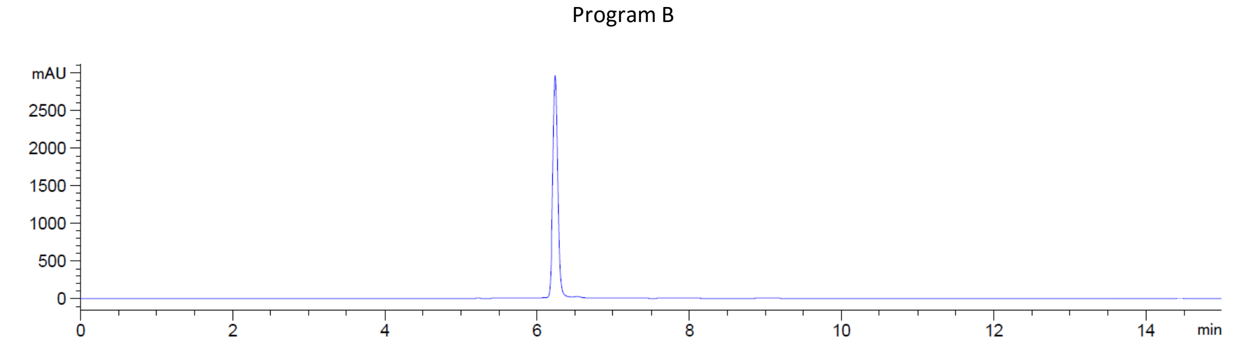

HRMS

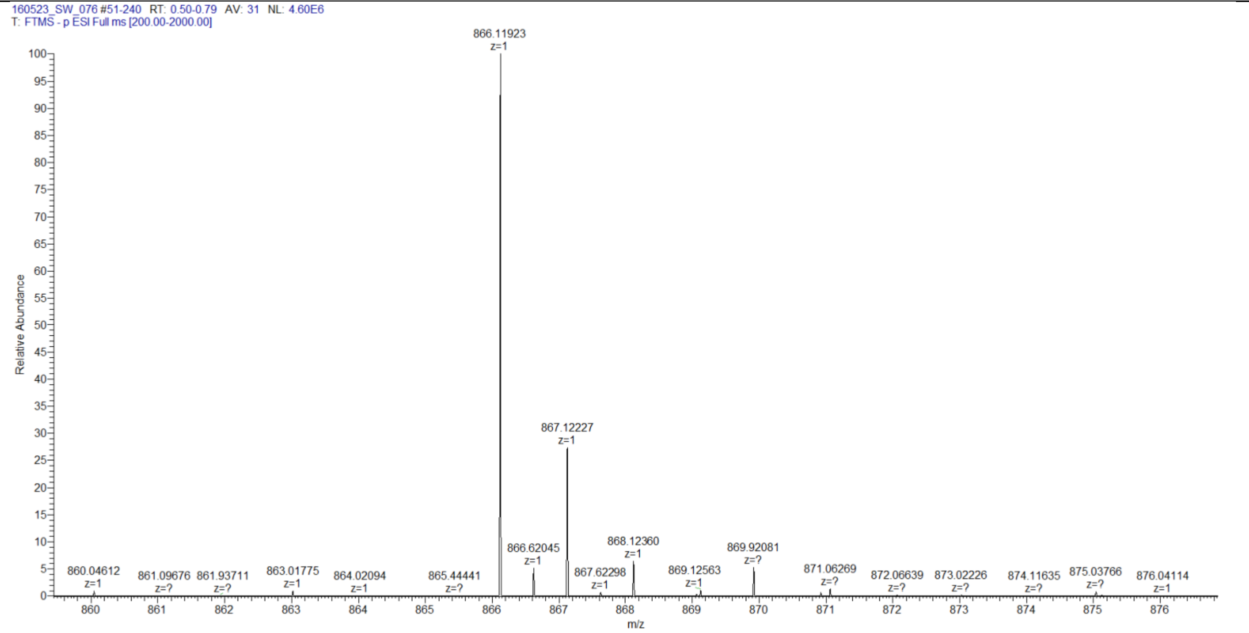

^{1}H NMR

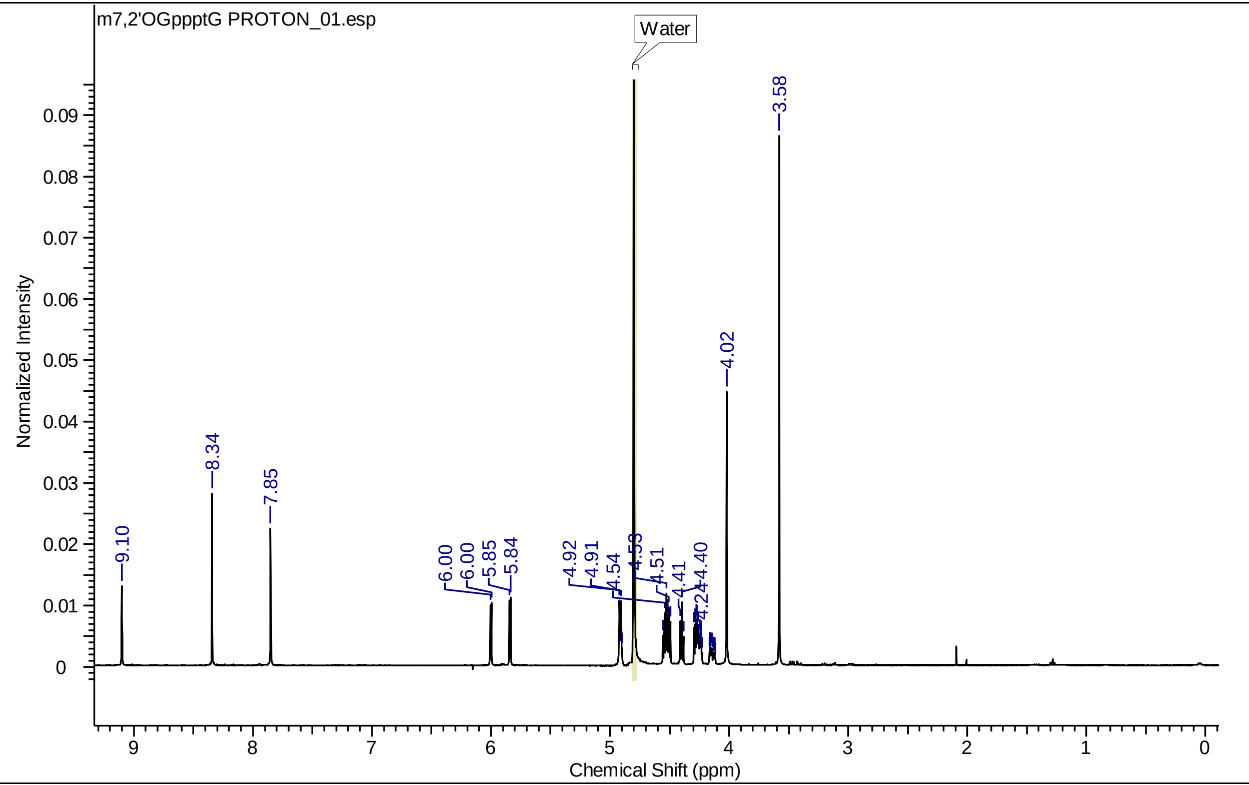

**^{31}P NMR**	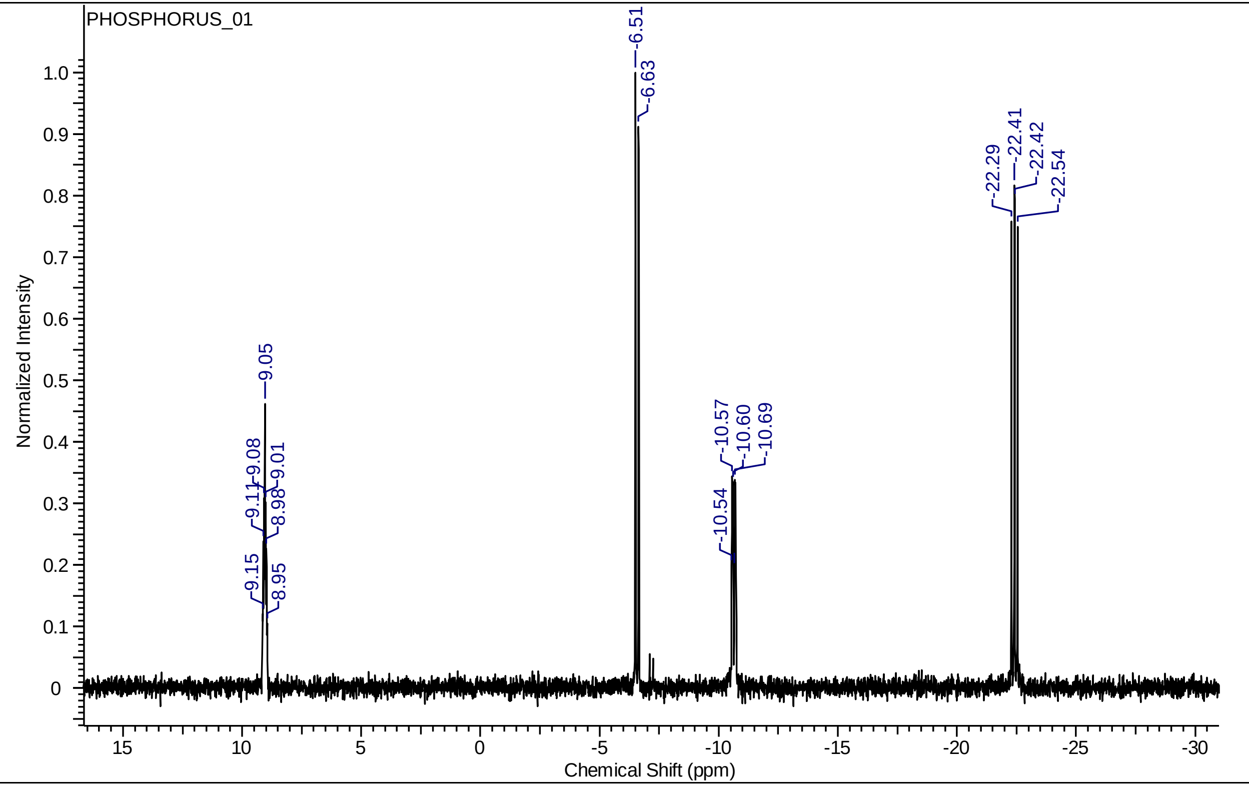

(4a) m^{7}G-triazole-CH$_2$OppG

**Structure**	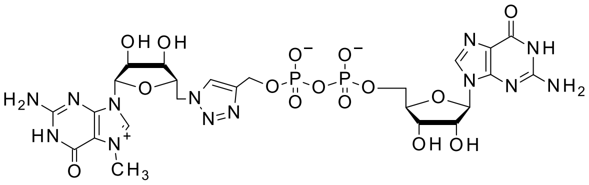
**Reaction HPLC profile**	Program C; before and after the reaction 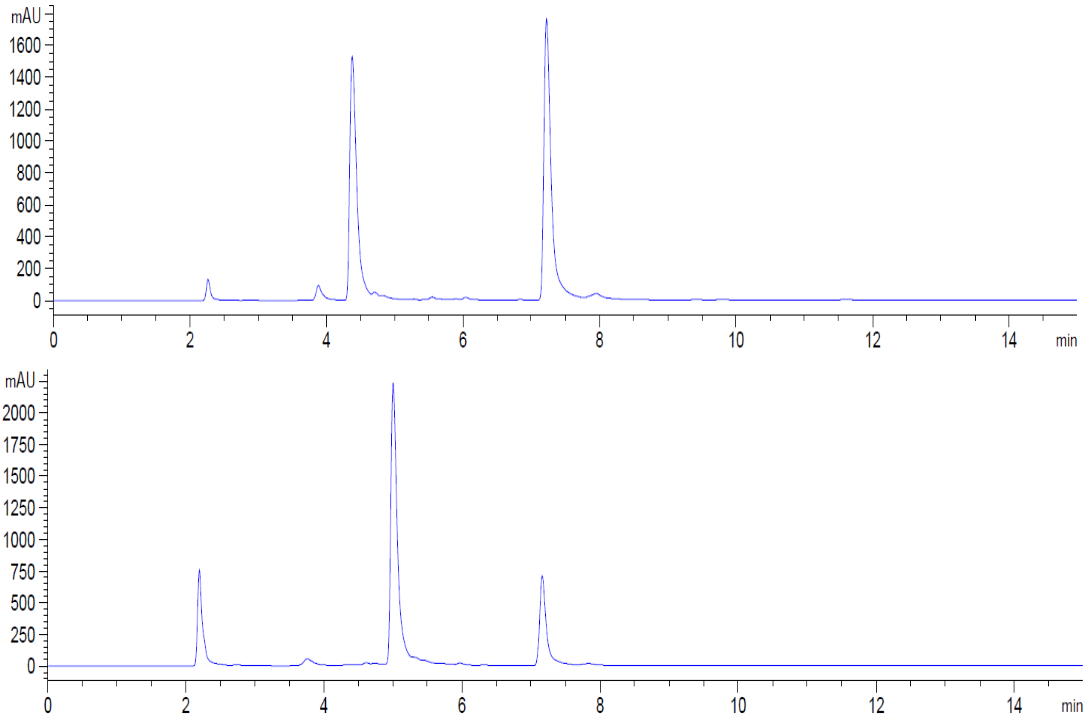

**Purified compound HPLC profile**	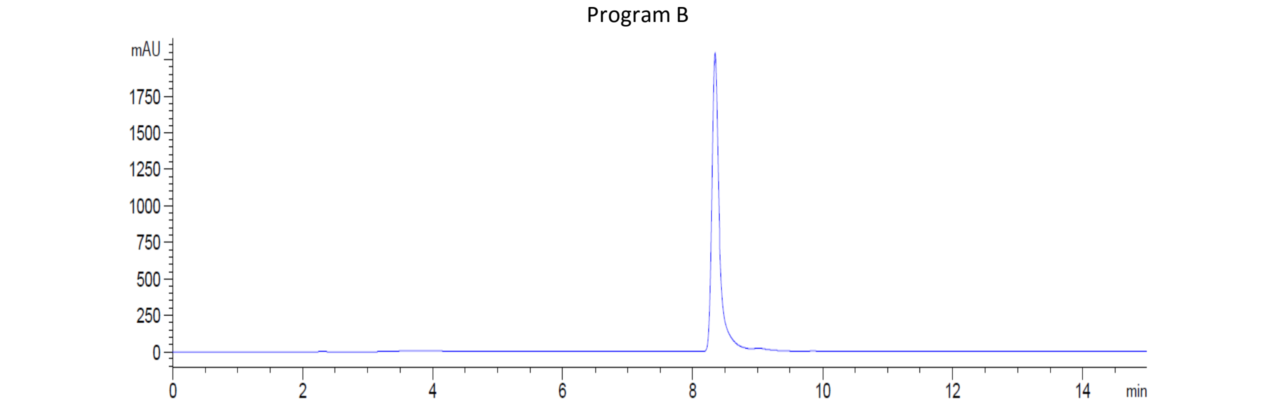
**HRMS**	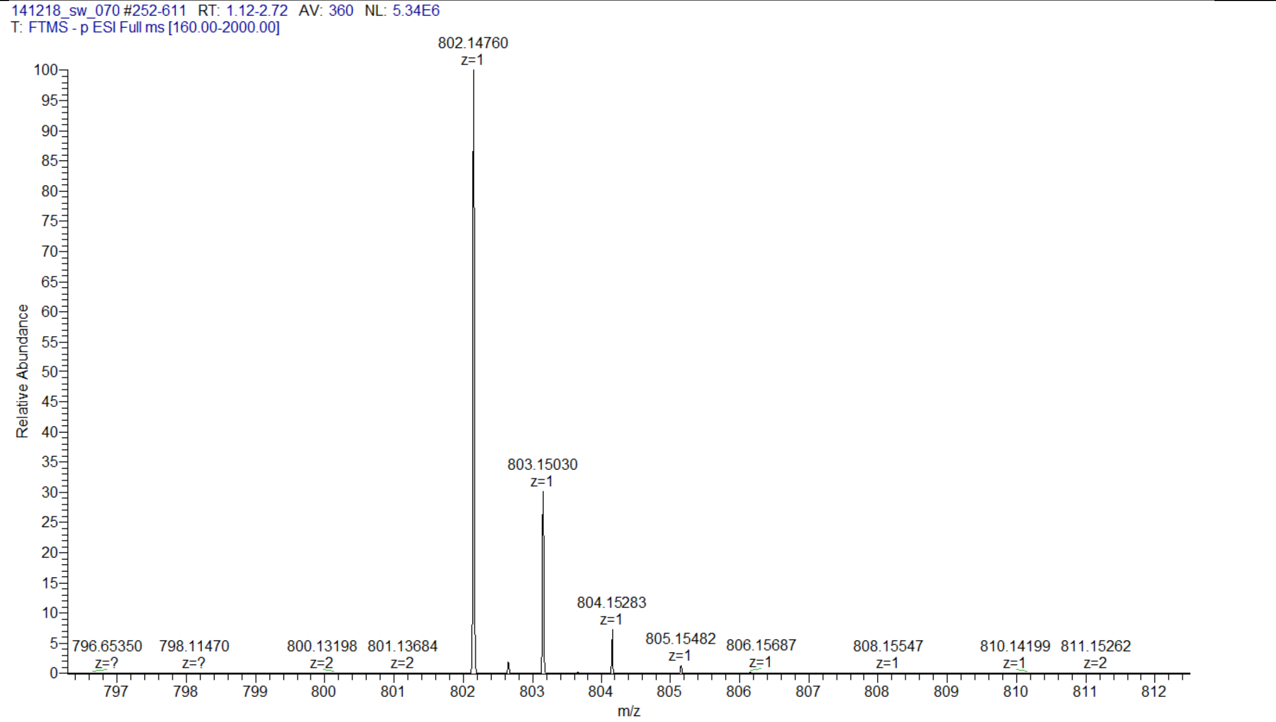
**^{1}H NMR**	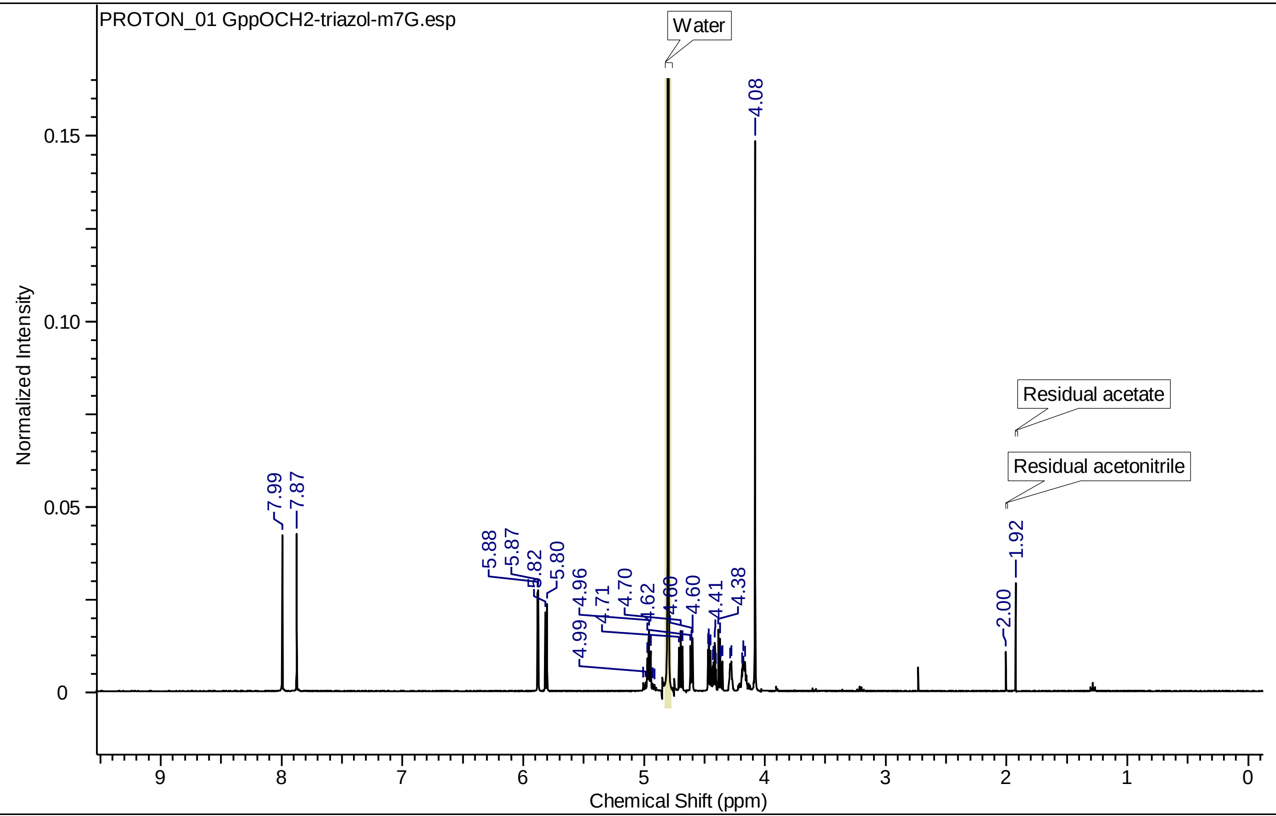

^{31}P NMR

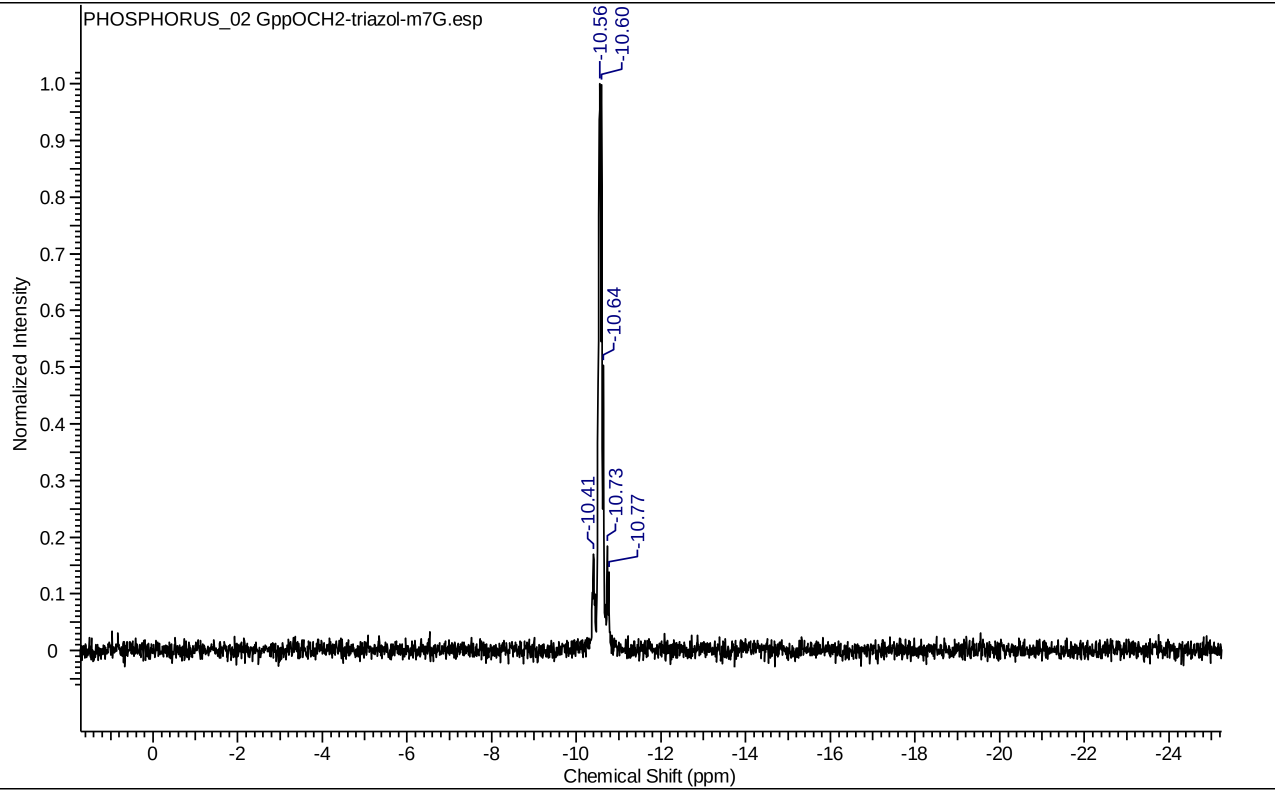

(4b) m^{7}G-triazole-CH$_2$OpppG

Structure

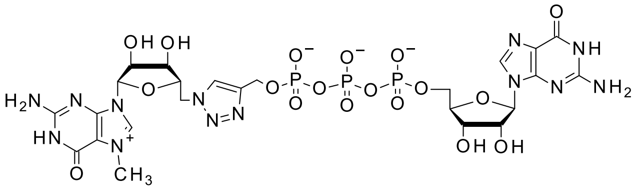

Reaction HPLC profile

Program C; before and after the reaction

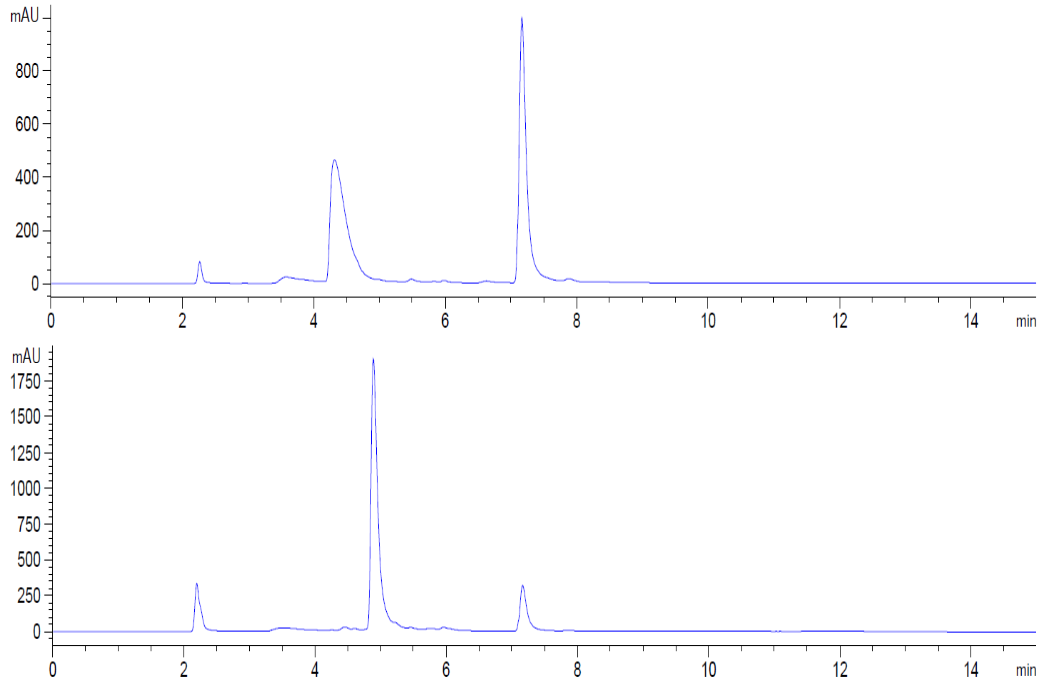

Purified compound HPLC profile

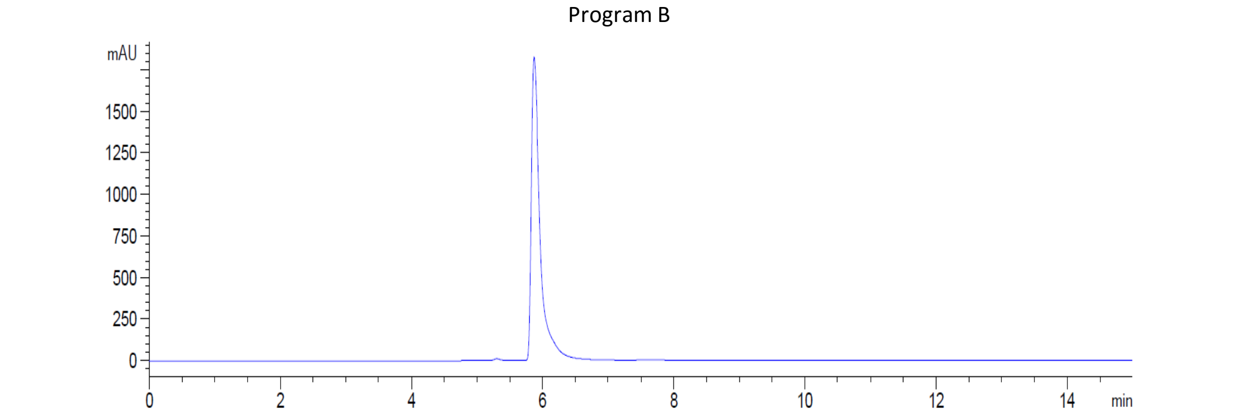

HRMS

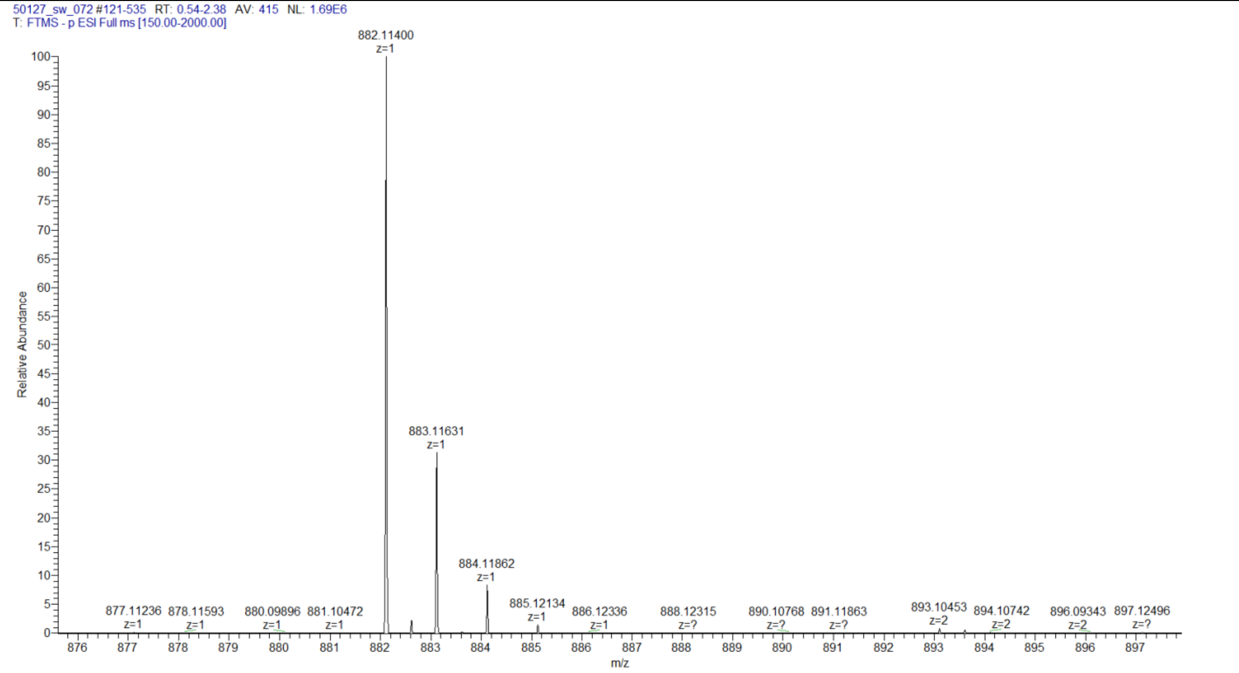

^{1}H NMR

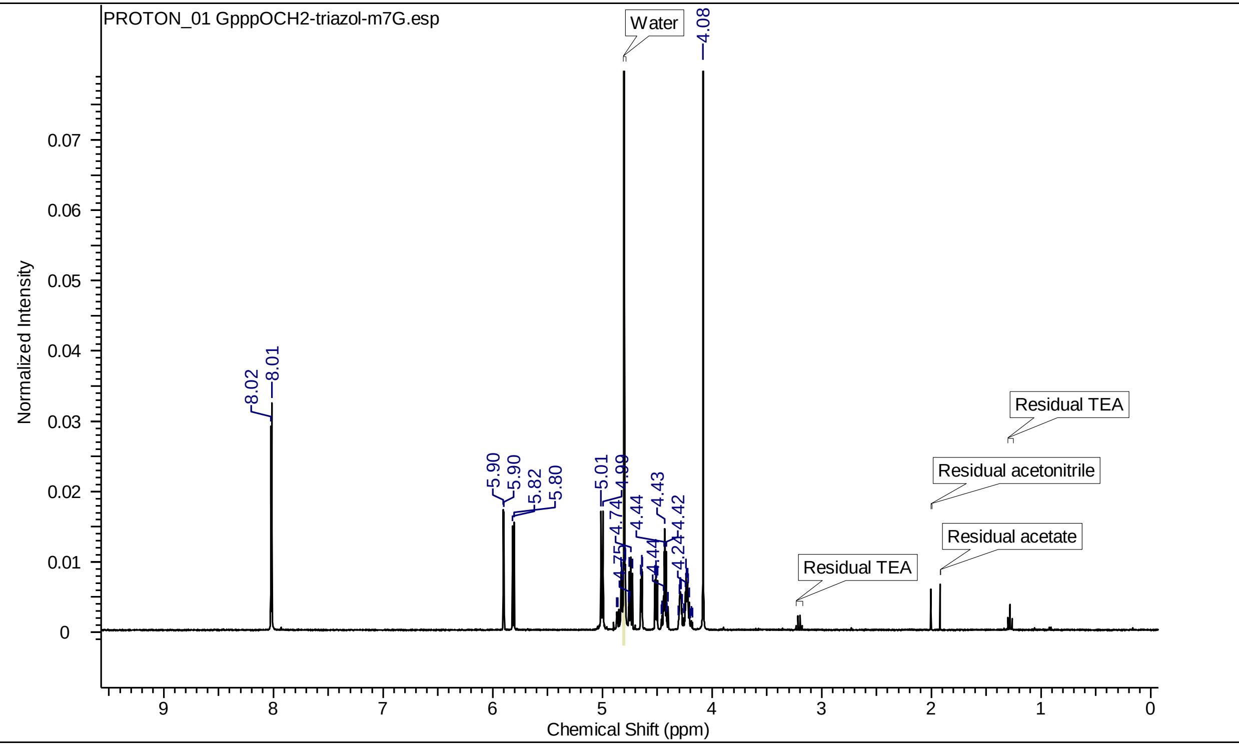

^{31}P NMR

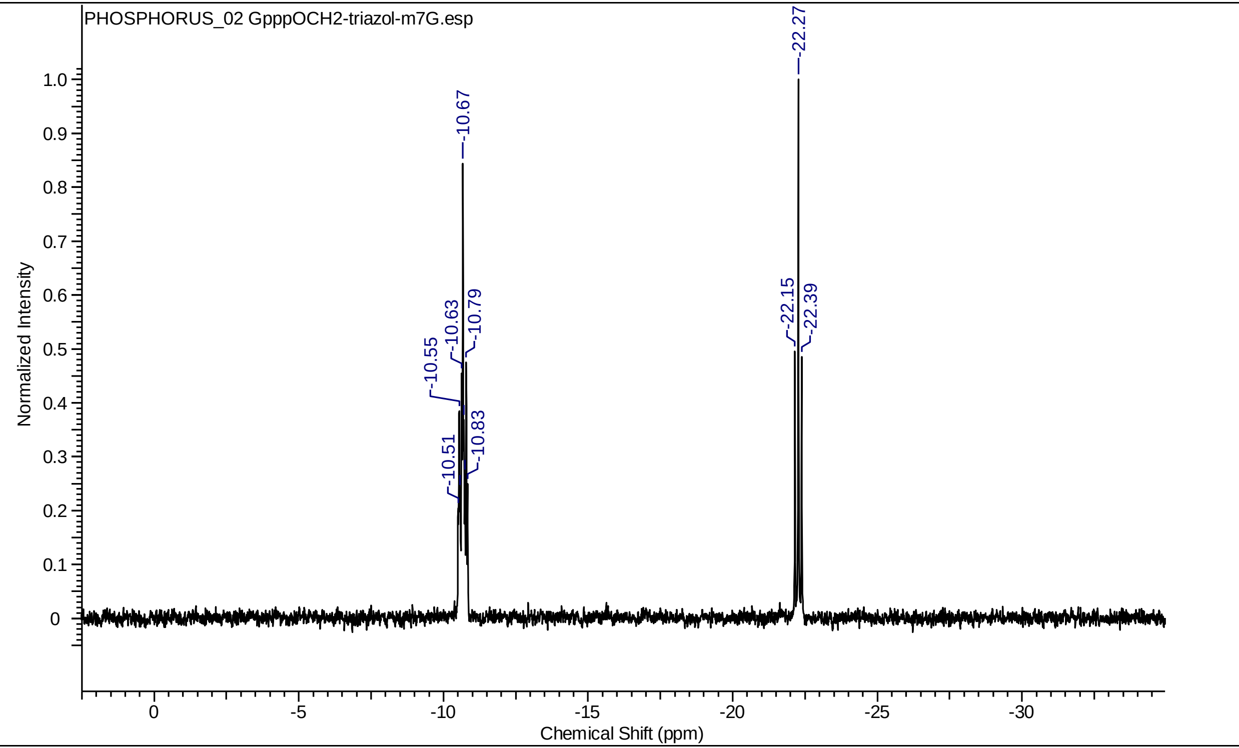

(4c) m^{7}GppOCH$_2$-triazole-G

Structure

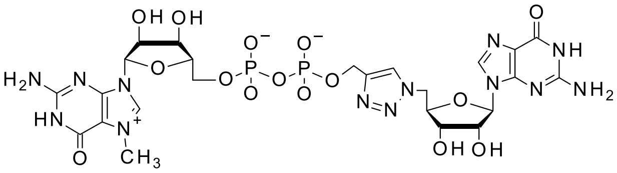

Reaction HPLC profile

Program C; before and after the reaction

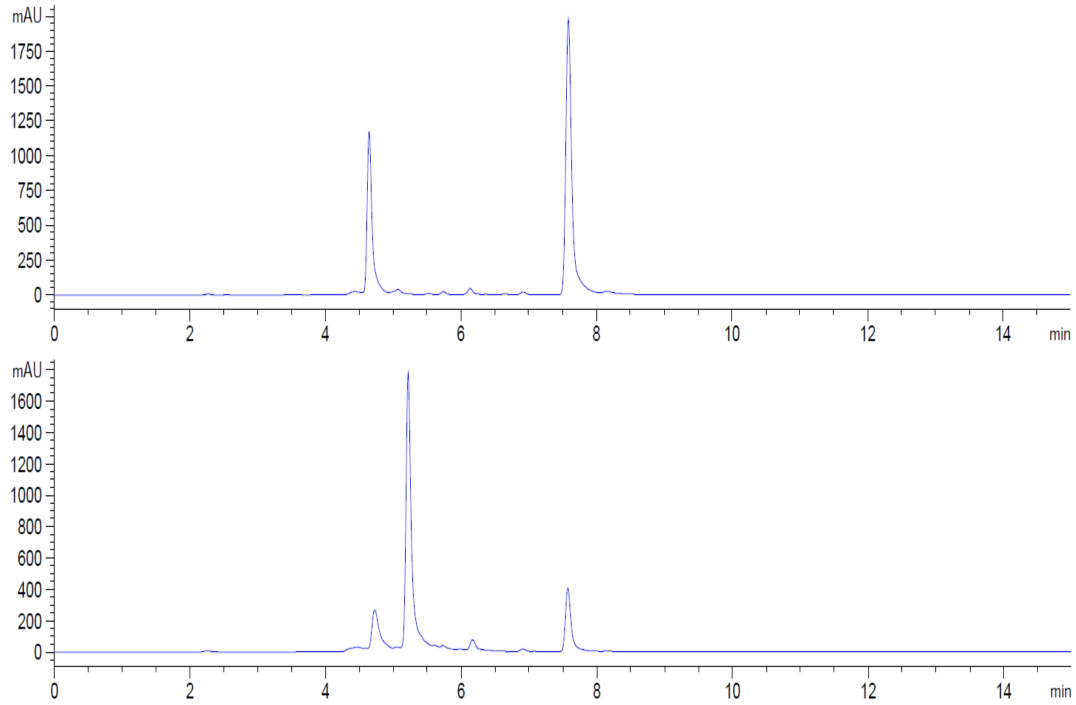

Purified compound HPLC profile

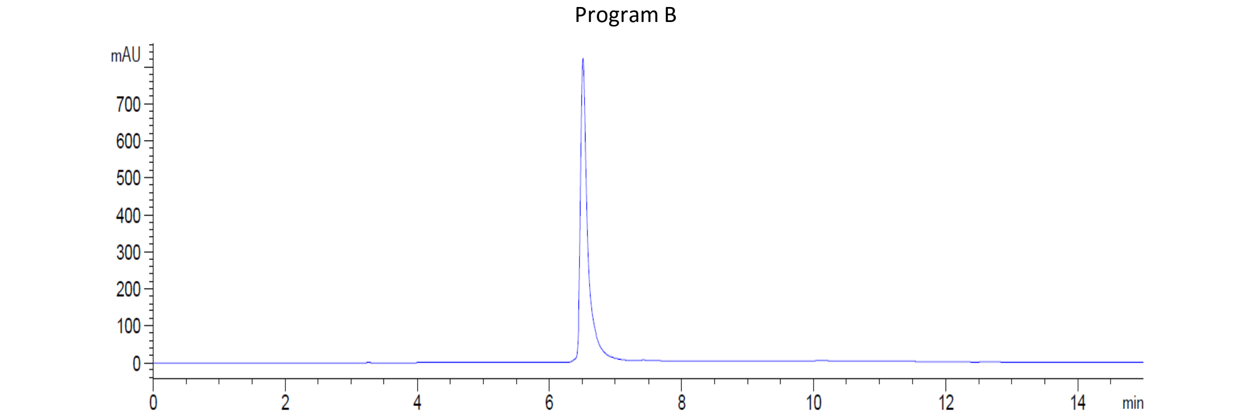

HRMS

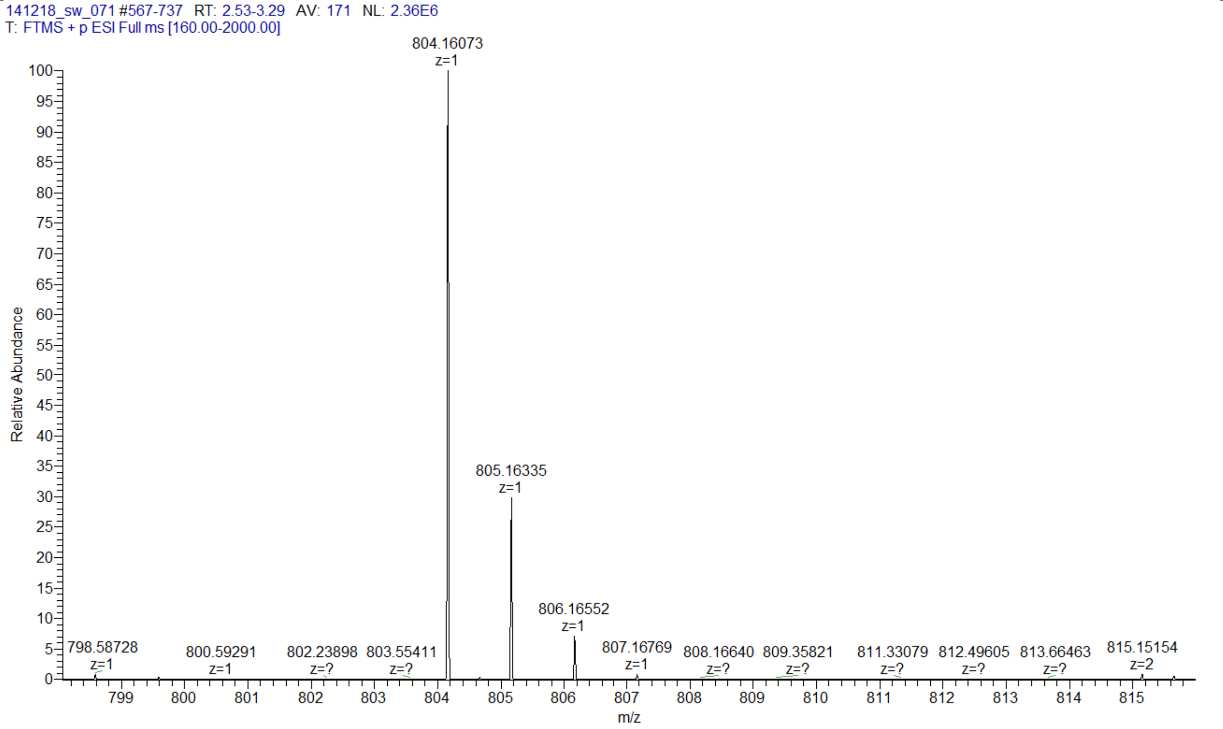

^{1}H NMR

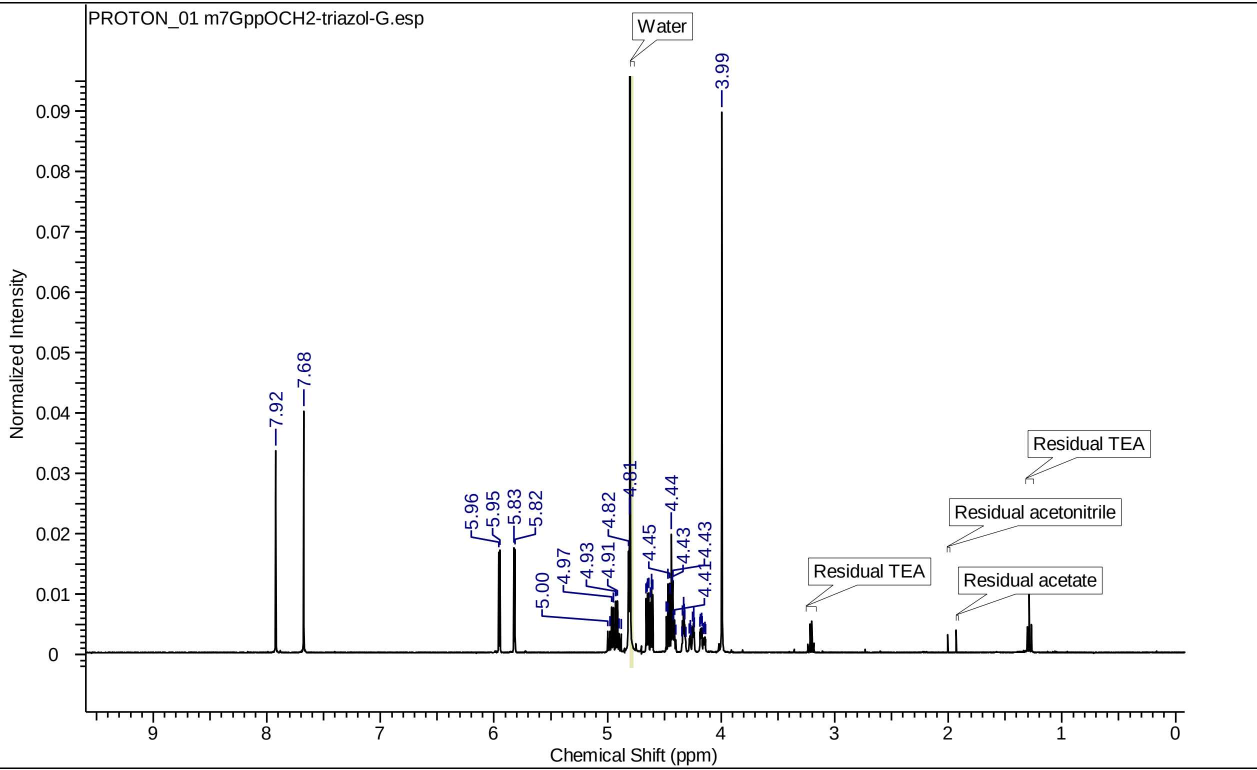

^{31}P NMR	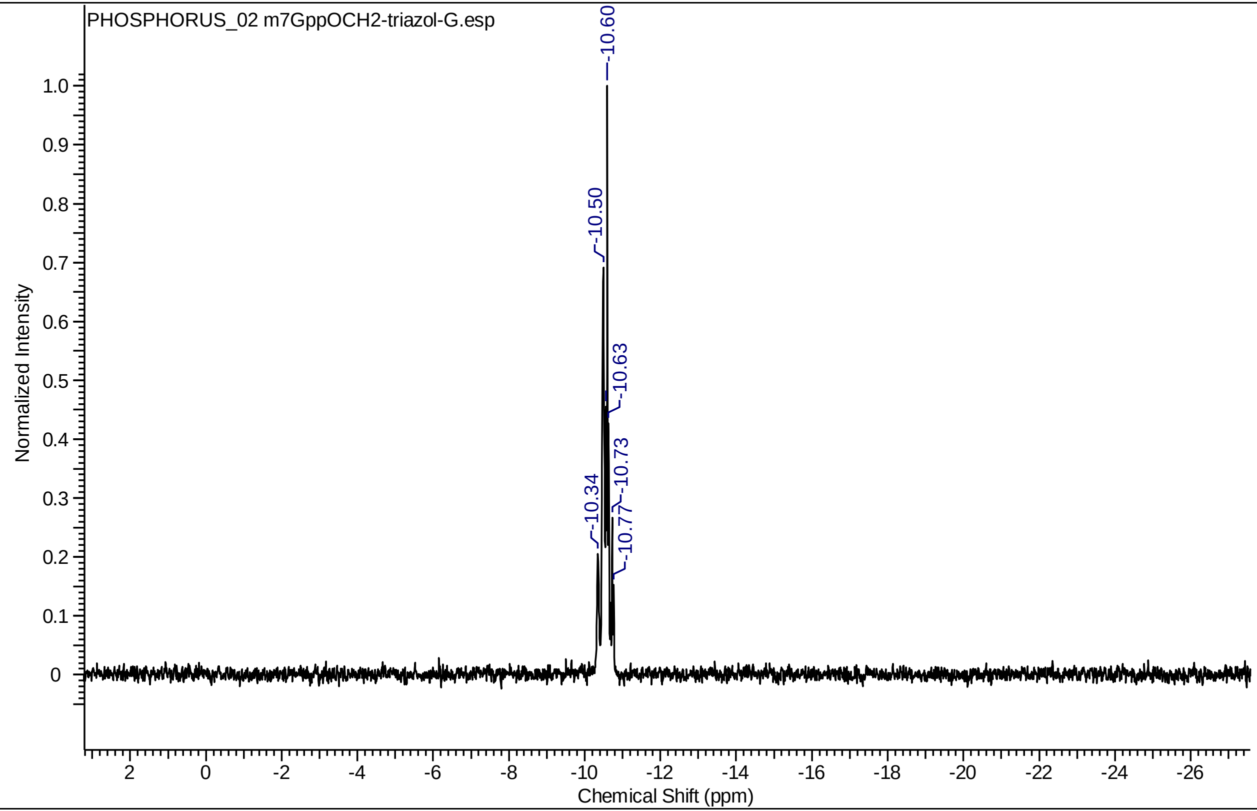

(4d) m^{7}GpppOCH$_2$-triazole-G

Structure	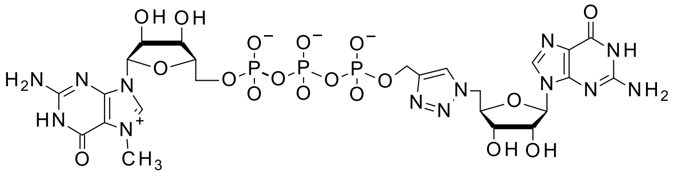
Reaction HPLC profile	Program C; before and after the reaction 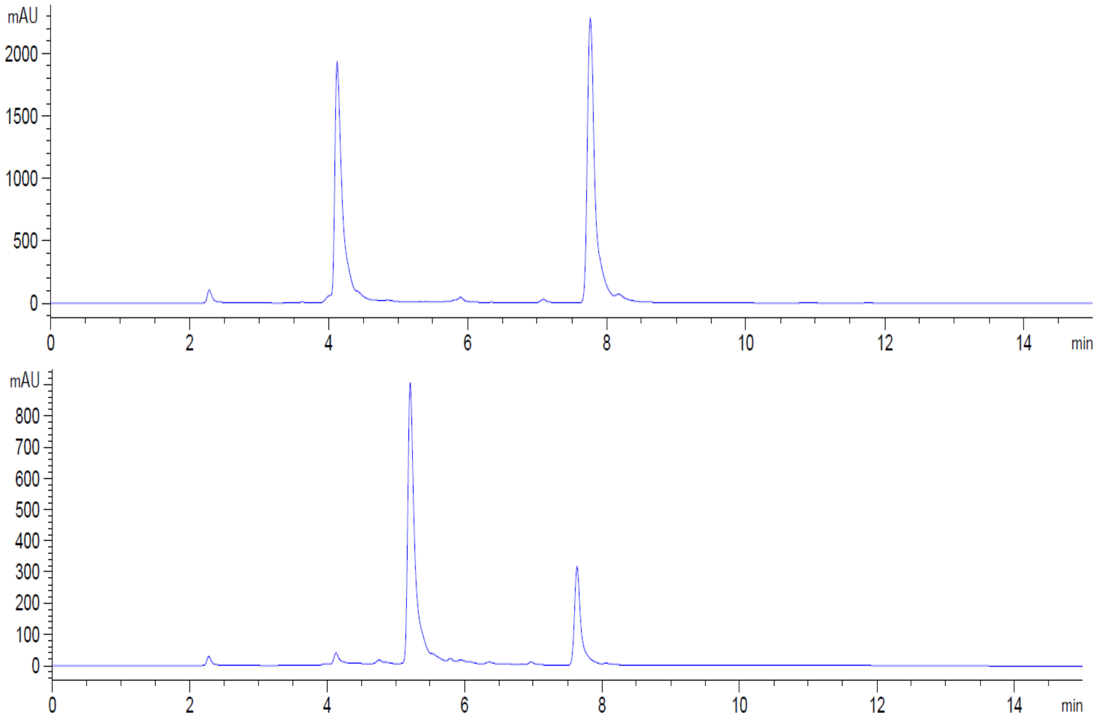

**Purified compound HPLC profile**	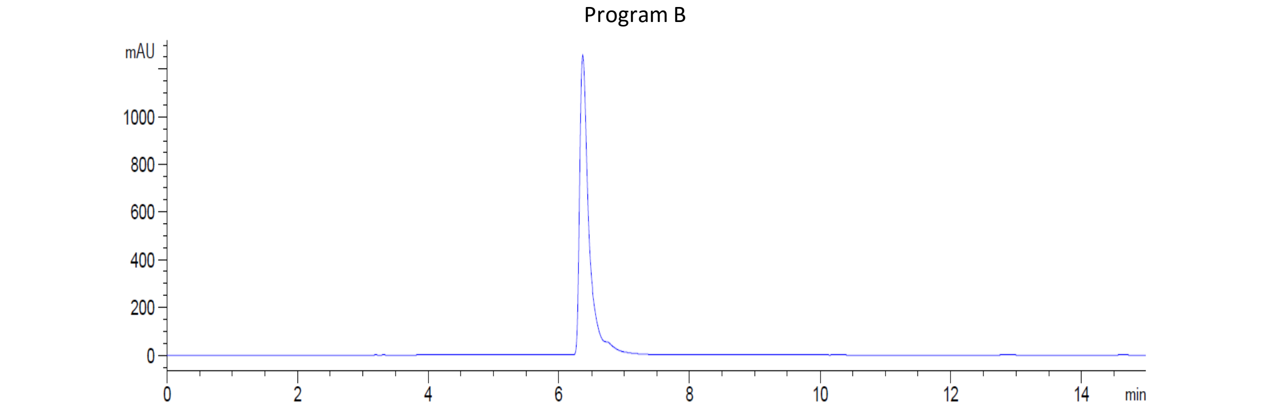
**HRMS**	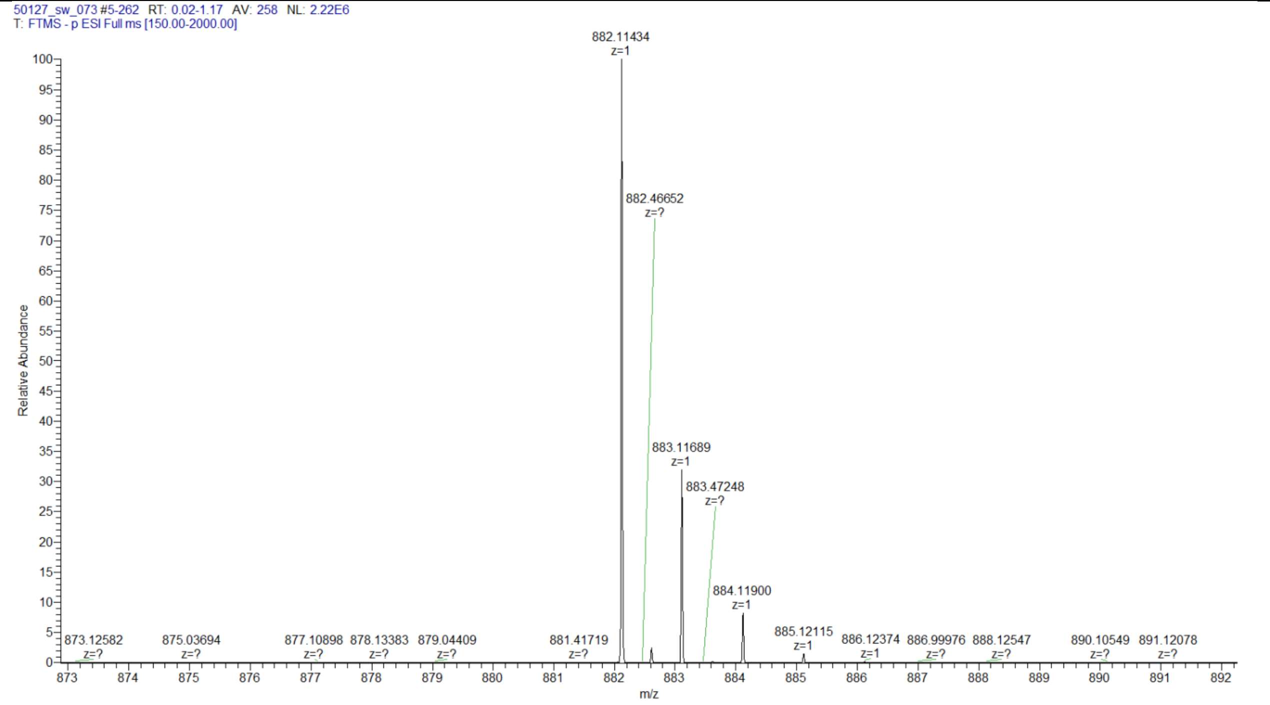
**^{1}H NMR**	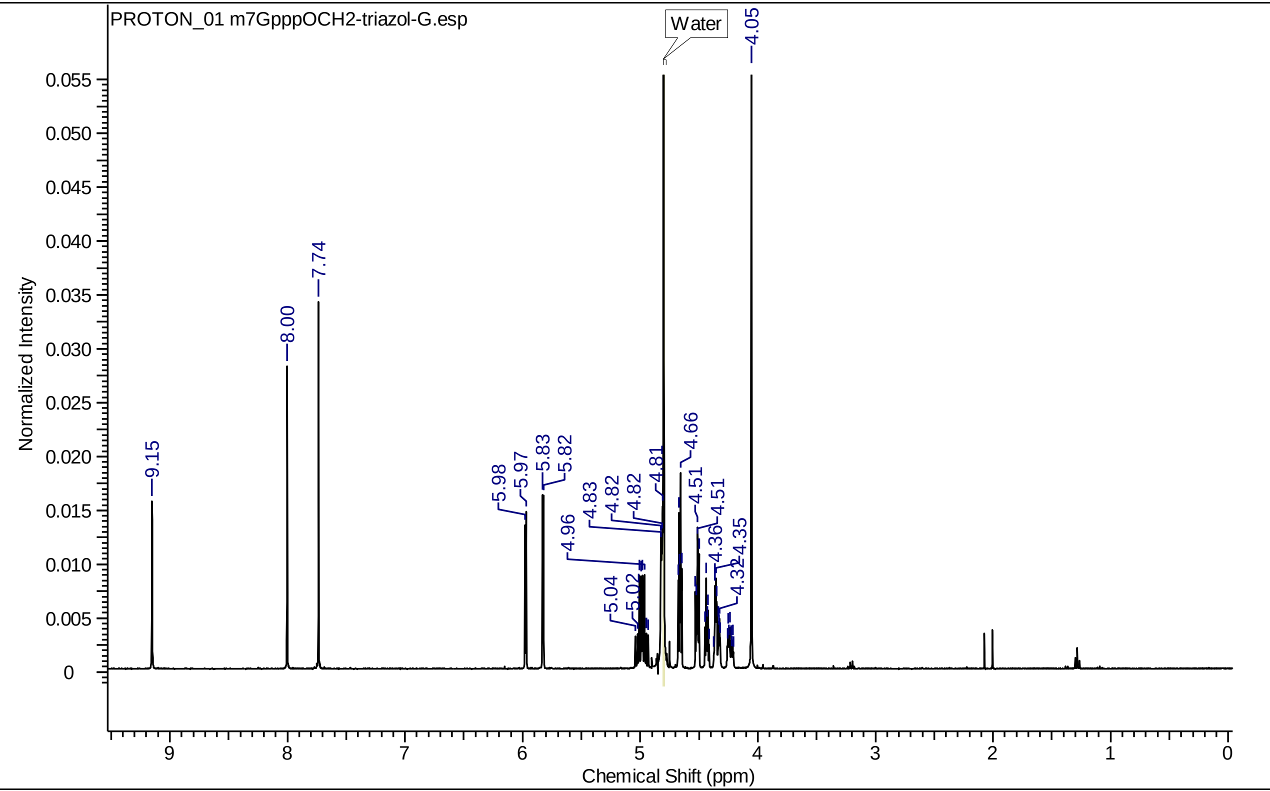

^{31}P NMR	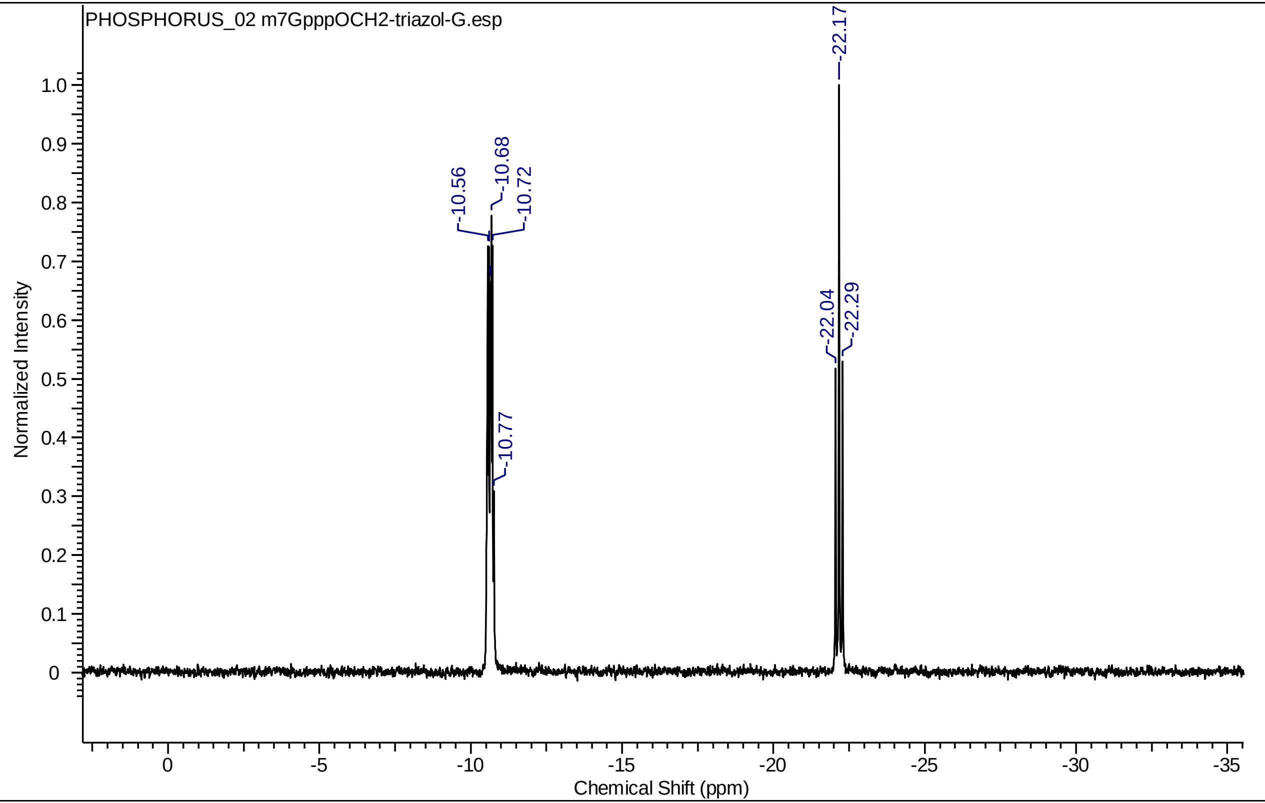

(5a) m^{7}G-triazole-CH$_2$SppG

Structure	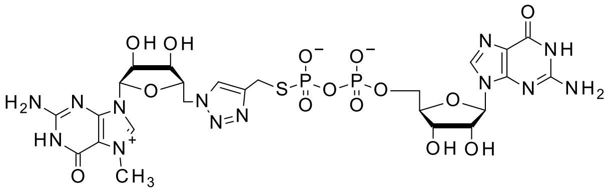
Reaction HPLC profile	Program B; before and after the reaction 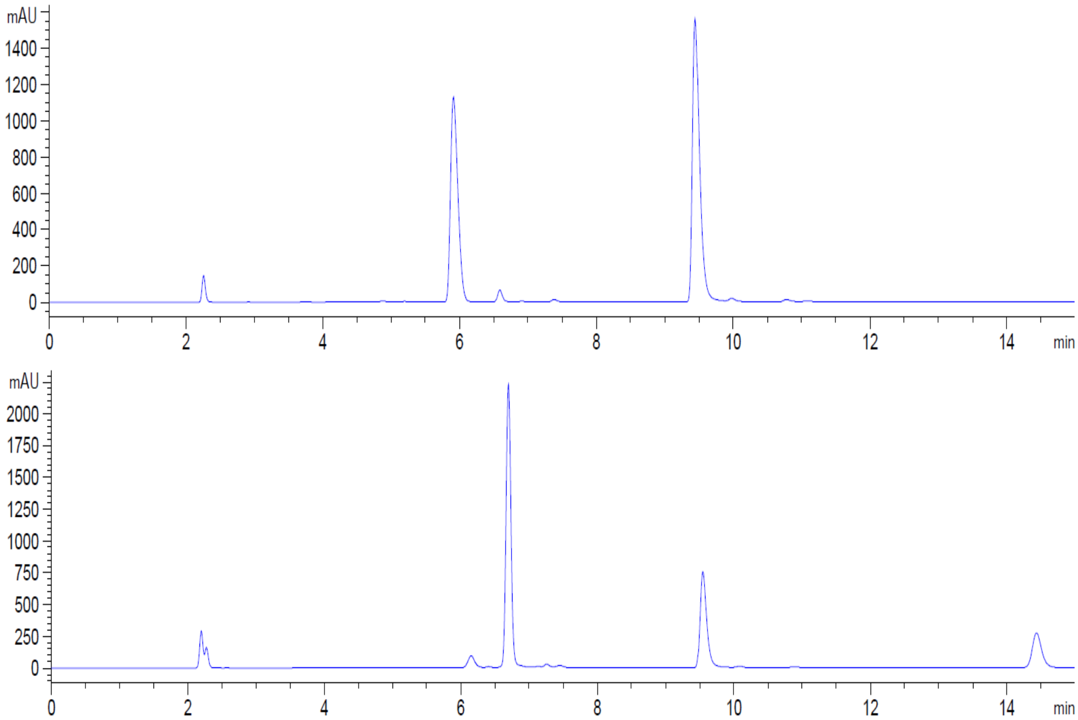

Purified compound HPLC profile

Program B

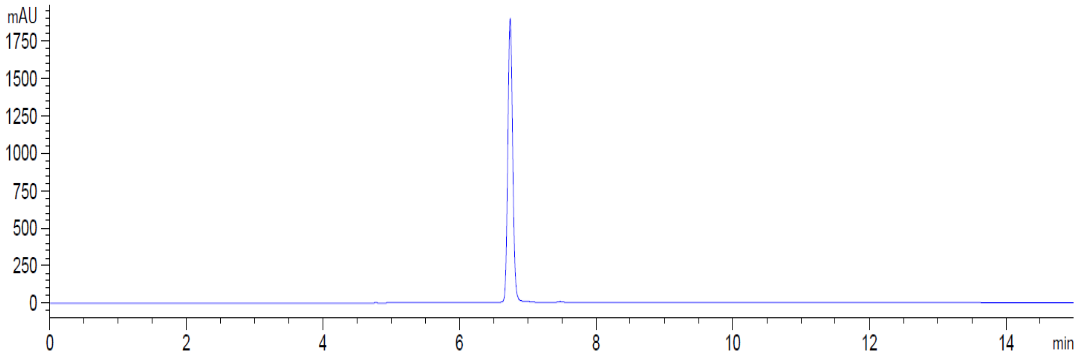

HRMS

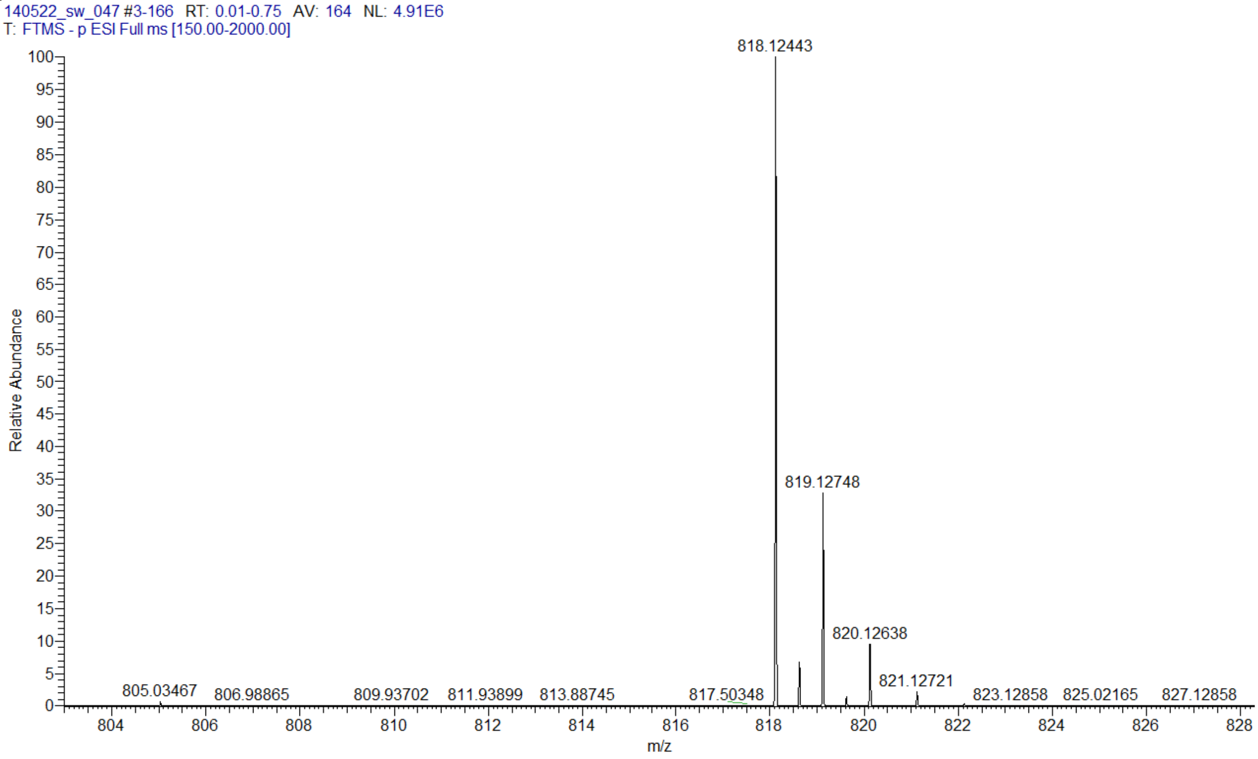

^{1}H NMR

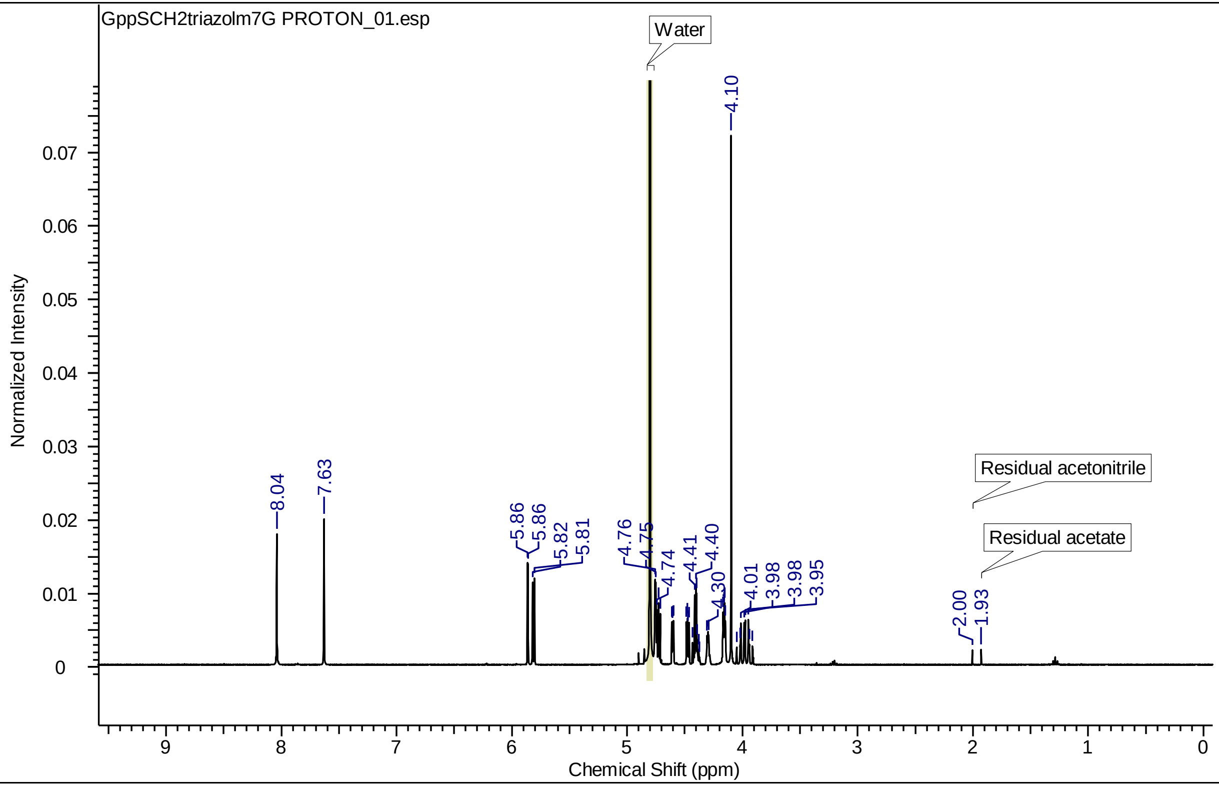

^{31}P NMR

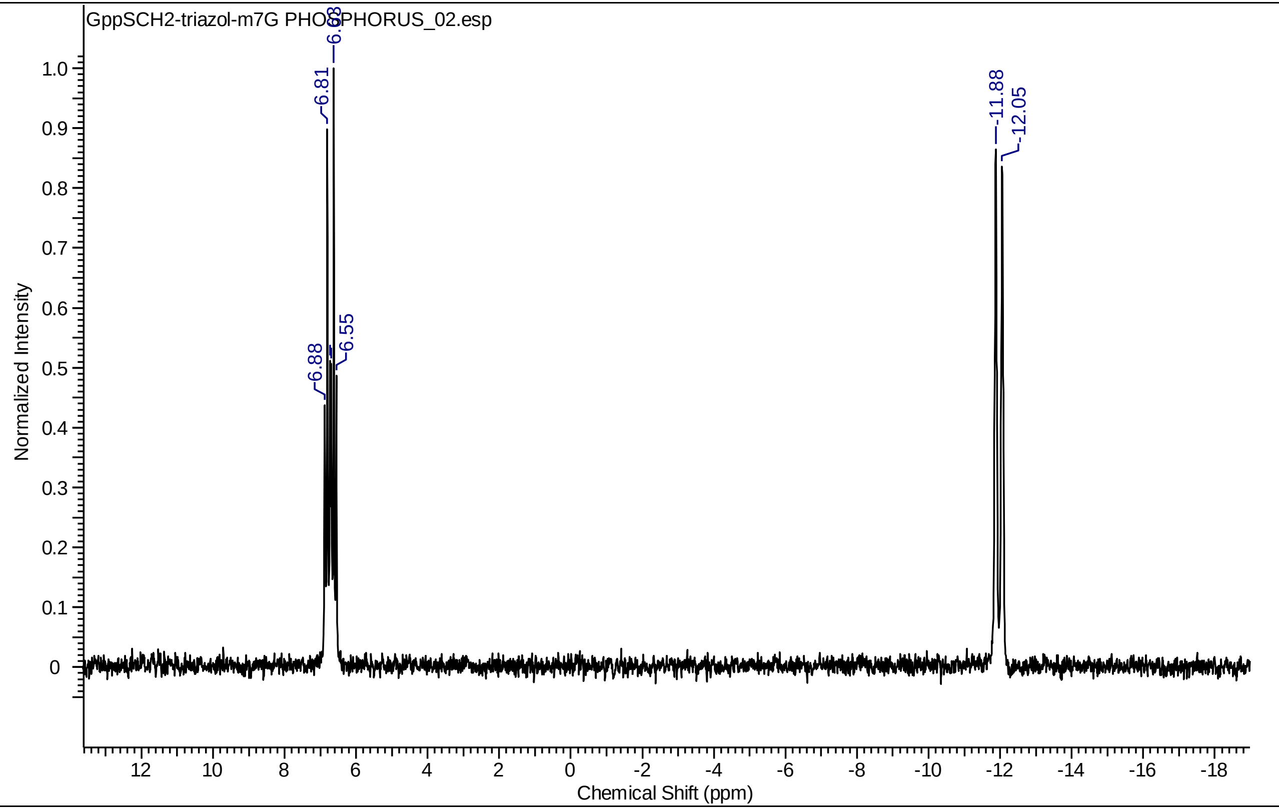

(5b) m^{7}Gp-triazole-CH$_2$SpppG

Structure

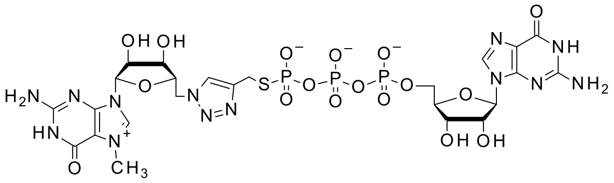

**Reaction HPLC profile**	Program B; before and after the reaction 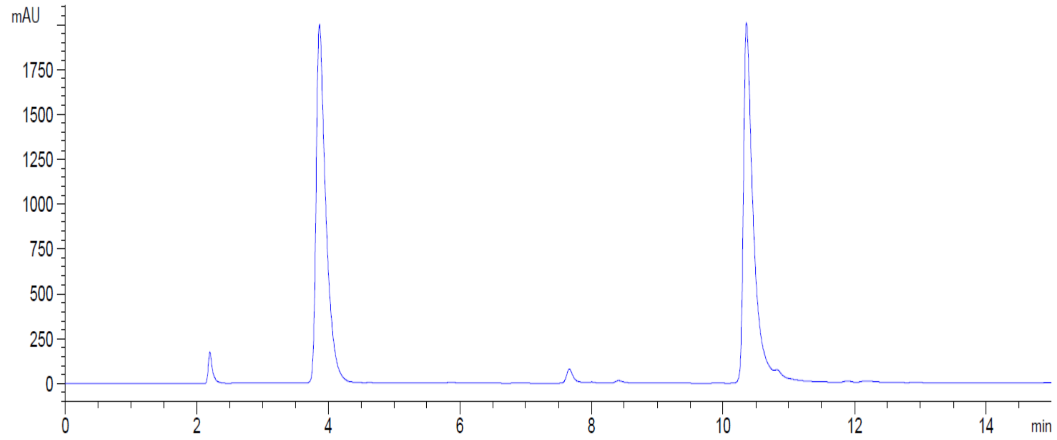 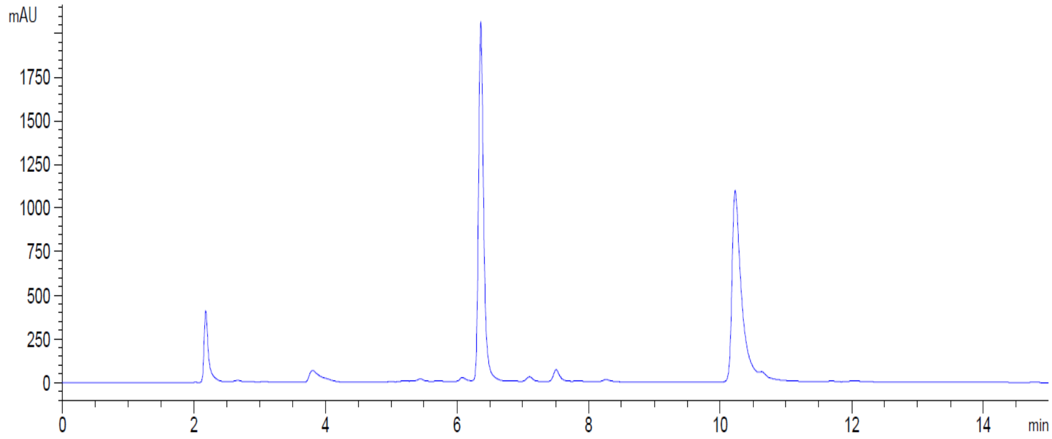
**Purified compound HPLC profile**	Program B 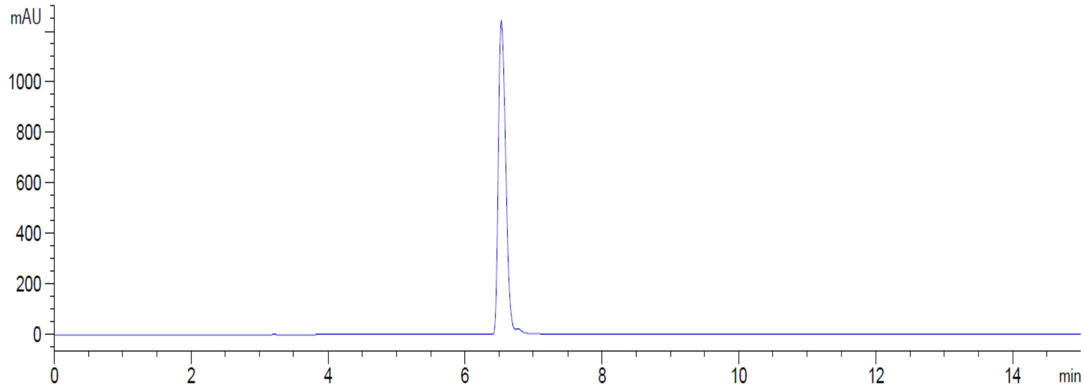

HRMS

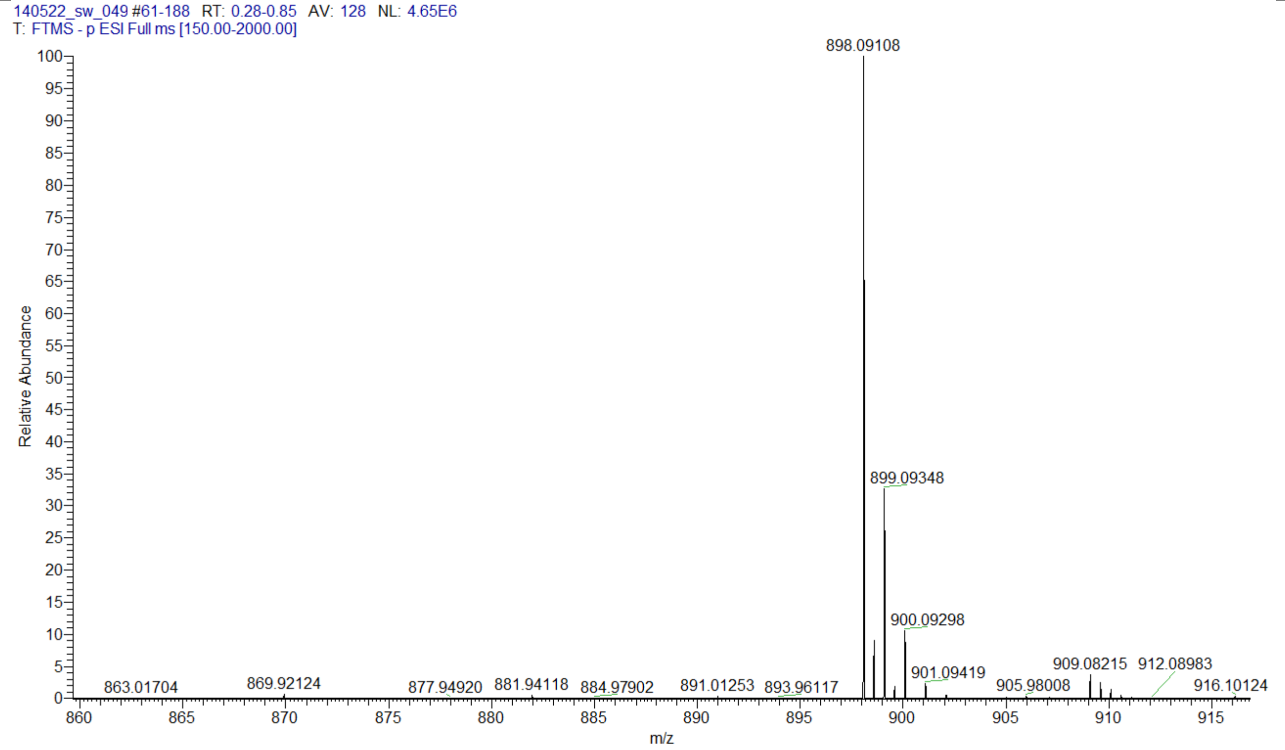

140522_sw_049 #61-188 RT: 0.28-0.85 AV: 128 NL: 4.65E6
T: FTMS - p ESI Full ms [150.00-2000.00]
Relative Abundance
m/z
898.09108
899.09348
900.09298
901.09419
905.98008
909.08215
912.08983
916.10124
863.01704
869.92124
877.94920
881.94118
884.97902
891.01253
893.96117

^{1}H NMR

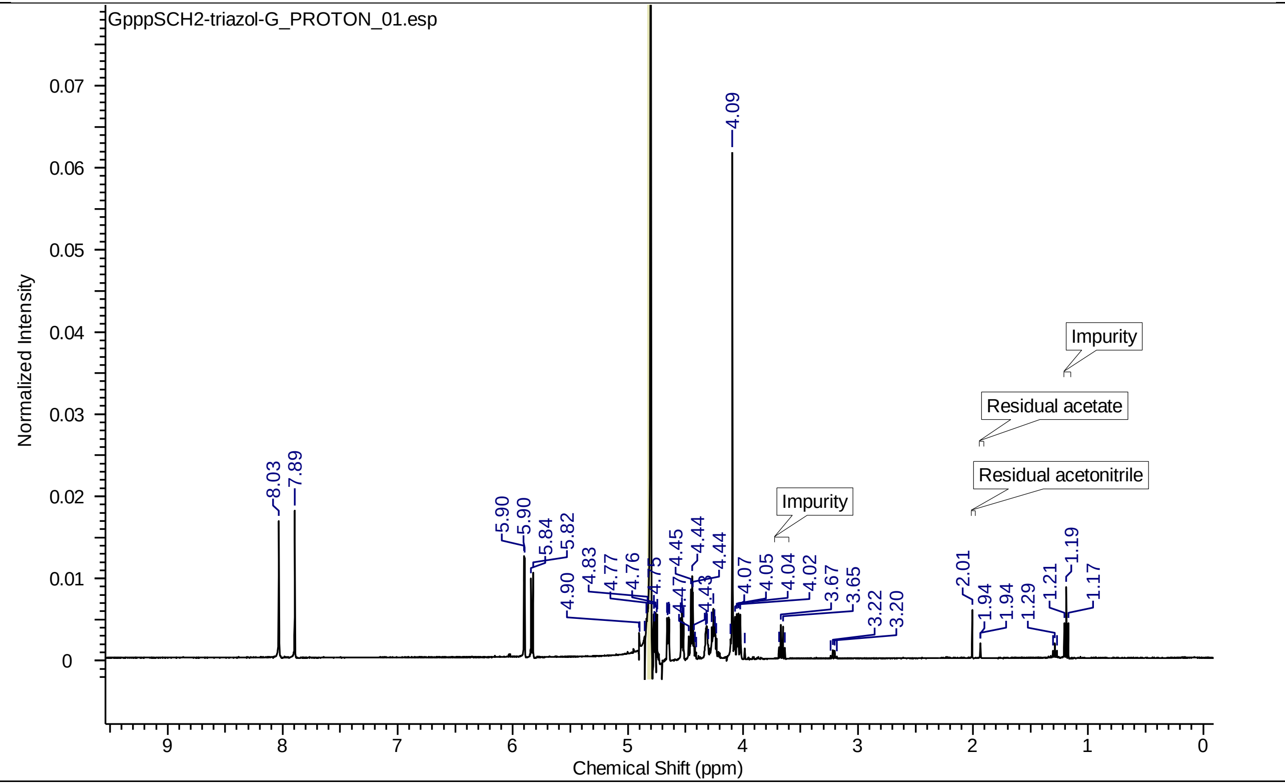

GpppSCH2-triazol-G_PROTON_01.esp
Normalized Intensity
Chemical Shift (ppm)
Impurity
Residual acetate
Residual acetonitrile
Impurity
8.03
7.89
5.90
5.90
5.84
5.82
4.90
4.83
4.77
4.76
4.75
4.47
4.45
4.44
4.43
4.44
4.09
4.07
4.05
4.04
4.02
3.67
3.65
3.22
3.20
2.01
1.94
1.94
1.29
1.21
1.19
1.17

^{31}P NMR

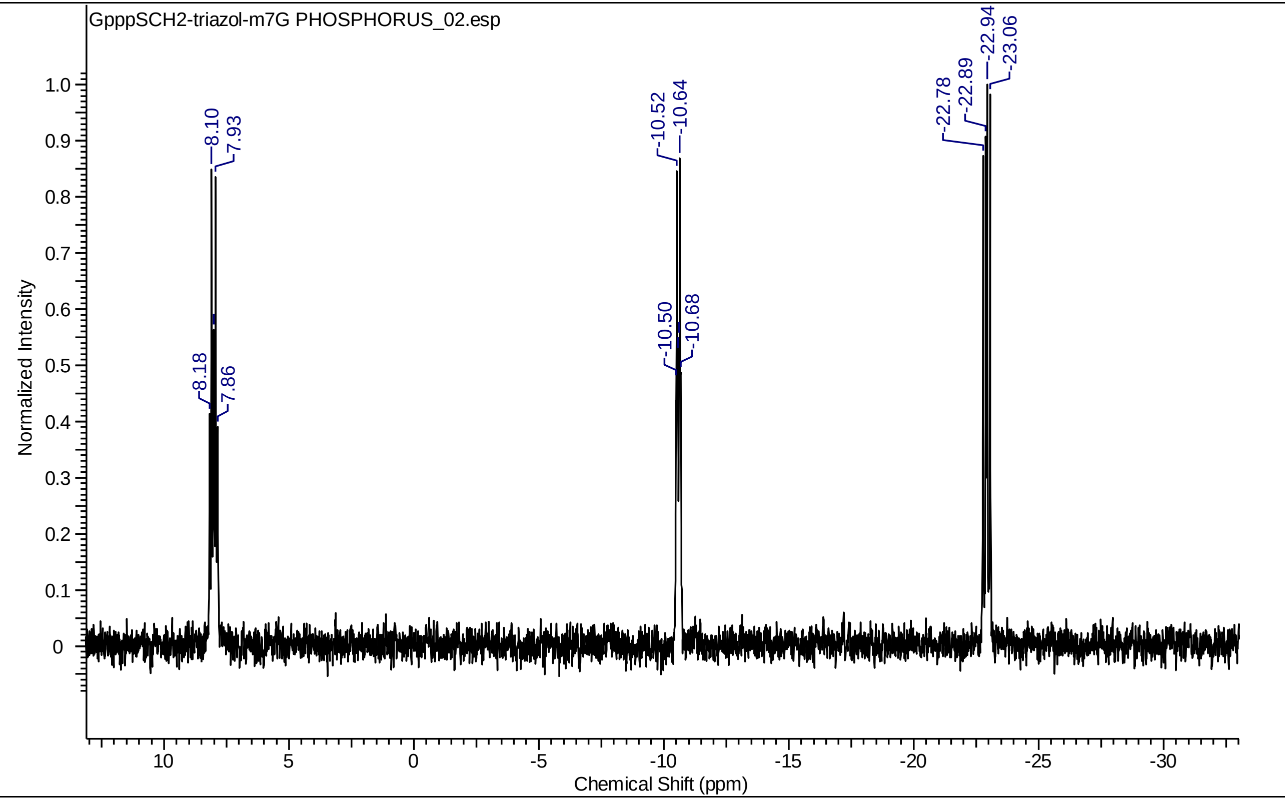

(5c) m^{7}GppSCH$_2$-triazole-G

Structure

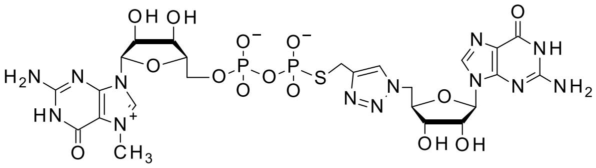

Reaction HPLC profile

Program B; before and after the reaction

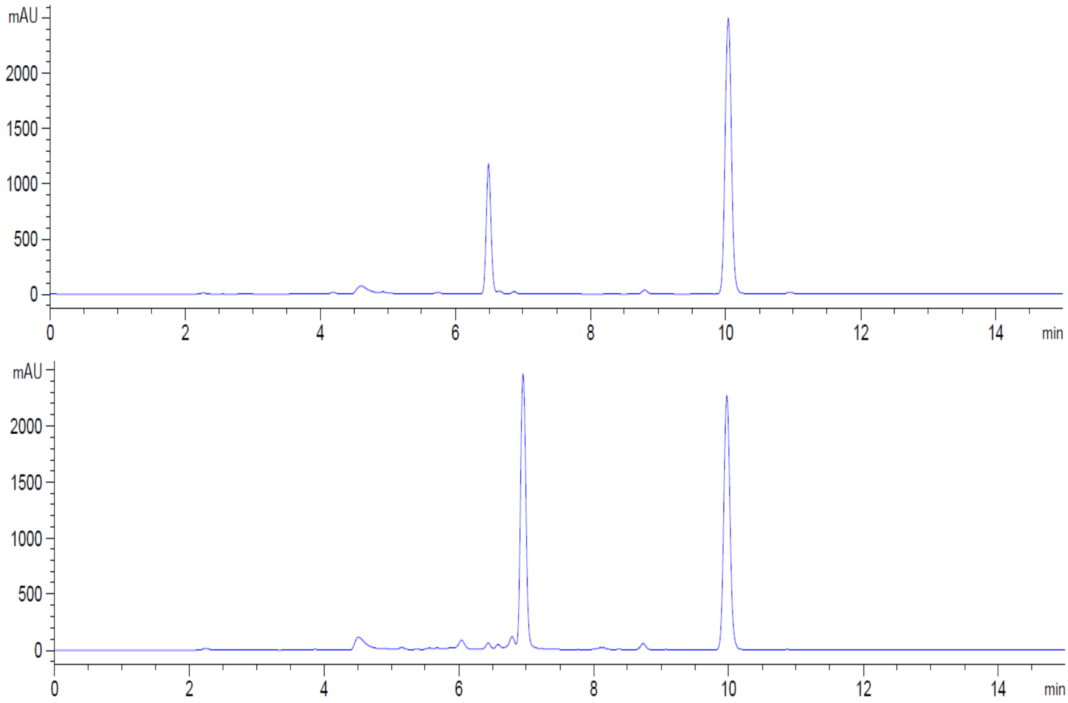

Purified compound HPLC profile

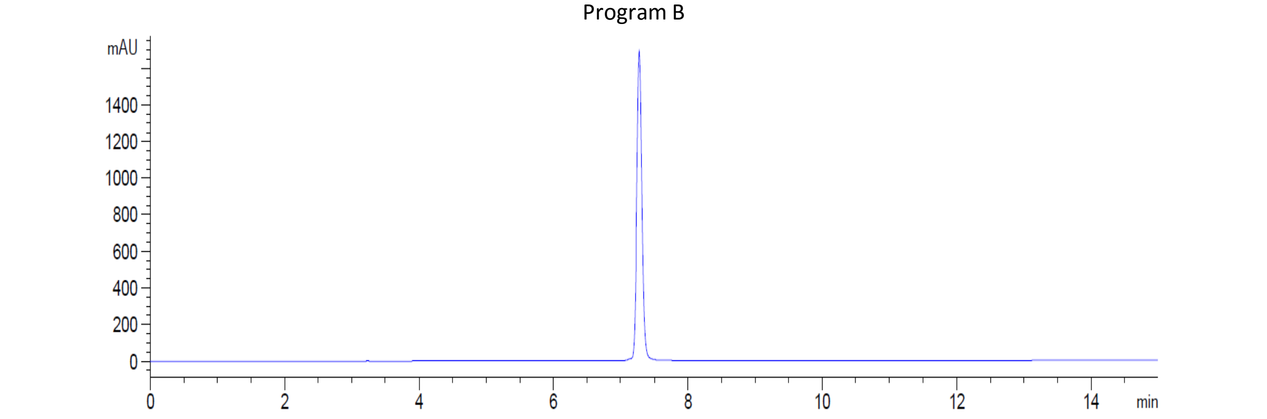

HRMS

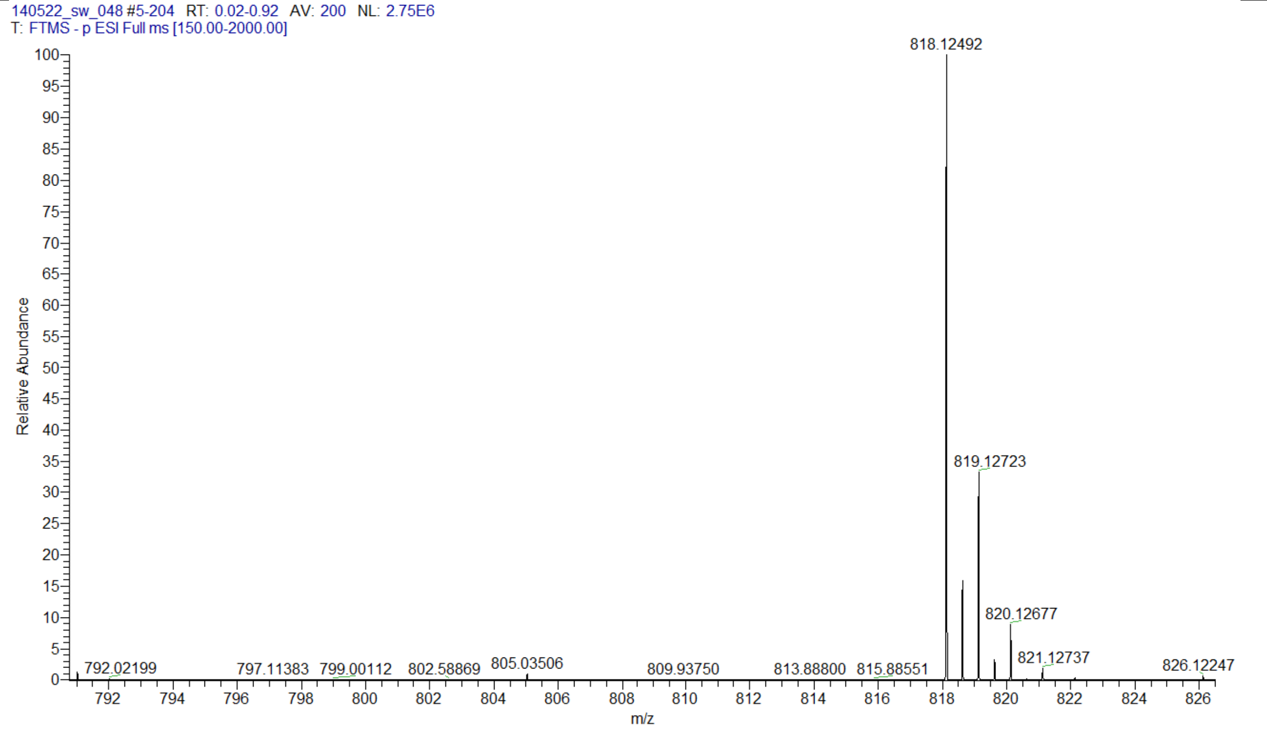

^{1}H NMR

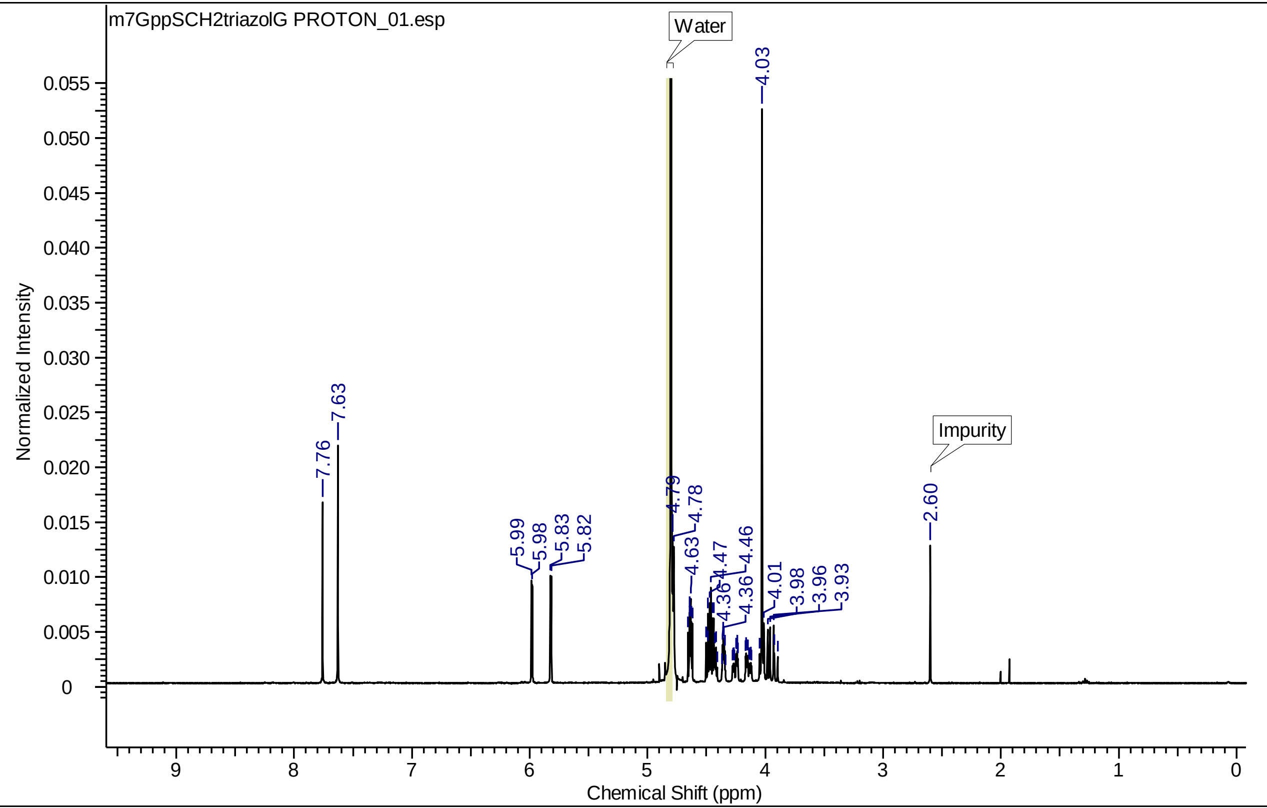

^{31}P NMR

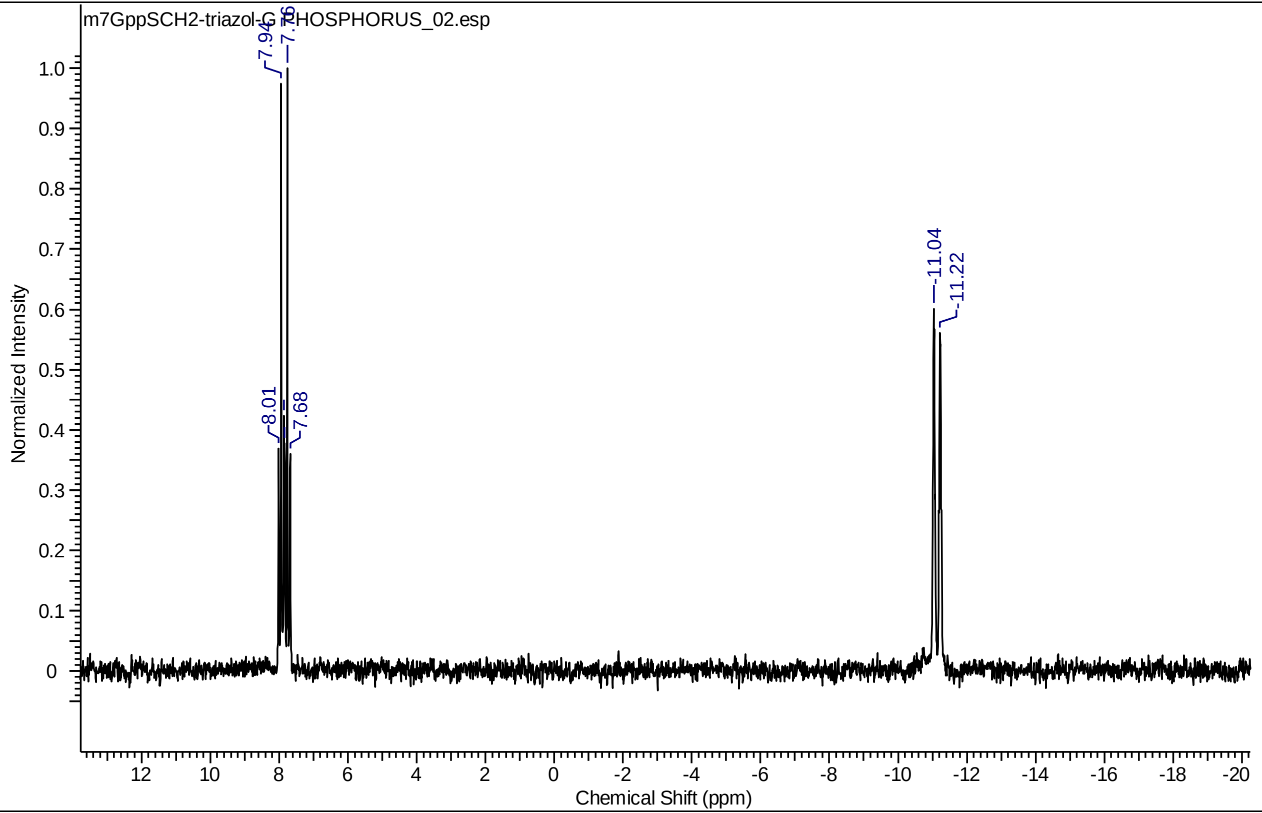

(5d) m^{7}GpppSCH$_2$-triazole-G

Structure

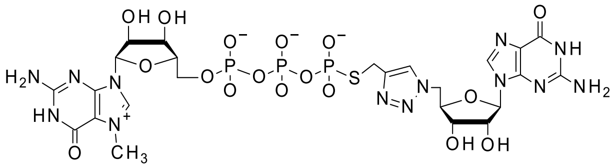

Reaction HPLC profile

Program B; before and after the reaction

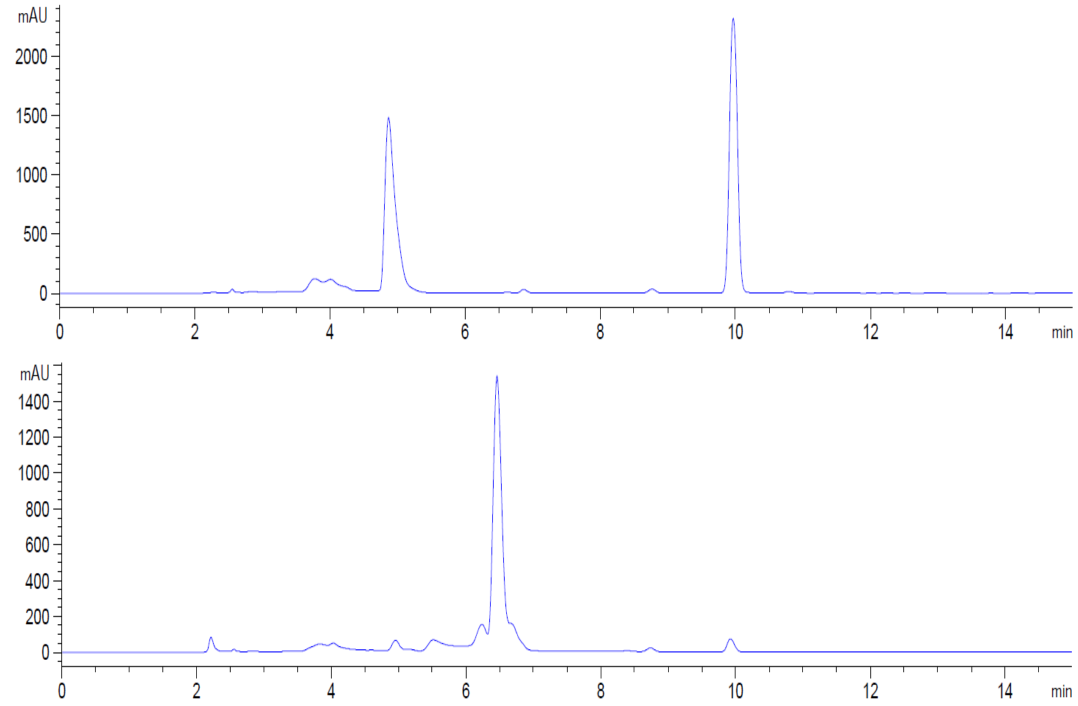

**Purified compound HPLC profile**	Program B 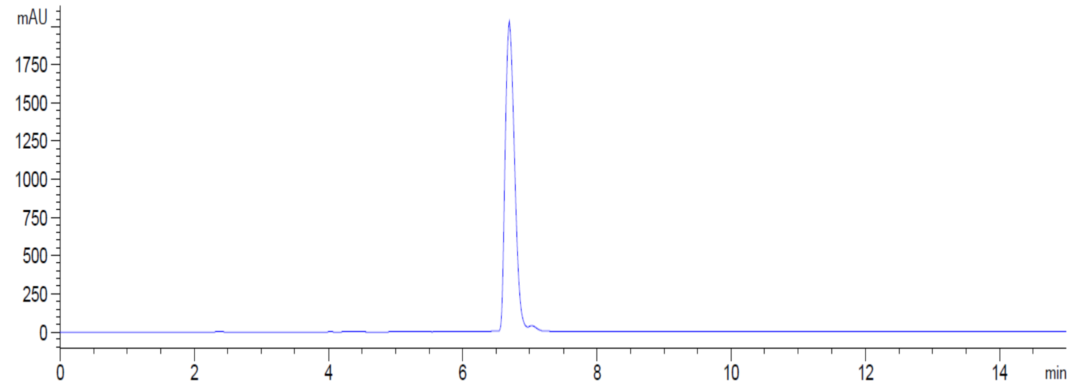
**HRMS**	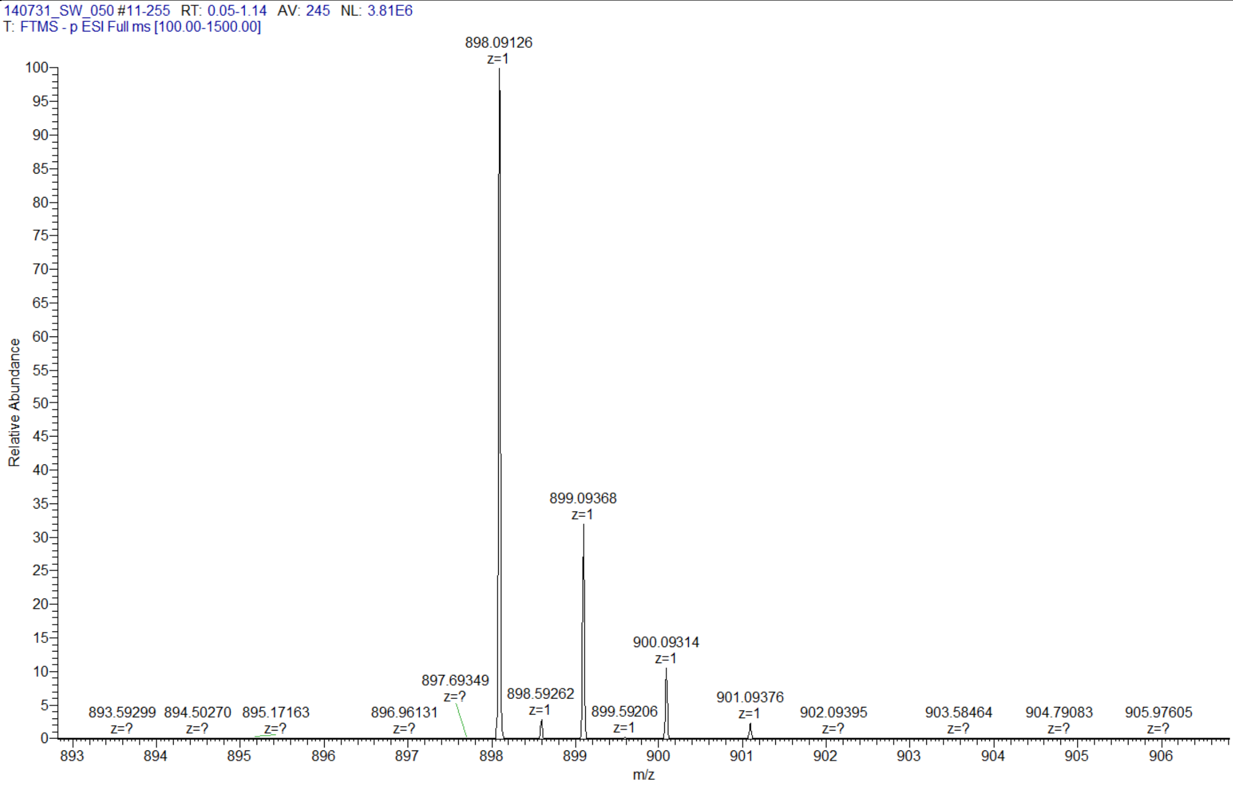

^{1}H NMR	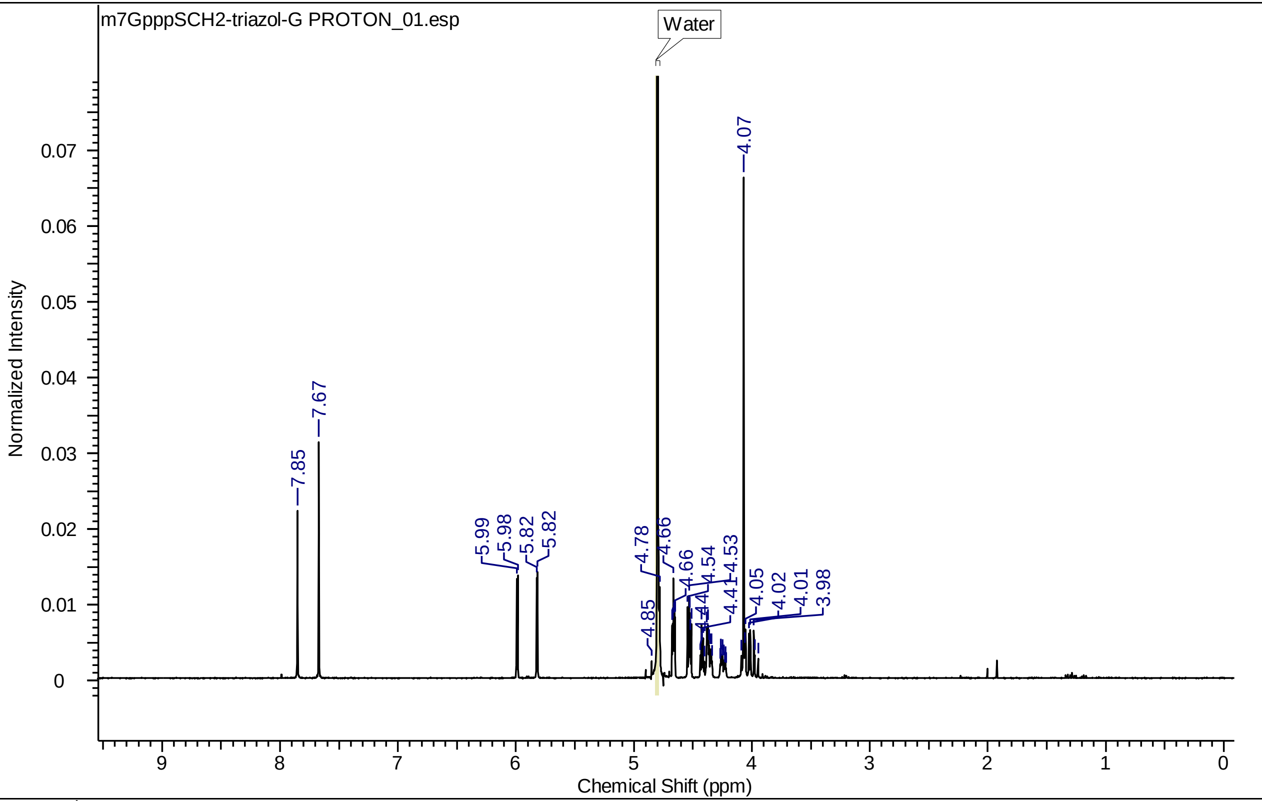
^{31}P NMR	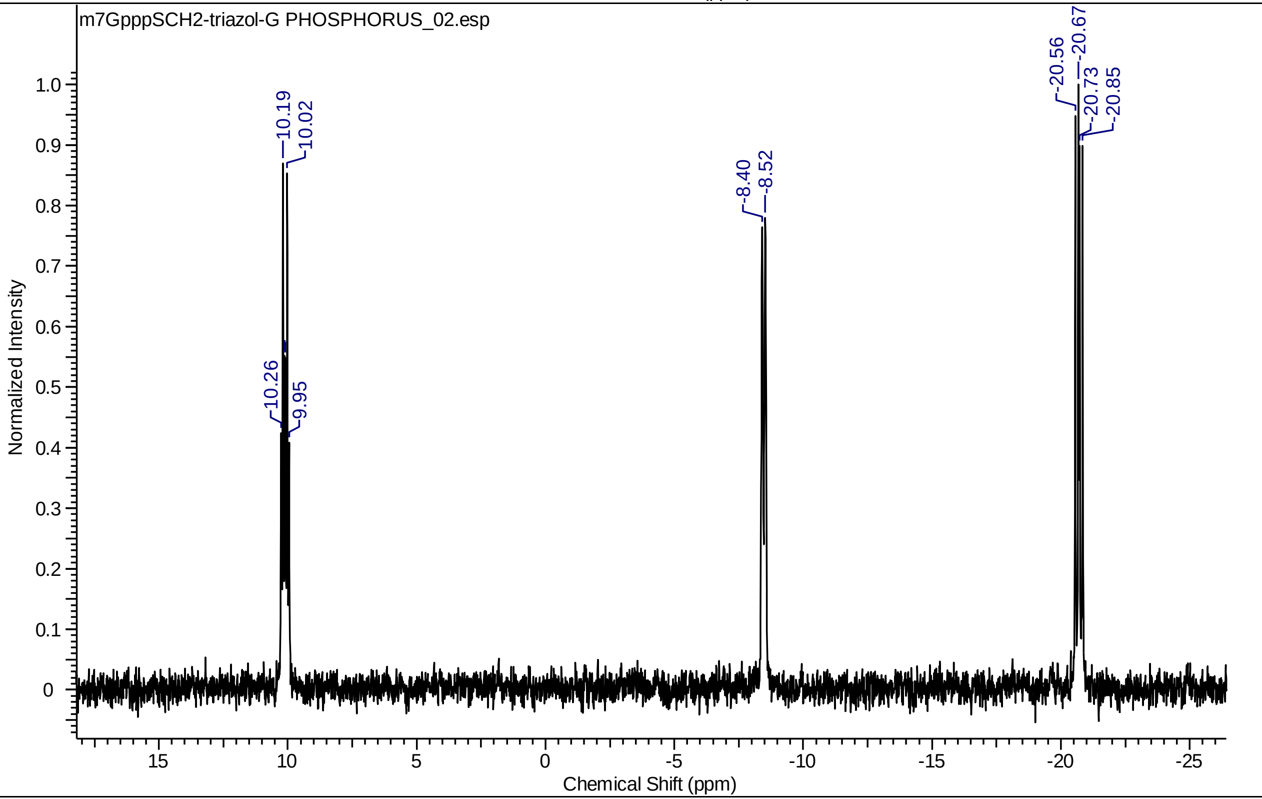

(6a) m^{7}G-triazole-CH$_2$NHppG

Structure	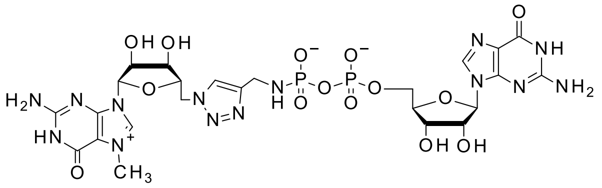

**Reaction HPLC profile**	Program B 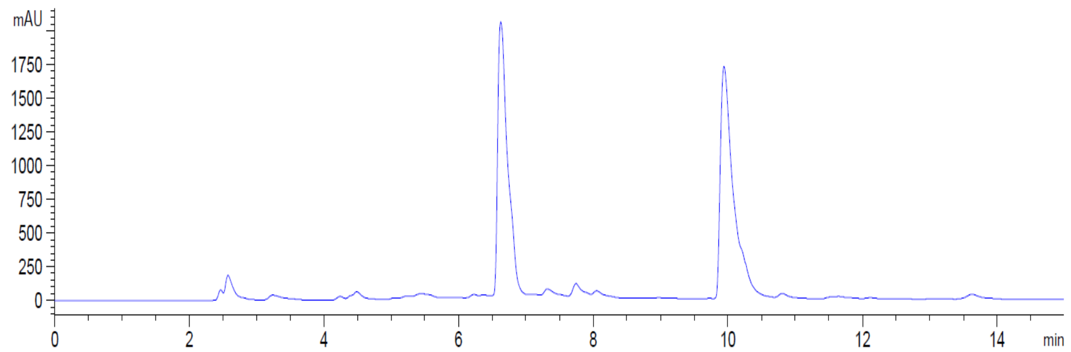
**Purified compound HPLC profile**	Program B 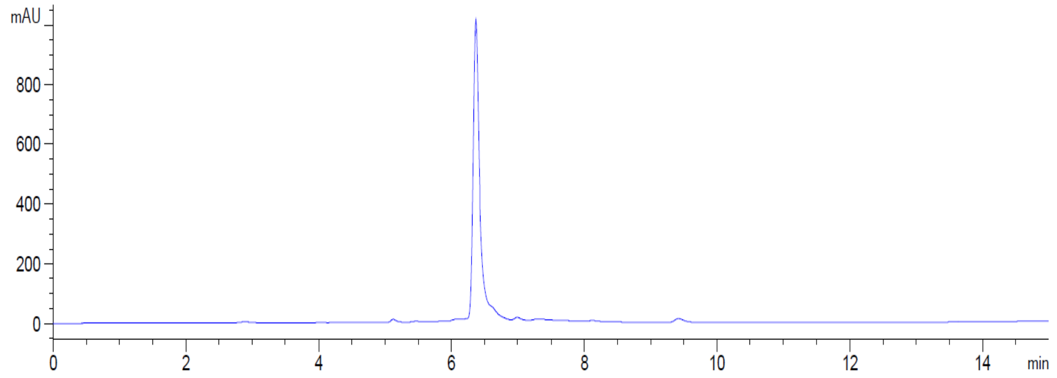
**HRMS**	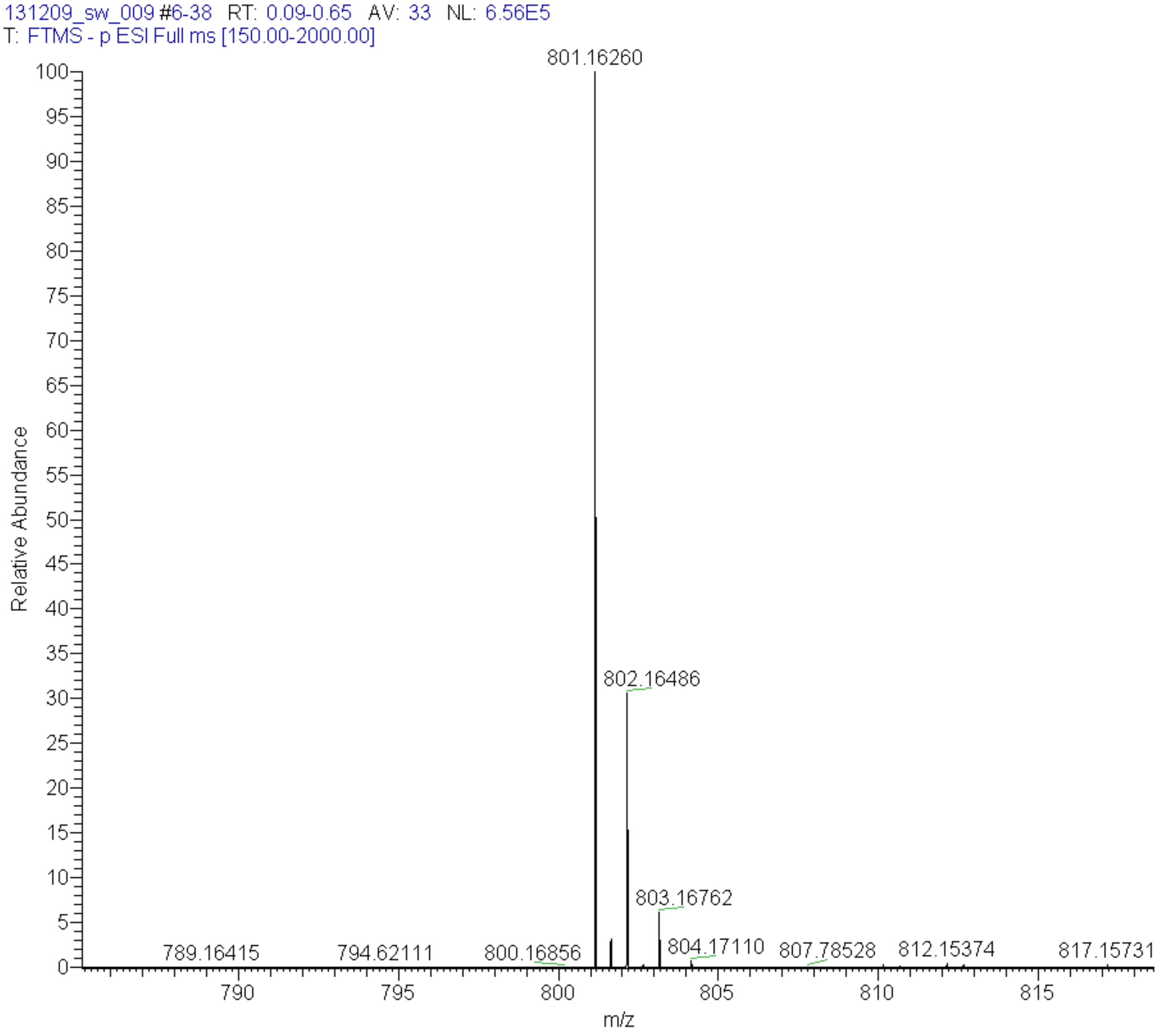

**^{1}H NMR**	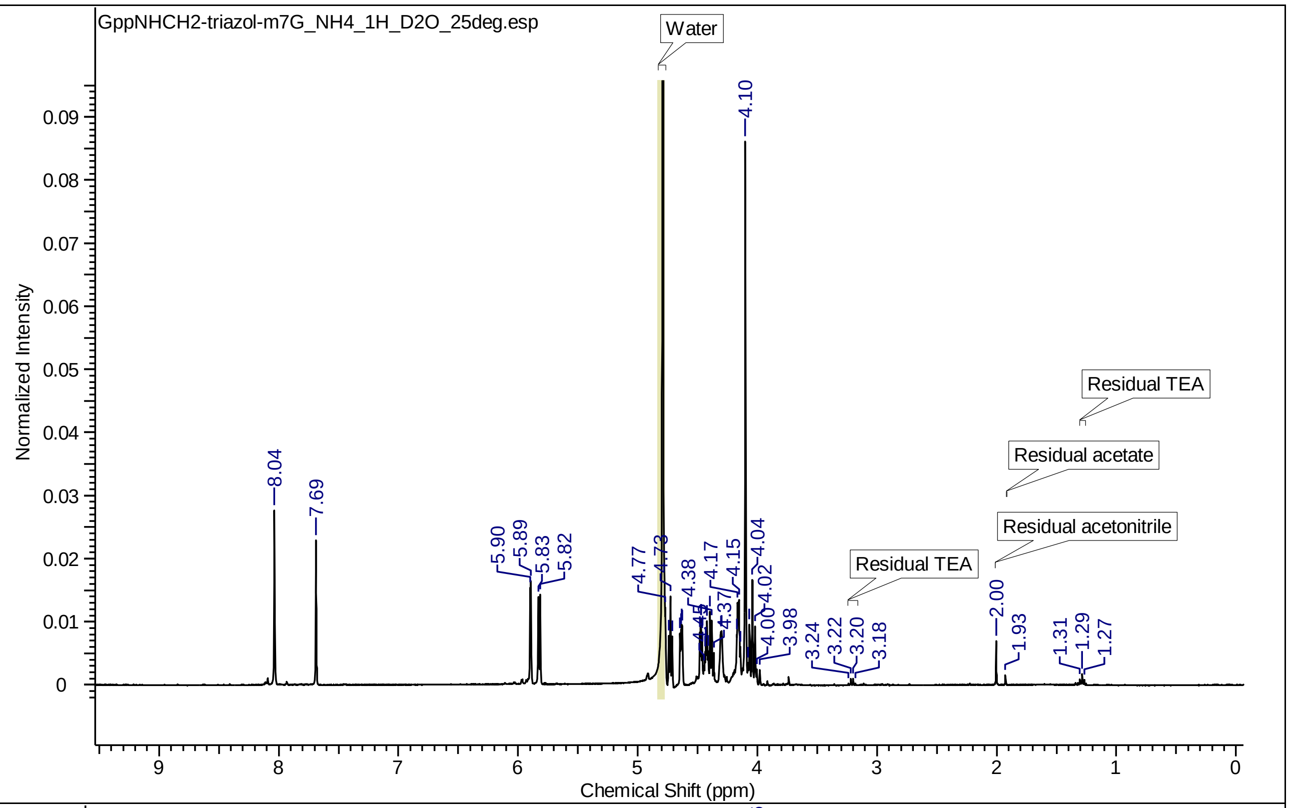
**^{31}P NMR**	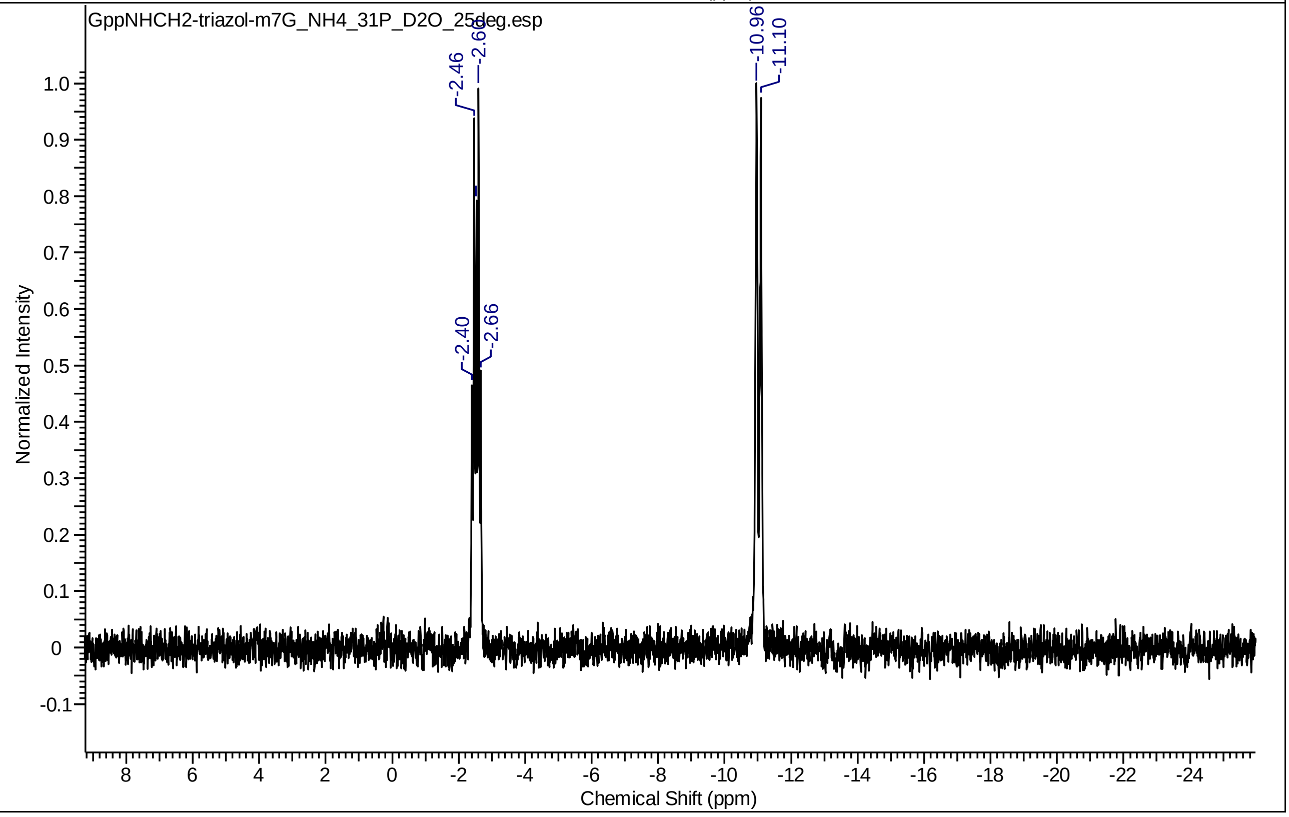

(6b) m^7G-triazole-CH_2NHpppG

**Structure**	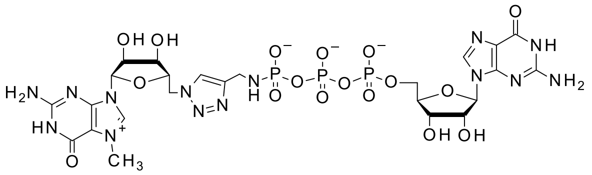

Reaction HPLC profile	Program C<br>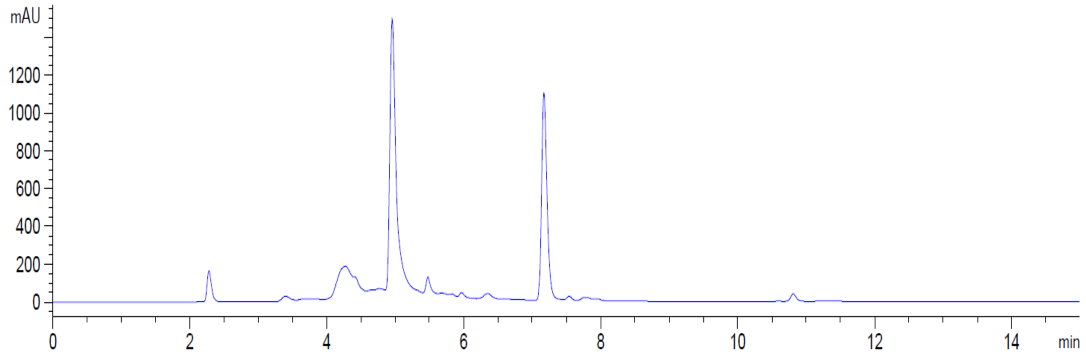
Purified compound HPLC profile	Program B<br>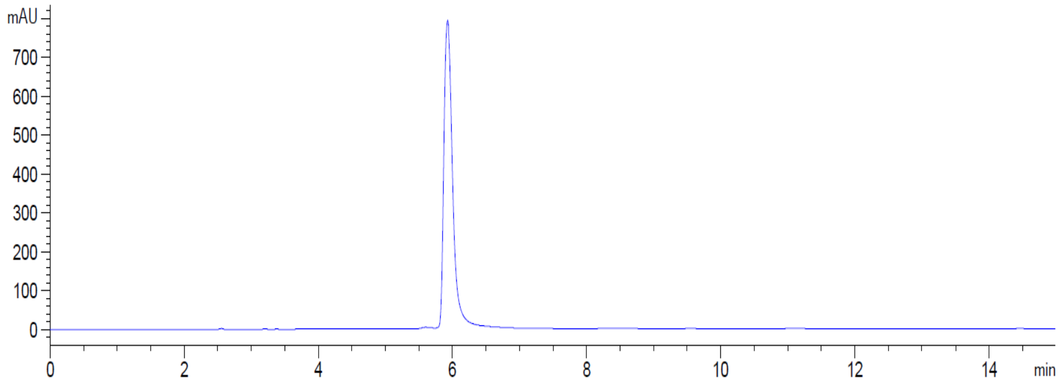
HRMS	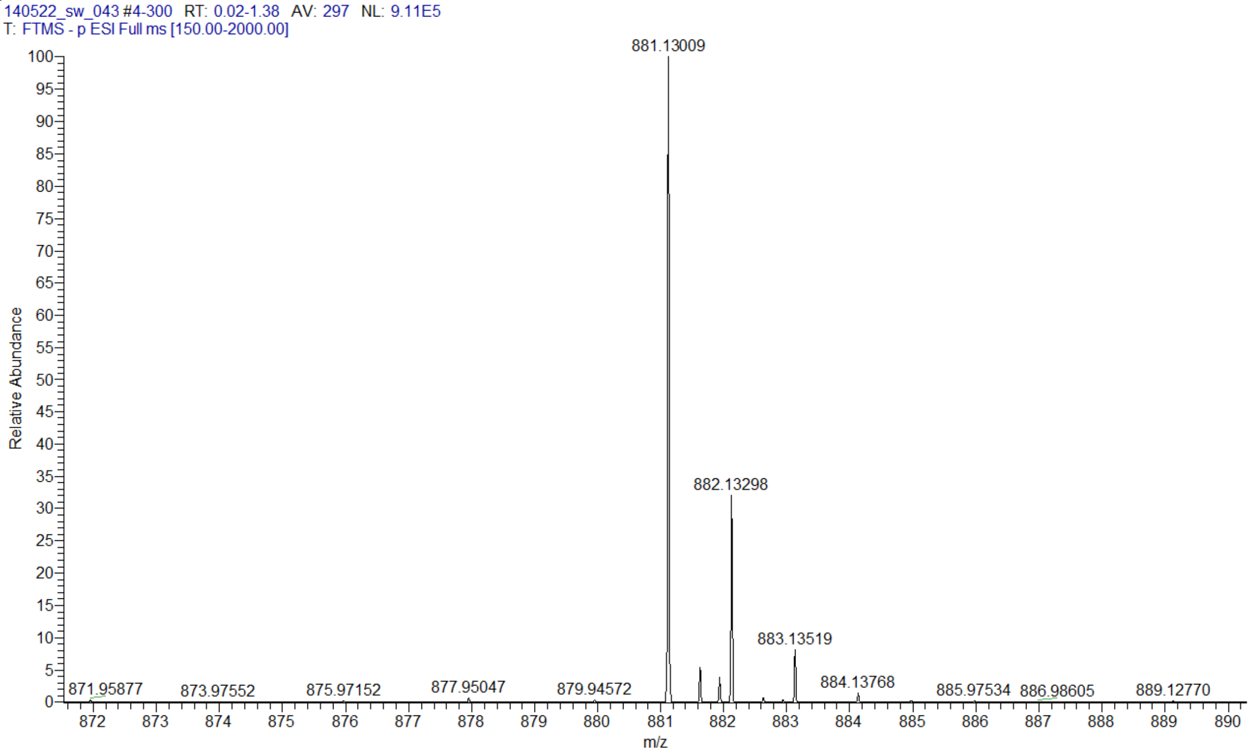

^{1}H NMR	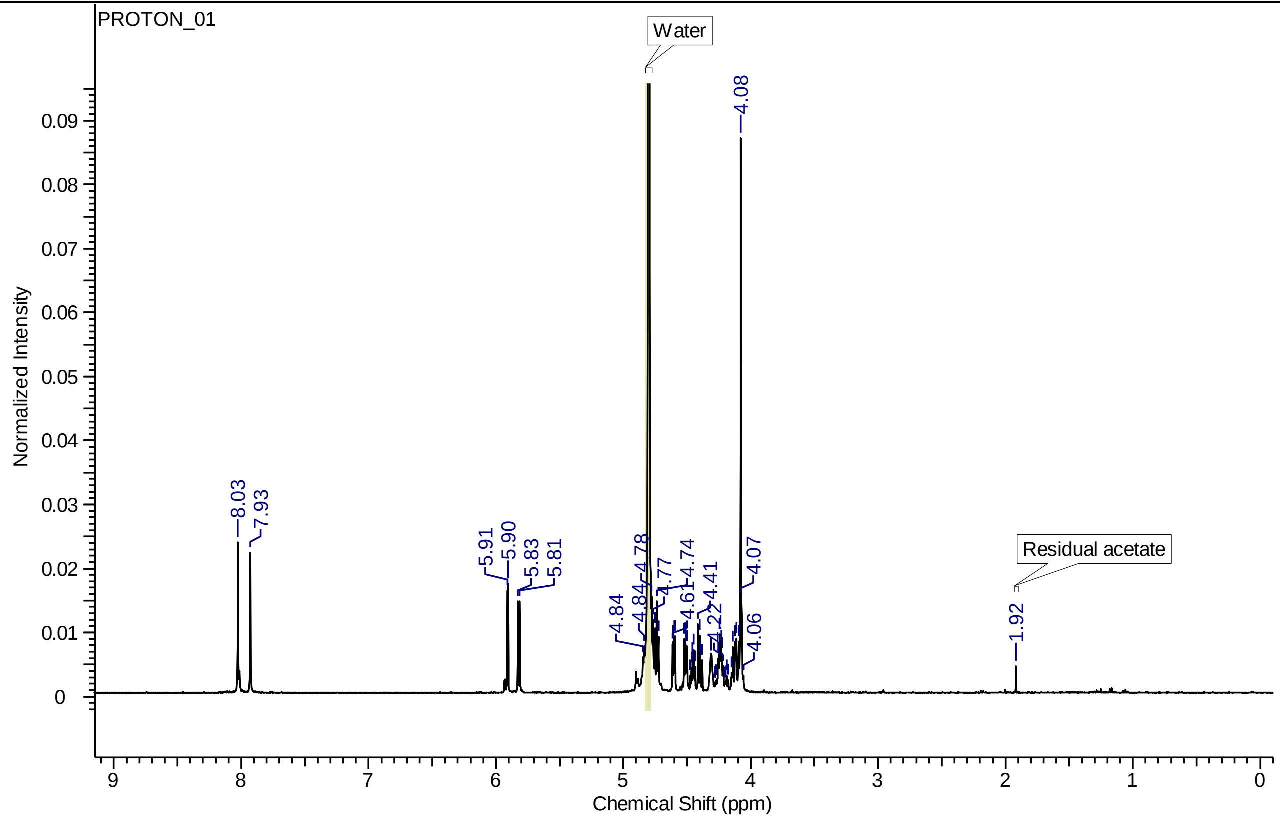
^{31}P NMR	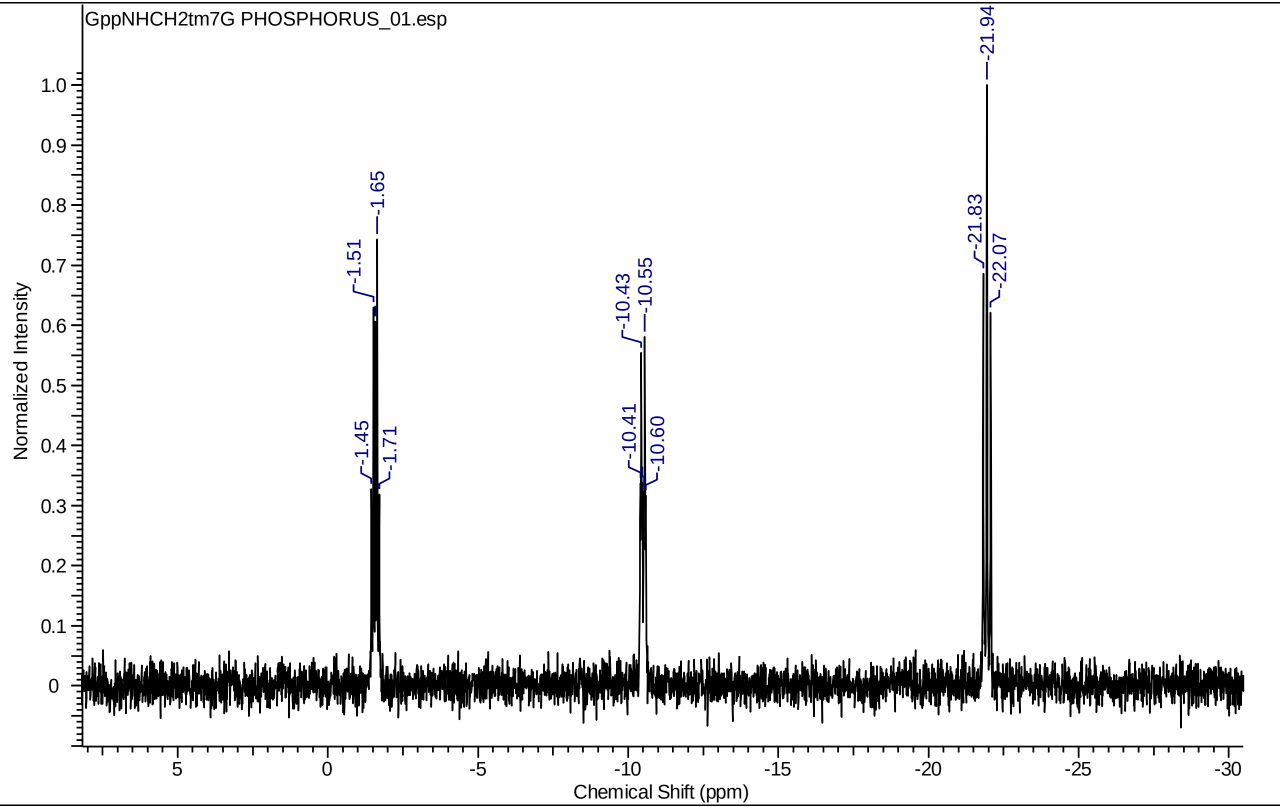

(6c) m^{7}GppNHCH$_2$-trazole-G

Structure	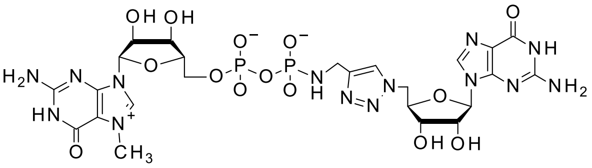

**Reaction HPLC profile**	Program B<br>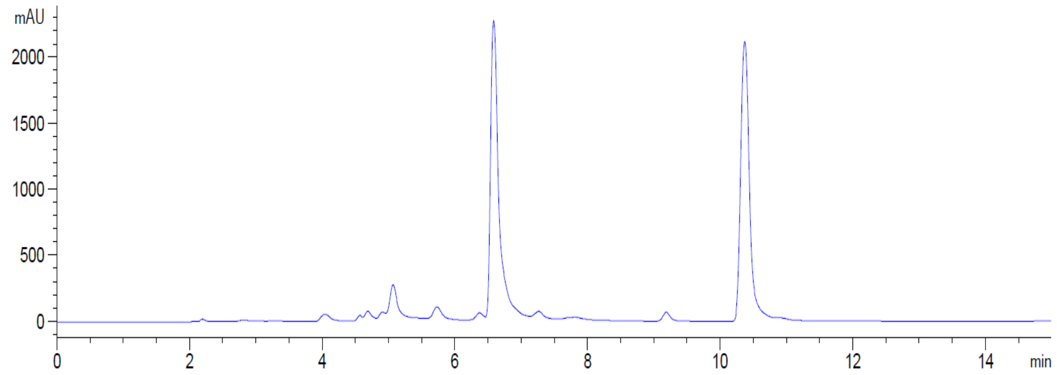
**Purified compound HPLC profile**	Program B<br>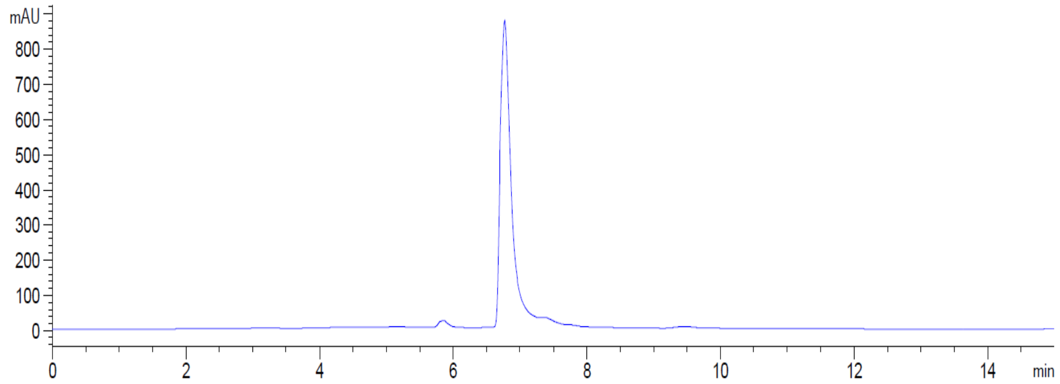
**HRMS**	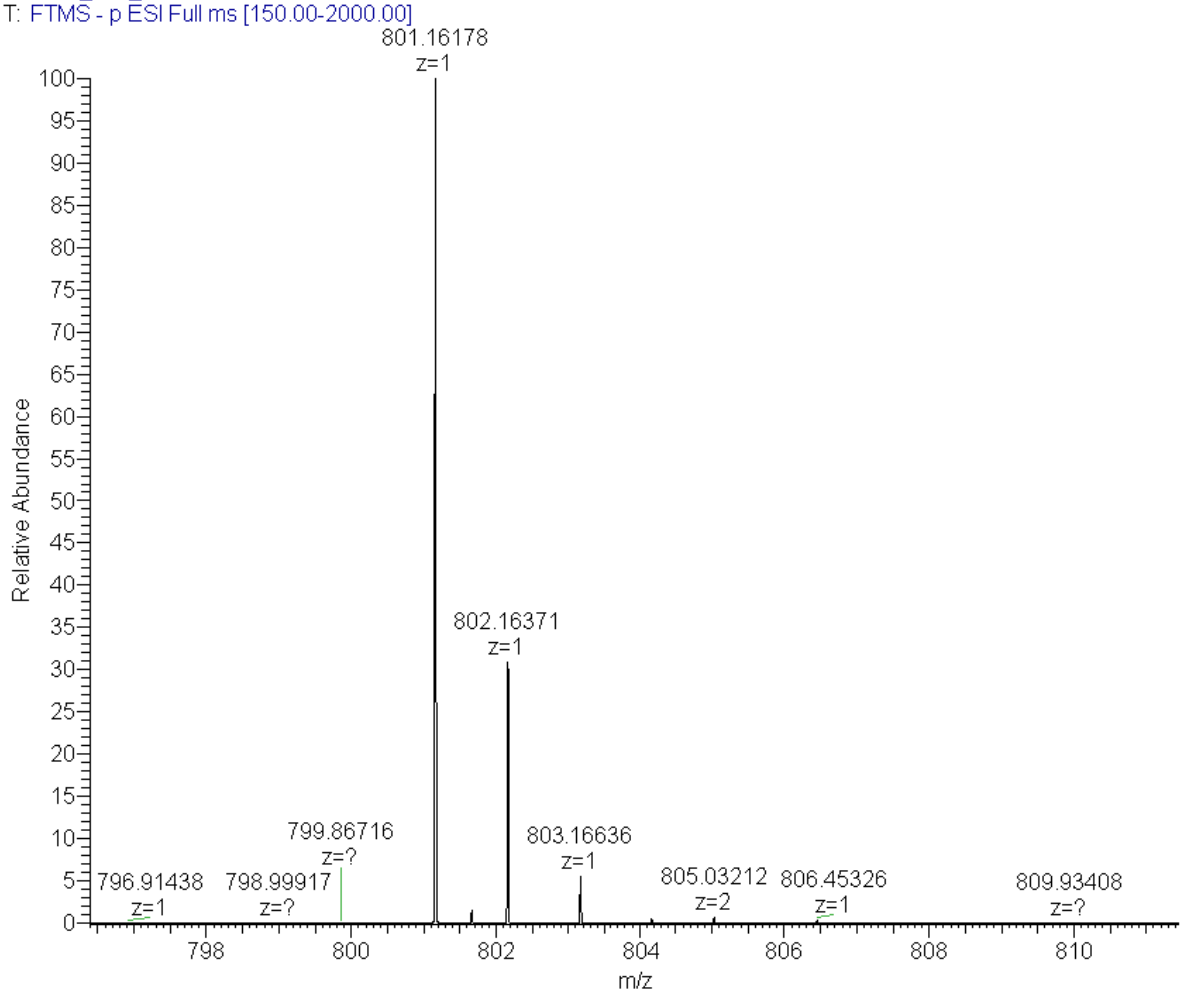

**^{1}H NMR**	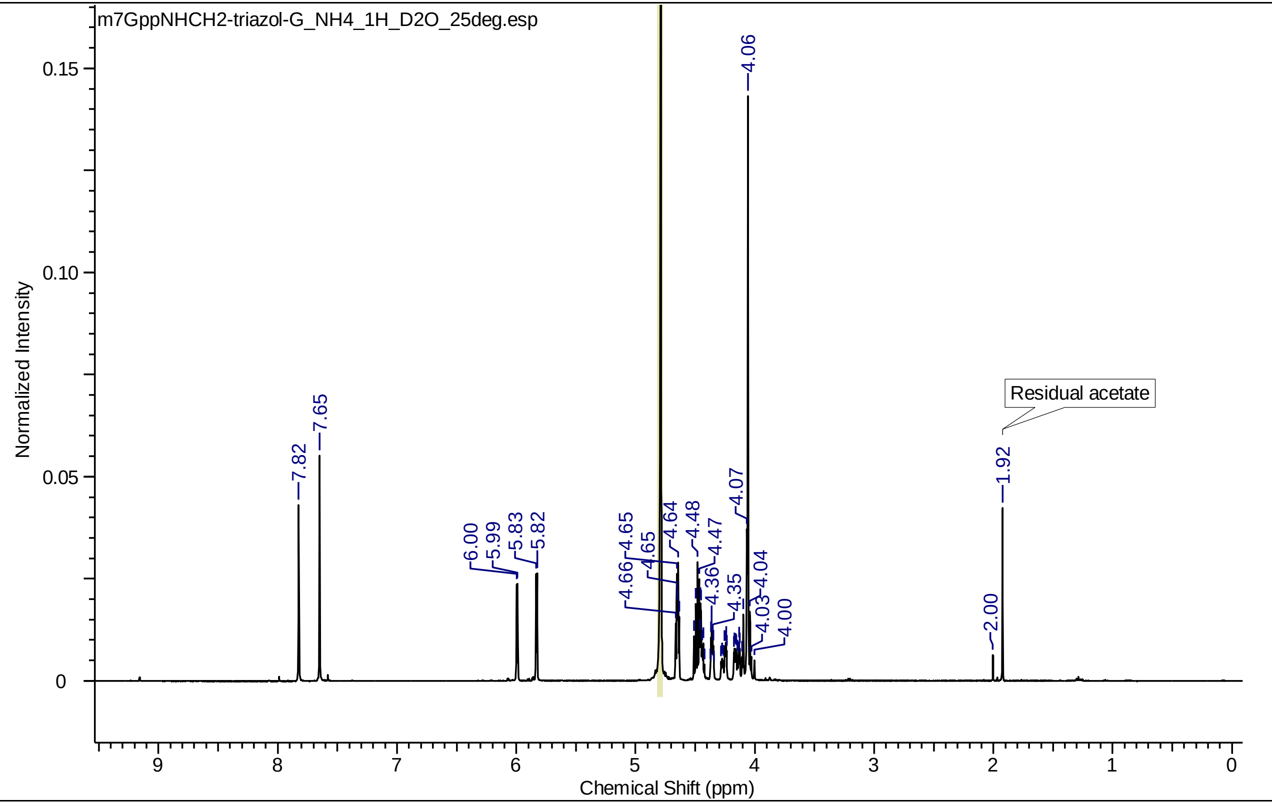
**^{31}P NMR**	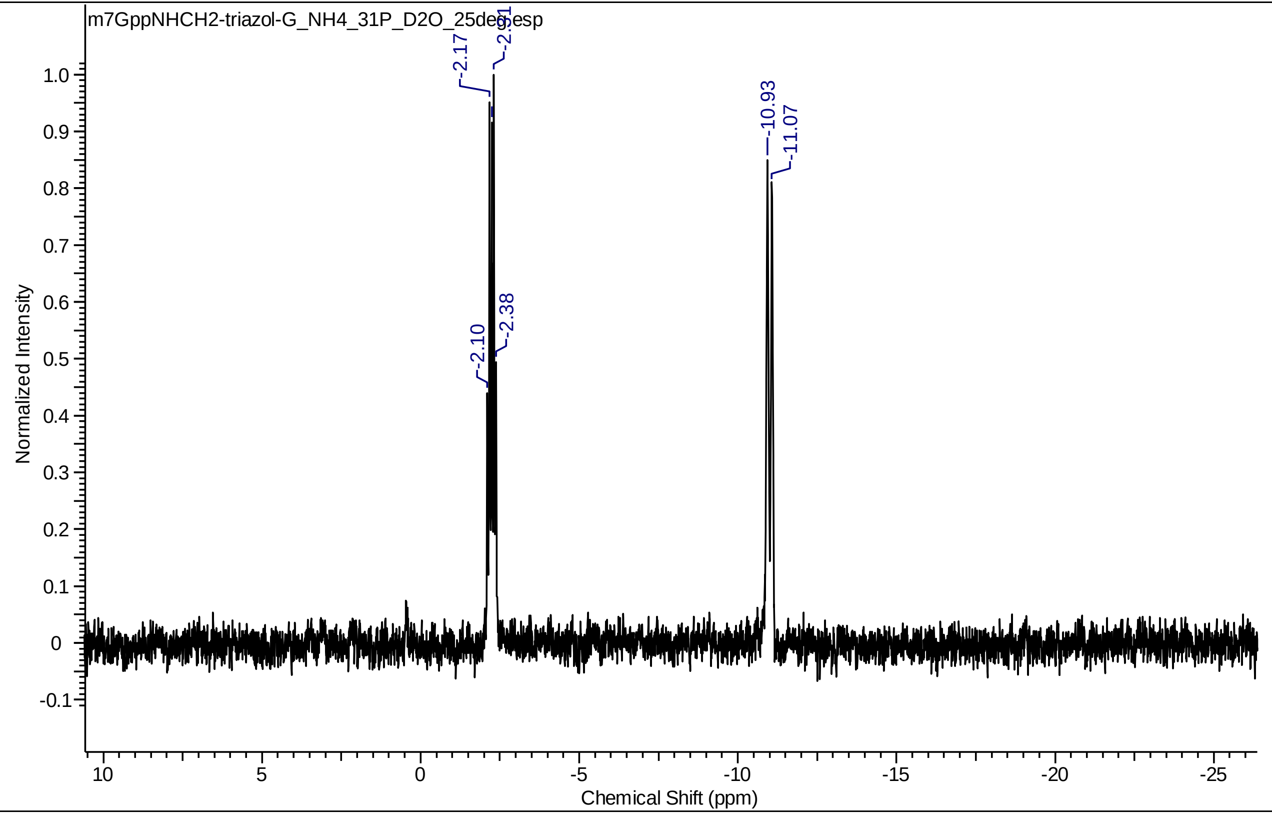

(6d) m^{7}GpppNHCH$_2$-triazole-G

**Structure**	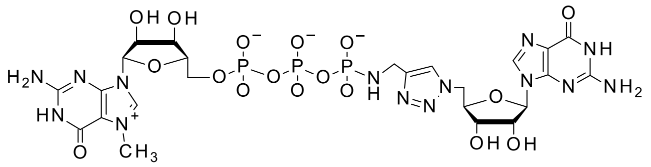

**Reaction HPLC profile**	Program C; before and after the reaction<br>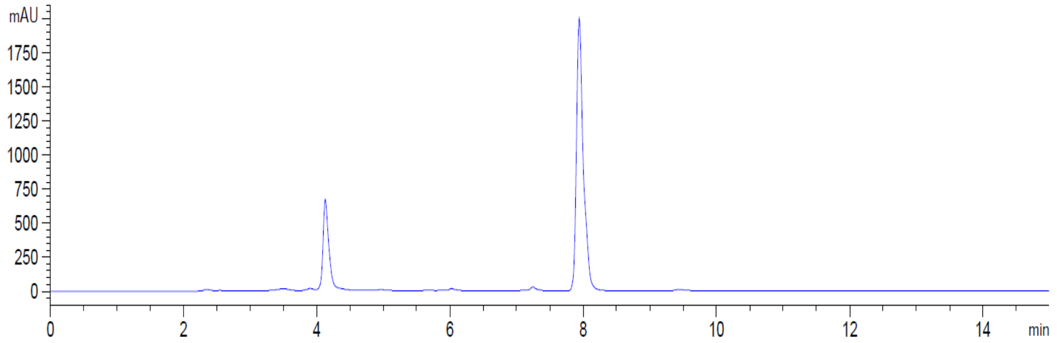<br><br>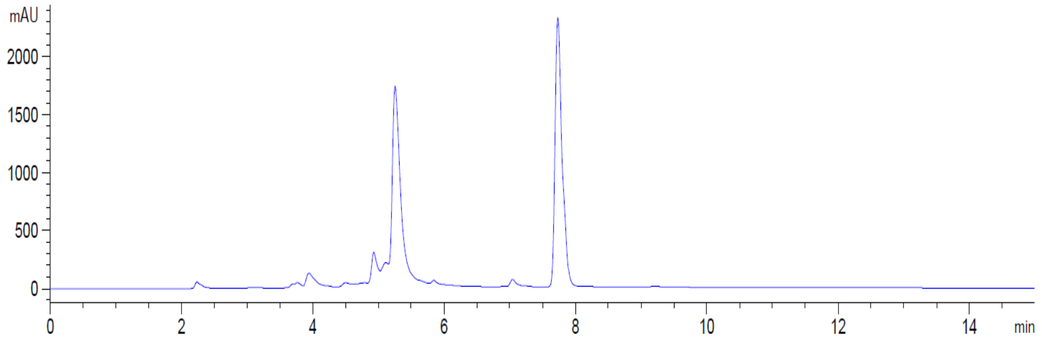
**Purified compound HPLC profile**	Program B<br>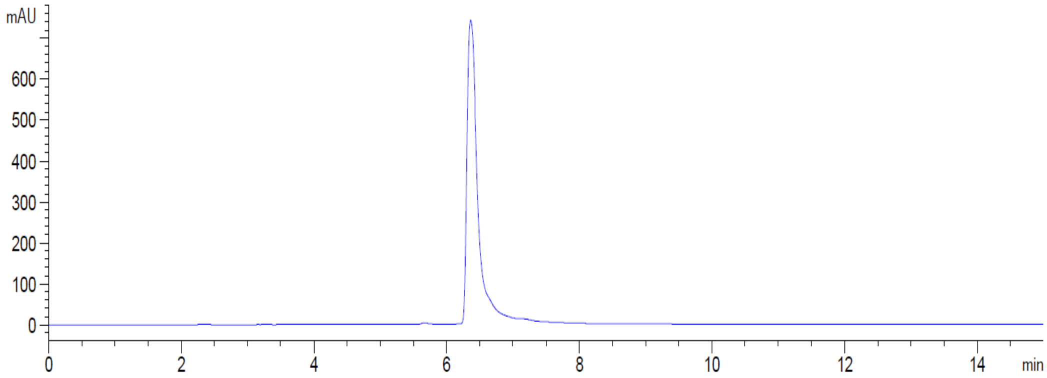
**HRMS**	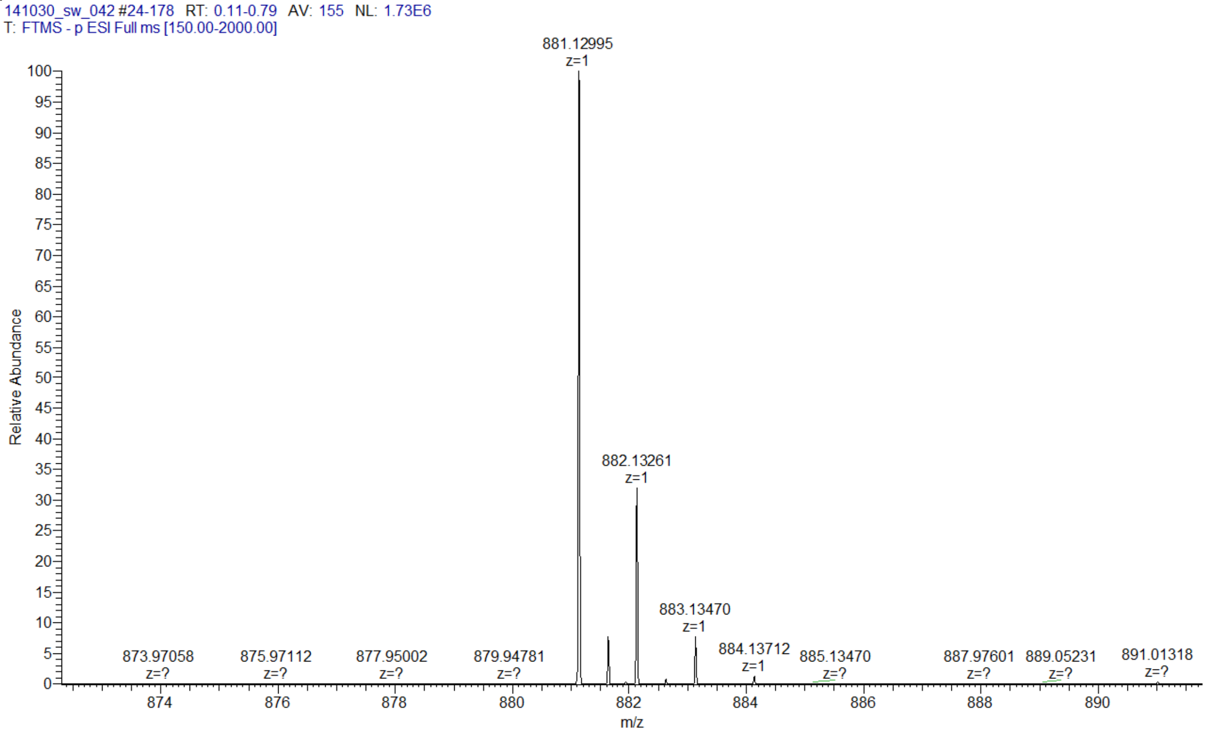

^{1}H NMR

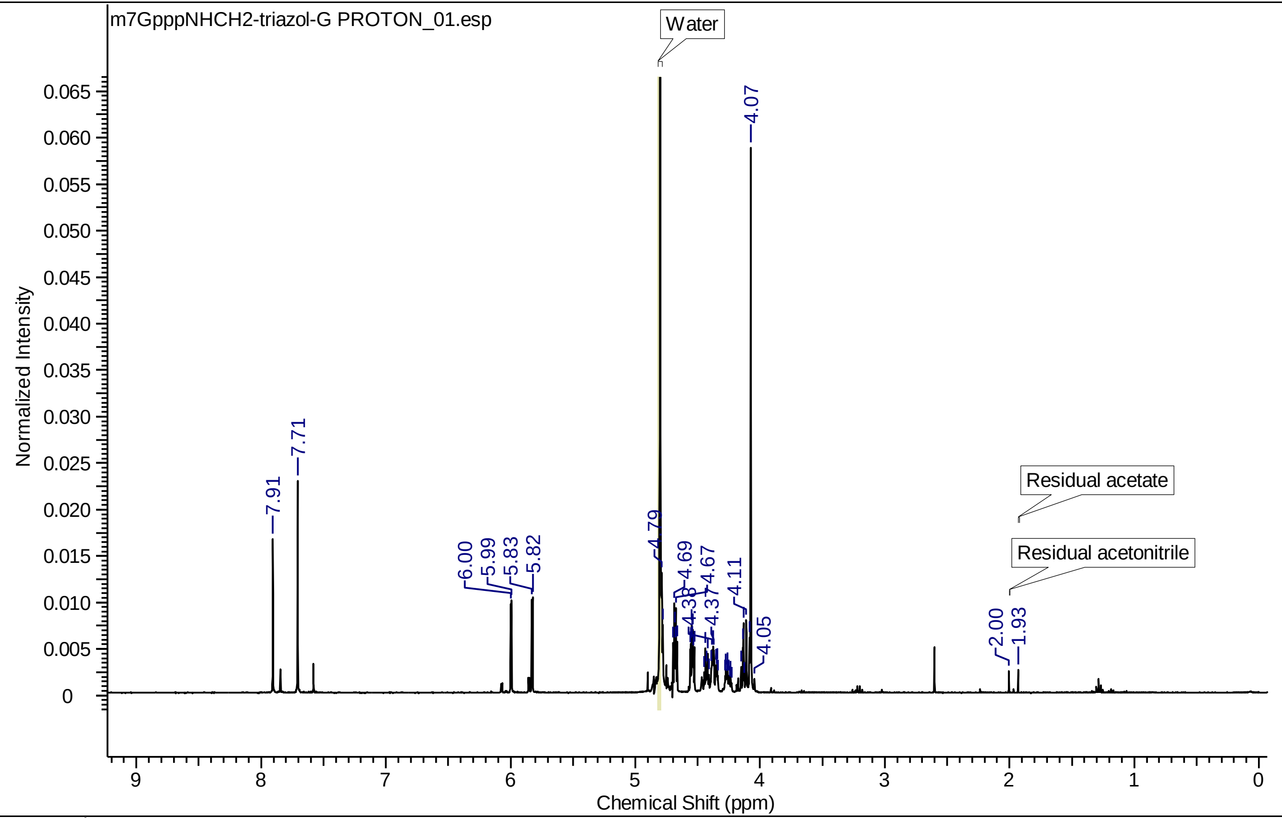

^{31}P NMR

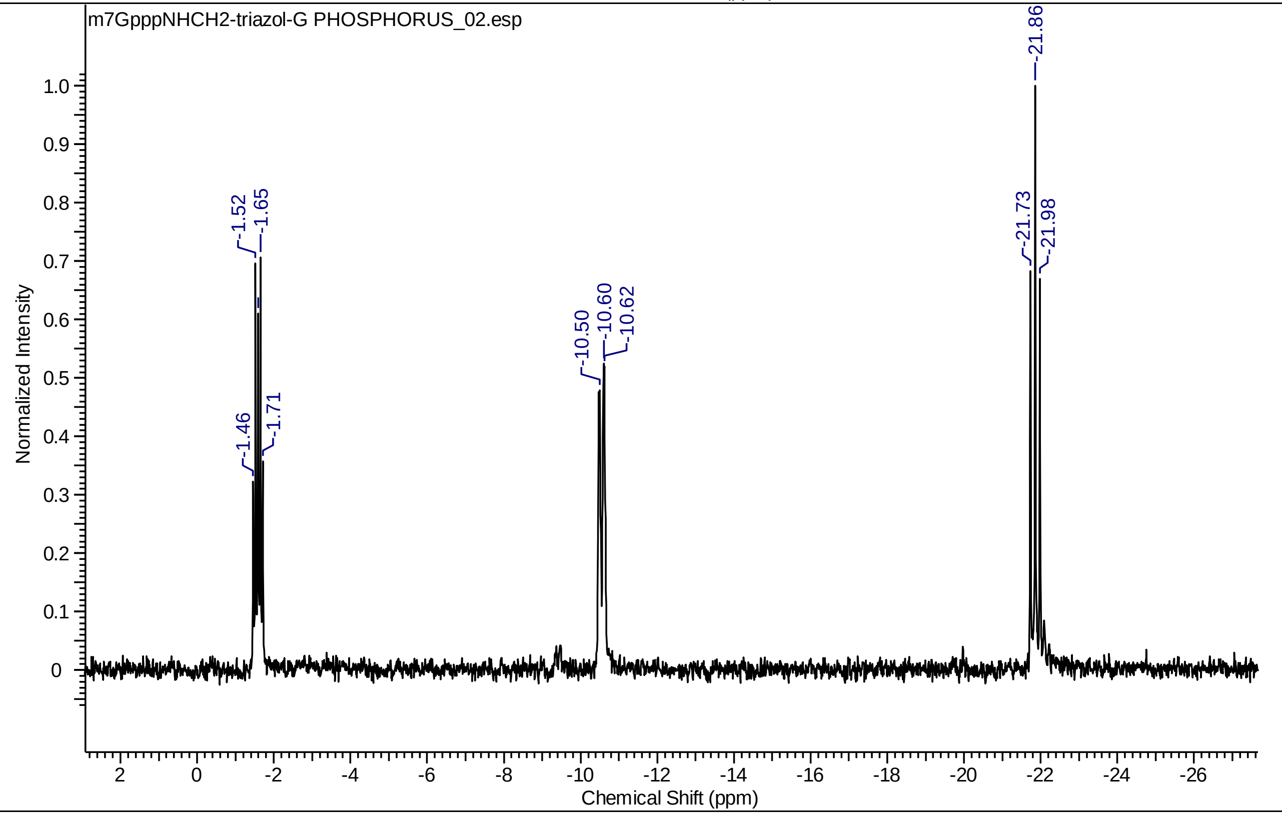

(7a) $m^7GpNHC_2H_4$-triazole-CH_2ppG

Structure

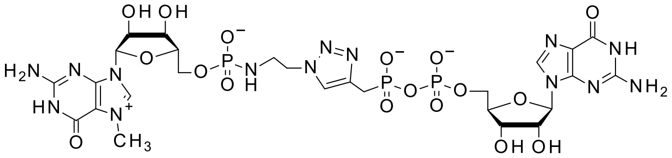

**Reaction HPLC profile**	Program B; before and after the reaction<br>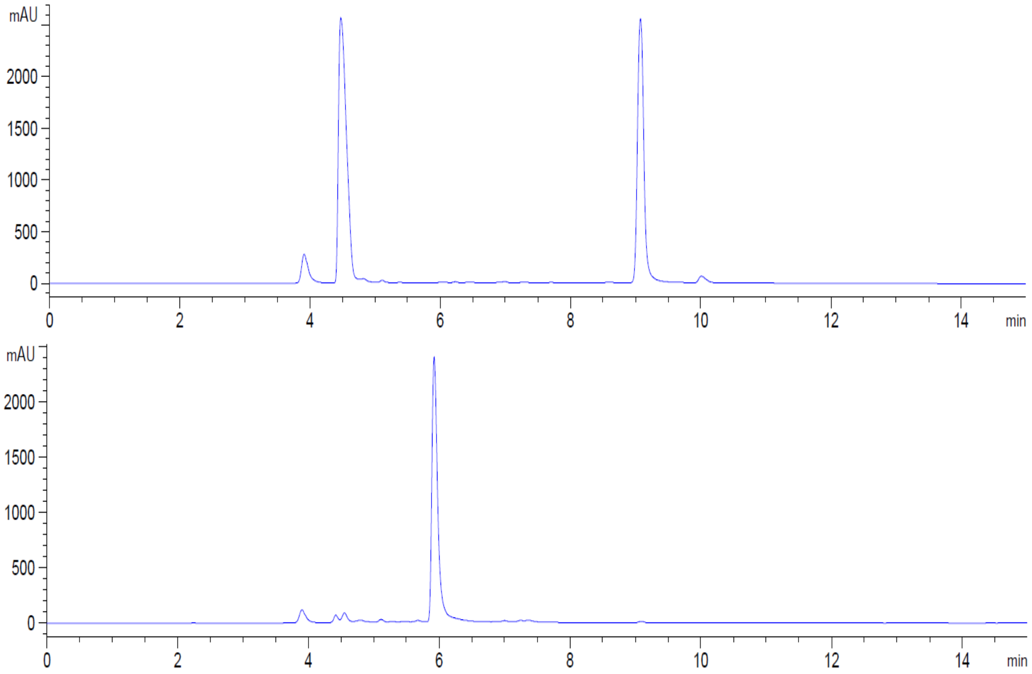
**Purified compound HPLC profile**	Program B<br>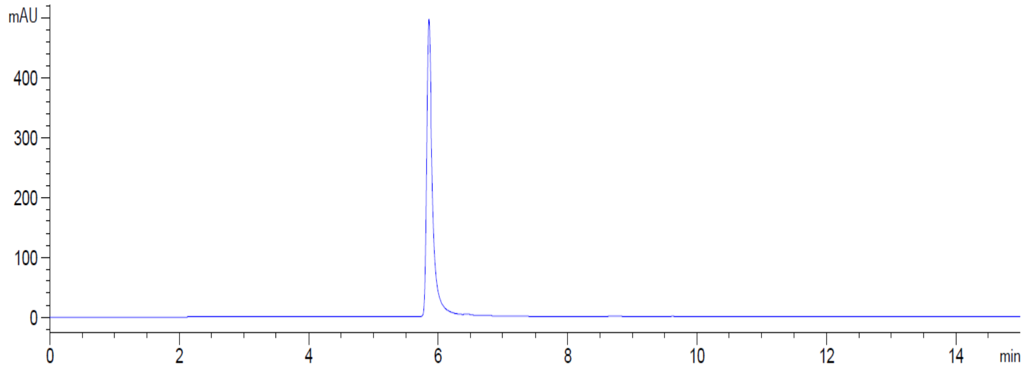
**HRMS**	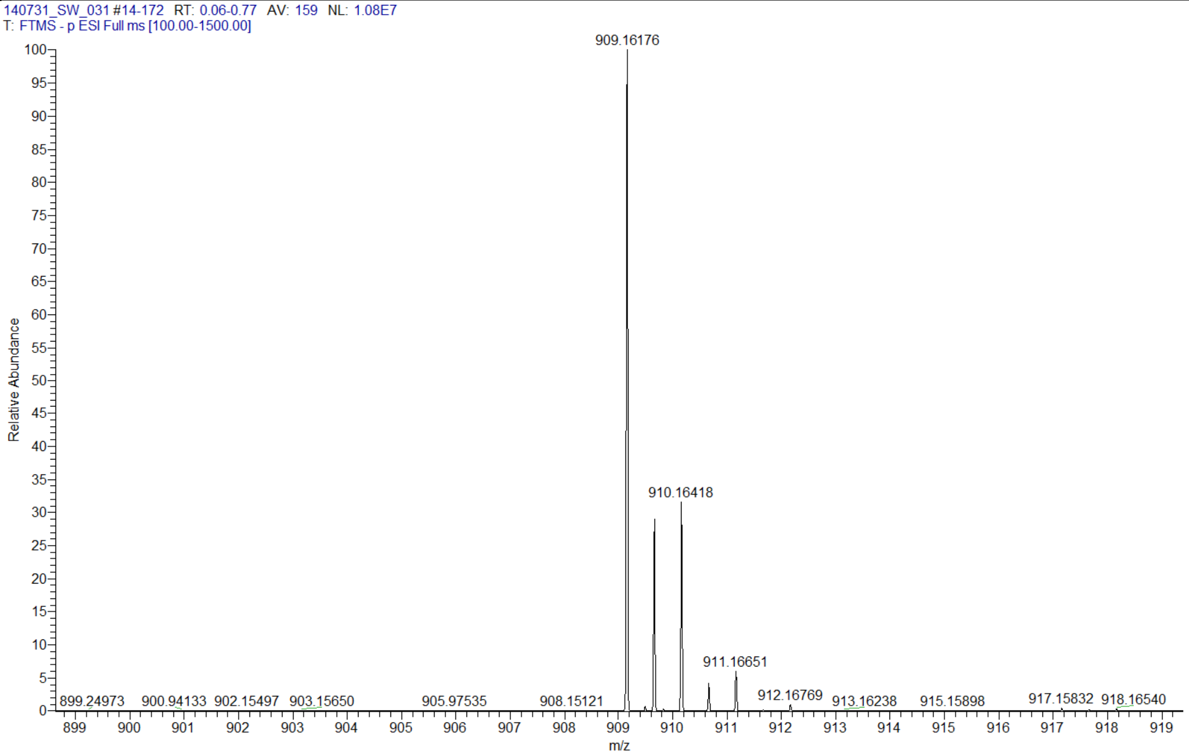

^{1}H NMR

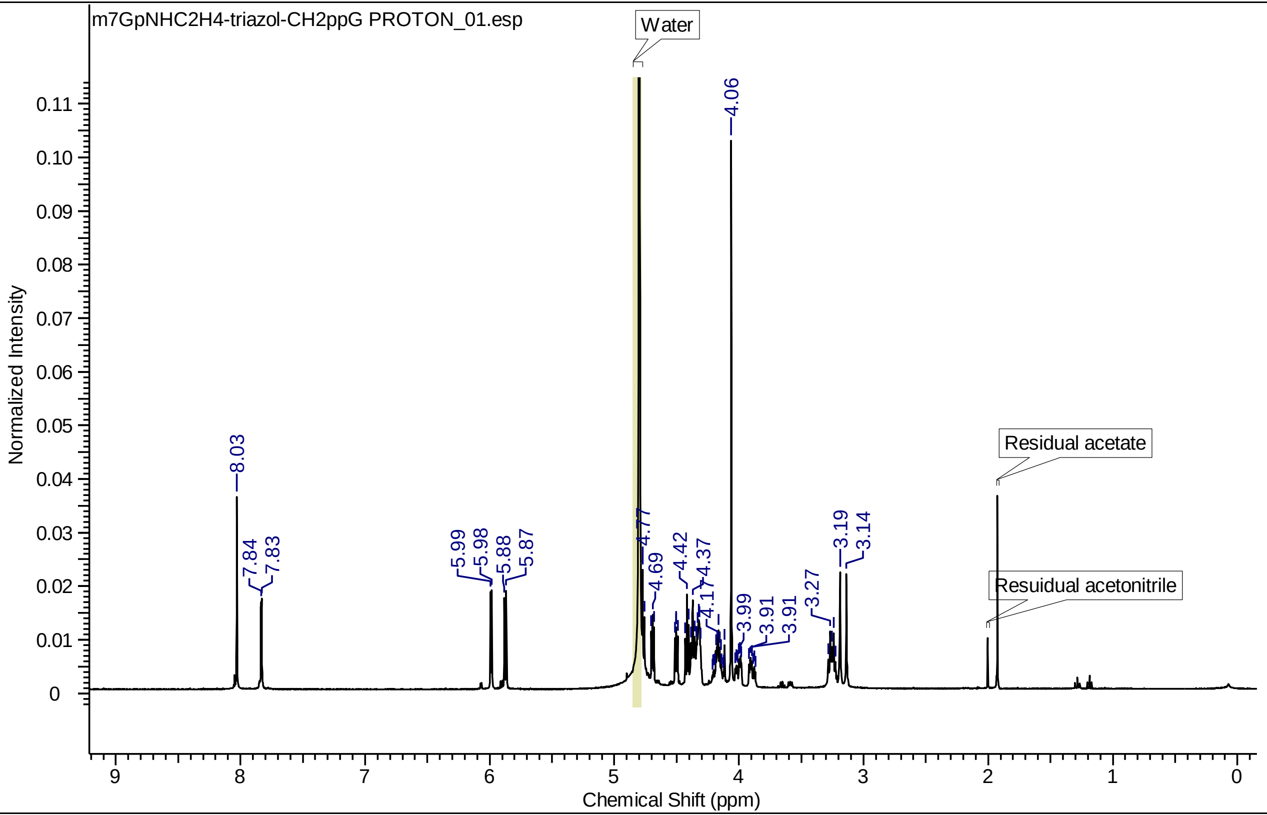

^{31}P NMR

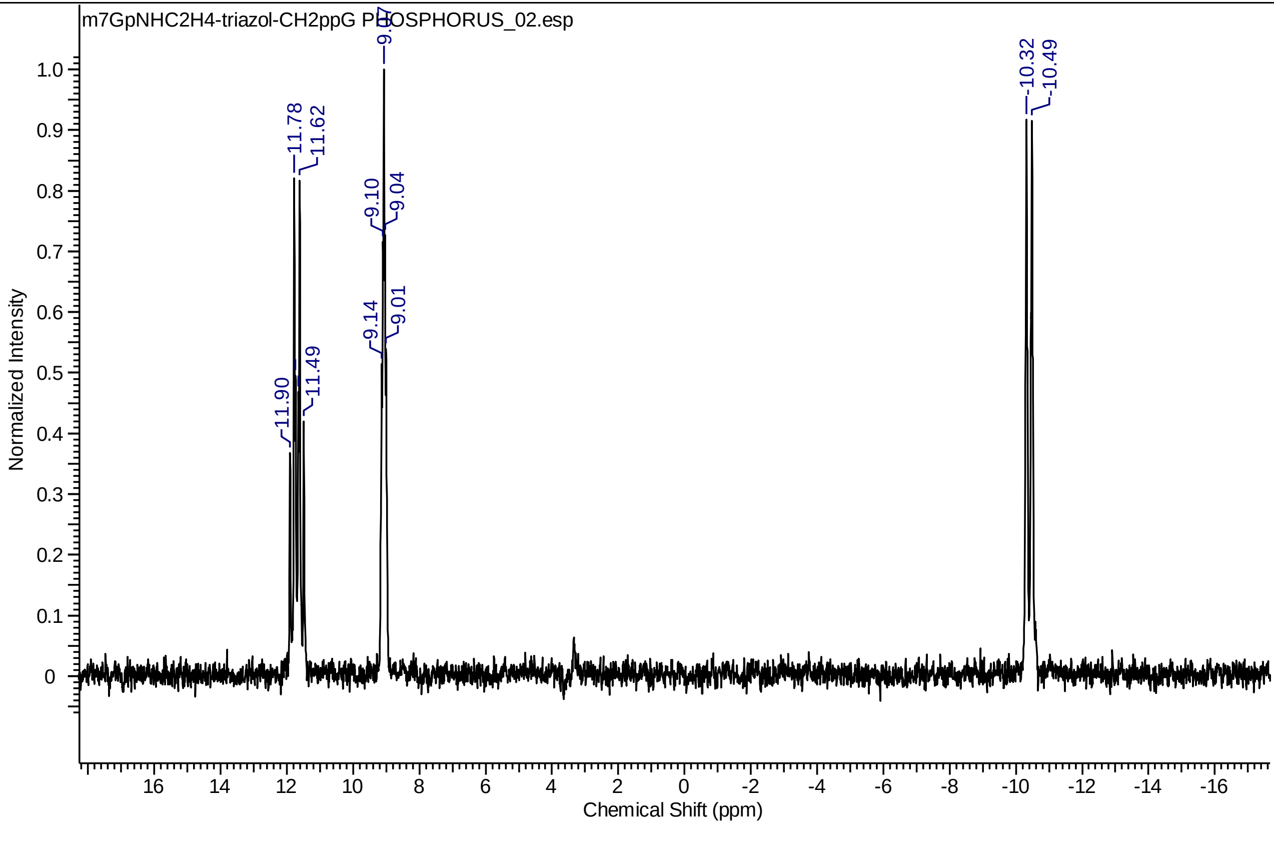

(7b) m^{7}GpNHC$_2$H$_4$-triazole-CH$_2$pppG

Structure

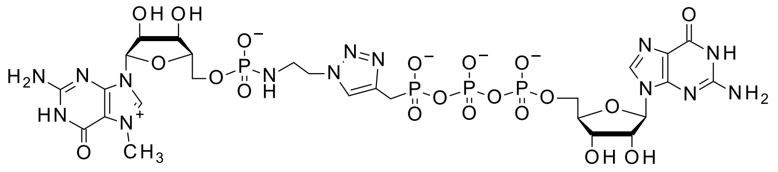

**Reaction HPLC profile**	Program B; before and after the reaction 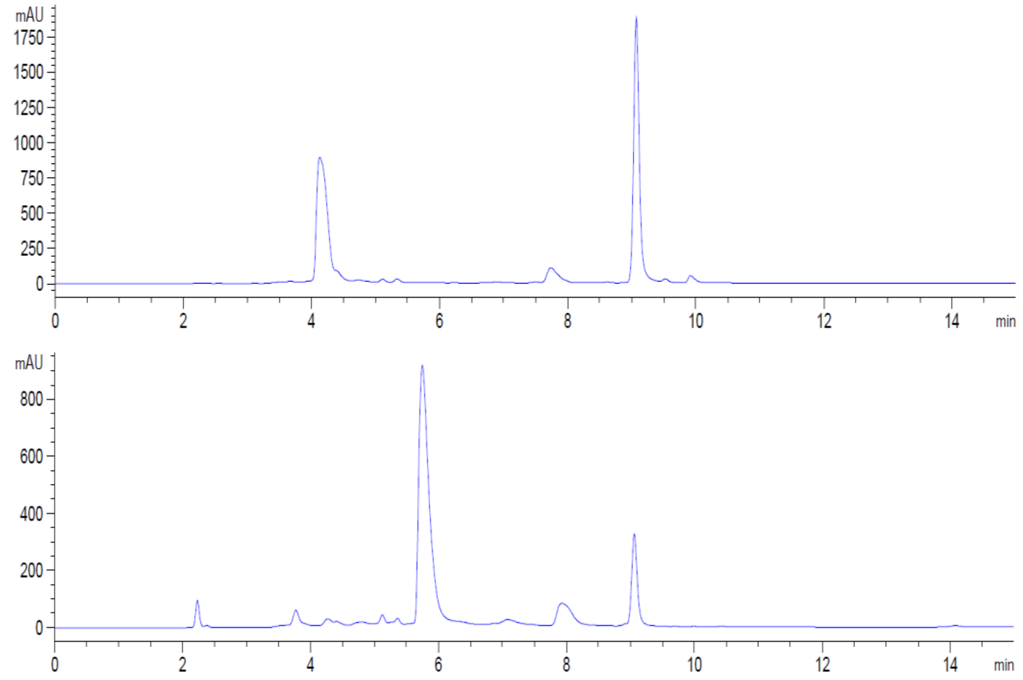
**Purified compound HPLC profile**	Program B 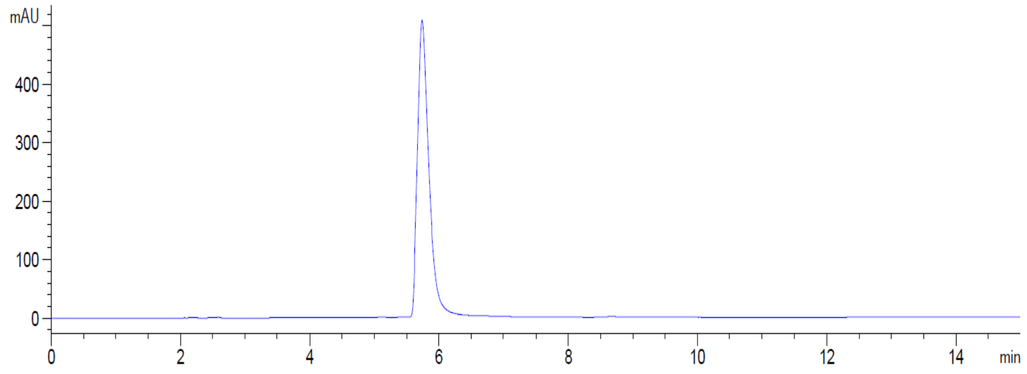
**HRMS**	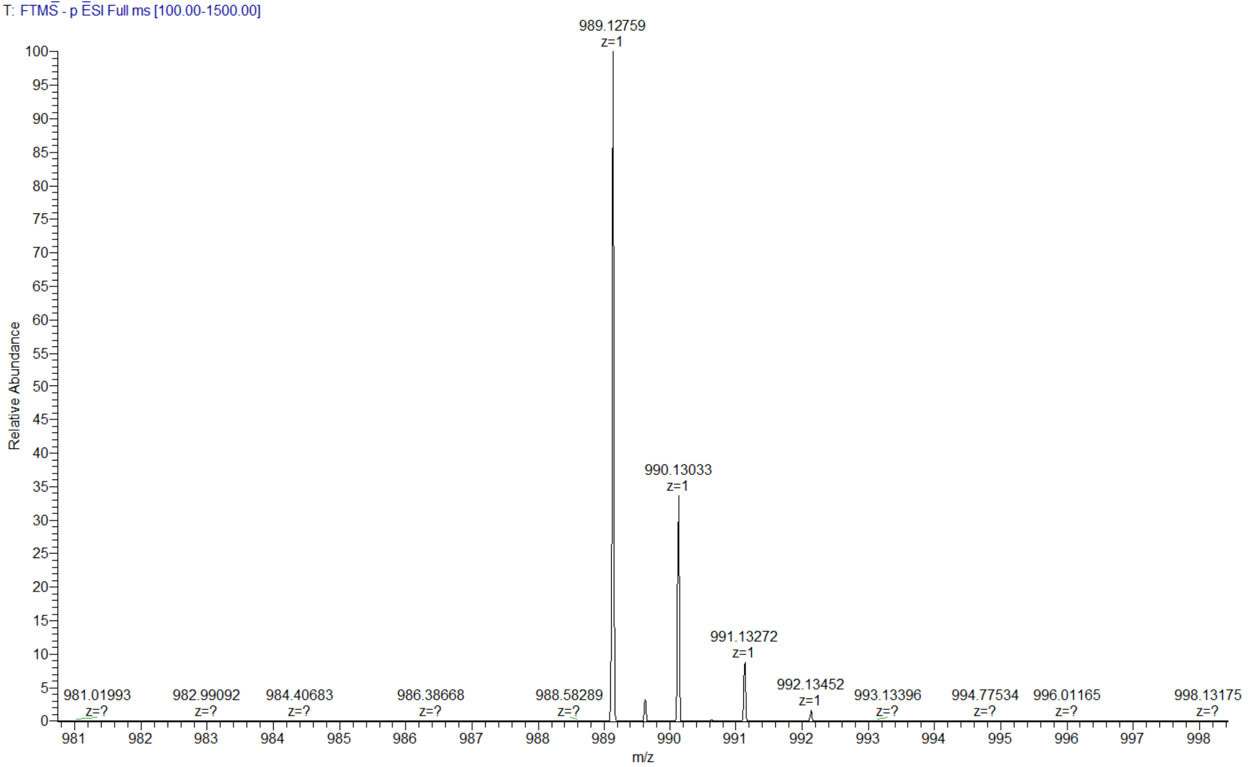

**^{1}H NMR**	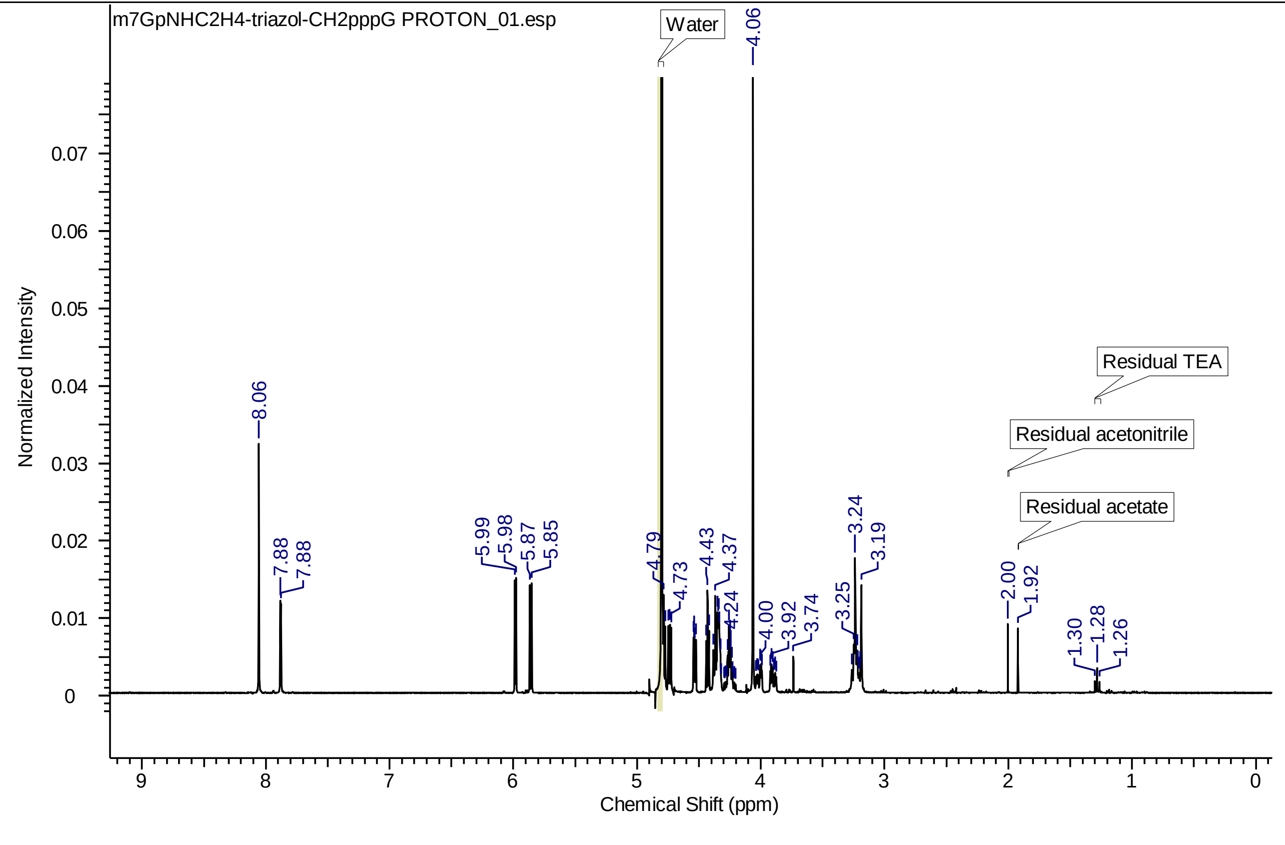
**^{31}P NMR**	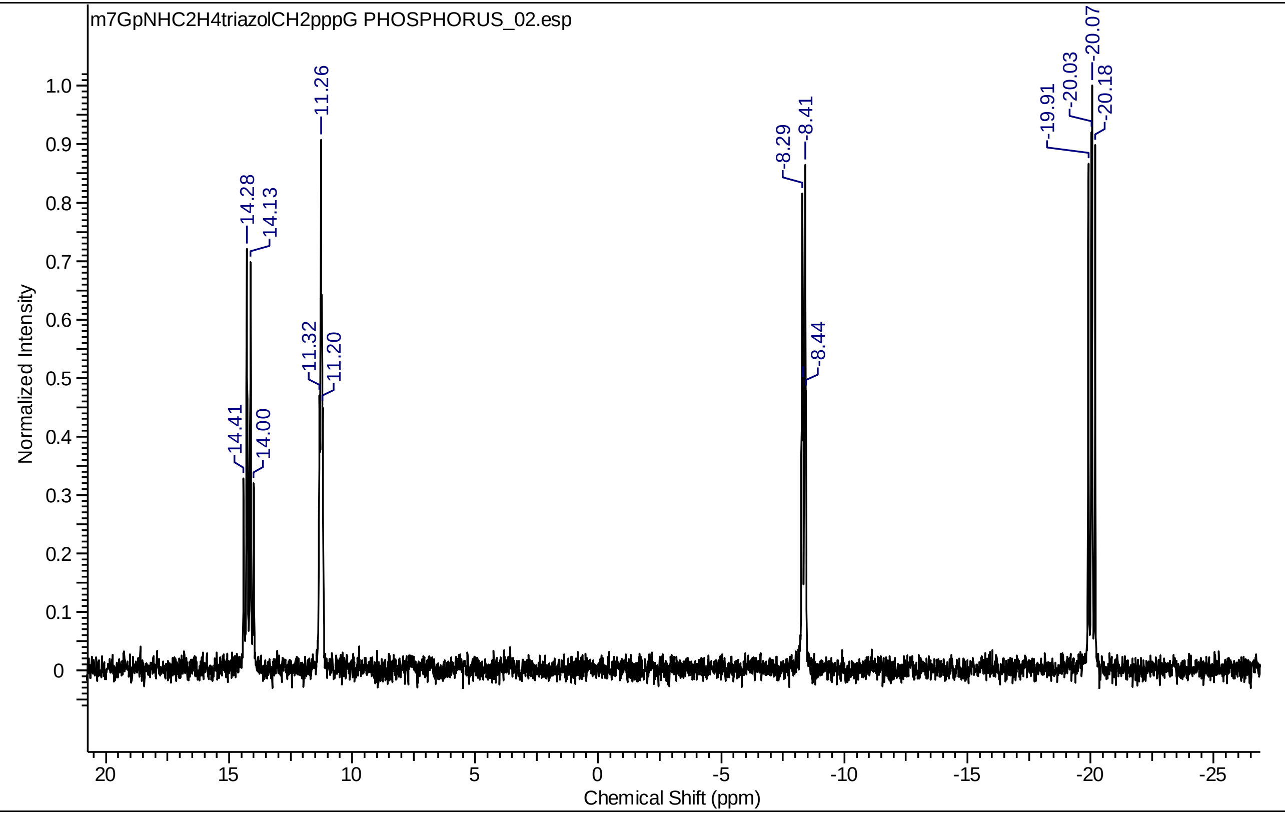

(7c) m^{7}GppCH$_2$-triazole-C$_2$H$_4$NHpG

**Structure**	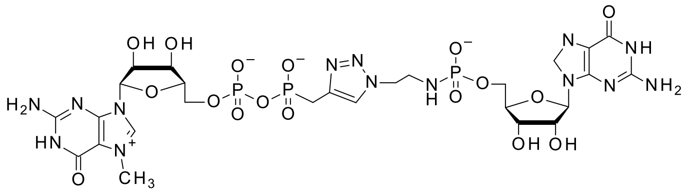

Reaction HPLC profile

Program C

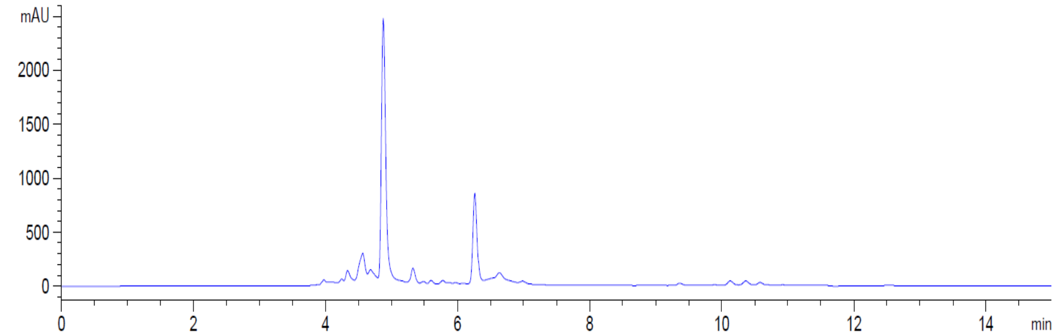

Purified compound HPLC profile

Program B

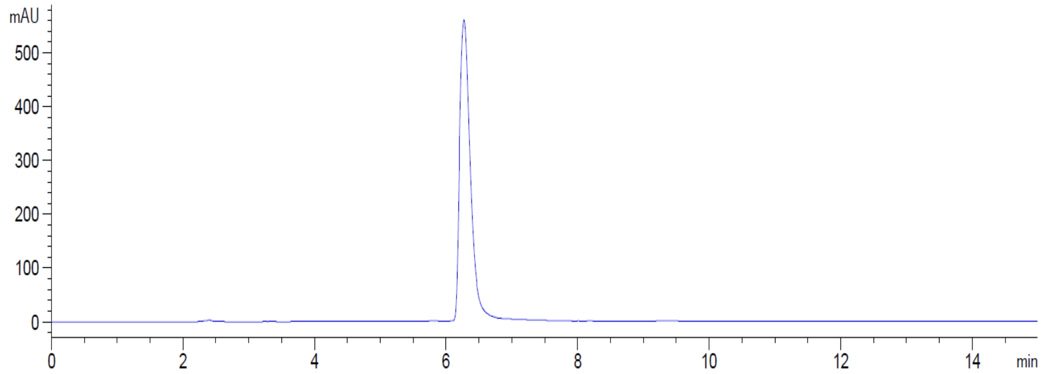

HRMS

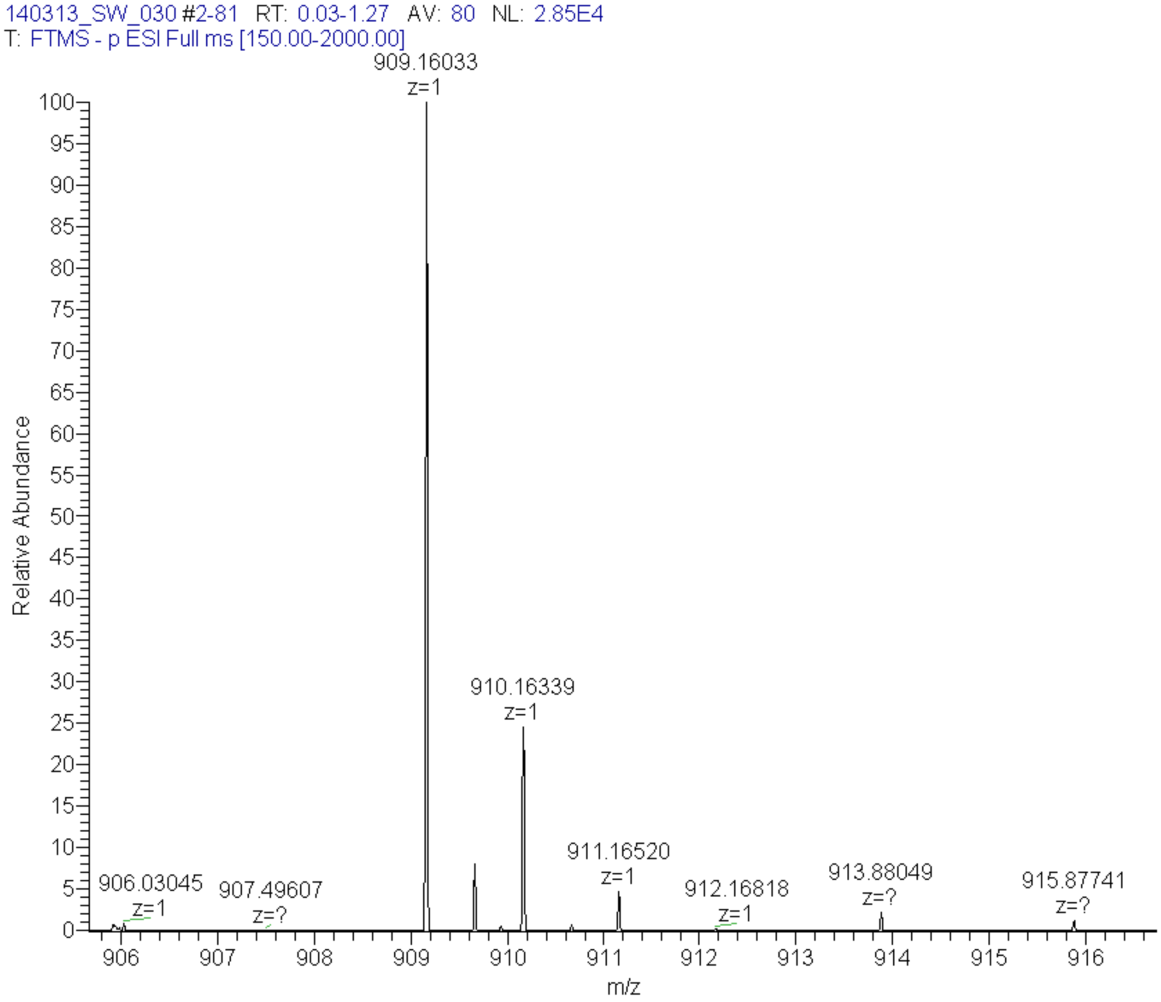

^{1}H NMR	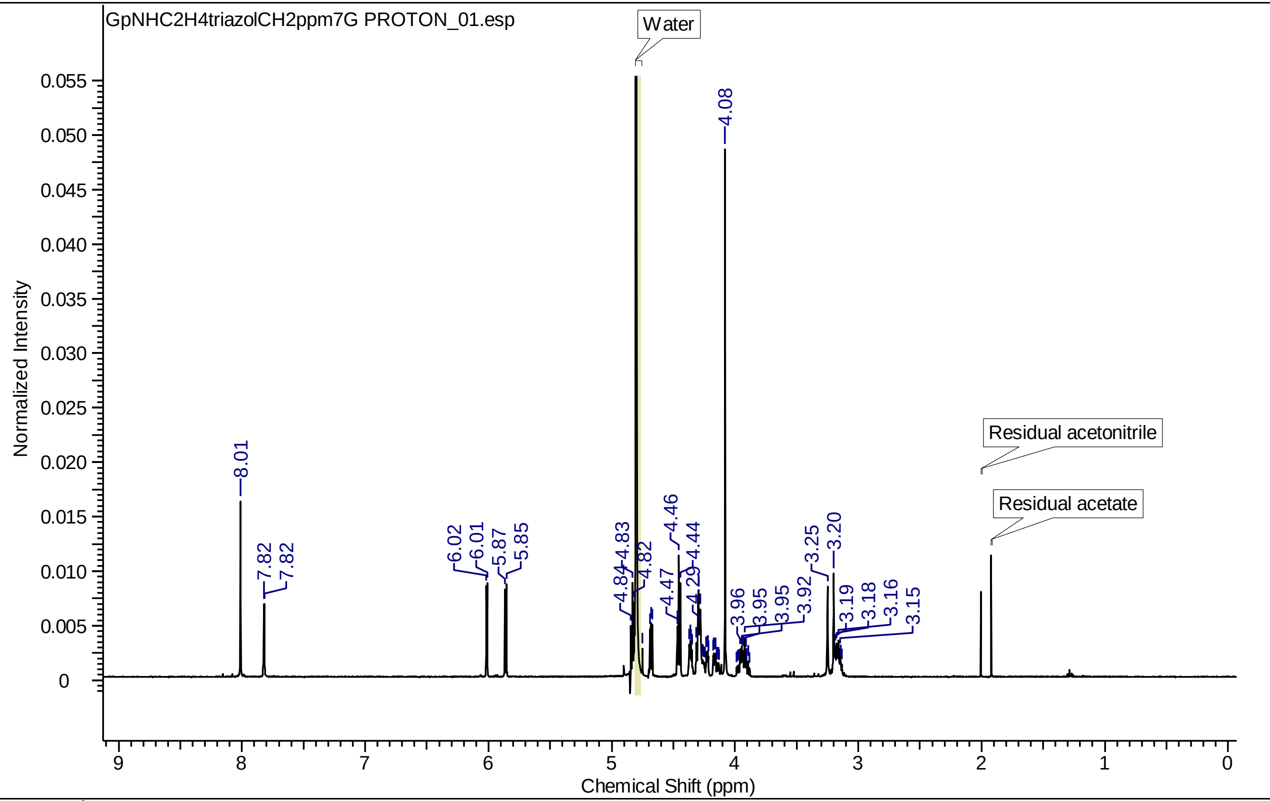
^{31}P NMR	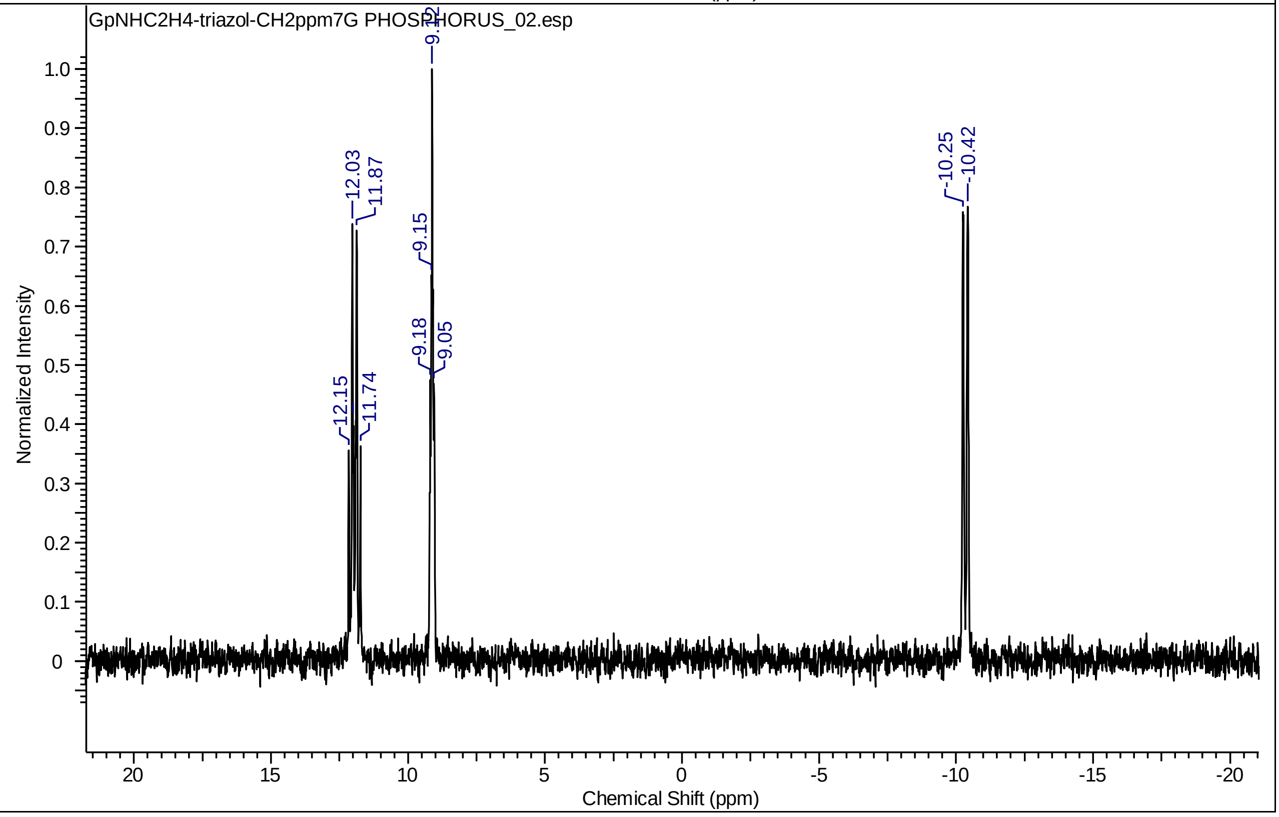

(7d) m^{7}GpppCH$_2$-triazole-C$_2$H$_4$NHpG

**Structure**	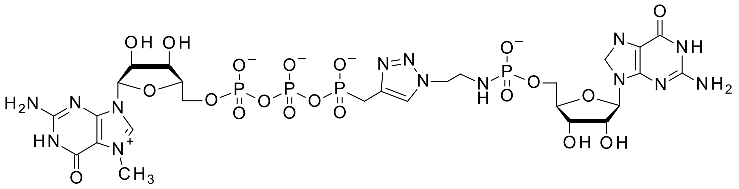

**Reaction HPLC profile**	Program B; before and after the reaction 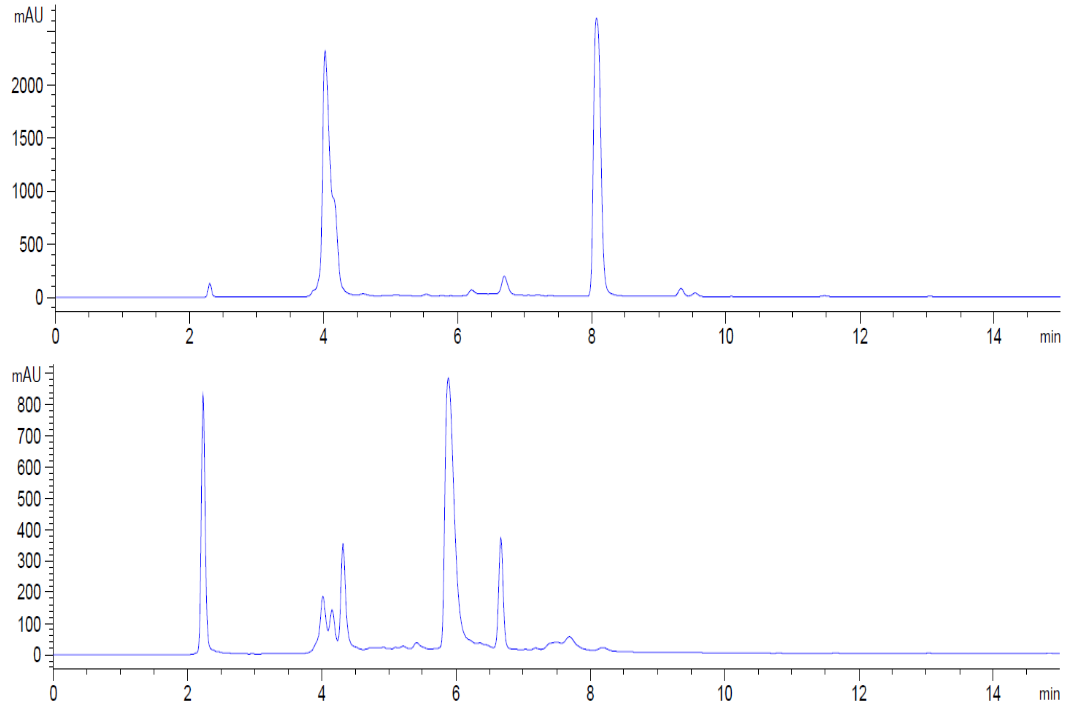
**Purified compound HPLC profile**	Program B 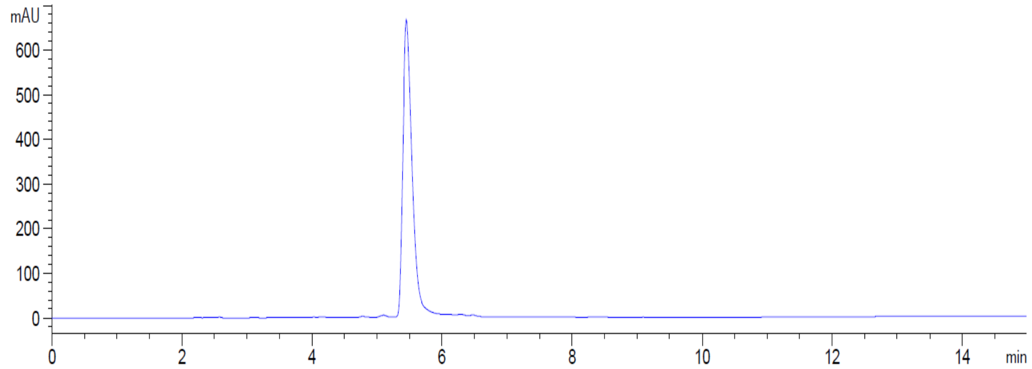
**HRMS**	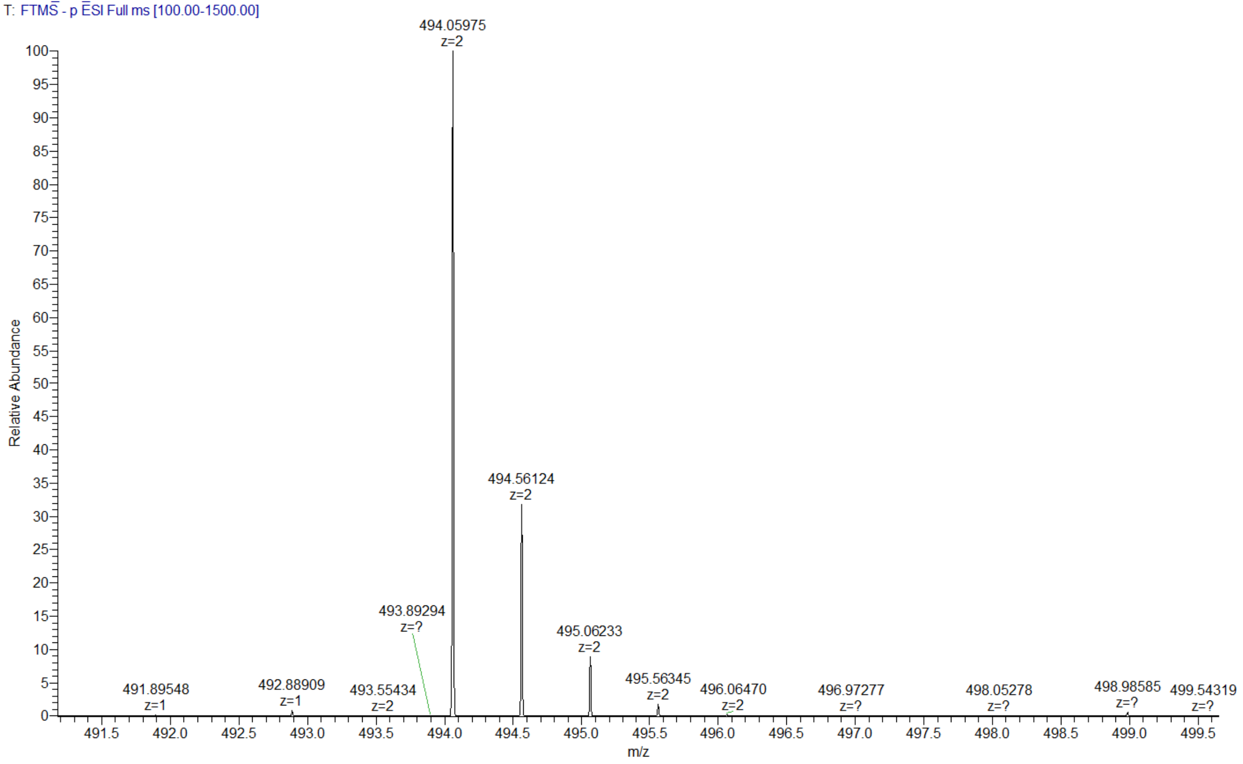

**^{1}H NMR**	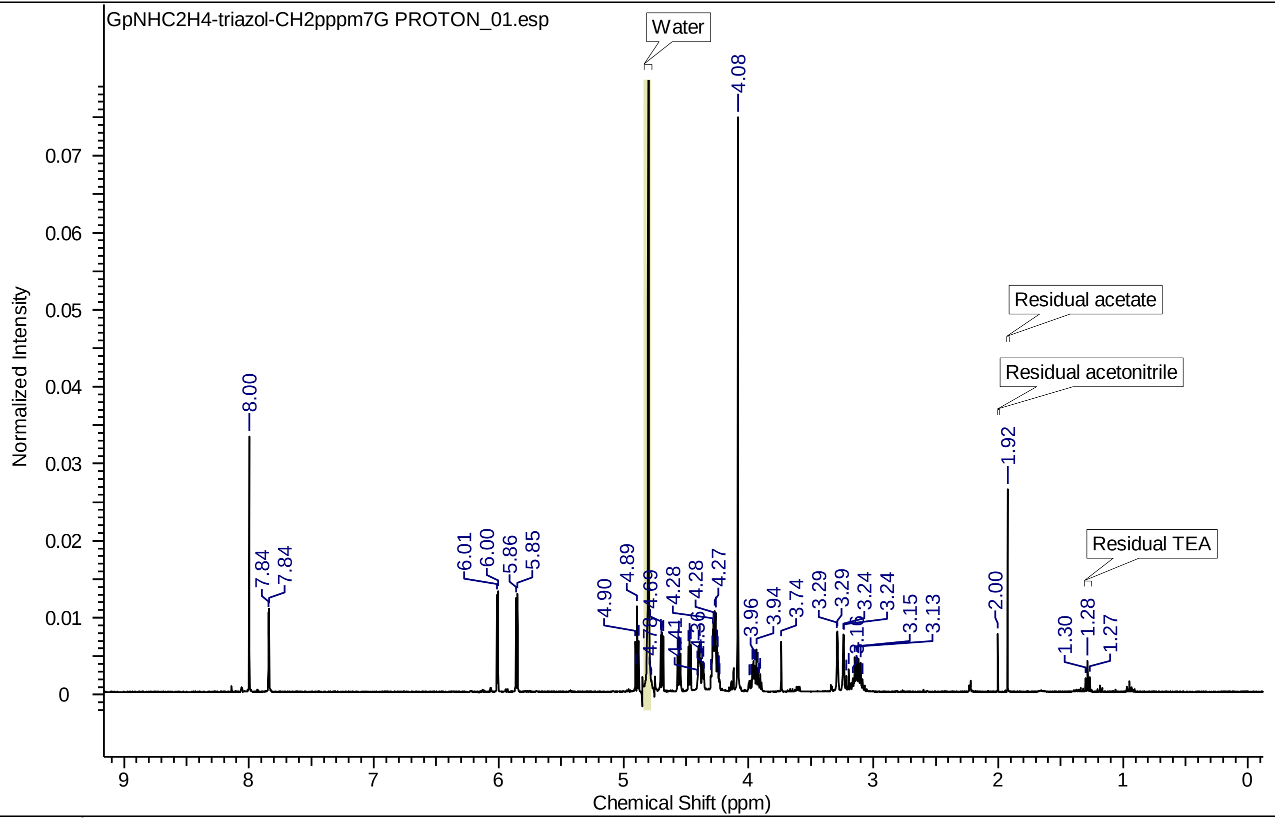
**^{31}P NMR**	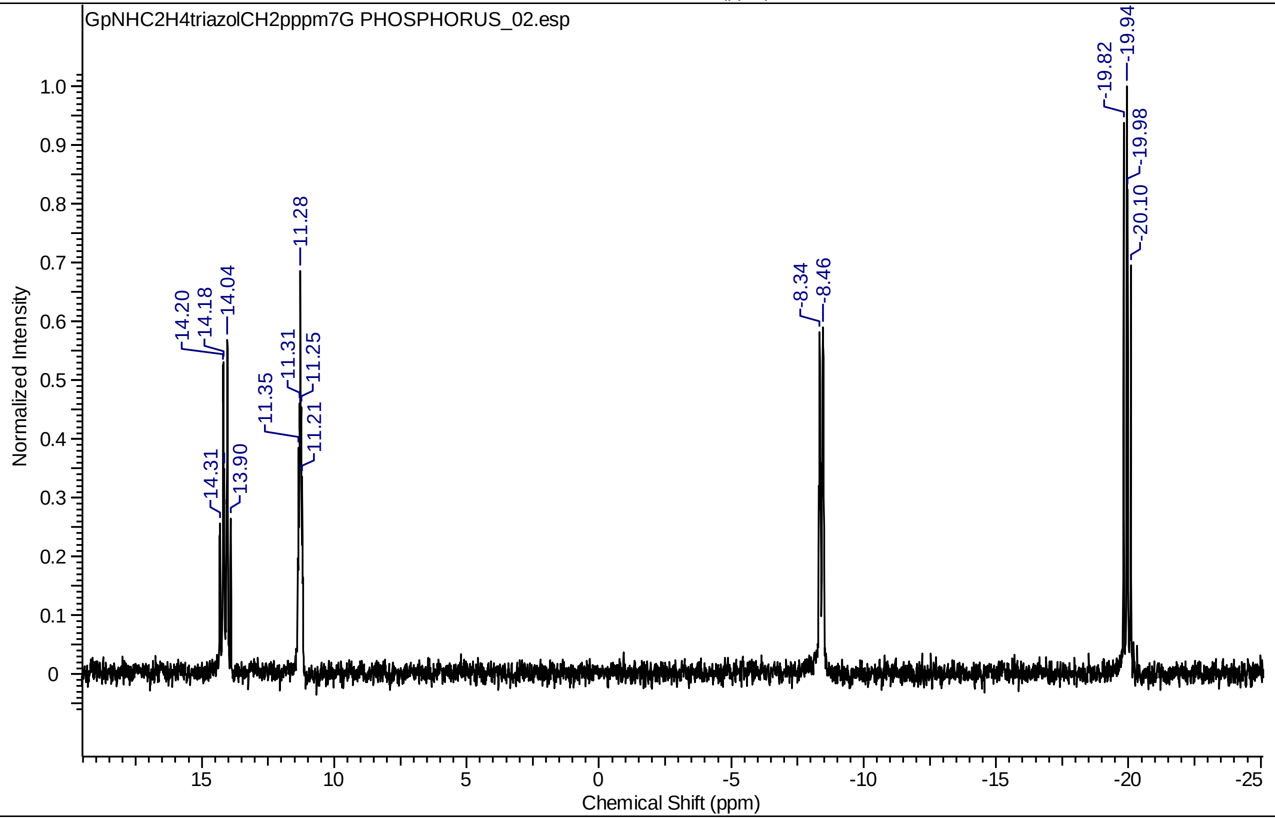

(8a) m^{7}GpNHC$_2$H$_4$-triazole-ppG

**Structure**	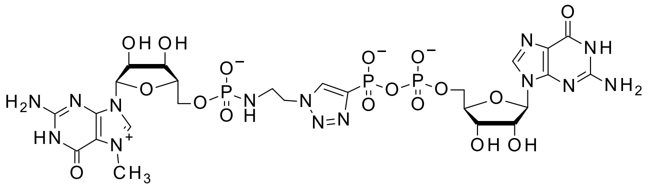

Reaction HPLC profile

Program B; before and after the reaction

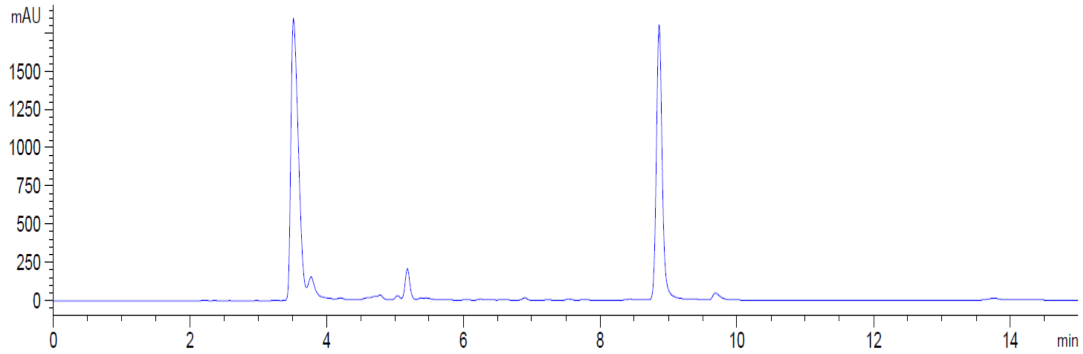

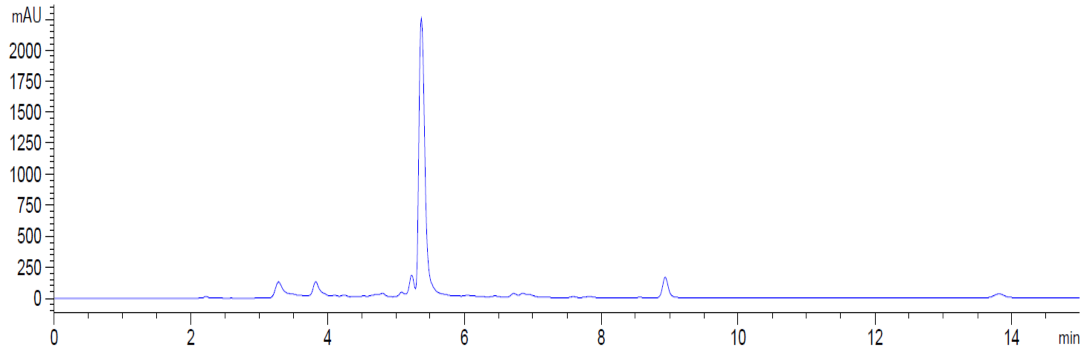

Purified compound HPLC profile

Program B

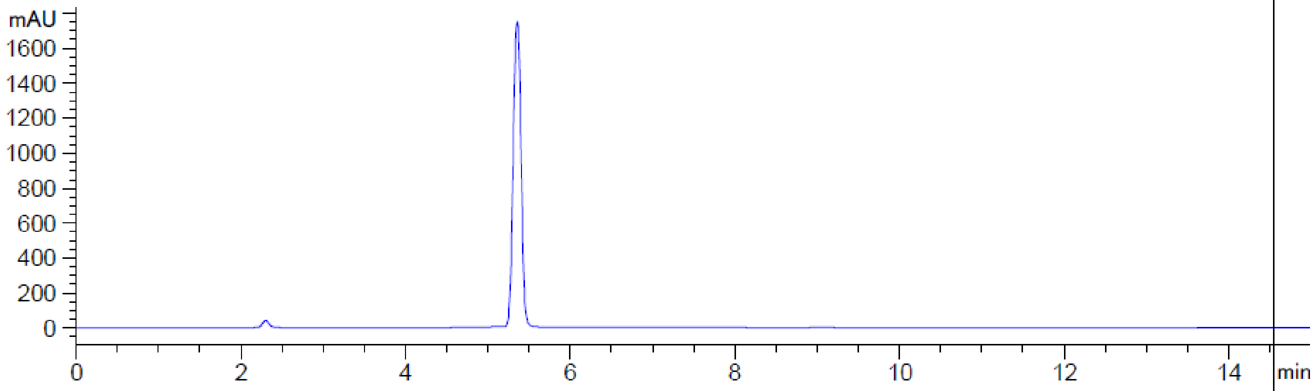

HRMS

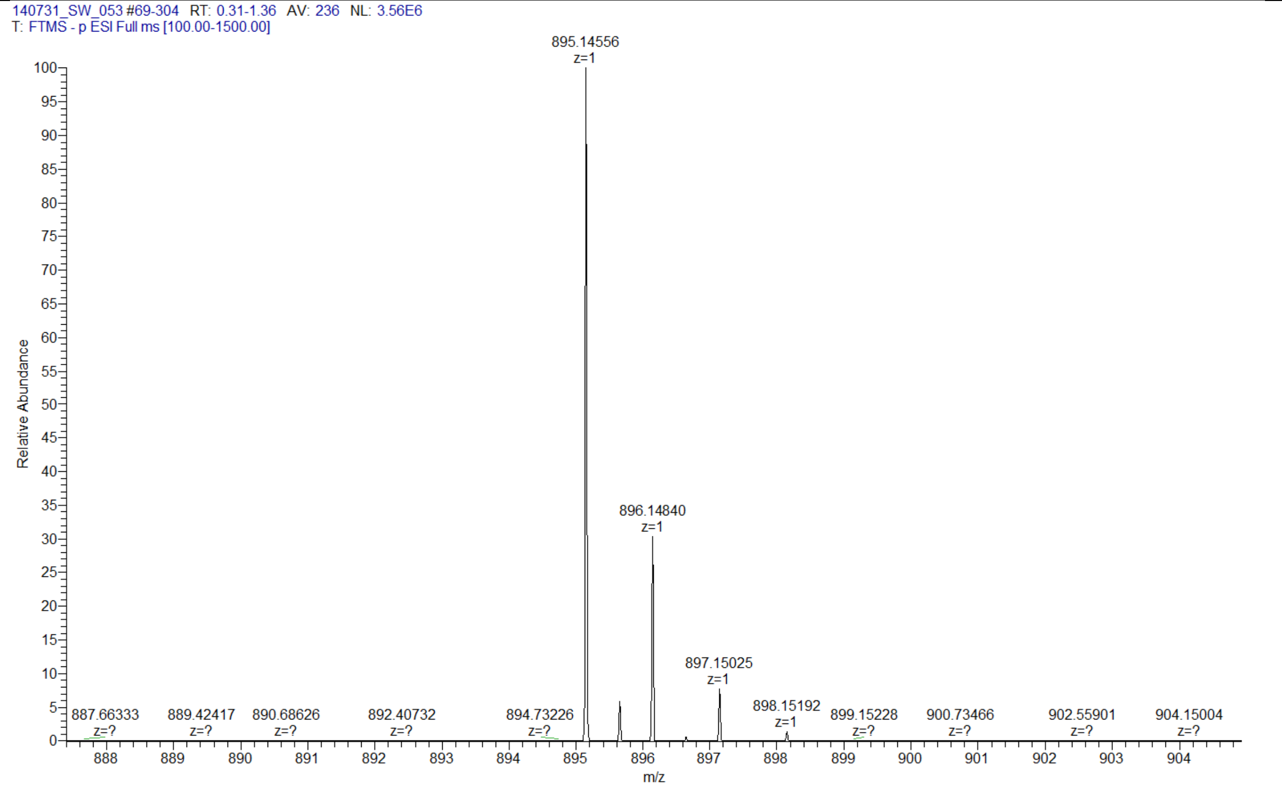

140731_SW_053 #69-304 RT: 0.31-1.36 AV: 236 NL: 3.56E6
T: FTMS - p ESI Full ms [100.00-1500.00]
895.14556 z=1
896.14840 z=1
897.15025 z=1
898.15192 z=1
887.66333 z=?
889.42417 z=?
890.68626 z=?
892.40732 z=?
894.73226 z=?
899.15228 z=?
900.73466 z=?
902.55901 z=?
904.15004 z=?
Relative Abundance
m/z

^{1}H NMR

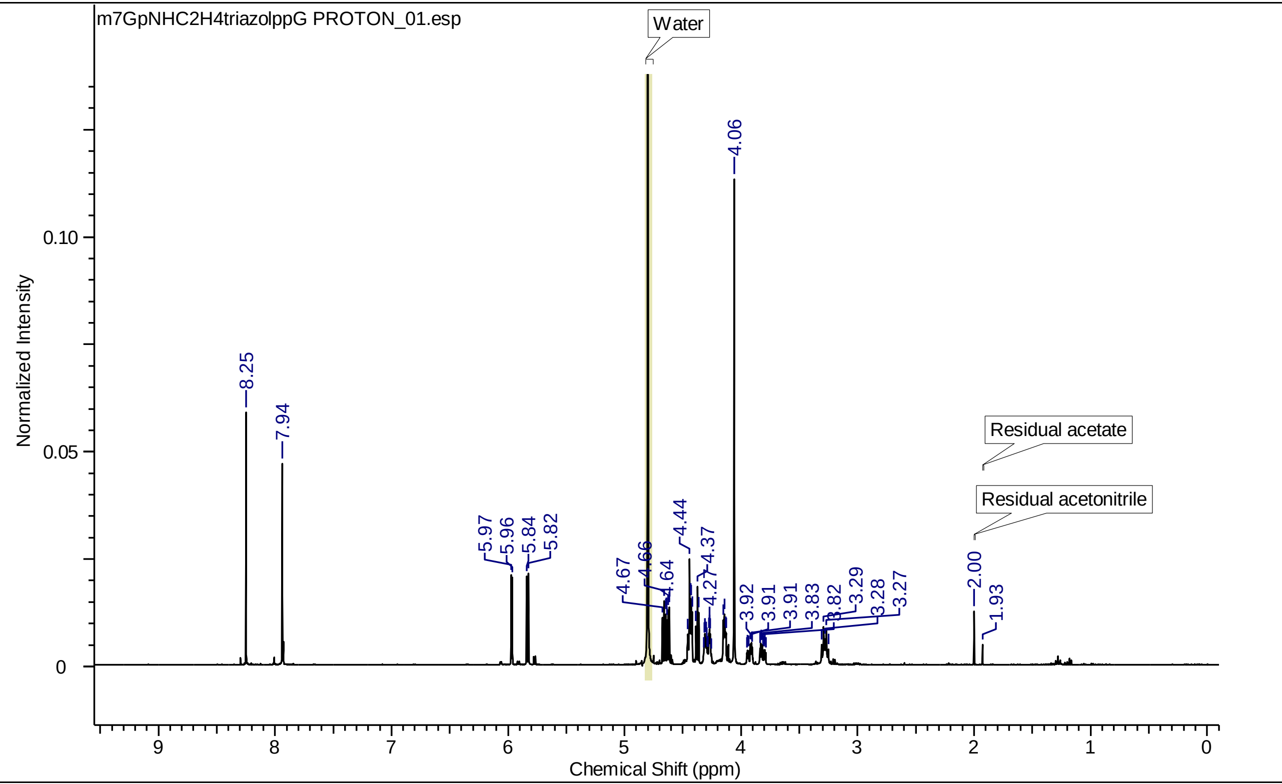

m7GpNHC2H4triazolppG PROTON_01.esp
Water
Residual acetate
Residual acetonitrile
8.25
7.94
5.97
5.96
5.84
5.82
4.67
4.66
4.64
4.44
4.37
4.27
4.06
3.92
3.91
3.91
3.83
3.82
3.29
3.28
3.27
2.00
1.93
Normalized Intensity
Chemical Shift (ppm)

^{31}P NMR	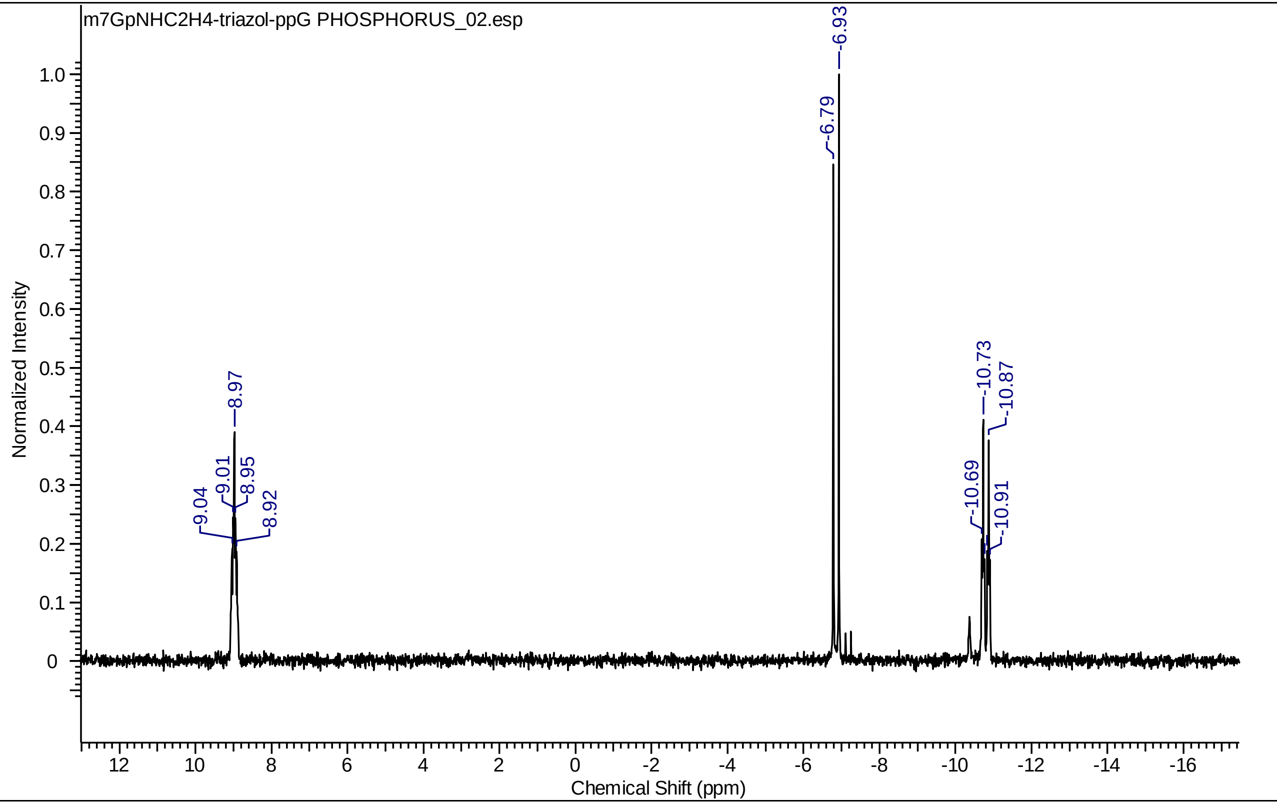

(8b) m^{7}GpNHC$_2$H$_4$-triazole-pppG

Structure	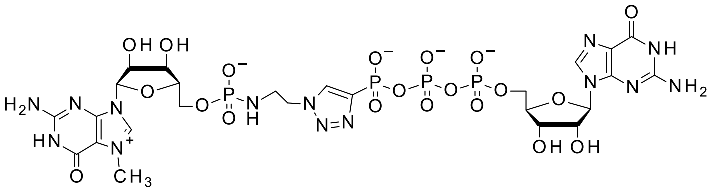
Reaction HPLC profile	Program B; before and after the reaction<br>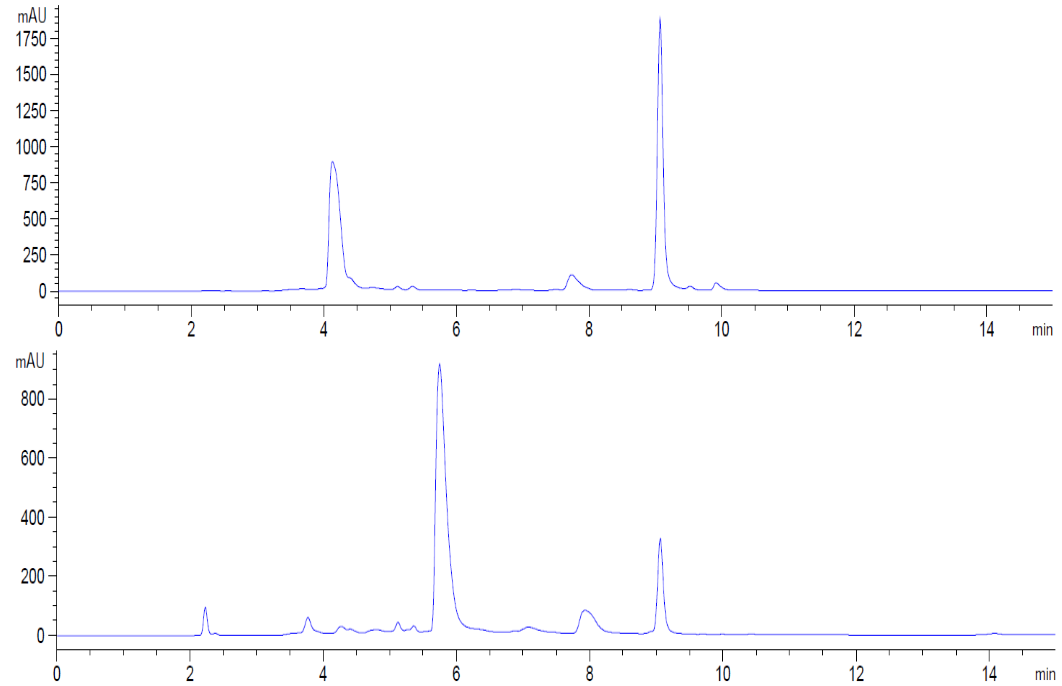

Purified compound HPLC profile	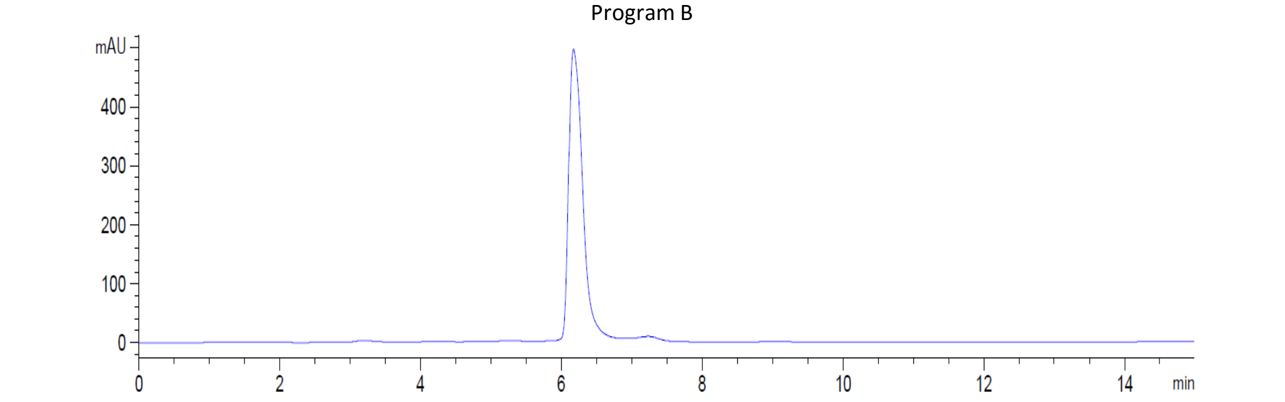
HRMS	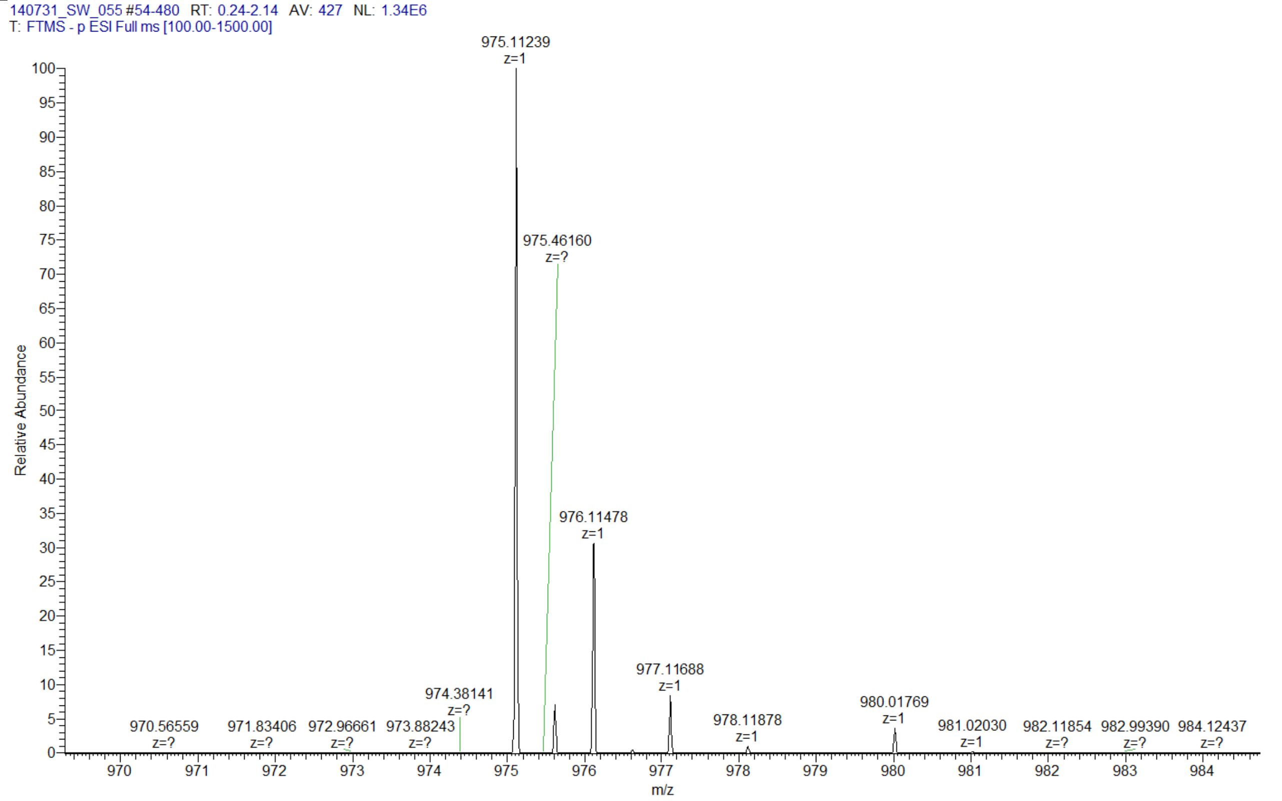

^{1}H NMR

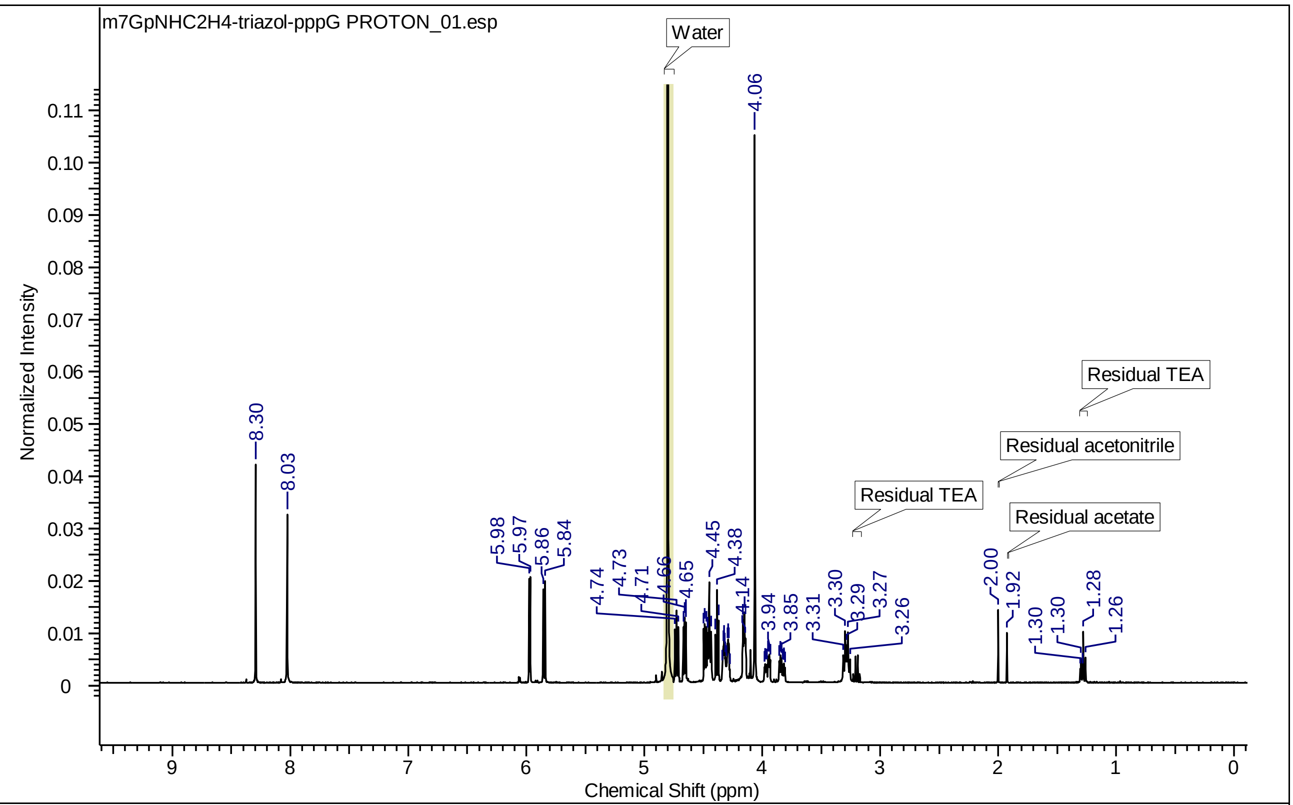

^{31}P NMR

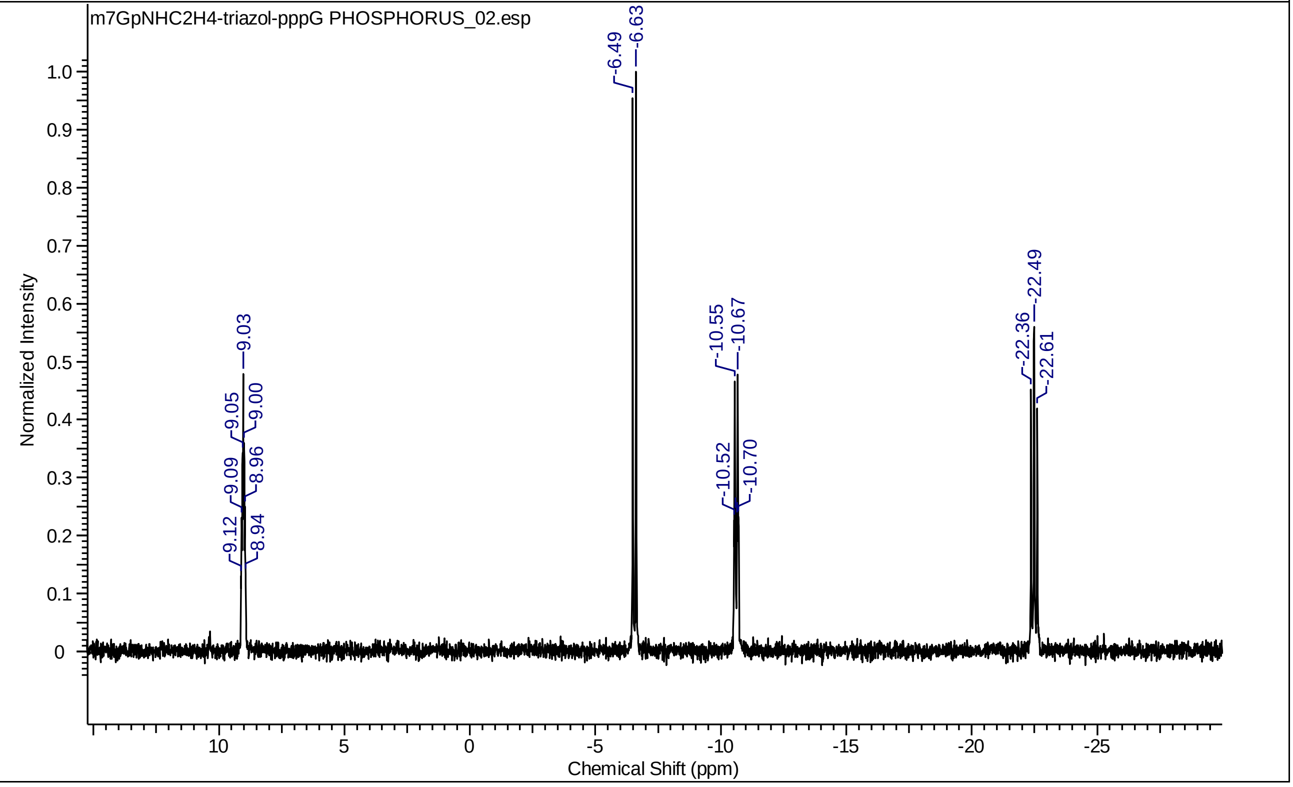

(8c) m^{7}Gpp-triazole-C_2H_4NHpG

Structure

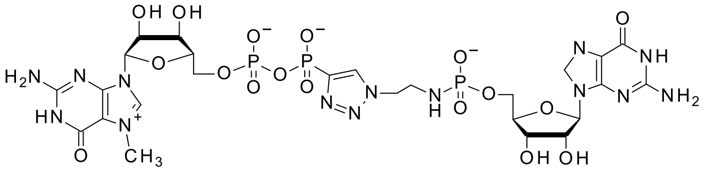

Reaction HPLC profile

Program B; before and after the reaction

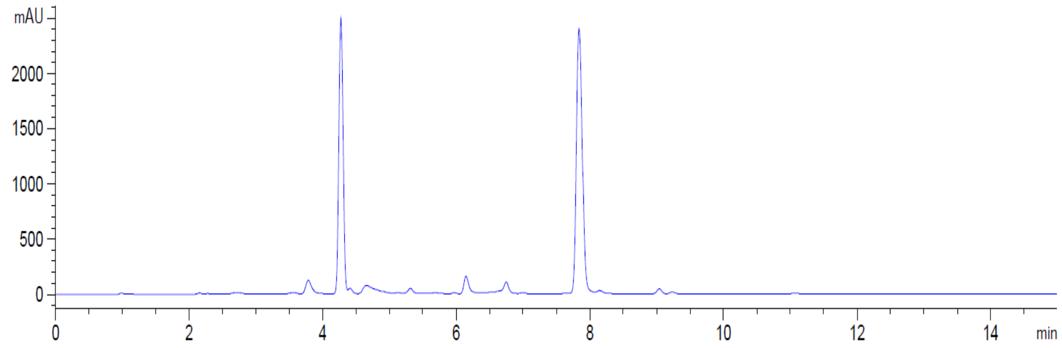

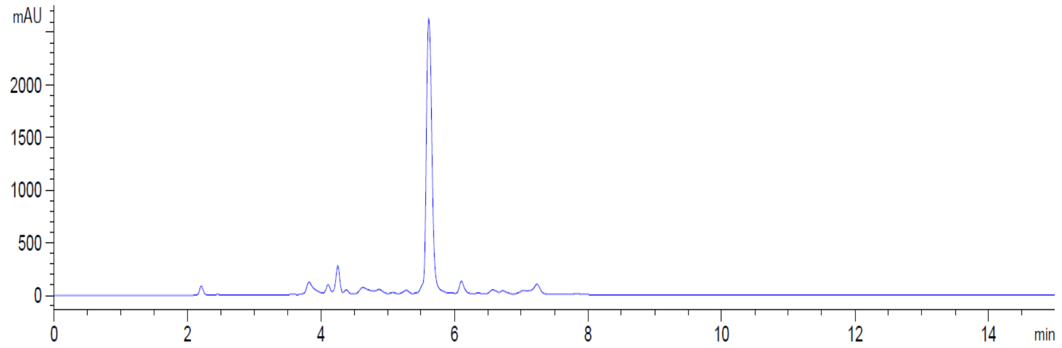

Purified compound HPLC profile

Program B

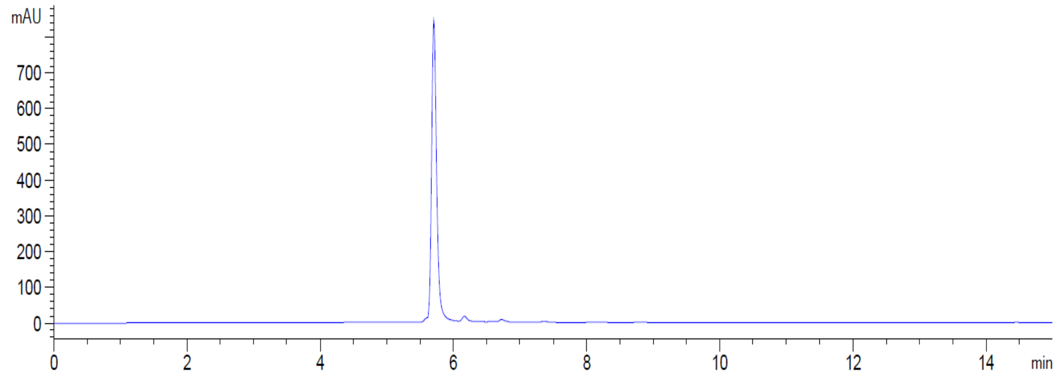

HRMS

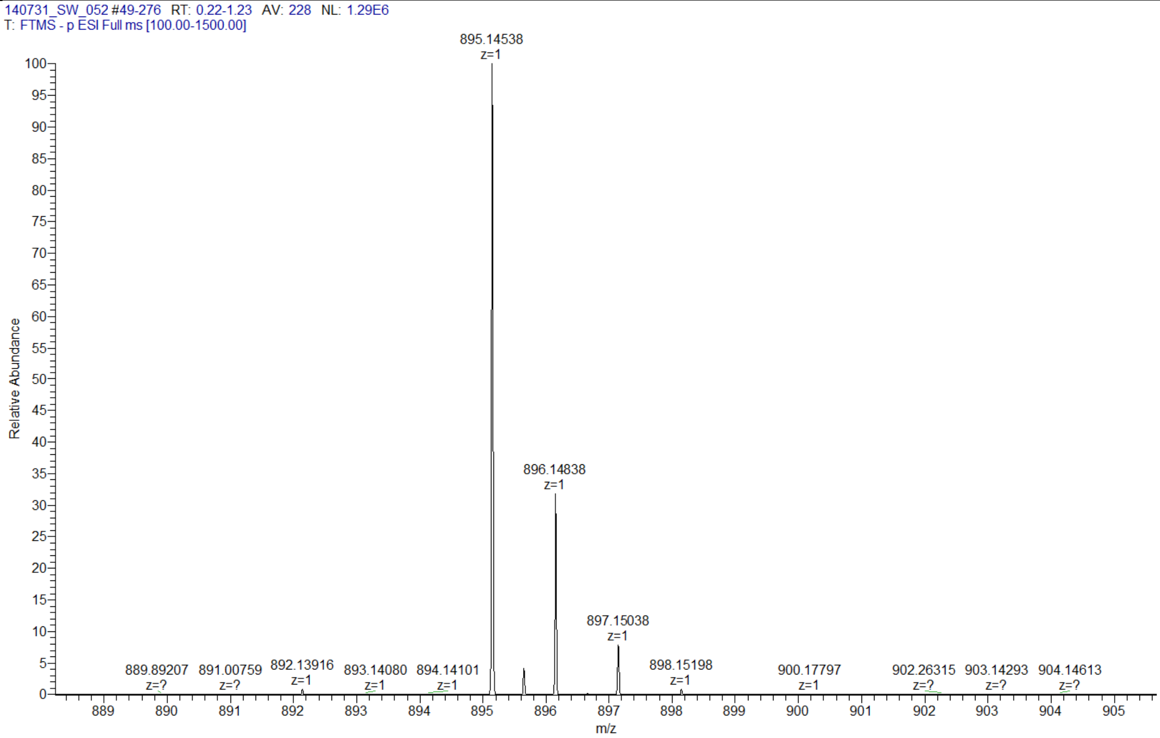

^{1}H NMR	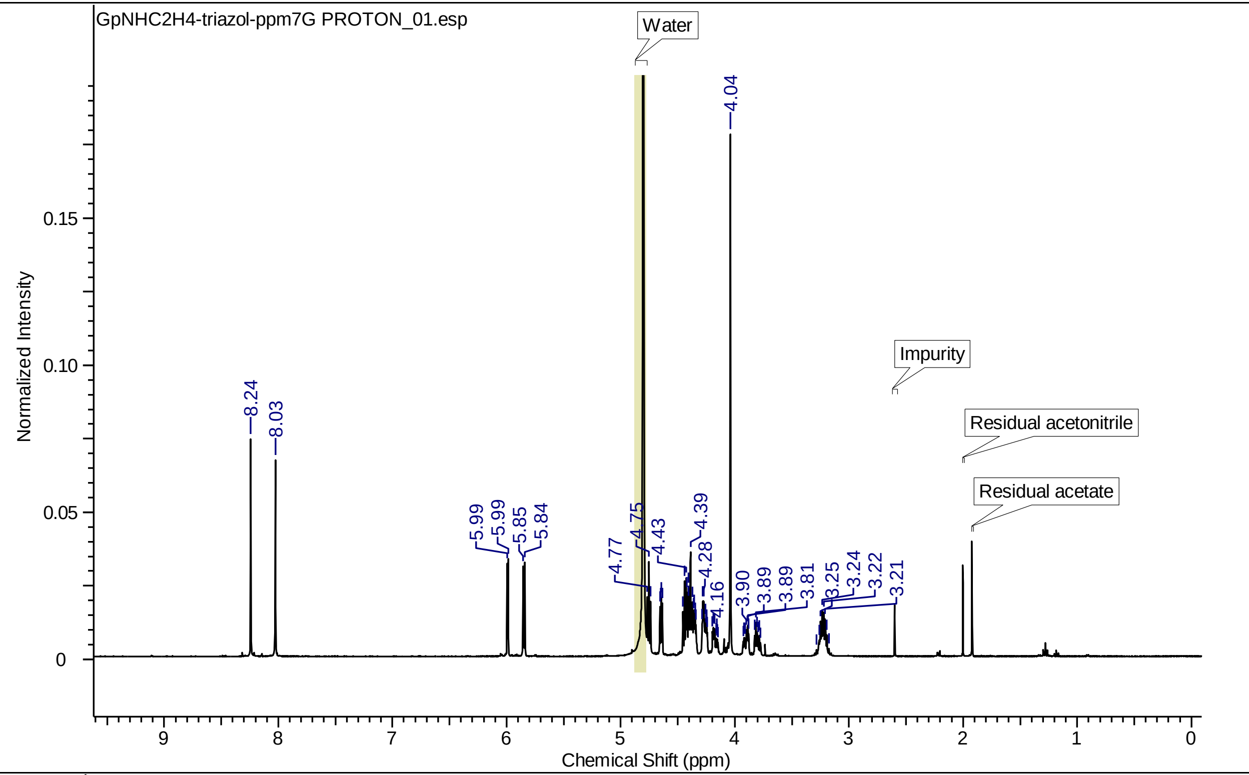
^{31}P NMR	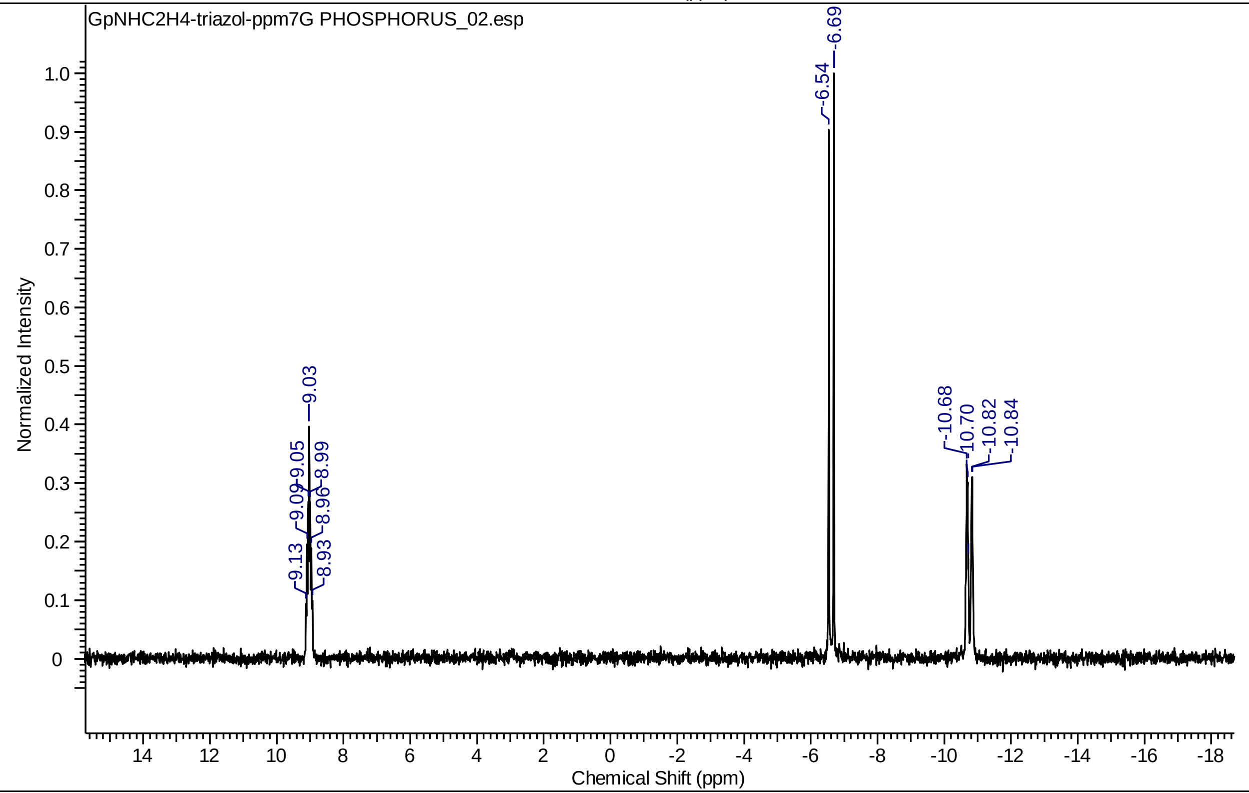

(8d) m^{7}Gppp-triazole-C_2H_4NHpG

Structure	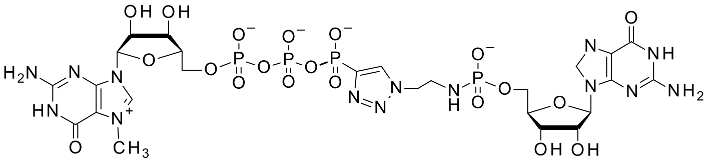

Reaction HPLC profile

Program B; before and after the reaction

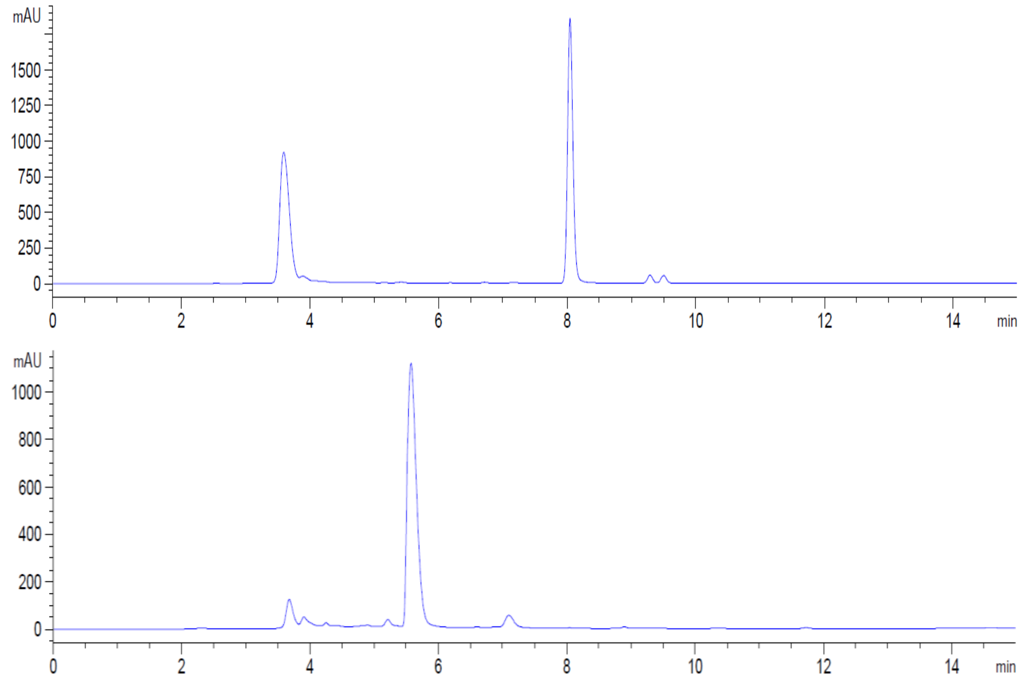

Purified compound HPLC profile

Program B

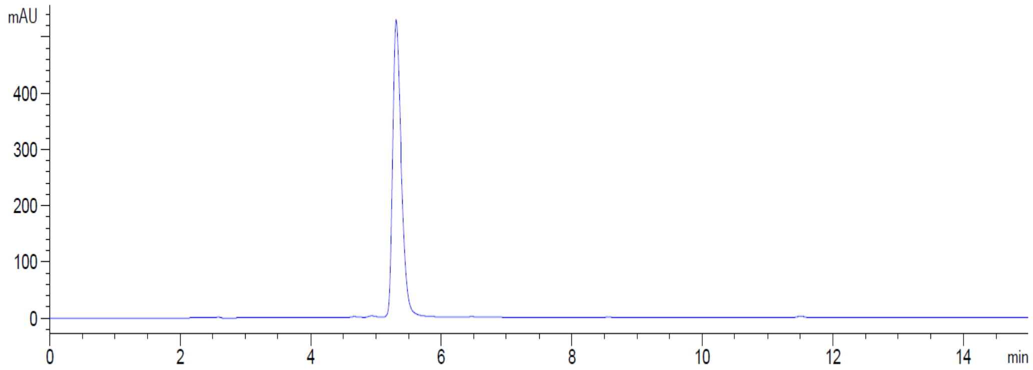

HRMS

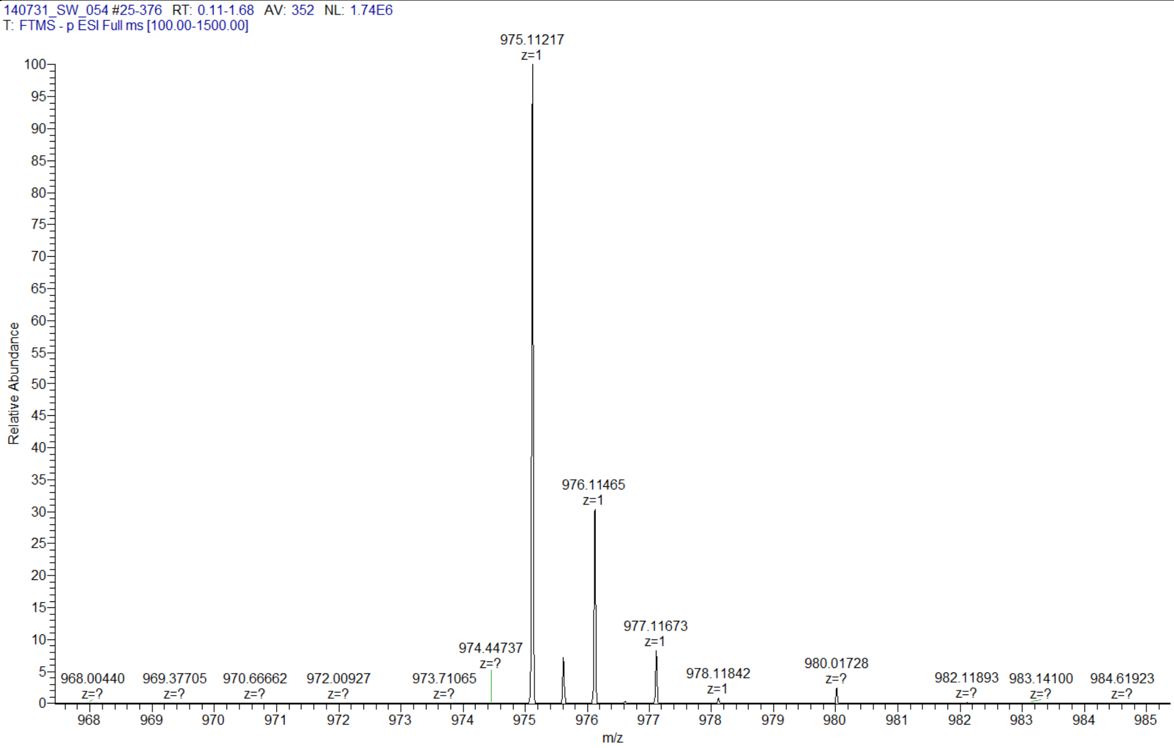

^{1}H NMR

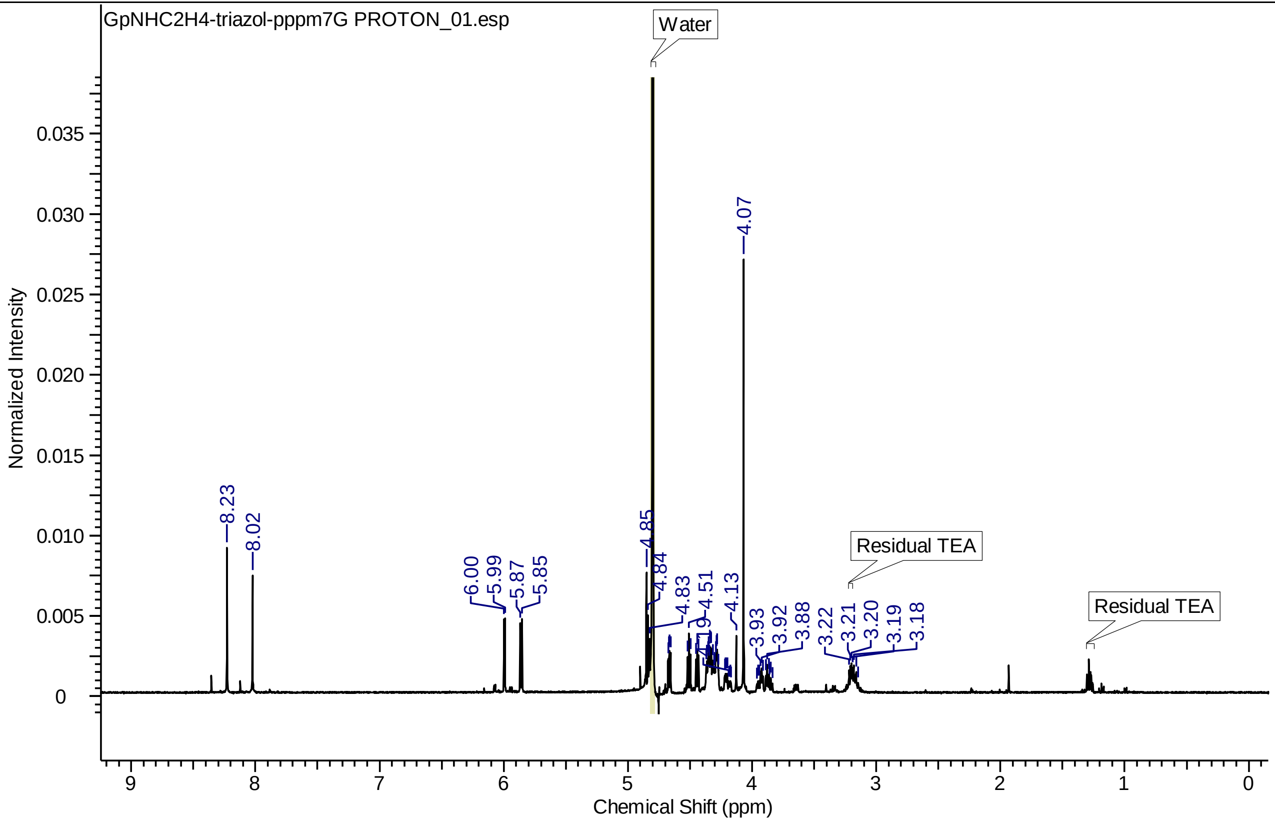

^{31}P NMR

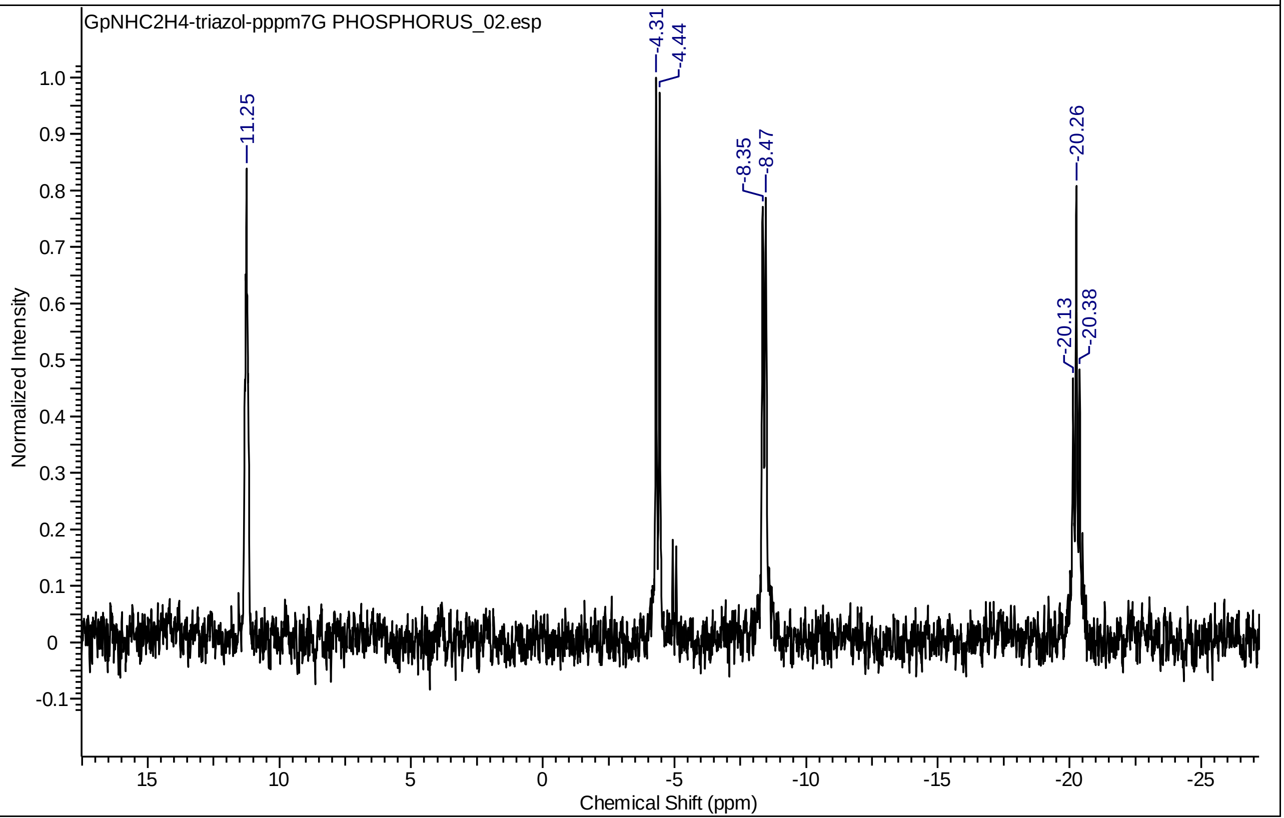

(8e) $m_2^{7,2'-O}$Gppp-triazole-C_2H_4NHpG

Structure

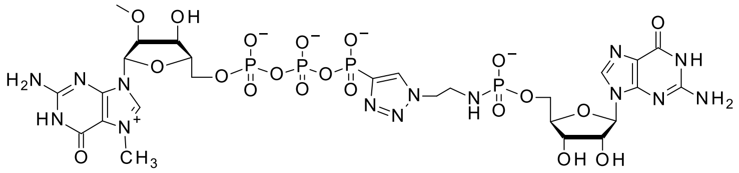

**Reaction HPLC profile**	Program B; before and after the reaction 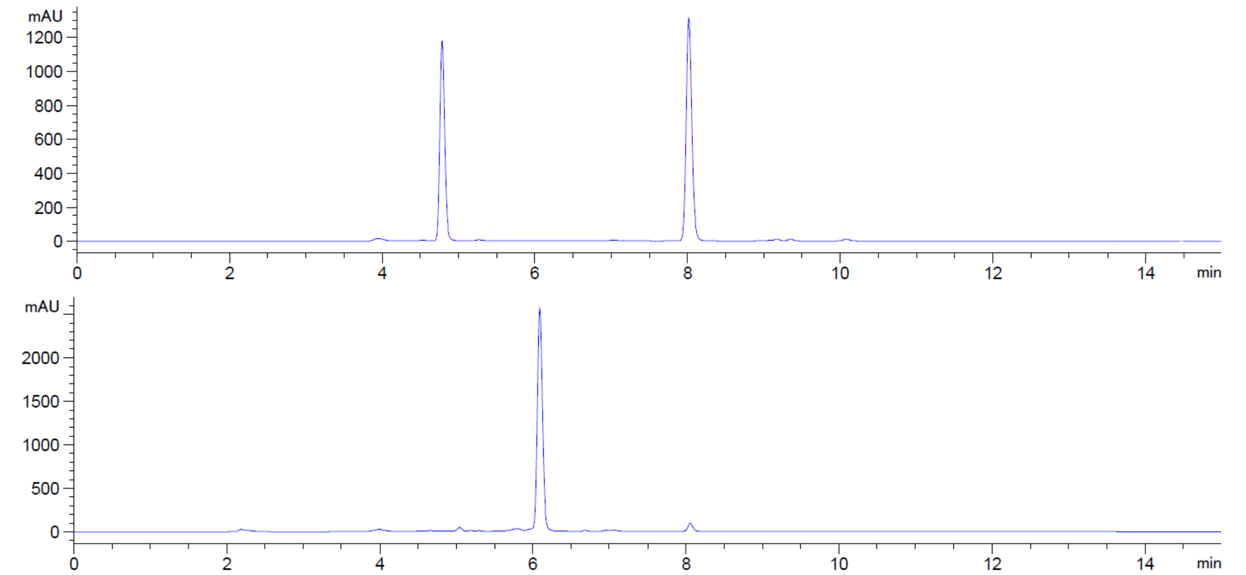
**Purified compound HPLC profile**	Program B 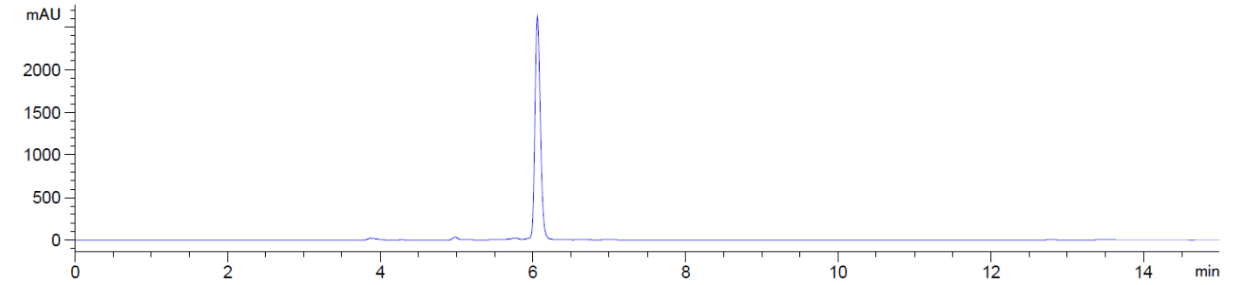
**HRMS**	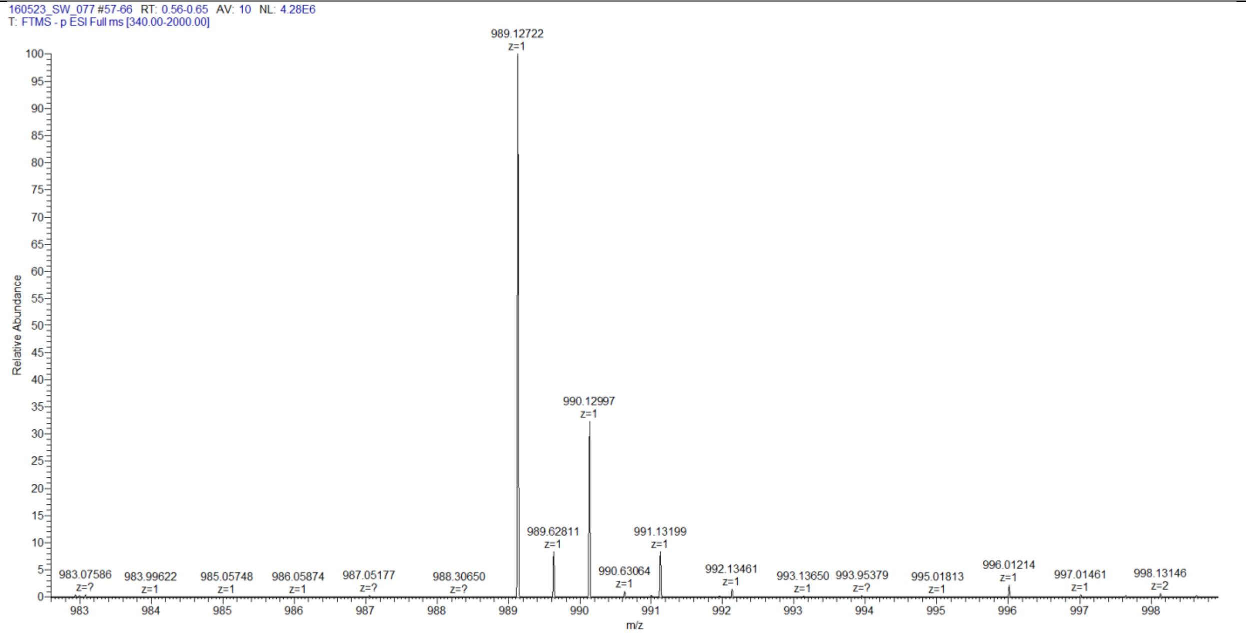

^{1}H NMR	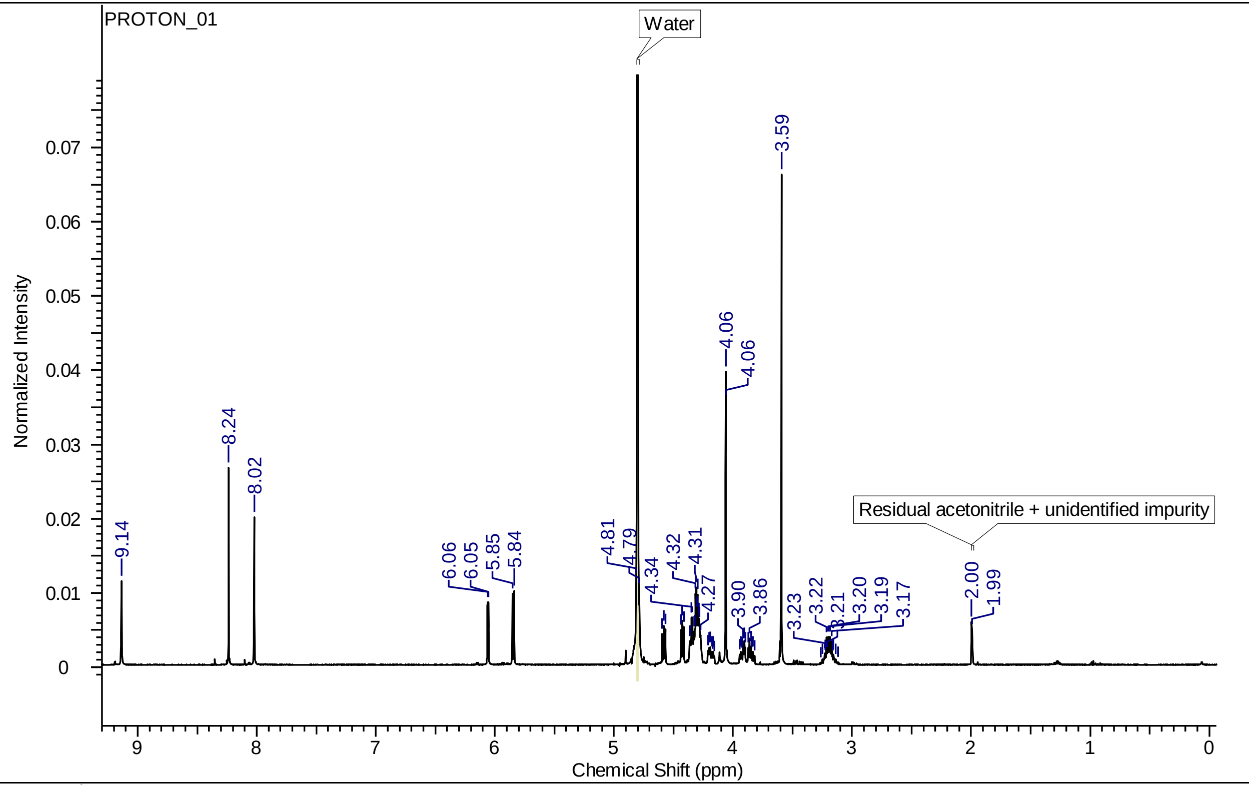
^{31}P NMR	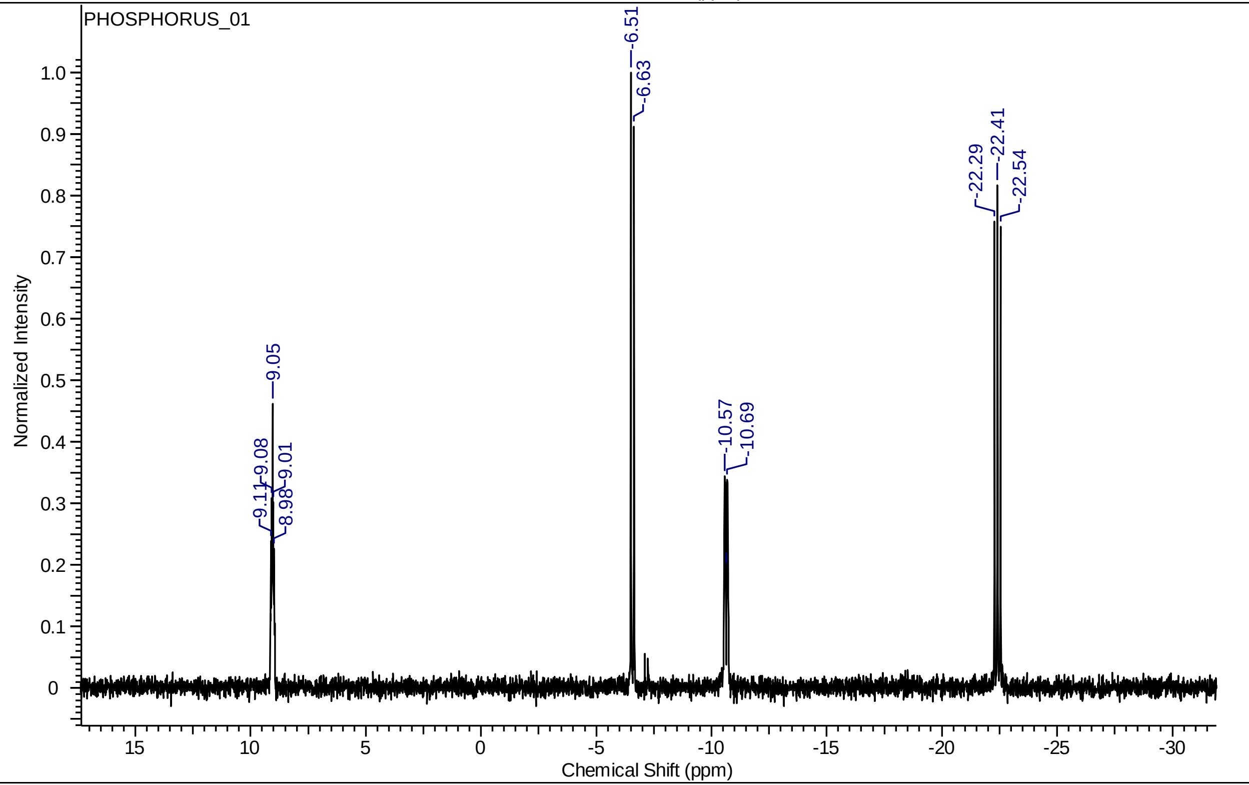

(9a) m^{7}GppNHC$_2$H$_4$-triazole-C$_2$H$_4$ppG

Structure	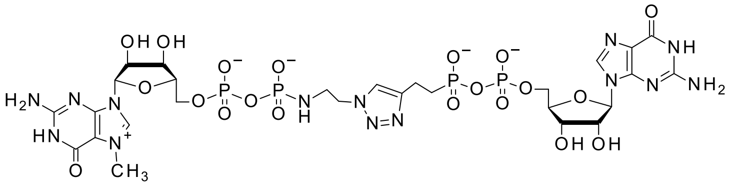

**Reaction HPLC profile**	Program A; before and after the reaction<br>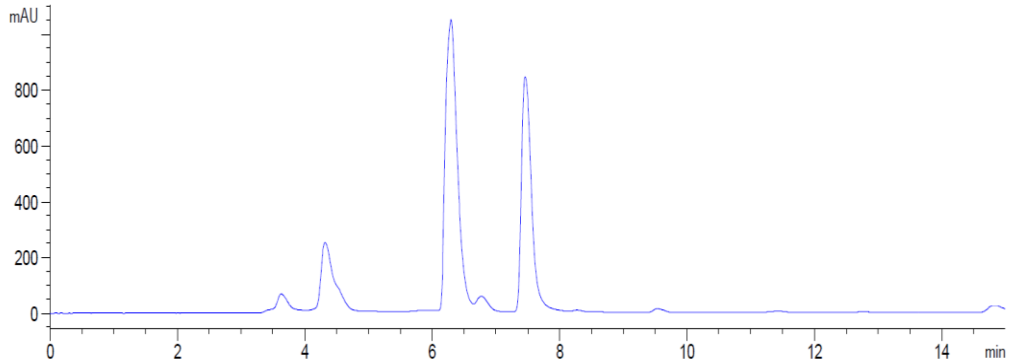<br><br>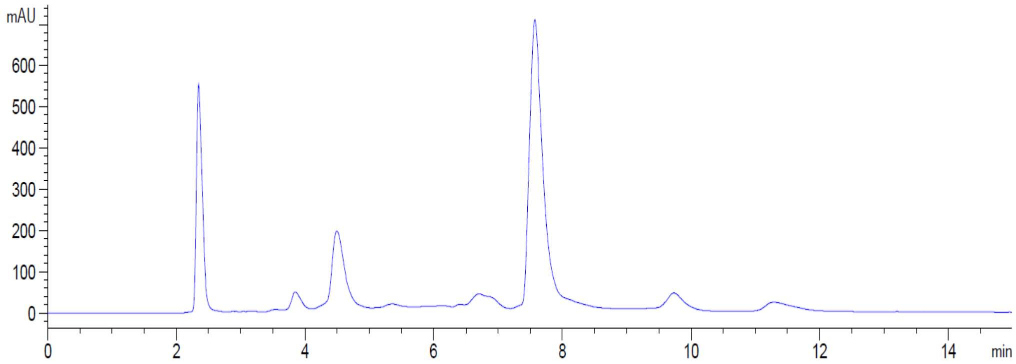
**Purified compound HPLC profile**	Program B<br>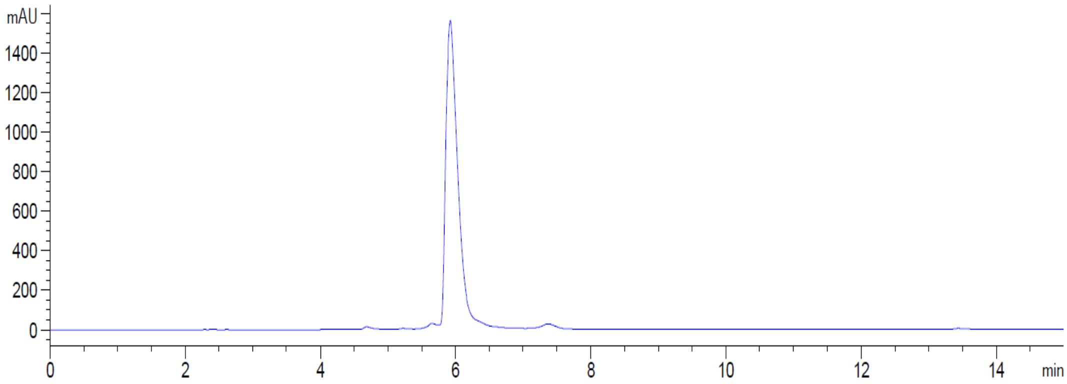
**HRMS**	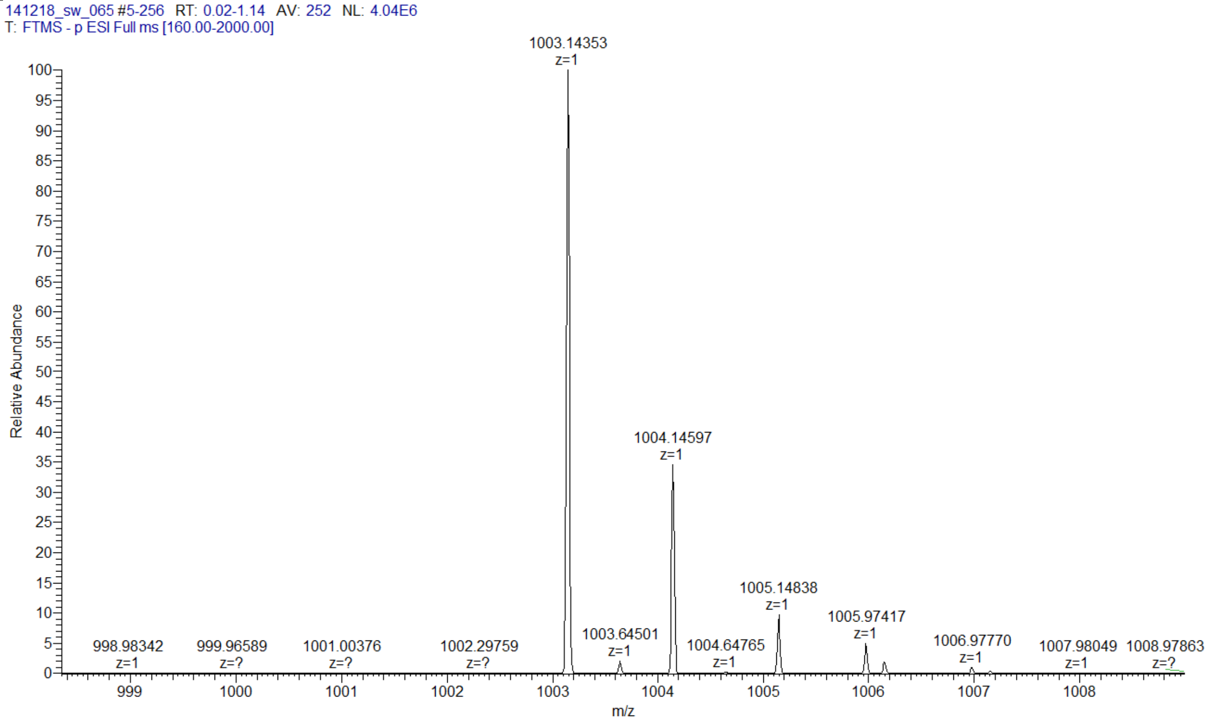

^{1}H NMR

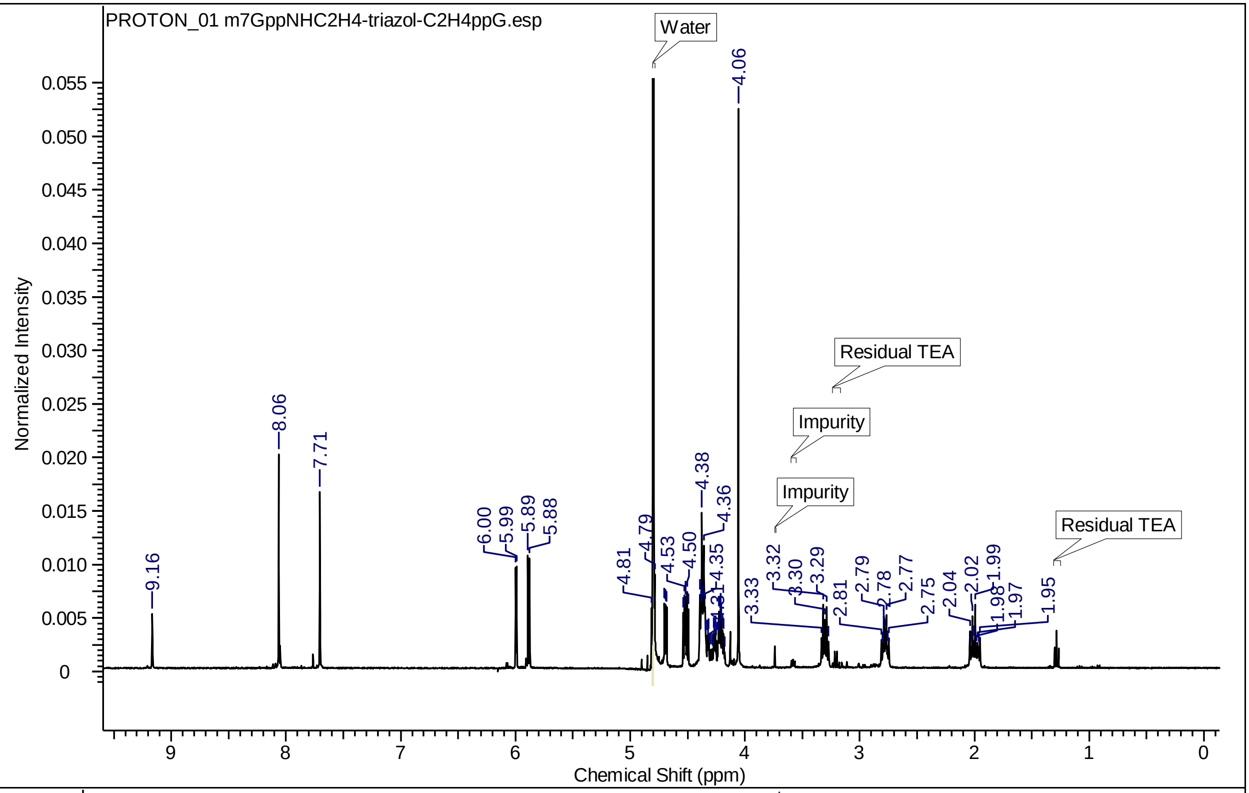

^{31}P NMR

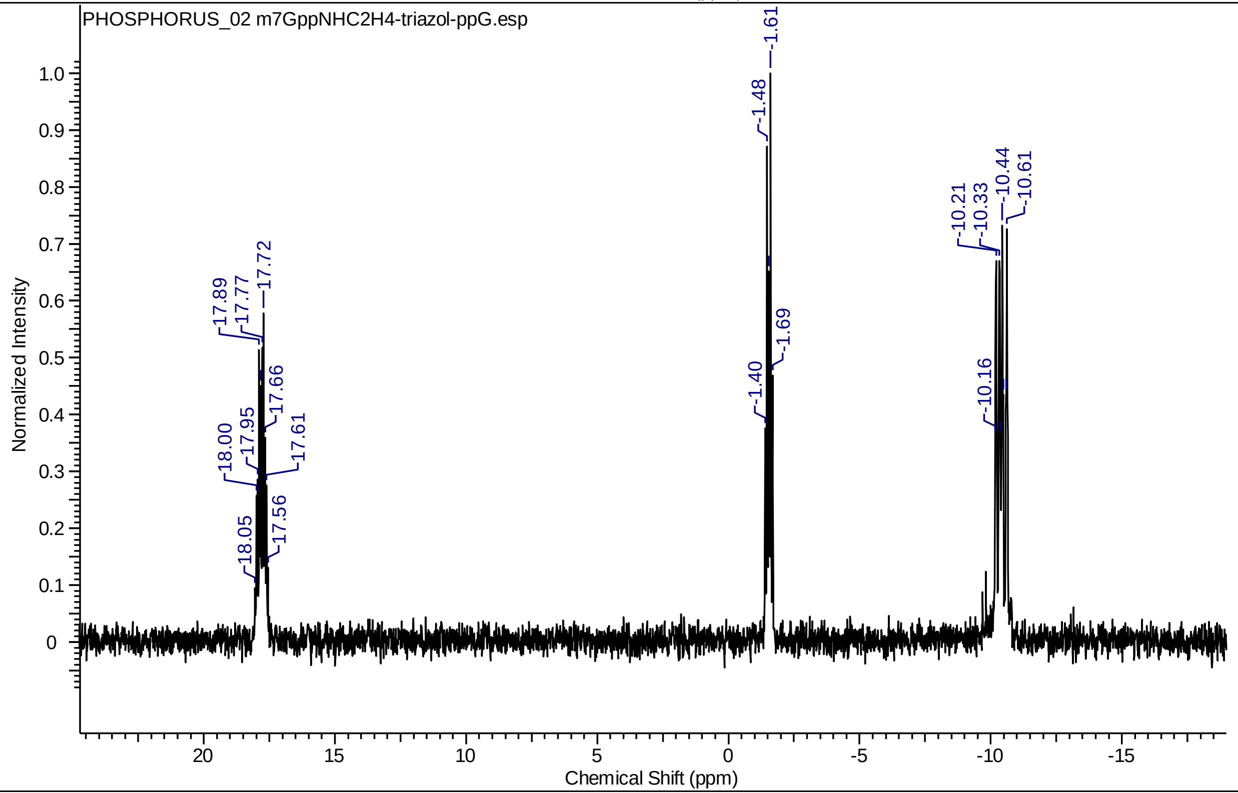

(9b) m^{7}GppC$_2$H$_4$-triazole-C$_2$H$_4$NHppG

Structure

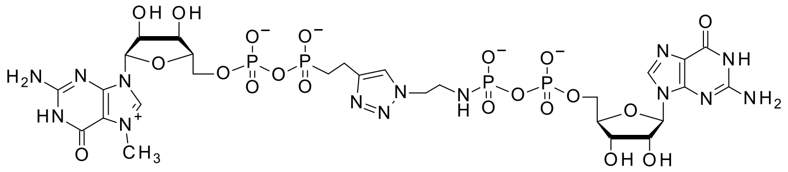

Reaction HPLC profile

Program A; before and after the reaction

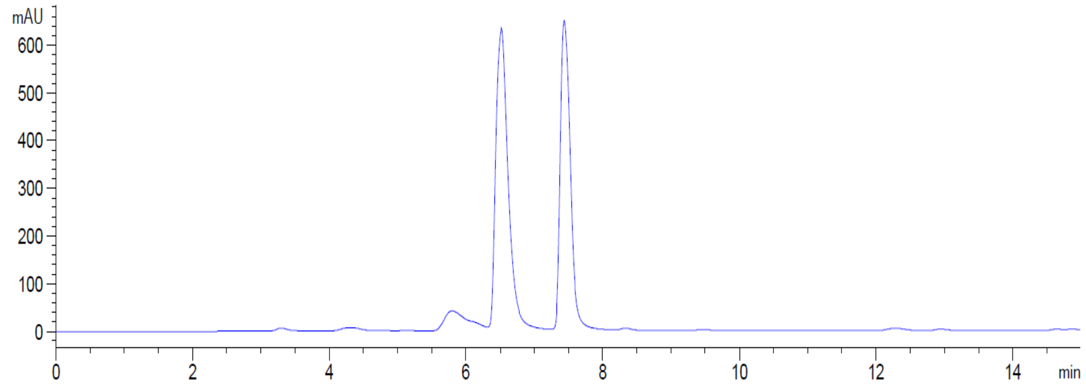

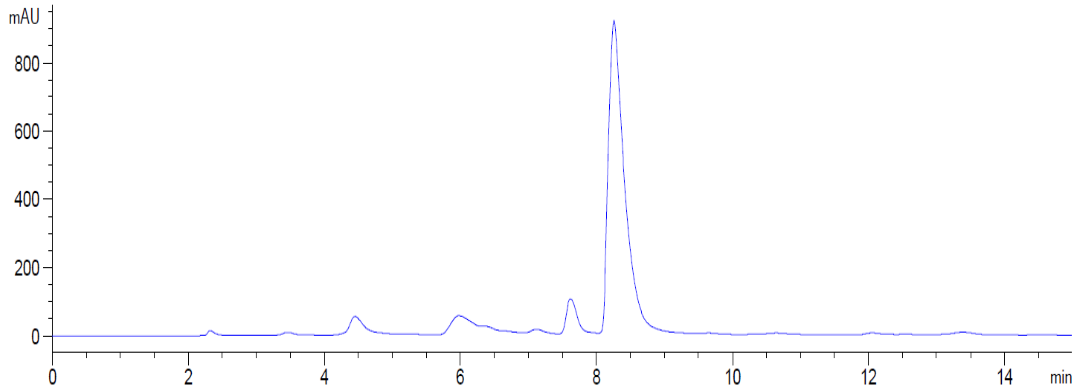

Purified compound HPLC profile

Program B

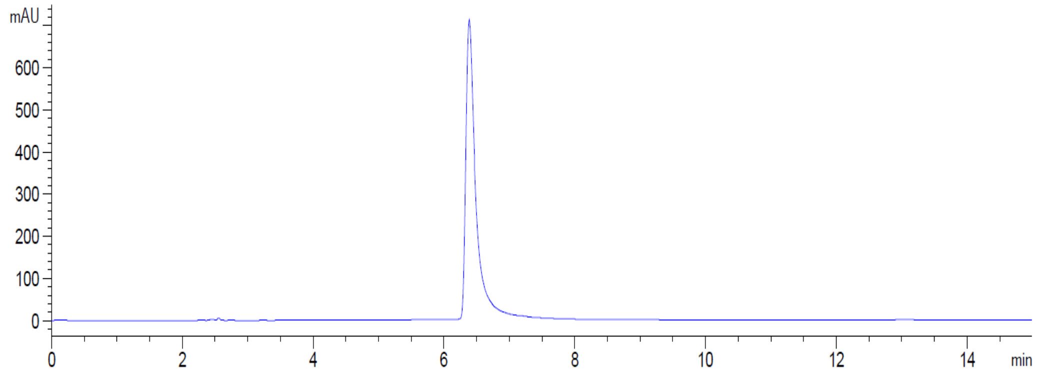

HRMS

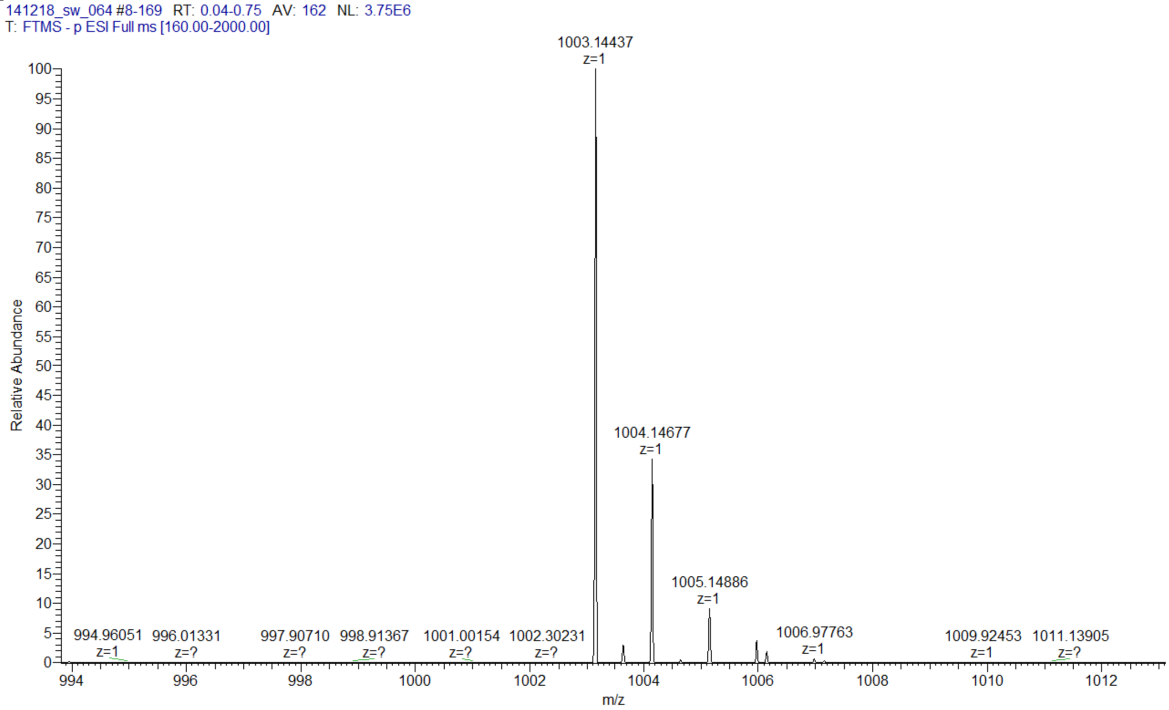

**^{1}H NMR**	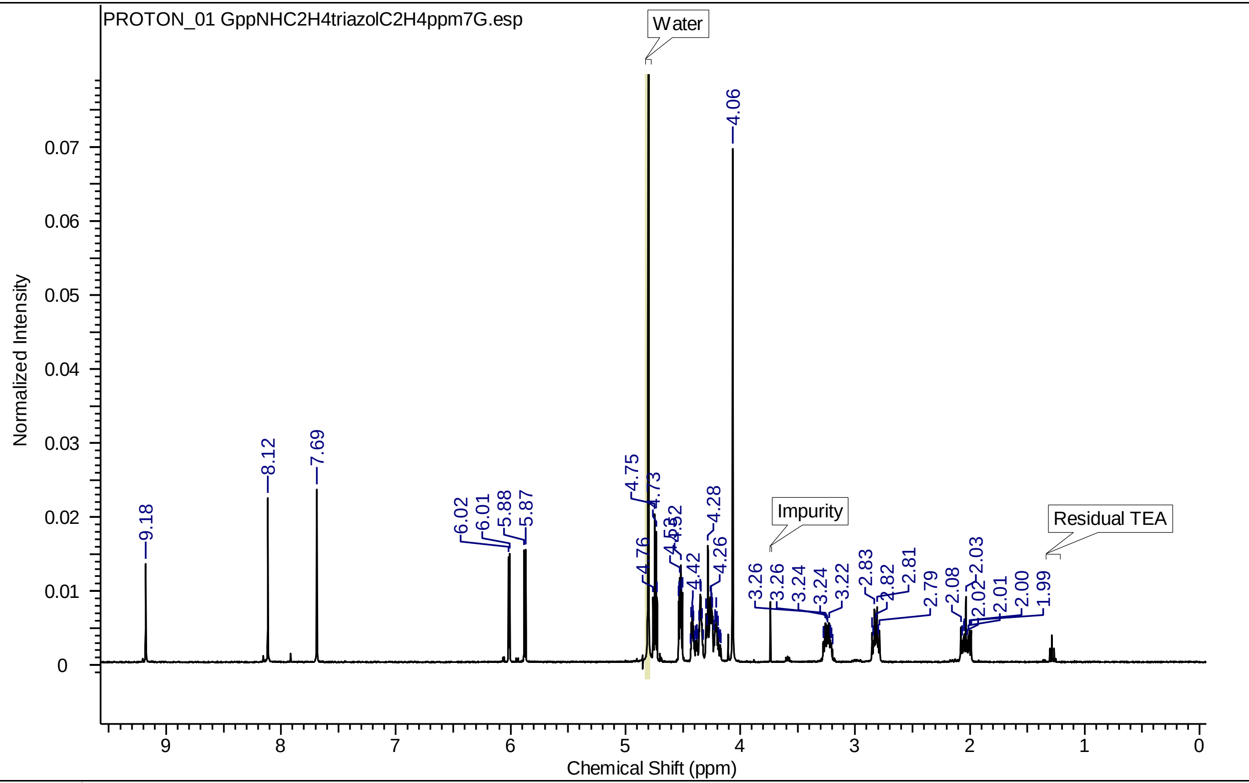
**^{31}P NMR**	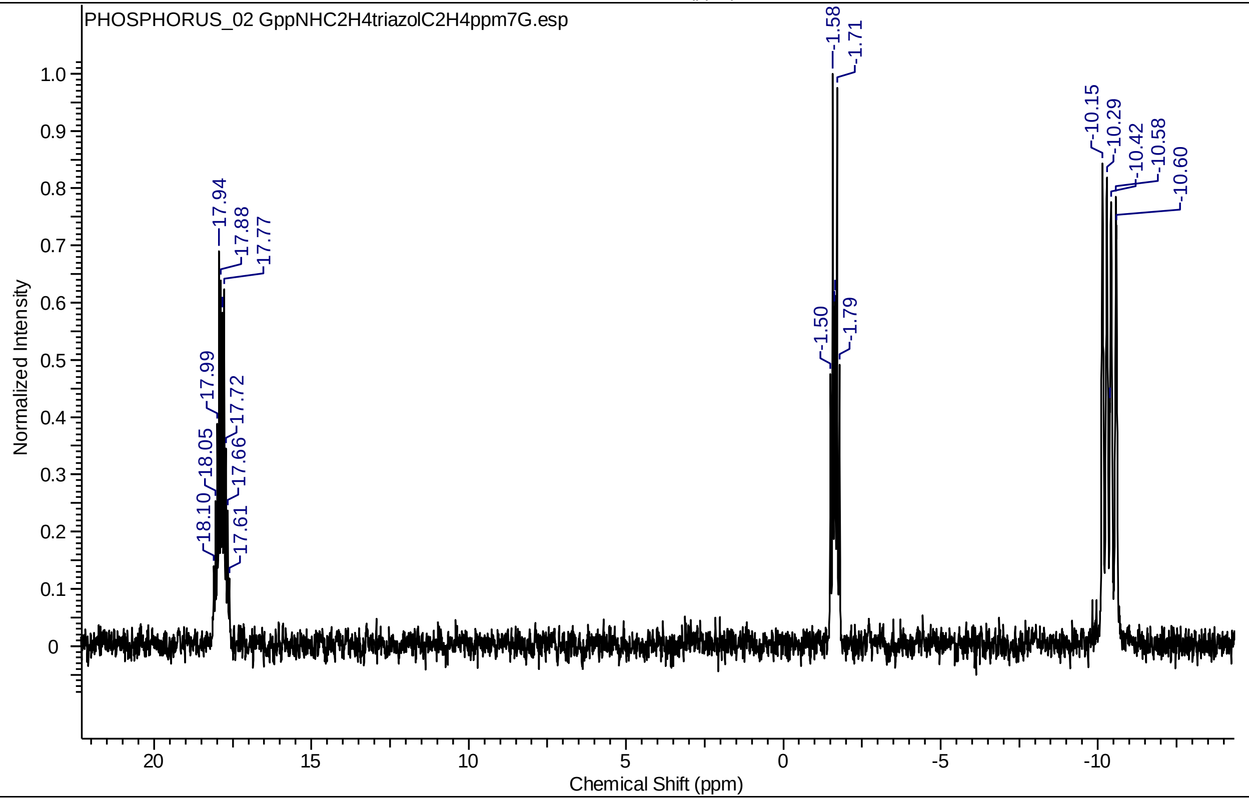

(10c) m^{7}GppC$_4$H$_5$

**Structure**	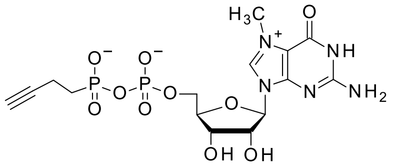

Reaction HPLC profile

Program A

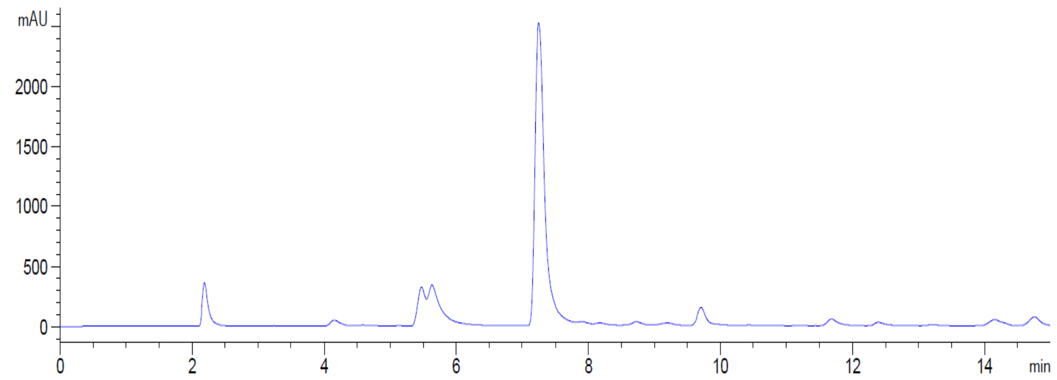

Purified compound HPLC profile

Program A

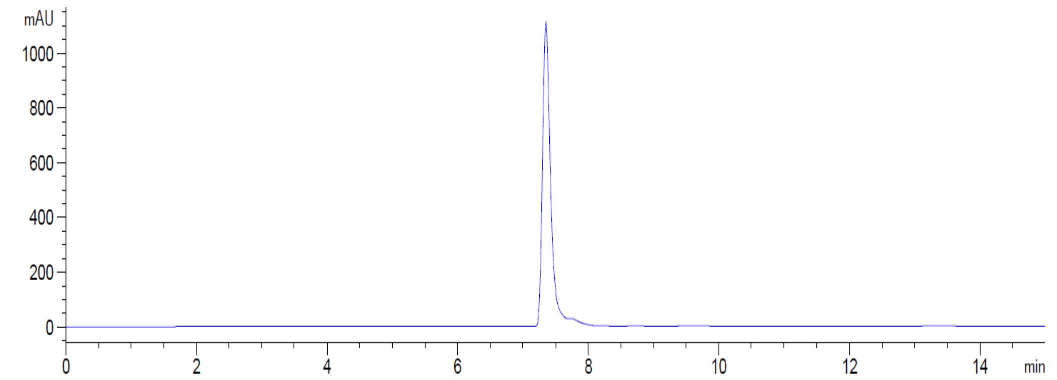

HRMS

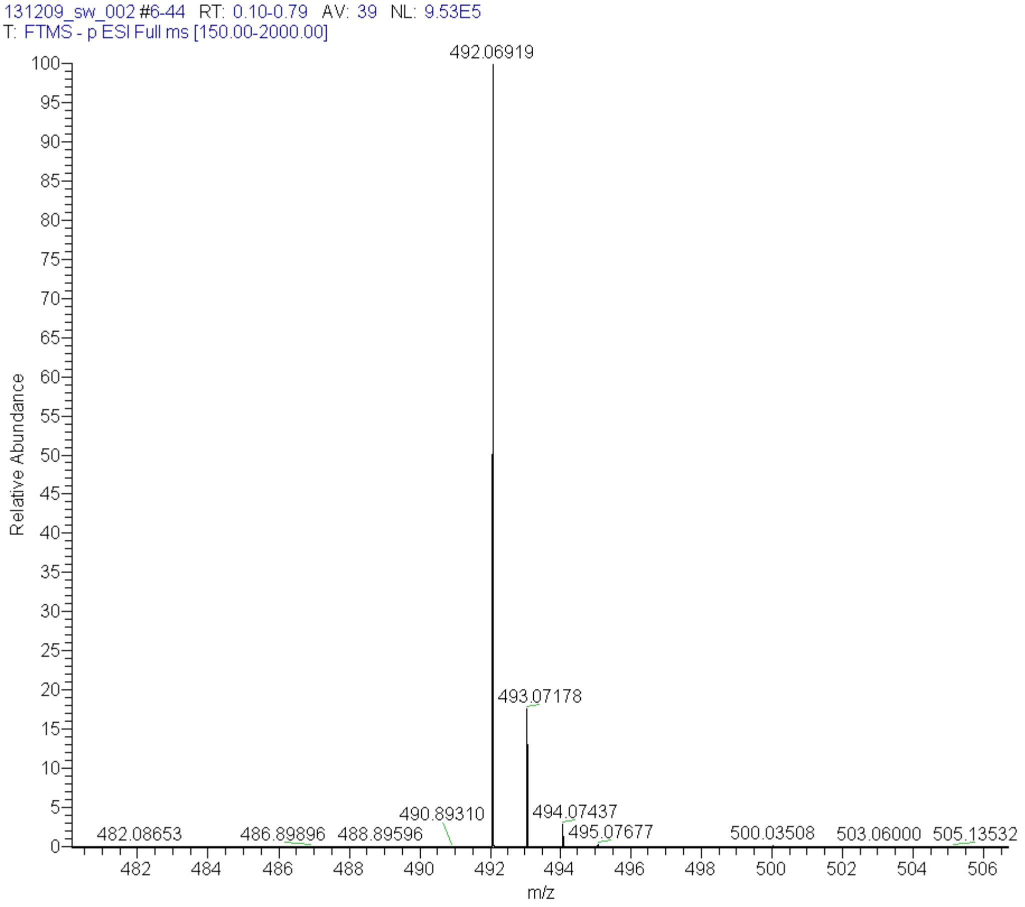

**^{1}H NMR**	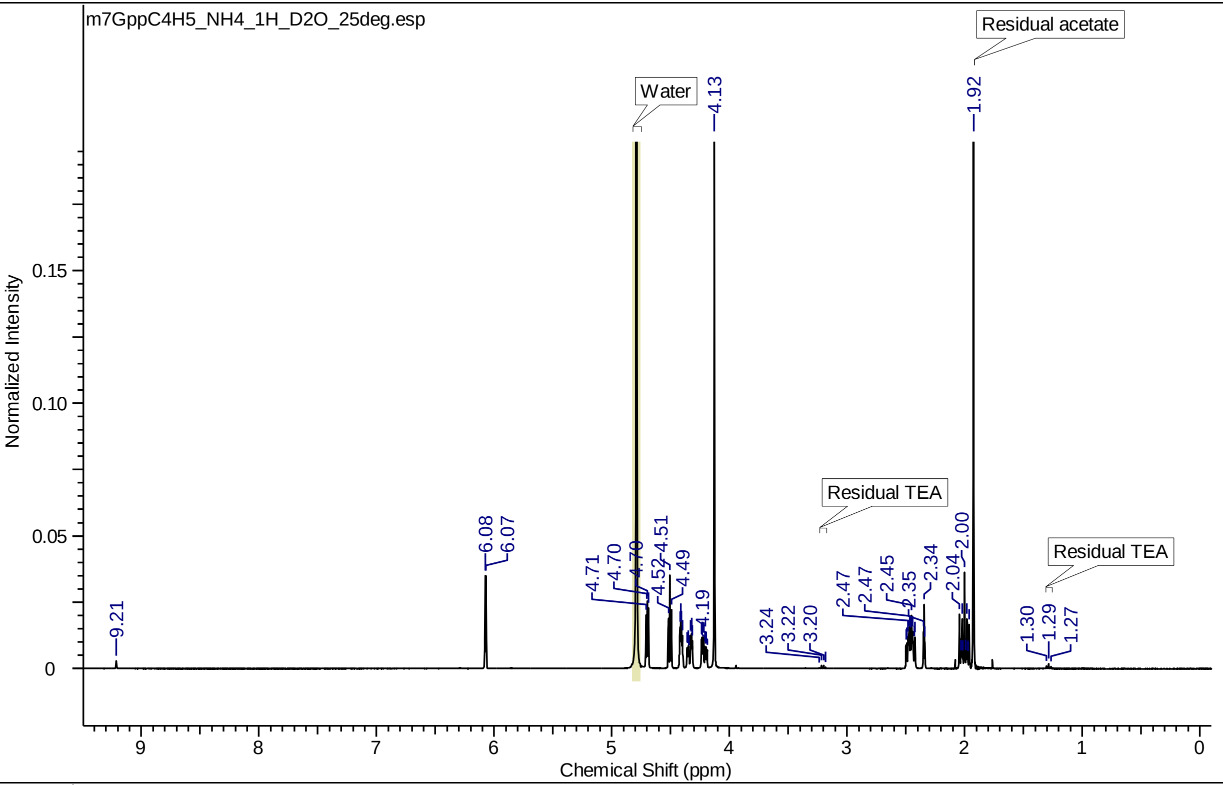
**^{31}P NMR**	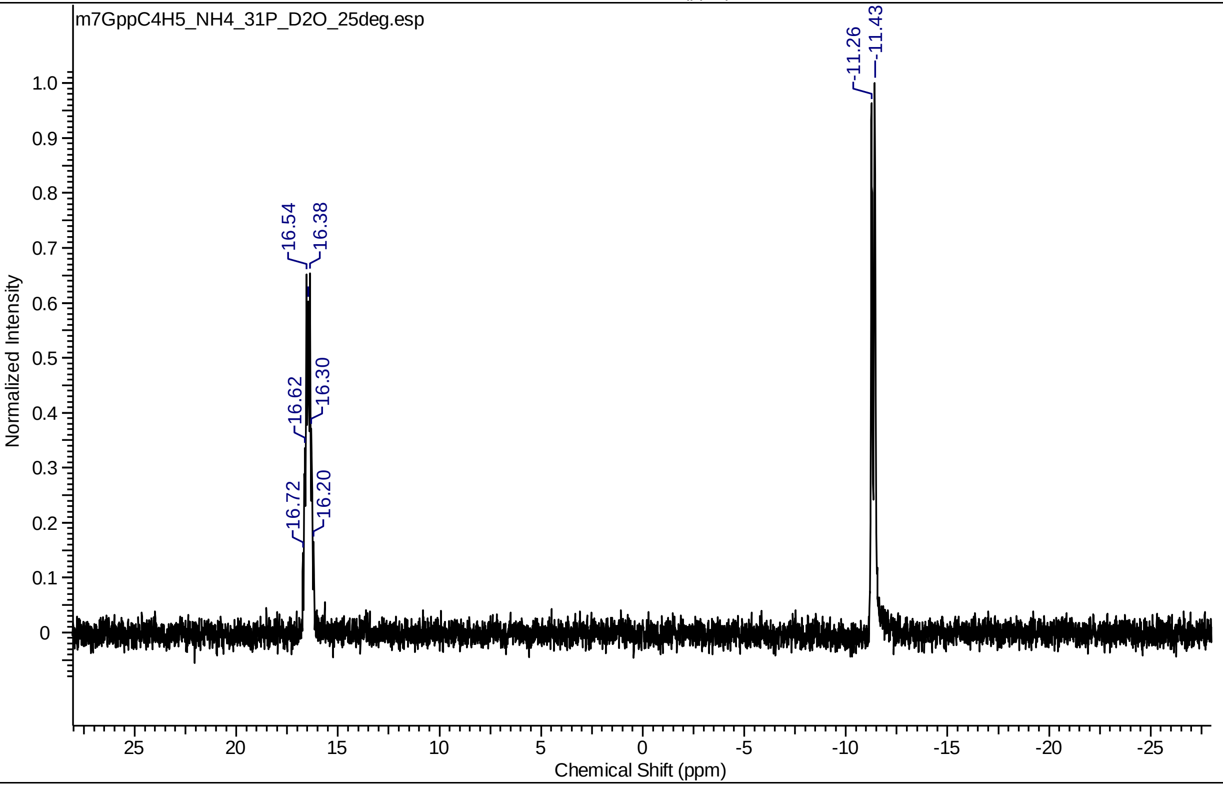

(10d) $m^7GpppC_4H_5$

**Structure**	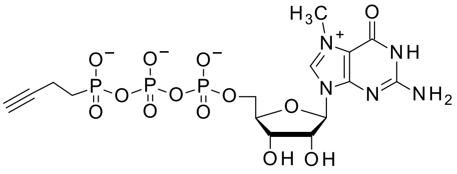

Reaction HPLC profile

Program A

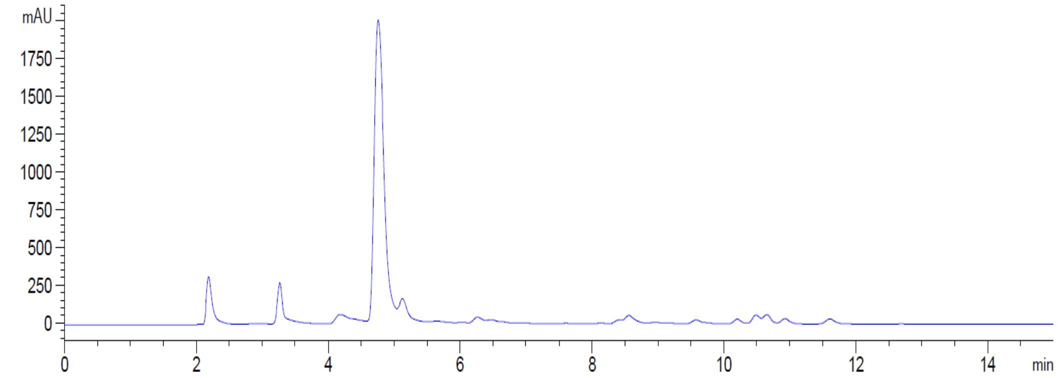

Purified compound HPLC profile

Program A

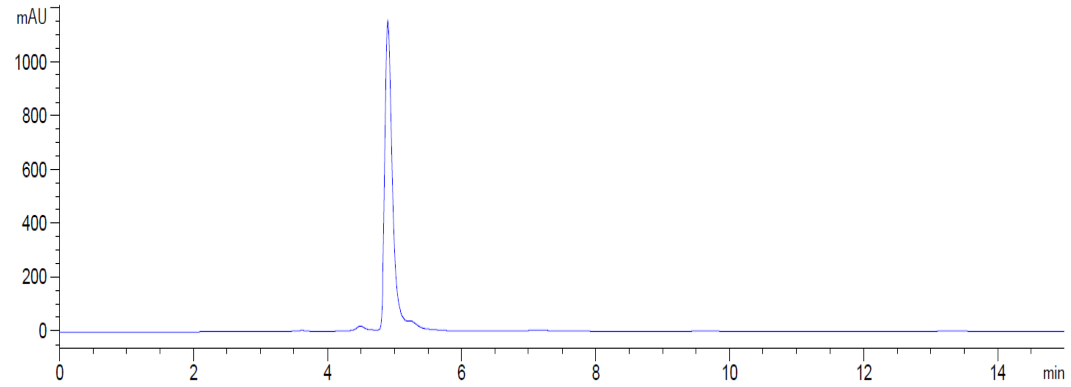

HRMS

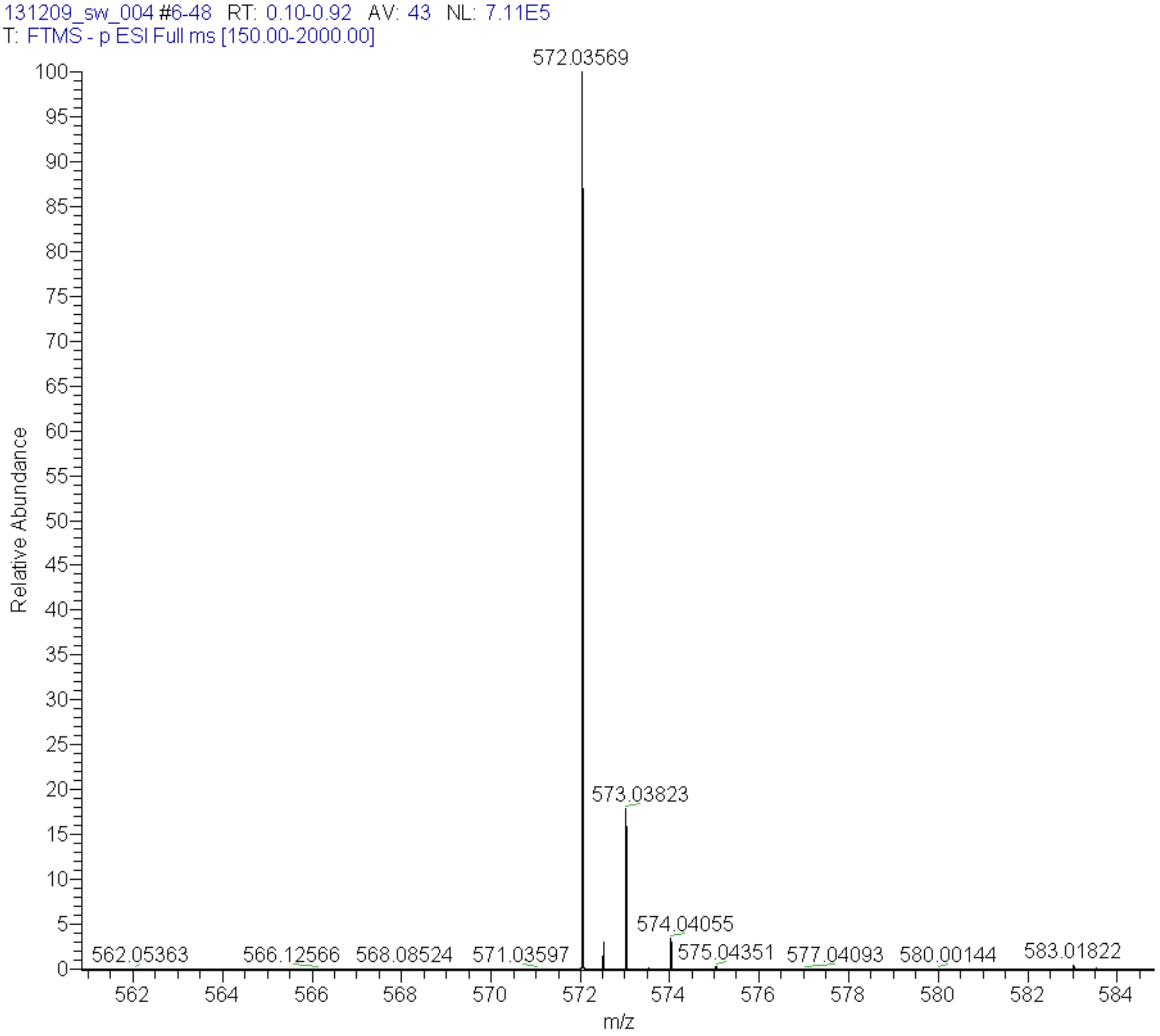

**^{1}H NMR**	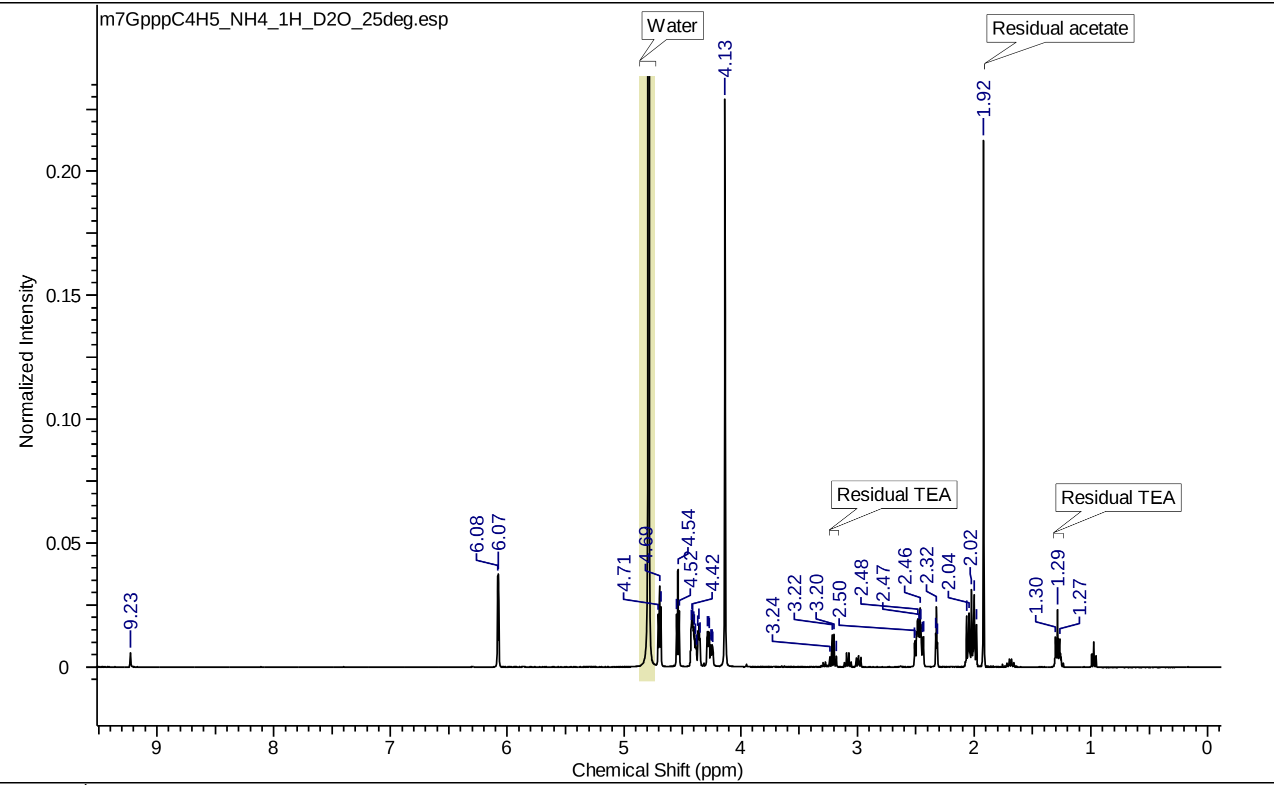
**^{31}P NMR**	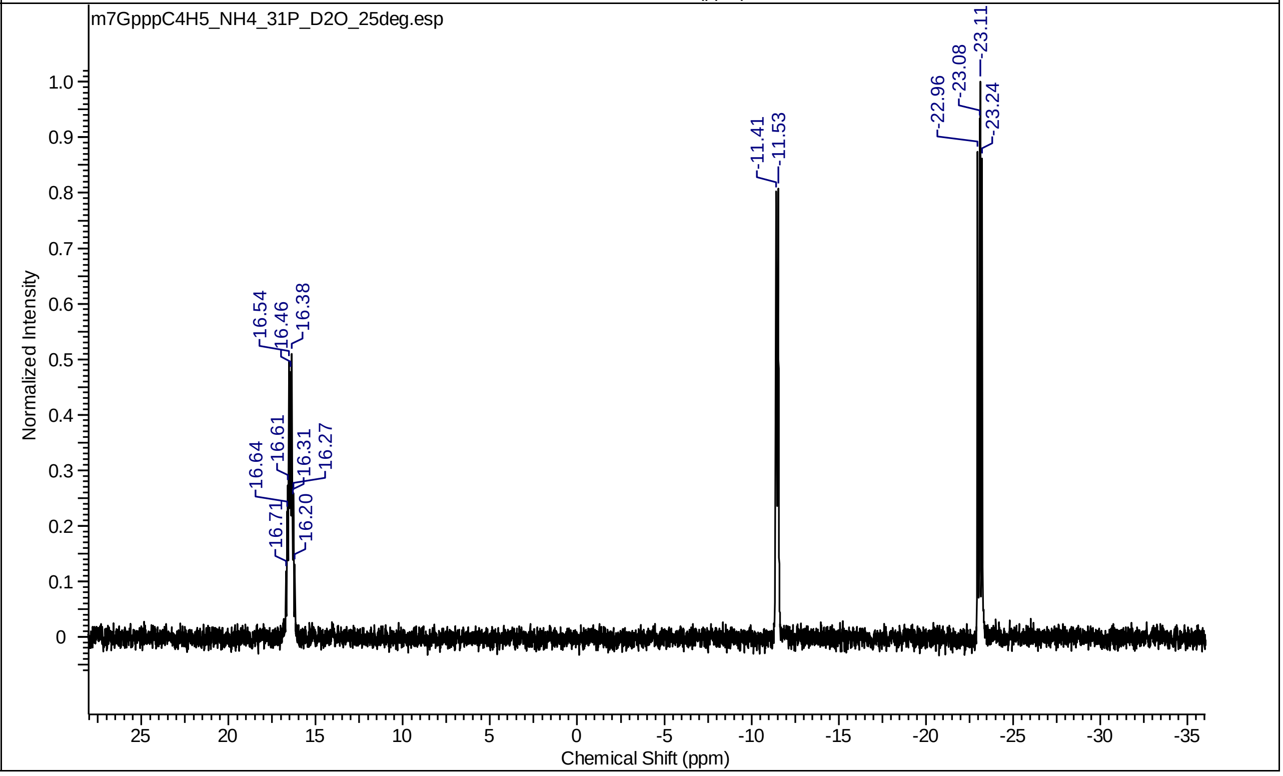

(11c) m^{7}GppC$_3$H$_3$

Structure	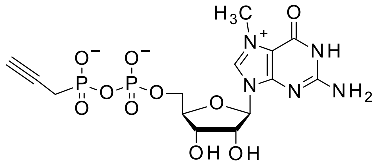

Reaction HPLC profile	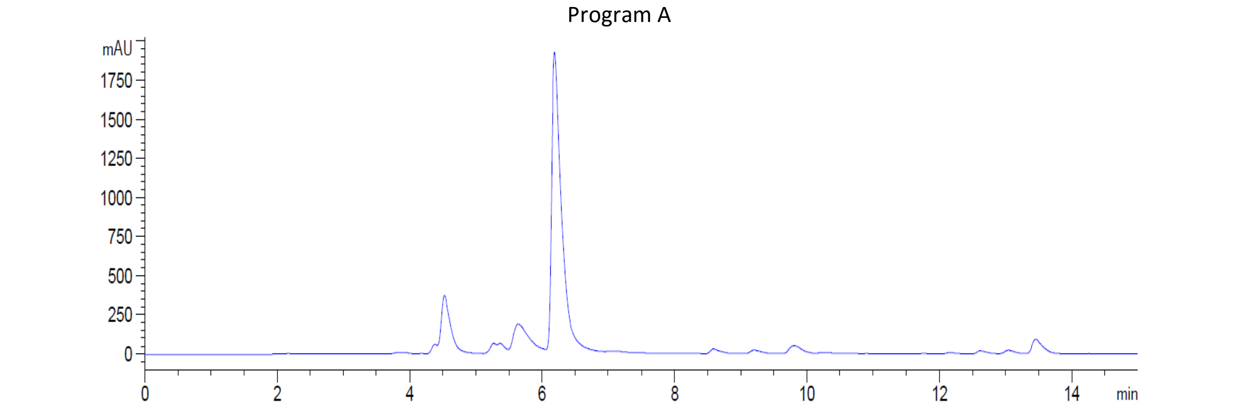
Purified compound HPLC profile	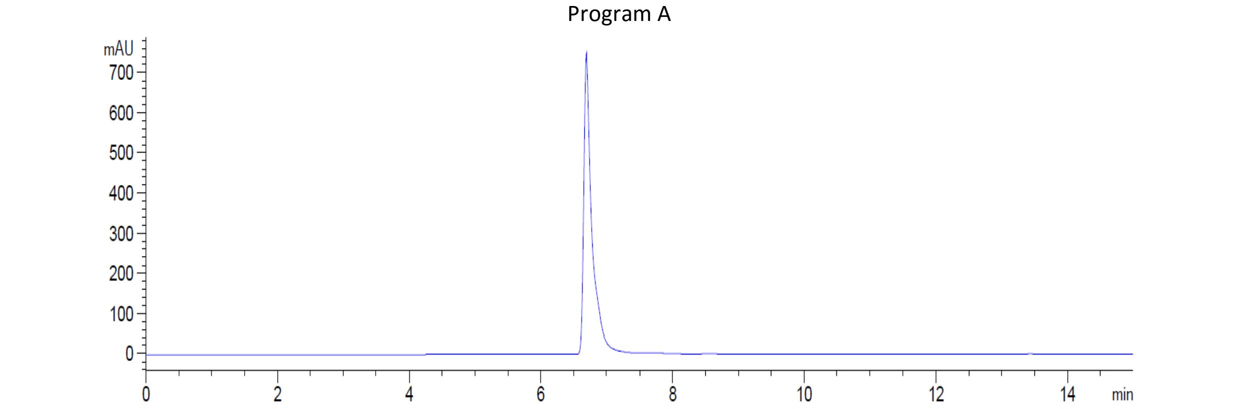
HRMS	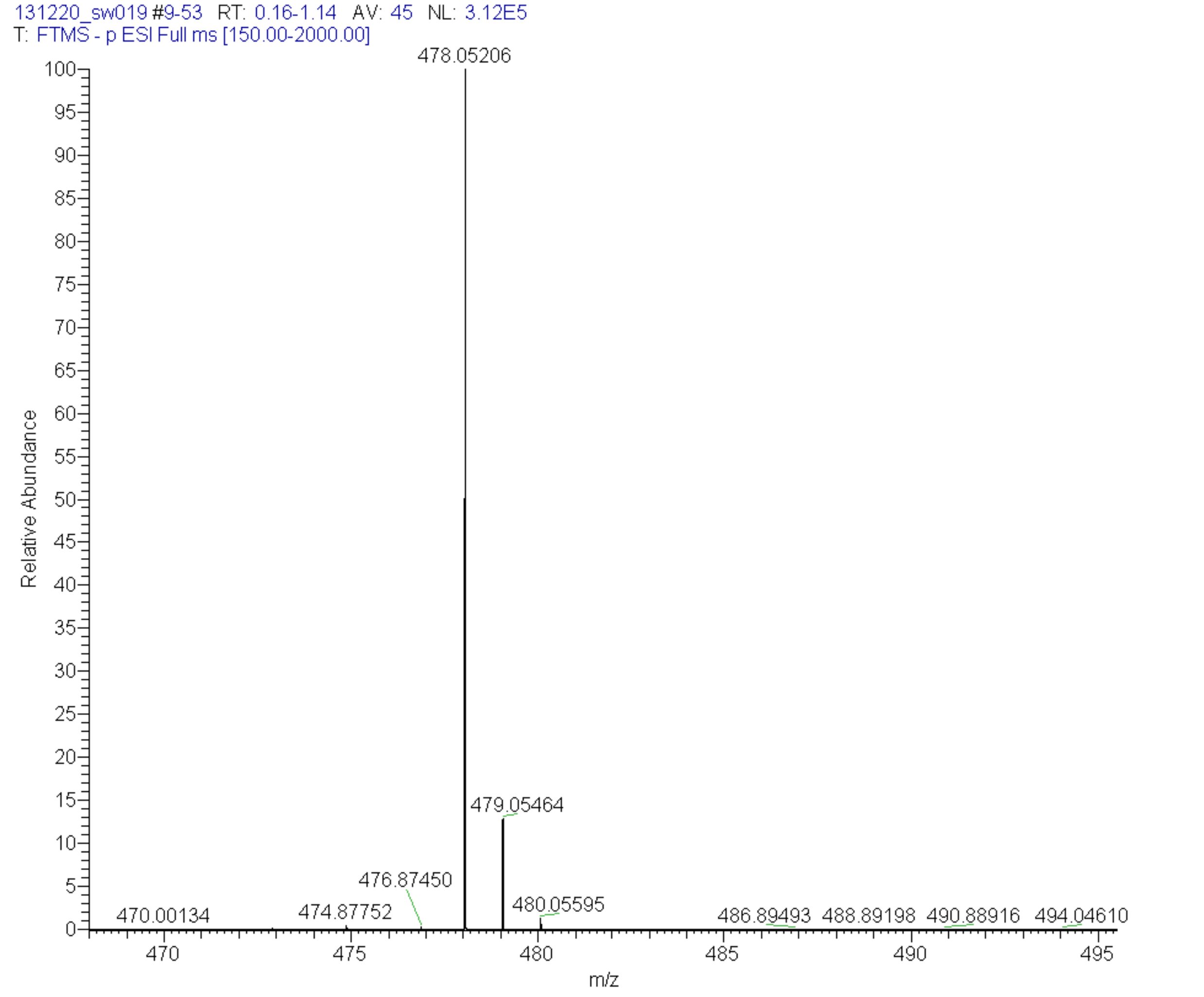

^{1}H NMR	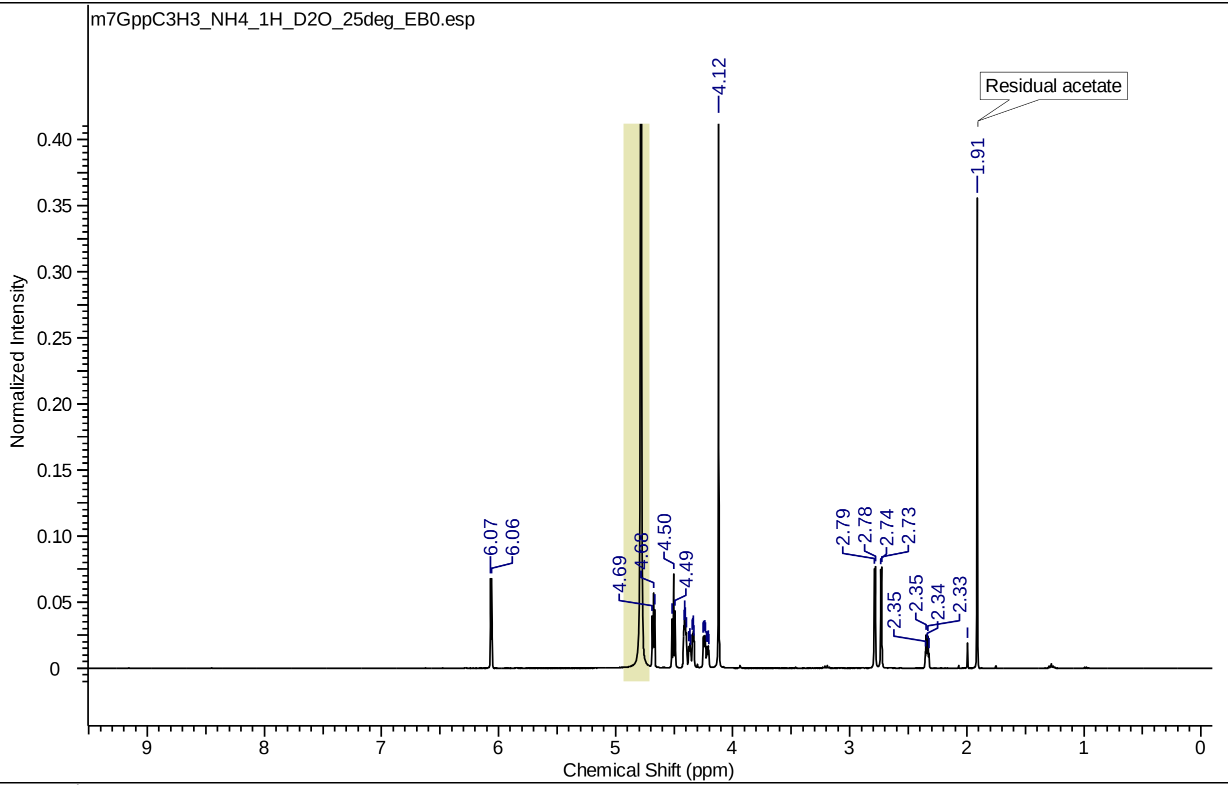
^{31}P NMR	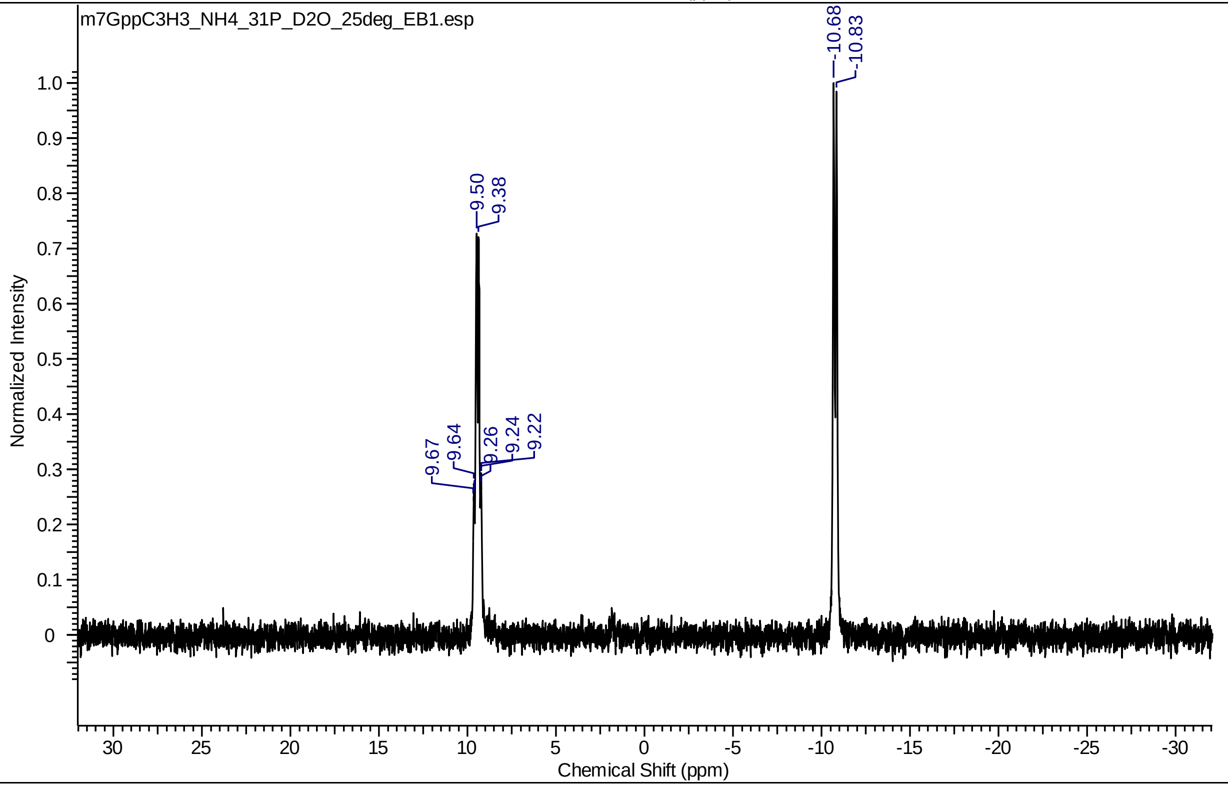

(11d) m^{7}GpppC$_3$H$_3$

**Structure**	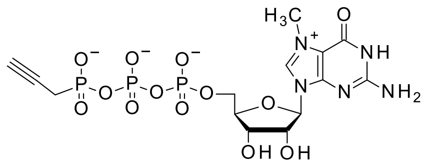

Reaction HPLC profile

Program A

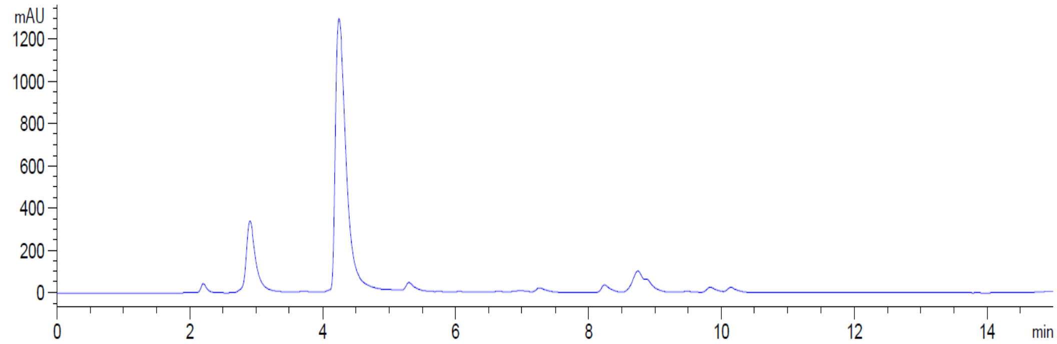

Purified compound HPLC profile

Program A

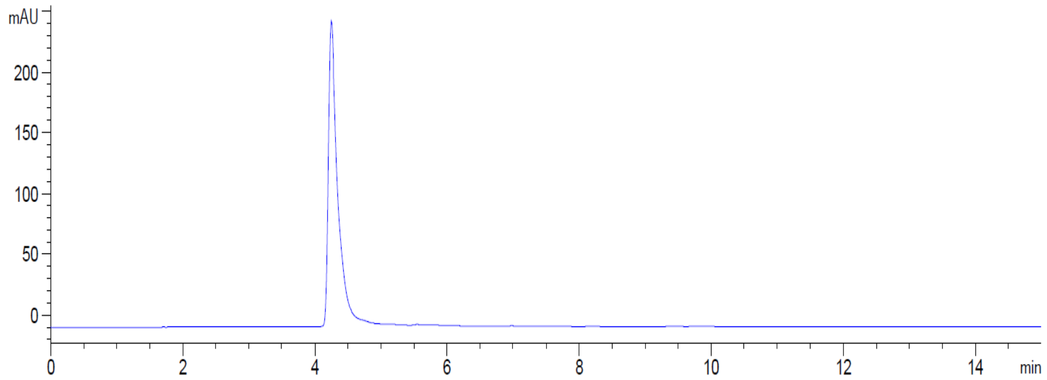

HRMS

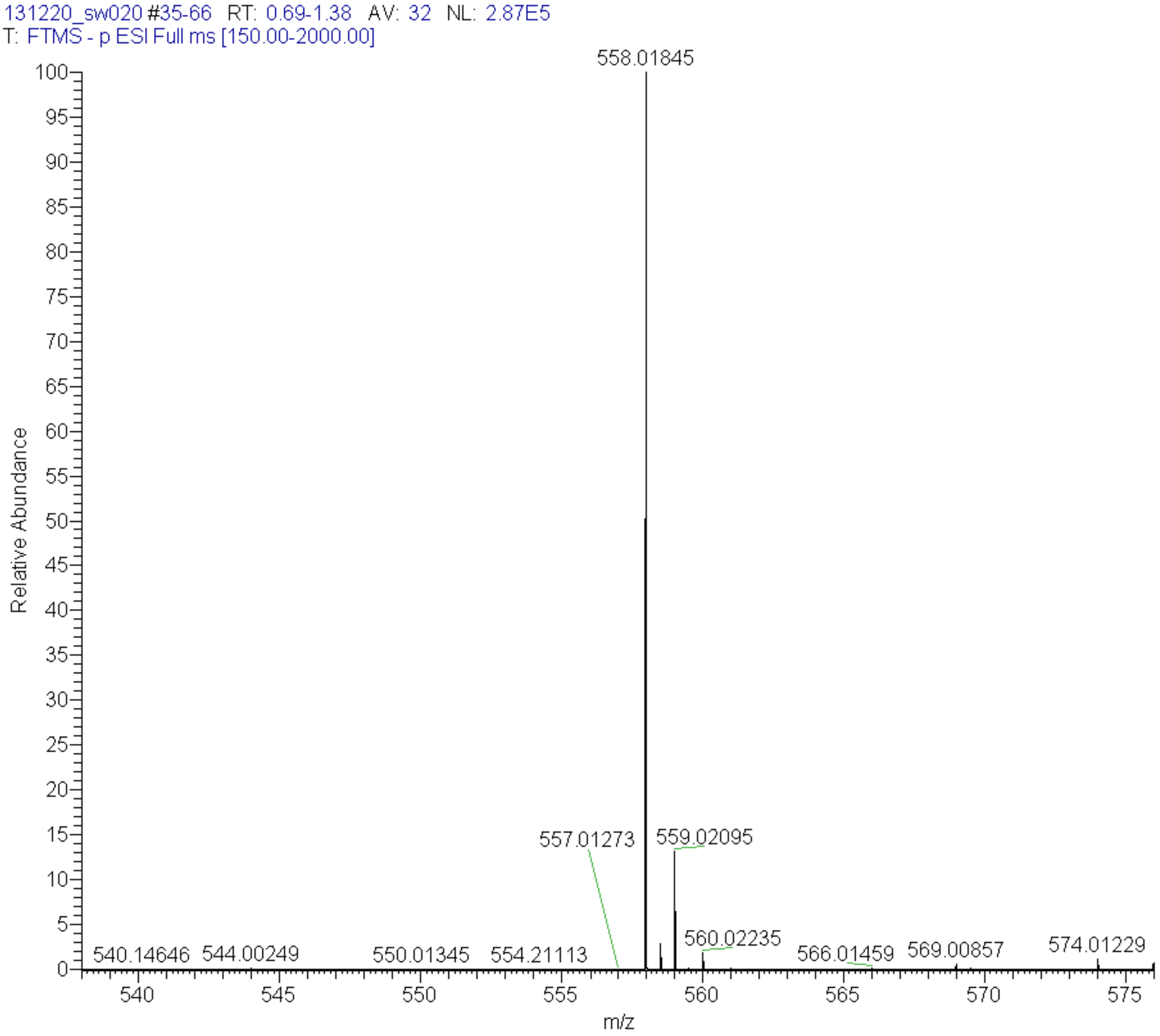

^{1}H NMR

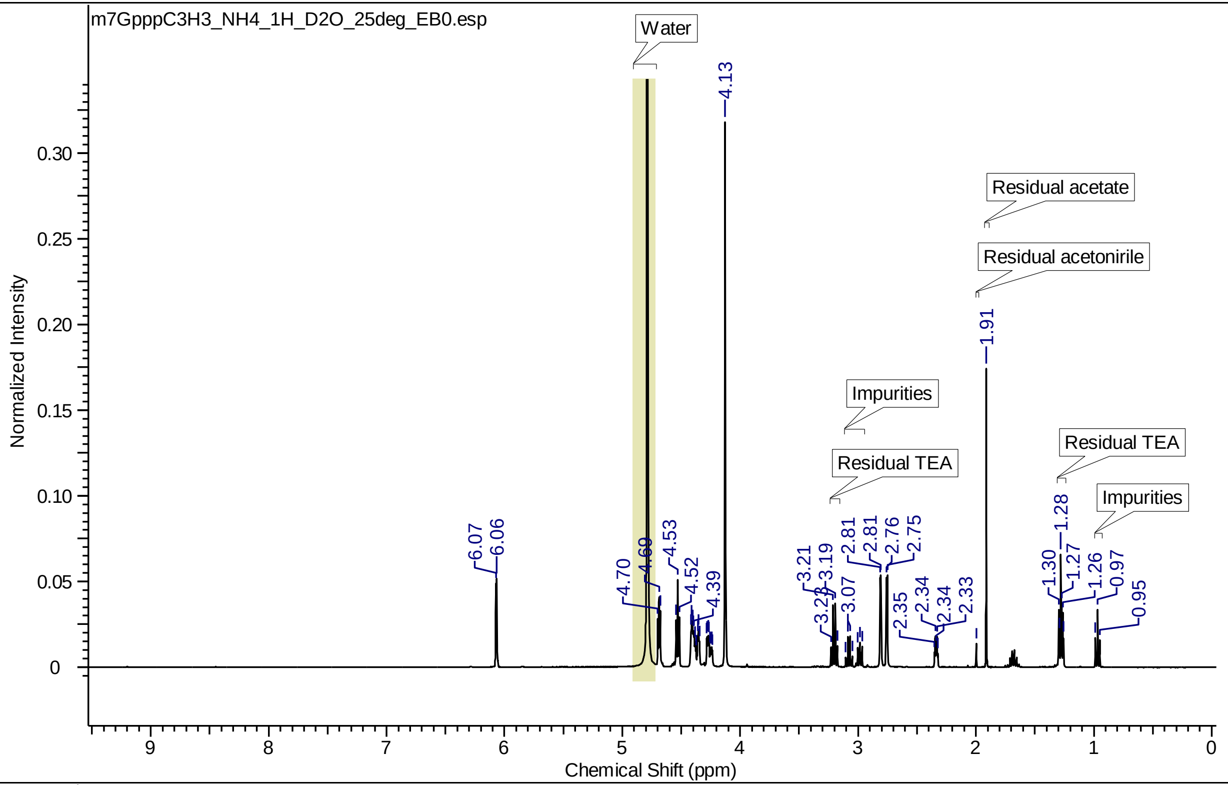

^{31}P NMR

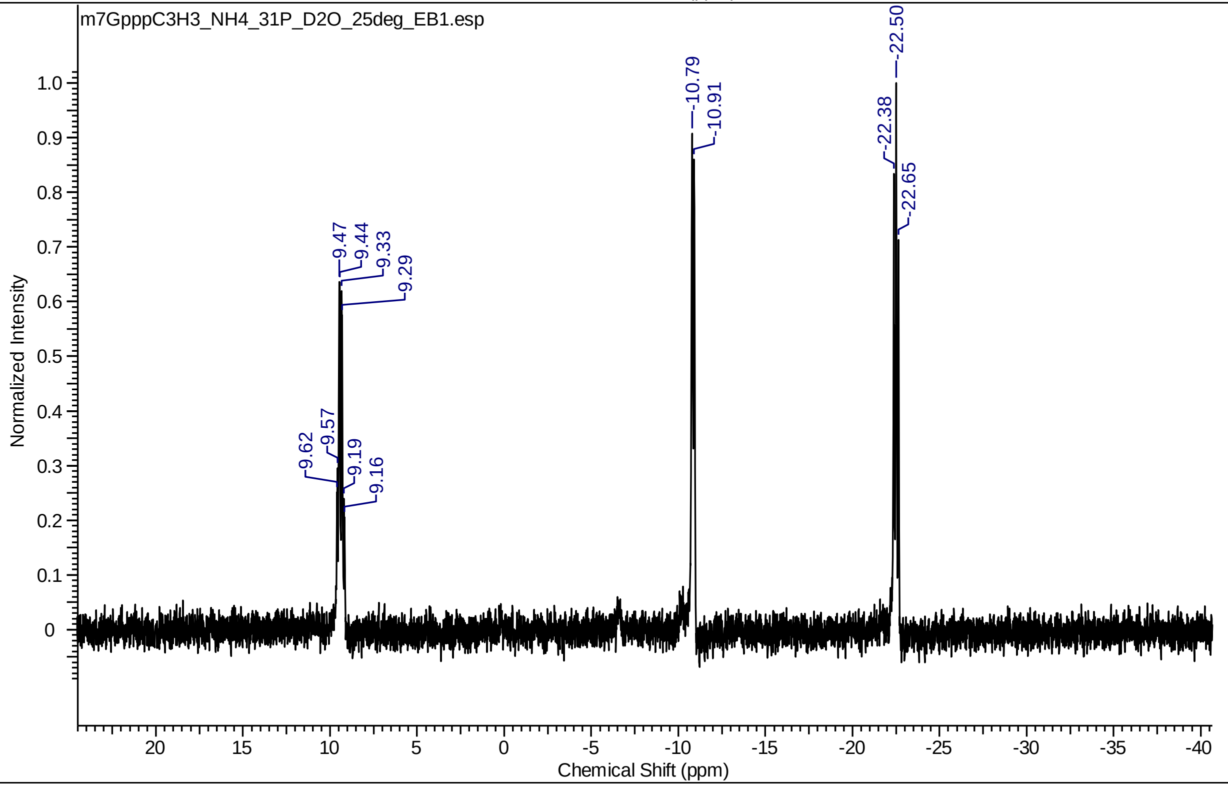

(12c) m^{7}GppC$_2$H

Structure

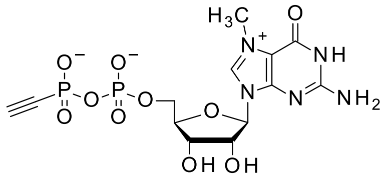

**Reaction HPLC profile**	Program A 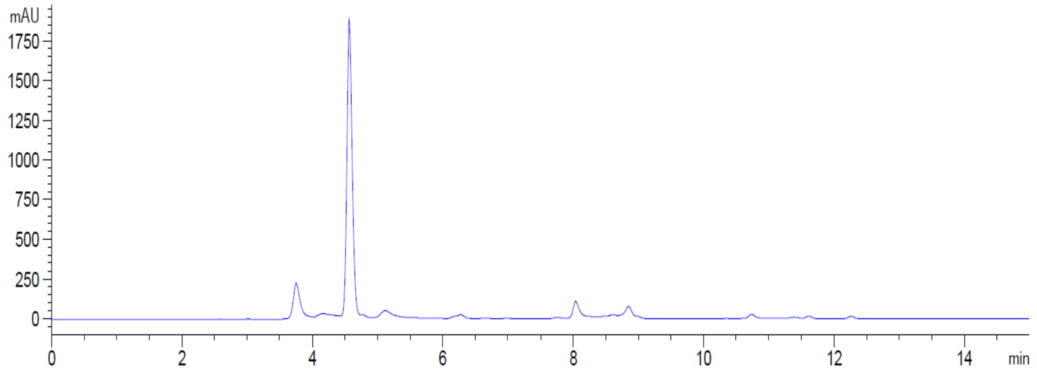
**Purified compound HPLC profile**	Program A 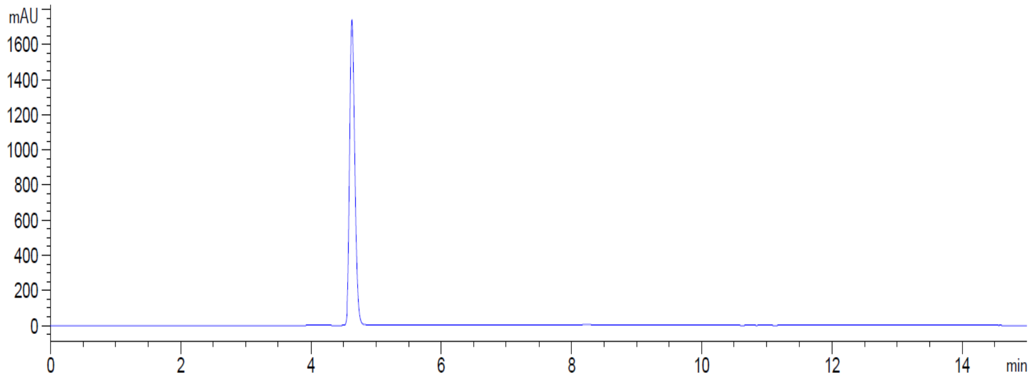
**HRMS**	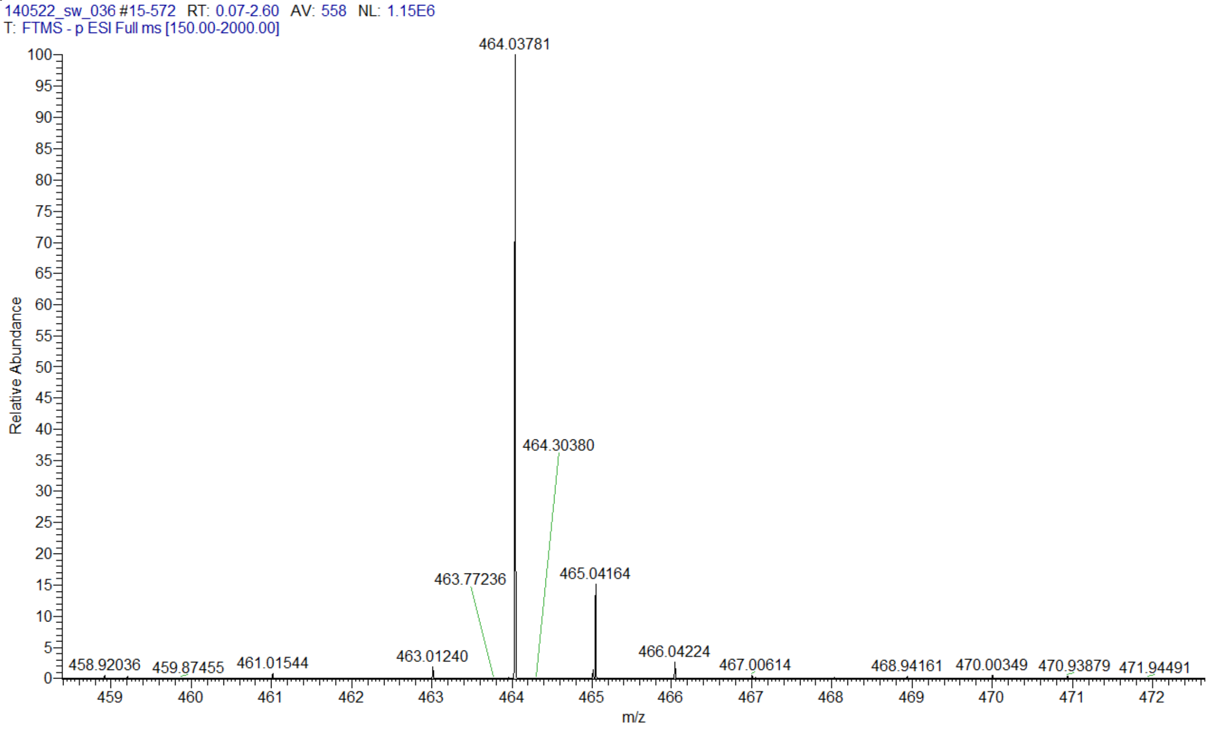

^{1}H NMR

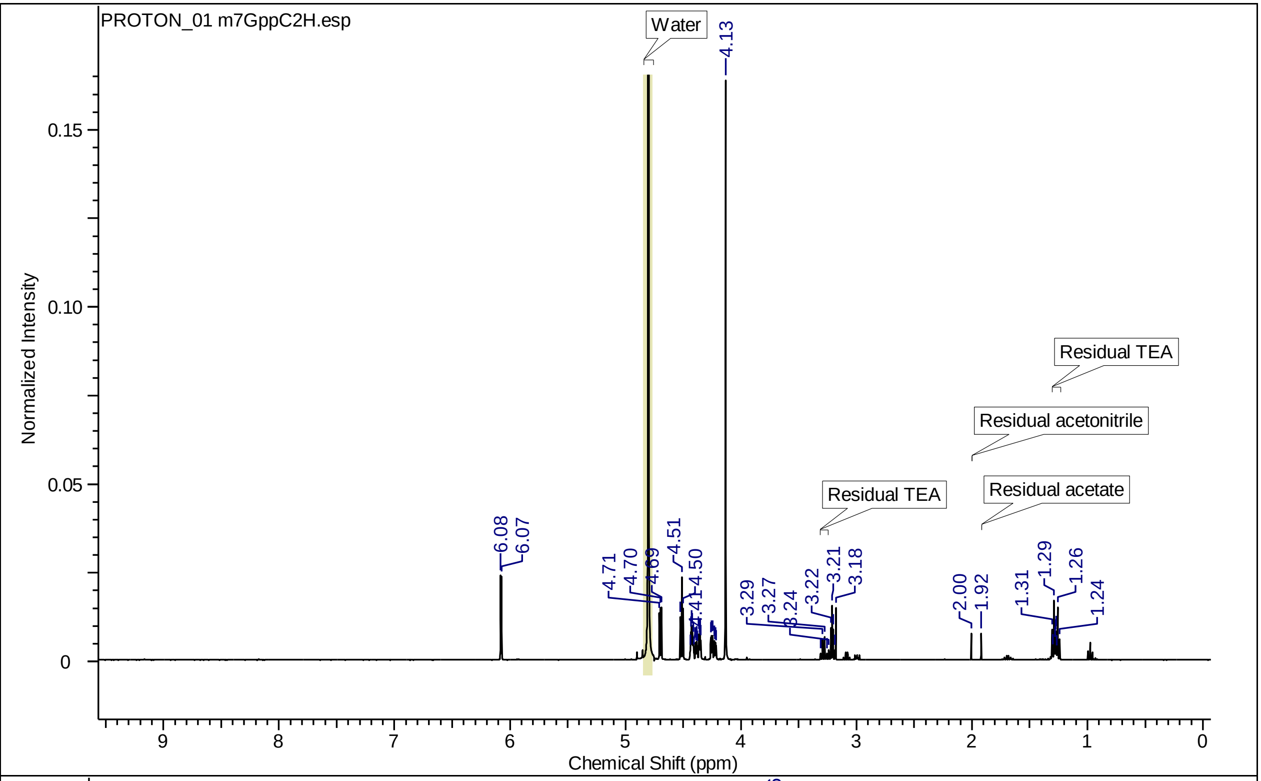

^{31}P NMR

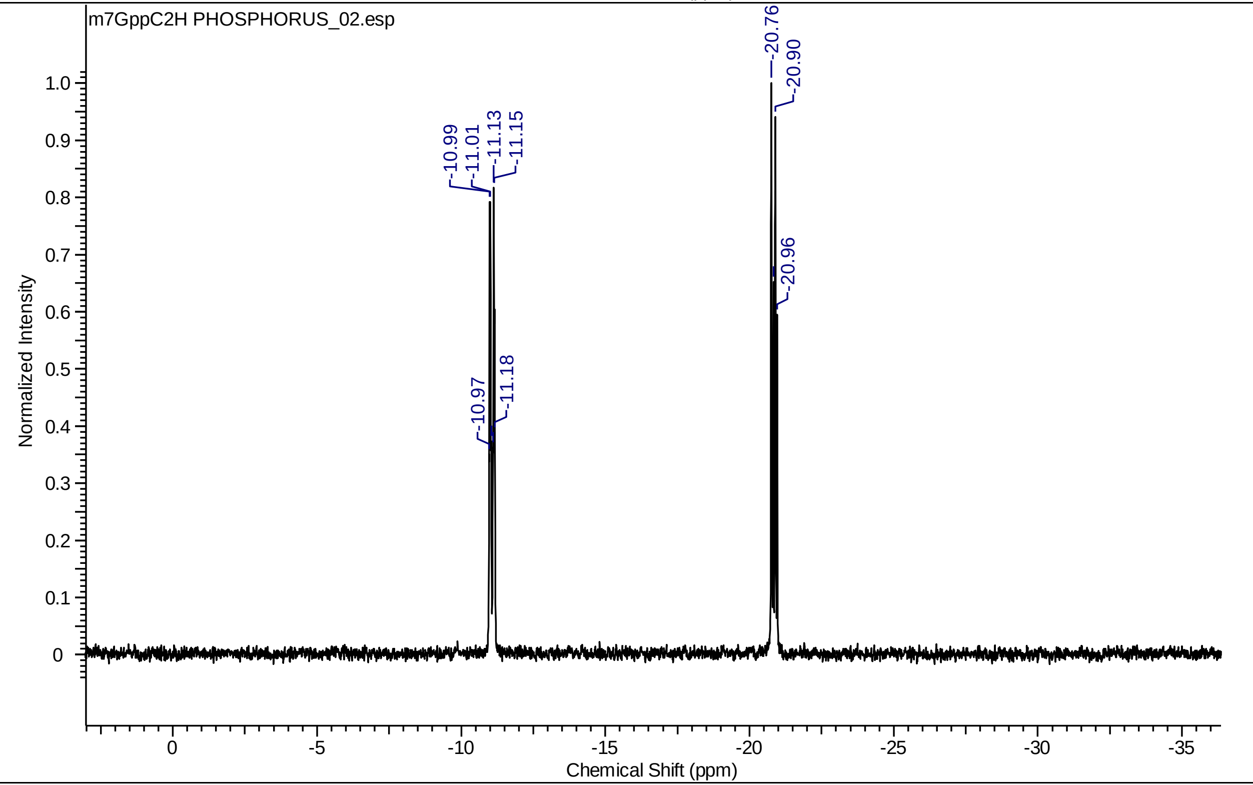

(12d) m^{7}GpppC$_2$H

Structure

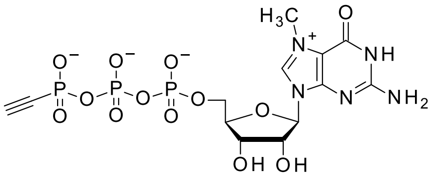

**Reaction HPLC profile**	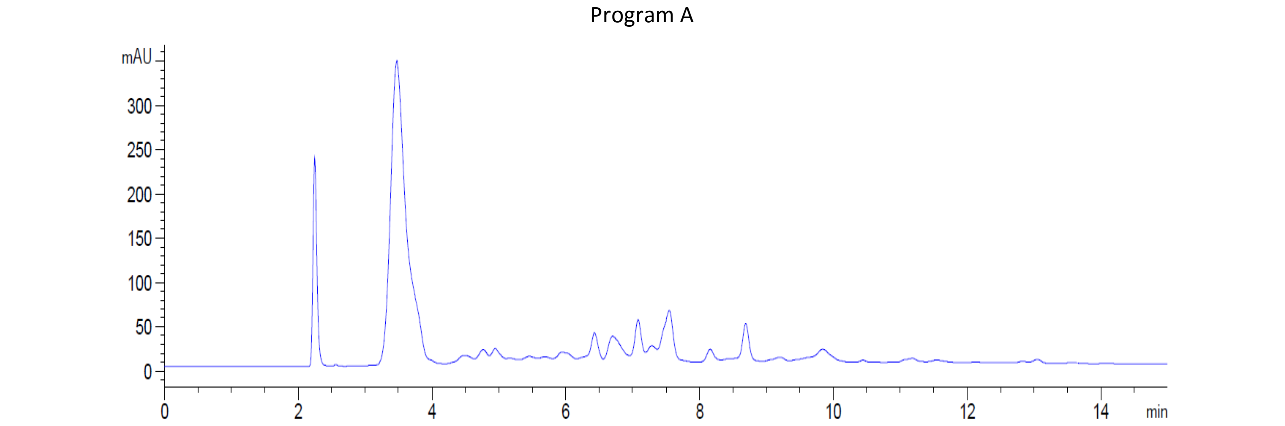
**Purified compound HPLC profile**	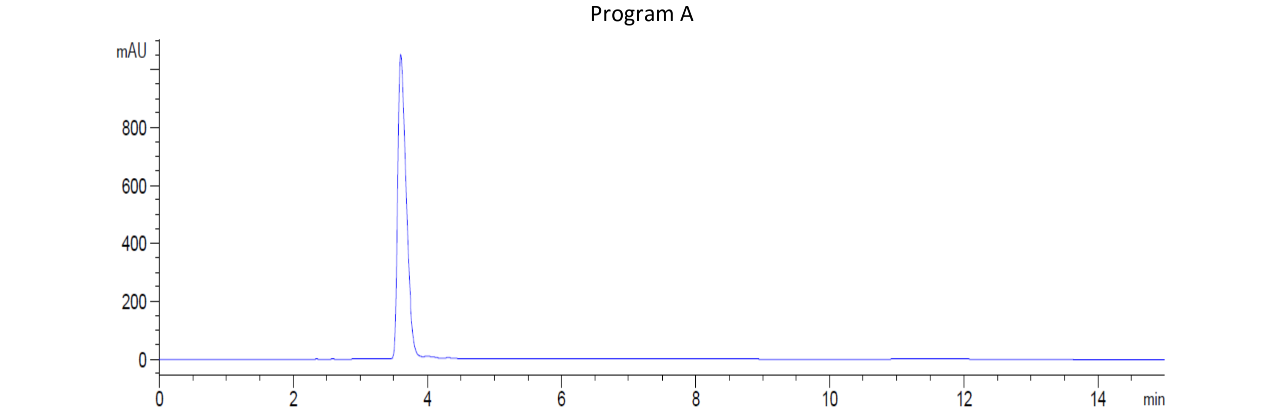
**HRMS**	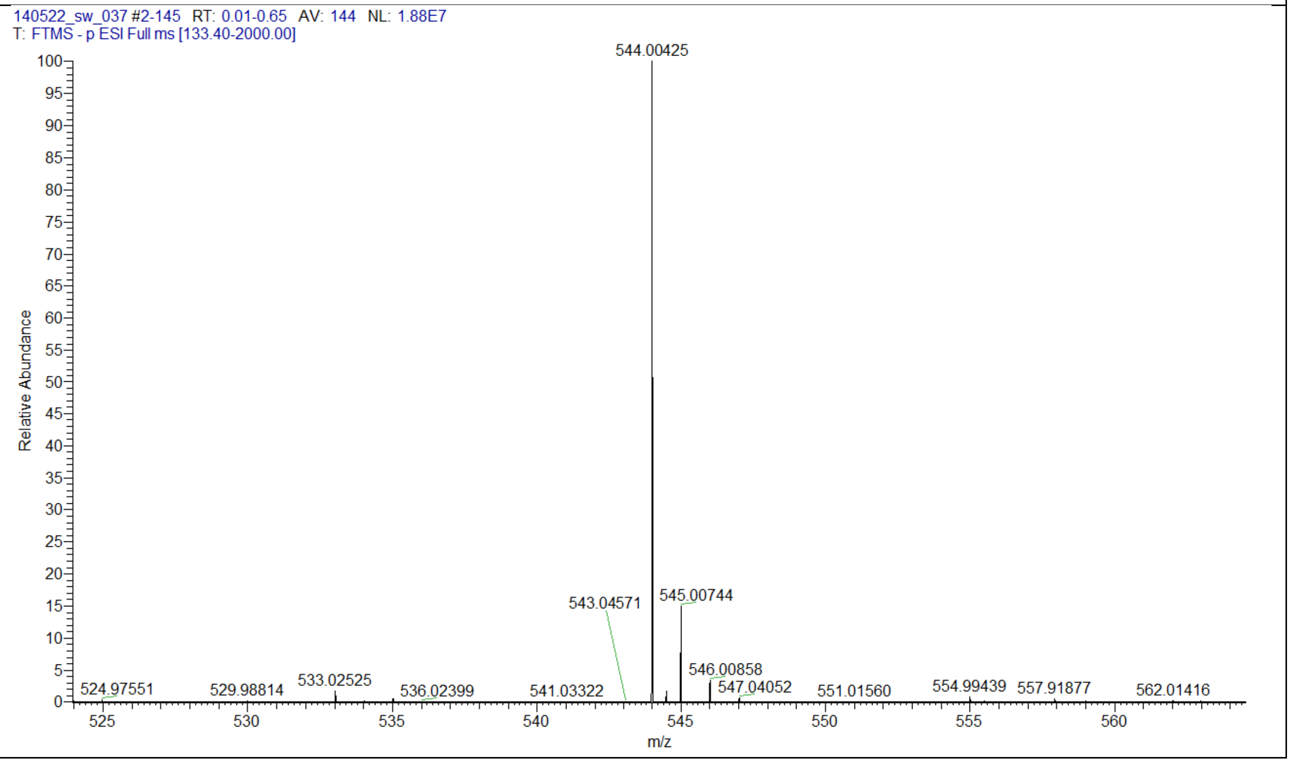

**^{1}H NMR**	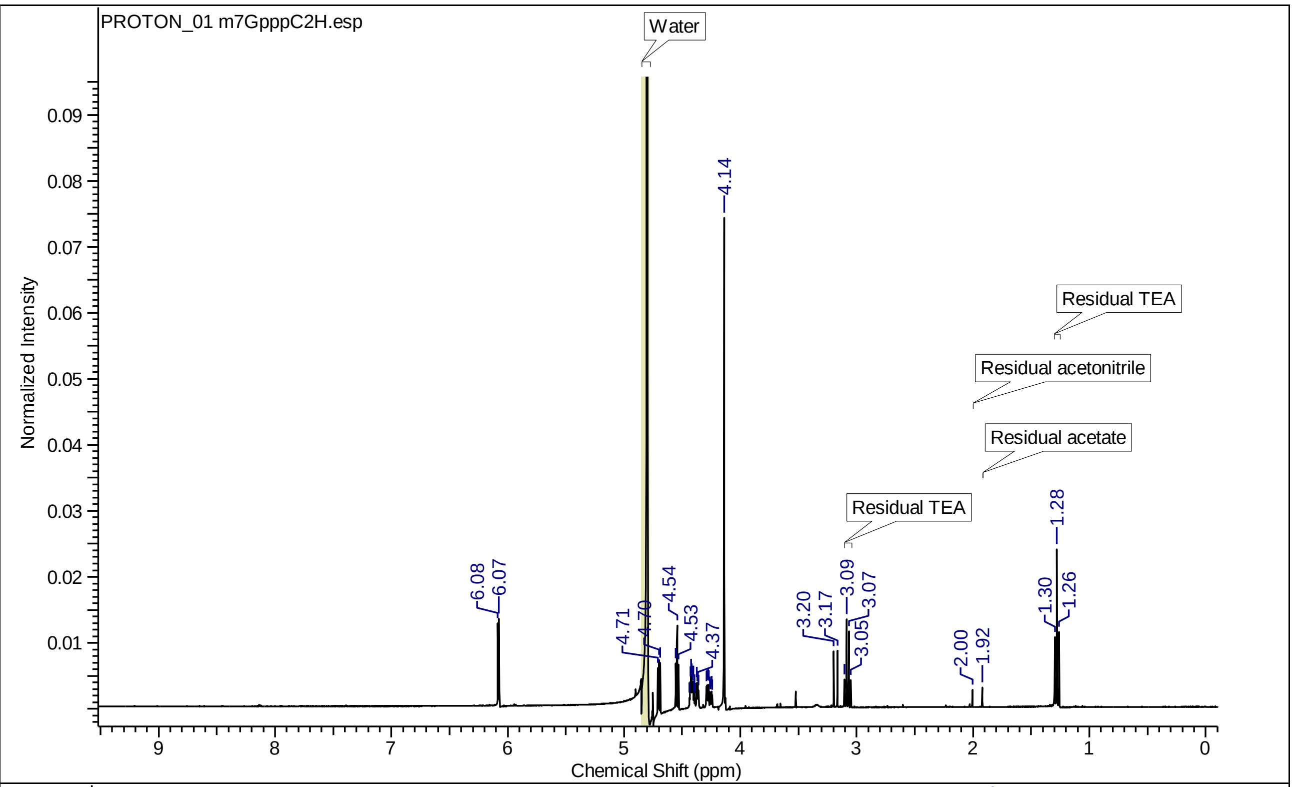
**^{31}P NMR**	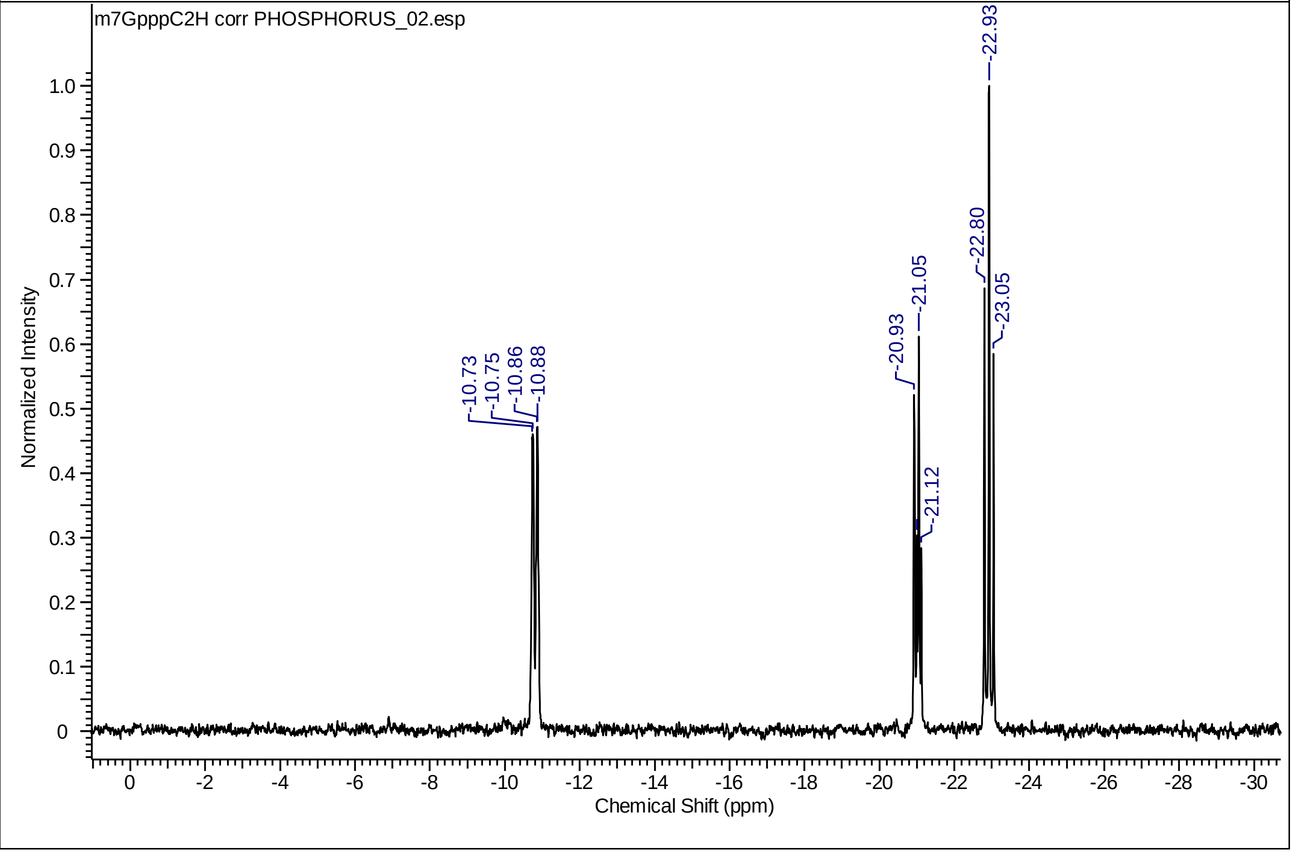

(12e) m$^{2'\text{-}O}$GpppC$_2$H

**Structure**	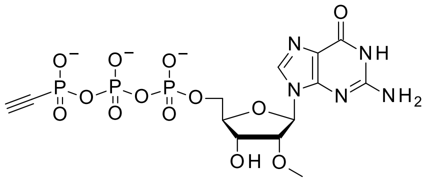

Reaction HPLC profile

Program A; before and after the reaction

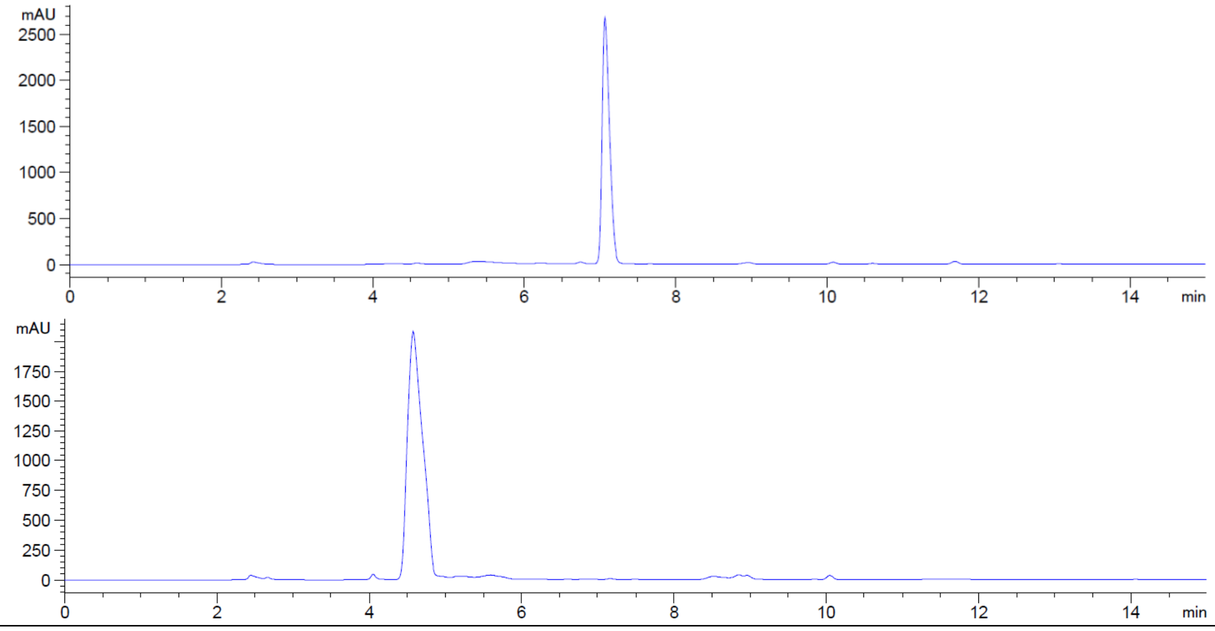

Purified compound HPLC profile

Program A

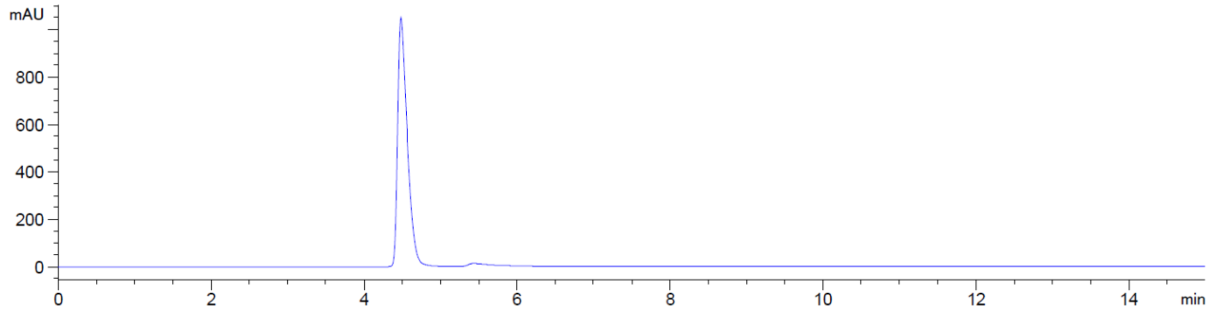

HRMS

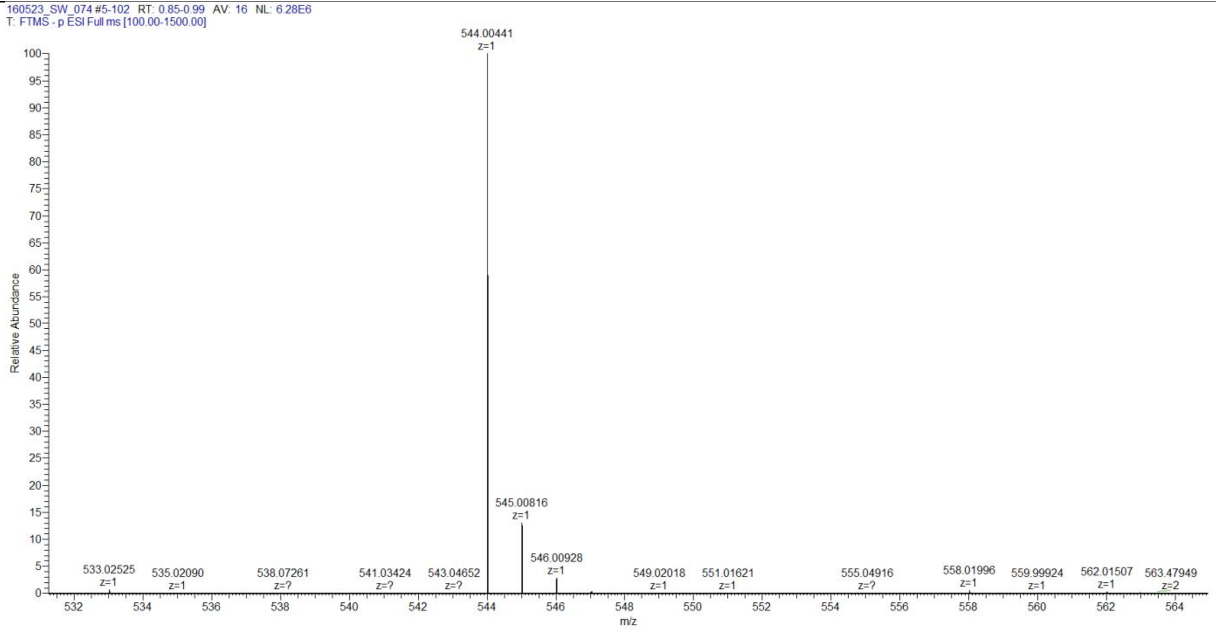

**^{1}H NMR**	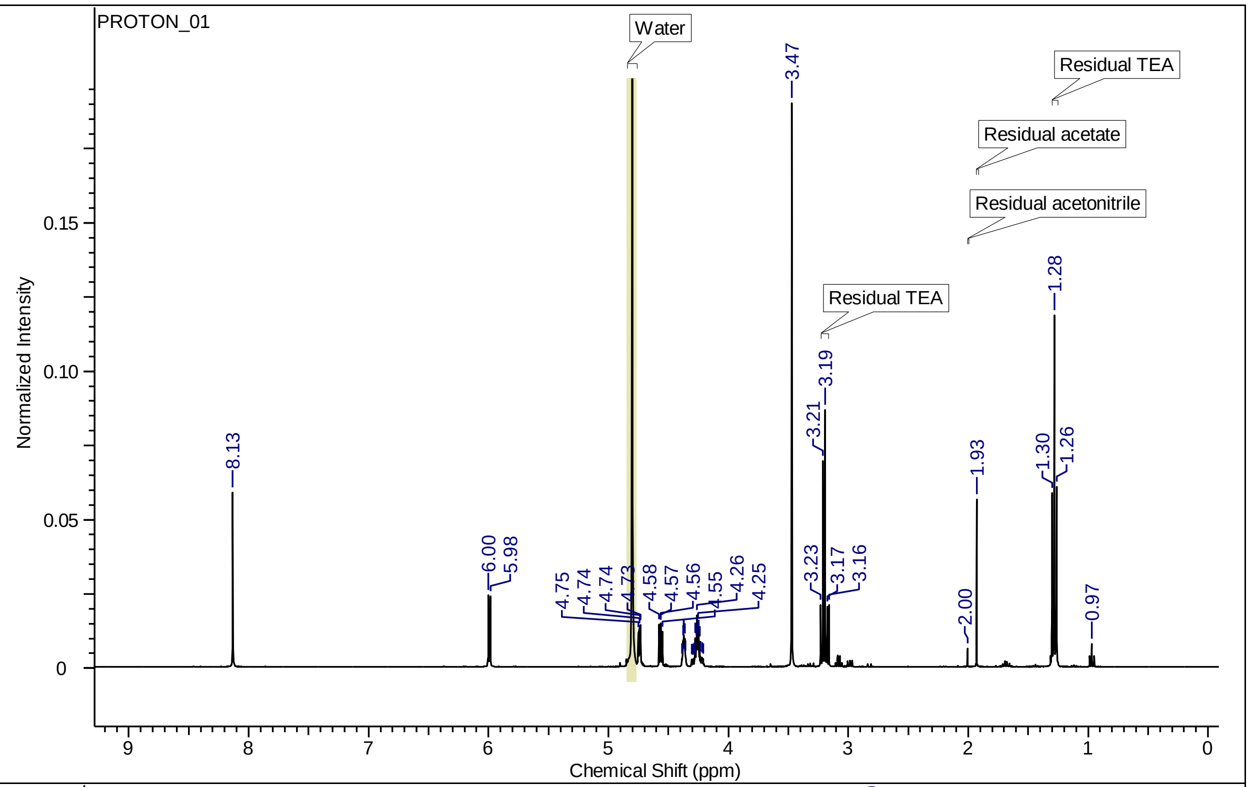
**^{31}P NMR**	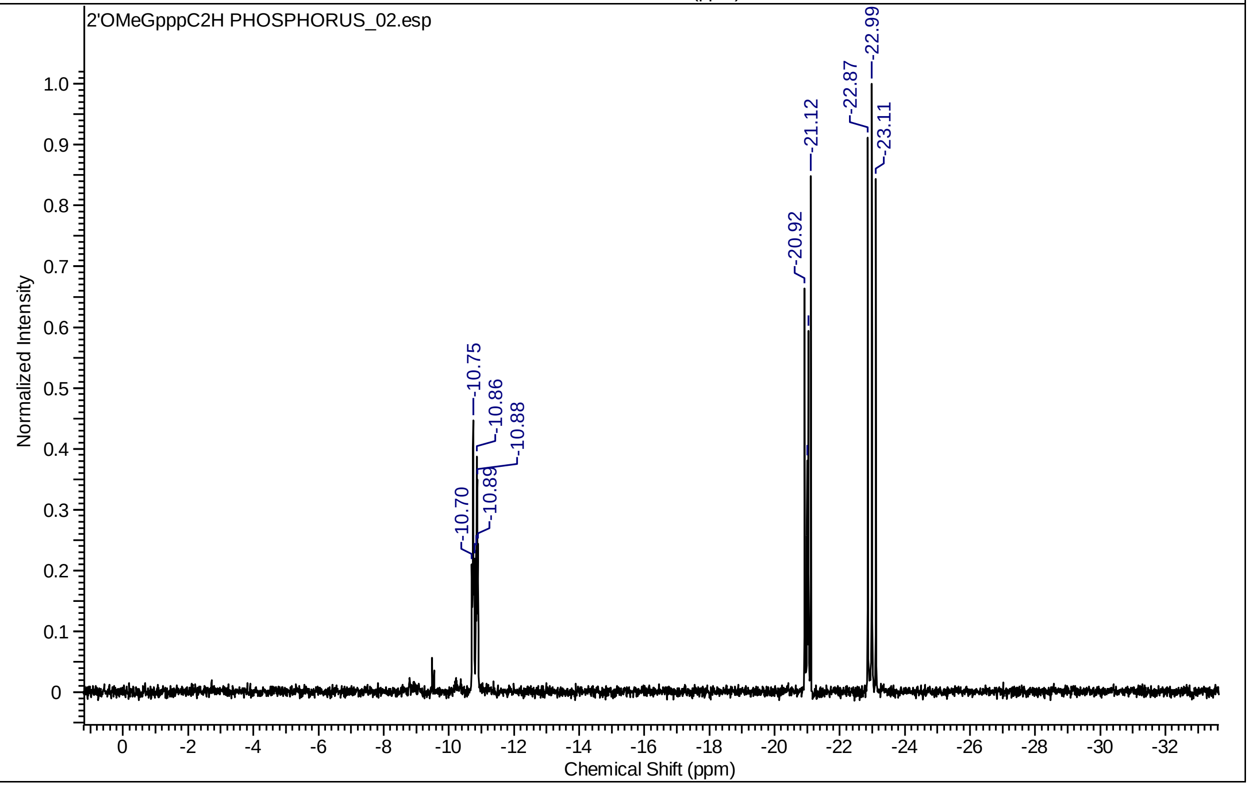

(12f) $m_2^{7,2'-O}GpppC_2H$

**Structure**	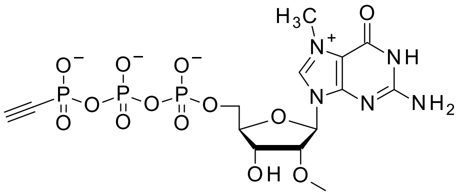

**Reaction HPLC profile**	Program A; before and after the reaction<br>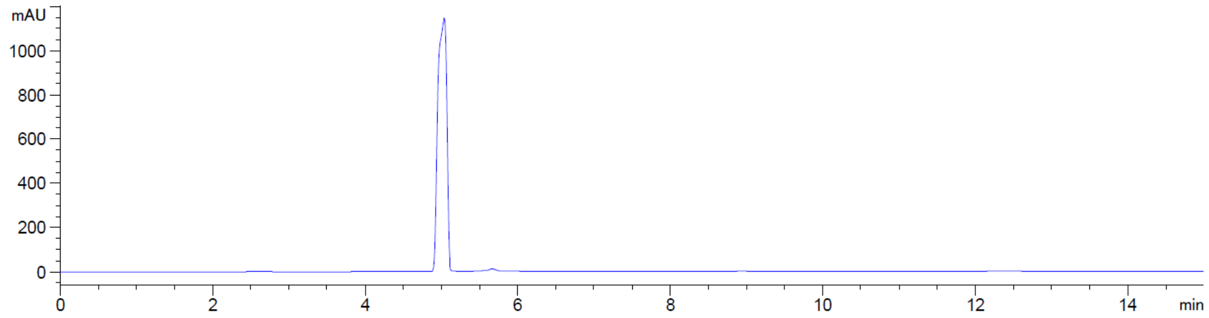<br><br>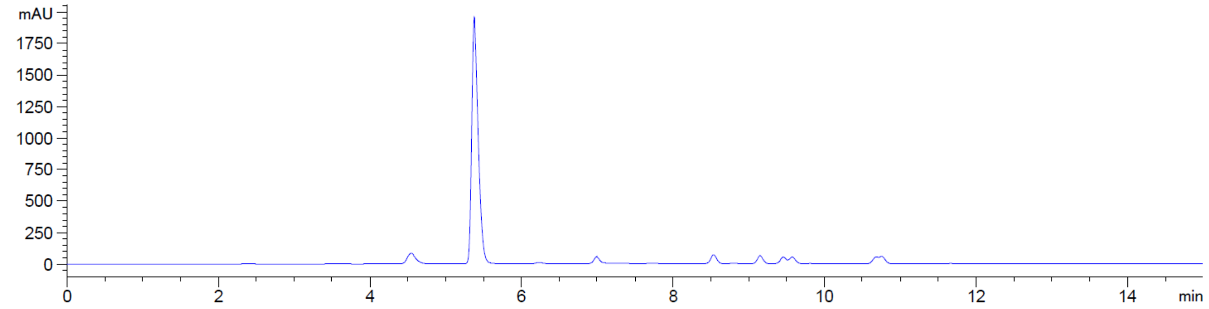
**Purified compound HPLC profile**	Program A<br>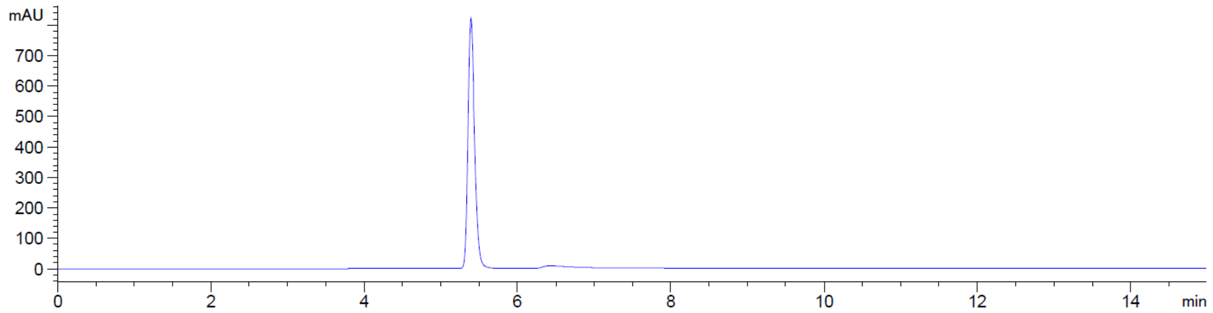
**HRMS**	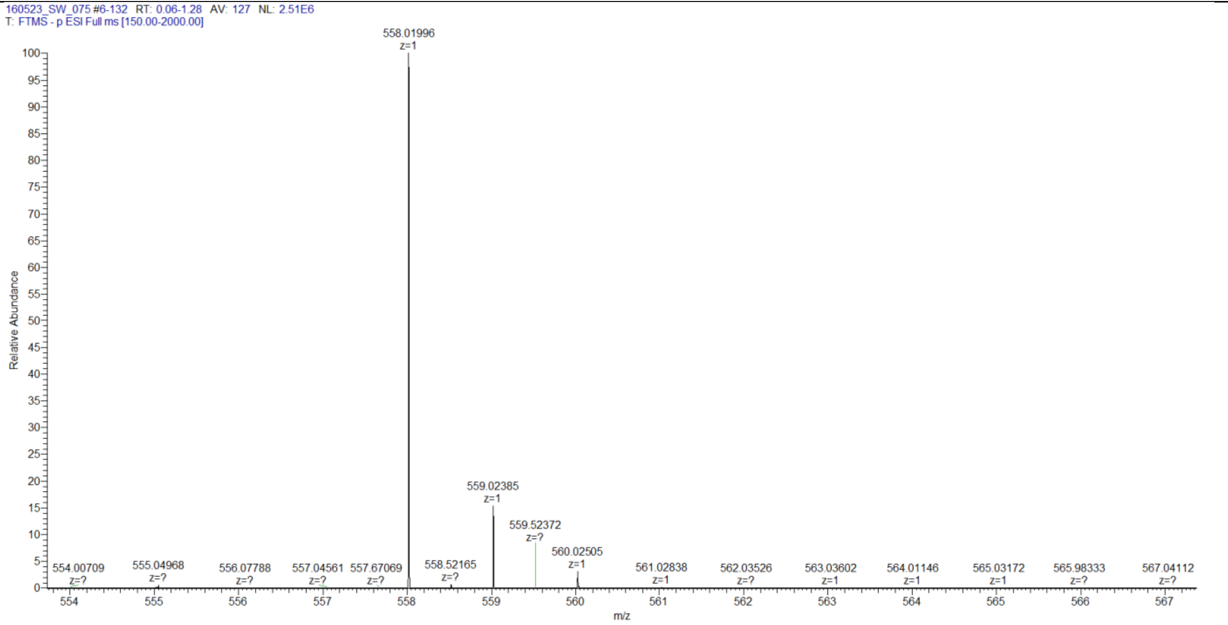

^{1}H NMR

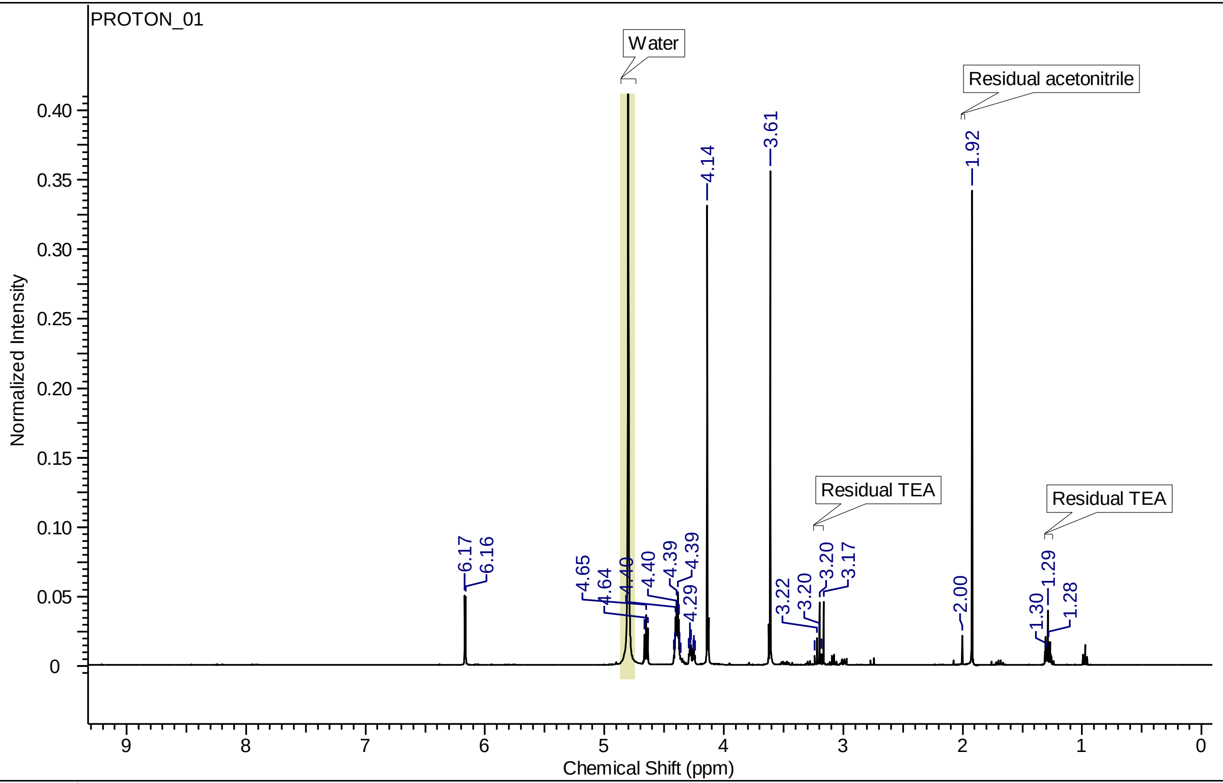

^{31}P NMR

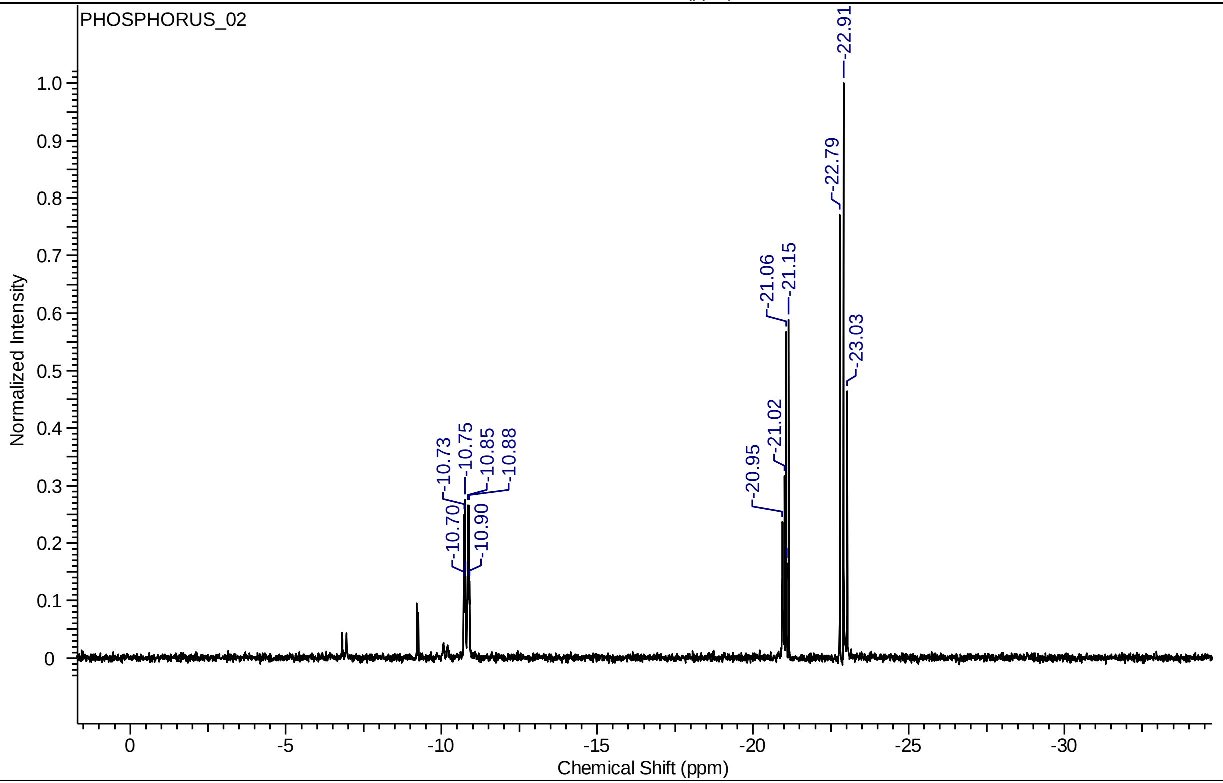

(13a) $GppOC_3H_3$

Structure

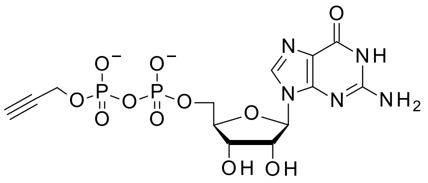

**Reaction HPLC profile**	Program A 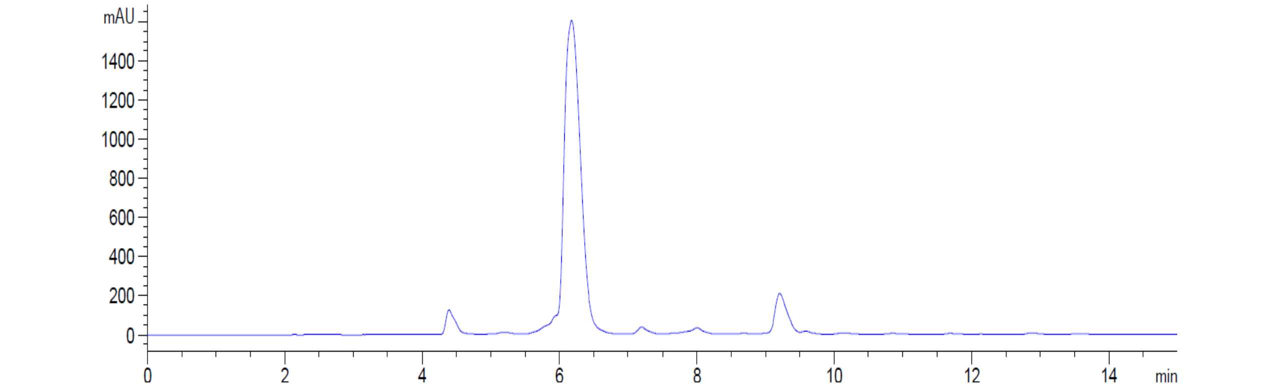
**Purified compound HPLC profile**	Program A 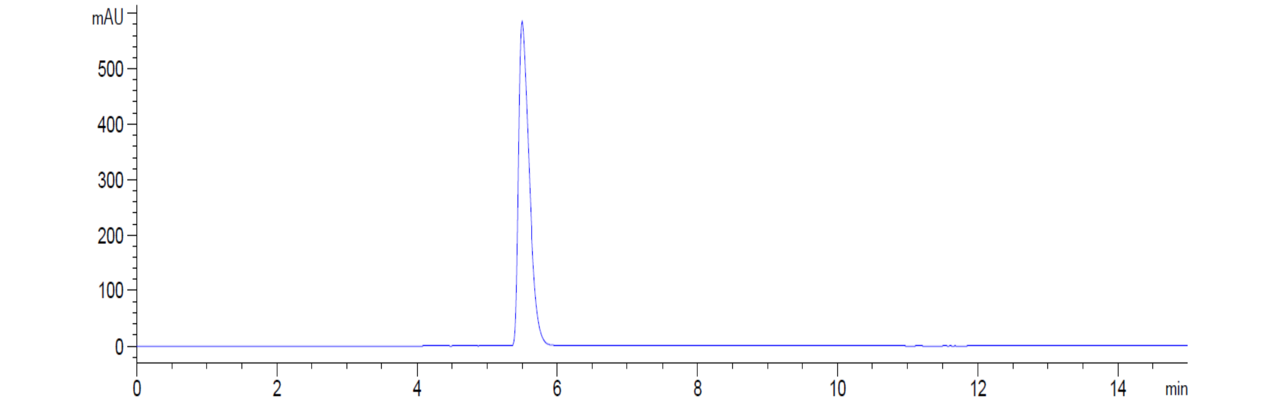
**HRMS**	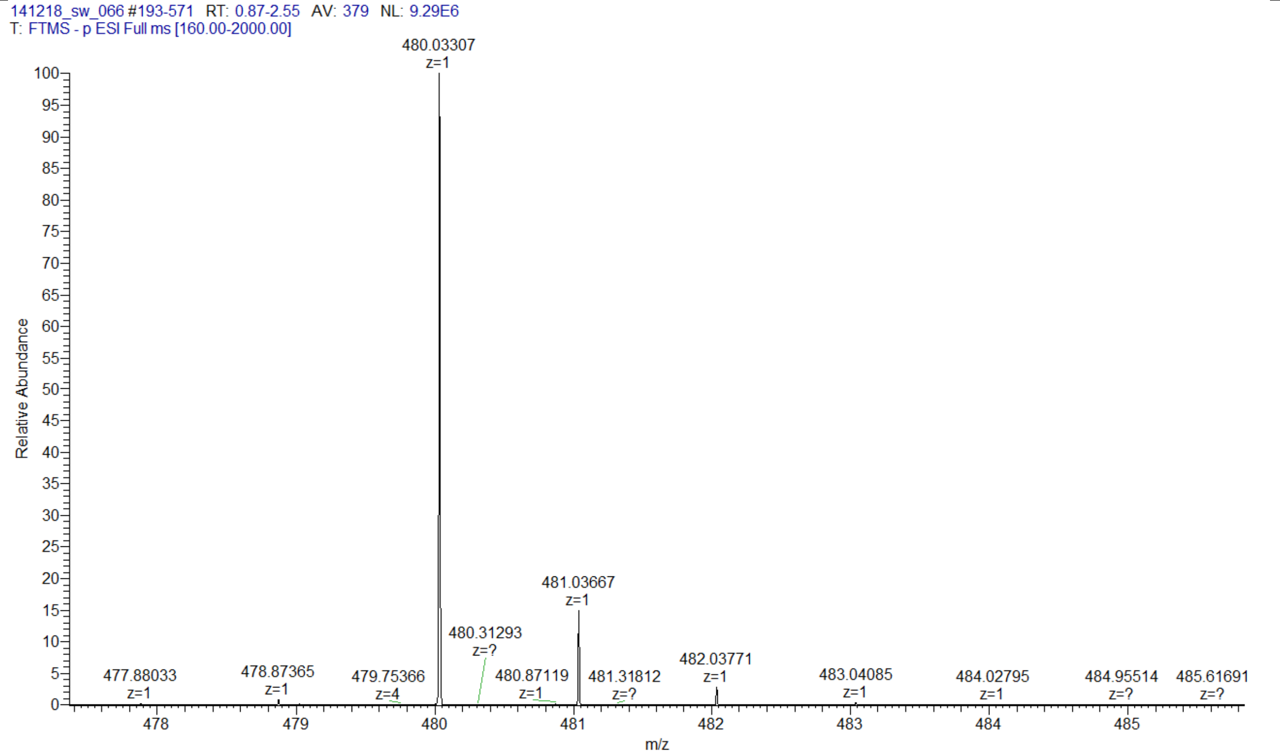

**^{1}H NMR**	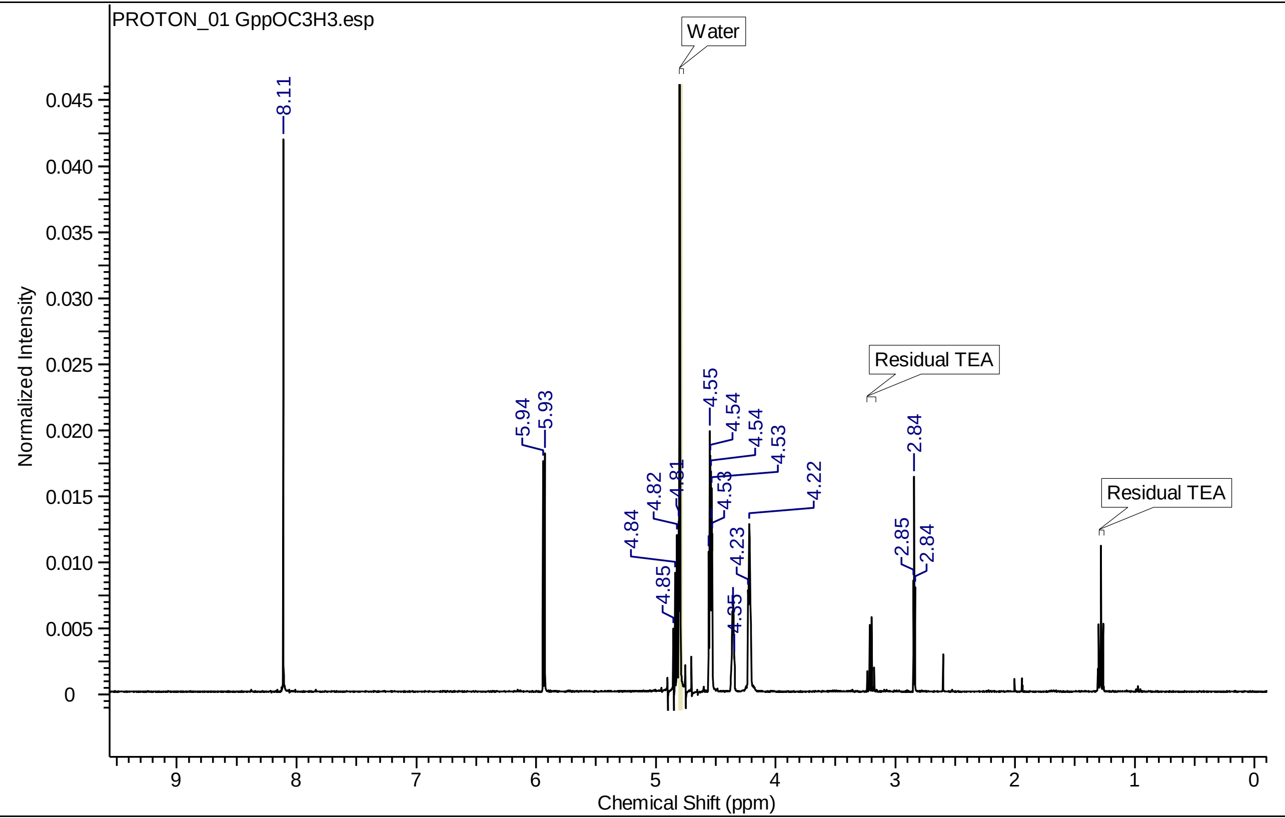
**^{31}P NMR**	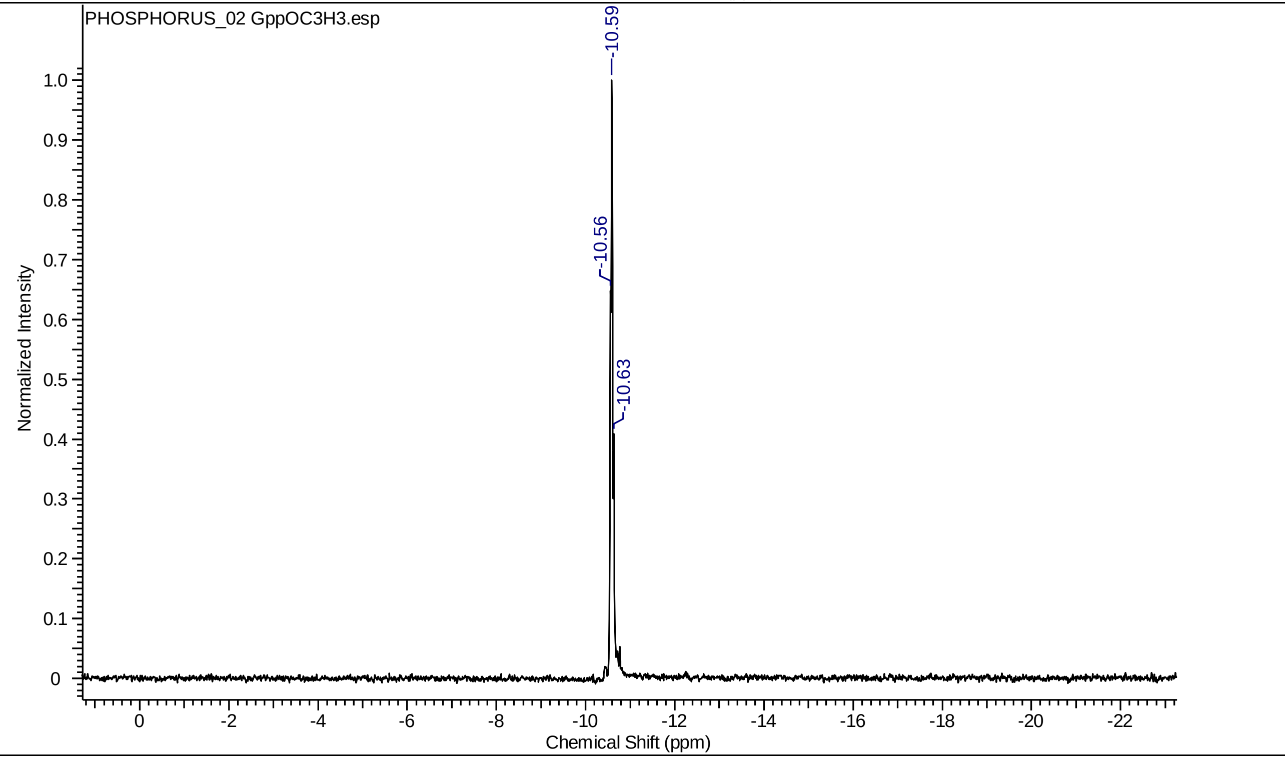

(13b) GpppOC$_3$H$_3$

**Structure**	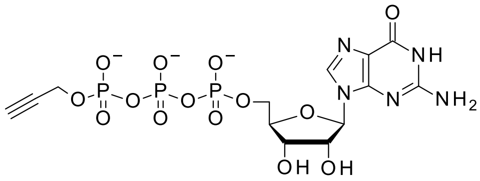

**Reaction HPLC profile**	Program A<br>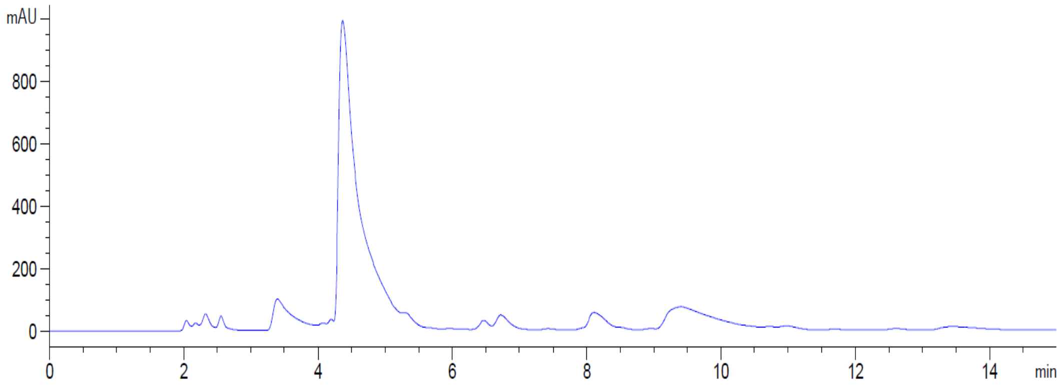
**Purified compound HPLC profile**	Program A<br>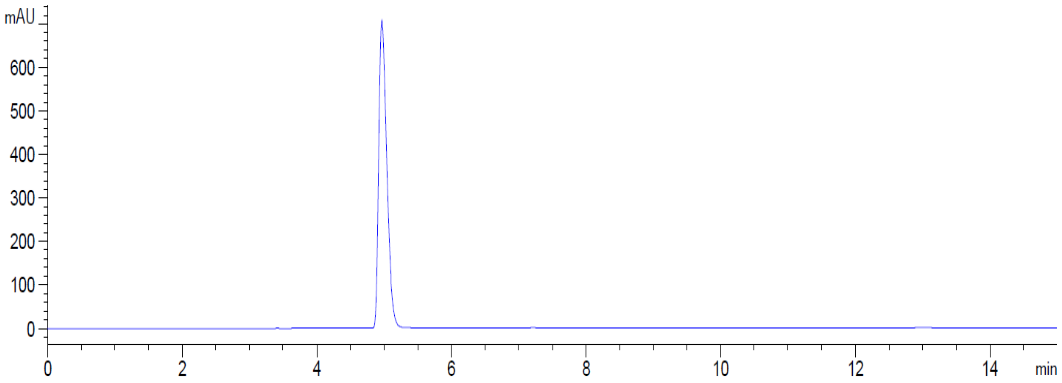
**HRMS**	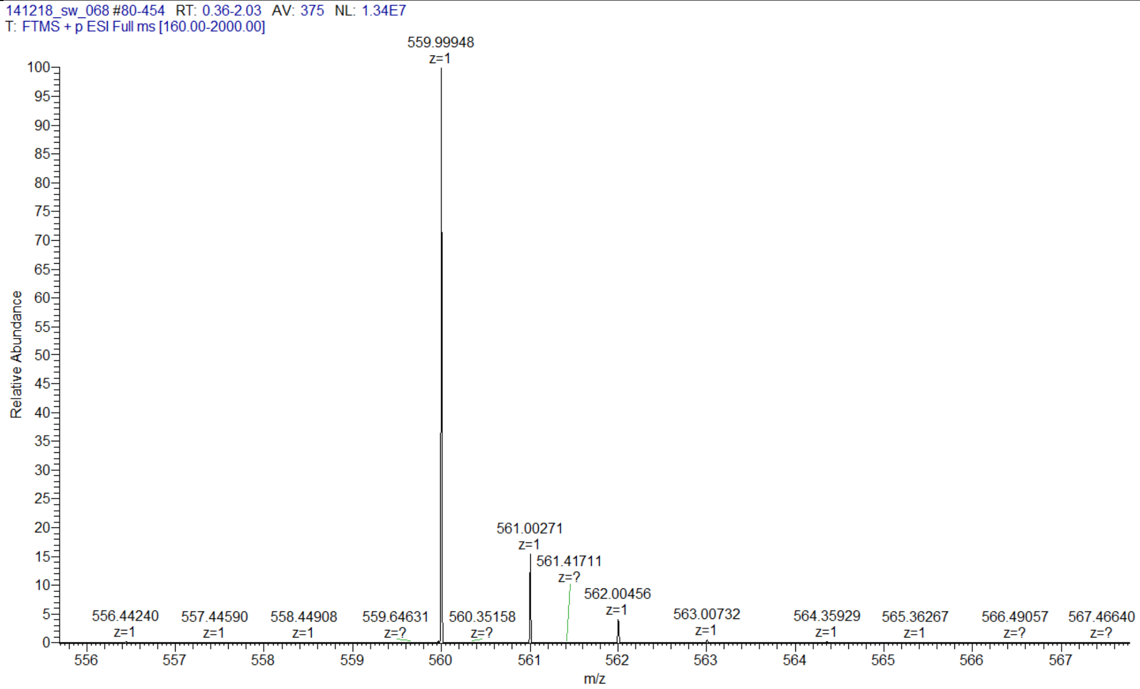

**^{1}H NMR**	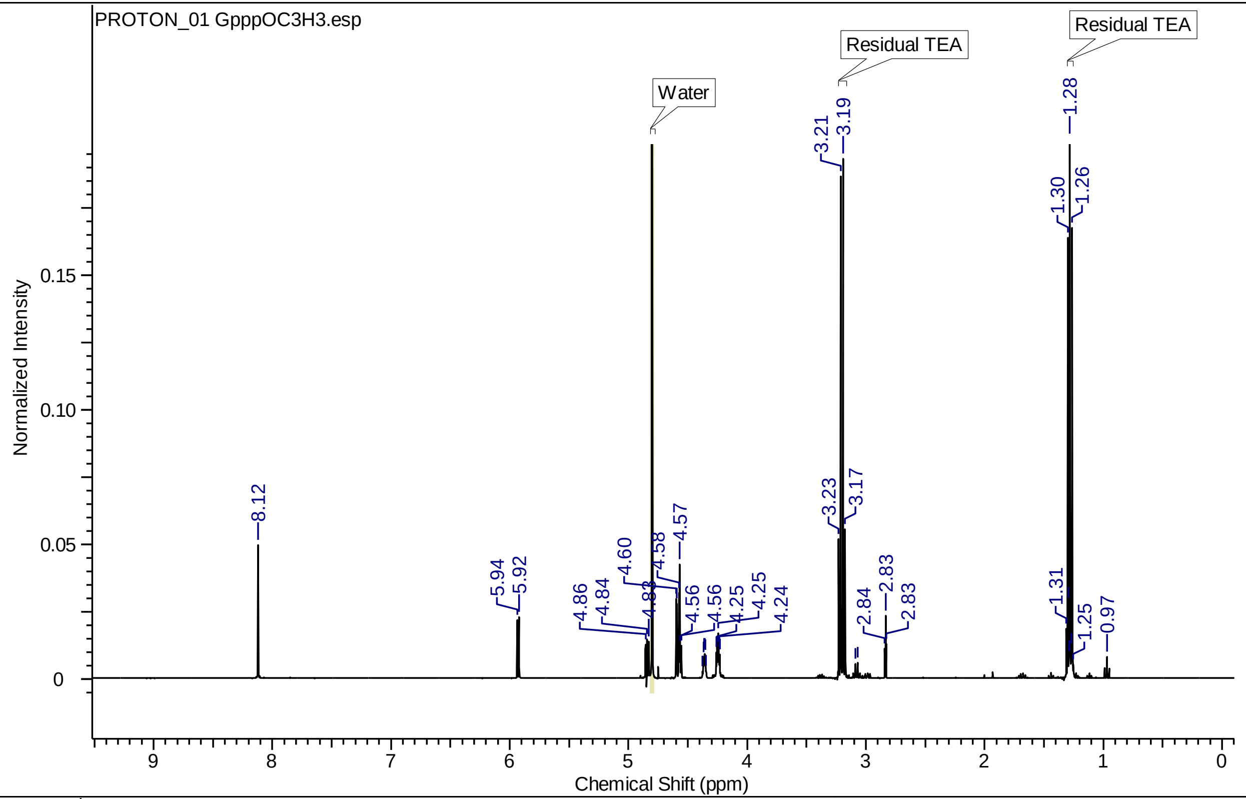
**^{31}P NMR**	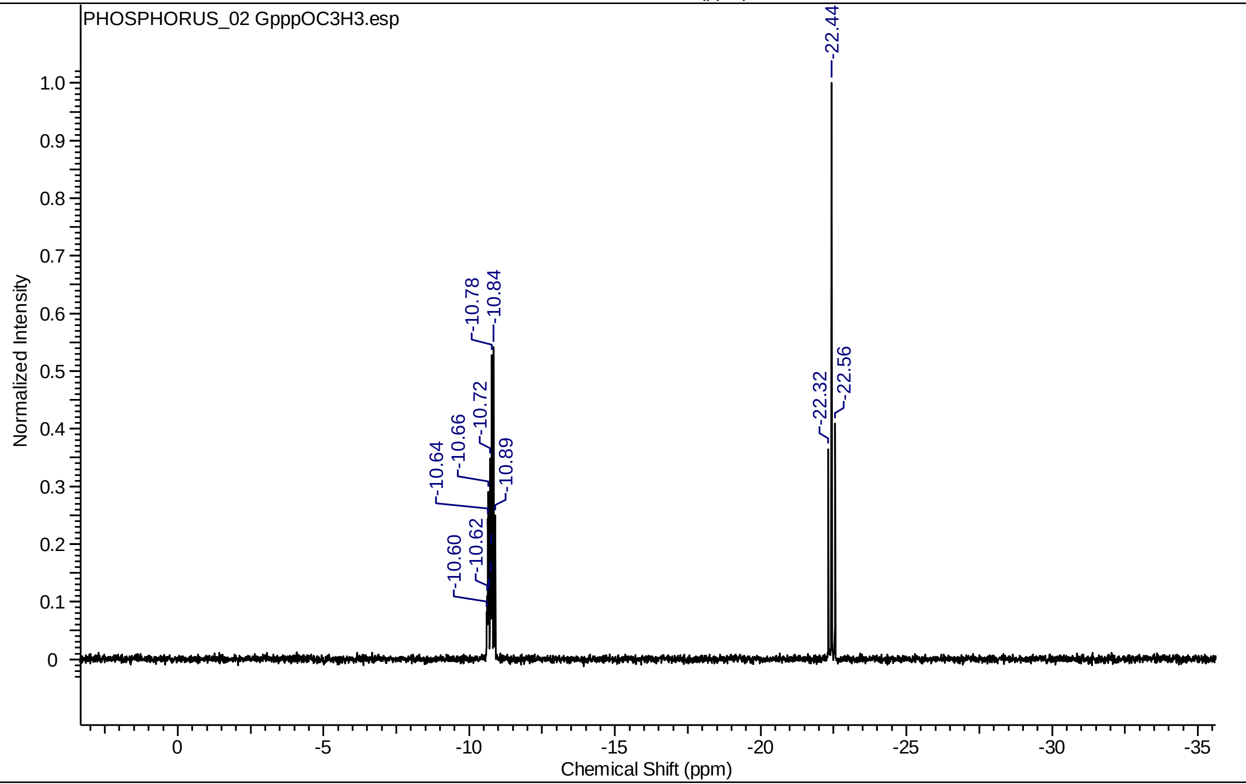

(13c) m^{7}GppOC$_3$H$_3$

**Structure**	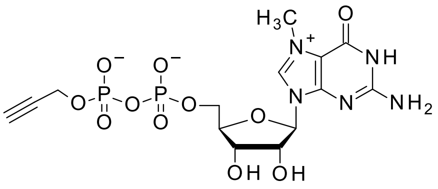

Reaction HPLC profile

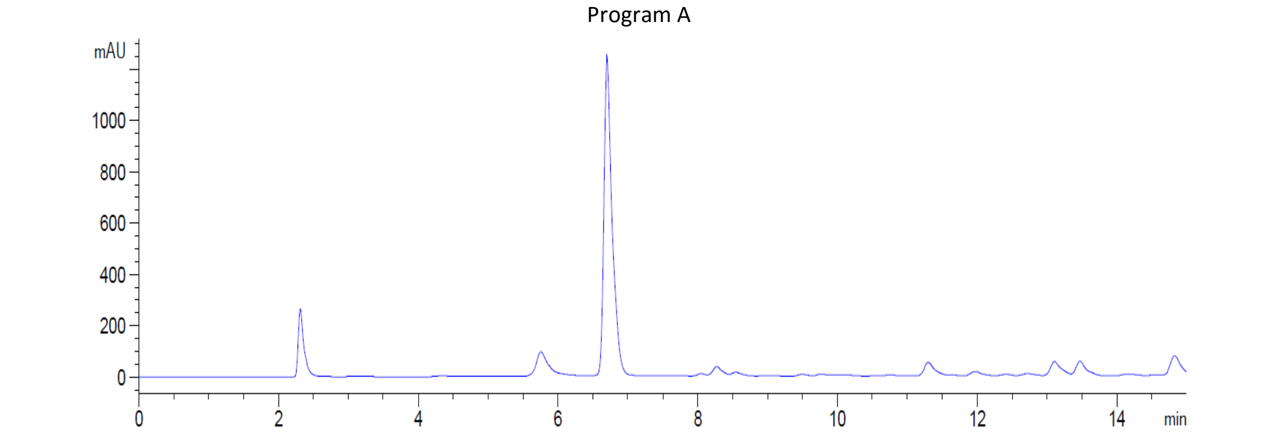

Purified compound HPLC profile

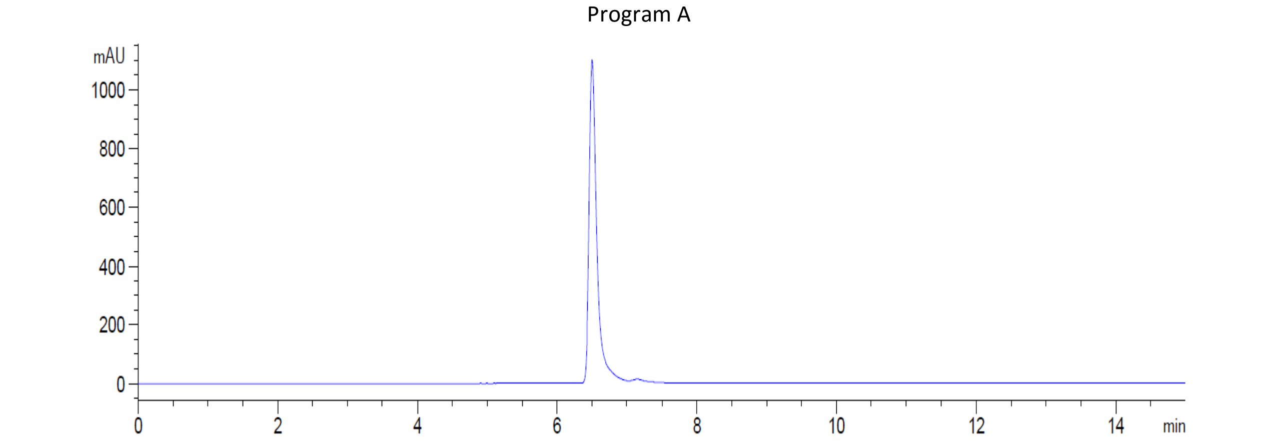

HRMS

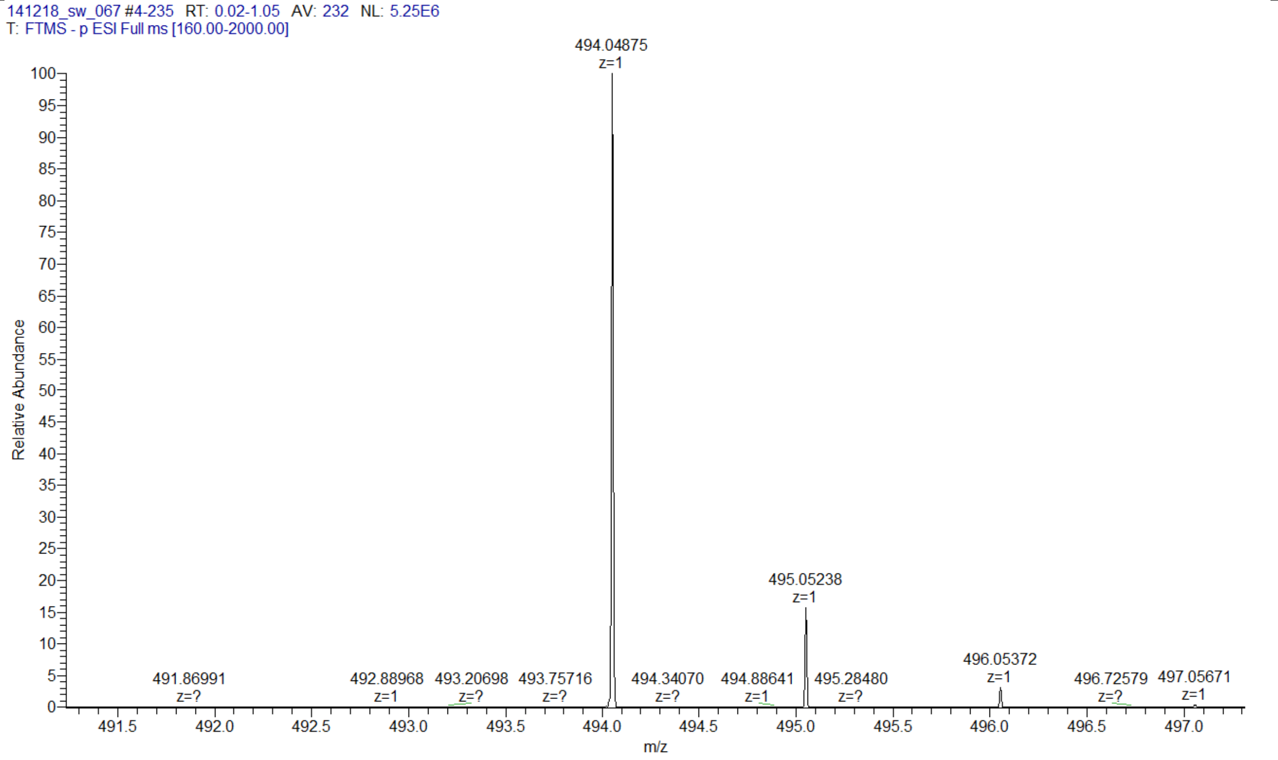

^{1}H NMR

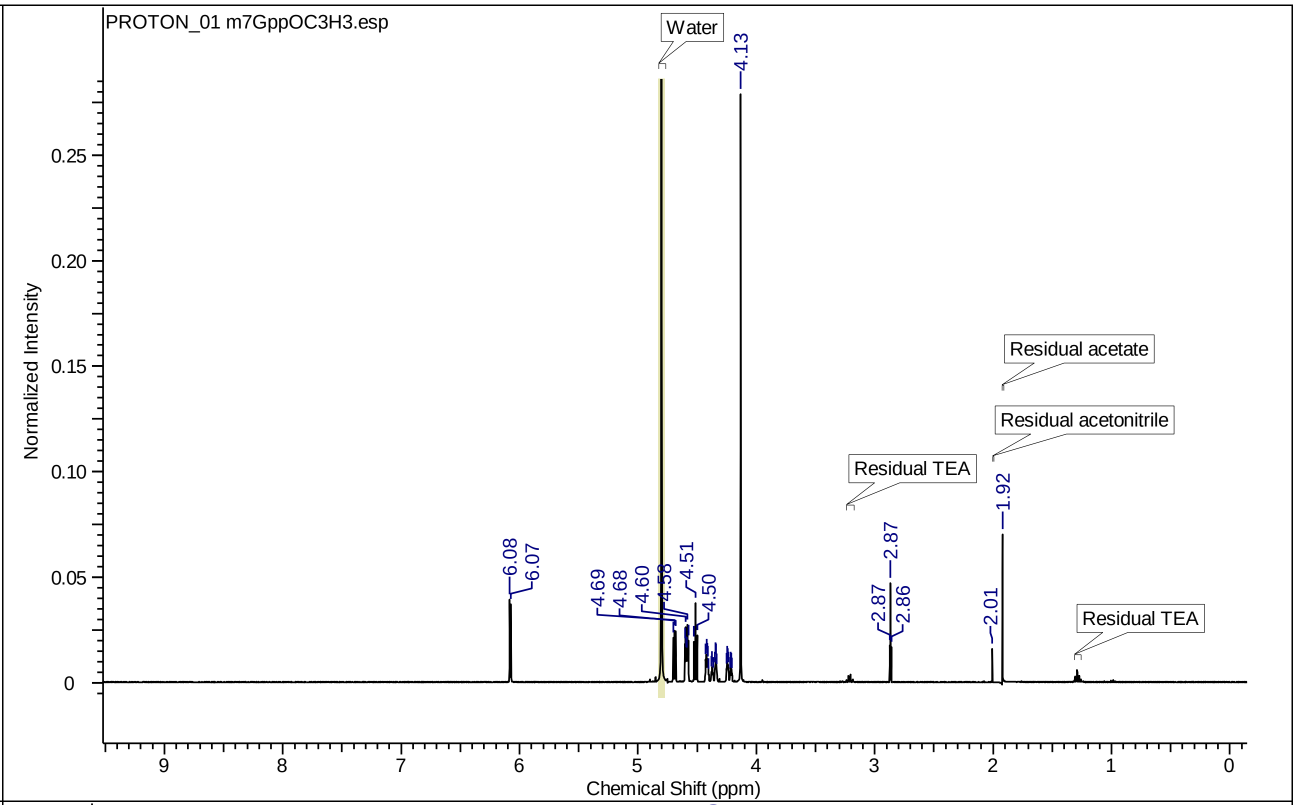

^{31}P NMR

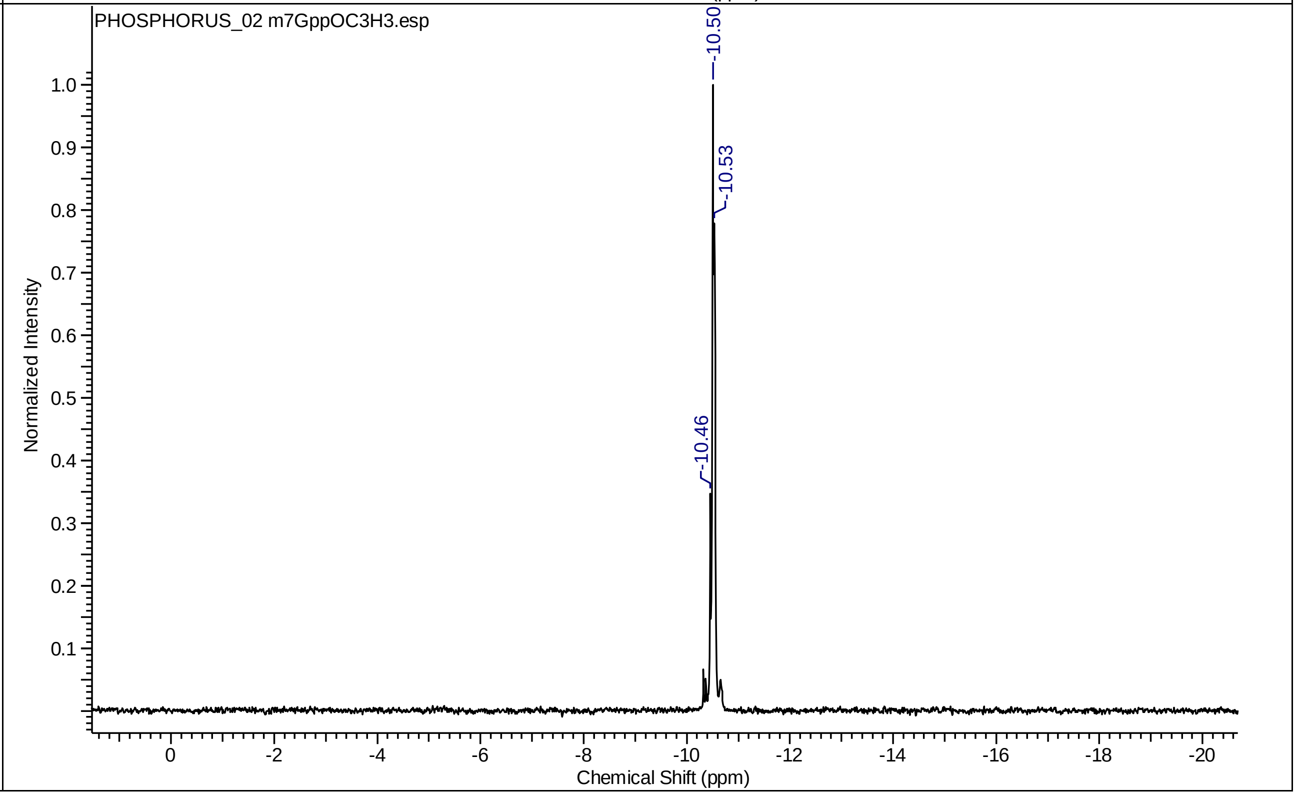

(13d) m^{7}GpppOC$_3$H$_3$

Structure

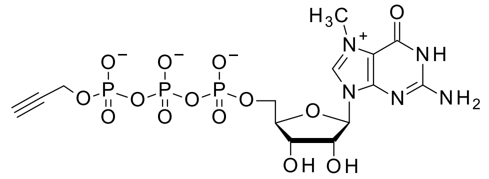

Reaction HPLC profile

Program A

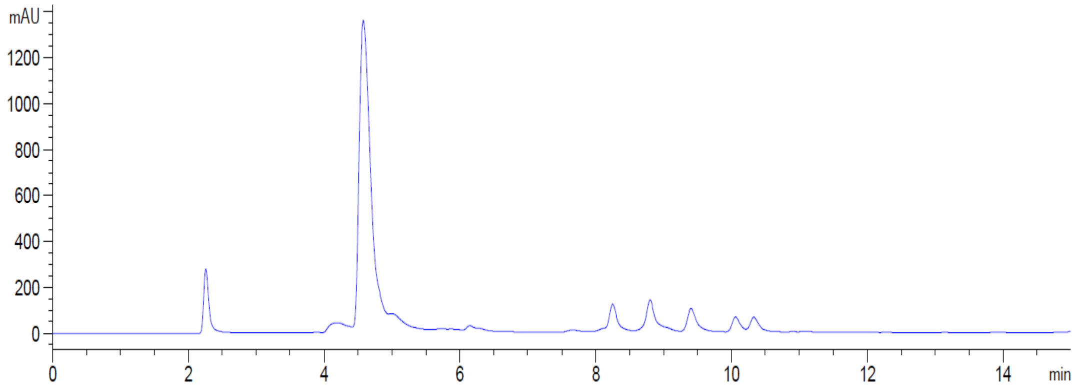

Purified compound HPLC profile

Program A

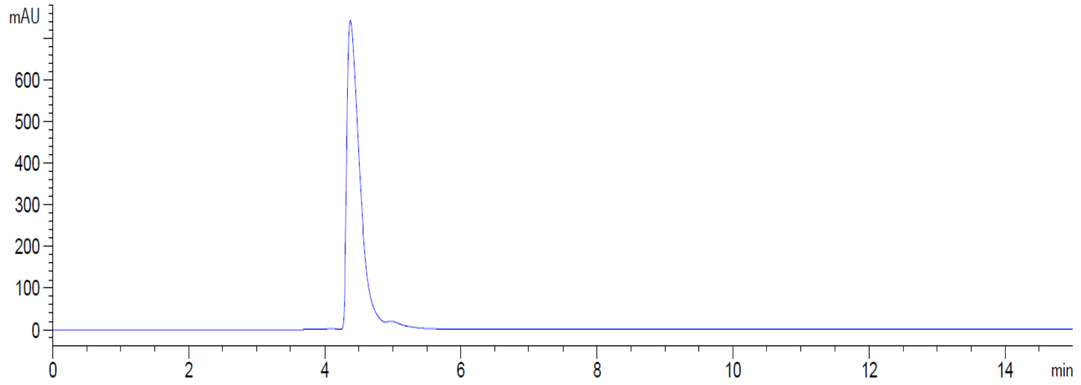

HRMS

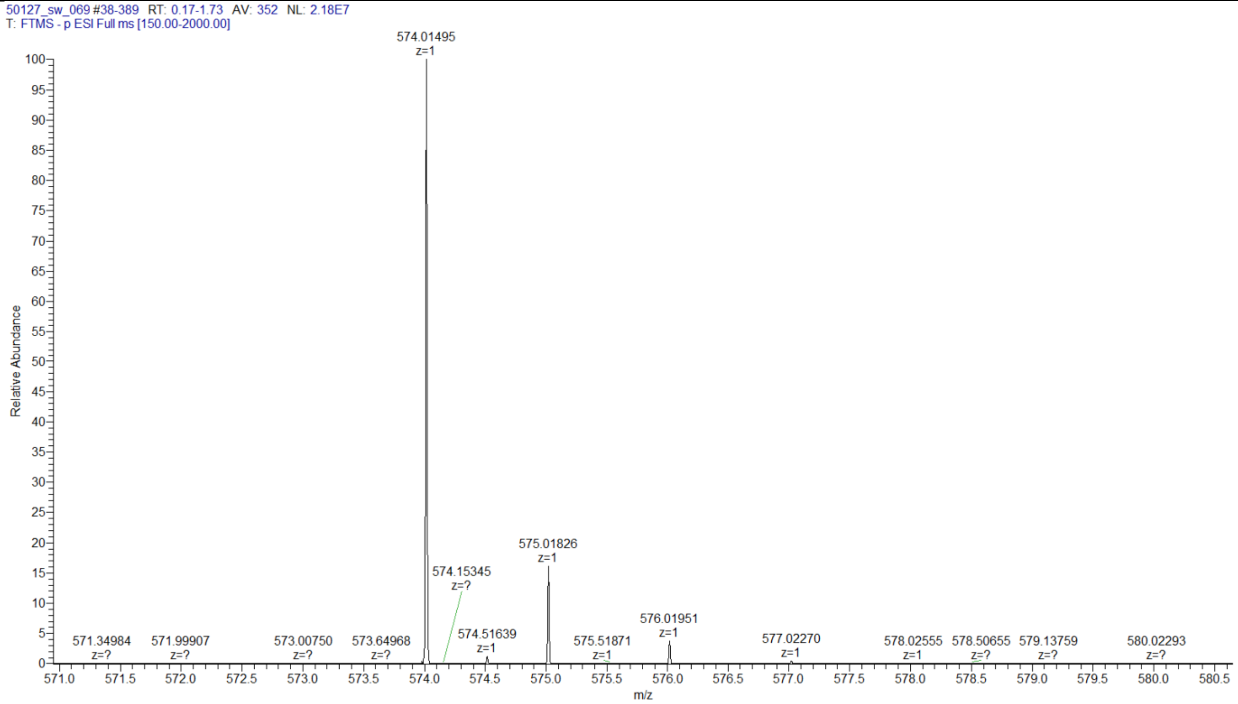

^{1}H NMR

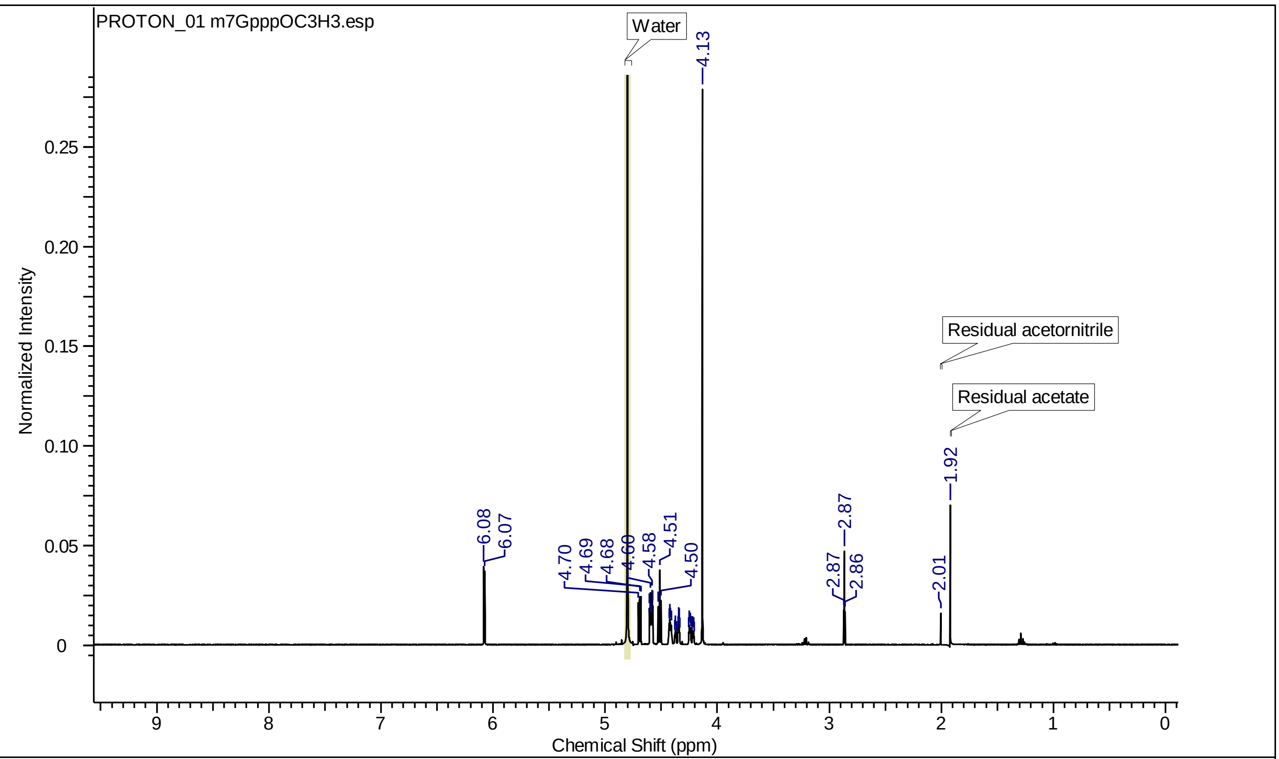

^{31}P NMR

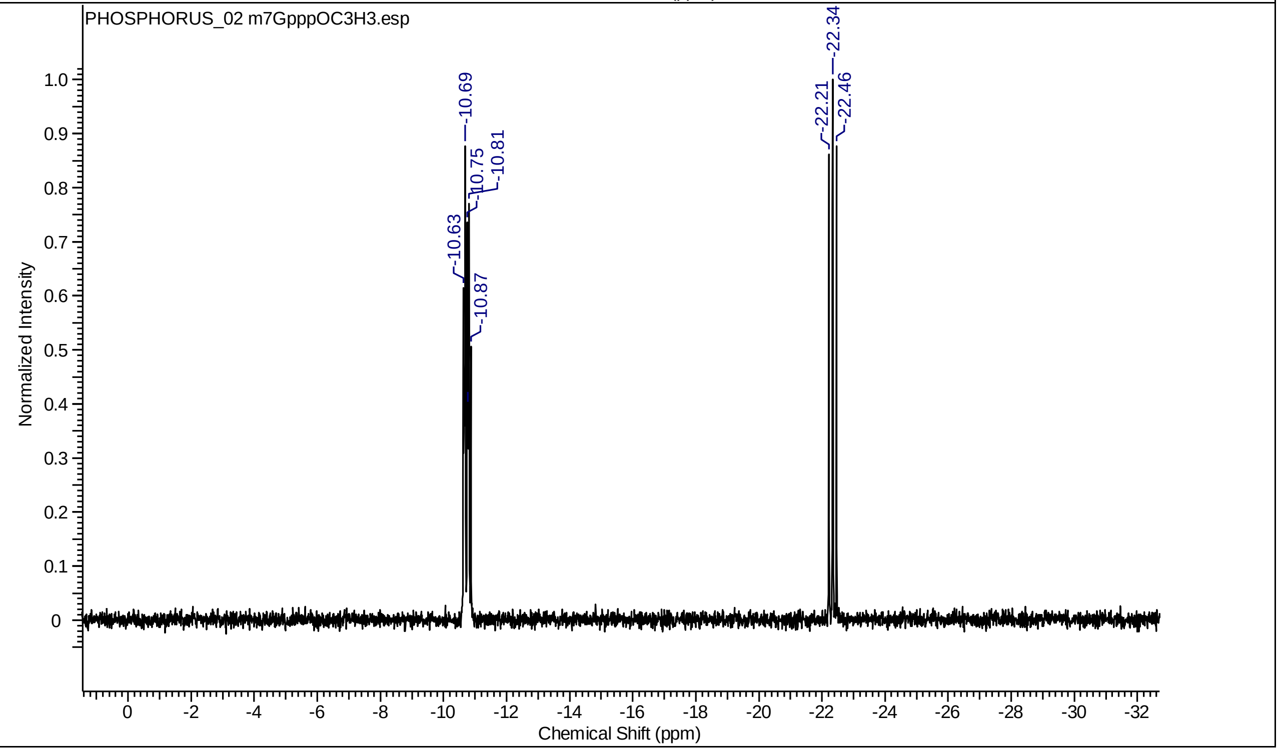

(14a) GppSC$_3$H$_3$

Structure

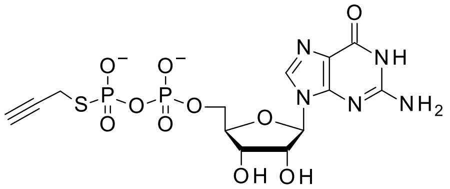

**Reaction HPLC profile**	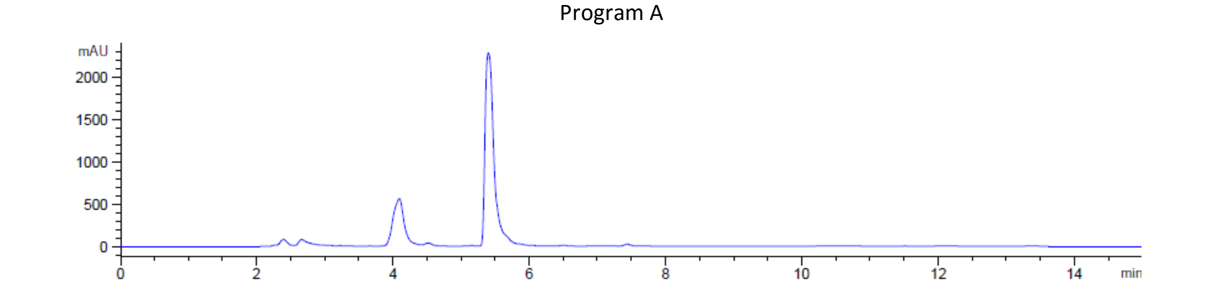
**Purified compound HPLC profile**	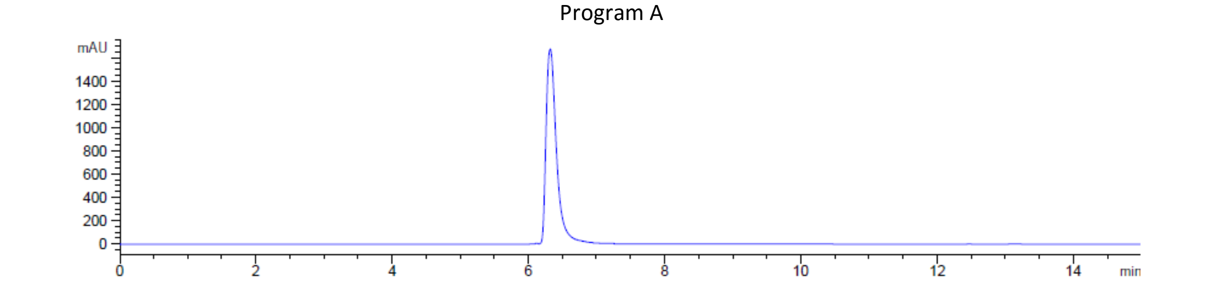
**HRMS**	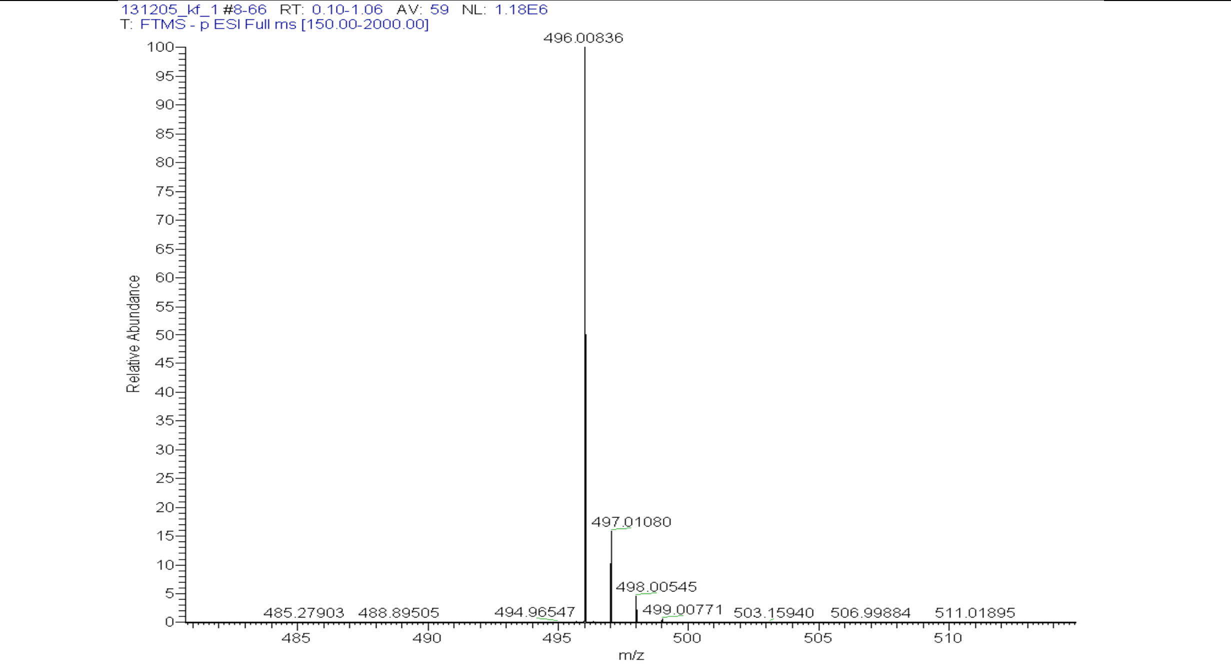
**^{1}H NMR**	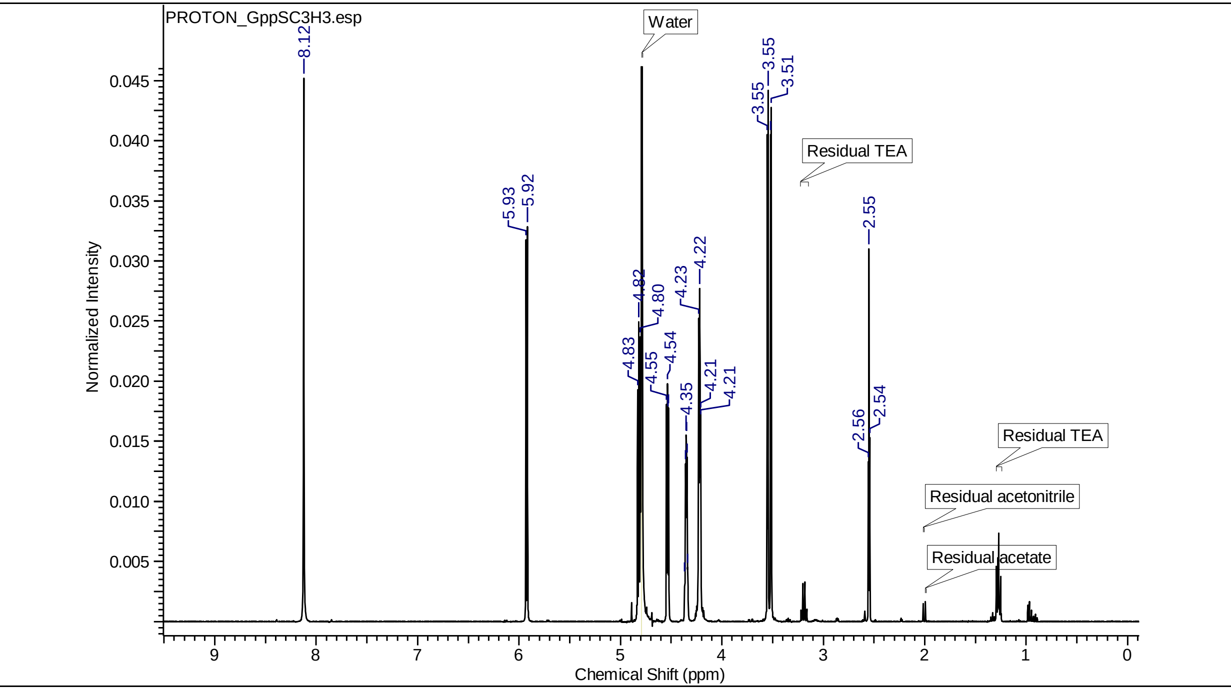

**^{31}P NMR**	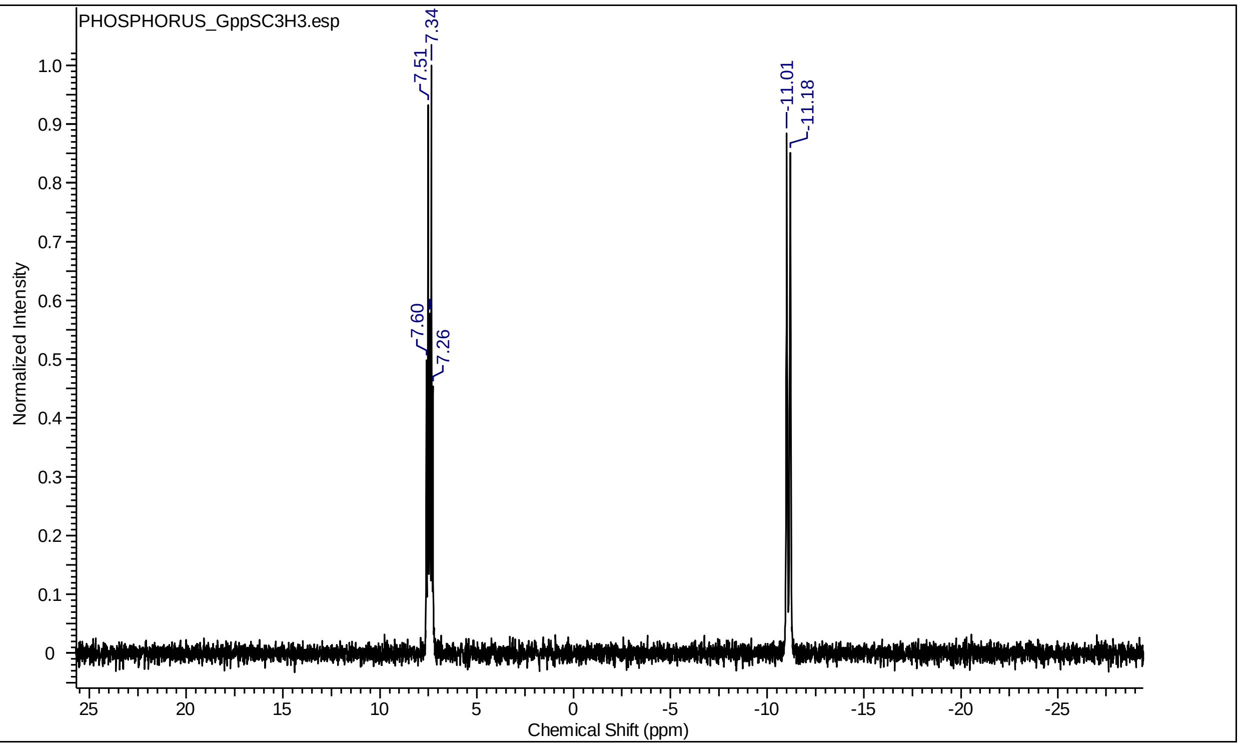

(14b) GpppSC$_3$H$_3$

**Structure**	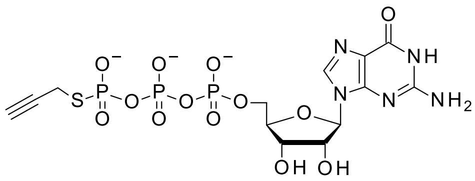
**Reaction HPLC profile**	Program A 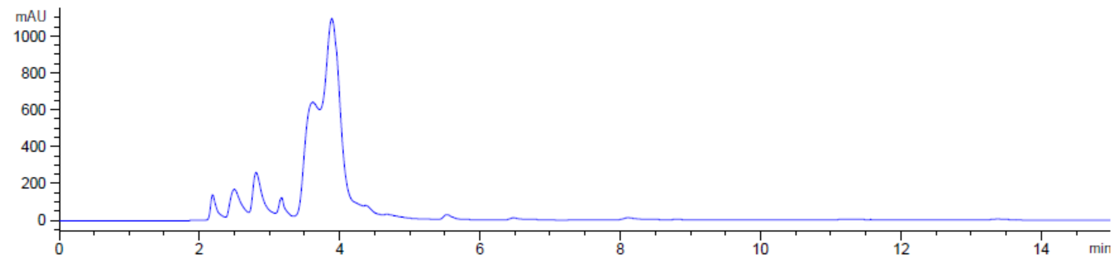
**Purified compound HPLC profile**	Program A 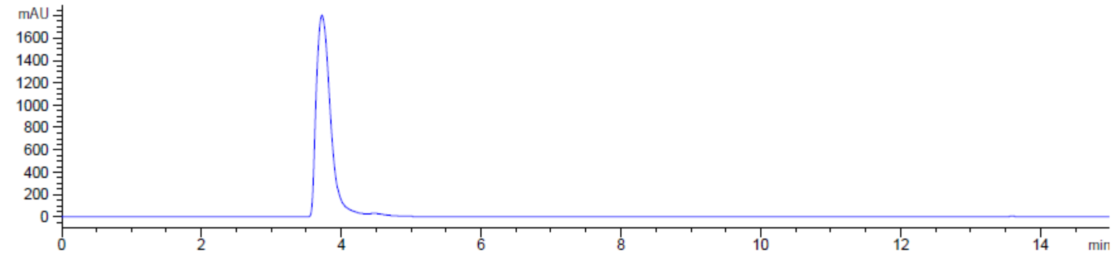

**HRMS**	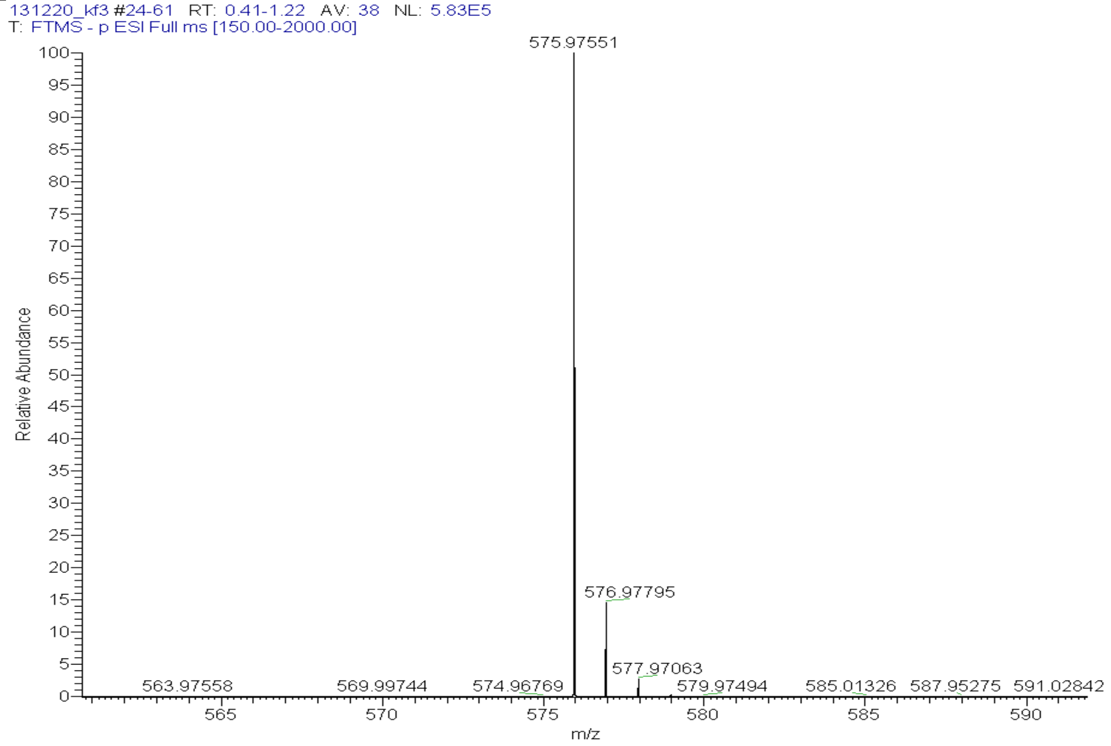
**^{1}H NMR**	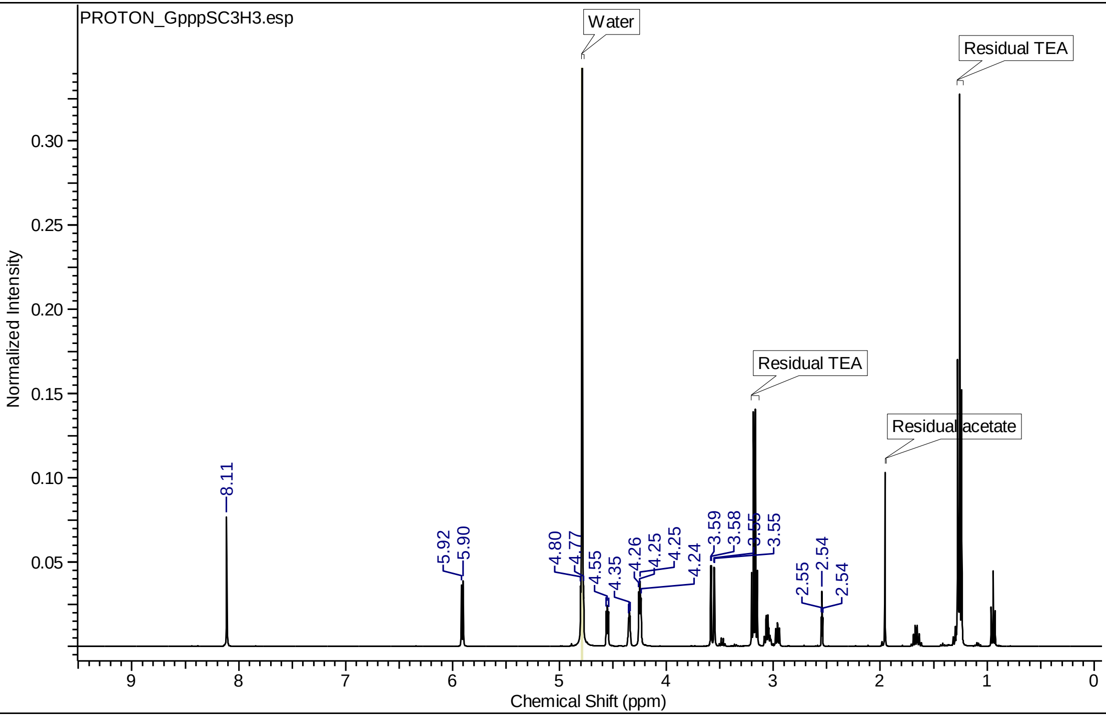

131220_kf3 #24-61 RT: 0.41-1.22 AV: 38 NL: 5.83E5
T: FTMS - p ESI Full ms [150.00-2000.00]
575.97551
576.97795
577.97063
563.97558
569.99744
574.96769
579.97494
585.01326
587.95275
591.02842
Relative Abundance
m/z

PROTON_GpppSC3H3.esp
Water
Residual TEA
Residual TEA
Residual acetate
8.11
5.92
5.90
4.80
4.77
4.55
4.35
4.26
4.25
4.25
4.24
3.59
3.58
3.55
3.55
2.55
2.54
2.54
Normalized Intensity
Chemical Shift (ppm)

**^{31}P NMR**	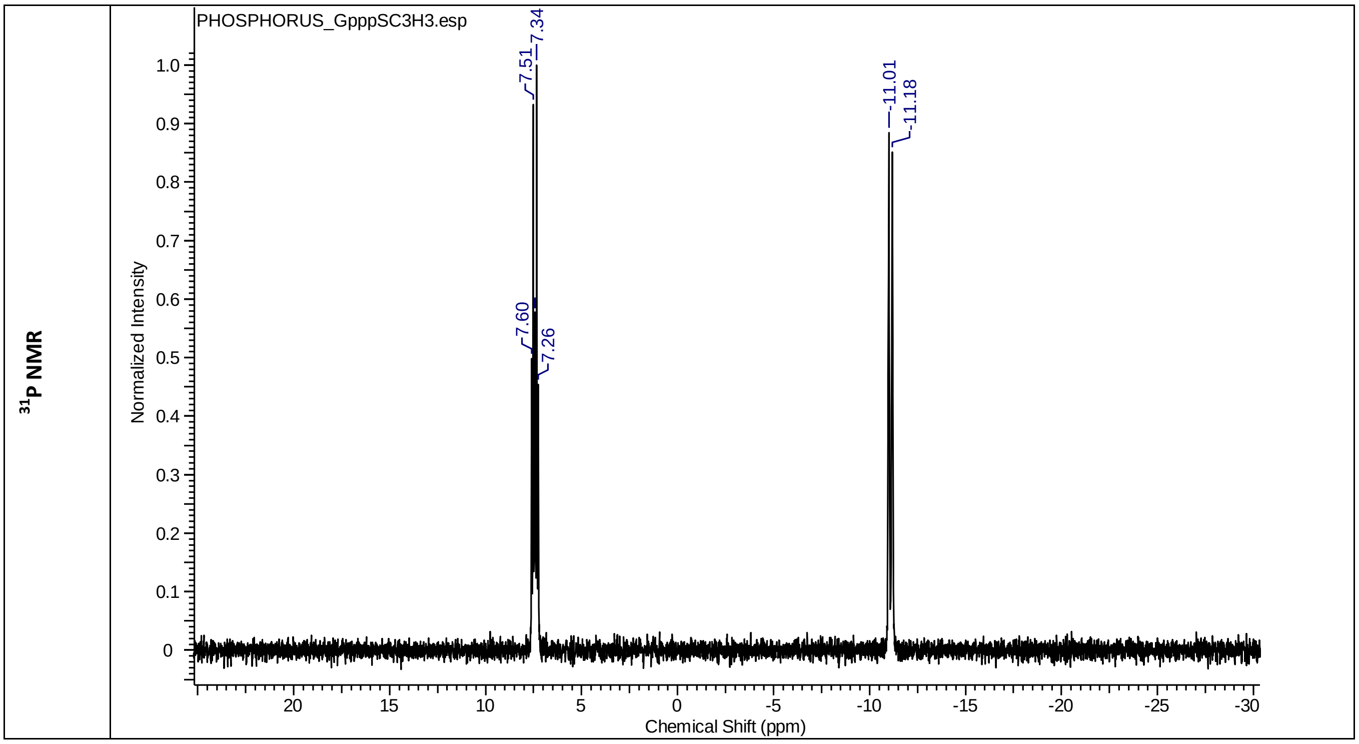

(14c) $m^7GppC_3H_3$

**Structure**	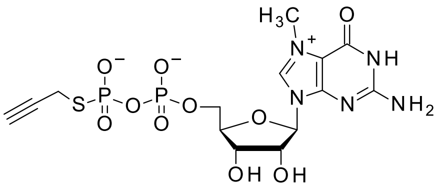
**Reaction HPLC profile**	Program A<br>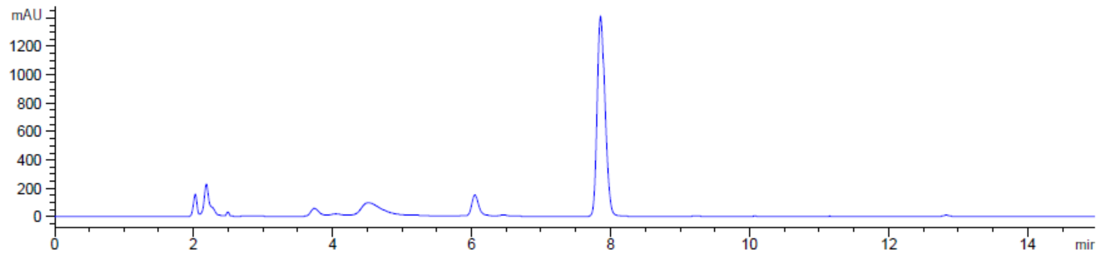
**Purified compound HPLC profile**	Program A<br>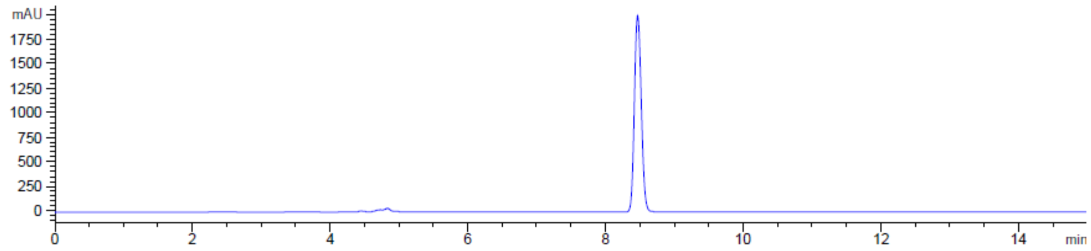

**HRMS**	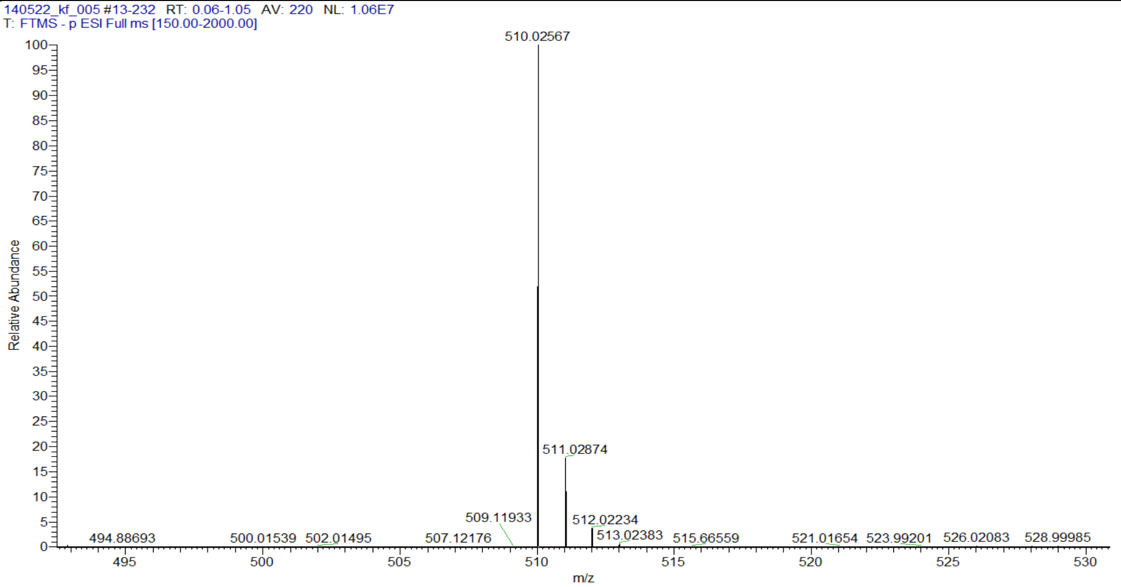
**^{1}H NMR**	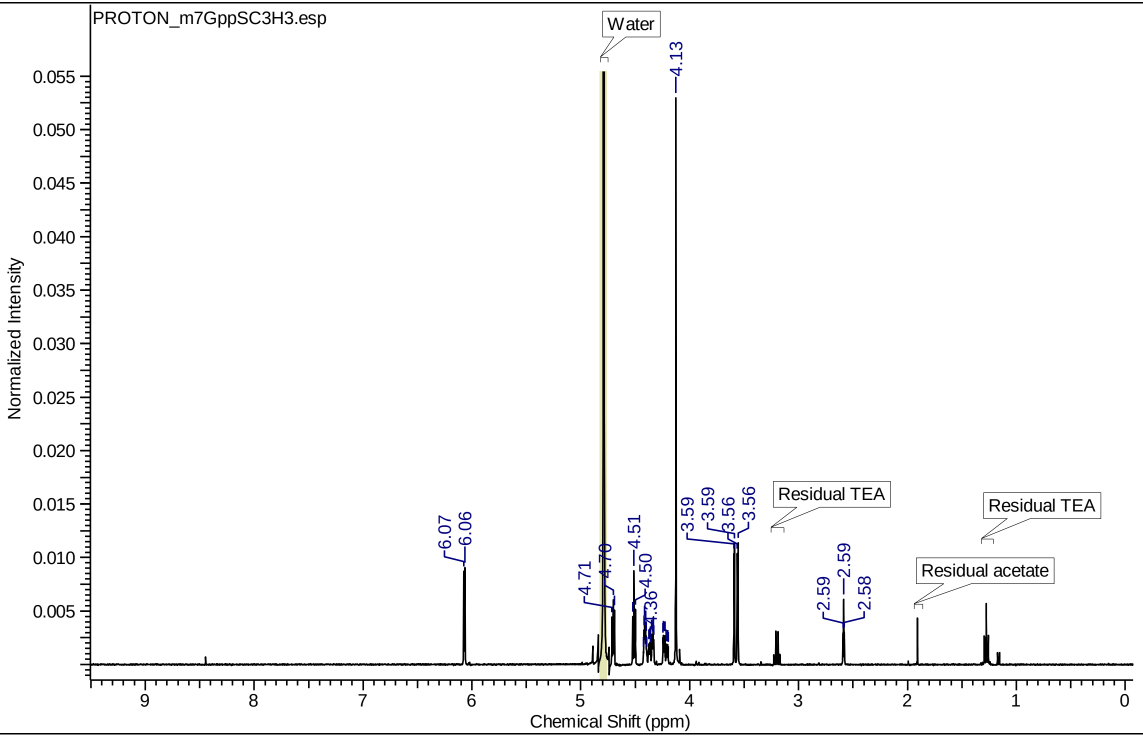

140522_kf_005 #13-232 RT: 0.06-1.05 AV: 220 NL: 1.06E7
T: FTMS - p ESI Full ms [150.00-2000.00]
510.02567
511.02874
509.11933
512.02234
513.02383
494.88693
500.01539
502.01495
507.12176
515.66559
521.01654
523.99201
526.02083
528.99985
Relative Abundance
m/z

PROTON_m7GppSC3H3.esp
Water
Residual TEA
Residual TEA
Residual acetate
6.07
6.06
4.71
4.70
4.51
4.50
4.36
4.13
3.59
3.59
3.56
3.56
2.59
2.59
2.58
Normalized Intensity
Chemical Shift (ppm)

^{31}P NMR	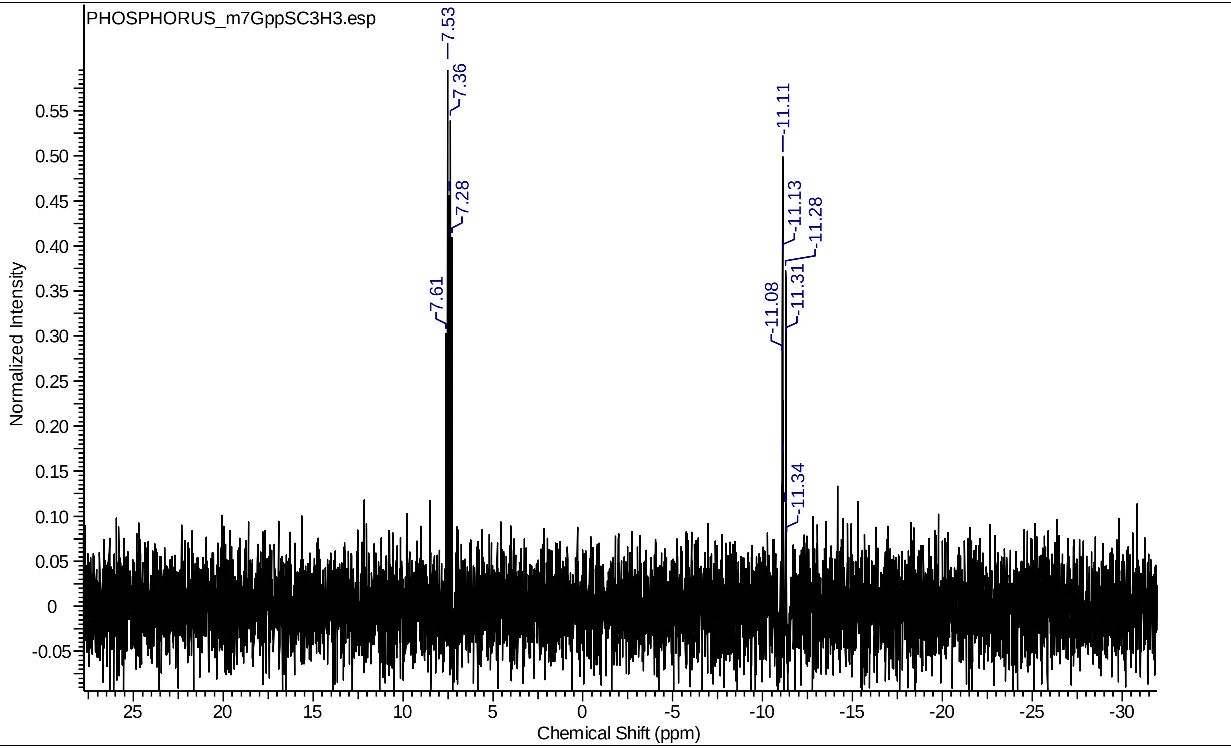

(14d) m^{7}GpppSC$_3$H$_3$

Structure	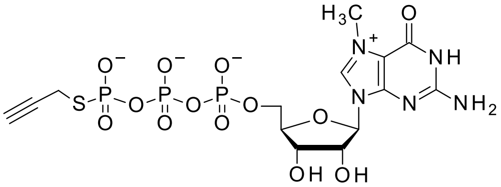
Reaction HPLC profile	Program A 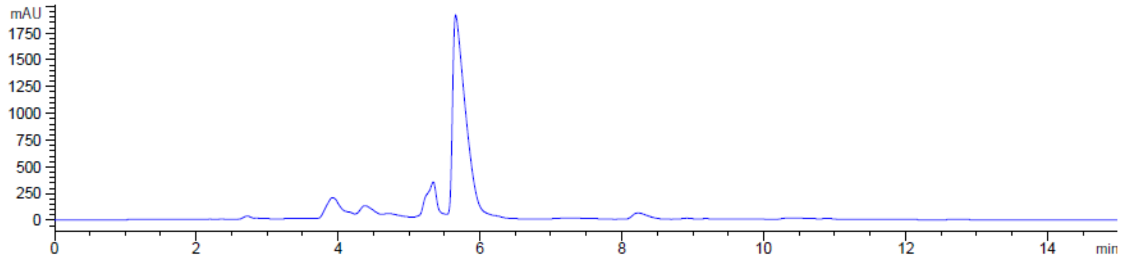
Purified compound HPLC profile	Program A 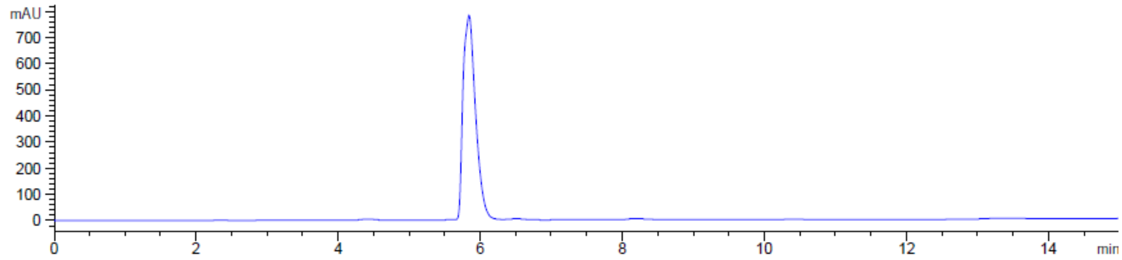

**HRMS**	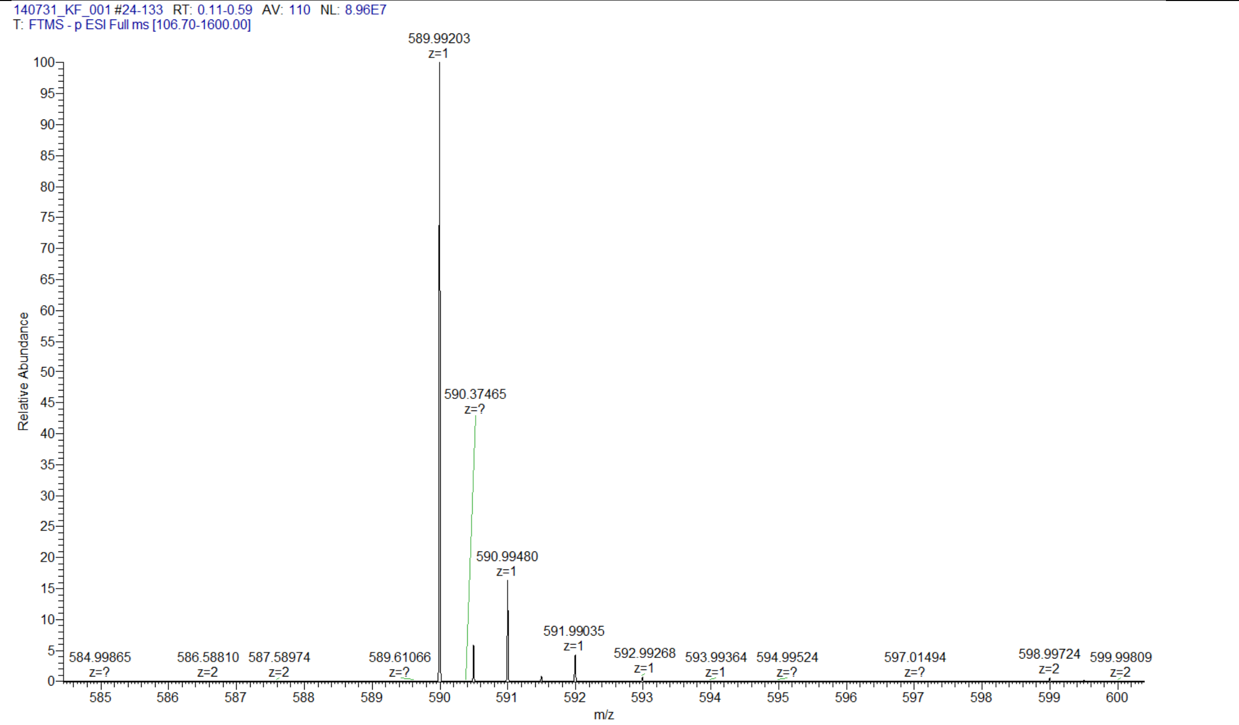
**^{1}H NMR**	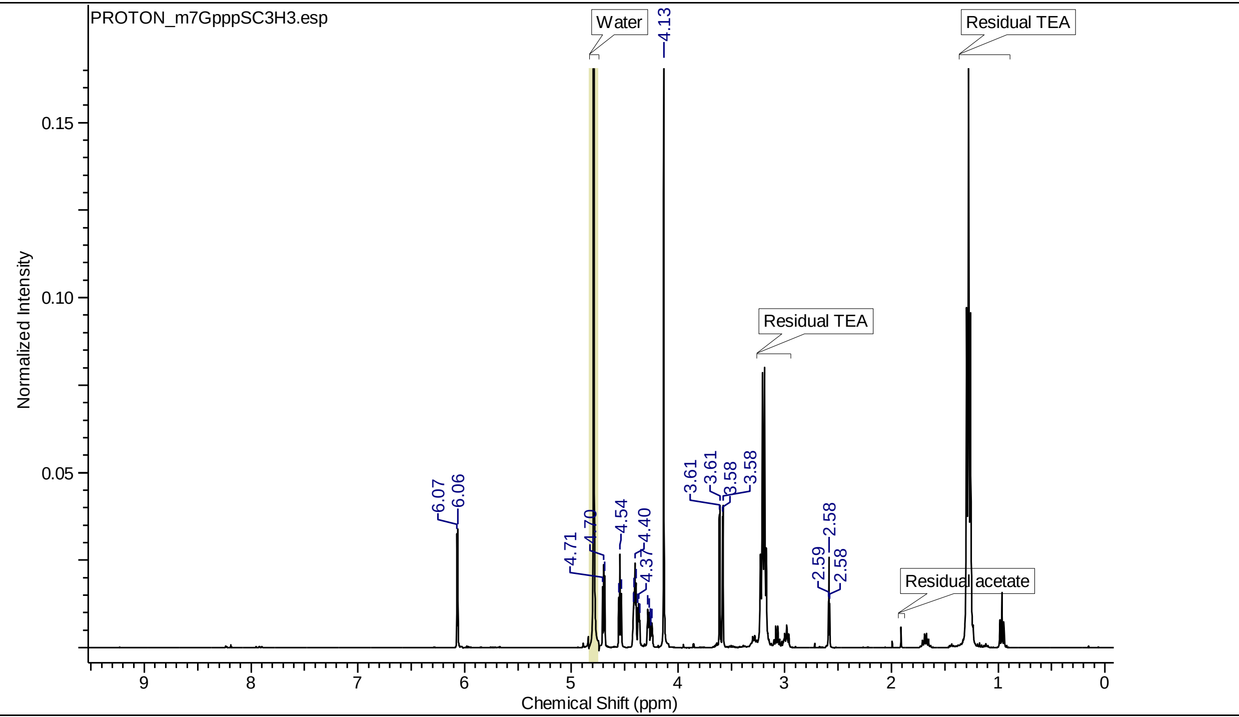

140731_KF_001 #24-133 RT: 0.11-0.59 AV: 110 NL: 8.96E7
T: FTMS - p ESI Full ms [106.70-1600.00]
589.99203 z=1
590.37465 z=?
590.99480 z=1
591.99035 z=1
584.99865 z=?
586.58810 z=2
587.58974 z=2
589.61066 z=?
592.99268 z=1
593.99364 z=1
594.99524 z=?
597.01494 z=?
598.99724 z=2
599.99809 z=2
Relative Abundance
m/z

PROTON_m7GpppSC3H3.esp
Water
Residual TEA
Residual TEA
Residual acetate
6.07
6.06
4.71
4.70
4.54
4.40
4.37
4.13
3.61
3.61
3.58
3.58
2.59
2.58
2.58
Normalized Intensity
Chemical Shift (ppm)

^{31}P NMR

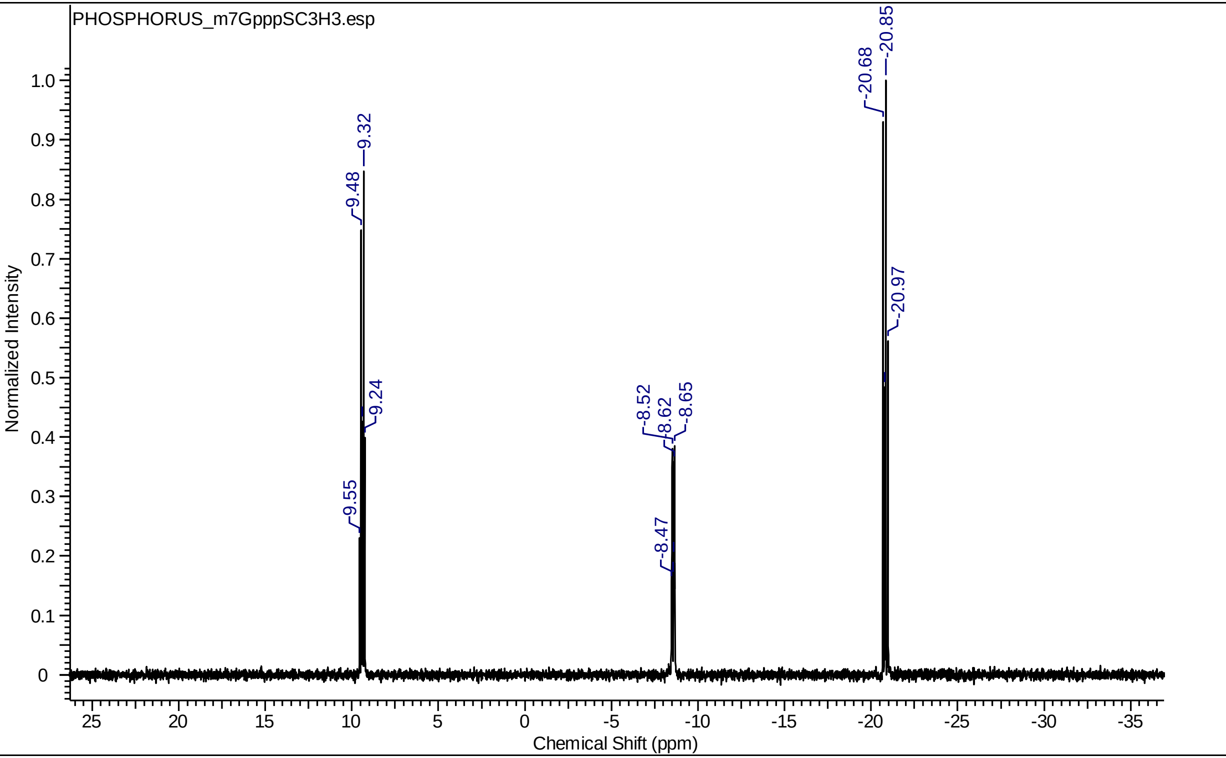

(15a) GppNHC$_3$H$_3$

Structure

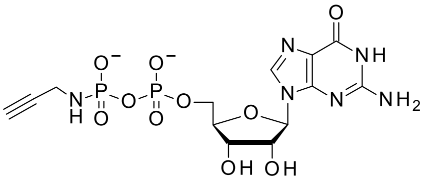

Reaction HPLC profile

Program A

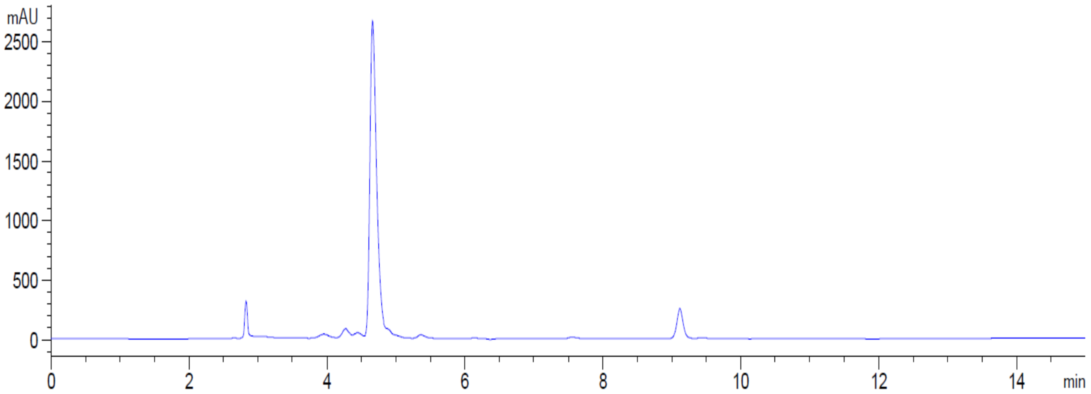

Purified compound HPLC profile

Program A

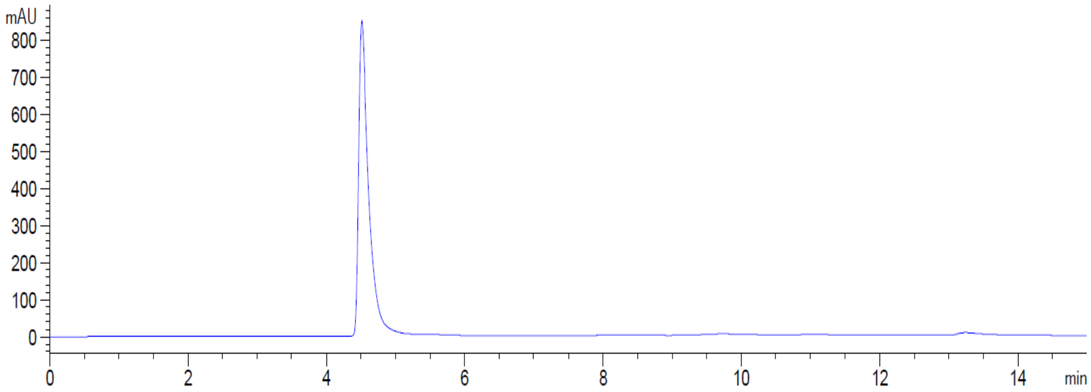

HRMS

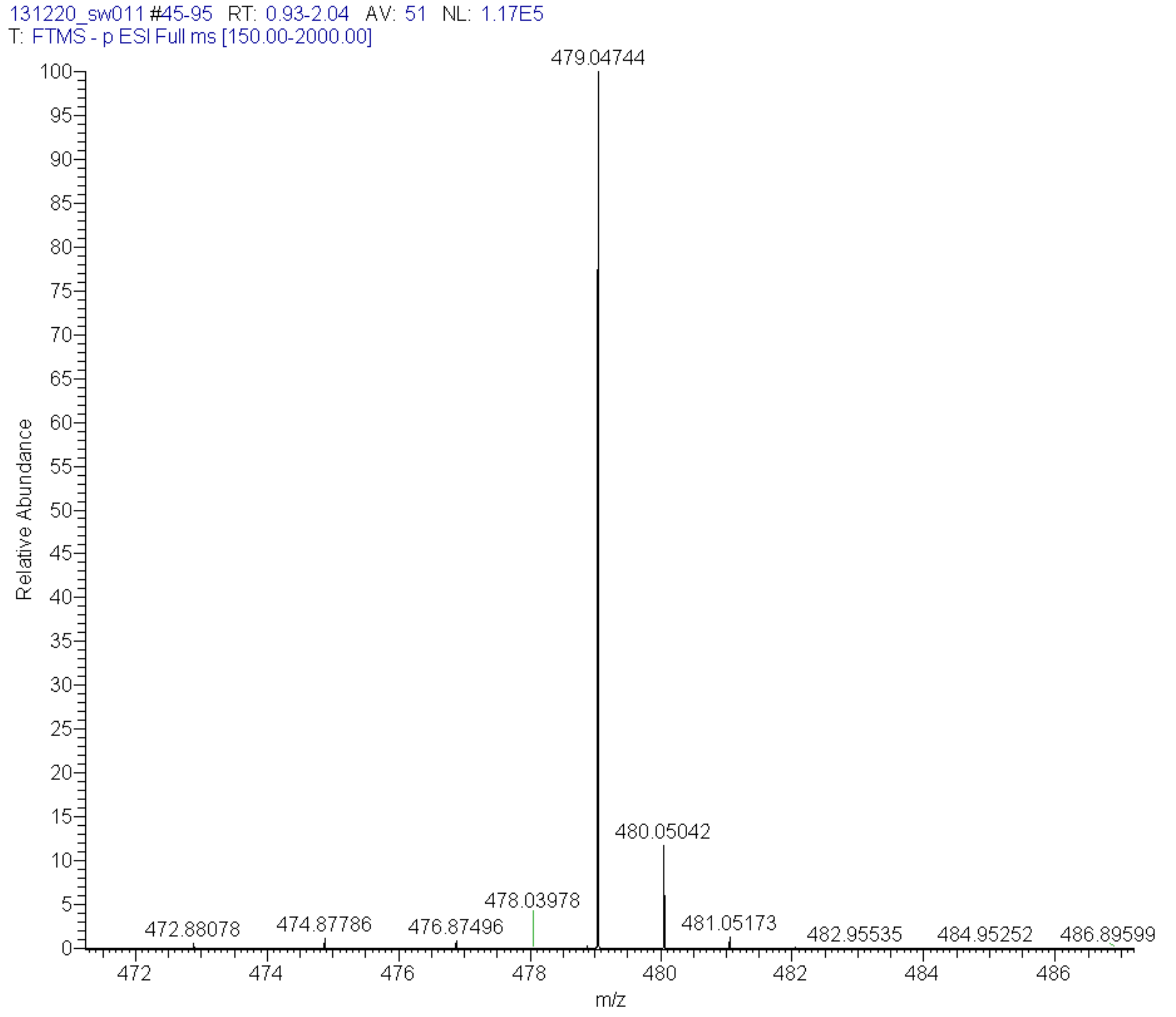
131220_sw011 #45-95 RT: 0.93-2.04 AV: 51 NL: 1.17E5
T: FTMS - p ESI Full ms [150.00-2000.00]
479.04744
480.05042
478.03978
472.88078
474.87786
476.87496
481.05173
482.95535
484.95252
486.89599
Relative Abundance
m/z

^{1}H NMR

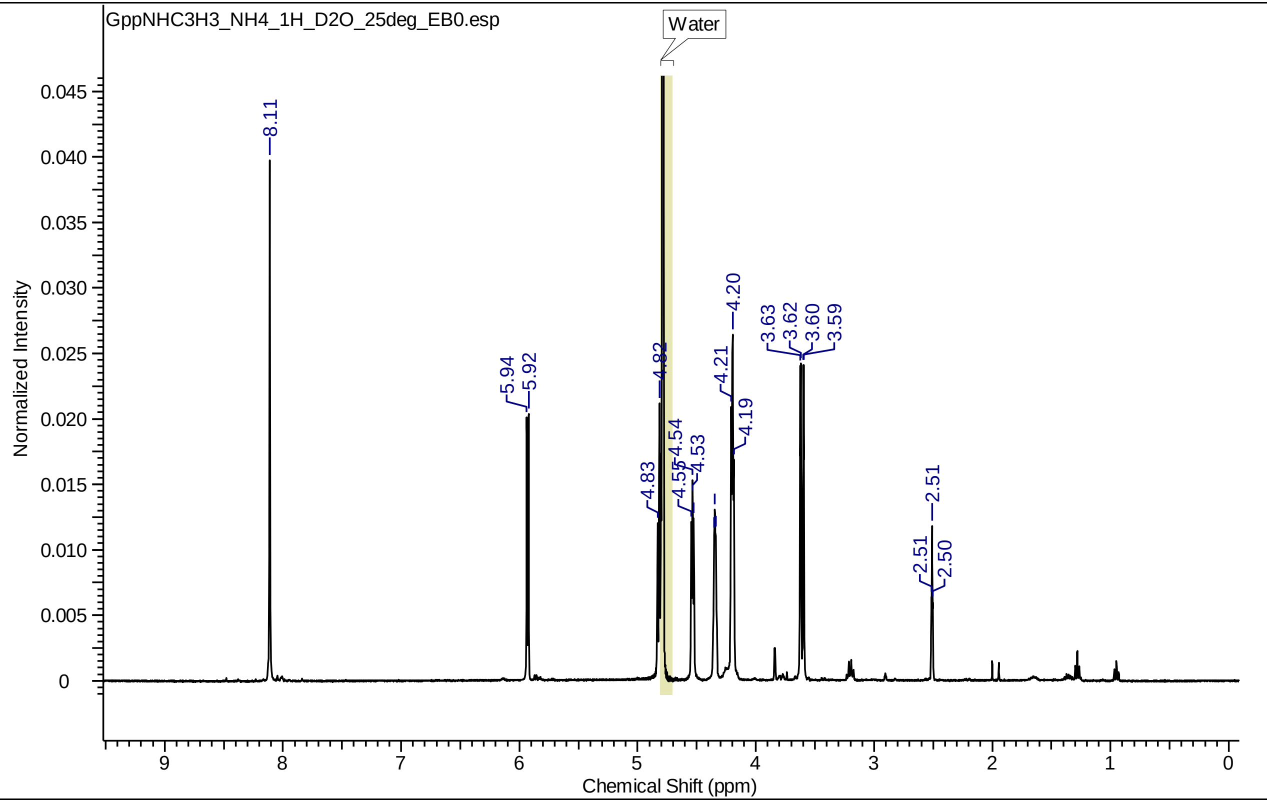
GppNHC3H3_NH4_1H_D2O_25deg_EB0.esp
Water
8.11
5.94
5.92
4.83
4.82
4.55
4.54
4.53
4.21
4.20
4.19
3.63
3.62
3.60
3.59
2.51
2.51
2.50
Normalized Intensity
Chemical Shift (ppm)

^{31}P NMR	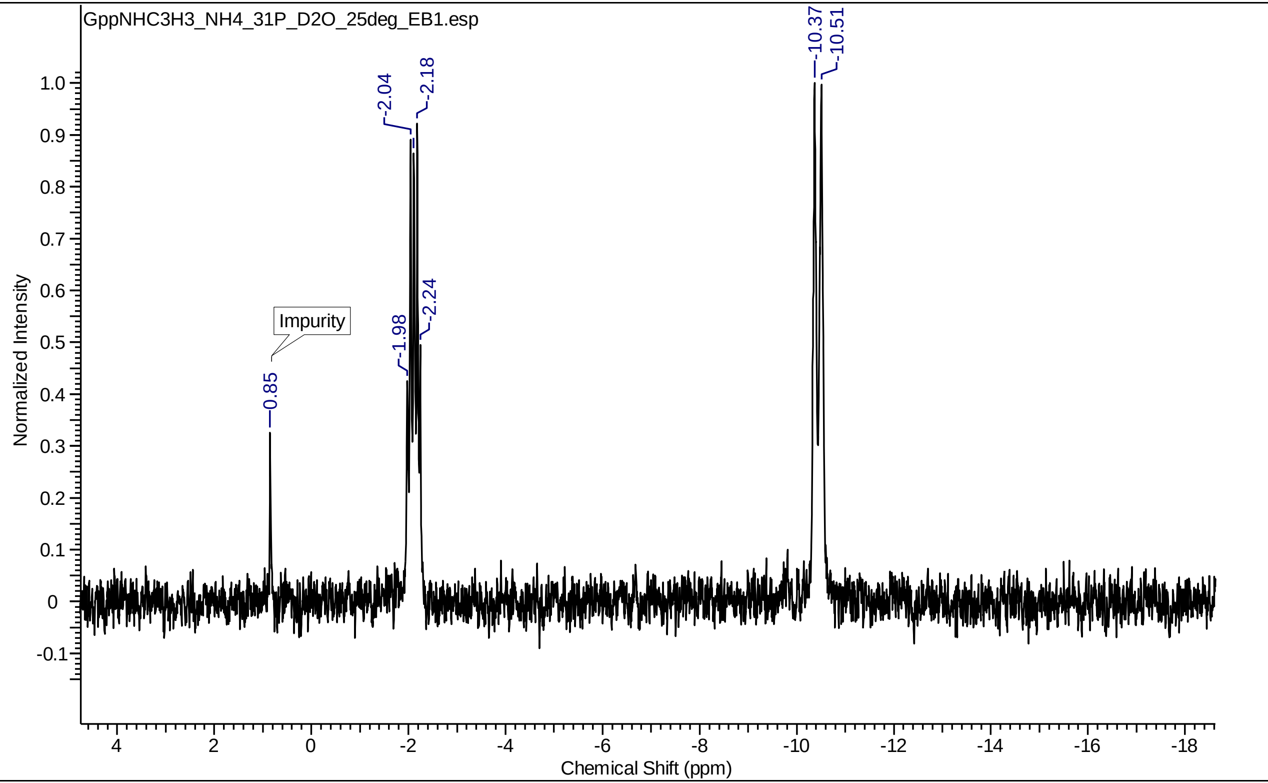

(15b) GpppNHC$_3$H$_3$

Structure	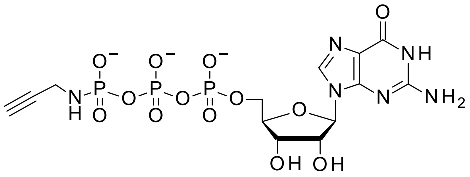
Reaction HPLC profile	Program A 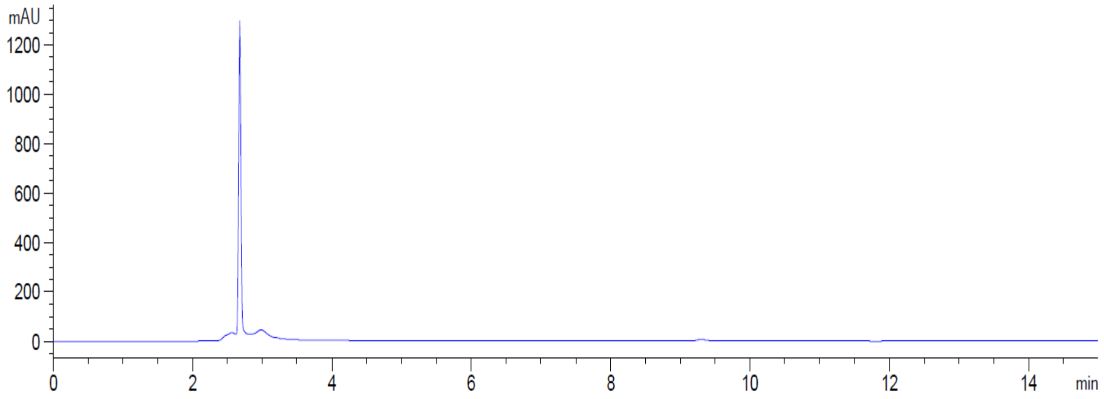

Purified compound HPLC profile

Program A

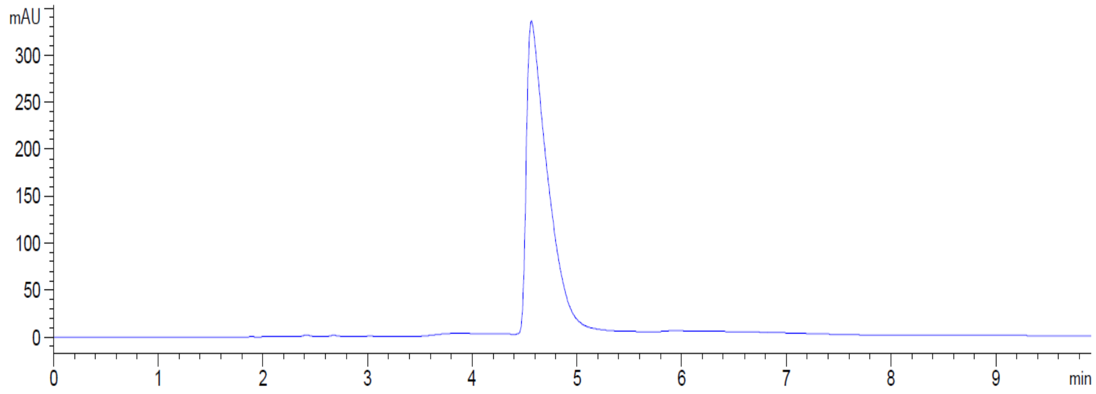

HRMS

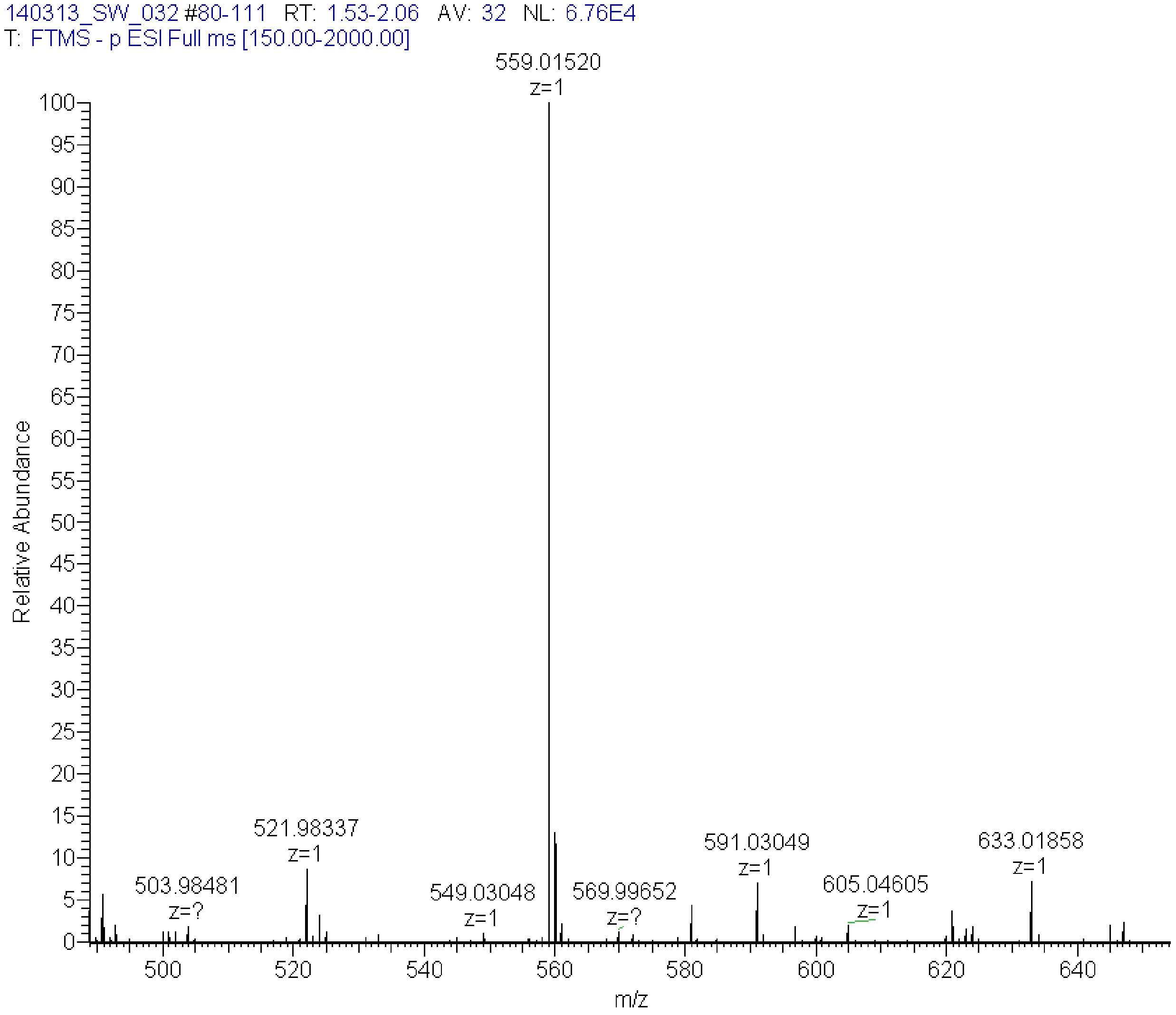

^{1}H NMR

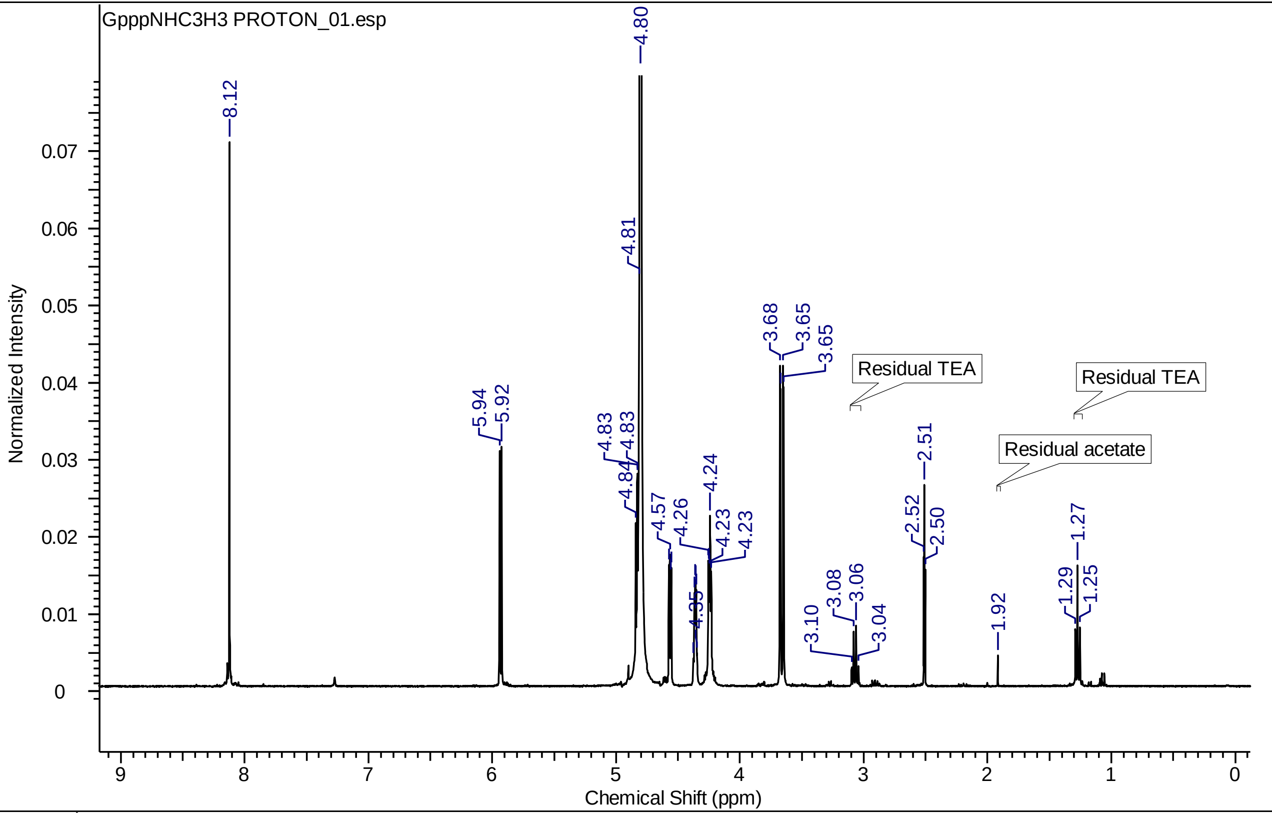

^{31}P NMR

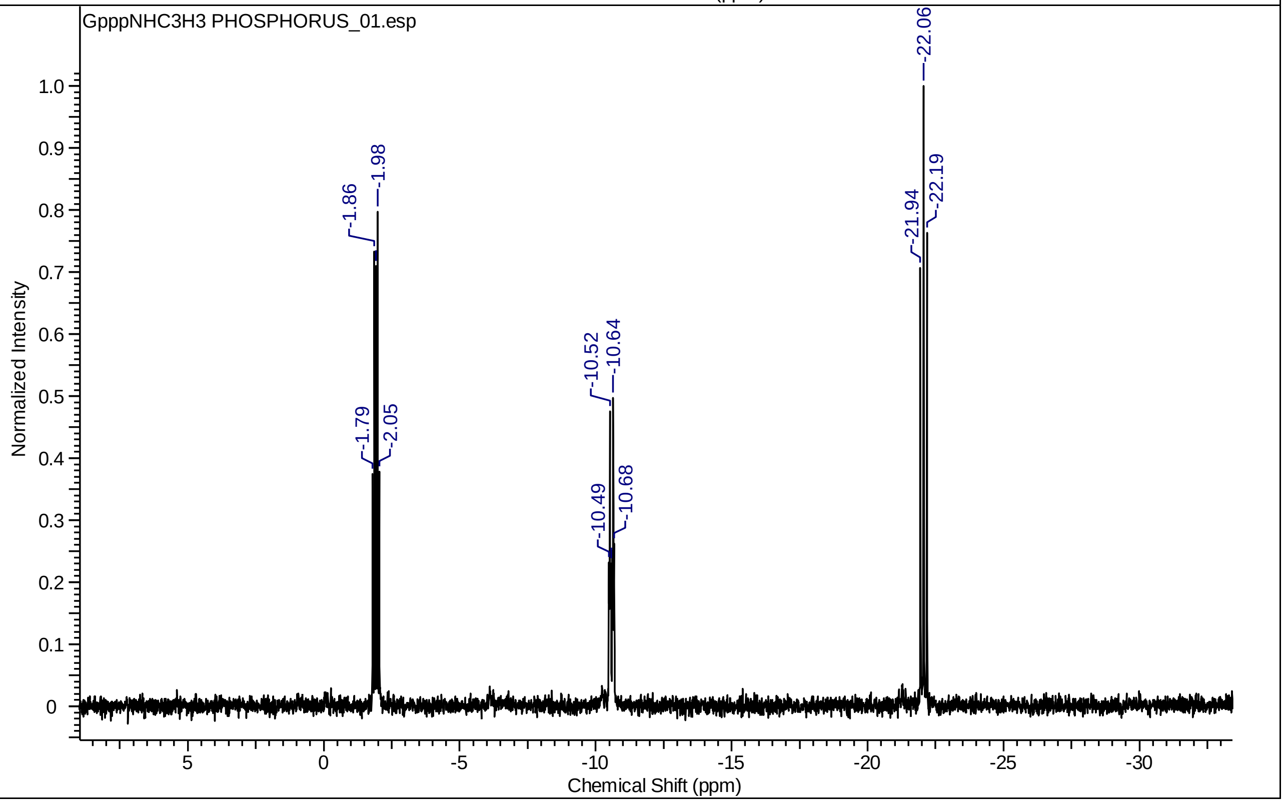

(15c) m^{7}GppNHC$_3$H$_3$

Structure

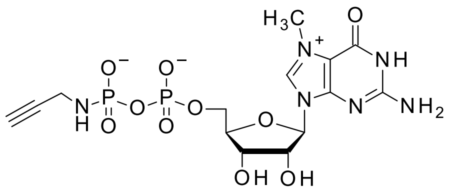

**Reaction HPLC profile**	Program A<br>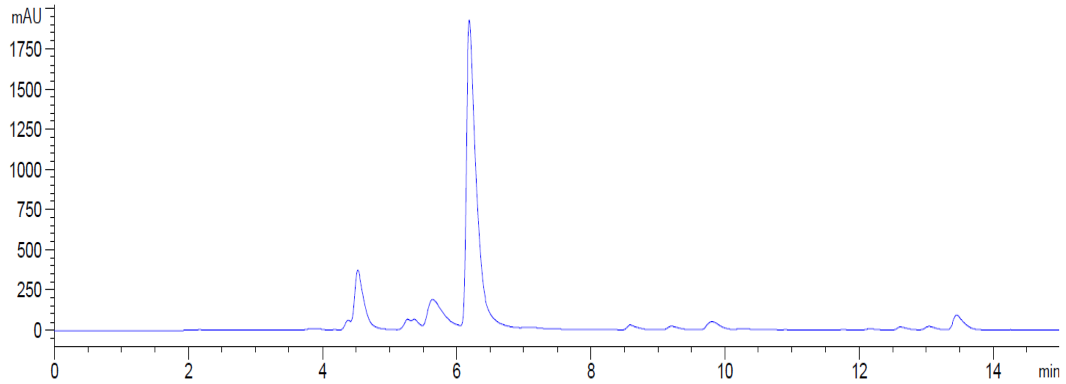
**Purified compound HPLC profile**	Program A<br>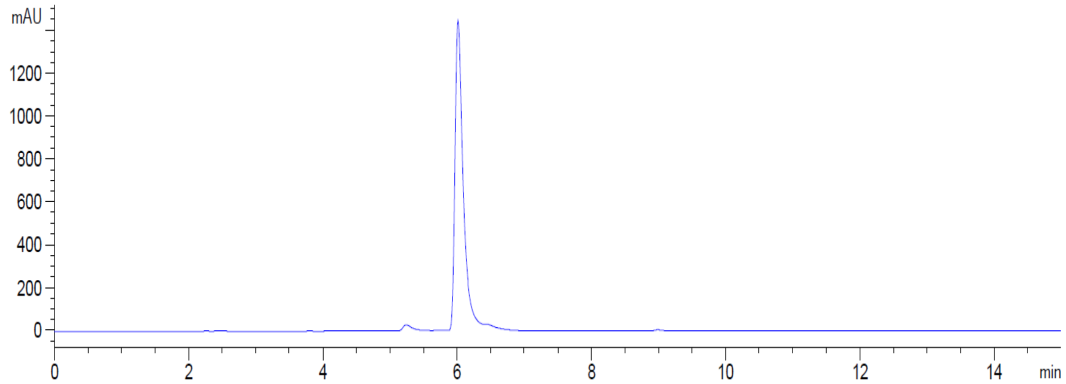
**HRMS**	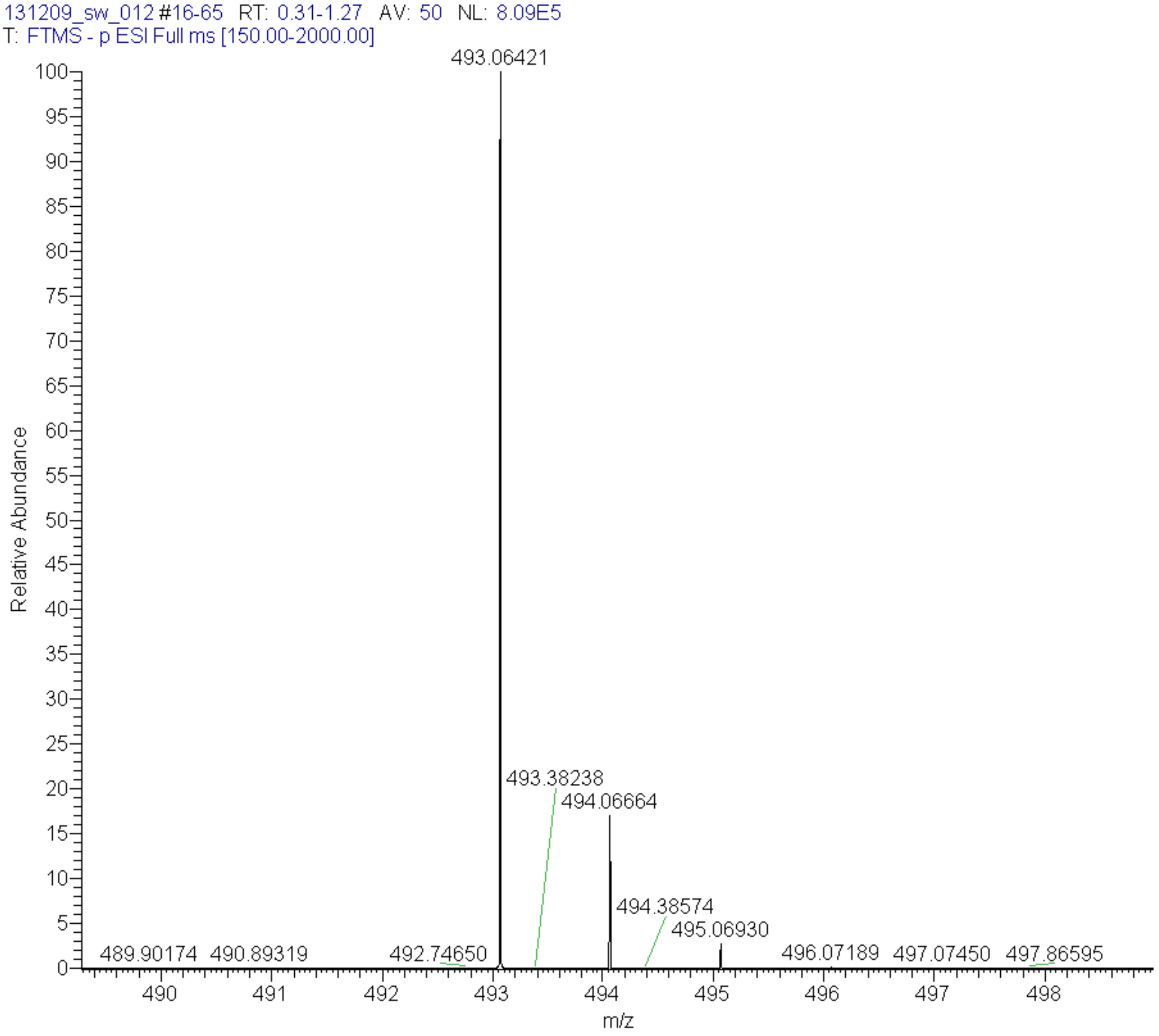

**^{1}H NMR**	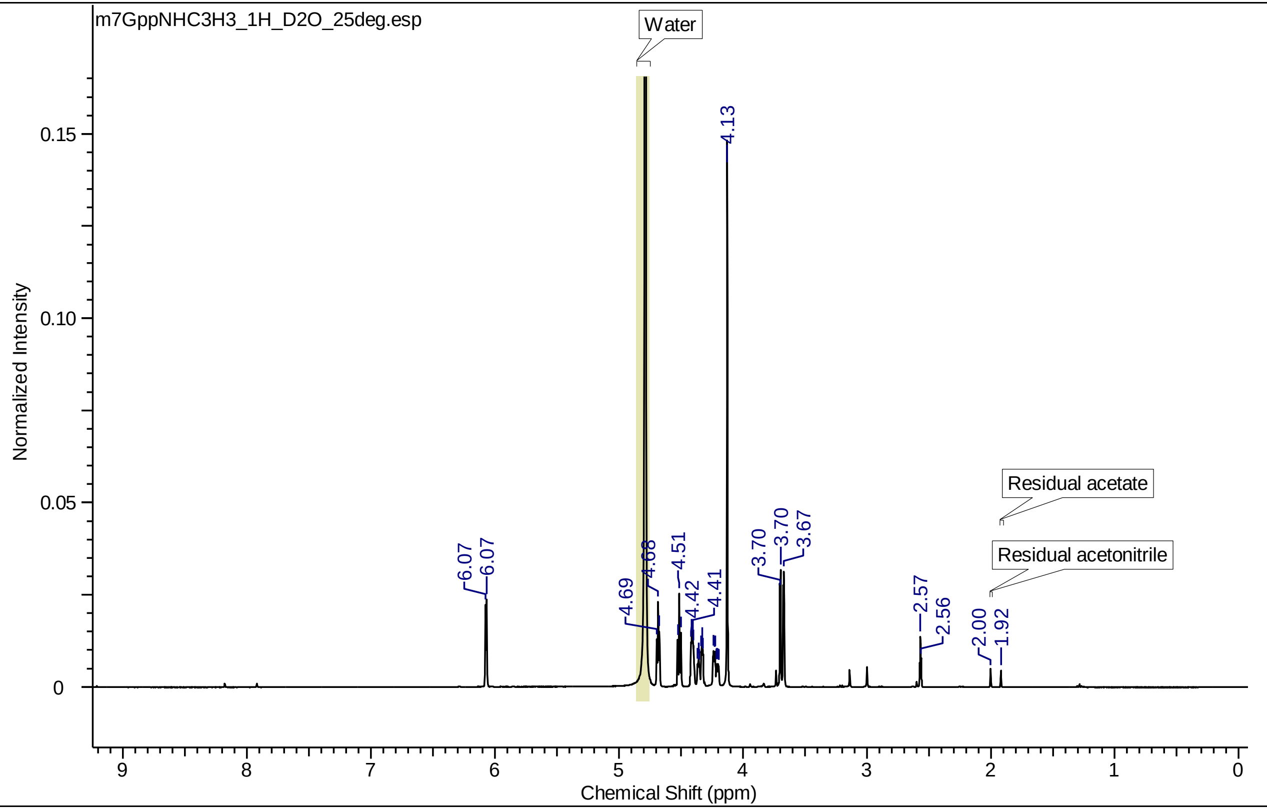
**^{31}P NMR**	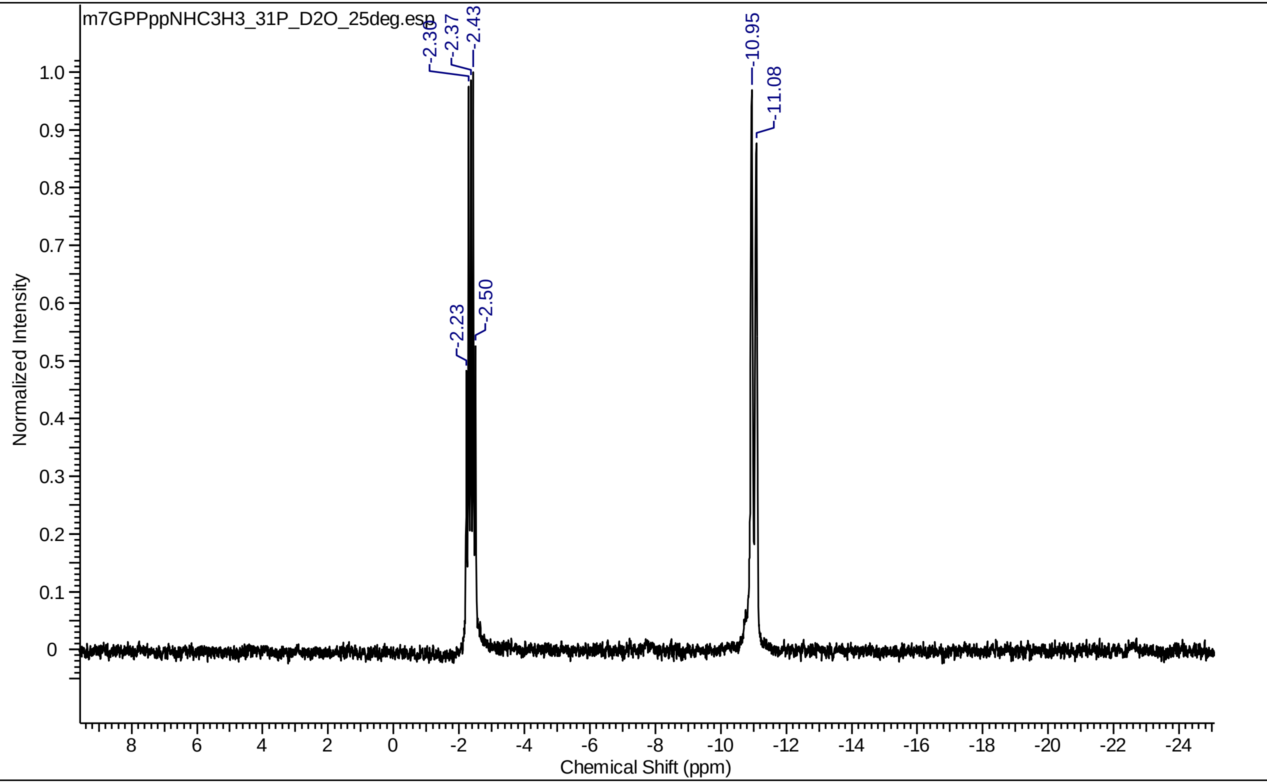

(15d) $m^{7}GpppNHC_3H_3$

**Structure**	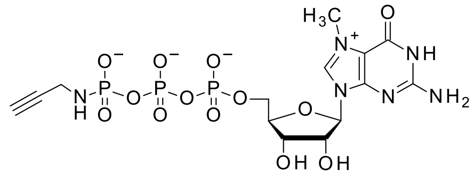

Reaction HPLC profile

Program A

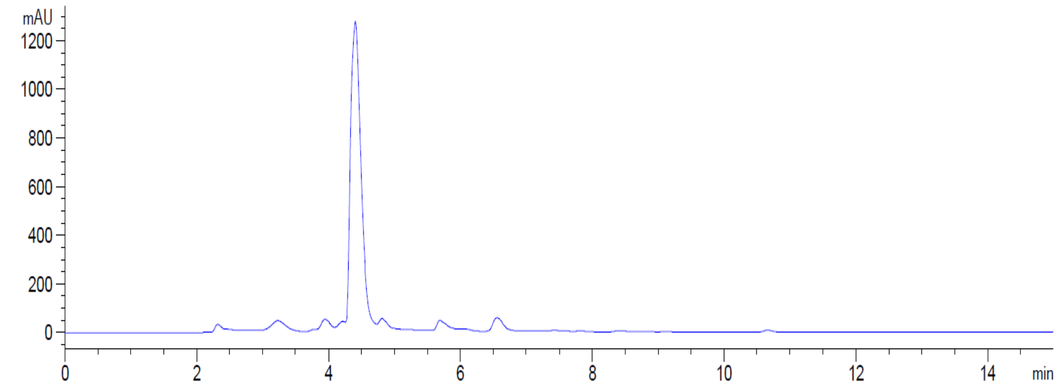

Purified compound HPLC profile

Program A

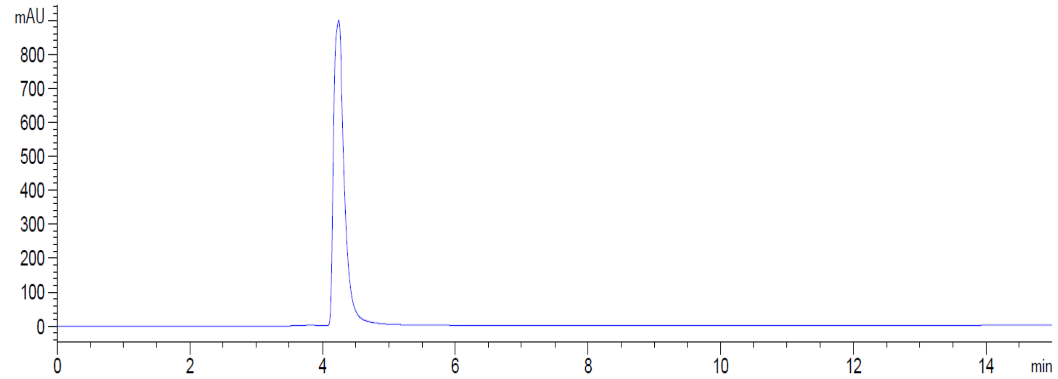

HRMS

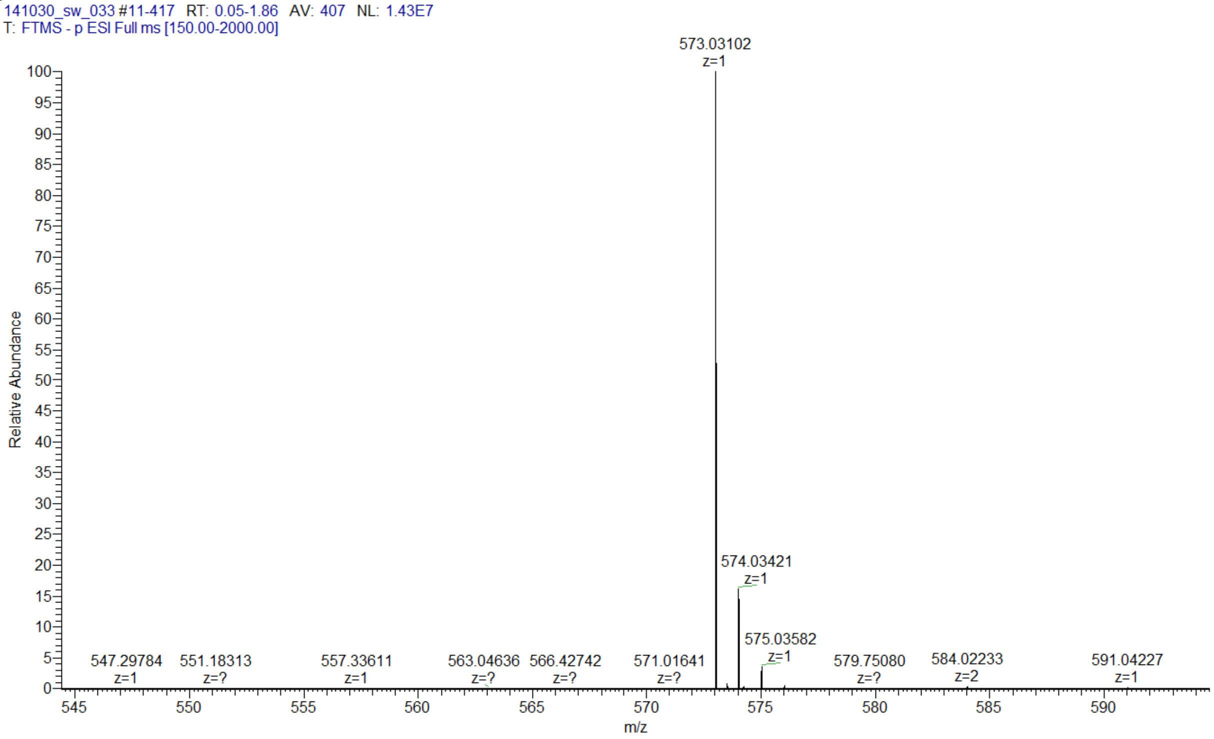

^{1}H NMR	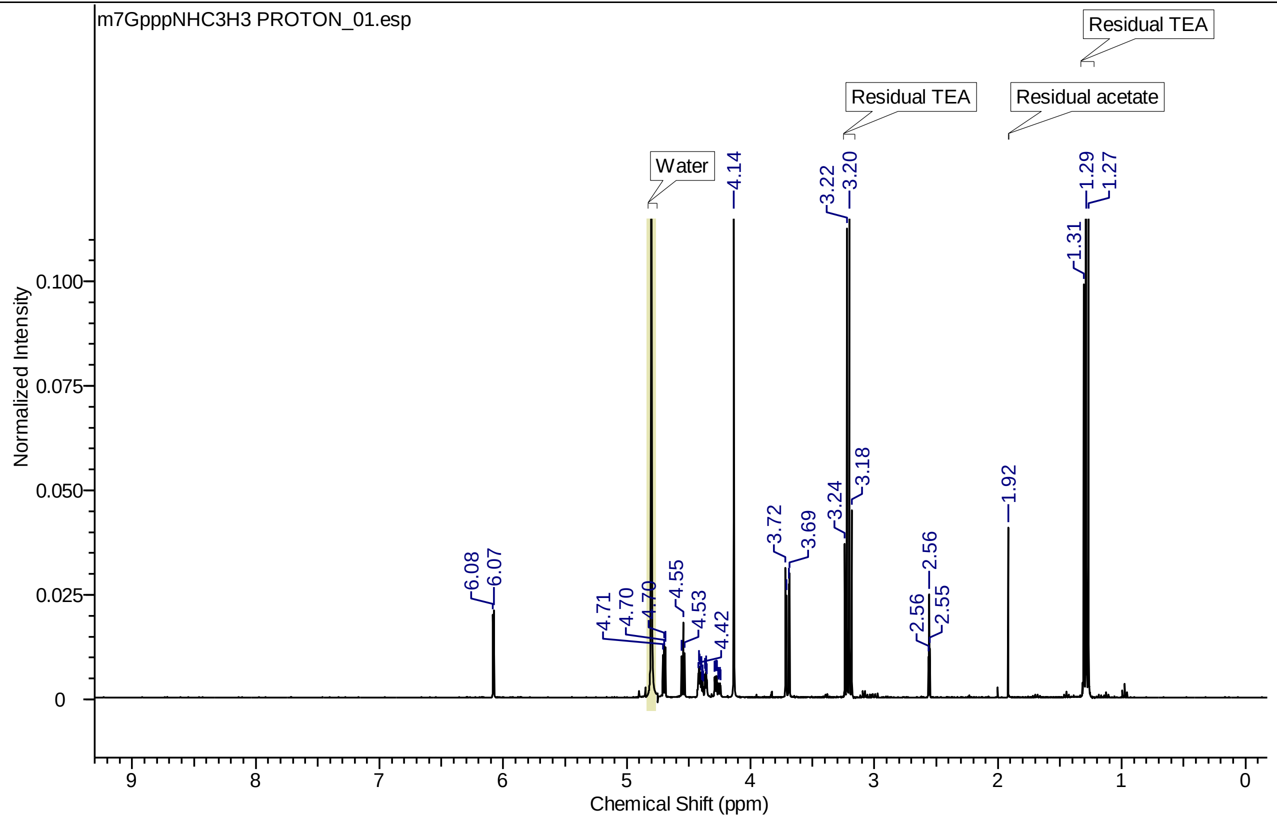
^{31}P NMR	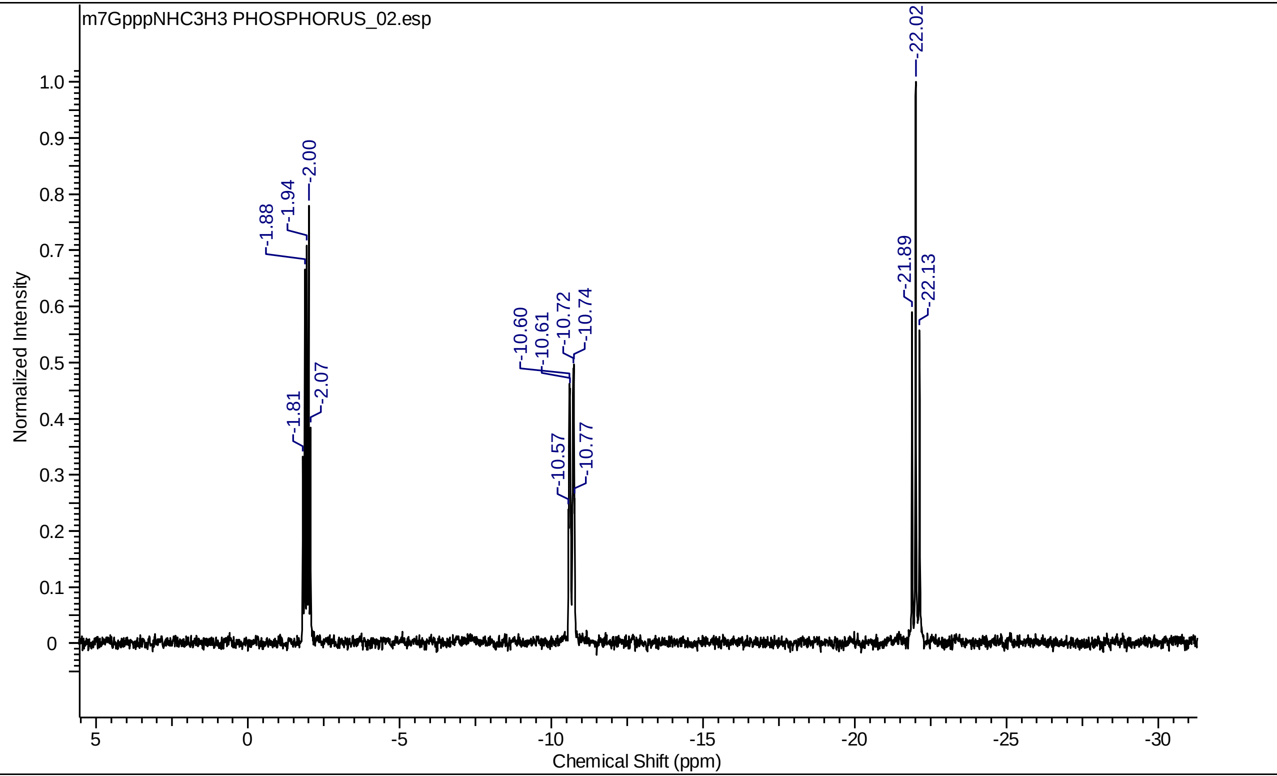

(16a) 5'-N_3-Guo

Structure	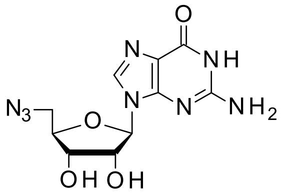

Reaction HPLC profile

Program B; before and after the reaction

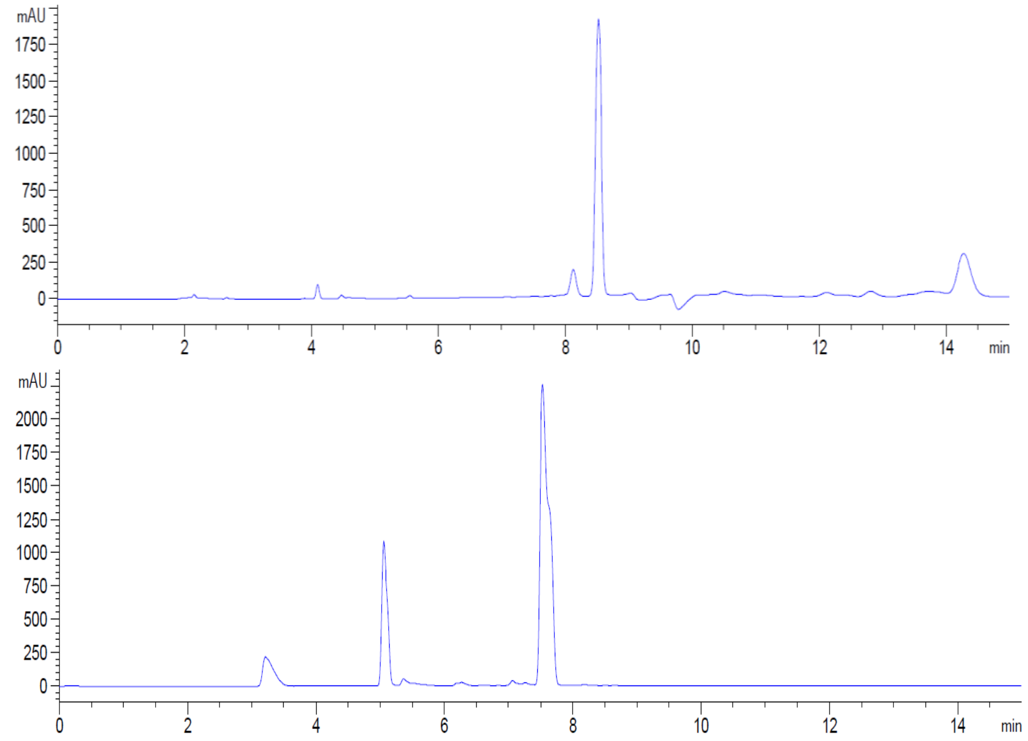

Purified compound HPLC profile

Program B

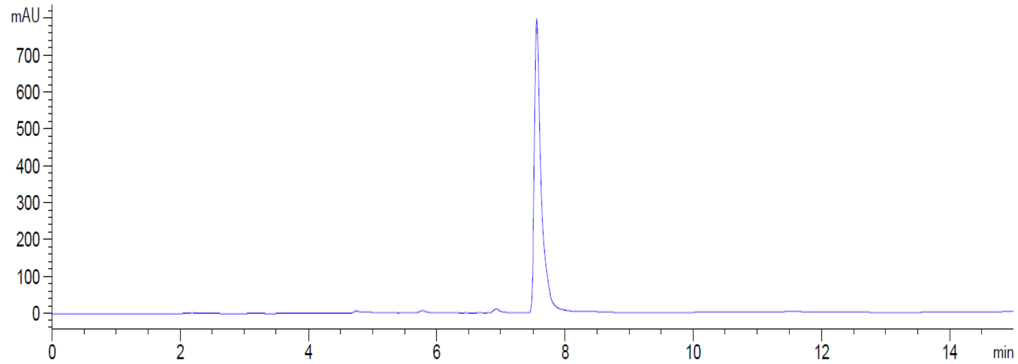

HRMS

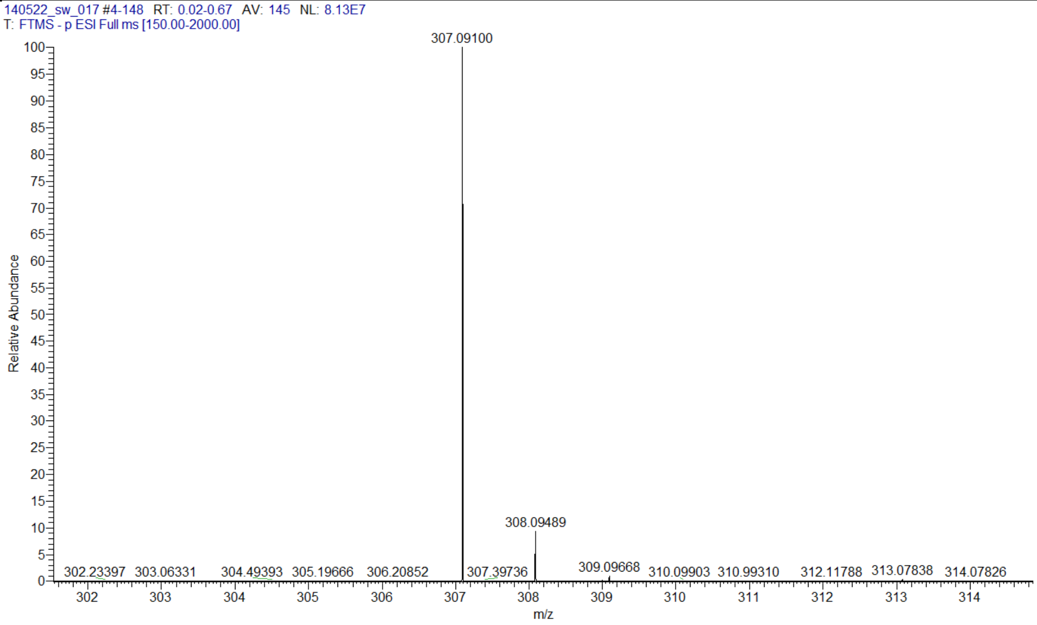

^{1}H NMR

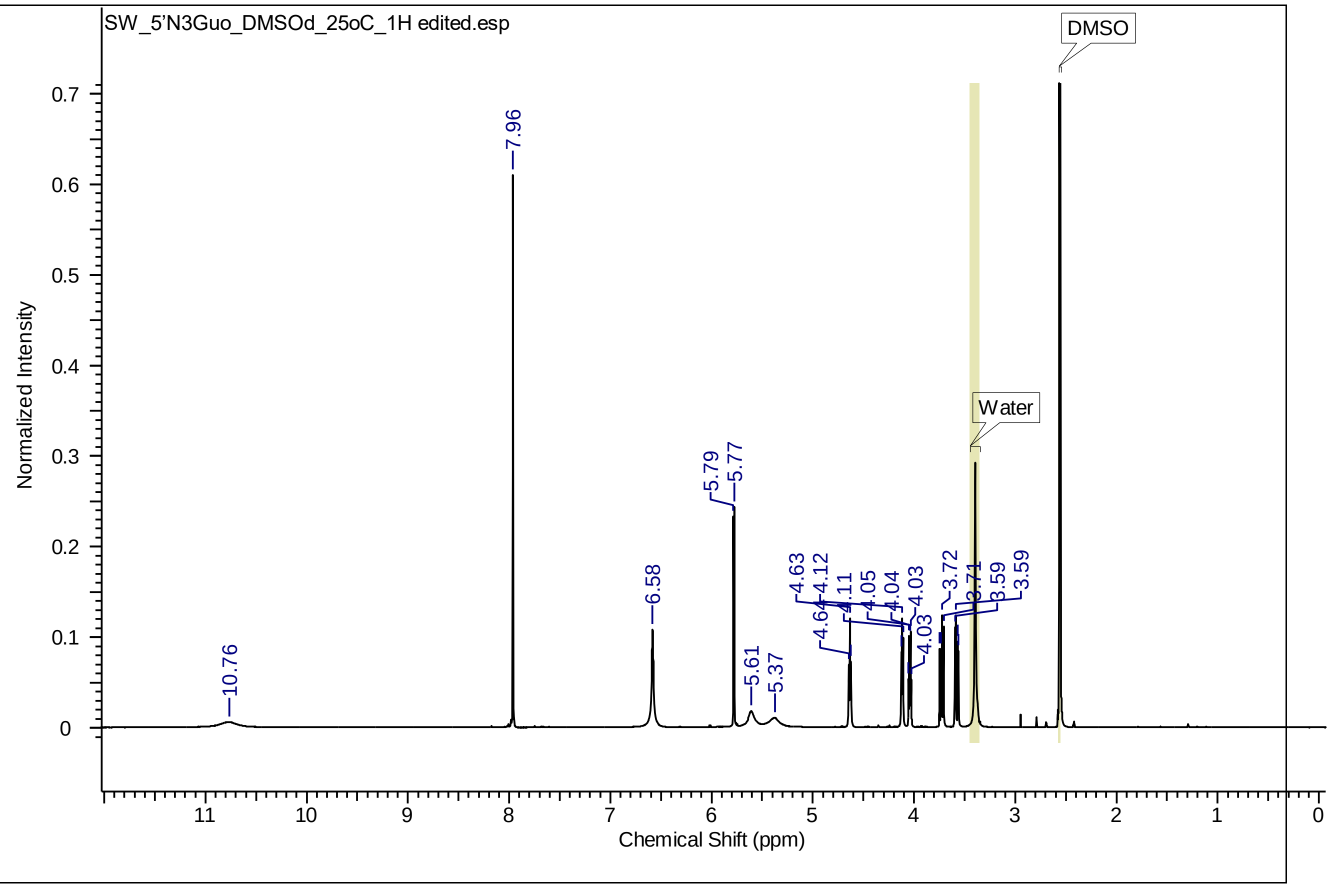

(16b) 5'-N_3-m[7]Guo

Structure

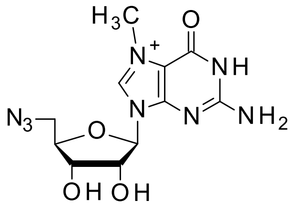

Reaction HPLC profile

Program B

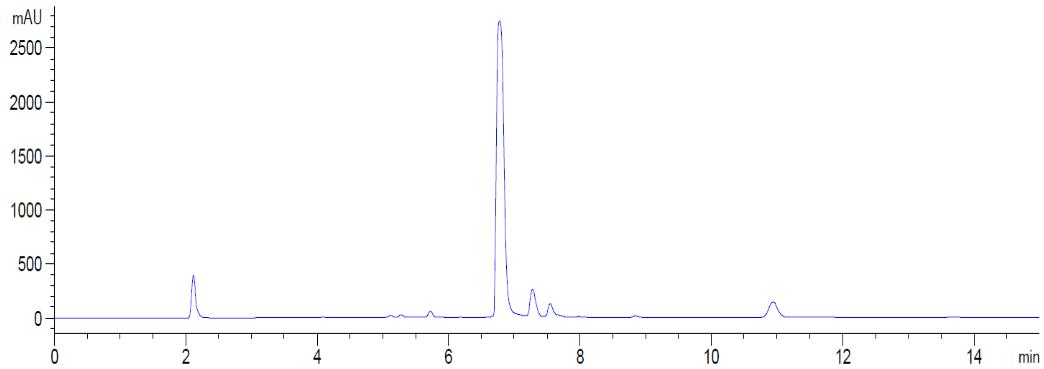

Purified compound HPLC profile

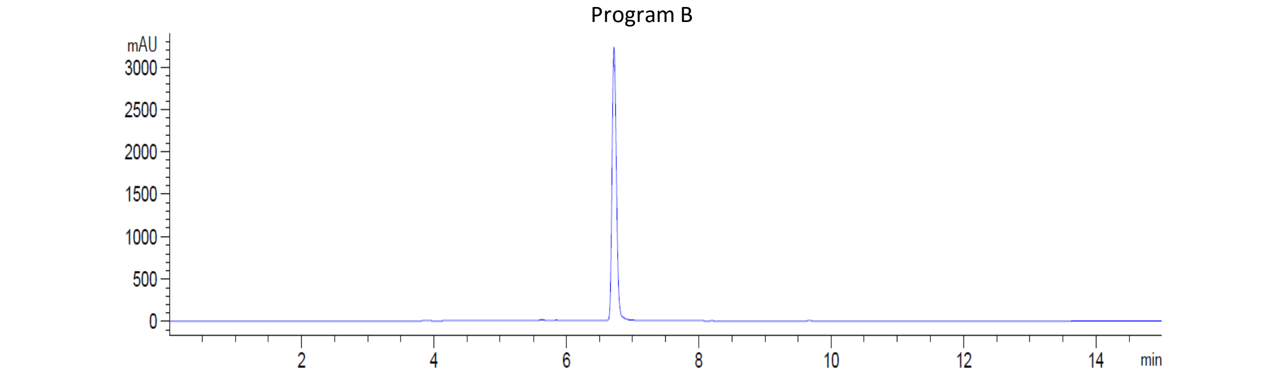

HRMS

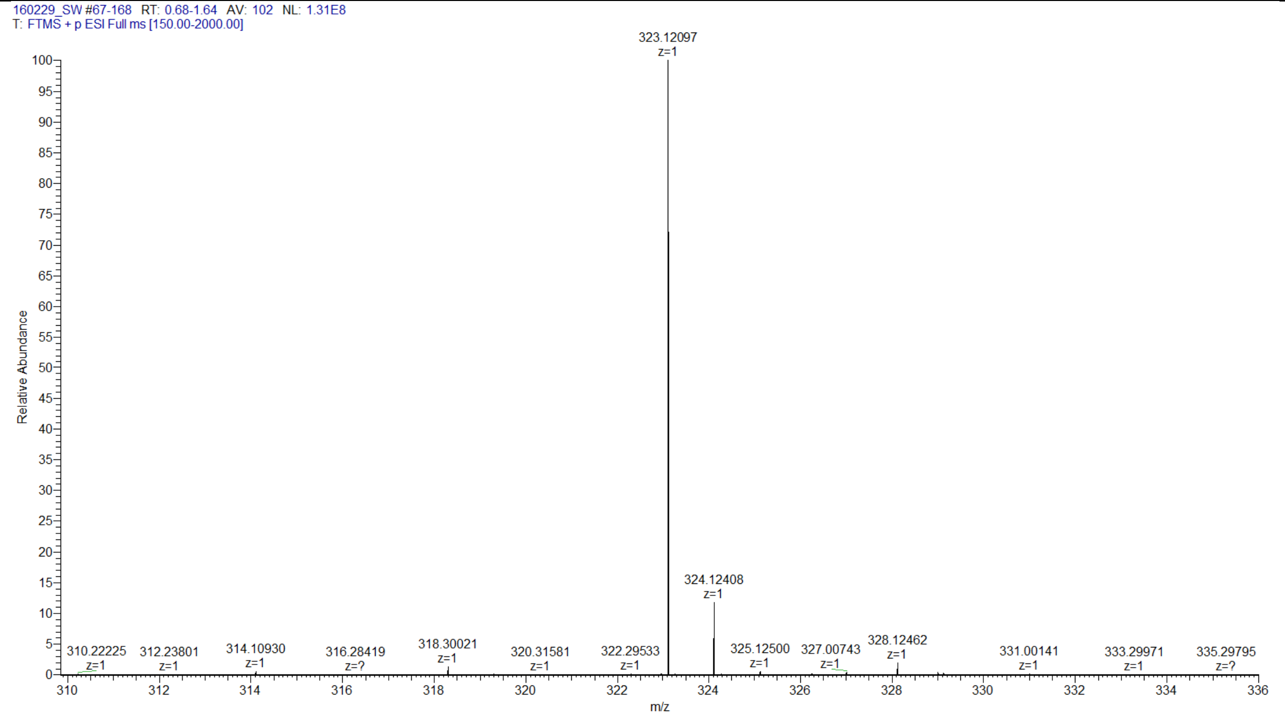

^{1}H NMR

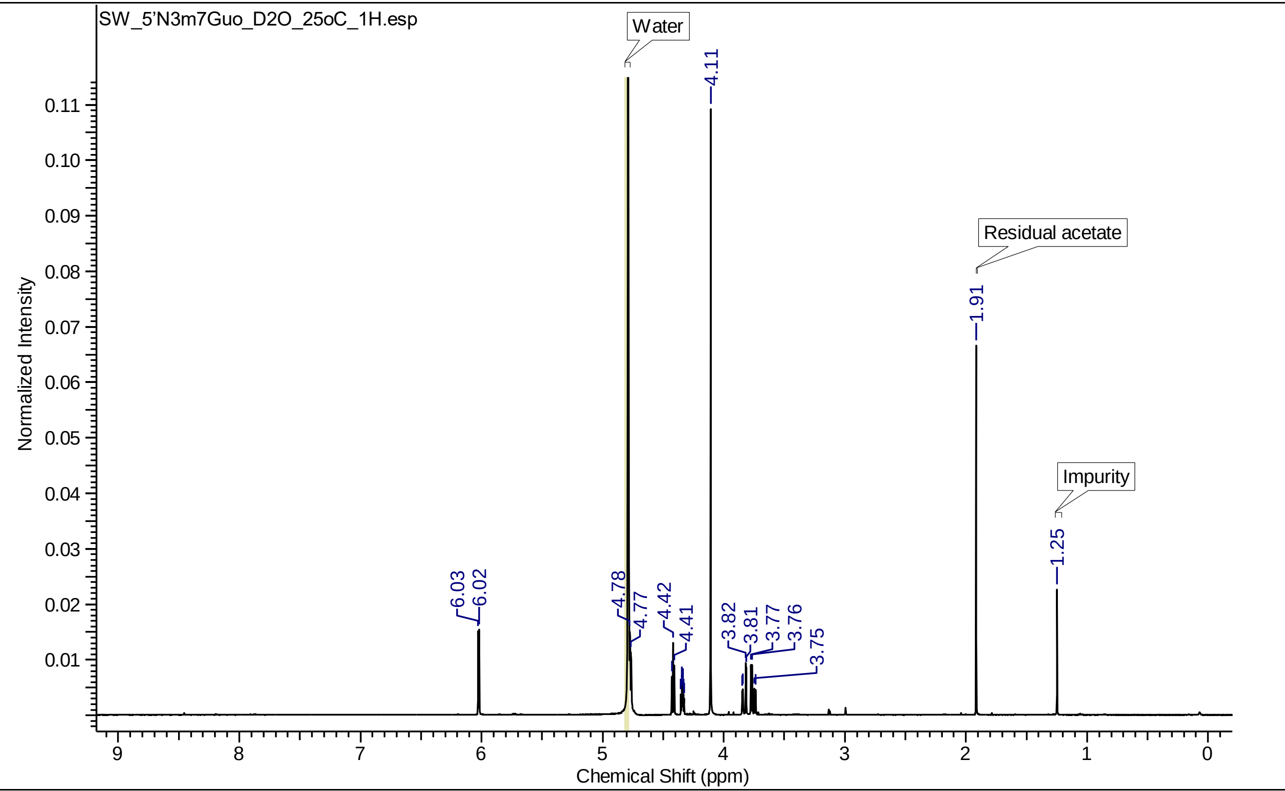

(17a) GpNHC$_2$H$_4$N$_3$

Structure

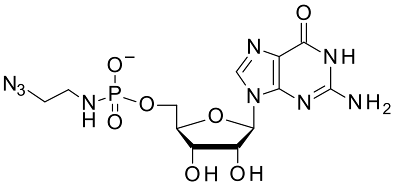

Reaction HPLC profile

Program A

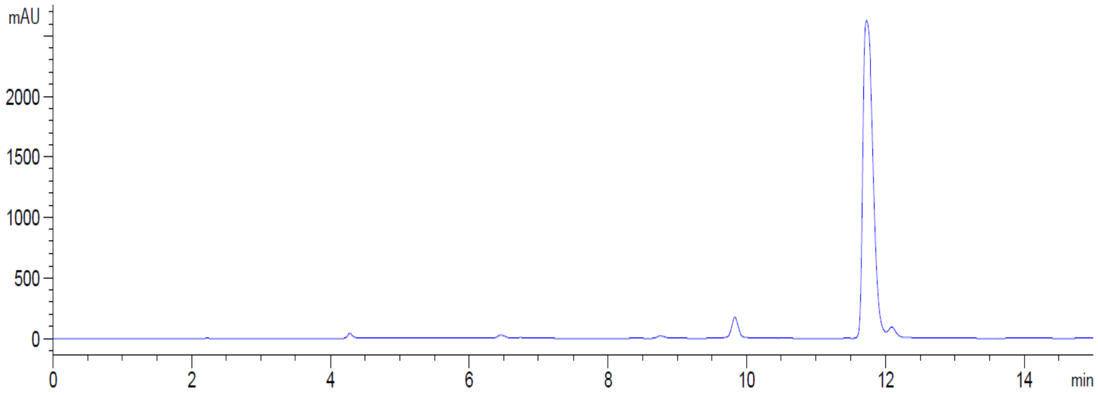

Purified compound HPLC profile

Program B

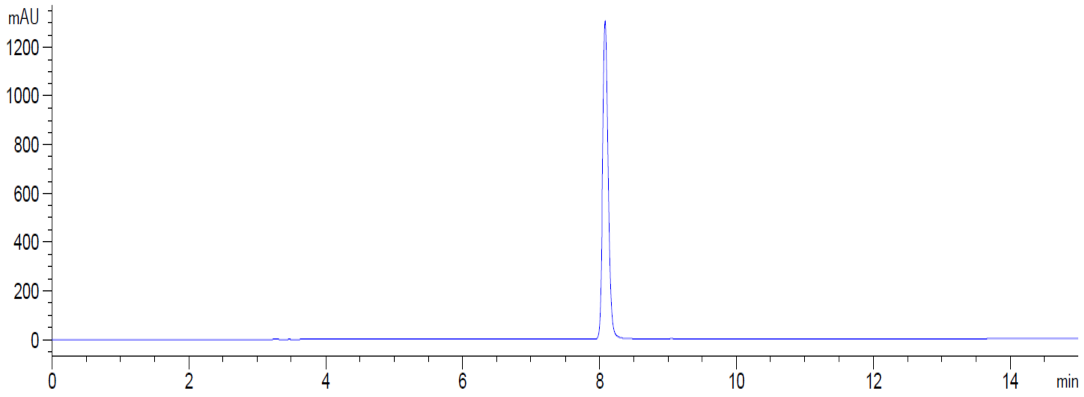

HRMS

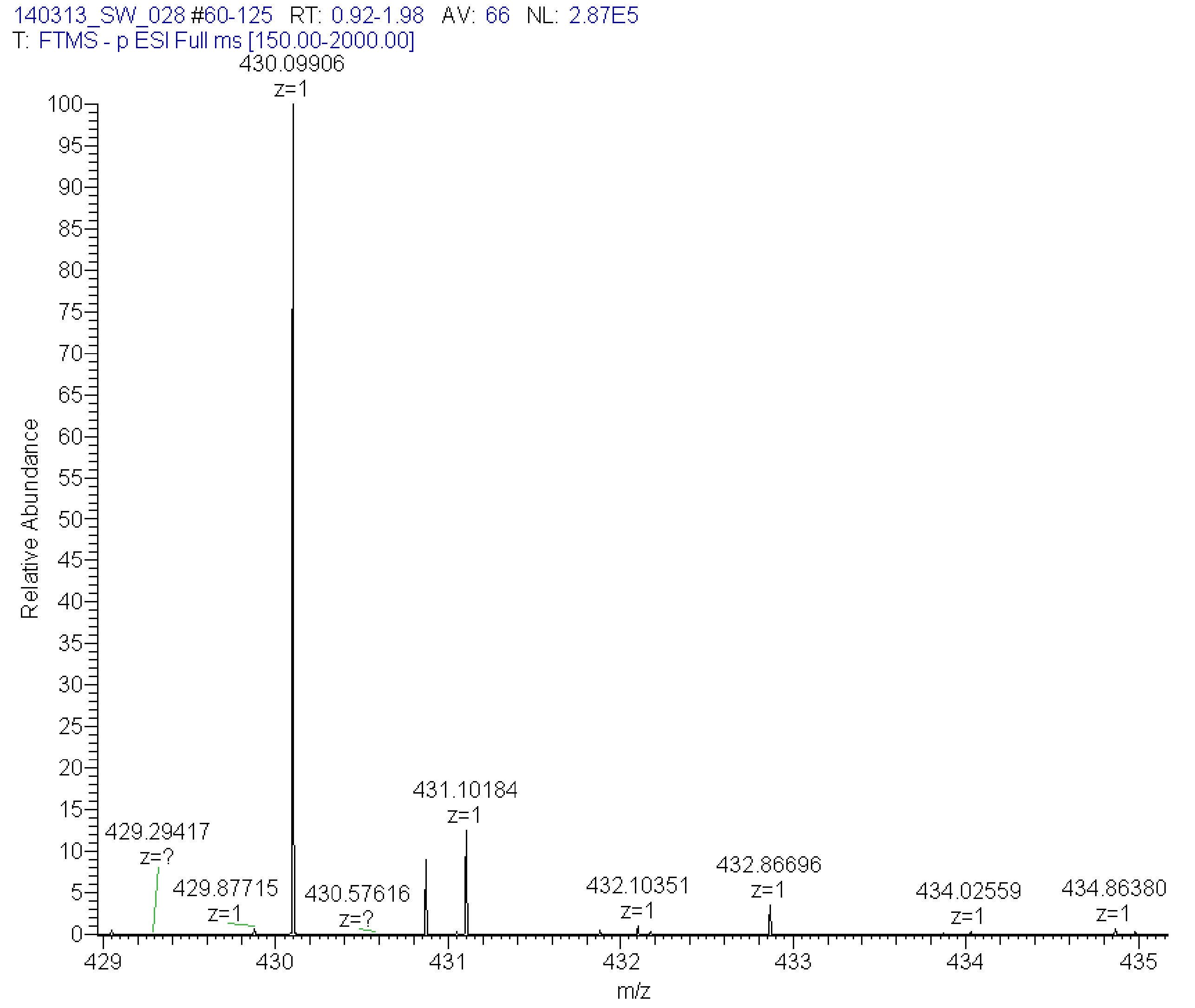
140313_SW_028 #60-125 RT: 0.92-1.98 AV: 66 NL: 2.87E5
T: FTMS - p ESI Full ms [150.00-2000.00]
430.09906
z=1
431.10184
z=1
429.29417
z=?
429.87715
z=1
430.57616
z=?
432.10351
z=1
432.86696
z=1
434.02559
z=1
434.86380
z=1
Relative Abundance
m/z

^{1}H NMR

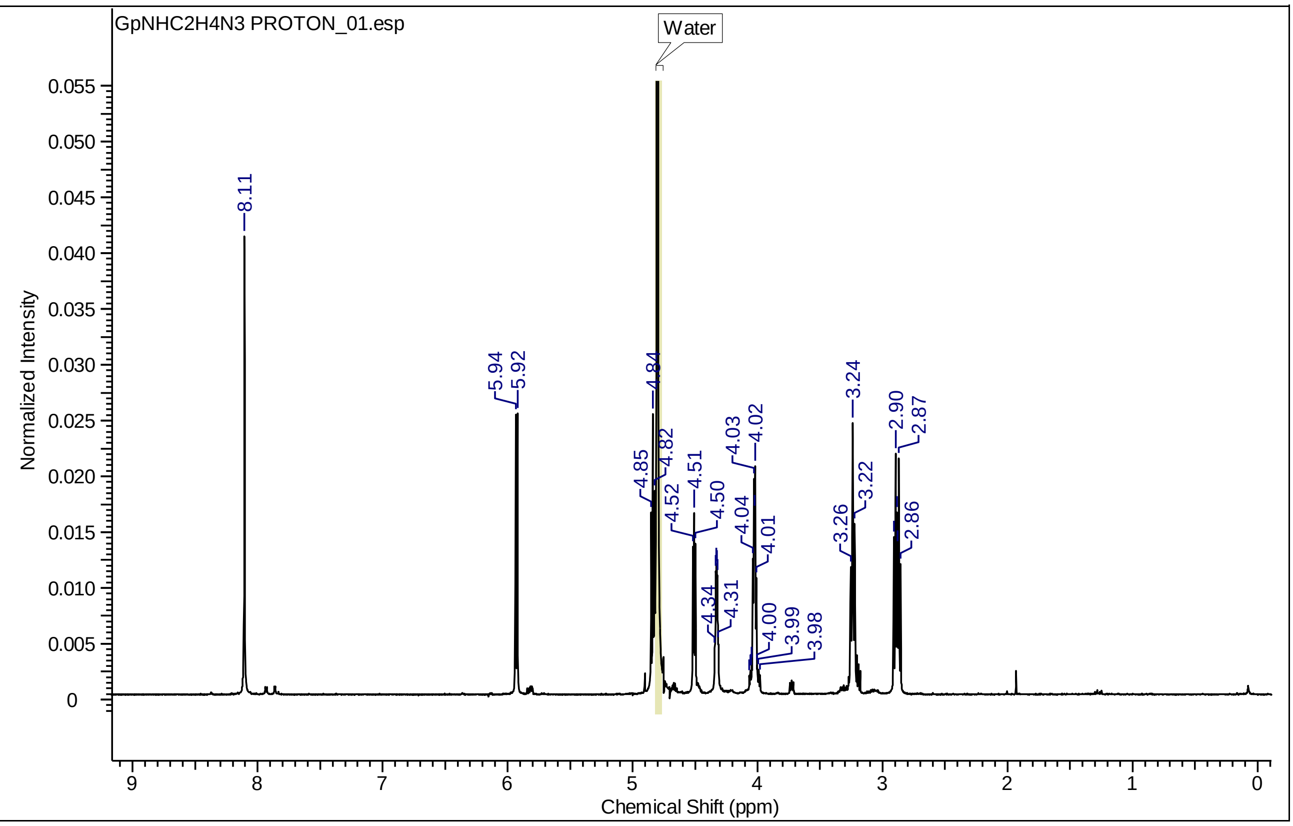
GpNHC2H4N3 PROTON_01.esp
Water
8.11
5.94
5.92
4.85
4.84
4.82
4.52
4.51
4.50
4.34
4.31
4.04
4.03
4.02
4.01
4.00
3.99
3.98
3.26
3.24
3.22
2.90
2.87
2.86
Normalized Intensity
Chemical Shift (ppm)

^{31}P NMR	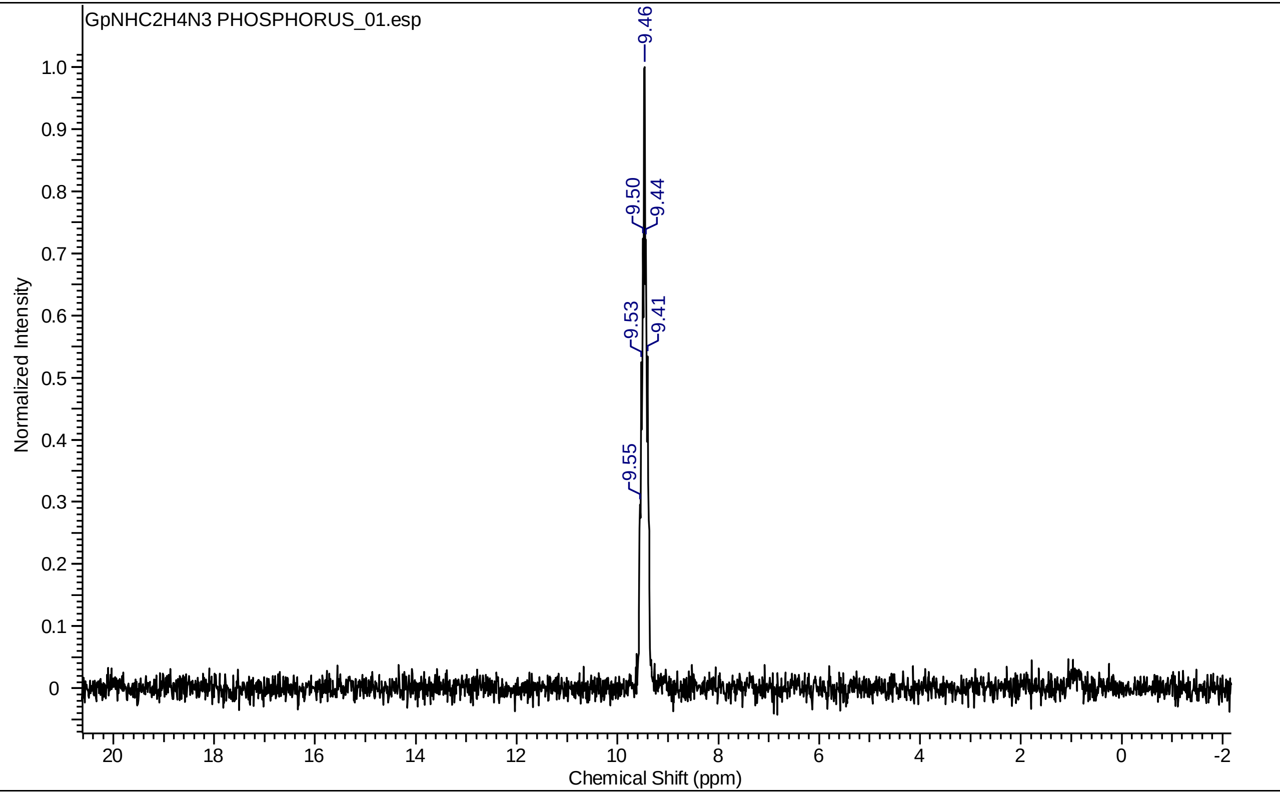

(17b) GppNHC$_2$H$_4$N$_3$

Structure	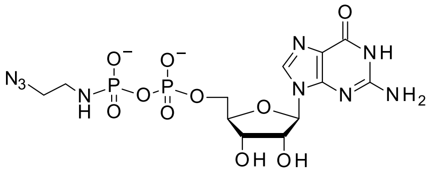
Reaction HPLC profile	Program A 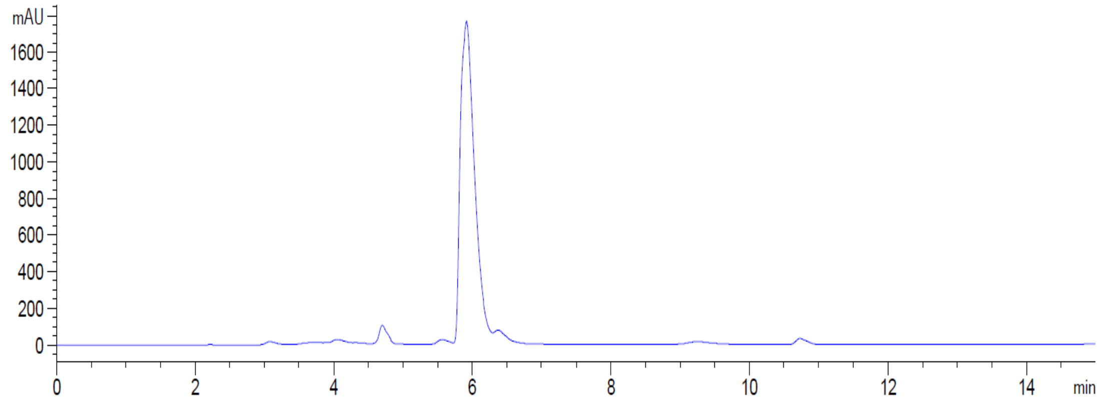
Purified compound HPLC profile	Program A 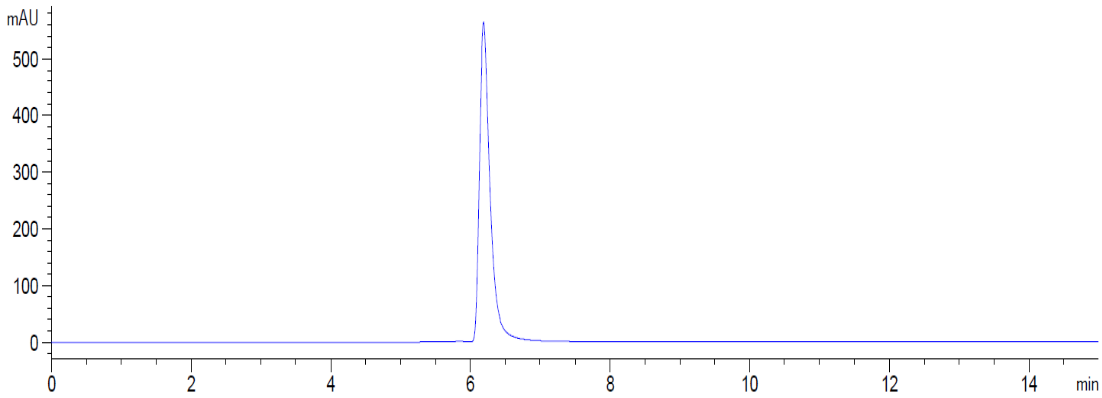

HRMS

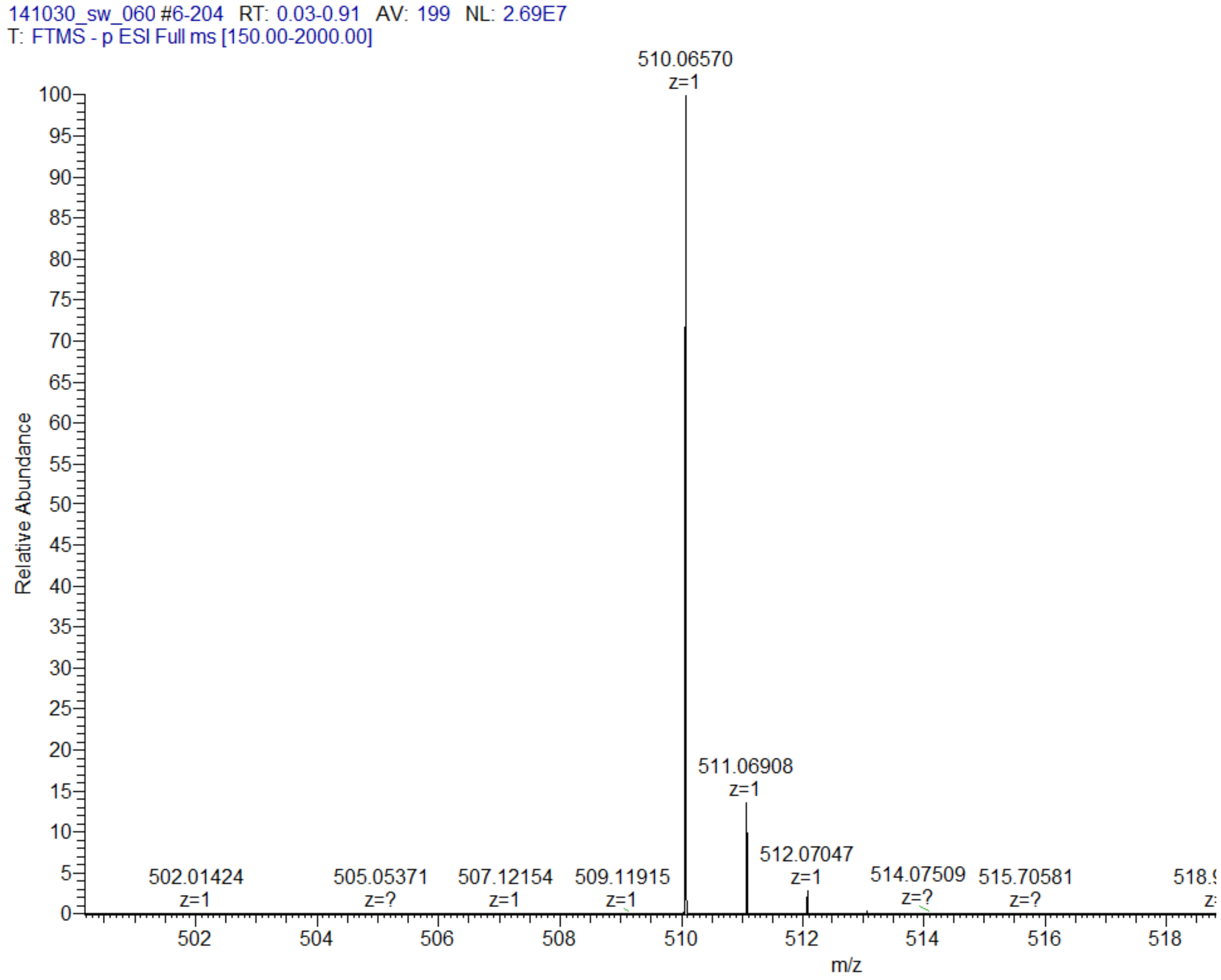

141030_sw_060 #6-204 RT: 0.03-0.91 AV: 199 NL: 2.69E7
T: FTMS - p ESI Full ms [150.00-2000.00]
510.06570 z=1
511.06908 z=1
512.07047 z=1
502.01424 z=1
505.05371 z=?
507.12154 z=1
509.11915 z=1
514.07509 z=?
515.70581 z=?
Relative Abundance
m/z

^{1}H NMR

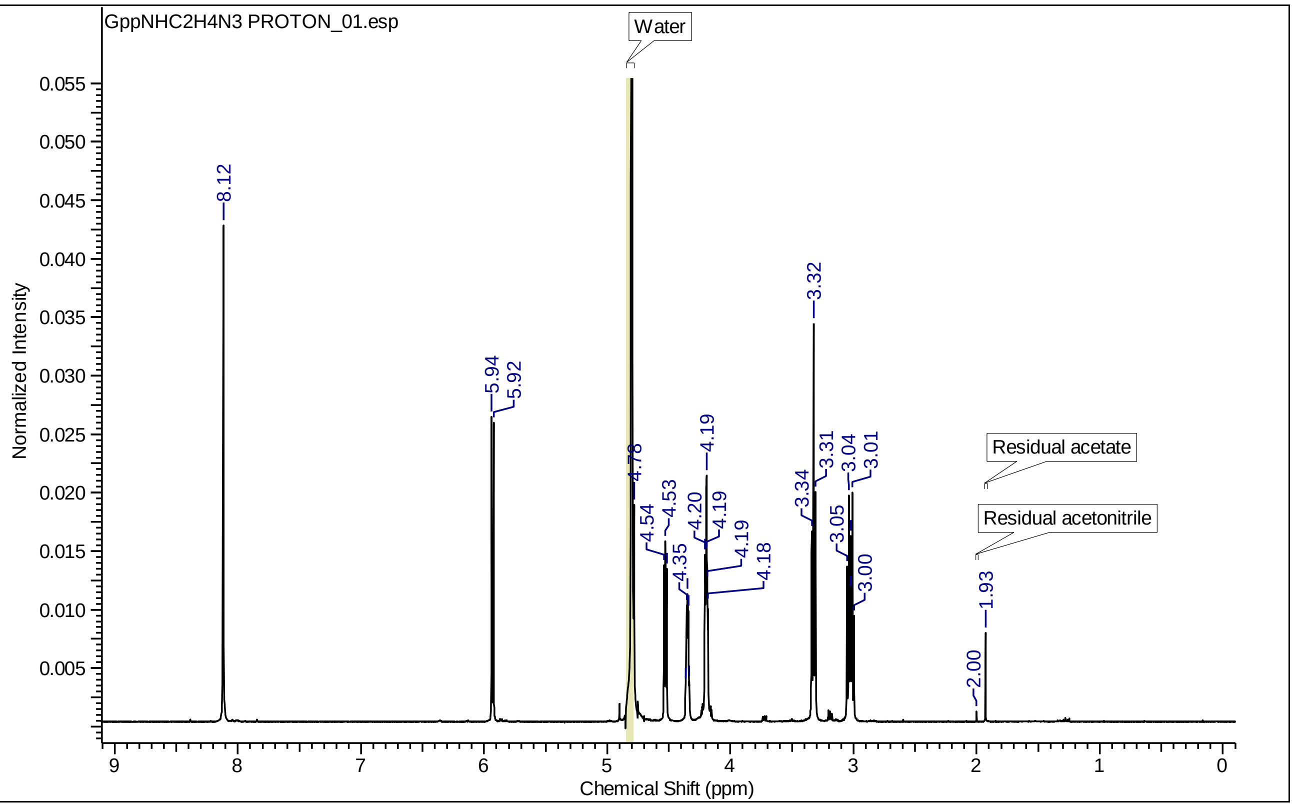

GppNHC2H4N3 PROTON_01.esp
Water
Residual acetate
Residual acetonitrile
8.12
5.94
5.92
4.78
4.54
4.53
4.35
4.20
4.19
4.19
4.19
4.18
3.34
3.32
3.31
3.05
3.04
3.01
3.00
2.00
1.93
Normalized Intensity
Chemical Shift (ppm)

^{31}P NMR

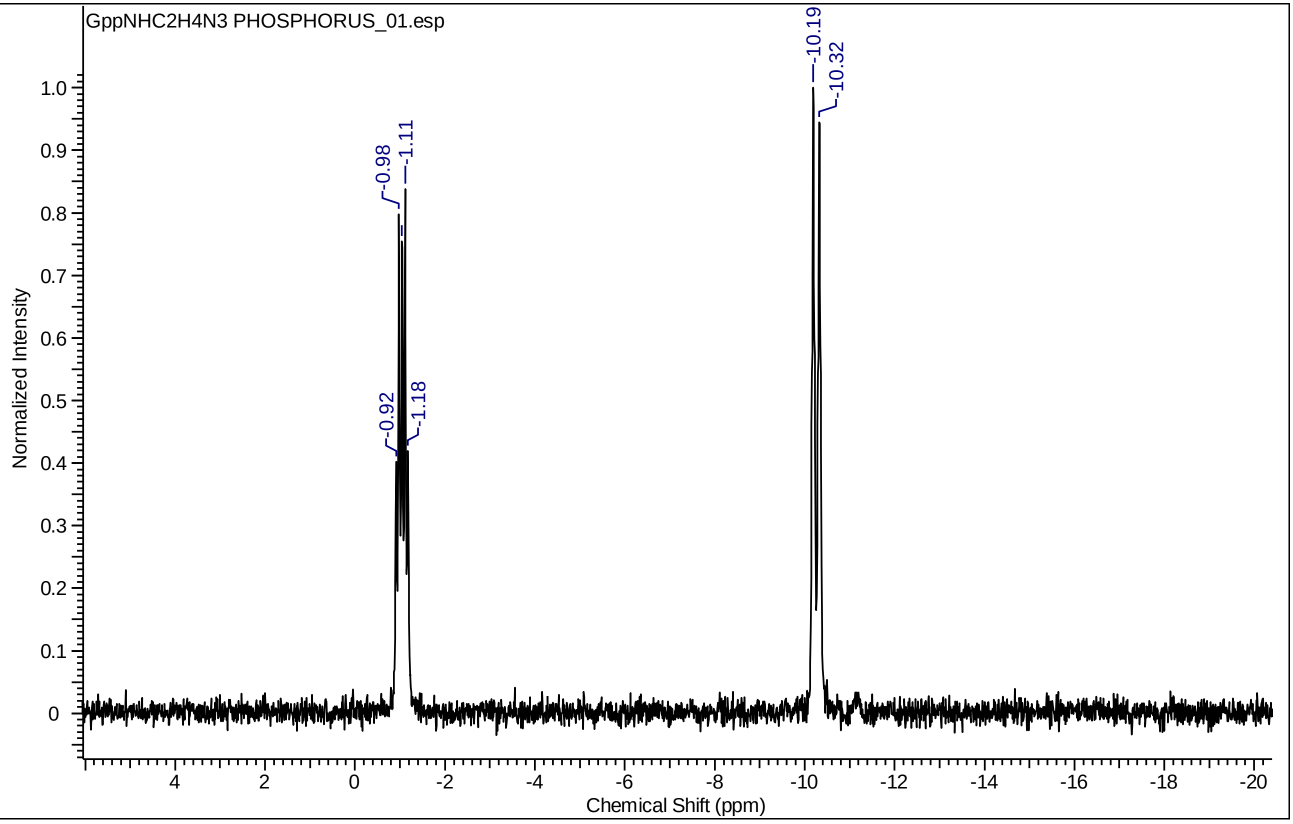

(17c) m^{7}GpNHC$_2$H$_4$N$_3$

Structure

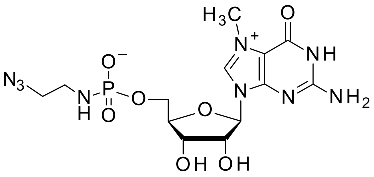

Reaction HPLC profile

Program B

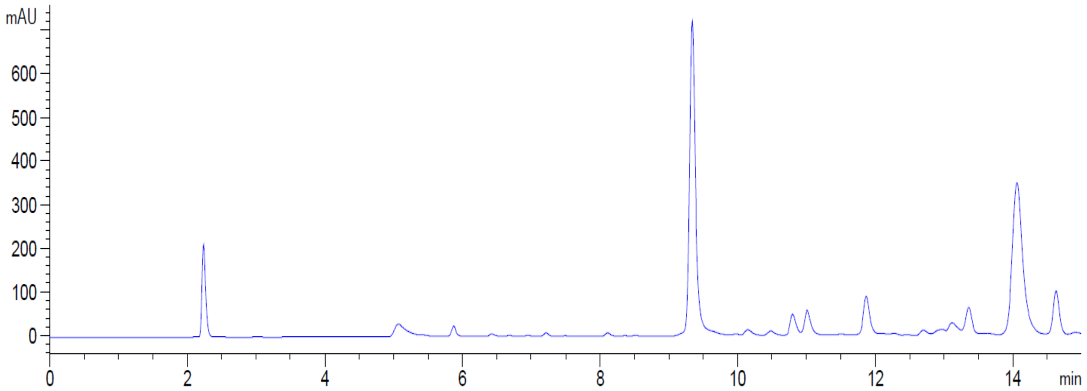

Purified compound HPLC profile

Program B

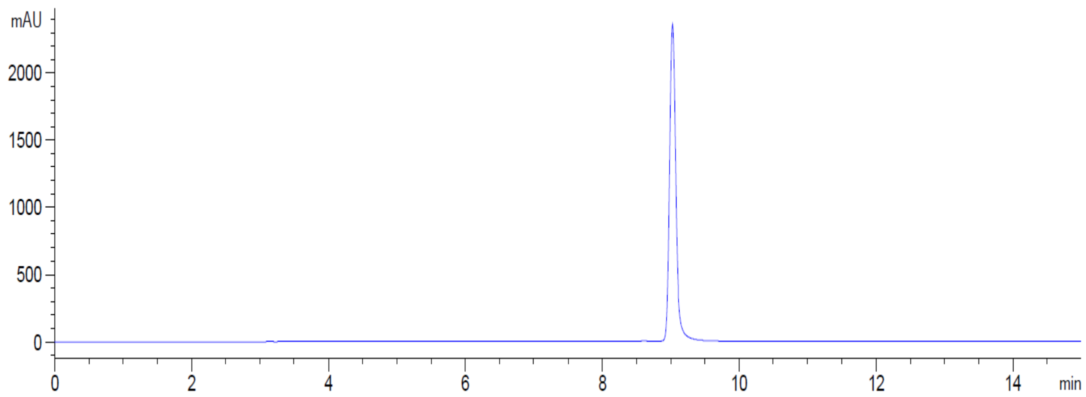

HRMS

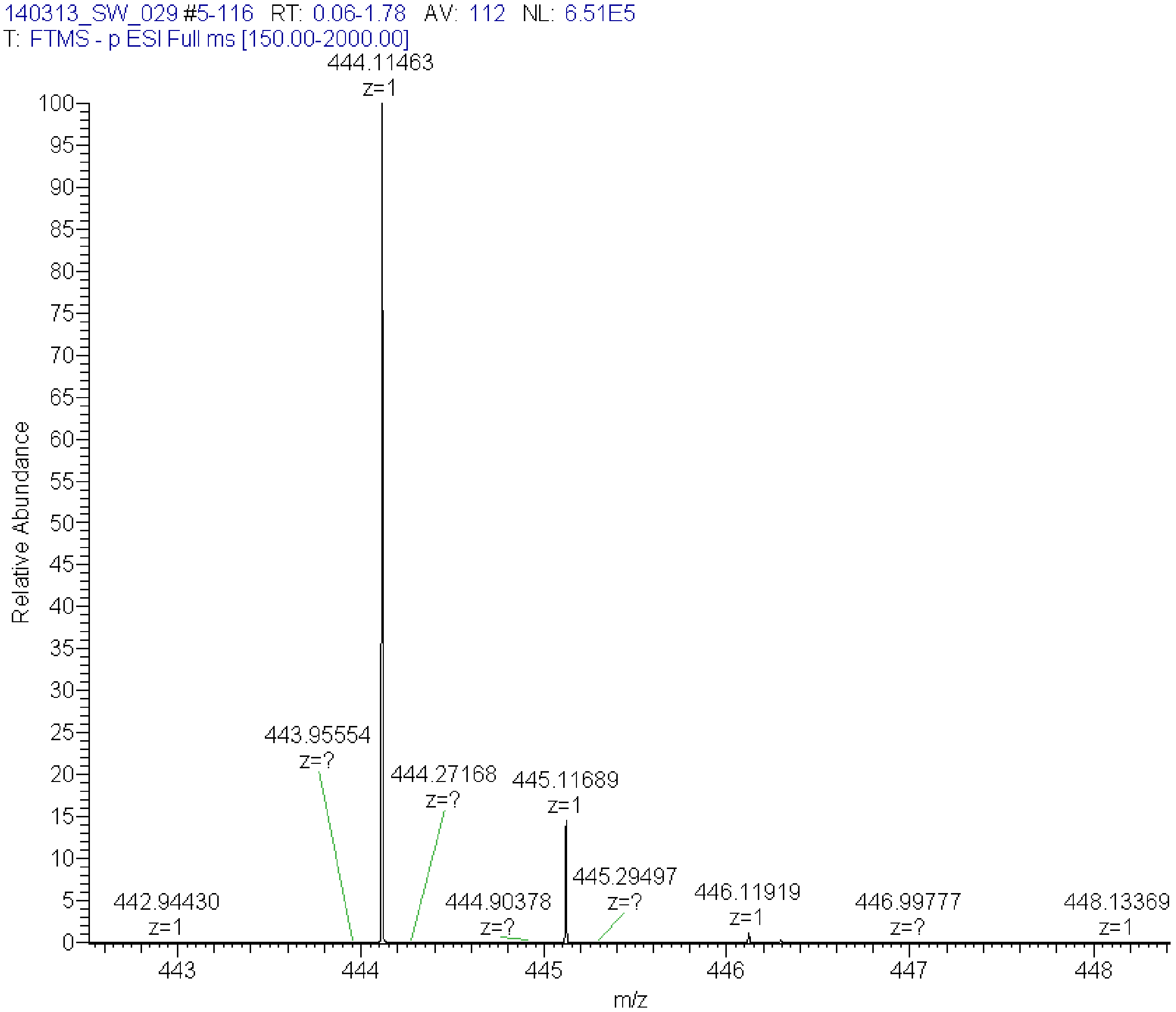

^{1}H NMR

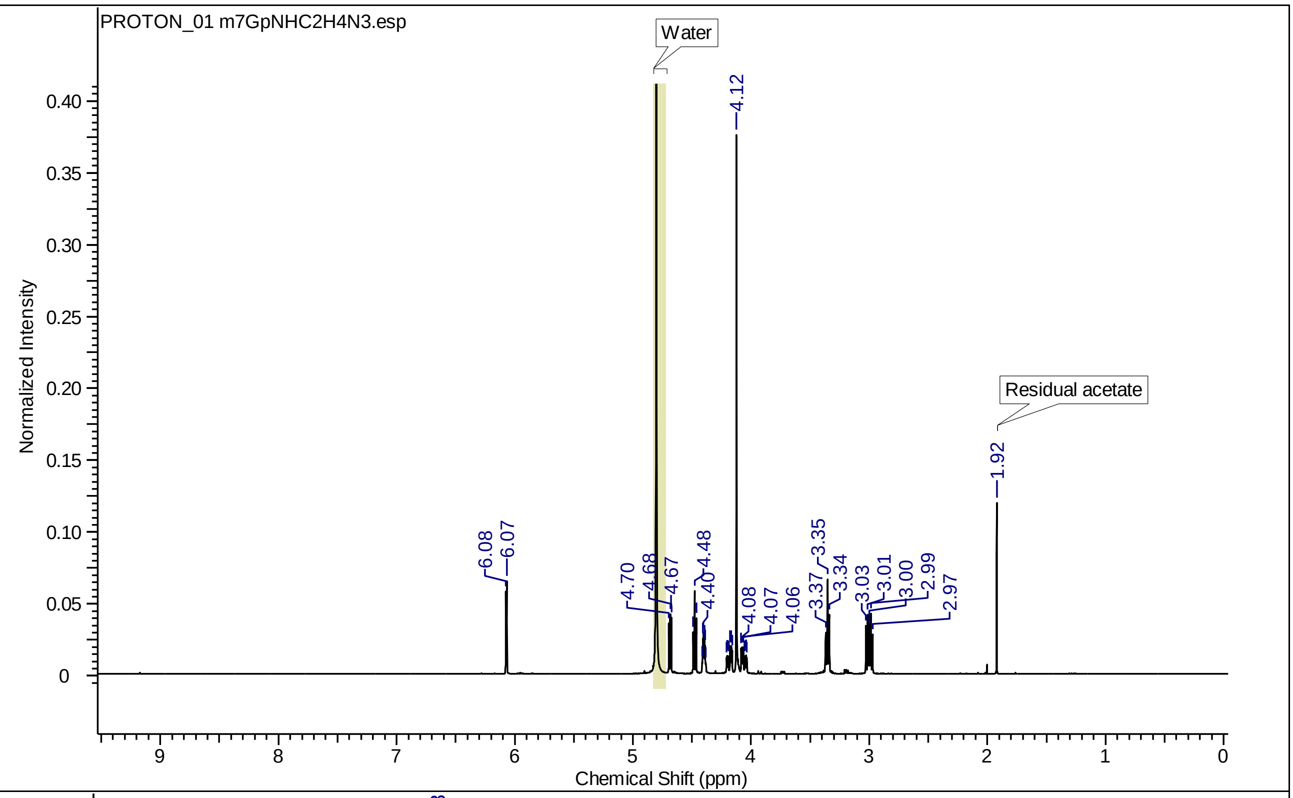

^{31}P NMR

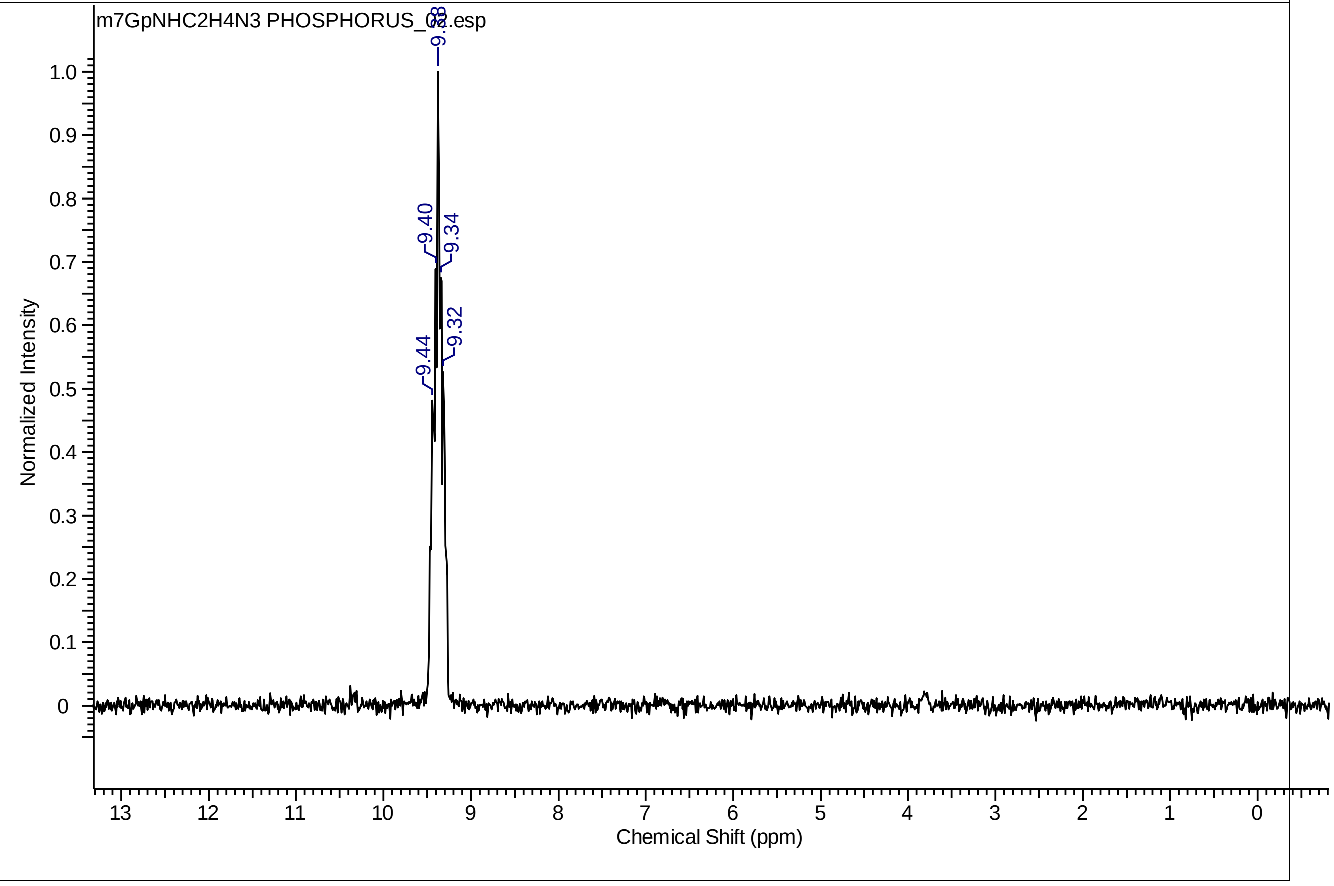

(17d) $m^{7}GppNHC_2H_4N_3$

Structure

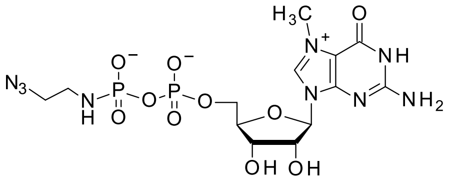

**Reaction HPLC profile**	Program A 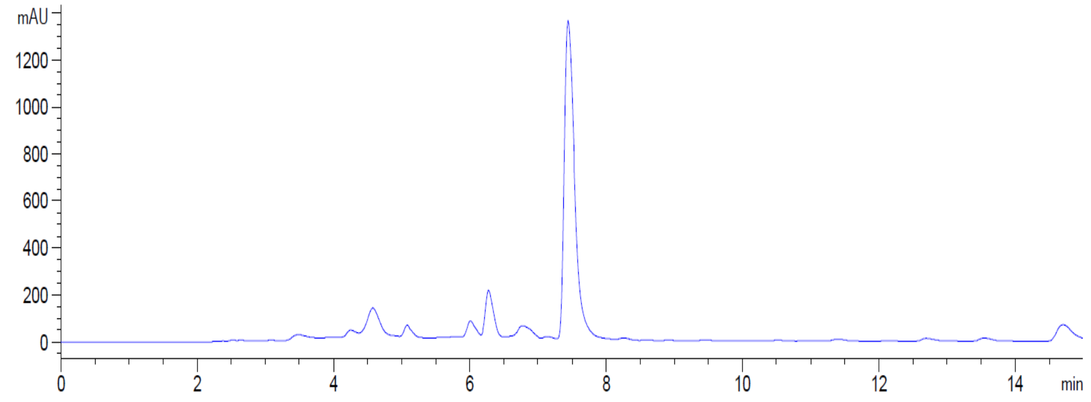
**Purified compound HPLC profile**	Program A 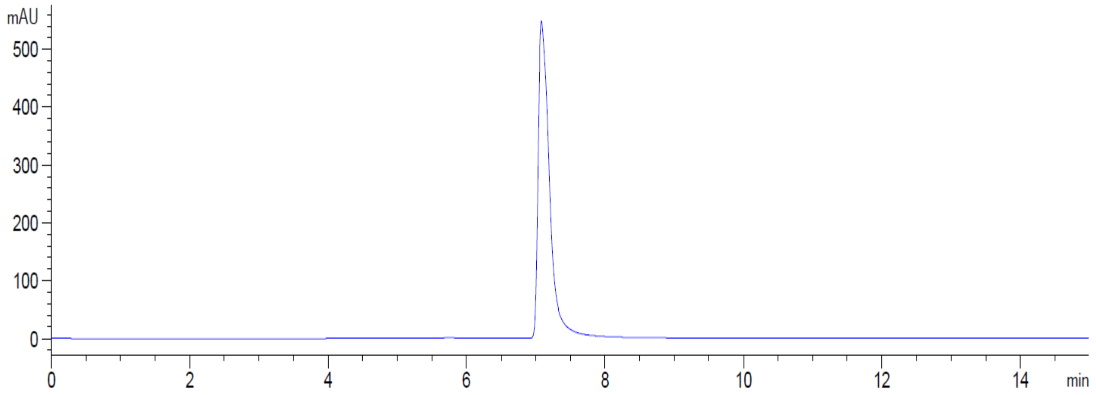
**HRMS**	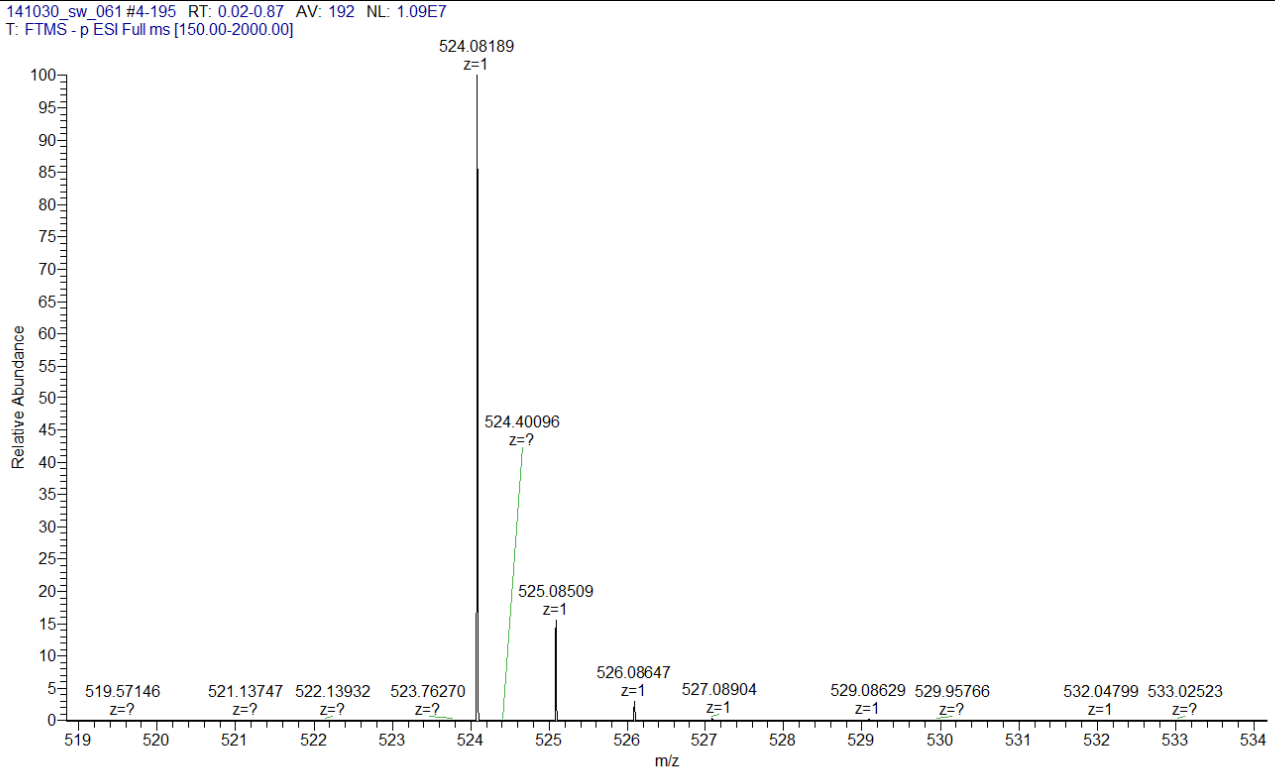

^{1}H NMR	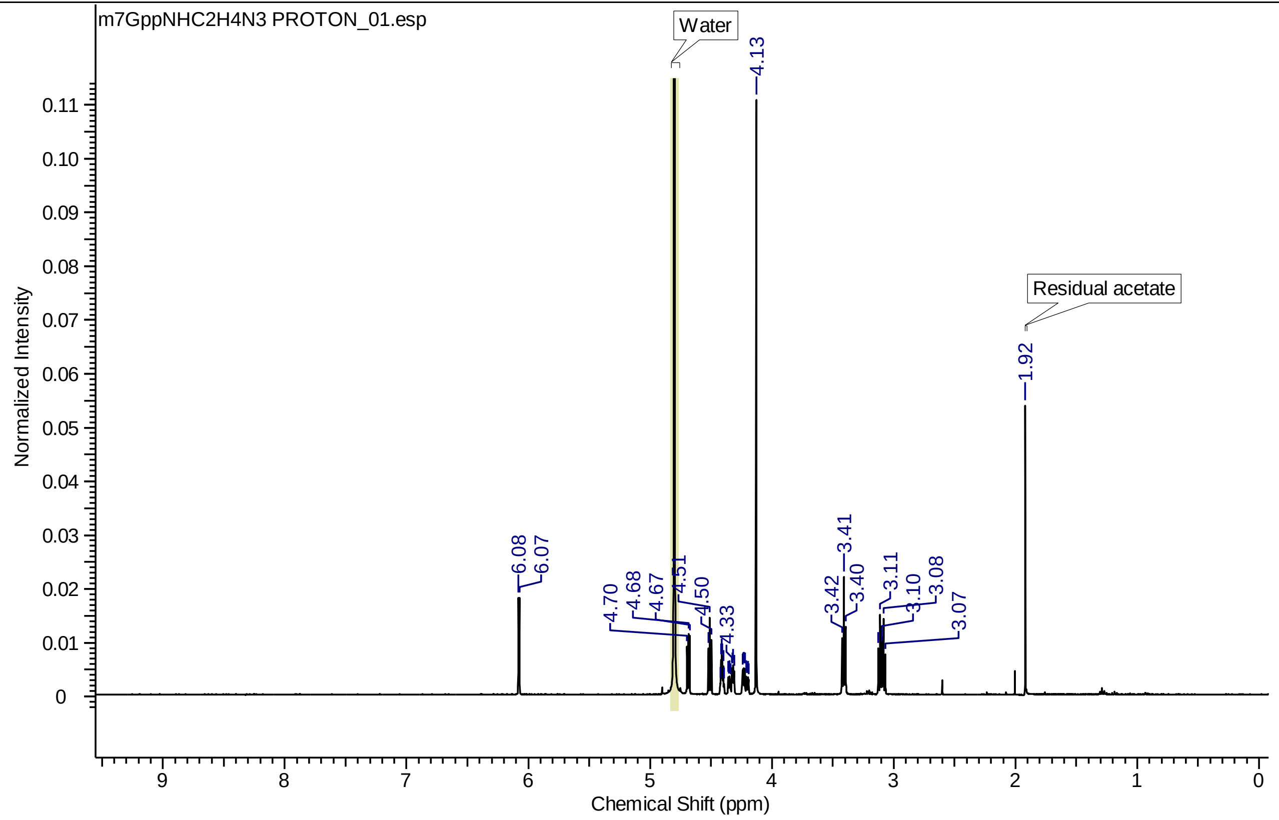
^{31}P NMR	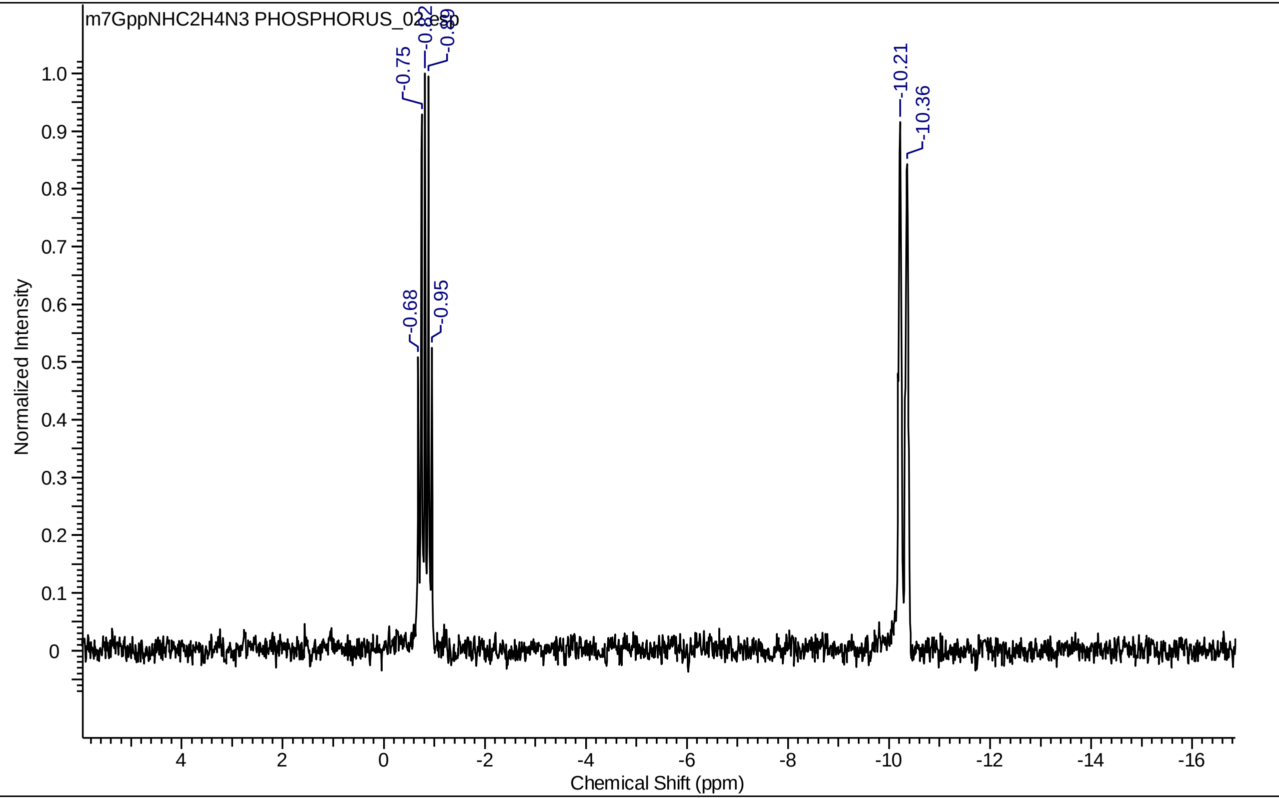

(18d) O-(2-propynyl) phosphate ester

Structure	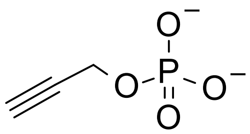

HRMS

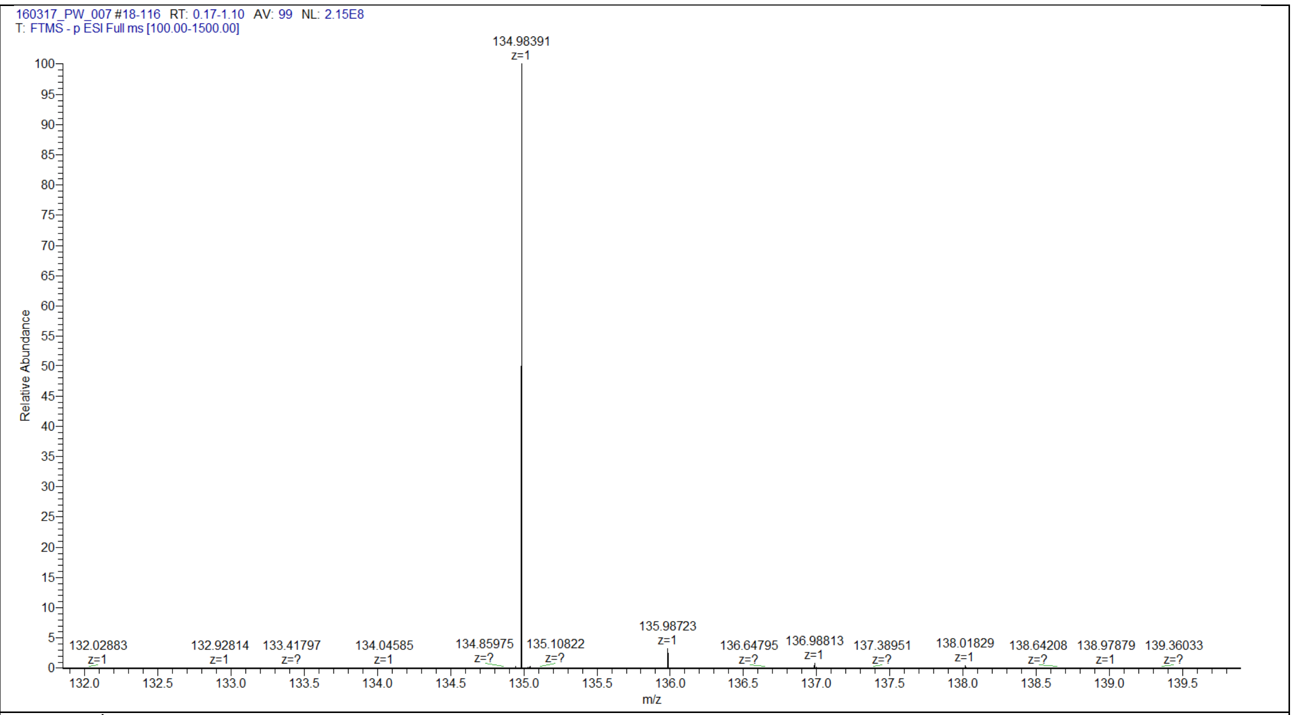
160317_PW_007 #18-116 RT: 0.17-1.10 AV: 99 NL: 2.15E8
T: FTMS - p ESI Full ms [100.00-1500.00]
134.98391
z=1
Relative Abundance
135.98723
z=1
132.02883
132.92814
133.41797
134.04585
134.85975
135.10822
136.64795
136.98813
137.38951
138.01829
138.64208
138.97879
139.36033
m/z

^{1}H NMR

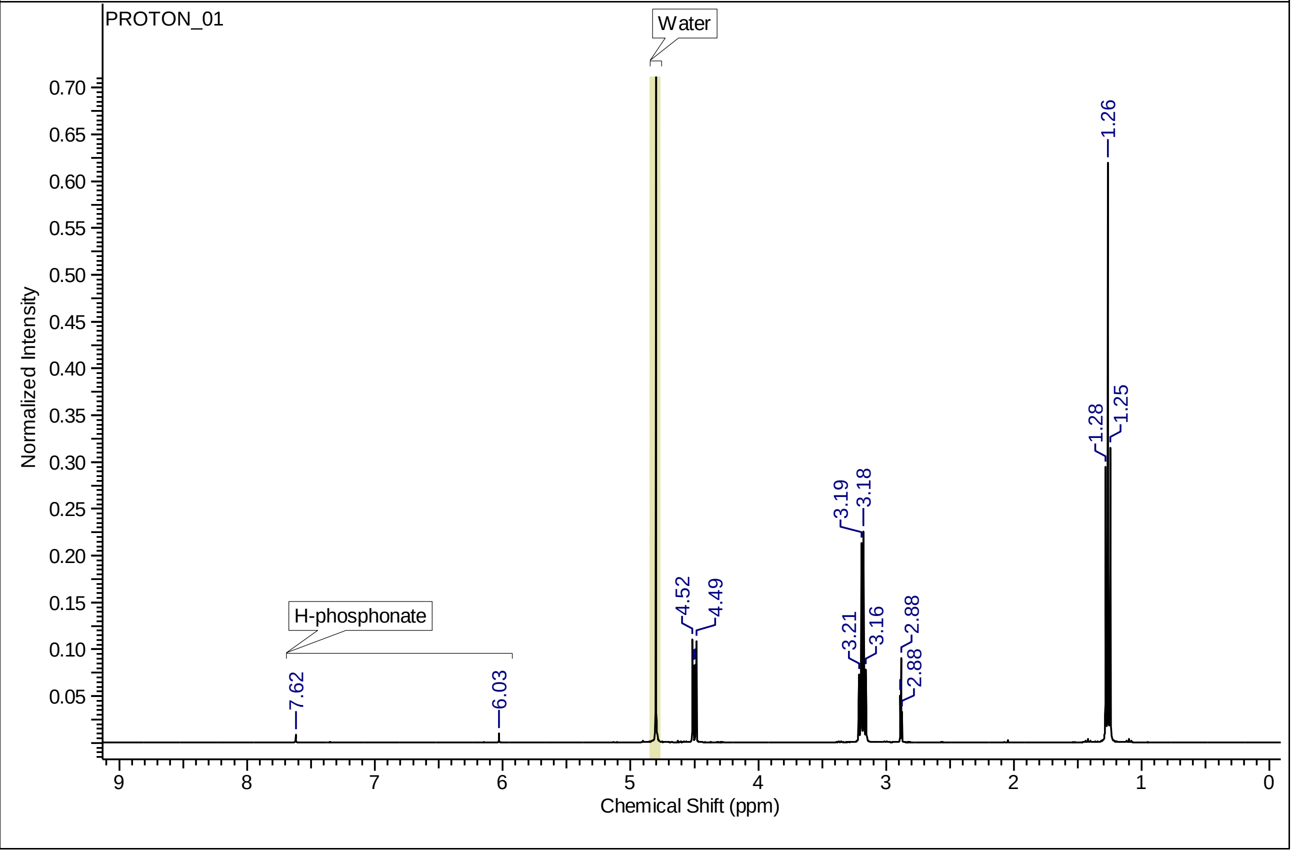
PROTON_01
Water
H-phosphonate
7.62
6.03
4.52
4.49
3.21
3.19
3.18
3.16
2.88
2.88
1.28
1.26
1.25
Normalized Intensity
Chemical Shift (ppm)

^{31}P NMR

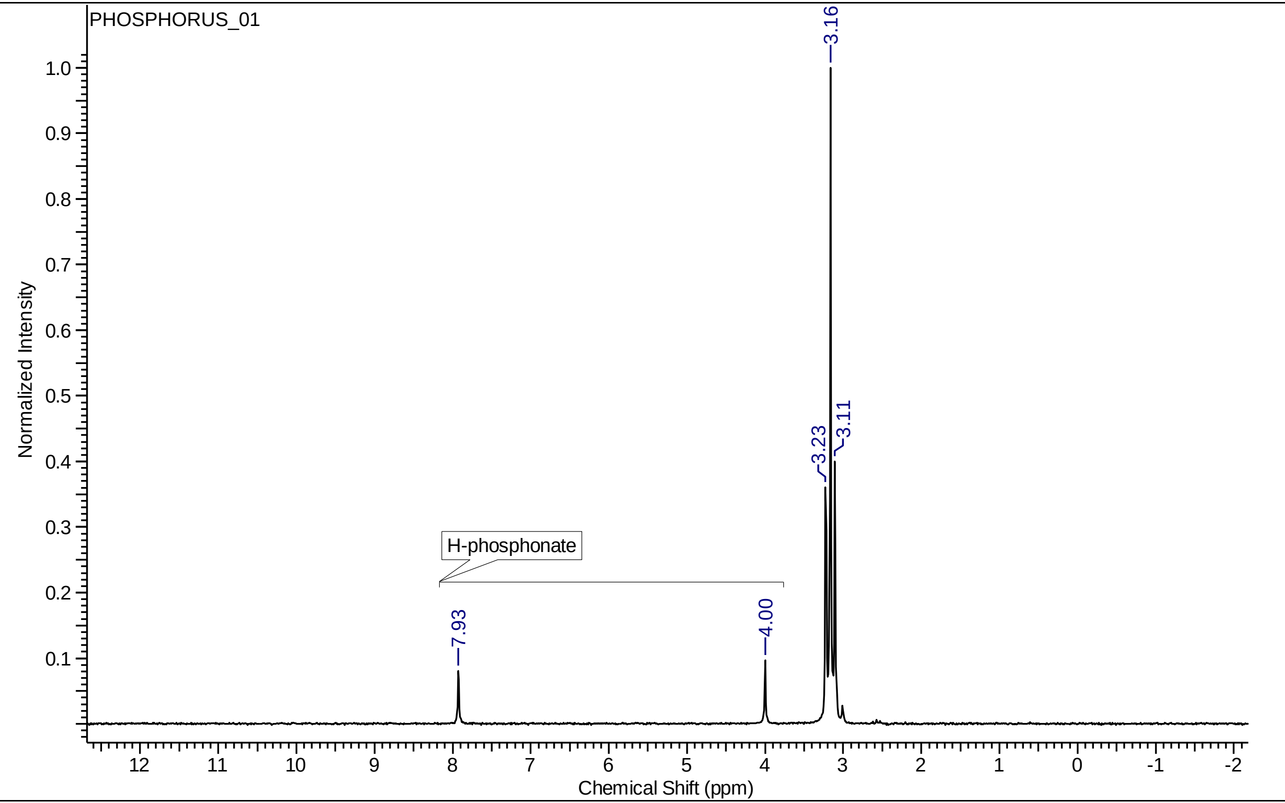
PHOSPHORUS_01
Normalized Intensity
Chemical Shift (ppm)
H-phosphonate
7.93
4.00
3.23
3.16
3.11
